# The m6A demethylase FTO promotes C/EBPβ‐LIP translation to perform oncogenic functions in breast cancer cells

**DOI:** 10.1111/febs.70033

**Published:** 2025-02-28

**Authors:** Hidde R. Zuidhof, Christine Müller, Gertrud Kortman, René Wardenaar, Ekaterina Stepanova, Fabricio Loayza‐Puch, Cornelis F. Calkhoven

**Affiliations:** ^1^ European Institute for the Biology of Ageing (ERIBA) University Medical Center Groningen, University of Groningen The Netherlands; ^2^ Translational Control and Metabolism German Cancer Research Center (DKFZ) Heidelberg Germany

**Keywords:** breast cancer, C/EBPβ, FTO, mRNA translation

## Abstract

N6‐methyladenosine (m6A) is a prevalent posttranscriptional mRNA modification involved in the regulation of transcript turnover, translation, and other aspects of RNA fate. The modification is mediated by multicomponent methyltransferase complexes (so‐called writers) and is reversed through the action of the m6A‐demethylases fat mass and obesity‐associated (FTO) and alkB homolog 5 (ALKBH5) (so‐called erasers). FTO promotes cell proliferation, colony formation and metastasis in models of triple‐negative breast cancer (TNBC). However, little is known about genome‐wide or specific downstream regulation by FTO. Here, we examined changes in the genome‐wide transcriptome and translatome following FTO knockdown in TNBC cells. Unexpectedly, FTO knockdown had a limited effect on the translatome, while transcriptome analysis revealed that genes related to extracellular matrix (ECM) and epithelial‐mesenchymal transition (EMT) are regulated through yet unidentified mechanisms. Differential translation of CEBPB mRNA into the C/EBPβ transcription factor isoform C/EBPβ‐LIP is known to act in a pro‐oncogenic manner in TNBC cells through regulation of EMT genes. Here we show that FTO is required for efficient C/EBPβ‐LIP expression, suggesting that FTO has oncogenic functions through regulation of C/EBPβ‐LIP.

## Graphical abstract

N6‐methyladenosine is a key mRNA modification that regulates transcript turnover, translation, and other aspects of RNA fate. The m6A‐demethylase ‘eraser’ enzyme FTO reverses this modification. While FTO is known to promote oncogenic signaling in triple‐negative breast cancer, its wider role across the genome and specific downstream signaling has yet to be explored. Here, we investigated the changes in the genome‐wide transcriptome and translatome associated with FTO in triple‐negative breast cancer and identified the CEBPB mRNA as a critical downstream target.

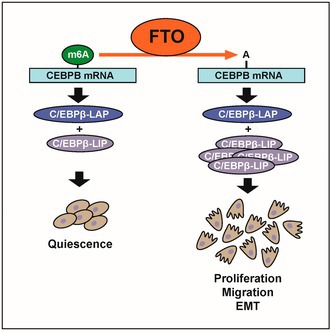

**Abbreviations**
(Me)RIP: (methylated‐)RNA immunoprecipitation
ALKBH5: alkB homolog 5
C/EBP (CEBPB): CCAAT enhancer binding protein (the gene)
ECM: extracellular matrix
EMT: epithelial‐mesenchymal transition
FTO: fat mass and obesity‐associated
LAP: Liver‐enriched activating protein (translational isoform of C/EBPβ)
LIP: Liver‐enriched inhibitory protein (translational isoform of C/EBPβ)
m6A: N6‐methyladenosine
METTL: methyltransferase‐like
RT‐qPCR: real time reverse transcription polymerase chain reaction.
TNBC: triple‐negative breast cancer
WTAP: Wilms' tumor 1‐associated protein

## Introduction

*N* ^6^‐Methyladenosine (m6A) is the most prevalent modification of mammalian mRNA and is involved in multiple steps of gene regulation [1, 2]. m6A modification is performed by a methyltransferase complex consisting of methyltransferase‐like 3 (METTL3), METTL14 and multiple accessory proteins including Wilms' tumor 1‐associating protein (WTAP), and is most often found in the 3′ untranslated region (3′UTR) and tends to occur at RRACH (R = G or A; H = A, C or U) consensus motifs [3, 4, 5, 6, 7]. m6A modification may destabilize mRNA through binding of YTH domain containing family proteins 1–3 (YTHDF1‐3) [8, 9], or if found in the coding region may regulate mRNA translation [10, 11, 12]. However, the transcriptome‐wide effects of m6A on translation and the involvement of YTHDF1 are disputed [13, 14].

Two mammalian demethylases are known: fat mass and obesity‐associated (FTO) and alkB homolog 5 (ALKBH5) [15, 16]. Both are ubiquitously expressed but their targets and biological functions appear to differ. *Alkbh5*‐knockout mice are anatomically normal but less fertile due to erroneous splicing and rapid degradation of genes involved in spermatogenesis [16, 17]. *Fto*‐knockout mice on the other hand are smaller with less lean mass and adipose tissue while no reduction in fertility has been reported [18]. In humans, single nucleotide polymorphisms (SNPs) in the *FTO* gene are the strongest genetic link with obesity in genome‐wide association studies (GWAS) [19]. More recently, FTO was shown to play a role in many types of cancer, both with oncogenic and tumor suppressive functions depending on tumor type and/or stage [20]. Mostly, FTO was found to act as an oncogene, for example in acute myeloid leukemia (AML) subtypes [21, 22], breast cancer [23] and glioma [24].

Triple‐negative breast cancer (TNBC) encompasses 10–15% of all breast tumors and is characterized by loss of estrogen and progesterone receptor expression and a lack of HER2 amplification, which are therefore not available as therapeutic targets [25]. TNBC has a poor prognosis with an overall 5‐year relapse of approximately 40% due to metastatic spread [26]. To be able to migrate to distant sites tumor cells have to undergo epithelial to mesenchymal transition (EMT) [27]. Cell migration processes are regulated by genes involved in extracellular matrix modulation, cell adhesion regulation, and actin cytoskeleton mobilization [28].

In tumor samples of breast cancer patients, high expression of the C/EBPβ transcription factor protein isoform C/EBPβ‐LIP correlates with loss of estrogen/progesterone receptor expression, a high Ki‐67 proliferative index, and lack of differentiation [29, 30, 31]. Previously, we showed that C/EBPβ‐LIP induces cancer‐type metabolic reprogramming through the induction of aerobic glycolysis to sustain cell growth [32] and promotes EMT gene expression as well as migration and invasion of breast cancer cells and untransformed epithelial cells [33]. Three C/EBPβ protein isoforms are expressed from the CEBPB mRNA through differential translation. The protein isoforms designated C/EBPβ‐LAP1 and C/EBPβ‐LAP2 (liver‐enriched activating proteins) are expressed through regular translation. These are transcriptional activators containing N‐terminal transcriptional transactivation domains and a C‐terminal basic DNA‐binding and leucine‐zipper dimerization domain (bZIP) [31, 34]. The expression of C/EBPβ‐LIP (liver‐enriched inhibitory protein) is strictly dependent on the initial translation of a *cis*‐regulatory small upstream open reading frame (uORF) and successive translation reinitiation from a downstream translation initiation site (for a detailed explanation, see [31]). The C/EBPβ‐LIP isoform lacks the transactivation domains but does bind to DNA through the bZIP domain [35]. C/EBPβ‐LIP translation is stimulated by the nutrient and energy sensing mTORC1‐4E‐BP‐eIF4E pathway, while its expression is restricted when mTORC1 signaling is suppressed and/or when under stress conditions activation of eIF‐2α‐kinases prevents reloading of post‐uORF ribosomes with initiator Met‐tRNA_i_^Met^ [31].

The molecular mechanisms and downstream targets of FTO's function in breast cancer growth and migration remain largely unexplored. We hypothesized that FTO may contribute to these processes through regulation of general mRNA translation and/or specific mRNA translation, the latter exemplified by translation of CEBPB mRNA. To reveal the genome‐wide effects of FTO expression in TNBC, we performed transcriptome and ribosome profiling experiments. While FTO knockdown hardly affected the translatome, transcriptome analysis revealed the regulation of several genes involved in extracellular matrix (ECM) and epithelial‐mesenchymal transition (EMT). Similar results were obtained by treatment with the catechol‐*O*‐methyltransferase and the FTO inhibitor entacapone. In addition, our data show that FTO knockdown affects C/EBPβ‐LIP expression, decreasing the C/EBPβ‐LIP/LAP ratio, while knockdown of the m6A‐methyltransferase complex component WTAP increases C/EBPβ‐LIP expression. Furthermore, we show that in TNBC cells ectopic expression of LIP rescues the migration defect caused by FTO knockdown, suggesting that LIP is involved in FTO‐mediated cell migration.

## Results

### FTO facilitates breast cancer cell proliferation and migration

**Fig. 1:**
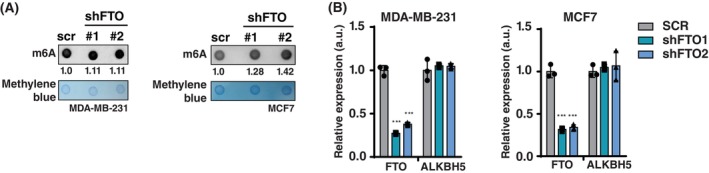
FTO knockdown increases m6A loading of mRNAs. (A) m6A dot blot of 100 ng mRNA from MDA‐MB‐231 cells or 150 ng mRNA from MCF‐7 cells with FTO knockdown (shFTO1 or shFTO2) versus scrambled‐shRNA (scr) control. The blots shown are representative of 3 biological replicates used for quantification of m6A signal relative to scr control shown beneath the blot. Methylene blue staining is shown as loading control. (B) RT‐qPCR analysis of *FTO* and *ALKBH5* gene expression in MDA‐MB‐231 or MCF‐7 cells with FTO knockdown (shFTO1 or shFTO2) versus scrambled‐shRNA control (scr). Values are normalized to scr control per gene. Significance was determined by one‐way ANOVA with Dunnett's *post hoc* test, ***P < 0.001 (mean ± SD, *n* = 3 biological replicates).

**Fig. 2:**
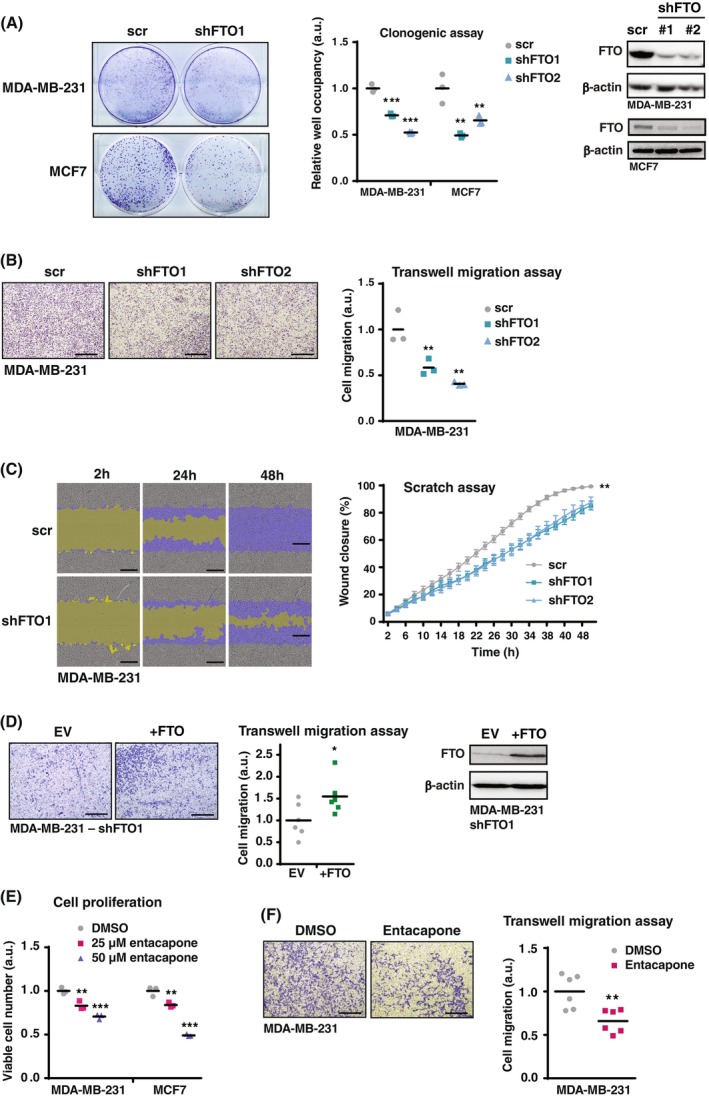
FTO knockdown impairs breast cancer cell proliferation and migration. (A) Clonogenic assay of MDA‐MB‐231 and MCF‐7 cells with FTO knockdown (shFTO1) or scrambled control (scr). Cells were harvested and stained after 7 days in cell culture. Quantification for two independent knockdown constructs (shFTO1 and shFTO2) is shown in the middle. At the right, immunoblots showing FTO expression levels in FTO knockdown cells and scr control cells, and β‐Actin was used as loading control. Values are shown as mean (−) and individual data points (*n* = 3); significance was determined by one‐way ANOVA with Dunnett's *post hoc* test, **P < 0.01, ***P < 0.001 (*n* = 3 biological replicates). (B) Transwell migration assay of MDA‐MB‐231 cells with FTO knockdown (shFTO1 or shFTO2) or scrambled control (scr) at 48 h. Scale bar represents 500 μm. Quantification is shown at the right. Values are shown as mean (−) and individual data points (*n* = 3); significance determined by one‐way ANOVA with Dunnett's *post hoc* test, **P < 0.01 (*n* = 3 biological replicates). (C) Time course of live‐cell imaging scratch wound assays using IncuCyte (Sartorius) of MDA‐MB‐231 cells with FTO knockdown (shFTO1 or shFTO2) or scrambled control (scr). Scale bar represents 300 μm. Quantification is shown at the right. Significance was determined by one‐way ANOVA with Dunnett's *post hoc* test, **P < 0.01 (mean ± SEM, *n* = 4 biological replicates). (D) Transwell migration assay of MDA‐MB‐231 cells with FTO knockdown (shFTO1) with re‐expression of FTO or empty vector (EV) control at 48 h. Scale bar represents 500 μm. Quantification of cell migration is shown as mean (−) and individual data points (*n* = 6, technical replicates); significance was determined by Student's *t*‐test, *P < 0.05. At the right immunoblots showing FTO re‐expression or empty vector (EV) control in FTO knockdown MDA‐MB‐231 cells, and β‐Actin as loading control. (E) Cell viability assay of MDA‐MB‐231 and MCF‐7 cells treated with 25 or 50 μm entacapone or DMSO control for 3 days. Values are shown as mean (−) and individual data points (*n* = 3); significance was determined by one‐way ANOVA with Dunnett's *post hoc* test, **P < 0.01, ***P < 0.001 (*n* = 3 biological replicates). (F) Transwell migration assay at 48 h of MDA‐MB‐231 cells treated with 25 μm entacapone or DMSO. Scale bar represents 500 μm. Quantification is shown at the right as mean (−) and individual data points (*n* = 6); significance determined by Student's *t*‐test. **P < 0.01 (*n* = 3 biological replicates).

**Fig. 3:**
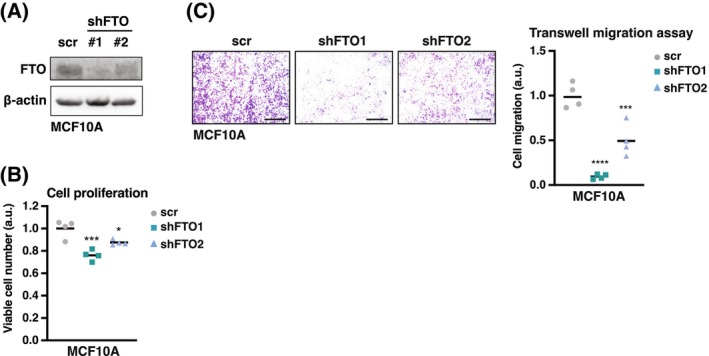
FTO knockdown impairs cell proliferation and migration in untransformed MCF10A cells. (A) A representative immunoblot showing FTO expression levels in MCF10A cells with FTO knockdown (shFTO1 or shFTO2) or in scrambled‐shRNA control cells (scr) (*n* = 3 technical replicates). The immunoblot showing β‐Actin expression was used as loading control. (B) Cell viability assay of MCF10A cells with FTO knockdown (shFTO1 or shFTO2) or scrambled control (scr) after cultivation for 3 days. Significance was determined by one‐way ANOVA with Dunnett's *post hoc* test, *P < 0.05, ***P < 0.001 (*n* = 4) biological replicates. (C) Transwell migration assay of MCF10A cells with FTO knockdown (shFTO1 or shFTO2) or scrambled control (scr) at 48 h. Scale bar represents 500 μm. Quantification is shown at the right. Significance determined by one‐way ANOVA with Dunnett's *post hoc* test, ***P < 0.001, ****P < 0.0001 (*n* = 4 biological replicates).

We examined the effect of FTO knockdown on the proliferation and migration of the breast cancer cell lines MDA‐MB‐231 (triple‐negative breast cancer) and MCF‐7 (luminal A type metastatic adenocarcinoma) using short hairpin lentiviral vectors (shFTO). FTO knockdown resulted in the expected general increase in the m6A loading of mRNAs, as measured by m6A‐mRNA dot blotting (Fig. 1A), while it did not affect the expression of the other known m6A demethylase ALKBH5 (Fig. 1B). In a clonogenic assay, cell proliferation was significantly reduced upon FTO knockout in MDA‐MB‐231 and MCF‐7 cells (Fig. 2A). The effect of FTO knockdown on the migration of MDA‐MB‐231 cells was measured using a Boyden chamber (Transwell migration) assay (Fig. 2B) and by live‐cell imaging scratch wound assays using the IncuCyte system (Sartorius) (Fig. 2C). Both assays showed reduced cell migration upon FTO knockdown. Moreover, stable re‐expression of FTO in FTO‐knockdown MDA‐MB‐231 cells resulted in rescue of the Transwell migration (Fig. 2D). In addition, treatment with the FTO inhibitor entacapone caused a dose‐dependent decrease in cell proliferation in both MDA‐MB‐231 and MCF‐7 cells (Fig. 2E) and caused a reduction in MDA‐MB‐231 Transwell migration (Boyden chamber) (Fig. 2F). Finally, FTO knockdown in the untransformed MCF10A breast epithelial cell line inhibited cell proliferation and Transwell migration in the presence of growth factors (Fig. 3A–C). Together, these data and data from another laboratory [23] show that the m6A‐demethylase FTO promotes proliferation and migration in breast cancer cells.

### FTO regulates ECM and EMT gene expression

**Fig. 4:**
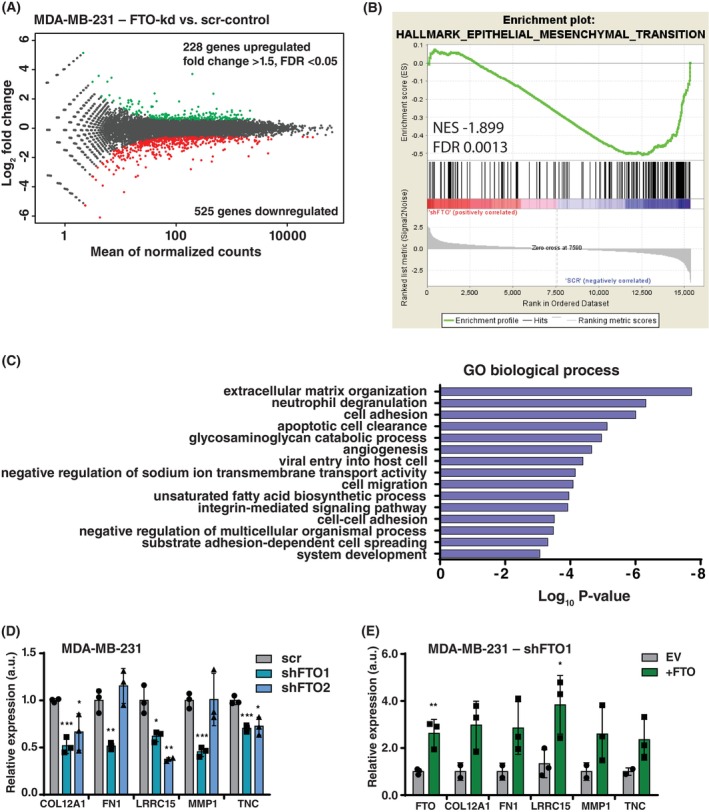
Transcriptome analysis revealing FTO‐regulated extracellular matrix (ECM) and epithelial‐mesenchymal transition (EMT) genes. (A) Transcriptome analysis of MDA‐MB‐231 cells with FTO knockdown versus scrambled‐shRNA (scr) control (*n* = 2 biological replicates). Significantly regulated genes are displayed in either red (downregulated) or green (upregulated)(fold change >1.5; FDR <0.05). (B) Gene set enrichment analysis (GSEA) for the hallmark epithelial‐mesenchymal transition shows significant negative enrichment in MDA‐MB‐231 FTO knockdown cells versus scrambled‐shRNA (scr) control. (C) Gene ontology (GO) term analysis of biological processes downregulated upon FTO knockdown in MDA‐MB‐231 cells versus scrambled‐shRNA control. Significantly enriched GO‐terms (*P* < 0.001) are shown with their respective *P*‐values (Fisher elim. topGO). (D) RT‐qPCR analysis of ECM/EMT‐related genes found to be downregulated upon FTO knockdown (shFTO1 or shFTO2) versus shRNA‐scrambled (scr) control in MDA‐MB‐231 cells. Significance was determined by one‐way ANOVA with Dunnett's *post hoc* test, *P < 0.05, **P < 0.01, ***P < 0.001 (mean ± SD, *n* = 3 biological replicates). (E) RT‐qPCR analysis of ECM/EMT‐related genes in MDA‐MB‐231 FTO knockdown (shFTO1) cells after re‐expression of FTO compared to empty vector (EV) control. Significance was determined by one‐way ANOVA with Dunnett's *post hoc* test, *P < 0.05, **P < 0.01 (mean ± SD, *n* = 3 biological replicates).

Since FTO was shown to regulate mRNA‐stability we examined FTO knockdown‐induced alterations in the genome‐wide transcriptome. Transcriptome analysis of MDA‐MB‐231 cells identified 228 up‐ and 525 downregulated genes upon FTO knockdown (FDR <0.05, fold change >1.5) (Fig. 4A; Table S1). The finding that more genes were downregulated than upregulated agrees with the described mRNA destabilizing effect of m6A modification [9]. Gene Set Enrichment Analysis (GSEA) (https://www.gsea‐msigdb.org/gsea, Broad Institute) [36] revealed a significant negative enrichment of the epithelial to mesenchymal transition (EMT) signature in FTO knockdown cells compared to control cells (Fig. 4B). Furthermore, most of the top 15 significantly enriched gene ontology (GO) terms from the downregulated genes are related to the extracellular matrix (ECM) or EMT (Fig. 4C). qPCR analysis confirmed the downregulation of a subset of genes by FTO knockdown, including Collagen Type XII Alpha 1 Chain (*COL12A1*), Fibronectin 1 (*FN1*), Matrix Metalloproteinase 1 (*MMP1*), Leucine Rich Repeat Containing 15 (*LRRC15*) and Tenascin C (*TNC*) (Fig. 4D). Moreover, in MDA‐MB‐231 cells with FTO knockdown, the reintroduction of FTO led to a notable increase in the expression of the aforementioned genes. However, statistical significance was only reached for LRRC15 among these genes (Fig. 4E). Overall, the results concur with the rescue of the Transwell migration phenotype upon FTO re‐expression in FTO‐knockdown MDA‐MB‐231 cells (Fig. 4D).

**Fig. 5:**
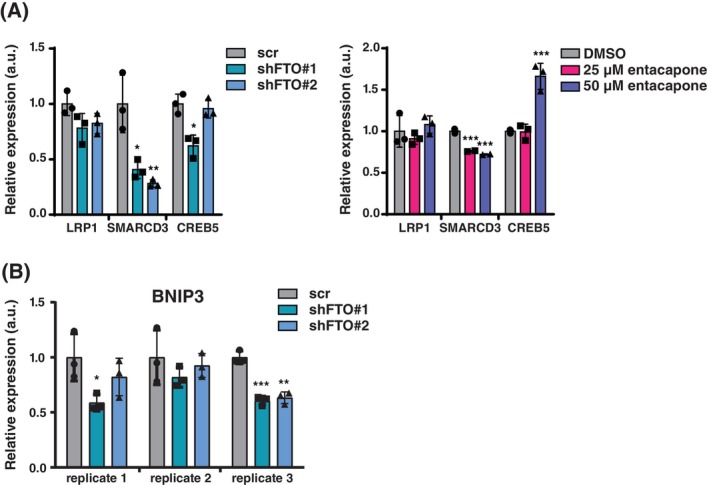
Analysis of specific genes regulation by FTO knockdown or inhibition of FTO. (A) Left graph, RT‐qPCR analysis of *LRP1*, *SMARCD3* and *CREB5* expression in MDA‐MB‐231 cells upon FTO knockdown (shFTO1 or shFTO2) versus shRNA‐scrambled (scr) control. Significance was determined by one‐way ANOVA with Dunnett's *post hoc* test, *P < 0.05, **P < 0.01 (mean ± SD, *n* = 3 biological replicates). Right graph, RT‐qPCR analysis of *LRP1*, *SMARCD3* and *CREB5* expression in MDA‐MB‐231 cells treated with 25 or 50 μm entacapone or DMSO for 3 days. Significance was determined by one‐way ANOVA with Dunnett's *post hoc* test, ***P < 0.001 (mean ± SD, *n* = 3 biological replicates). (B) RT‐qPCR analysis of *BNIP3* expression in MDA‐MB‐231 cells with FTO knockdown (shFTO1 or shFTO2) versus shRNA‐scrambled (scr) control. Significance was determined by one‐way ANOVA with Dunnett's *post hoc* test, *P < 0.05, **P < 0.01, ***P < 0.001 (mean ± SD, *n* = 3 biological replicates).

Others have screened cell signaling components for their effect on TNBC cell migration [37]. Out of 4198 individual genes, 153 were identified and validated to regulate cell migration in MDA‐MB‐231 cells [37]. We overlaid our list of downregulated genes with the validated hits from [37] and identified Low Density Lipoprotein Receptor‐Related Protein 1 (*LRP1*), cAMP Responsive Element Binding Protein 5 (*CREB5*) and SWI/SNF related, matrix Associated, Actin Dependent Regulator of Chromatin, Subfamily D, Member 3 (*SMARCD3*) as the only three shared regulated genes. Upon FTO knockdown or treatment with entacapone we observed a significant and consistent downregulation only for *SMARCD3* in MDA‐MB‐231 cells (Fig. 5A). In another study, pro‐apoptotic BCL2 interacting protein 3 (BNIP3) was identified as the main gene that is downregulated by FTO through m6A demethylation. However, we failed to observe upregulation of BNIP3 in response to FTO knockdown in MDA‐MB‐231 cells and rather observed a trend toward downregulation of BNIP3 (Fig. 5B).

### FTO does not directly regulate ECM/EMT mRNA stability

**Fig. 6:**
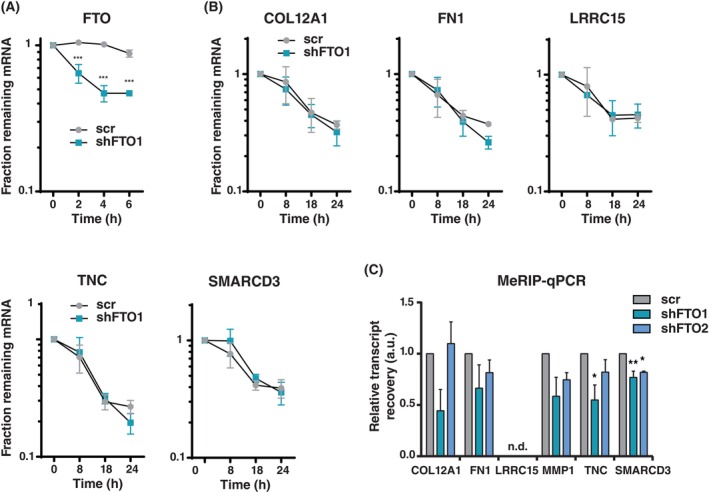
FTO does not regulate ECM/EMT target gene transcript stability or level of m6A modification. (A) Decay curves of FTO mRNA in MDA‐MB‐231 cells with FTO knockdown (shFTO1) versus scrambled‐shRNA (scr) control. Significance was determined by multiple Student's *t*‐test with Holm‐Sidak correction, ***P < 0.001 (mean ± SEM, *n* = 3 biological replicates). (B) Decay curves of mRNA from indicated genes in MDA‐MB‐231 cells with FTO knockdown (shFTO1) versus scrambled (scr) control. Significance was determined by multiple Student's *t*‐test with Holm‐Sidak correction (mean ± SEM, *n* = 3 biological replicates). (C) Quantification of mRNA‐m6A levels of indicated genes in MDA‐MB‐231 cells with FTO knockdown (shFTO1 or shFTO2) versus scrambled (scr) control by MeRIP‐qPCR. Significance was determined by one‐way ANOVA with Dunnett's *post hoc* test.*P < 0.05, **P < 0.01. n.d.: not detected (mean ± SEM, *n* = 3 biological replicates).

m6A‐modification of mRNAs is linked to the regulation of mRNA stability by promoting degradation [9, 14]. To examine whether the downregulation of ECM and EMT mRNAs in response to FTO knockdown is caused by mRNA degradation, we treated the cells with the transcription inhibitor actinomycin D and followed the degradation of selected transcripts over time. As expected, FTO mRNA levels declined in FTO‐knockdown cells (Fig. 6A). However, none of the regulated transcripts showed changes in mRNA stability upon FTO knockdown (Fig. 6B). In addition, we did not observe an increase in mRNA‐m6A levels for any of the regulated genes analyzed by methylated‐RNA immunoprecipitation using a m6A antibody followed by real‐time reverse transcription polymerase chain reaction (MeRIP‐RT‐qPCR) (Fig. 6C). These data indicate that FTO does not regulate these transcripts directly.

### FTO deficiency does not significantly affect translation in TNBC cells

**Fig. 7:**
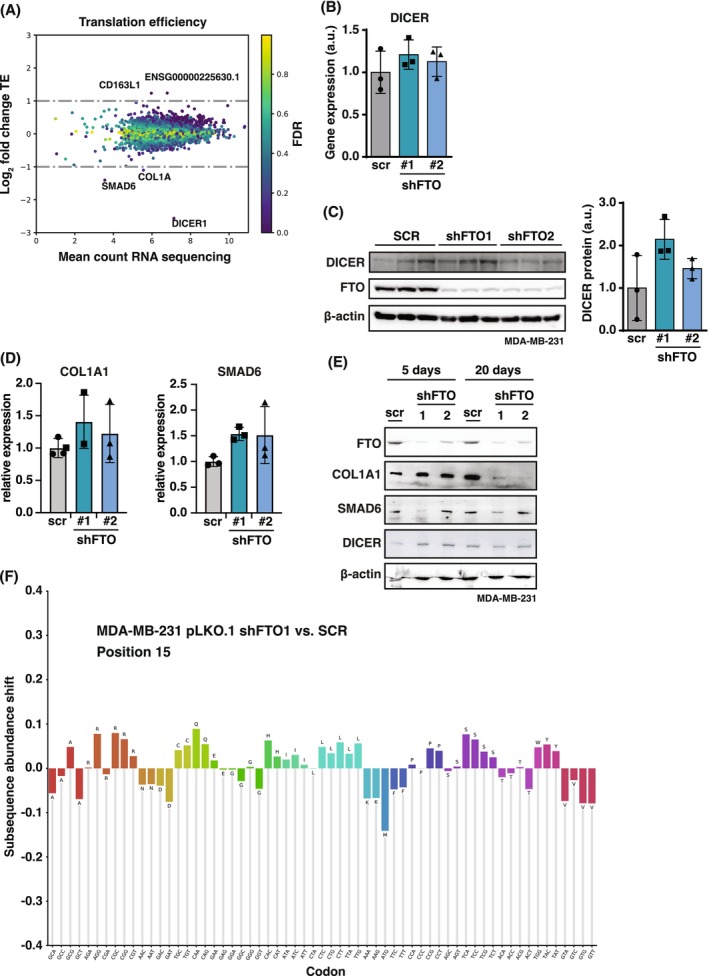
FTO knockdown has a limited effect on mRNA translation. (A) Fold change in translation efficiency upon FTO knockdown in MDA‐MB‐231 cells is displayed, color coded by FDR. Significantly regulated genes above threshold are indicated by gene symbol. (B) RT‐qPCR analysis of *DICER* mRNA expression in MDA‐MB‐231 cells with FTO knockdown (shFTO1 or shFTO2) versus shRNA‐scrambled (scr) control. Significance was determined by one‐way ANOVA with Dunnett's *post hoc* test (mean ± SEM, *n* = 3 biological replicates). (C) Immunoblots showing no change in DICER protein expression upon knockdown of FTO (shFTO1 or shFTO2) in MDA‐MB‐231 versus scrambled control (SCR). β‐Actin was used as loading control. Quantification is shown on the right. Significance was determined by one‐way ANOVA with Dunnett's *post hoc* test (mean ± SEM, *n* = 3 biological replicates). (D) RT‐qPCR analysis of *COL1A1* and *SMAD6* mRNA expression in MDA‐MB‐231 upon FTO knockdown (shFTO1 or shFTO2) versus shRNA‐scrambled (scr) control. Significance was determined by one‐way ANOVA with Dunnett's *post hoc* test (mean ± SD, *n* = 3 biological replicates). (E) Immunoblot analysis of COL1A1, SMAD6, and DICER protein expression in MDA‐MB‐231 upon FTO knockdown (shFTO1 or shFTO2) versus shRNA‐scrambled (scr) control, and β‐Actin as loading control. 5 days and 20 days refer to days in cell culture after transduction with shRNA retroviral vector. (F) Subsequence abundance analysis of codons at the +15 position relative to the ribosome protected fragment (RPF) 5′‐end. All transcripts in the coding frame are considered for this analysis. No significant enrichment or depletion of codons can be seen, indicating no obvious shortages of amino acids causing ribosome stalls/halts.

In addition to mRNA turnover, FTO function has been linked to the regulation of mRNA‐translation [10, 12]. To investigate FTO‐dependent differences in the translatome, we performed ribosome profiling experiments with FTO knockdown and scrambled‐shRNA MDA‐MB‐231 TNBC cells. To our surprise, the translatome was only mildly affected by FTO deficiency and only five transcripts showed more than twofold and significantly altered translation efficiencies (FDR <0.05, fold change >2, Fig. 7A; Table S2). The most strongly reduced in ribosome loading was DICER1, yet DICER mRNA levels determined by RT‐qPCR or DICER protein levels analyzed by immunoblotting did not reveal a significant change upon FTO knockdown (Fig. 7B,C,E). The SMAD6 and COL1A1 transcripts showed mildly reduced ribosome loading. RT‐qPCR analysis showed no significant difference in expression levels (Fig. 7D). Protein levels of COL1A1 were found reduced after long‐term (20 days posttransduction) culture of FTO‐knockdown cells and protein levels of SMAD6 were found lower only for the most effective shRNA1‐FTO knockdown (Fig. 7E). These analyses suggest that FTO‐mediated demethylation of the COL1A1 and SMAD6 mRNAs enhances their translation, of which FTO‐COL1A1 regulation has also been observed by others [38].

The ribosome profiling experiment provides additional data about possible FTO‐related amino acid or tRNA metabolism. The position of a ribosome on the mRNA‐ribosome protected fragment (RPF) can be deduced from the RPF 5′ end. Using this information and the known mRNA reading frame, the codons in the ribosomal A (aminoacyl), P (peptidyl‐transfer) and E (exit) sites can be determined. Positions 9, 12 and 15 from the 5′ end of the RFPs correspond to respectively the ribosomal E, P and A site, respectively. When for example a particular amino acid or tRNA is depleted from cells, this will cause an accumulation of ribosomes on the corresponding codons. Since amino acid recognition takes place at the A‐site, the affected codons will be enriched at position 15 in RPFs [39]. Using differential ribosome codon reading (diricore) analysis [39] no significant differences in ribosome A‐site occupancy were observed (Fig. 7F). As no specific amino acid or tRNA shortages are induced by FTO deficiency, this indicates that FTO is not critically involved in their metabolic regulation.

### FTO and WTAP regulate C/EBPβ isoform expression

**Fig. 8:**
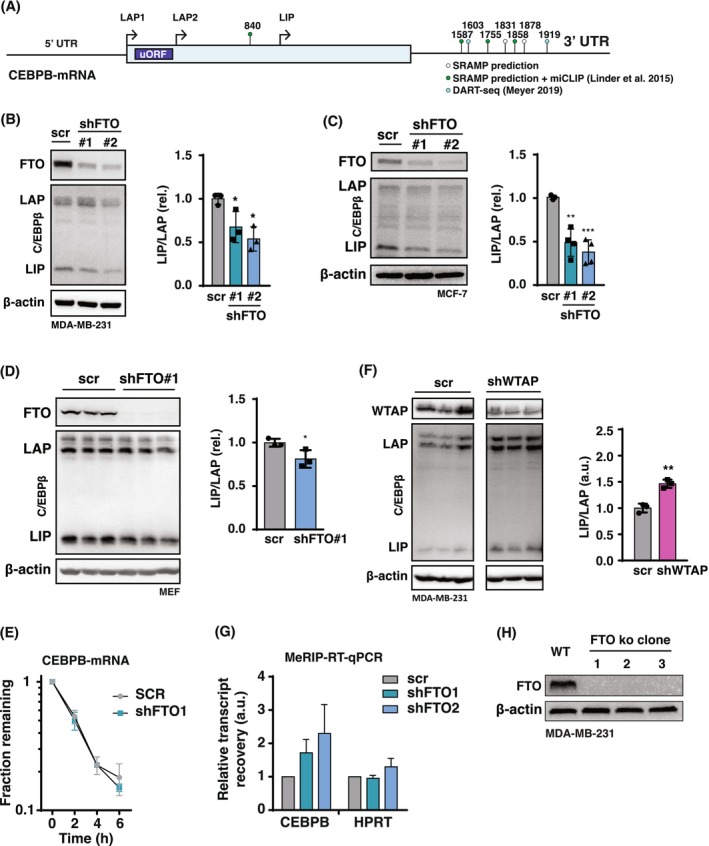
FTO or WTAP knockdown reciprocally regulates C/EBPβ isoform expression. (A) Schematic overview of the human CEBPB mRNA with indicated translation start sites for LAP1, LAP2, LIP and the uORF. Predicted (SRAMP [41]) and experimentally detected m6A modifications from HEK293T cells (miCLIP: Linder *et al*. 2015 [42], DARTseq Meyer 2019 [43]) are indicated with adenosine base number. (B) Immunoblot showing reduced levels of C/EBPβ‐LIP upon knockdown of FTO (shFTO1 or shFTO2) in MDA‐MB‐231 cells versus scrambled control (scr). β‐Actin was used as loading control. Quantification of C/EBPβ‐LIP/LAP ratio with significance determined by Student's *t*‐test. *P < 0.05 (*n* = 3 biological replicates). (C) Immunoblot showing reduced levels of C/EBPβ‐LIP upon knockdown of FTO (shFTO1 or shFTO2) in MCF‐7 cells versus scrambled control (scr). β‐Actin was used as loading control. Quantification of C/EBPβ‐LIP/LAP ratio with significance determined by Student's *t*‐test. **P < 0.01, ***P < 0.001 (*n* = 4 biological replicates). (D) Immunoblot showing reduced levels of C/EBPβ‐LIP upon knockdown of FTO (shFTO1) in MEF cells versus scrambled control (SCR). β‐Actin was used as loading control. Quantification of C/EBPβ‐LIP/LAP ratio with significance determined by Student's *t*‐test. *P < 0.05 (*n* = 3 biological replicates). (E) Decay curves of CEBPB mRNA from MDA‐MB‐231 cells with FTO knockdown (shFTO1) versus shRNA‐scrambled (scr) control. Values represent mean ± SEM of 3 biological replicates. Significance was determined by multiple Student's *t*‐test with Holm‐Sidak correction. (F) Immunoblot showing increased levels of C/EBPβ‐LIP upon knockdown of WTAP (shWTAP) in MDA‐MB‐231 cells versus scrambled control (scr). β‐Actin was used as loading control. Quantification of C/EBPβ‐LIP/LAP ratio with significance determined by Student's *t*‐test. **P < 0.01 (*n* = 3 biological replicates). (G) Quantification of mRNA‐m6A levels for *CEBPB* and *HPRT* (control gene not regulated by m6A) in MDA‐MB‐231 cells upon knockdown of FTO (shFTO1 or shFTO2) versus scrambled control (scr) by MeRIP‐RT‐qPCR. Bars represent mean ± SEM (*n* = 3 biological replicates). (H) Immunoblot showing loss of FTO expression in three independent FTO knockout clones obtained by CRISPR‐Cas9 targeting compared to parental MDA‐MB‐231 cells. β‐Actin was used as loading control.

Although transcript levels of genes associated with ECM/EMT were reduced upon FTO knockdown in MDA‐MB‐231 cells, this effect seems to be not directly mediated by FTO since FTO knockdown neither reduced the ribosome occupancy nor the stability of the transcripts. We therefore reasoned that their regulation likely occurs at the transcriptional level through a factor that is controlled by FTO. Additionally, the unexpectedly low number of genes exhibiting more than a twofold translational regulation prompted the question of whether FTO might influence specific translation events on individual mRNAs without significantly altering overall ribosome loading. The regulation and functions of the transcription factor of C/EBPβ fits in this scenario, as it undergoes distinct translation into three protein isoforms and these isoforms play a pivotal role in regulating ECM/EMT genes [40]. In addition to C/EBPβ‐LAP1 and ‐LAP2 protein isoforms that are generated by regular translation, translation into an additional N‐terminally truncated C/EBPβ‐LIP isoform involves a *cis*‐regulatory upstream open reading frame (uORF) in the transcript leader sequence (Fig. 8A) [31]. We have shown previously that its translational upregulation supports breast cancer cell proliferation and migration and regulates the expression of ECM/EMT transcripts [40]. Sequence‐based RNA adenosine methylation site predictor (SRAMP, http://www.cuilab.cn/sramp) identified six putative m6A sites with the highest confidence level in the CEBPB mRNA [41]. Published data on m6A individual‐nucleotide‐resolution crosslinking and immunoprecipitation (miCLIP) experiments confirmed m6A modification of four of these sites [42], while antibody‐free deamination adjacent to RNA modification targets (DART)‐seq identified two additional sites [43]. In concordance with the genome‐wide distribution of m6A on transcripts [6], all but one of these sites are in the 3′UTR of the CEBPB mRNA (Fig. 8A). In a study using m^6^A‐level and isoform‐characterization sequencing (m^6^A‐LAIC‐seq), CEBPB mRNA was found to be m6A‐modified in a human embryonic stem cell or B‐cell lymphoblastoid cell line [44]. To study whether m6A modification of the CEBPB mRNA contributes to its translational regulation, we applied FTO knockdown (shFTO) in triple‐negative MDA‐MB‐231 and luminal A‐type MCF‐7 breast cancer cell lines, and mouse embryonic fibroblasts (MEFs). Knockdown of FTO decreased C/EBPβ‐LIP levels with a concomitant decrease in the C/EBPβ‐LIP/LAP ratio in MDA‐MB‐231 cells (Fig. 8B) and MCF‐7 cells (Fig. 8C), and to a lesser extent in MEFs (Fig. 8D). At least in MDA‐MB‐231 cells, FTO knockdown did not affect CEBPB mRNA stability (Fig. 8E). Subsequently, we investigated the effects of abolishing methyltransferase activity, which catalyzes m6A modification, by targeting the methyltransferase complex component, WTAP. Knockdown of WTAP in MDA‐MB‐231 cells resulted in elevated expression levels of C/EBPβ‐LIP (Fig. 8F). To directly examine FTO‐related changes in CEBPB mRNA‐m6A levels, we performed methylated RNA immunoprecipitation using a m6A antibody followed by real‐time reverse transcription polymerase chain reaction (MeRIP‐RT‐qPCR). Recovery after IP over input was determined as a reflection of transcript m6A levels. A clear trend toward increased CEBPB mRNA m6A‐levels upon FTO knockdown was observed that did not reach statistical significance because of high experimental variation (Fig. 8G).

**Fig. 9:**
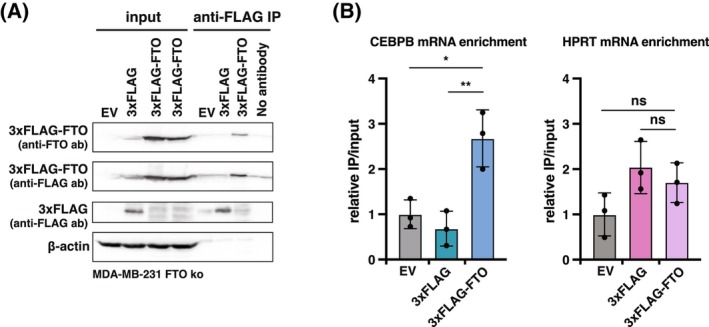
FTO binds the CEBPB mRNA. (A) Immunoblot with a FLAG‐specific antibody showing immunoprecipitated 3xFLAG‐FTO and 3xFLAG control peptide after FLAG‐specific immunoprecipitation as well as in input control samples as indicated (3xFLAG control peptide is fusion to a random sequence derived from the expression vector). Immunoprecipitation of 3xFLAG‐FTO was confirmed with an FTO‐specific antibody. A β‐Actin antibody served as loading control for the input samples. The experiment was performed once (*n* = 1). (B) *CEBPB* mRNA (left) or *HPRT* mRNA (right, negative control) enrichment in 3xFLAG‐FTO cells and in 3xFLAG control cells presented as IP/input and normalized to empty vector control cells. Significance determined by one‐way ANOVA with Dunnett's *post hoc* test, ns, not significant, *P < 0.05, **P < 0.01, (*n* = 3 technical replicates).

To investigate whether FTO interacts with CEBPB mRNA, we conducted an FTO‐specific RNA immunoprecipitation (RIP). We generated MDA‐MB‐231 cells which an FTO knockout using CRISPR/Cas9 gene editing (clone 2, Fig. 8H). In these cells, we ectopically expressed human FTO fused to a 3xFLAG tag at its N‐terminus (3xFLAG‐FTO) or the 3xFLAG tag alone as a control. Additionally, HPRT mRNA, which is known not to be acetylated, was measured as a negative control. Following FLAG‐specific immunoprecipitation, we assessed the binding of CEBPB or HPRT mRNA to 3xFLAG‐FTO compared to the 3xFTO control (Fig. 9A,B). As shown in Fig. 9B, CEBPB mRNA was significantly enriched in 3xFLAG‐FTO immunoprecipitation relative to the 3xFLAG control, demonstrating that FTO binds to CEBPB mRNA. In contrast, no differential binding was observed for HPRT.

Taken together, the data suggest that reversible WTAP‐ and FTO‐controlled m6A modification of CEBPB mRNA alters its translation.

### C/EBPβ‐LIP overexpression partially rescues the migration phenotype of cells with FTO knockdown

**Fig. 10:**
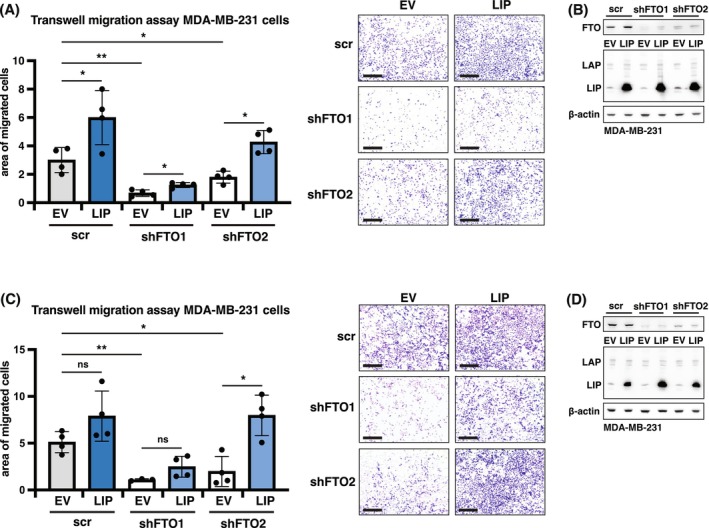
Ectopic expression of C/EBPβ‐LIP enhances cell migration independent of FTO (*n* = 2 biological replicates). (A) First Transwell migration assay of MDA‐MB‐231 cells with FTO knockdown (shFTO1 or shFTO2) or scrambled control (scr) with overexpression C/EBPβ‐LIP or empty vector (EV) control, at 48 h. The graph shows quantification of migrated cells with pictures of stained cells presented on the right. Scale bar represents 500 μm. Significance determined by one‐way ANOVA with Dunnett's *post hoc* test, *P < 0.05, **P < 0.01 (*n* = 4 technical replicates). (B) Immunoblot showing overexpression of C/EBPβ‐LIP in FTO knockdown (shFTO1 or shFTO2) or scrambled‐shRNA (scr) control MDA‐MB‐231 cells. β‐Actin was used as loading control. (C) Second Transwell migration assay of MDA‐MB‐231 cells with FTO knockdown (shFTO1 or shFTO2) or scrambled control (scr) with overexpression C/EBPβ‐LIP or empty vector (EV) control, at 48 h. The graph shows quantification of migrated cells with pictures of stained cells presented on the right. Scale bar represents 500 μm. Significance determined by one‐way ANOVA with Dunnett's *post hoc* test, *P < 0.05, **P < 0.01 (*n* = 4 technical replicates). (D) Immunoblot showing overexpression of C/EBPβ‐LIP in FTO‐knockdown (shFTO1 or shFTO2) or scrambled‐shRNA (scr) control MDA‐MB‐231 cells. β‐Actin was used as loading control.

Since we showed earlier that LIP stimulates the migration of breast cancer as well as untransformed breast epithelial cells [40], we investigated whether overexpression of C/EBPβ‐LIP would reverse the reduced MDA‐MB‐231 cell migration phenotype caused by FTO knockdown. Overexpression of C/EBPβ‐LIP in wt MDA‐MB‐231 control cells significantly further increased cell migration (Boyden chamber) in one of the two independent experiments (Fig. 10A–D). FTO knockdown strongly reduced cell migration in all cases, as expected. C/EBPβ‐LIP overexpression efficiently rescued cell migration in shFTO2‐knockout cells and moderately rescued migration in one of the shFTO1‐knockout cells. Altogether, the data indicate that (m6A)‐demethylation of CEBPB mRNA facilitates C/EBPβ‐LIP upregulation, which could contribute to the promigratory phenotype induced by FTO.

## Discussion

Here we present data showing that FTO expression stimulates the proliferation and migration of MDA‐MB‐231 (triple‐negative breast cancer) and MCF‐7 (luminal A type metastatic adenocarcinoma) breast cancer cells in cell culture. Our ribosome profiling experiment and transcriptome analysis using MDA‐MB‐231 cells and FTO knockdown showed little effect on the translatome but revealed significant regulation of transcripts involved in the ECM and EMT. In addition, the data show that FTO knockdown reduces the expression of C/EBPβ‐LIP, while WTAP knockdown results in higher levels of C/EBPβ‐LIP, suggesting that binding of FTO to CEBPB mRNA and FTO‐mediated m6A demethylation is involved in the regulated translation of the CEBPB mRNA into its protein isoforms C/EBPβ‐LAP1/2 and C/EBPβ‐LIP.

Various roles of FTO‐mediated m6A demethylation in the development of cancer progression has emerged in recent years. While tumor suppressive functions for FTO have been described in for example colorectal cancer and hepatocellular carcinoma, reports describing oncogenic functions in different cancer types including lung cancer, glioma and AML are far more abundant (reviewed in [20]). Analysis of broad collections of TNBC cells and samples suggests that FTO function must be maintained for TNBC cell survival. The catalog of somatic mutations in cancer (COSMIC, https://cancer.sanger.ac.uk/cosmic) database reports only a single intronic mutation in the FTO gene in 117 TNBC samples analyzed. Furthermore, in the 83 breast cancer cell lines listed in the cancer cell line encyclopedia (CCLE) dependency map portal (depmap, https://depmap.org/portal/) only a single cell line with an intronic mutation was found.

Our results and data presented by Niu *et al* [23] show that FTO function is required for breast cancer cell proliferation and migration. The study by Niu *et al*. [23] showed that FTO is upregulated in breast tumor samples together with downregulation of METTL3/14, ALKBH5, YTHDC2, and YTHDF1, and is associated with an overall decline in global mRNA m6A levels. Furthermore, they showed that in breast cancer patients high FTO levels correlate with poorer survival. Niu *et al*. [23] identified the pro‐apoptotic BNIP3 as the downstream target of FTO by transcriptome and m6A‐seq analysis using MDA‐MB‐231 FTO‐knockdown and MCF‐7 cells treated with the FTO inhibitor GSE3188. They also showed that BNIP3 expression alleviates FTO‐dependent tumor growth in a xenograft mouse model. However, we did not observe such regulation of BNIP3 in MDA‐MB‐231 cells and rather observed downregulation upon FTO knockdown.

Instead, our study shows that FTO knockdown inhibits and WTAP knockdown stimulates translation into C/EBPβ‐LIP, which can act as an oncogene and regulates EMT/ECM genes in TNBC [40]. This suggests that for efficient uORF‐dependent regulation of LIP, FTO‐mediated CEBPB mRNA demethylation is required, which is supported by the results of our MeRIP‐RT‐qPCR and FTO‐specific RNA‐immunoprecipitation experiments shown in Fig. 5E–G, respectively. Published data and bioinformatic analysis identified several m6A sites in the CEBPB mRNA (Fig. 5A) [41, 42, 43] and future studies should aim to identify the m6A site(s) and involved translational mechanisms in FTO‐C/EBPβ regulation. In addition to m6A sites, FTO also demethylates m6Am site that are located adjacent to the mRNA 7‐methylguanosine cap [20, 45], which raises the question whether the regulation of CEBPB mRNA translation may involve an m6Am site. However, this is rather unlikely because previous m6Am‐seq experiments conducted by others did not detect an m6Am site in the CEBPB mRNA sequence [46].

We show that ectopic expression of C/EBPβ‐LIP stimulates cell migration in FTO knockdown cells, suggesting that at least part of the effects of FTO on cell migration occur through regulation of C/EBPβ‐LIP. The question that arises from this observation is whether FTO collectively regulates mRNAs containing uORFs or other translation *cis*‐regulatory elements. In line with this hypothesis, amino acid starvation was shown to regulate the translation activating transcription factor 4 (ATF4) via m6A [47]. ATF4 translation depends on reinitiation following uORF translation, allowing bypassing of an inhibitory second uORF when eIF2‐GTP levels are low [48]. FTO or ALKBH5 knockdown represses ATF4 reinitiation, whereas knocking down the m(6)A methyltransferase subunits METTL3 or METTL14 promotes ATF4 translation [47]. Translation initiation site profiling experiments using the inhibitor harringtonine, which stalls ribosomes at the initiation sites [49], should be performed to investigate whether FTO translational regulation is more about start‐site selection than general translation efficiency.

The translatome experiment only showed very limited changes upon FTO knockdown, with only two transcripts being translationally upregulated and three transcripts being translationally downregulated. Immunoblotting experiments showed that COL1A1 and SMAD6 protein levels were accordingly downregulated, yet that DICER protein levels were not changed, although DICER was the most strongly regulated mRNA. It is possible that FTO knockdown did not reach levels low enough to broadly affect translation, and FTO knockout would have been a better strategy to reveal such regulation. Nonetheless, in a (patho)physiological setting, one would rather expect levels of FTO to vary than to be lost completely. For example, in humans, a recessive loss‐of‐function mutation in *FTO* leads to postnatal growth retardation, microcephaly, facial dimorphism, functional brain deficits and death within 30 months of age [50].

FTO initially became known as the most significant genetic factor associated with human obesity. Its key role in fat metabolism was experimentally revealed by whole‐body knockout of *Fto* in mice, which resulted in decreased body weight in both males and females on normal chow and a significantly reduced weight gain on a high‐fat diet. Conversely, ubiquitous overexpression of FTO in mice led to increased body weight and fat mass in both males and females, observed across normal as well as high‐fat diet conditions (reviewed in [20]). Intriguingly, mutation of the uORF in the CEBPB gene in mice causing deficiency in C/EBPβ‐LIP expression results in a metabolic phenotypes resembling those found in FTO knockout mice [51]. Although not investigated, this suggests that part of the FTO‐related phenotype in mice is caused through C/EBPβ regulation.

Taking into account the many studies on the diverse and partially contradictory roles of FTO in cancer (reviewed in [20, 52]), one must conclude that the effects of FTO on specific and global regulation of RNA demethylation can only be understood and eventually employed for therapeutic strategies in specific situations, e.g., a specific cancer type at a specific stage.

## Materials and methods

### Cell culture

MDA‐MB‐231 (RRID:CVCL_0062; cell line authentication by Eurofins Genomics, Ebersberg, Germany), HEK293T (RRID:CVCL_0063) and MEF [51] cells were cultured in DMEM, high glucose with GlutaMAX and pyruvate (Gibco, Thermo Fisher Scientific, Sittard, The Netherlands) supplemented with 10% FCS, 10 mm HEPES and penicillin/streptomycin. MCF‐7 (RRID:CVCL_0031) cells were cultured in RPMI 1640 with GlutaMax and 25 mm HEPES (Gibco, Thermo Fisher Scientific, Sittard, The Netherlands) supplemented with 10% FCS, 1 mm pyruvate and penicillin/streptomycin. MCF10A (RRID:CVCL_0598) cells were cultured in DMEM/F12 containing 5% horse serum, 10 mm HEPES, and 100 U·mL^−1^ Penicillin/Streptomycin, supplemented with 20 ng·mL^−1^ EGF, 100 ng·mL^−1^ Cholera toxin, 10 μg·mL^−1^ insulin, and 0.5 μg·mL^−1^ hydrocortisone. All cell lines were incubated at 37 °C with 5% CO_2_ in a humidified incubator. All experiments were performed using Mycoplasma‐free cells, regularly verified by PCR.

### DNA constructs

Short hairpin RNA sequences targeting FTO or nontarget ‘scrambled’ shRNA control (Sigma‐Aldrich, Merck, Dordrecht, The Netherlands) were ordered as forward and reverse oligos, they were annealed and inserted into the pLKO1puro vector using AgeI and EcoRI restriction sites. ShFTO1 fw: 5′‐CCG GTC ACC AAG GAG ACT GCT ATT TCT CGA GAA ATA GCA GTC TCC TTG GTG ATT TTT G‐3′, shFTO1 rv: 5′‐AAT TCA AAA ATC ACC AAG GAG ACT GCT ATT TCT CGA GAA ATA GCA GTC TCC TTG GTG A‐3′, shFTO2 fw: 5′‐CCG GTA GTC TGA CTT GGT GTT AAA TCT CGA GAT TTA ACA CCA AGT CAG ACT ATT TTT G‐3′, shFTO2 rv: 5′‐AAT TCA AAA ATA GTC TGA CTT GGT GTT AAA TCT CGA GAT TTA ACA CCA AGT CAG ACT A‐3′, shWTAP fw: 5′‐CCG GAT GGC AAG AGA TGA GTT AAT TCT CGA GAA TTA ACT CAT CTC TTG CCA TTT TTT G‐3′, shWTAP rv: 5′‐AAT TCA AAA AAT GGC AAG AGA TGA GTT AAT TCT CGA GAA TTA ACT CAT CTC TTG CCA T‐3′. For rescue of FTO expression, human FTO (from pCMV‐FTO, HG12125‐UT, Sino Biological, Eschborn, Germany) was subcloned and inserted into the pcDNA3.1+ expression vector using KnpI/XbaI. The 3xFLAG‐FTO construct was produced by annealing the following forward and reverse oligos to generate 3xFLAG‐N (fw: 5′‐CTA GCT ATG GAC TAC AAA GAC CAT GAC GGT GAT TAT AAA‐3′; rv: 5′‐TAC TAG AAA TAT TAG TGG CAG TAC CAG AAA CAT CAG GTA TC‐3′) and 3xFLAG‐C (fw: 5′‐GAT CAT GAC ATC GAT TAC AAG GAT GAC GAT GAC AAG GGA A‐3′; rv: 5′‐TCG AAA GGG AAC AGT AGC AGT AGG AAC ATT AGC TAC AG‐3′) sequences and ligating these together into the human FTO pcDNA3.1+ expression vector described above via NheI/HindIII restriction sites to produce a fusion at the N‐terminus of FTO. For the 3xFLAG control vector the 3xFLAG‐N and 3xFLAG‐C sequences were ligated into an empty pcDNA3.1+ expression vector using the same restriction sites. Subsequently, the 3xFLAG‐FTO and the 3xFLAG control sequences were cloned into the lentiviral expression vector pLVX‐IRES‐neo using NheI and XbaI restriction sites. For ectopic LIP expression, human LIP was cloned and inserted into the lentiviral expression vector pLVX‐IRES‐neo as described in [40].

### Knockdown cells

For knockdown cells, 3.8 × 10^6^ HEK293T cells were seeded in 10 cm dishes one day prior to calcium phosphate transfection of pLKO.1 constructs along with pMDL‐RRE, pCMV‐VSVg and pRSV‐Rev expression constructs. After 2 days virus was harvested and used for transduction of target cells followed by selection with either 0.25 μg·mL^−1^ (MDA‐MB‐231), 0.5 μg·mL^−1^ (MCF‐7) or 1 μg·mL^−1^ (MCF10A) puromycin. For ectopic LIP expression concomitant with nontarget ‘scrambled’ sh control RNA or FTO sh RNAs in MDA‐MB‐231 cells, HEK293T cells were cotransfected with pLKO.1 constructs and either human LIP pLVX‐IRESneo vector or empty pLVX‐IRESneo vector as a control along with pMDL‐RRE, pCMV‐VSVg and pRSV‐Rev expression constructs. Virus was harvested and used for transduction of target cells as described above and cells were selected with both puromycin (0.5 μg·mL^−1^) and Geneticin (1 mg·mL^−1^). For FTO‐overexpressing/rescued cells, MDA‐MB‐231 cells were seeded in 6‐well plates one day prior to FuGene transfection with pcDNA3.1+ empty vector or FTO according to the manufacturer's instructions. Cells were selected with 700 μg·mL^−1^ Geneticin.

### MDA‐MB‐231 FTO knockout cells and re‐expression of 3xFLAG‐FTO

Guide RNA 5′‐CACCGCGGTGGGTGGAACTAAACCG‐3′ targeting FTO's fifth exon was designed using chopchop (https://chopchop.cbu.uib.no/). MDA‐MB‐231 cells were seeded in 6‐well plates 1 day prior to transfection with pSpCas9(BB)‐2A‐Puro (PX459) V2.0 (a gift from Feng Zhang, Addgene plasmid #62988) using X‐treme GENE™ 360 DNA transfection reagent (Sigma‐Aldrich, Merck, Dordrecht, The Netherlands). After two days, cells were selected with 0.75 μg·mL^−1^ puromycin and individual clones were grown out and assayed for FTO expression by immunoblotting and for gene disruption by Sanger sequencing. To express 3xFLAG‐FTO or 3xFLAG in these cells, one FTO knockout clone was selected and lentivirally infected with supernatant from HEK293T cells transfected with the 3xFLAG‐FTO or the 3xFLAG control pLVX‐IRES‐neo or empty vector plasmids respectively. Infected cells were selected using 1 mg·mL^−1^ Geneticin.

### Immunoblotting

Cells were washed twice in ice‐cold PBS prior to harvesting by scraping in ice‐cold PBS. Cells were lysed by sonication in 50 mm Tris pH 7.5, 150 mm NaCl, 1 mm EDTA, and 1% Triton‐X 100 supplemented with protease and phosphatase inhibitors (Roche, Woerden, The Netherlands). Equal amounts of protein were separated by SDS/PAGE using Mini‐PROTEAN® TGX™ Precast Protein Gels (#4561094; Bio‐Rad, Veenendaal, The Netherlands) and transferred to 0.2 μm PVDF membranes by a Trans‐Blot Turbo System (Bio‐Rad, Veenendaal, The Netherlands), using RTA Midi kit (#1704273; Bio‐Rad, Veenendaal, The Netherlands) following the manufacturer's instructions. Blots were washed in TBS‐T (50 mm Tris, 150 mm NaCl, 0.02% Tween‐20) and blocked/stained in 5% milk or BSA in TBS‐T. Following overnight incubation with primary antibody, blots were stained with secondary antibodies (mouse or rabbit IgG HRP‐linked antibody, Thermo Fisher Scientific, Sittard, The Netherlands, 1:5000), and signals were visualized by chemiluminescence (ECL, Amersham, Merck, Dordrecht, The Netherlands) on an ImageQuant LAS 4000 mini biomolecular imager (GE Healthcare, Amersfoort, The Netherlands). The supplied software was used for image quantification. The following primary antibodies were used for detection: FTO: NB110‐60935, Novus Biologicals, Abingdon, UK (1:1666); β‐Actin: 691001, MP Bio, Heerlen, The Netherlands (1:5000); C/EBPβ: ab32358, Abcam, Cambridge, UK (1:1000); WTAP: #56501 (1:1000), Cell Signaling Technology, Leiden, The Netherlands; COL1A1: #72026 (1:1000), Cell Signaling Technology, Leiden, The Netherlands; SMAD6: NB100‐56440, Novus Biologicals, Abingdon, UK (1:1000), FLAG‐M2: F‐3165, Sigma Aldrich, Merck, Dordrecht, The Netherlands (1:1000). DICER protein levels were examined by tris‐acetate immunoblotting as described [53] with minor modifications. Briefly, 80 μg protein per sample was loaded on 7% Tris‐acetate polyacrylamide gels (3% stacking) and transferred overnight at 20 V to 0.45 μm PVDF membrane by tank blotting in bicine/bis‐tris transfer buffer. The membranes were further processed as described, and the primary antibody was DICER (sc‐136 979; Santa Cruz Biotechnology, Heidelberg, Germany (1:1000)).

### RT‐qPCR

RNA was isolated from cells using the RNeasy Kit (Qiagen, Venlo, The Netherlands) according to the manufacturer's instructions. 1 μg of total RNA was reverse transcribed using a Transcriptor First Strand cDNA Synthesis Kit (Roche, Woerden, The Netherlands) with random hexamer primers. Gene expression was analyzed using SYBR Green Master Mix (Bio‐Rad, Veenendaal, The Netherlands) on a LightCycler 480 (Roche, Woerden, The Netherlands) and normalized to β‐actin expression. Oligo sequences are listed in Table 1.

**Table 1:** Sequences of oligonucleotides used in this study.

Gene	Forward primer 5′‐3′	Reversed primer 5′‐3′
*ACTB*	CCAACCGCGAGAAGATGA	CCAGAGGCGTACAGGGATAG
*ALKBH5*	CGGCGAAGGCTACACTTACG	CCACCAGCTTTTGGATCACCA
*COL12A1*	ATCCCATACAACGGGCAAGG	AATAAGCACGTGCGCAAACA
*CTHRC1*	ATCCCCAAGGGGAAGCAAAAG	GCTGGCCCTTGTAAGCACA
*E2F1*	CCTGGAAACTGACCATCAGTACCT	GGATTTCACACCTTTTCCTGGAT
*FN1*	CGGTGGCTGTCAGTCAAAG	AAACCTCGGCTTCCTCCATAA
*FTO*	GGAGGGTGTGACAAATGCTGTG	GCAGAGGCATCGAAGCATCATC
*LRRC15*	CTGCTGGCTATGCCACTGAA	GAGCAGGTACACTCGCTAGG
*MMP1*	CATGCTTTTCAACCAGGCCC	GTCCAAGAGAATGGCCGAGT
*SMARCD3*	ACTGGATCAAACCATCATGCG	CAATGCTGCCGTCGGAATC
*TNC*	TCCCAGTGTTCGGTGGATCT	TTGATGCGATGTGTGAAGACA
*SMARCD3*	ACTGGATCAAACCATCATGCG	CAATGCTGCCGTCGGAATC
*MYC*	GGACCCGCTTCTCTGAAAGG	TAACGTTGAGGGGCATCGTC
*HPRT*	CATTATGCTGAGGATTTGGAAAGG	CTTGAGCACACAGAGGGCTACA
*DICER1*	TTCAGATCTCAAGGTTGGGGA	TCAAGGCGACATAGCAAGTCAT
*CEBPb*	TTTCGAAGTTGATGCAATCG	CAACAAGCCCGTAGGAACAT
*EEF1A1*	CGGTCTCAGAACTGTTTGTTTC	AAACCAAAGTGGTCCACAAA
FTO geno	CATGTCCTGAGCAGAGGTTGT	CTGGTCATCTCCTGCCCTTG
FTO geno seq	GTCACCTACTTATAAGGGATTGATTAAAG	

### Cell migration assays

Cells were serum starved in their canonical medium with 0.1–1% serum. For scratch assays, 15 000 MDA‐MB‐231 cells were seeded in 96‐well ImageLock plates compatible with the Incucyte Zoom system (Sartorius, Nieuwegein, The Netherlands). 24 h after seeding a scratch wound was made using the WoundMaker Tool (Sartorius, Nieuwegein, The Netherlands) and the cells were incubated in low‐serum medium after washing twice. Cells were imaged every 2 h for 48 h and the relative wound density was calculated using the provided software according to the manufacturer's instructions. For Transwell migration assays, 20 000 cells were seeded in 0.1–1% FCS for MDA‐MB‐231 cells and in 1% horse serum supplemented with EGF, Cholera toxin, insulin, and hydrocortisone for MCF10A cells in the upper compartment of 8 μm Corning Transwell polycarbonate membrane cell culture inserts (3422; Corning, Amsterdam, The Netherlands) and attracted to the bottom by medium with 10% serum (MDA‐MB‐231 cells) or with 5% horse serum supplemented with EGF, Cholera toxin, insulin, and hydrocortisone (MCF10A cells). After 48 h, the cells were fixed with 96% ethanol on ice and stained with 0.5% crystal violet in 10% ethanol in water. Inserts were rinsed with demi water, air dried and imaged using a Leica DMC2900 digital microscope. For quantification, at least 3 inserts with 3 random images from each insert per condition were analyzed using ImageJ. To determine the effect of entacapone on cell migration, cells were pretreated with 25 μm entacapone (SML‐0654; Sigma‐Aldrich, Dordrecht, The Netherlands) for 24 h prior to seeding as described with 25 μm entacapone in both low‐serum and attractant media.

### Clonogenic and cell viability assays

For clonogenic assays, 3000 cells were seeded in 5 mL of their normal medium in 6‐well plates and incubated for 7 days. Cells were washed twice in PBS, fixed in 96% ethanol on ice and washed again twice in PBS before staining with 0.5% crystal violet in 10% ethanol in water. Wells were washed with demi water, air dried and imaged prior to quantification using imagej. For cell viability assays, 2500 (MDA‐MB‐231) or 7500 (MCF‐7) cells were seeded per well in 96‐well plates with the indicated drug concentrations of entacapone and/or HLM006474 (SML1260; Sigma‐Aldrich, Merck, Dordrecht, The Netherlands). After 72 h cell viability was determined using a CellTiter‐Fluor™ Cell Viability Assay (Promega, Leiden, The Netherlands) according to the manufacturer's instructions. For MCF10A, FTO knockdown and ‘scrambled’ control cells, 3000 cells were seeded per well in 96 well plates in the presence of puromycin and the culture medium was renewed after 48 h. The relative number of viable cells as a measure of cell proliferation was determined after 72 h using a CellTiter‐Glo™ Assay (Promega, Leiden, The Netherlands) according to the manufacturer's instructions.

### m6A dot blotting

mRNA was isolated from total RNA using Dynabeads™ Oligo(dT)25 (Thermo Fisher Scientific, Sittard, The Netherlands) and diluted to 50 (MDA‐MB‐213) or 75 (MCF‐7) ng·μL^−1^ in RNase free water. Samples were denatured for 3 min at 95 degrees and 2 μL was spotted on Hybond®‐N+ hybridization membrane (Amersham, Merck, Dordrecht, The Netherlands). mRNA was crosslinked to the membrane using a Stratalinker 2400 UV crosslinker (twice 120 000 μJ·cm^−2^) and the membrane was washed for 10 min in TBS‐T. Membranes were stained with 0.05% methylene blue in 0.5 m acetic acid for 15 min and washed three times for 2 min in Milli‐Q water. Following imaging for loading control the membrane was blocked in 5% BSA in TBS‐T and incubated overnight at 4 °C using N6‐methyladenosine antibody (ab151230; Abcam). The next day, the membranes were washed three times in TBS‐T, stained with 1:5000 rabbit IgG HRP‐linked antibody (Thermo Fisher Scientific, Sittard, The Netherlands) for 1 h and washed again three times in TBS‐T. The m6A signal was developed using chemiluminescence as described for immunoblotting.

### Ribosome profiling

Preparation of ribosome‐protected footprints for deep sequencing was essentially performed as described [54]. Briefly, 30 × 10^6^ cells were harvested in ice‐cold PBS with 100 μg·mL^−1^ cycloheximide (Sigma‐Aldrich, Merck, Dordrecht, The Netherlands) and 10% was kept aside for RNA‐sequencing. Cells were lysed in lysis buffer (20 mm Tris/HCl pH 7.5, 10 mm MgCl_2_, 100 mm KCl, 1% Triton X‐100, 2 mm DTT, 100 μg·mL^−1^ cycloheximide and protease inhibitors (EDTA‐free, Roche, Woerden, The Netherlands)) and lysates were treated with RNAseI (Ambion, Thermo Fisher Scientific, Sittard, The Netherlands) before ultracentrifugation over a sucrose gradient using an SW‐41Ti rotor at 222,200 x g for 2 h at 4 °C. Cytosolic ribosome fractions were collected and digested with PCR grade proteinase K (Roche, Woerden, The Netherlands) in the presence of 1% SDS prior to RNA isolation using TRIzol (Invitrogen, Thermo Fisher Scientific, Sittard, The Netherlands). RNA fragments were run on a denaturing urea polyacrylamide gel and correctly sized fragments were eluted in 0.3 M sodium acetate pH 5.5 and recovered by ethanol precipitation. RNA was 3′ dephosphorylated using T4 Polynucleotide Kinase (NEB, Bioké, Leiden, The Netherlands) and preadenylated linkers were ligated to the RNA fragments using T4 RNA Ligase 2 K277Q (NEB, Bioké, Leiden, The Netherlands). After 5′ deadenlyase and RecJ exonuclase treatment, fragments were cleaned using an Oligo Clean & Concentrator kit (Zymo, Freiburg Germany) before rRNA depletion by subtractive hybridization using MyOne Streptavidin C1 DynaBeads (Thermo Fischer Scientific, Sittard, The Netherlands) and biotinylated rRNA oligos. Following ethanol precipitation, samples were reverse transcribed using SuperScript III RT (Invitrogen, Thermo Fisher Scientific, Sittard, The Netherlands) and again ethanol precipitated prior to gel purification using urea PAGE. Fragments were circularized by CircLigase II (Epicentre, LGC, Den Haag, The Netherlands), recovered by ethanol precipitation, and subjected to library construction PCR using Q5 HF mastermix (NEB, Bioké, Leiden, The Netherlands), which was size selected using nondenaturing PAGE and recovered by ethanol precipitation. Library quality was confirmed using a Bioanalyzer 2100 (Agilent, Middelburg, The Netherlands) with the DNA 7500 kit (Agilent, Middelburg, The Ntherlands) and libraries were quantified by Qubitt dsDNA BR assay (Thermo Fisher Scientific, Sittard, The Netherlands) prior to high‐throughput 75 bp single‐end sequencing on an Illumina NextSeq machine. Translation efficiencies were calculated from ribosome profiling and RNA sequencing data using RiboDiff version 0.2.1 [55] and differential codon occupancy was determined using diricore as described [39].

### RNA sequencing

RNA was isolated from a portion of cells processed for ribosome profiling using the RNeasy kit (Qiagen, Venlo, The Netherlands). mRNA was isolated by NEXTFLEX® Poly(A) Beads (Perkin Elmer, Revvity, Groningen, The Netherlands), quality checked by RNA 6000 nano chip (Agilent, Middelburg, The Netherlands) on a Bioanalyzer 2100 (Agilent, Middelburg, The Netherlands) and sequencing libraries were prepared using NEXTFLEX® Rapid Directional qRNA‐Seq™ Library Prep Kit (Perkin Elmer, Revvity, Groningen, The Netherlands) according to the manufacturer's instructions. Library quality was confirmed using a Bioanalyzer 2100 (Agilent, Middelburg, The Netherlands) with the DNA 7500 kit (Agilent, Middelburg, The Netherlands) and libraries were quantified by Qubitt dsDNA BR assay (Thermo Fisher Scientific, Sittard, The Netherlands) prior to high‐throughput 75 bp paired‐end sequencing on an Illumina NextSeq 500 machine. Reads were demultiplexed by sample‐specific barcodes and changed to fastq files using bcl2fastq version 1.8.4 (Illumina, Eindhoven, The Netherlands). An in‐house Perl script was used to remove barcodes, and low‐quality trimming was performed on both mates using Cutadapt [56] version 1.12 (settings: q = 15, O = 5, e = 0.1, m = 20) prior to poly A tail removal using poly A or poly T as an adapter. Reads shorter than 20 bases after trimming were discarded. The trimmed reads were aligned to GRCh38, Ensembl release 95 using HISAT2 [57] version 2.1.0 (settings: k = 1000, ‐‐norc). Reads were only mapped against the forward strand. An in‐house Perl script was used to remove reads that mapped to multiple genes. A script from Bio Scientific (dqRNASeq; settings: s = 8, q = 0, m = 1) was used to determine the number of unique fragments per transcript. Unique fragments per gene were determined using an in‐house Perl script. Differentially expressed genes were identified using the Bioconductor (https://www.bioconductor.org/) package edgeR  [58] version 3.32.1 using R version 4.0.3. Gene expression was normalized using trimmed mean of m‐values (TMM), and multiple testing was corrected for using Benjamini‐Hochberg correction. Genes with a false discovery rate (FDR) <0.05 and a fold change of at least 1.5‐fold were considered differentially expressed. Data were analyzed further using custom R‐scripts and topGO version 2.44.0. For gene set enrichment analysis (GSEA), the desktop application (https://www.gsea‐msigdb.org/gsea, The Broad Institute) was used to compare the ranked gene list to hallmark signatures using the gene set permutation type with 1000 permutations [36].

### mRNA stability assays

Cells were seeded in 6 cm dishes one day prior to treatment with 1 μg·mL^−1^ actinomycin D (Sigma‐Aldrich, Merck, Dordrecht, The Netherlands) for the indicated time. Cells were washed twice in ice‐cold PBS and harvested by scraping in ice‐cold PBS. RNA was isolated and processed to cDNA as described above for RT‐qPCR. The fraction of remaining mRNA after actinomycin D treatment was calculated by the 2^−ΔΔCt^ method.

### MeRIP‐qPCR analysis

mRNA was isolated as described above and 1 μg was precipitated with 2 μL anti‐m6A antibody (ab151230; Abcam, Cambridge, UK) or normal rabbit IgG (sc‐2027; Santa Cruz Biotechnology, Heidelberg, Germany) in IPP buffer (10 mm Tris/HCl (pH 7.5), 150 mm NaCl, 0.1% NP‐40, 1 mm DTT) at 4 °C for 4 h under constant rotation. Then, 20 μL prewashed Dynabeads Protein A (Thermo Fischer Scientific, Sittard, The Netherlands) in IPP buffer was added, followed by another 2 h on the rotator at 4 °C. The beads were washed 5x in IPP buffer and transferred to fresh tubes after the 3rd and 5th washes. Samples were eluted twice in RLT+ buffer (Qiagen, Venlo, The Netherlands) with β‐mercaptoethanol for 10 min at room temperature and RNA was recovered by ethanol precipitation. Pellets were resuspended in nuclease‐free water and subjected to reverse transcription along with 100 ng mRNA input samples followed by RT‐qPCR analysis. Transcript recovery over input was calculated and normalized to a positive control mRNA (EEF1A1).

### RNA immunoprecipitation (RIP)

FTO‐specific RIP was performed as described in [54]. MDA‐MB‐231 FTO knockout cells expressing 3xFLAG‐FTO and controls (empty vector or 3xFLAG) were seeded in 10 cm plates. After 24 h, cells were washed, harvested in ice‐cold PBS, lysed, and pooled from two plates per condition. Lysates were sonicated and treated with DNase after which input controls for protein and RNA were taken (5% each).

Then lysates were pre‐cleared with Protein A‐coupled magnetic beads and incubated with anti‐FLAG antibody‐conjugated beads or Protein A‐coupled beads for the “no antibody” control, both pre‐treated with yeast tRNA for blocking. After 4 h of incubation, the beads were washed four times and 10% of the beads were separated to check success of the immunoprecipitation. Co‐precipitated RNA was eluted from the rest of the beads using RLT buffer (RNeasy Mini Kit, Qiagen, Venlo, The Netherlands) supplemented with β‐mercaptoethanol. RNA was precipitated overnight at −80 °C, treated with DNase, and reverse transcribed into cDNA as described above which was used for qPCR to detect CEBPB mRNA levels. CEBPB mRNA enrichment was calculated using the ΔΔCT method as described in [55].

### Statistical analyses

Analysis of ribosome and RNA‐sequencing data is described above. For all remaining analyses Student's *t*‐test or one‐, or two‐way ANOVA with Dunnett's *post hoc* test (when appropriate) was used as described in the figure legends. Statistical testing was performed using GraphPad Prism 6 and statistically significant differences are indicated: *P < 0.05, **P < 0.01, ***P < 0.001, ****P < 0.0001.

## Conflict of interest

The authors declare no competing interests.

## Author contributions

HRZ and CFC conceived the project with input from CM. HRZ, CM and GK performed experiments and analyzed the data. FL‐P provided expertise and lab resources on the ribosome profiling experiments. RW helped with mapping and processing the RNA‐sequencing data. ES mapped and analyzed the ribosome profiling data. HRZ, CM and CFC wrote the manuscript. CFC and FL‐P supervised the work.

### Peer review

The peer review history for this article is available at https://www.webofscience.com/api/gateway/wos/peer‐review/10.1111/febs.70033.

## Supporting information


**Table S1.** Data transcriptome analysis of MDA‐MB‐231 cells.
**Table S2.** Data ribosome profiling of MDA‐MB‐231 cells.

## Data Availability

RNA‐seq and the Ribo‐seq data that support the findings in this study are openly available in the ArrayExpress database at EMBL‐EBI (www.ebi.ac.uk/arrayexpress) under accession numbers E‐MTAB‐14808 and E‐MTAB‐14809.
