# Supplementary material for: The m6A demethylase FTO promotes C/EBPβ‐LIP translation to perform oncogenic functions in breast cancer cells
Source: FEBS J. 2025 Feb 28;292(10):2688–709. doi: 10.1111/febs.70033 (PMC12103066; doi:10.1111/febs.70033)
Supplement: Supplementary file 1 — Table S1. Data transcriptome analysis of MDA‐MB‐231 cells. Table S2. Data ribosome profiling of MDA‐MB‐231 cells. [file FEBS-292-2688-s001.pdf]

Supplementary Table 1 Figure 4A

SCR1_repl1	SCR2_repl2	shFTO1_repl1	shFTO_repl2	logFC	logCPM	PValue	FDR	updown	external_gene_name	ensembl_gene_id
6926	5525	6169	5942	-0,17716358	9,327001895	0,008063304	0,049413987	down	SPTAN1	ENSG00000197694
2954	2332	3376	3228	0,184137371	8,272870104	0,007814217	0,048230116	up	CALM1	ENSG00000198668
8219	7347	10228	9373	0,186807502	9,839231898	0,007332916	0,045854767	up	SRRM2	ENSG00000167978
2259	1787	2613	2454	0,186985061	7,889405048	0,007595115	0,047121273	up	STARD7	ENSG00000084090
40439	32447	46674	44617	0,187222356	12,0591094	0,003046427	0,022635391	up	HSP90AA1	ENSG00000080824
3702	3141	4426	4170	0,187493643	8,651042203	0,005269828	0,035018463	up	EIF5	ENSG00000100664
11763	9189	10711	9481	-0,190972381	10,06982222	0,005523084	0,036404065	down	TUBB4B	ENSG00000188229
1672	1423	1999	1899	0,191465391	7,509817576	0,007254865	0,045501131	up	MED1	ENSG00000125686
9975	7664	8718	8245	-0,191486871	9,820230253	0,005129468	0,034260437	down	ATP1A1	ENSG00000163399
2081	1605	2361	2266	0,192157856	7,756559873	0,00808353	0,049498871	up	SRP68	ENSG00000167881
5803	4650	6684	6459	0,192912135	9,261191347	0,003879399	0,027571251	up	LOXL2	ENSG00000134013
1507	1294	1809	1724	0,193269118	7,367464748	0,007673011	0,047528595	up	FARSB	ENSG00000116120
1912	1545	2258	2095	0,193389976	7,667416834	0,005652053	0,0370497	up	SPAG9	ENSG00000008294
2144	1748	2557	2347	0,193688523	7,838776658	0,005292727	0,035139273	up	CNBP	ENSG00000169714
4696	4067	5680	5395	0,195118207	9,01298534	0,003996355	0,028234486	up	TMEM123	ENSG00000152558
1535	1215	1763	1701	0,195600776	7,338090832	0,007281557	0,045613272	up	USP39	ENSG00000168883
4356	3677	5278	4873	0,195912795	8,886390345	0,003213787	0,023612693	up	FOXM1	ENSG00000111206
2851	2290	3305	3176	0,196229369	8,24028716	0,004248046	0,029621868	up	PKP4	ENSG00000144283
2167	1910	2711	2454	0,196880962	7,911791708	0,006780085	0,043168489	up	DLGAP4	ENSG00000080845
2286	1891	2011	2001	-0,197116456	7,744606969	0,006118123	0,039586805	down	SMAD3	ENSG00000166949
2099	1670	2441	2313	0,197132258	7,793032558	0,004947456	0,033259326	up	INCENP	ENSG00000149503
2317	1838	2038	1947	-0,197718806	7,734369211	0,00527002	0,035018463	down	COL5A1	ENSG00000130635
1541	1308	1923	1685	0,198037551	7,39391582	0,007841475	0,048359918	up	ZNF146	ENSG00000167635
3927	3358	3567	3438	-0,198095312	8,548368198	0,003753224	0,026772535	down	HK1	ENSG00000156515
2912	2417	3452	3288	0,198886828	8,295382676	0,003317174	0,024263963	up	PDCD6IP	ENSG00000170248
1308	1123	1615	1465	0,198889716	7,166410471	0,007337915	0,045855323	up	MRPL19	ENSG00000115364
2098	1731	2522	2326	0,200115569	7,819380305	0,003890167	0,027635131	up	RFWD3	ENSG00000168411
2689	2357	3271	3131	0,200335869	8,220709406	0,00461305	0,031613437	up	SEPT11	ENSG00000138758
1092	908	1011	908	-0,20056511	6,681514263	0,007177659	0,045199461	down	MAN2B2	ENSG00000013288
2566	2155	2329	2199	-0,201040693	7,920159461	0,003580996	0,025756964	down	IPO9	ENSG00000198700

18923	14970	16514	15892	-0,201271212	10,75967756	0,001983262	0,016226593	down	AXL	ENSG00000167601
3262	2538	3734	3594	0,201398611	8,41495326	0,004092868	0,028772365	up	GCN1	ENSG00000089154
1286	1062	1136	1113	-0,201443297	6,912032125	0,007576626	0,047062971	down	ITGAV	ENSG00000138448
1262	1097	1560	1435	0,201563221	7,125170175	0,006937045	0,043951601	up	RBPJ	ENSG00000168214
2472	1954	2260	1977	-0,201844154	7,823273928	0,005986825	0,038883065	down	JUN	ENSG00000177606
4353	3631	3870	3777	-0,202003069	8,677194672	0,002935144	0,022029897	down	CALM3	ENSG00000160014
9494	7710	8225	8225	-0,202127408	9,782861637	0,002915009	0,021921148	down	SPTBN1	ENSG00000115306
4644	3844	4222	3907	-0,202783042	8,764642136	0,002221757	0,017702241	down	PRRC2B	ENSG00000130723
3110	2377	2745	2493	-0,203111083	8,130532757	0,00531025	0,035240545	down	PSMC3	ENSG00000165916
2568	1938	2981	2717	0,203155047	8,05045008	0,006099812	0,039484778	up	BCAT1	ENSG00000060982
1538	1281	1896	1685	0,203723949	7,380743612	0,005250519	0,034948699	up	RANBP1	ENSG00000099901
6017	5075	7277	6820	0,204501885	9,356203327	0,001759933	0,014733151	up	SMC1A	ENSG00000072501
1185	921	1375	1295	0,205141466	6,958420384	0,006655003	0,042546541	up	TNKS2	ENSG00000107854
2543	2073	2917	2938	0,205206486	8,090749118	0,004432277	0,030659331	up	TMED10	ENSG00000170348
1870	1443	1637	1522	-0,205258494	7,402911348	0,005547058	0,036524126	down	FKBP10	ENSG00000141756
924	810	848	811	-0,205969859	6,47567517	0,008076415	0,04947481	down	MAP4K5	ENSG00000012983
1754	1530	1580	1560	-0,20666637	7,396116518	0,005570752	0,036630847	down	CAPZB	ENSG00000077549
880	681	1029	953	0,206810425	6,52859801	0,007534317	0,046875163	up	TIMM50	ENSG00000105197
3293	2827	3921	3871	0,207215604	8,501364447	0,003206926	0,023590881	up	BAZ1B	ENSG00000009954
2255	1766	2698	2411	0,207426522	7,890882077	0,004052675	0,028541495	up	YAP1	ENSG00000137693
7957	6062	9102	8668	0,207598637	9,68899905	0,002920702	0,021945007	up	XPO1	ENSG00000082898
921	717	794	766	-0,20768605	6,387828027	0,007935612	0,048793747	down	LRBA	ENSG00000198589
5234	4537	5112	4248	-0,207791482	8,968305989	0,007485877	0,046626106	down	CDC37	ENSG00000105401
3236	2688	3085	2577	-0,208034029	8,243652583	0,006429094	0,041357667	down	RAB1B	ENSG00000174903
3091	2532	2669	2686	-0,208556496	8,167384291	0,003590986	0,025816865	down	DSG2	ENSG00000046604
1366	1111	1602	1551	0,20938866	7,196412192	0,004170158	0,029170443	up	SLC39A9	ENSG00000029364
1921	1594	1683	1665	-0,209509762	7,490137402	0,00385116	0,027383089	down	EMP1	ENSG00000134531
1269	1094	1553	1462	0,209535325	7,131514138	0,004608588	0,031610764	up	TOPBP1	ENSG00000163781
1658	1249	1888	1803	0,20956344	7,422904411	0,005613389	0,036827343	up	AMD1	ENSG00000123505
2850	2283	3304	3225	0,209610532	8,245079239	0,002573615	0,01991314	up	PPP1CC	ENSG00000186298
1096	866	942	924	-0,209798787	6,64731308	0,006186541	0,039946276	down	SPR	ENSG00000116096
1919	1429	2204	2047	0,209882072	7,625687458	0,005718739	0,037423642	up	SEH1L	ENSG00000085415

959	784	1143	1078	0,210079441	6,6913095	0,004417773	0,030612941	up	ELMSAN1	ENSG00000156030
717	583	880	778	0,210618015	6,269660473	0,007722239	0,047773247	up	TM7SF3	ENSG00000064115
1862	1599	1715	1585	-0,211050981	7,468892986	0,003199623	0,023559482	down	PAFAH1B2	ENSG00000168092
884	765	1051	1054	0,211289946	6,614709372	0,007260461	0,045517844	up	INTS6	ENSG00000102786
773	602	887	865	0,212213602	6,349531422	0,006788157	0,043202171	up	C12orf49	ENSG00000111412
905	767	1116	1021	0,212562038	6,634344205	0,004750039	0,032323993	up	MEPCE	ENSG00000146834
838	682	755	690	-0,212775789	6,279549987	0,005822282	0,038037115	down	PPFIBP1	ENSG00000110841
2028	1559	1836	1569	-0,212920981	7,513556166	0,006643639	0,042491381	down	CCN2	ENSG00000118523
681	563	816	773	0,213169056	6,208449615	0,006397862	0,04117381	up	MSI2	ENSG00000153944
1343	1183	1652	1581	0,21317443	7,230669539	0,004497451	0,031018696	up	NCOA6	ENSG00000198646
3340	2792	4133	3709	0,213189894	8,505795038	0,001841112	0,015280933	up	NOP56	ENSG00000101361
710	598	871	801	0,213232613	6,281266065	0,005866492	0,038252402	up	CRCP	ENSG00000241258
2315	1775	2696	2517	0,214018784	7,91775472	0,003053592	0,02267778	up	KIF11	ENSG00000138160
3656	3318	3384	3265	-0,214069252	8,481623021	0,003849997	0,027383089	down	TPR	ENSG00000047410
7577	6171	6754	6297	-0,214317678	9,453884562	0,000965822	0,009031851	down	TUBA1C	ENSG00000167553
3360	3069	3156	2975	-0,214528344	8,364375603	0,004032477	0,028463791	down	SMURF2	ENSG00000108854
1792	1335	1516	1441	-0,214907155	7,313232133	0,005961611	0,03875172	down	KRT80	ENSG00000167767
1078	900	1260	1269	0,215202819	6,877029161	0,005586056	0,036710056	up	ATP2B1	ENSG00000070961
1550	1310	1875	1786	0,215320633	7,408577879	0,002644147	0,020337116	up	CLINT1	ENSG00000113282
3290	2715	2888	2807	-0,215715207	8,259275263	0,001664954	0,014113027	down	OGDH	ENSG00000105953
777	651	967	862	0,215736737	6,408654737	0,005883234	0,03833854	up	FHOD1	ENSG00000135723
1604	1450	1934	1983	0,215832968	7,50707839	0,007383135	0,046082279	up	ANTXR1	ENSG00000169604
1386	1188	1754	1547	0,2158856	7,257861611	0,004048201	0,028528984	up	KDELR1	ENSG00000105438
1565	1376	1434	1362	-0,21593382	7,233036345	0,00371644	0,02657118	down	ZMIZ1	ENSG00000108175
5361	4632	6457	6350	0,21612475	9,213536757	0,001768061	0,014783237	up	CSE1L	ENSG00000124207
4670	3897	5627	5343	0,216308722	8,989436423	0,001035903	0,009518664	up	TRIP12	ENSG00000153827
653	551	816	727	0,216436725	6,163956243	0,007396264	0,046145677	up	BRMS1	ENSG00000174744
3265	2889	2994	2857	-0,216624212	8,298367584	0,002352213	0,018514368	down	CAPNS1	ENSG00000126247
1726	1442	2059	1998	0,216855497	7,556128737	0,002304723	0,018225815	up	WAPL	ENSG00000062650
995	761	1137	1106	0,216923734	6,702765493	0,004785624	0,032494955	up	RBSN	ENSG00000131381
1380	1227	1645	1699	0,217124844	7,278837088	0,007130551	0,04497576	up	ZNF664	ENSG00000179195
1749	1353	1989	1972	0,217145469	7,521853961	0,003690857	0,026436958	up	GRB2	ENSG00000177885

1292	1138	1140	1166	-0,217318492	6,957250986	0,00670714	0,042809359	down	SVIL	ENSG00000197321
686	536	816	748	0,217511404	6,183045123	0,00645914	0,04153374	up	BRK1	ENSG00000254999
941	706	1054	1049	0,217672232	6,610125199	0,007021363	0,044341091	up	IST1	ENSG00000182149
1201	1002	1057	1030	-0,217904654	6,812899112	0,004141958	0,029038584	down	TOR1AIP1	ENSG00000143337
720	565	826	818	0,217968002	6,255698446	0,006467139	0,041567963	up	ZW10	ENSG00000086827
703	540	839	752	0,217994459	6,207087016	0,007250611	0,045492825	up	CRY1	ENSG00000008405
1957	1633	2368	2235	0,218134728	7,736882162	0,001719786	0,014467259	up	NRAS	ENSG00000213281
1558	1167	1371	1203	-0,218352077	7,113663073	0,006604042	0,042255529	down	KCNAB2	ENSG00000069424
1465	1079	1710	1540	0,218411453	7,234587747	0,006056355	0,039268953	up	LARP4B	ENSG00000107929
2293	1906	2836	2553	0,218764484	7,962754018	0,001745938	0,014639671	up	PAK2	ENSG00000180370
1929	1606	2461	2081	0,219068588	7,715049013	0,004152186	0,029057817	up	PRKAR2A	ENSG00000114302
735	556	873	780	0,219097551	6,261477112	0,007318274	0,04580632	up	STEAP3	ENSG00000115107
878	725	771	746	-0,219139152	6,353770773	0,004229353	0,029518032	down	TMEM164	ENSG00000157600
2496	2247	2295	2208	-0,219422101	7,922300047	0,003151023	0,023256768	down	PPIB	ENSG00000166794
1600	1317	1849	1889	0,219551516	7,437917782	0,003692748	0,026438305	up	AP1G1	ENSG00000166747
2006	1652	2384	2307	0,219567285	7,763978268	0,001734882	0,014554824	up	SRPK1	ENSG00000096063
922	799	1144	1068	0,220033561	6,680958842	0,003510233	0,02534189	up	NOL8	ENSG00000198000
1679	1367	1526	1356	-0,220063309	7,277665951	0,002697311	0,020633762	down	SELENON	ENSG00000162430
17447	15380	16036	15106	-0,220316794	10,71199201	0,000963535	0,009028325	down	MSN	ENSG00000147065
2619	2115	2333	2142	-0,220332992	7,912867135	0,00150683	0,01301422	down	WSB2	ENSG00000176871
2055	1695	2555	2263	0,22045275	7,800309206	0,002026811	0,016495993	up	PPRC1	ENSG00000148840
4037	3119	3455	3292	-0,220545537	8,505955136	0,001768772	0,014783237	down	RRM1	ENSG00000167325
941	751	1153	1019	0,220719568	6,653289272	0,003928867	0,027833656	up	NIP7	ENSG00000132603
1162	930	1366	1317	0,220808301	6,958726481	0,003168672	0,023364821	up	WWC2	ENSG00000151718
3197	2509	2721	2658	-0,221334489	8,180033738	0,001824385	0,015166417	down	NDC1	ENSG00000058804
1814	1408	1554	1483	-0,221765159	7,355370344	0,002726068	0,020802509	down	CAPN1	ENSG00000014216
1913	1524	2277	2134	0,221792762	7,673630469	0,001601113	0,013691459	up	CCDC6	ENSG00000108091
1386	1128	1249	1126	-0,222000716	7,000060662	0,002873373	0,02168154	down	MAPK12	ENSG00000188130
5730	5238	7652	6533	0,222312067	9,354824934	0,004947333	0,033259326	up	SET	ENSG00000119335
711	587	817	850	0,22244905	6,274557194	0,00746799	0,046537084	up	BTF3L4	ENSG00000134717
845	684	768	676	-0,222557313	6,28317921	0,004698281	0,032112502	down	UBE2E3	ENSG00000170035
660	511	792	712	0,222636139	6,124240735	0,006507629	0,041759079	up	C1orf109	ENSG00000116922

890	667	1019	977	0,222636188	6,53209729	0,005322244	0,03530505	up	TRAPPC8	ENSG00000153339
2873	2363	2507	2433	-0,223056063	8,058018542	0,001292011	0,011419255	down	MICAL2	ENSG00000133816
1200	1046	1534	1362	0,223119606	7,066315842	0,003562878	0,025674215	up	BCCIP	ENSG00000107949
9734	8387	12731	10668	0,223354089	10,07565436	0,002878747	0,021701	up	FTL	ENSG00000087086
719	604	880	823	0,223593071	6,303231404	0,003649974	0,026176707	up	GTF2I	ENSG00000263001
776	575	886	847	0,2236464	6,328365529	0,006155803	0,039764339	up	MMS22L	ENSG00000146263
747	638	677	631	-0,22377161	6,142333622	0,004872151	0,032940969	down	ARAF	ENSG00000078061
1169	989	1401	1377	0,224005185	7,007804597	0,002982573	0,022288918	up	UBA2	ENSG00000126261
747	615	640	644	-0,2240768	6,116757174	0,006337034	0,04086703	down	CAT	ENSG00000121691
1037	754	1194	1104	0,225099	6,733134801	0,005626419	0,036897238	up	HIGD1A	ENSG00000181061
623	472	708	699	0,225285966	6,028551163	0,007867589	0,048482455	up	VCPIP1	ENSG00000175073
894	705	1055	1002	0,225316806	6,574065652	0,003009201	0,022404989	up	TIMELESS	ENSG00000111602
955	725	799	780	-0,225317177	6,414821095	0,005717274	0,037423642	down	ALDH1B1	ENSG00000137124
1124	891	1017	881	-0,225666857	6,678278068	0,003753115	0,026772535	down	ALG12	ENSG00000182858
1151	953	1412	1300	0,225752426	6,971227379	0,002324205	0,018349048	up	CGGBP1	ENSG00000163320
1860	1503	1712	1458	-0,225874976	7,417272164	0,003080447	0,02285536	down	FAM83G	ENSG00000188522
821	711	710	734	-0,225927285	6,287346579	0,006382402	0,041091342	down	TSPAN3	ENSG00000140391
4383	3403	3803	3515	-0,225978035	8,625402103	0,001178761	0,010562601	down	CNN3	ENSG00000117519
1237	989	1436	1428	0,226094656	7,050865829	0,002844848	0,021497636	up	GEMIN5	ENSG00000082516
748	609	937	813	0,226290558	6,339930699	0,004555031	0,031340307	up	GRPEL1	ENSG00000109519
1144	950	1417	1285	0,22690538	6,965132401	0,002357132	0,018533621	up	CKAP2L	ENSG00000169607
2363	1823	2775	2610	0,227193471	7,958649479	0,001518206	0,013097902	up	TMED2	ENSG00000086598
842	694	1017	964	0,227228082	6,519151568	0,002720483	0,020780318	up	SMU1	ENSG00000122692
4287	3096	5030	4447	0,227295503	8,771976086	0,004813803	0,032643472	up	RANGAP1	ENSG00000100401
630	477	726	699	0,227529583	6,045428335	0,006485565	0,041669154	up	CHSY1	ENSG00000131873
611	492	753	670	0,22758237	6,04245013	0,005571644	0,036630847	up	STAMBPL1	ENSG00000138134
2208	1753	1903	1817	-0,22809873	7,651279417	0,001305496	0,011512237	down	POFUT1	ENSG00000101346
1768	1531	2193	2072	0,228144481	7,622534576	0,001421465	0,012394119	up	LARP4	ENSG00000161813
681	568	835	778	0,228502554	6,222892608	0,003365266	0,024523329	up	CSNK1E	ENSG00000213923
3626	2795	3115	2907	-0,228544223	8,345829771	0,001273933	0,011278734	down	PRSS23	ENSG00000150687
4405	3571	5307	4983	0,22863299	8,891258682	0,000544361	0,005643371	up	PTPN11	ENSG00000179295
1547	1333	1880	1844	0,229426026	7,427297557	0,00179693	0,014986322	up	TAOK1	ENSG00000160551

3647	3215	4652	4241	0,22944211	8,679971694	0,001121467	0,010176901	up	MAT2A	ENSG00000168906
1468	1186	1322	1172	-0,229568616	7,074257235	0,002154014	0,017286657	down	COX4I1	ENSG00000131143
482	402	599	544	0,230013207	5,727110259	0,007246276	0,045487701	up	SOS1	ENSG00000115904
2720	2069	2323	2161	-0,230379352	7,921415662	0,001730853	0,014536728	down	FOSL2	ENSG00000075426
496	381	576	556	0,230546298	5,713459405	0,00815135	0,049776764	up	IL4R	ENSG00000077238
906	739	1071	1054	0,230559653	6,619151663	0,00242598	0,018940696	up	ANKIB1	ENSG00000001629
505	420	606	590	0,230843646	5,792656917	0,006720767	0,042878714	up	SMYD4	ENSG00000186532
886	761	1097	1035	0,230851971	6,623229343	0,002288118	0,018116167	up	COPS2	ENSG00000166200
527	441	636	616	0,231073741	5,858233924	0,00597267	0,038807359	up	RIPK1	ENSG00000137275
1199	1009	1073	1001	-0,231259541	6,810285407	0,002120213	0,017064673	down	NEDD4	ENSG00000069869
1464	1244	1714	1788	0,231463583	7,338992224	0,003421699	0,024852928	up	SAMHD1	ENSG00000101347
783	588	929	841	0,231566105	6,354346451	0,004062386	0,02858396	up	CDC42EP4	ENSG00000179604
821	676	736	669	-0,231761776	6,249117344	0,002842769	0,021492386	down	SCAMP2	ENSG00000140497
690	546	610	549	-0,231785501	5,971828562	0,005400542	0,035702434	down	LRRC20	ENSG00000172731
4316	3556	3836	3551	-0,231884032	8,642148775	0,000496044	0,005250898	down	FRMD6	ENSG00000139926
1378	1095	1176	1140	-0,232235715	6,970459686	0,002084685	0,016869758	down	DOCK1	ENSG00000150760
10075	7400	8525	7787	-0,232262999	9,784855204	0,002017315	0,016442613	down	HSPA5	ENSG00000044574
683	562	868	745	0,232424466	6,219886588	0,004711493	0,032160342	up	CENPO	ENSG00000138092
1736	1433	1529	1443	-0,232463777	7,329705066	0,001140271	0,010271033	down	CLIP1	ENSG00000130779
564	419	664	606	0,232604622	5,877002498	0,007699732	0,047675092	up	POLR2G	ENSG00000168002
995	891	888	884	-0,232678482	6,585304437	0,003732459	0,026661159	down	MAGEB2	ENSG00000099399
2135	1780	2653	2420	0,232734645	7,869601577	0,000822484	0,007947946	up	EXOC7	ENSG00000182473
1357	1243	1698	1679	0,232814783	7,285051737	0,003504903	0,025323915	up	MORF4L1	ENSG00000185787
2215	1805	2713	2494	0,233554121	7,906908973	0,000737613	0,007245028	up	TASOR2	ENSG00000108021
791	576	890	874	0,233599334	6,349874825	0,005512384	0,036348962	up	POLR2D	ENSG00000144231
1377	1116	1254	1083	-0,233677578	6,982290654	0,002490749	0,019387893	down	CANT1	ENSG00000171302
652	591	821	793	0,233760619	6,222125343	0,004872523	0,032940969	up	NEO1	ENSG00000067141
2015	1731	2524	2338	0,233801514	7,808264404	0,000902035	0,008567405	up	SMC2	ENSG00000136824
1558	1292	1408	1264	-0,233991474	7,17622779	0,001412204	0,012327215	down	BRD1	ENSG00000100425
3229	2560	4056	3445	0,23407075	8,430969669	0,001818674	0,015143268	up	MCM5	ENSG00000100297
2683	1968	3112	2888	0,234093993	8,111097656	0,002337722	0,018437017	up	PFAS	ENSG00000178921
4937	3713	5850	5321	0,234135081	9,006470236	0,001299947	0,011476336	up	EPHA2	ENSG00000142627

1191	1042	1469	1430	0,234459018	7,064323436	0,002030857	0,016520263	up	GTF2A1	ENSG00000165417
2376	1959	2975	2649	0,234576933	8,016798772	0,000855823	0,008207534	up	KHDRBS1	ENSG00000121774
825	670	741	659	-0,234743295	6,245225541	0,002877801	0,021701	down	VPS13C	ENSG00000129003
1128	994	1394	1362	0,234782089	6,991431446	0,002372291	0,018633945	up	CTCF	ENSG00000102974
707	581	606	599	-0,235293933	6,03099496	0,00408175	0,028707196	down	PRMT3	ENSG00000185238
882	709	1078	985	0,235413299	6,573150373	0,001852531	0,015342912	up	IPO8	ENSG00000133704
882	786	1103	1065	0,235485578	6,645655135	0,002577487	0,019923242	up	R3HDM1	ENSG00000048991
837	710	723	725	-0,235559326	6,295977744	0,002981522	0,022288918	down	NEK2	ENSG00000117650
1363	1101	1165	1138	-0,23576558	6,964368363	0,001714958	0,014450124	down	CAMSAP2	ENSG00000118200
520	418	642	575	0,23584296	5,814256011	0,005660316	0,037088205	up	GNA15	ENSG00000060558
960	668	1109	993	0,235846799	6,599709742	0,007936957	0,048793747	up	MIEF1	ENSG00000100335
518	438	631	610	0,236012208	5,843367711	0,005253394	0,034952851	up	ARHGAP12	ENSG00000165322
4793	3648	5792	5134	0,236103439	8,97303603	0,001214876	0,010817548	up	IMPDH2	ENSG00000178035
1585	1227	1321	1302	-0,236216287	7,152371321	0,002051259	0,016651337	down	ZHX3	ENSG00000174306
2686	2257	3080	3327	0,236225808	8,207891077	0,003793243	0,027020765	up	CBX5	ENSG00000094916
1345	1181	1702	1584	0,236297125	7,243070933	0,001499908	0,012968857	up	CUL3	ENSG00000036257
1263	928	1032	1008	-0,236577058	6,790138899	0,00456133	0,031369747	down	NDUFS2	ENSG00000158864
1447	1136	1815	1537	0,236608027	7,269678387	0,002775513	0,021096887	up	MAF1	ENSG00000179632
2735	2149	3366	2968	0,236613906	8,186919951	0,001009805	0,009323398	up	U2AF2	ENSG00000063244
700	515	791	781	0,236745559	6,182846897	0,005561932	0,036597981	up	GID8	ENSG00000101193
881	700	794	684	-0,236775494	6,323879202	0,003306798	0,024199466	down	RNPEP	ENSG00000176393
839	677	770	648	-0,236935808	6,263982181	0,004201698	0,029351402	down	FURIN	ENSG00000140564
1587	1195	1288	1302	-0,236970412	7,135074779	0,00337136	0,024544704	down	RCN1	ENSG00000049449
2341	1944	2837	2727	0,236985354	8,001809544	0,000602615	0,006152538	up	FKBP4	ENSG00000004478
838	721	776	684	-0,237039027	6,306859821	0,002651602	0,020368753	down	TMEM104	ENSG00000109066
2103	1757	1858	1752	-0,237129291	7,61262526	0,000701337	0,006963598	down	CTSA	ENSG00000064601
4325	4118	4282	3672	-0,237234717	8,749847804	0,005223464	0,034798447	down	RPS23	ENSG00000186468
4795	3949	5924	5436	0,237266464	9,029435618	0,000321286	0,003633832	up	RPL7L1	ENSG00000146223
1067	847	1294	1190	0,237430494	6,839478244	0,001717453	0,014456404	up	PPP4R3B	ENSG00000275052
4078	3326	5071	4550	0,237657639	8,789251177	0,00045512	0,00487754	up	ETS1	ENSG00000134954
879	639	748	666	-0,237657818	6,261163954	0,006501665	0,041738057	down	TOE1	ENSG00000132773
1401	1207	1243	1197	-0,237690842	7,048518589	0,001440192	0,012536293	down	WBP2	ENSG00000132471

1287	1155	1170	1118	-0,23776707	6,955682963	0,002240407	0,017814278	down	DDA1	ENSG00000130311
1567	1210	1326	1262	-0,23806023	7,133344679	0,00163313	0,013888757	down	TOR1AIP2	ENSG00000169905
4679	3707	5605	5274	0,238084765	8,967214844	0,000385764	0,00423958	up	CTNNAL1	ENSG00000119326
459	346	526	518	0,238089958	5,594151123	0,008022276	0,049220833	up	GMNN	ENSG00000112312
2302	1985	2104	1909	-0,238327379	7,76515446	0,000809856	0,007845446	down	LAMC1	ENSG00000135862
563	458	478	475	-0,238354662	5,694972933	0,006199819	0,040015376	down	TBC1D2B	ENSG00000167202
1874	1557	2281	2179	0,238418715	7,682417541	0,000646612	0,006516055	up	AAK1	ENSG00000115977
712	558	845	804	0,238688433	6,250129418	0,002367414	0,018605049	up	AC092881.1	ENSG00000111596
620	495	564	477	-0,239072175	5,82045972	0,007020153	0,044341091	down	ALG3	ENSG00000214160
825	770	1034	1047	0,239428608	6,585641483	0,005415518	0,035786202	up	SETD3	ENSG00000183576
577	475	713	656	0,239782296	5,981945359	0,003370868	0,024544704	up	SHOC2	ENSG00000108061
1047	824	944	801	-0,239929808	6,564476407	0,003112085	0,02301313	down	IPO13	ENSG00000117408
1159	905	981	941	-0,240036054	6,705592549	0,001526042	0,013136315	down	MADD	ENSG00000110514
1132	991	1000	983	-0,24039116	6,751344976	0,002170791	0,017349567	down	TEX2	ENSG00000136478
603	503	710	729	0,240535368	6,054808323	0,004653497	0,031848462	up	DERL2	ENSG00000072849
2004	1408	2244	2168	0,240608158	7,666193645	0,004450826	0,030745941	up	PANK3	ENSG00000120137
760	661	657	669	-0,240751986	6,172188178	0,003909184	0,027719503	down	ERLIN2	ENSG00000147475
2208	2190	2098	2033	-0,240848026	7,809190394	0,007150472	0,04504652	down	SPATS2L	ENSG00000196141
868	752	736	775	-0,240855542	6,360875962	0,004149644	0,029057817	down	KIAA1109	ENSG00000138688
1292	958	1461	1456	0,241000572	7,070598673	0,002820479	0,021355059	up	NUP210	ENSG00000132182
1479	1219	1754	1757	0,241276893	7,337447935	0,001171331	0,010508141	up	TARDBP	ENSG00000120948
1005	754	1162	1122	0,241377987	6,717664561	0,001986986	0,016248504	up	FANCI	ENSG00000140525
1020	975	1022	850	-0,241474742	6,66567178	0,008108946	0,049623159	down	PPIG	ENSG00000138398
8123	6710	7171	6657	-0,241475903	9,551825713	0,000188205	0,002307181	down	NUCKS1	ENSG00000069275
577	545	779	689	0,24164965	6,08092907	0,007670537	0,047528595	up	IWS1	ENSG00000163166
555	404	646	600	0,241733138	5,845791333	0,006818548	0,043324562	up	EPHB4	ENSG00000196411
1160	892	1374	1294	0,242015865	6,940613408	0,00153265	0,013172083	up	KANSL3	ENSG00000114982
955	764	1144	1094	0,242164197	6,687942804	0,001136893	0,010252505	up	ARPC4	ENSG00000241553
741	639	692	596	-0,242263461	6,128814425	0,00353974	0,025543039	down	CHML	ENSG00000203668
2898	2362	3523	3329	0,242341407	8,299042466	0,00033837	0,003791156	up	KARS	ENSG00000065427
1313	1028	1641	1409	0,242659965	7,131308536	0,001970373	0,016146663	up	MED15	ENSG00000099917
2158	1786	2582	2557	0,242765262	7,885301075	0,000651544	0,006548759	up	SMG1	ENSG00000157106

434	383	561	506	0,242818397	5,622747438	0,007222116	0,045424156	up	MTBP	ENSG00000172167
1171	803	1357	1202	0,242911018	6,879459633	0,007926846	0,048793747	up	TBC1D4	ENSG00000136111
1255	878	1032	943	-0,242976161	6,746018102	0,006775595	0,043157593	down	USP19	ENSG00000172046
1371	1002	1597	1485	0,2431001	7,147579386	0,002353807	0,018516854	up	TMX1	ENSG00000139921
496	406	454	386	-0,243390697	5,514441425	0,007554701	0,0469774	down	ARID5B	ENSG00000150347
1410	1190	1728	1665	0,243444613	7,286558611	0,000800497	0,007788791	up	GPD2	ENSG00000115159
1133	1036	1508	1334	0,243748494	7,029800696	0,002626275	0,020219847	up	HEXIM1	ENSG00000186834
712	542	566	597	-0,243778433	5,985444534	0,007800555	0,048164936	down	AP1M2	ENSG00000129354
1231	985	1475	1413	0,243889546	7,054156653	0,000967091	0,009036075	up	HMGXB3	ENSG00000113716
575	447	688	644	0,243952617	5,940757692	0,003282399	0,024043571	up	MRGBP	ENSG00000101189
1110	850	1406	1150	0,244223721	6,875554852	0,004835561	0,032773991	up	GLS	ENSG00000115419
1106	881	1384	1209	0,244411656	6,897266294	0,001642037	0,013949228	up	SLC7A6	ENSG00000103064
1003	874	885	863	-0,244452672	6,571624642	0,001569315	0,013449171	down	ASB1	ENSG00000065802
994	806	1194	1154	0,244653711	6,75609201	0,001334096	0,011724491	up	ZMYND11	ENSG00000015171
479	380	403	395	-0,244765661	5,442847481	0,007422712	0,046292089	down	BPNT1	ENSG00000162813
668	565	797	813	0,245149389	6,214098816	0,002979161	0,022288918	up	SMARCAD1	ENSG00000163104
384	312	476	433	0,245176282	5,39163715	0,006538741	0,041906769	up	SRSF8	ENSG00000263465
1178	1006	1416	1437	0,245269352	7,037122825	0,001621931	0,0138238	up	NFAT5	ENSG00000102908
576	505	515	491	-0,24545206	5,776294243	0,004341788	0,030180672	down	CD276	ENSG00000103855
938	770	813	774	-0,245556276	6,432524707	0,00132454	0,011660305	down	ZNF562	ENSG00000171466
543	469	662	661	0,245893879	5,931354813	0,003950574	0,027949189	up	CLPX	ENSG00000166855
1748	1540	1552	1509	-0,245991975	7,380060341	0,000957619	0,009000401	down	CDCA7L	ENSG00000164649
489	415	423	417	-0,246178069	5,5176114	0,005809918	0,037972316	down	PTPN3	ENSG00000070159
1618	1293	1970	1831	0,246304296	7,447997885	0,000554038	0,005716716	up	LRRC58	ENSG00000163428
1513	1264	1820	1809	0,246508875	7,382560947	0,000802435	0,007793008	up	SLC25A37	ENSG00000147454
1019	739	1162	1126	0,246534011	6,718002423	0,002530865	0,019650904	up	MIB1	ENSG00000101752
2433	2237	2257	2100	-0,246538138	7,888580641	0,001271301	0,011261855	down	MAP7D3	ENSG00000129680
1643	1288	2000	1826	0,246544917	7,456972684	0,000680616	0,00679262	up	EHD4	ENSG00000103966
946	826	842	806	-0,246672449	6,487660031	0,001438921	0,012532253	down	COA1	ENSG00000106603
1890	1811	2461	2404	0,246694901	7,804237033	0,003241092	0,023797128	up	DHFR	ENSG00000228716
2993	2454	2608	2448	-0,24704395	8,103699262	0,000282938	0,0032643	down	ADAM9	ENSG00000168615
1077	850	1331	1187	0,247047164	6,854191395	0,001326601	0,011671835	up	SHCBP1	ENSG00000171241

1066	868	929	866	-0,247090073	6,610460563	0,000954946	0,008992942	down	CDC73	ENSG00000134371
488	439	635	580	0,247268053	5,807376615	0,005097058	0,034073274	up	MNAT1	ENSG00000020426
626	476	527	494	-0,247342284	5,798111306	0,004919906	0,033217925	down	TMEM115	ENSG00000126062
423	359	365	361	-0,247382973	5,308636337	0,007985213	0,049051574	down	PRXL2A	ENSG00000122378
1812	1496	2259	2071	0,247955192	7,634690947	0,000398816	0,004358347	up	NAA15	ENSG00000164134
1411	1099	1708	1571	0,248019249	7,234311892	0,000801235	0,007791097	up	CHTF8	ENSG00000168802
758	695	962	945	0,248630754	6,455524581	0,002752115	0,020954576	up	CCDC14	ENSG00000175455
3288	2930	4334	3840	0,248730358	8,549050795	0,00070213	0,006967012	up	DEK	ENSG00000124795
488	407	423	407	-0,24876396	5,501447675	0,004879004	0,032956089	down	DOK1	ENSG00000115325
1021	869	1302	1176	0,248798687	6,83073094	0,001121956	0,010176901	up	TAF2	ENSG00000064313
1125	972	1403	1346	0,248863376	6,981100111	0,000972094	0,009053966	up	BTAF1	ENSG00000095564
565	474	475	488	-0,249070257	5,716142788	0,005059253	0,033835118	down	BICC1	ENSG00000122870
1435	1142	1716	1656	0,250190731	7,274505738	0,000610132	0,006217022	up	PPP2R5C	ENSG00000078304
653	483	769	716	0,250242632	6,094073875	0,00308518	0,022879545	up	CENPU	ENSG00000151725
660	499	543	528	-0,250349956	5,868995099	0,004434339	0,030659331	down	ANO10	ENSG00000160746
1181	1013	1420	1457	0,250744319	7,046920301	0,001493764	0,012922929	up	PPIP5K2	ENSG00000145725
765	564	635	592	-0,250824693	6,064495187	0,004044417	0,028522138	down	CRELD2	ENSG00000184164
1200	1202	1140	1100	-0,250870287	6,931713716	0,007070282	0,044613736	down	H3F3A	ENSG00000163041
603	508	524	505	-0,250922562	5,811763438	0,002948957	0,022112191	down	IVNS1ABP	ENSG00000116679
1258	956	1551	1347	0,251025973	7,0541734	0,001619217	0,013815833	up	CTBP1	ENSG00000159692
1238	1119	1635	1468	0,251390326	7,153108487	0,001329899	0,011694221	up	RPRD2	ENSG00000163125
607	475	715	702	0,251410914	6,026997428	0,002381662	0,018679207	up	BAG5	ENSG00000166170
825	760	775	697	-0,251938697	6,326777757	0,00245017	0,019110341	down	CRTC2	ENSG00000160741
738	541	820	851	0,252200744	6,264664558	0,00440707	0,030552405	up	CMTR1	ENSG00000137200
495	394	575	590	0,252251238	5,746004444	0,004775954	0,032457676	up	CNOT8	ENSG00000155508
659	466	748	722	0,252291391	6,07926265	0,005034351	0,033706889	up	LPGAT1	ENSG00000123684
610	517	546	497	-0,252851217	5,831572577	0,002575389	0,019916941	down	LRCH1	ENSG00000136141
482	369	582	534	0,252880262	5,682461628	0,00393666	0,02786636	up	MUS81	ENSG00000172732
1230	1068	1040	1086	-0,252916719	6,859406001	0,00200409	0,016353919	down	TRIM14	ENSG00000106785
945	677	1123	999	0,253113805	6,605346639	0,002930803	0,022007952	up	MAEA	ENSG00000090316
1080	739	1275	1101	0,253379305	6,767623585	0,006949863	0,043996913	up	POLD1	ENSG00000062822
1429	1163	1757	1646	0,253384791	7,285733683	0,000417972	0,004526277	up	PPP4R1	ENSG00000154845

2030	1546	2452	2233	0,253487834	7,745480629	0,000595519	0,006092243	up	MTCL1	ENSG00000168502
813	672	986	964	0,253532532	6,485157214	0,000964517	0,009028325	up	ST13	ENSG00000100380
1671	1367	1468	1340	-0,25356553	7,258567188	0,000448239	0,004813769	down	STAT6	ENSG00000166888
831	679	1054	931	0,254048118	6,508903335	0,001067269	0,009743454	up	LATS2	ENSG00000150457
2695	2079	2265	2134	-0,254051132	7,906776987	0,000430438	0,004641855	down	ATIC	ENSG00000138363
1634	1417	1436	1387	-0,254053423	7,267559253	0,000570867	0,00587086	down	MXRA7	ENSG00000182534
882	759	1091	1067	0,254057285	6,63082277	0,000915586	0,008683562	up	EXOC6B	ENSG00000144036
928	854	875	778	-0,254107473	6,494739711	0,002118556	0,017064673	down	ROBO1	ENSG00000169855
2325	2027	2033	1994	-0,254173989	7,780205813	0,000471413	0,005017524	down	QSER1	ENSG00000060749
4113	3177	4864	4686	0,254213966	8,772564945	0,00029434	0,003370804	up	G3BP1	ENSG00000145907
549	470	681	659	0,254294259	5,945542039	0,002203044	0,017575115	up	CDC7	ENSG00000097046
1794	1450	1603	1394	-0,254474523	7,352083111	0,000653432	0,006554223	down	AC005840.1	ENSG00000111321
459	339	550	497	0,254486619	5,589958393	0,005368131	0,03553355	up	MIGA1	ENSG00000180488
934	869	890	783	-0,254501395	6,51202286	0,002586702	0,019984526	down	EPS15L1	ENSG00000127527
521	419	607	627	0,254636599	5,827714553	0,004101266	0,028818353	up	ACTR10	ENSG00000131966
1200	1030	1482	1452	0,254741337	7,072558751	0,000707438	0,007011676	up	PHF20L1	ENSG00000129292
1139	1033	1097	918	-0,254943702	6,779312664	0,002804013	0,021261481	down	AKT1S1	ENSG00000204673
526	443	634	640	0,255026125	5,873154616	0,003102778	0,02297713	up	CASP8AP2	ENSG00000118412
1261	943	1471	1417	0,255174087	7,049167288	0,001124211	0,010185455	up	TMEM33	ENSG00000109133
1468	1121	1798	1600	0,25521266	7,281794666	0,000843329	0,008119067	up	GPS1	ENSG00000169727
576	488	487	495	-0,255624478	5,74762449	0,003666171	0,02627225	down	NECAP1	ENSG00000089818
462	377	392	382	-0,255656424	5,404730488	0,004962568	0,033332057	down	ARHGAP31	ENSG00000031081
621	556	750	800	0,255811237	6,155445039	0,005023128	0,033651488	up	DNAJC21	ENSG00000168724
7529	6721	6636	6548	-0,256122679	9,492305716	0,000375668	0,004149683	down	MAP4	ENSG00000047849
1501	1136	1261	1165	-0,256186698	7,049374833	0,000978561	0,009099441	down	SOAT1	ENSG00000057252
1325	1090	1682	1498	0,256222778	7,185998033	0,000531823	0,005527896	up	SNX5	ENSG00000089006
1131	908	959	920	-0,256347594	6,681734715	0,000595533	0,006092243	down	GBE1	ENSG00000114480
1241	958	1532	1358	0,256380353	7,048142195	0,000882559	0,008408133	up	RRP7A	ENSG00000189306
951	798	1196	1108	0,256727441	6,722864266	0,000529162	0,005509337	up	PGM2	ENSG00000169299
757	611	926	874	0,256745995	6,368000082	0,000724469	0,007147577	up	TBC1D1	ENSG00000065882
581	486	501	483	-0,256902508	5,750580608	0,002515493	0,019560889	down	MECOM	ENSG00000085276
1891	1476	2296	2129	0,256924124	7,662046655	0,000325976	0,003676164	up	ALKBH5	ENSG00000091542

3458	2921	4159	4238	0,256941208	8,58711359	0,000402124	0,004388324	up	SCRN1	ENSG00000136193
541	465	687	639	0,257085278	5,928722977	0,001964771	0,016109251	up	MCTS1	ENSG00000232119
891	784	1194	1019	0,257446855	6,663244322	0,001532742	0,013172083	up	BASP1	ENSG00000176788
671	553	549	578	-0,257559784	5,947348541	0,00339321	0,024692198	down	PCID2	ENSG00000126226
777	667	946	957	0,257775123	6,448805264	0,001342965	0,011795764	up	TRMT112	ENSG00000173113
922	862	1229	1133	0,258784884	6,757637908	0,001896793	0,015651066	up	CCDC80	ENSG00000091986
833	764	780	696	-0,258831356	6,334253588	0,00180659	0,015058793	down	TAOK3	ENSG00000135090
576	480	516	457	-0,258919862	5,734489827	0,002527486	0,01963448	down	WASF3	ENSG00000132970
1065	919	1259	1357	0,259288962	6,907062256	0,002565487	0,019865365	up	PEG10	ENSG00000242265
1300	970	1567	1418	0,259301432	7,093744462	0,000959216	0,009000401	up	PHC2	ENSG00000134686
1564	1491	2144	1911	0,259443466	7,534387394	0,001947353	0,015983324	up	TCERG1	ENSG00000113649
15997	13079	14221	12557	-0,259515025	10,51388105	8,55E-05	0,001213675	down	ANXA2	ENSG00000182718
1873	1485	2277	2146	0,259607477	7,660517612	0,0002339	0,002774817	up	LMNB1	ENSG00000113368
1512	1216	1754	1836	0,259617358	7,362084534	0,00104001	0,00955075	up	TNS3	ENSG00000136205
851	687	710	704	-0,259686282	6,274323213	0,001003287	0,009290423	down	AFF1	ENSG00000172493
1213	1067	1093	1010	-0,259804482	6,845414631	0,000724368	0,007147577	down	CTR9	ENSG00000198730
329	279	429	374	0,259939517	5,207118827	0,007925359	0,048793747	up	PSMC3IP	ENSG00000131470
1138	875	952	896	-0,260172847	6,659482179	0,00074229	0,007286355	down	TMEM127	ENSG00000135956
2338	1869	1976	1889	-0,260183942	7,723697864	0,000221904	0,002646658	down	DHX33	ENSG00000005100
707	582	873	828	0,260323139	6,285234528	0,000707866	0,007011676	up	URI1	ENSG00000105176
470	378	416	364	-0,260438127	5,417240665	0,004396648	0,03049377	down	VAMP8	ENSG00000118640
607	472	511	480	-0,260770871	5,762352062	0,002611724	0,020138854	down	ATP6V1H	ENSG00000047249
2243	1729	2768	2466	0,260812267	7,901360072	0,000388297	0,004264407	up	MAZ	ENSG00000103495
3076	2403	2815	2223	-0,261275729	8,102740194	0,002786456	0,021159356	down	SLC7A11	ENSG00000151012
1450	1215	1955	1574	0,261278775	7,331652948	0,002096867	0,01693305	up	SYDE1	ENSG00000105137
1830	1751	2427	2329	0,261352595	7,764645645	0,001611968	0,013761544	up	KCMF1	ENSG00000176407
1658	1294	1372	1335	-0,2614669	7,21112891	0,000492241	0,005214196	down	S100A16	ENSG00000188643
996	760	1208	1107	0,26163318	6,727119925	0,000647222	0,006517975	up	CYB5B	ENSG00000103018
1559	1174	1228	1272	-0,261853853	7,097922234	0,001621659	0,0138238	down	TSC22D1	ENSG00000102804
496	402	607	579	0,26193815	5,766441279	0,002023741	0,016479638	up	BMI1	ENSG00000168283
784	595	672	593	-0,261969824	6,113566797	0,00205628	0,016683369	down	GRHPR	ENSG00000137106
912	829	881	726	-0,262147704	6,457023134	0,00284817	0,021512275	down	PYCR2	ENSG00000143811

1135	1031	996	999	-0,262201911	6,771980515	0,001421187	0,012394119	down	GPSM2	ENSG00000121957
1411	1100	1648	1661	0,262322902	7,242731632	0,000652225	0,006551361	up	NUP93	ENSG00000102900
2288	1863	1918	1890	-0,262722233	7,704238142	0,000231532	0,002750931	down	CREB3L2	ENSG00000182158
658	565	573	551	-0,26277569	5,945188585	0,0014443	0,012565008	down	STAC	ENSG00000144681
1392	984	1677	1451	0,263360208	7,158639427	0,002658459	0,020395827	up	RECQL4	ENSG00000160957
963	729	1187	1047	0,263577474	6,674423395	0,000893277	0,008492067	up	TEAD4	ENSG00000197905
738	534	873	804	0,263596838	6,262758203	0,001868414	0,015449734	up	RFC5	ENSG00000111445
475	332	563	501	0,263702736	5,609014737	0,007145424	0,04503299	up	MZT1	ENSG00000204899
493	390	623	545	0,263906614	5,742486943	0,00265964	0,020395827	up	NOSIP	ENSG00000142546
985	676	1141	1044	0,263934194	6,643244406	0,003248552	0,023840627	up	CBFB	ENSG00000067955
927	705	1114	1041	0,264309642	6,623222518	0,000653524	0,006554223	up	ATP6V1C1	ENSG00000155097
2150	1725	2681	2444	0,2643228	7,86994408	0,000158474	0,001999135	up	PRELID1	ENSG00000169230
353	262	431	382	0,264610155	5,222626301	0,007585402	0,047082171	up	CLPTM1L	ENSG00000049656
1712	1466	1517	1402	-0,264764441	7,320757259	0,000250008	0,002941197	down	MYO1B	ENSG00000128641
1895	1501	1673	1440	-0,264853699	7,412237811	0,00049826	0,005270758	down	MAST2	ENSG00000086015
2209	1710	2816	2369	0,265279503	7,884678741	0,000773595	0,007555446	up	ADRM1	ENSG00000130706
654	561	548	566	-0,265387708	5,934590728	0,002249694	0,017860682	down	PERP	ENSG00000112378
762	665	954	938	0,265452197	6,436664169	0,000829617	0,008006915	up	BRI3BP	ENSG00000184992
1108	918	926	930	-0,265553164	6,669712108	0,000526211	0,005493818	down	CTSB	ENSG00000164733
2456	1975	3011	2852	0,26565253	8,063926212	0,000113976	0,001519257	up	PRNP	ENSG00000171867
676	521	840	744	0,265722532	6,179269511	0,001221369	0,010862912	up	FBXO9	ENSG00000112146
717	678	967	889	0,265860928	6,407795461	0,001993145	0,016281718	up	POLD3	ENSG00000077514
1207	882	1432	1325	0,265912946	6,97686404	0,000999109	0,009257262	up	PCNA	ENSG00000132646
2176	1962	1880	1921	-0,265949274	7,703991855	0,000764987	0,007480172	down	CKAP4	ENSG00000136026
825	723	713	707	-0,266098874	6,28402439	0,000891697	0,008484779	down	GNAQ	ENSG00000156052
507	391	441	381	-0,266280244	5,495505358	0,004150656	0,029057817	down	NDUFA4	ENSG00000189043
4608	3976	4012	3864	-0,266346934	8,754090615	0,00010045	0,001368329	down	CHD3	ENSG00000170004
353	309	442	436	0,266411009	5,333667578	0,004877031	0,032956089	up	PANX1	ENSG00000110218
347	281	293	282	-0,266535106	4,983532441	0,008147941	0,049775523	down	SLC35A3	ENSG00000117620
759	534	888	819	0,267023794	6,28685087	0,002345381	0,018478651	up	ARMC1	ENSG00000104442
432	329	528	480	0,267182539	5,529984073	0,002946764	0,022106421	up	NAA30	ENSG00000139977
385	357	355	326	-0,26721651	5,226485483	0,006982812	0,044169485	down	ERG28	ENSG00000133935

1105	888	1387	1255	0,267247118	6,914590919	0,000384675	0,004230607	up	EVPL	ENSG00000167880
776	614	686	586	-0,267509041	6,124059028	0,001463777	0,012705978	down	C5orf24	ENSG00000181904
1777	1232	1480	1259	-0,26753288	7,22937209	0,004116271	0,028896405	down	CD81	ENSG00000110651
492	397	406	407	-0,267624463	5,482029262	0,003171905	0,023366884	down	SNX30	ENSG00000148158
339	296	425	418	0,267853657	5,274668991	0,004840179	0,032779387	up	PLPBP	ENSG00000147471
1023	761	1183	1175	0,268174959	6,752144663	0,001066994	0,009743454	up	CD164	ENSG00000135535
1075	915	1292	1348	0,268188826	6,915656462	0,000969839	0,009041396	up	TMEM230	ENSG00000089063
594	527	731	758	0,268273028	6,09182654	0,002320553	0,018329533	up	AGO1	ENSG00000092847
3005	2029	3539	3092	0,268397338	8,238639502	0,004109385	0,028862343	up	SNRPB	ENSG00000125835
2484	2046	2074	2066	-0,268577112	7,828207418	0,00018083	0,002228654	down	ILK	ENSG00000166333
1089	976	1448	1302	0,268647278	6,97164846	0,000623617	0,006337797	up	ELOA	ENSG00000011007
1924	1522	2425	2146	0,268712723	7,702614817	0,000219755	0,002623041	up	H2AFZ	ENSG00000164032
1671	1318	1401	1328	-0,268777886	7,226264361	0,000256524	0,003008734	down	IARS2	ENSG00000067704
334	311	435	423	0,268838483	5,29949116	0,006535191	0,041901311	up	RFC4	ENSG00000163918
1423	1233	1820	1712	0,268878913	7,332542046	0,000229725	0,002735738	up	PSMF1	ENSG00000125818
1436	1036	1660	1605	0,268947522	7,219791478	0,001013518	0,009346157	up	CNP	ENSG00000173786
498	428	420	427	-0,268993312	5,541996105	0,002872066	0,02168154	down	XYLT2	ENSG00000015532
871	677	1080	973	0,269059127	6,550836494	0,0005468	0,00565707	up	PTAR1	ENSG00000188647
353	317	459	432	0,269219735	5,353104743	0,004370221	0,030346084	up	RPRD1A	ENSG00000141425
527	428	656	612	0,269314894	5,858911859	0,001217736	0,010836805	up	KCTD3	ENSG00000136636
575	458	466	477	-0,2693445	5,696594896	0,002674327	0,020496215	down	HDAC6	ENSG00000094631
843	710	1106	960	0,269722759	6,559217812	0,000632621	0,006417814	up	SLBP	ENSG00000163950
739	584	632	576	-0,269950747	6,051878541	0,000955888	0,008996352	down	C11orf68	ENSG00000175573
569	432	450	462	-0,270388288	5,649149795	0,003700095	0,026466499	down	IGSF3	ENSG00000143061
488	433	410	432	-0,270445663	5,53464616	0,004476366	0,030908624	down	GPAT3	ENSG00000138678
1072	903	925	880	-0,270474925	6,631387919	0,000293003	0,003357969	down	TBC1D16	ENSG00000167291
484	419	650	553	0,270994915	5,781579767	0,002781384	0,021131173	up	UTP6	ENSG00000108651
334	265	405	391	0,271163437	5,190442859	0,004540004	0,03126462	up	DEPDC1B	ENSG00000035499
487	445	597	644	0,271226497	5,829218034	0,005789545	0,037855093	up	TIMP3	ENSG00000100234
9923	9864	14006	12559	0,271429005	10,23822664	0,002148245	0,017258201	up	PTMA	ENSG00000187514
611	542	538	516	-0,271671574	5,857384137	0,001522566	0,013128226	down	GOSR1	ENSG00000108587
1012	769	1194	1168	0,271674906	6,752719648	0,000628975	0,006388066	up	NOB1	ENSG00000141101

813	797	1146	1010	0,271901014	6,619573887	0,002811465	0,021297194	up	SMC5	ENSG00000198887
1237	1000	1121	922	-0,272011985	6,808262842	0,00107003	0,009762922	down	PELP1	ENSG00000141456
3327	2591	4111	3748	0,272071482	8,482425446	9,91E-05	0,001356292	up	TYMS	ENSG00000176890
3208	2751	2698	2742	-0,272084107	8,224478022	0,000209714	0,002528456	down	DDAH1	ENSG00000153904
601	467	743	677	0,272476659	6,019665109	0,00095861	0,009000401	up	GRK6	ENSG00000198055
1038	816	1226	1236	0,272599134	6,81253178	0,000564455	0,005816467	up	WDHD1	ENSG00000198554
399	338	483	498	0,272849718	5,490349923	0,003631962	0,026087298	up	MBD2	ENSG00000134046
5551	4383	4724	4321	-0,272996853	8,956233256	5,68E-05	0,00084799	down	ARF1	ENSG00000143761
1909	1331	1495	1439	-0,273001542	7,333715467	0,001938247	0,015916992	down	ITGA5	ENSG00000161638
702	530	556	564	-0,273077646	5,946157385	0,002140398	0,01720406	down	GTPBP1	ENSG00000100226
312	266	395	375	0,273146479	5,142120072	0,004641989	0,031783707	up	ZNF702P	ENSG00000242779
963	820	1234	1143	0,273285008	6,760376549	0,00034034	0,003807736	up	RTF2	ENSG00000022277
1086	898	960	850	-0,273293133	6,635747319	0,000307664	0,003507866	down	QSOX2	ENSG00000165661
1898	1789	1724	1654	-0,273397719	7,536537462	0,000746509	0,00731568	down	MYL12A	ENSG00000101608
322	276	396	401	0,274196121	5,191686496	0,005354893	0,035461039	up	HOOK3	ENSG00000168172
1102	916	1369	1320	0,274276306	6,93800057	0,000241977	0,002857537	up	RNF167	ENSG00000108523
1459	1199	1703	1832	0,274288671	7,333837313	0,000933973	0,008838314	up	TNFRSF21	ENSG00000146072
601	461	500	466	-0,274354676	5,732732237	0,001753303	0,014693491	down	ASH2L	ENSG00000129691
312	263	272	252	-0,274375305	4,854296404	0,008128057	0,049712723	down	CAMK2D	ENSG00000145349
596	496	514	481	-0,274420261	5,775554139	0,001143432	0,010293537	down	TMEM14B	ENSG00000137210
623	455	506	473	-0,274557461	5,752254489	0,002990625	0,022306157	down	SLC35E2B	ENSG00000189339
4342	3671	3683	3618	-0,274726219	8,649701707	6,52E-05	0,000957253	down	CRTAP	ENSG00000170275
406	294	455	475	0,275293249	5,412823523	0,00668225	0,042703154	up	DDHD2	ENSG00000085788
2199	2233	2103	1968	-0,275382221	7,805881816	0,003269154	0,023958632	down	PLP2	ENSG00000102007
726	569	914	812	0,275514475	6,298373542	0,000513133	0,005393334	up	DSN1	ENSG00000149636
2917	2273	3610	3299	0,275528884	8,295267012	8,70E-05	0,001231812	up	MCM3	ENSG00000112118
1493	1134	1249	1136	-0,275738192	7,035074822	0,000398779	0,004358347	down	PPP1R15B	ENSG00000158615
784	697	1030	950	0,275741687	6,496533977	0,000461797	0,004925309	up	NBN	ENSG00000104320
474	391	595	559	0,275830776	5,720948455	0,001164635	0,010460162	up	PLIN2	ENSG00000147872
713	642	639	597	-0,275909224	6,088603539	0,000937058	0,008862106	down	LAYN	ENSG00000204381
699	608	897	849	0,276049657	6,315974374	0,000413708	0,004492638	up	FKTN	ENSG00000106692
1350	1150	1190	1088	-0,276050245	6,969201103	0,00021353	0,002564503	down	ENAH	ENSG00000154380

785	745	1019	1030	0,276281275	6,546860397	0,001733027	0,014547119	up	BRCA1	ENSG00000012048
869	801	824	702	-0,276523483	6,390865904	0,001271176	0,011261855	down	PFDN2	ENSG00000143256
925	688	1144	1004	0,276623073	6,612110807	0,000724238	0,007147577	up	POLR3H	ENSG00000100413
490	393	425	378	-0,276637733	5,467769992	0,002117327	0,017064673	down	AMBRA1	ENSG00000110497
348	297	425	436	0,276706511	5,301454425	0,004200605	0,029351402	up	LMBRD2	ENSG00000164187
305	275	381	394	0,276808874	5,150720298	0,007585781	0,047082171	up	MORC3	ENSG00000159256
2071	1795	2694	2478	0,276825363	7,877764209	9,41E-05	0,001311209	up	RRM2	ENSG00000171848
6451	4917	5184	5110	-0,276960602	9,146675022	0,000136175	0,001760745	down	COL4A2	ENSG00000134871
3269	2616	2702	2640	-0,276999357	8,200108506	7,21E-05	0,001049796	down	COL4A1	ENSG00000187498
364	331	323	310	-0,277036786	5,127236494	0,005548356	0,036524126	down	ISOC1	ENSG00000066583
1245	1031	1562	1477	0,277068004	7,112502968	0,00014482	0,001854578	up	UGP2	ENSG00000169764
706	526	877	765	0,277230561	6,225628971	0,001094282	0,009966626	up	WTAP	ENSG00000146457
477	378	377	399	-0,277274455	5,420885769	0,004131135	0,02897579	down	JARID2	ENSG00000008083
523	467	671	653	0,277311275	5,918304769	0,001258902	0,011177522	up	RSPRY1	ENSG00000159579
1323	1127	1091	1136	-0,277456169	6,939507439	0,000552232	0,005705666	down	VANGL1	ENSG00000173218
710	595	620	567	-0,277478349	6,030868753	0,000574223	0,005901459	down	TOLLIP	ENSG00000078902
1392	1084	1732	1570	0,27752065	7,230902457	0,000194745	0,002370054	up	SLC35E1	ENSG00000127526
589	535	490	533	-0,27769513	5,81903738	0,004433719	0,030659331	down	LHFPL6	ENSG00000183722
474	375	553	579	0,277711512	5,693817375	0,002658935	0,020395827	up	SLC5A3	ENSG00000198743
1663	1411	1470	1328	-0,277745492	7,266317221	0,000137262	0,001773325	down	FAM129A	ENSG00000135842
670	575	570	562	-0,277999604	5,963838123	0,000871951	0,008332633	down	MANBAL	ENSG00000101363
665	551	842	783	0,278010061	6,211463443	0,000372346	0,004118349	up	CTDSPL2	ENSG00000137770
326	260	418	365	0,278093249	5,16287812	0,004714534	0,032166961	up	GALNT14	ENSG00000158089
2895	2142	2385	2173	-0,278274602	7,970529164	0,00031221	0,003544089	down	CPNE1	ENSG00000214078
1241	953	1010	978	-0,278303037	6,775029009	0,000419882	0,004542527	down	HEXB	ENSG00000049860
691	581	591	565	-0,278316256	5,993785839	0,000633144	0,006417814	down	MON1B	ENSG00000103111
660	546	835	777	0,278394963	6,199754482	0,000376606	0,004153633	up	LARS2	ENSG00000011376
508	400	420	404	-0,278475599	5,506606229	0,001828525	0,015184584	down	SLC39A8	ENSG00000138821
3508	2750	2811	2855	-0,278583764	8,286764269	0,00017217	0,002137189	down	FADS2	ENSG00000134824
896	760	1163	1053	0,278595059	6,656791022	0,000217479	0,002599874	up	SUZ12	ENSG00000178691
4814	3772	5890	5561	0,278778957	9,022729228	3,97E-05	0,000622842	up	NSD2	ENSG00000109685
379	283	303	297	-0,278836051	5,05125955	0,006890808	0,043712157	down	C1orf52	ENSG00000162642

591	428	725	634	0,279096786	5,953178793	0,002094439	0,016922241	up	REST	ENSG00000084093
1891	1388	2268	2098	0,279170547	7,632736555	0,00032645	0,003676434	up	FAM91A1	ENSG00000176853
392	331	354	303	-0,279352972	5,180718101	0,004430415	0,030659331	down	CLCN4	ENSG00000073464
1927	1395	2213	2204	0,279579453	7,650825061	0,000623275	0,006337797	up	ZMPSTE24	ENSG00000084073
490	408	660	543	0,279716495	5,777192733	0,002713206	0,020739777	up	PTPN2	ENSG00000175354
494	425	442	393	-0,279868227	5,525869779	0,001692518	0,014319174	down	PBDC1	ENSG00000102390
5063	3956	6284	5758	0,279935486	9,094098395	4,17E-05	0,000649457	up	MCM4	ENSG00000104738
518	402	434	400	-0,279955976	5,524266464	0,001784597	0,014893152	down	SPAG7	ENSG00000091640
531	417	623	643	0,280168817	5,853195526	0,001637811	0,013920948	up	DHTKD1	ENSG00000181192
310	241	390	347	0,280268074	5,075325459	0,004942099	0,033259326	up	ZKSCAN5	ENSG00000196652
1708	1374	2279	1850	0,28042829	7,549280052	0,000727736	0,007170689	up	DOT1L	ENSG00000104885
533	465	452	454	-0,280502264	5,644892294	0,001627522	0,013856242	down	RNF111	ENSG00000157450
458	340	361	361	-0,280515485	5,318272489	0,004969504	0,033364213	down	PIGG	ENSG00000174227
1780	1468	2236	2111	0,280627502	7,62623771	5,86E-05	0,000871314	up	NUTF2	ENSG00000102898
498	401	625	578	0,280693663	5,778221814	0,00092159	0,008731771	up	NCOA2	ENSG00000140396
302	245	369	363	0,280915304	5,066379341	0,004279545	0,029801373	up	GPN2	ENSG00000142751
380	343	514	456	0,28091946	5,469096129	0,002988718	0,022306157	up	BID	ENSG00000015475
516	453	467	413	-0,281412006	5,6020835	0,001626906	0,013856242	down	AIP	ENSG00000110711
767	566	912	868	0,281680635	6,340894432	0,00057492	0,005904722	up	GLRX3	ENSG00000108010
438	323	347	341	-0,281689482	5,249401357	0,005018543	0,033635284	down	MTX2	ENSG00000128654
3042	2835	2714	2637	-0,281930826	8,205016184	0,000330026	0,003708372	down	ARHGDIB	ENSG00000111348
258	221	327	315	0,281953074	4,878097864	0,006803028	0,043279073	up	RIC8B	ENSG00000111785
400	360	530	490	0,282012614	5,541234015	0,001933763	0,015896984	up	TOMM5	ENSG00000175768
1718	1441	2216	2021	0,282211394	7,587986746	6,50E-05	0,000956152	up	RARS	ENSG00000113643
316	256	264	254	-0,282427332	4,842212304	0,006087406	0,039420903	down	GMIP	ENSG00000089639
596	402	713	618	0,282465887	5,922062742	0,005040035	0,033721113	up	TIMM13	ENSG00000099800
1286	1095	1655	1540	0,282623869	7,181851612	0,000110521	0,001478281	up	ODC1	ENSG00000115758
276	227	337	337	0,282645355	4,947617286	0,005953376	0,038730616	up	TTC7A	ENSG00000068724
1226	1180	1120	1071	-0,282803548	6,917157252	0,001066346	0,009743454	down	GDI1	ENSG00000203879
561	557	768	739	0,283392862	6,101162907	0,003076733	0,022838715	up	UBLCP1	ENSG00000164332
1606	1384	1476	1239	-0,283477765	7,224595764	0,000357036	0,003963121	down	KRT7	ENSG00000135480
526	418	647	618	0,283518474	5,849496202	0,000736916	0,00724277	up	ARHGAP18	ENSG00000146376

919	722	750	733	-0,283529034	6,355519829	0,000306227	0,003496618	down	PDHX	ENSG00000110435
1158	928	969	917	-0,283972649	6,701749057	0,000127786	0,001671762	down	ATP11A	ENSG00000068650
1227	1110	1650	1497	0,284171577	7,158942541	0,000263455	0,003073771	up	ANKRD1	ENSG00000148677
783	643	694	597	-0,284331381	6,154579373	0,000527712	0,005499894	down	BTBD2	ENSG00000133243
1146	996	998	943	-0,284373486	6,743487584	0,000217248	0,002599113	down	LPAR1	ENSG00000198121
2105	1881	1890	1728	-0,284630683	7,640861371	0,000120609	0,0015967	down	ERO1A	ENSG00000197930
390	318	323	317	-0,284970338	5,147241077	0,003009647	0,022404989	down	SPRYD7	ENSG00000123178
398	392	544	521	0,285072884	5,60200171	0,004705674	0,032134746	up	GCC2	ENSG00000135968
634	563	578	507	-0,285096301	5,905124289	0,000947568	0,0089506	down	PML	ENSG00000140464
1767	1490	1642	1312	-0,285126089	7,346128407	0,000796364	0,007763177	down	PTPN23	ENSG00000076201
669	624	602	571	-0,28517943	6,018334706	0,001182136	0,010578486	down	ATP5PD	ENSG00000167863
588	426	476	438	-0,285355767	5,659037943	0,002469136	0,019238941	down	NADK	ENSG00000008130
487	445	425	419	-0,285752348	5,545534384	0,002018135	0,016442613	down	PCBD1	ENSG00000166228
1741	1329	1409	1357	-0,285854209	7,255259961	0,0001751	0,002164911	down	FDPS	ENSG00000160752
751	652	606	662	-0,285890602	6,132864255	0,001763142	0,014752068	down	ARL6IP5	ENSG00000144746
1273	999	1128	925	-0,285924427	6,822797184	0,000720229	0,007119972	down	MICALL1	ENSG00000100139
475	358	374	377	-0,286011614	5,37785489	0,003211176	0,023610959	down	NEURL1B	ENSG00000214357
634	586	902	745	0,28605891	6,225363874	0,001982366	0,016226593	up	DUT	ENSG00000128951
871	661	706	675	-0,28608636	6,25392285	0,000461353	0,004923948	down	BTBD10	ENSG00000148925
316	281	276	264	-0,286177557	4,904036906	0,005864411	0,038252402	down	RABL3	ENSG00000144840
378	324	491	453	0,286214028	5,428193911	0,001559145	0,013376773	up	ITFG1	ENSG00000129636
408	355	520	506	0,286255063	5,548291369	0,001357909	0,011920281	up	SMARCA1	ENSG00000102038
276	262	378	347	0,286690913	5,049764418	0,006816837	0,043324562	up	GATAD1	ENSG00000157259
480	369	549	589	0,286897619	5,697801605	0,002905312	0,021858813	up	ACOX1	ENSG00000161533
386	301	484	439	0,286910681	5,395066777	0,00170847	0,014417539	up	UIMC1	ENSG00000087206
2118	2073	1948	1863	-0,286921658	7,71760434	0,000958732	0,009000401	down	CD68	ENSG00000129226
459	343	379	344	-0,287016316	5,322639256	0,003087488	0,022885728	down	DOCK6	ENSG00000130158
657	510	535	517	-0,287210293	5,862812008	0,000800356	0,007788791	down	KIAA0319L	ENSG00000142687
395	386	533	521	0,287285289	5,586438449	0,004057756	0,028564323	up	PPWD1	ENSG00000113593
2071	1545	2464	2378	0,287374115	7,778948536	0,000157146	0,00198885	up	SLC39A14	ENSG00000104635
1097	863	915	852	-0,287564681	6,609488728	0,000157495	0,001989265	down	STARD13	ENSG00000133121
314	233	396	339	0,287762142	5,067794325	0,005939755	0,038658195	up	C16orf70	ENSG00000125149

906	846	1240	1127	0,287778896	6,747569236	0,000567935	0,005848452	up	CHAMP1	ENSG00000198824
335	314	299	288	-0,288003963	5,024685313	0,006127391	0,039630259	down	TBC1D8B	ENSG00000133138
2800	2005	2245	2067	-0,288067884	7,896162538	0,000443314	0,004764167	down	SLC4A2	ENSG00000164889
852	697	707	690	-0,288138544	6,271982427	0,000212832	0,002559335	down	TMCO3	ENSG00000150403
2855	2424	2555	2218	-0,288539976	8,041546534	7,31E-05	0,001062524	down	SMTN	ENSG00000183963
1272	1136	1641	1607	0,288596126	7,203844024	0,000180297	0,002223859	up	BOD1L1	ENSG00000038219
606	546	744	808	0,288626502	6,143061298	0,002203538	0,017575115	up	CCDC93	ENSG00000125633
2186	1694	2603	2602	0,288650311	7,883227515	0,00010345	0,001403029	up	XPO7	ENSG00000130227
1268	995	1052	986	-0,288703481	6,815905021	0,000162209	0,002033043	down	RNF26	ENSG00000173456
334	270	426	387	0,288742644	5,212548415	0,002396146	0,018773843	up	ZBTB21	ENSG00000173276
801	680	1048	948	0,288902516	6,501894142	0,000172375	0,002138027	up	MT1E	ENSG00000169715
2132	1645	1758	1640	-0,288963476	7,553123433	7,89E-05	0,001136886	down	FNDC3A	ENSG00000102531
294	260	252	248	-0,288981179	4,795374768	0,006806284	0,043282055	down	CDK15	ENSG00000138395
444	422	609	561	0,289105571	5,734851001	0,002065101	0,016737449	up	UTP3	ENSG00000132467
470	321	573	488	0,289176428	5,593470676	0,005222352	0,034798447	up	IMP3	ENSG00000177971
313	251	383	376	0,289252884	5,114591437	0,002960281	0,022175688	up	PSMD9	ENSG00000110801
513	474	691	642	0,28961185	5,922050922	0,001008484	0,009323398	up	SNAP23	ENSG00000092531
3236	2440	2637	2461	-0,28965332	8,138672592	8,69E-05	0,001230834	down	TMEM245	ENSG00000106771
634	640	572	583	-0,289669665	5,998710116	0,004932954	0,033254381	down	LRRK1	ENSG00000154237
2098	1772	1831	1662	-0,289677318	7,59279646	4,26E-05	0,000659406	down	GFPT1	ENSG00000198380
743	597	651	557	-0,289688812	6,061702134	0,000569356	0,005859193	down	RIN1	ENSG00000174791
807	616	969	943	0,289697686	6,440589834	0,000273929	0,003178711	up	RFC2	ENSG00000049541
573	464	739	658	0,289794366	5,988433233	0,00047722	0,005072377	up	EXOC3	ENSG00000180104
7024	6015	9161	8441	0,29016333	9,636990132	9,92E-06	0,000184679	up	NAP1L1	ENSG00000187109
2006	1618	2446	2429	0,290193019	7,788057779	5,64E-05	0,000843067	up	SH2B3	ENSG00000111252
744	616	591	633	-0,290271538	6,084073782	0,001014222	0,00934709	down	ECI2	ENSG00000198721
601	455	752	669	0,29043658	6,012442078	0,00064898	0,006527211	up	UBL3	ENSG00000122042
463	386	569	575	0,290759604	5,702864912	0,001044981	0,009579401	up	RAP1A	ENSG00000116473
533	422	475	385	-0,291309825	5,573144737	0,002454474	0,019134301	down	STAT5B	ENSG00000173757
468	324	557	507	0,291336223	5,597201472	0,002898617	0,021819015	up	DDX51	ENSG00000185163
822	623	679	619	-0,29177915	6,167074298	0,000455921	0,004879394	down	DGCR2	ENSG00000070413
468	390	383	389	-0,291841595	5,420781866	0,001610156	0,013753633	down	C3orf38	ENSG00000179021

704	552	863	829	0,291922378	6,263682231	0,000200366	0,00243275	up	CLCN3	ENSG00000109572
891	727	1123	1059	0,291931461	6,629198003	9,08E-05	0,00127315	up	KIAA1671	ENSG00000197077
1878	1714	1668	1577	-0,291953992	7,488521594	0,000143462	0,001841179	down	ZFP36L1	ENSG00000185650
1336	1077	1674	1579	0,2921383	7,203661583	5,66E-05	0,000844499	up	RCC1	ENSG00000180198
3154	2671	4192	3684	0,29230455	8,475757721	3,43E-05	0,000546739	up	HNRNPAB	ENSG00000197451
1802	1504	2335	2130	0,292456218	7,658900332	3,10E-05	0,000503683	up	NCBP3	ENSG00000074356
443	374	595	509	0,292518916	5,649004905	0,001247684	0,011090605	up	FAM122B	ENSG00000156504
610	409	718	650	0,292877009	5,957473114	0,003107226	0,022999102	up	DENND6A	ENSG00000174839
894	735	1092	1105	0,292889213	6,639808308	0,000166846	0,002081081	up	ANO6	ENSG00000177119
2915	2309	2413	2277	-0,293059034	8,020382387	2,69E-05	0,000448119	down	VAT1	ENSG00000108828
1891	1498	1593	1451	-0,293225923	7,396339241	5,33E-05	0,000804528	down	SNAPC1	ENSG00000023608
1488	1218	1265	1168	-0,293242962	7,073549989	6,05E-05	0,000898408	down	MAN2A1	ENSG00000112893
342	276	455	380	0,293407919	5,247894946	0,003492079	0,025257798	up	WDR20	ENSG00000140153
220	195	291	270	0,293465363	4,680013303	0,00776455	0,047980778	up	TMA16	ENSG00000198498
946	798	820	749	-0,29373368	6,441433332	0,000123876	0,001634387	down	VAMP3	ENSG00000049245
746	644	659	592	-0,294109468	6,115235933	0,00027911	0,003227322	down	SH3BP5L	ENSG00000175137
709	613	933	856	0,294455403	6,342368913	0,000153171	0,001944885	up	PPIE	ENSG00000084072
332	249	268	253	-0,294888863	4,856898715	0,005867552	0,038252402	down	WNT5A	ENSG00000114251
270	232	340	339	0,295316036	4,95284371	0,003980502	0,028135279	up	SRD5A1	ENSG00000145545
382	255	462	396	0,295674011	5,286437372	0,008135218	0,049736931	up	MSTO1	ENSG00000125459
1118	812	1325	1274	0,295858526	6,8793573	0,000320091	0,003622951	up	TCEA1	ENSG00000187735
1222	1149	1142	998	-0,295891289	6,888804006	0,000512437	0,005391355	down	SPART	ENSG00000133104
383	297	294	315	-0,295935277	5,082779068	0,005608436	0,036810398	down	NTN1	ENSG00000065320
1765	1426	1423	1436	-0,296040114	7,309339573	8,15E-05	0,001169134	down	VPS13A	ENSG00000197969
3411	2730	4178	4114	0,296119616	8,550613693	2,04E-05	0,00034951	up	GPI	ENSG00000105220
751	622	962	896	0,296158948	6,396087626	8,91E-05	0,001256947	up	TBC1D2	ENSG00000095383
336	292	407	443	0,296865594	5,274949295	0,004703425	0,032133516	up	GINS1	ENSG00000101003
928	778	799	732	-0,297021849	6,40792255	0,000109205	0,001467	down	B4GALT3	ENSG00000158850
562	488	752	672	0,297249641	6,012939056	0,000375839	0,004149683	up	MEA1	ENSG00000124733
459	349	583	510	0,297339505	5,632548206	0,001105805	0,010054324	up	CYHR1	ENSG00000187954
573	506	744	719	0,297354876	6,05281957	0,000358135	0,00396965	up	ZRANB1	ENSG00000019995
434	316	511	502	0,297758025	5,524688672	0,001553166	0,013340215	up	NIPA1	ENSG00000170113

461	387	381	379	-0,297924359	5,401321961	0,001100196	0,010014618	down	CAMKK2	ENSG00000110931
532	408	429	412	-0,298110594	5,546455998	0,000989719	0,009186694	down	DAGLB	ENSG00000164535
482	388	629	550	0,298292975	5,740932259	0,000653776	0,006554223	up	TRMT2A	ENSG00000099899
1357	1086	1092	1093	-0,298424296	6,922727486	0,000108891	0,001464037	down	DNAJC13	ENSG00000138246
414	354	375	314	-0,298541639	5,258990597	0,002253911	0,017875882	down	ADARB1	ENSG00000197381
407	325	323	332	-0,298612566	5,188245175	0,002565874	0,019865365	down	DOCK2	ENSG00000134516
1283	1083	1120	1002	-0,29879585	6,878904325	7,87E-05	0,001136643	down	E2F7	ENSG00000165891
833	596	635	638	-0,299440385	6,144955982	0,0011599	0,010423664	down	NDFIP2	ENSG00000102471
2240	1798	2813	2657	0,299556426	7,948765362	1,54E-05	0,000273394	up	DHX15	ENSG00000109606
313	265	428	357	0,299672937	5,156833507	0,003504732	0,025323915	up	MSL2	ENSG00000174579
1244	1069	1041	1031	-0,299754603	6,846733445	0,000102878	0,001396496	down	REEP3	ENSG00000165476
385	378	363	323	-0,300085952	5,25361135	0,004611991	0,031613437	down	ALAD	ENSG00000148218
2547	1986	3141	2994	0,300133326	8,113962194	2,03E-05	0,000348006	up	PPME1	ENSG00000214517
356	294	439	443	0,300933862	5,325202978	0,001424767	0,012415937	up	SLCO4A1	ENSG00000101187
8014	6271	10065	9286	0,300980855	9,768736288	6,06E-06	0,000120115	up	IPO5	ENSG00000065150
402	347	329	341	-0,30104825	5,222300803	0,002248497	0,017860312	down	NUDT16	ENSG00000198585
348	284	286	279	-0,301058738	4,976849271	0,002772106	0,021081312	down	WIPI1	ENSG00000070540
374	301	299	304	-0,301388624	5,070819208	0,002623715	0,020210157	down	PGS1	ENSG00000087157
693	538	883	787	0,301397464	6,239636447	0,000178384	0,002203756	up	POLA1	ENSG00000101868
425	341	334	350	-0,301449333	5,252253541	0,002313598	0,018283898	down	EXOC8	ENSG00000116903
722	568	600	552	-0,301727768	6,000751159	0,00025379	0,002981166	down	HYI	ENSG00000178922
1388	1147	1748	1694	0,301781802	7,281241597	3,18E-05	0,000514215	up	STAG2	ENSG00000101972
709	595	909	863	0,301988701	6,325748566	8,40E-05	0,001194592	up	ZBTB44	ENSG00000196323
416	291	457	498	0,302040911	5,440258269	0,006042507	0,039211905	up	COG8	ENSG00000213380
269	231	363	317	0,302097266	4,950815421	0,00346375	0,02511138	up	DEF6	ENSG00000023892
549	411	439	417	-0,302179915	5,573984198	0,001008668	0,009323398	down	TMEM41B	ENSG00000166471
545	450	663	688	0,302547561	5,936930498	0,000544386	0,005643371	up	SCYL2	ENSG00000136021
385	316	446	505	0,3027716	5,434023098	0,004583994	0,031469647	up	ALPK2	ENSG00000198796
549	464	744	635	0,302865559	5,963631082	0,000489855	0,005192465	up	IRS2	ENSG00000185950
275	237	377	320	0,303022376	4,985438597	0,003921268	0,027792502	up	ATF1	ENSG00000123268
675	486	539	494	-0,30346952	5,844913246	0,001129141	0,010218009	down	IMPDH1	ENSG00000106348
634	491	521	482	-0,303589059	5,802451774	0,000438833	0,004722545	down	NXPE3	ENSG00000144815

920	731	1246	1001	0,303669201	6,663577245	0,000514032	0,005393334	up	CDC25A	ENSG00000164045
648	456	747	747	0,303750232	6,080491538	0,000990655	0,009189892	up	PYGO2	ENSG00000163348
394	315	500	464	0,303758593	5,450406502	0,0006792	0,006787188	up	TRIM16L	ENSG00000108448
907	737	1166	1070	0,303951538	6,658627616	4,53E-05	0,000699469	up	CIRBP	ENSG00000099622
1648	1307	2121	1896	0,304075221	7,501049617	2,99E-05	0,000489021	up	DDX18	ENSG00000088205
242	210	297	318	0,304245244	4,807957283	0,007332483	0,045854767	up	SLC25A17	ENSG00000100372
1080	814	886	800	-0,304298608	6,550515245	0,000163415	0,002046511	down	CAVIN3	ENSG00000170955
933	759	1226	1077	0,304302964	6,700226512	6,33E-05	0,000935034	up	TIMM44	ENSG00000104980
669	582	525	591	-0,304324957	5,959272032	0,001856001	0,015363457	down	CAP2	ENSG00000112186
302	238	365	369	0,304355237	5,060370343	0,002645436	0,020337116	up	GID4	ENSG00000141034
2908	2453	2475	2313	-0,3043724	8,056242259	9,38E-06	0,000175693	down	SARS	ENSG00000031698
454	381	380	365	-0,304669703	5,375946696	0,000775326	0,007567587	down	NRIP1	ENSG00000180530
481	343	394	339	-0,304684072	5,351595128	0,003472385	0,025138774	down	NAA38	ENSG00000183011
307	258	254	250	-0,304749334	4,815101867	0,003578739	0,025752658	down	SOCS5	ENSG00000171150
4817	4247	4397	3724	-0,304882948	8,816520518	6,77E-05	0,000994404	down	BCL9L	ENSG00000186174
1600	1216	1268	1235	-0,305058754	7,121852617	8,59E-05	0,001218173	down	HPCAL1	ENSG00000115756
642	560	540	533	-0,305059594	5,901378295	0,000324793	0,003667017	down	WIPF1	ENSG00000115935
576	473	762	667	0,305196243	6,014048798	0,000255307	0,002996719	up	MRPL11	ENSG00000174547
633	542	552	497	-0,305238025	5,867773867	0,000276385	0,003202968	down	FAM50A	ENSG00000071859
318	251	259	248	-0,305292546	4,823507543	0,003695536	0,02644607	down	HARS2	ENSG00000112855
1299	1170	1807	1569	0,305439266	7,249763582	0,000126501	0,001657753	up	GPATCH4	ENSG00000160818
433	313	495	517	0,305623817	5,520868115	0,002163617	0,017321365	up	NT5DC3	ENSG00000111696
778	663	674	612	-0,305680659	6,161356005	0,000122476	0,001617819	down	FLCN	ENSG00000154803
2032	1585	1646	1571	-0,305701798	7,483739331	2,79E-05	0,000461955	down	RC3H2	ENSG00000056586
751	537	912	836	0,305907742	6,303691399	0,000354274	0,003940914	up	MKRN2	ENSG00000075975
8664	6435	7141	6267	-0,305927745	9,541287908	6,44E-05	0,000948634	down	PTPRF	ENSG00000142949
579	440	451	455	-0,305938092	5,658457656	0,00086429	0,008274694	down	PAQR3	ENSG00000163291
2910	2436	2430	2336	-0,305983873	8,051042736	9,31E-06	0,00017457	down	TACC1	ENSG00000147526
364	276	459	412	0,306041562	5,303492314	0,001267903	0,011244579	up	GOSR2	ENSG00000108433
6142	4951	4994	4876	-0,306137907	9,101746456	5,67E-06	0,000113577	down	CTNNA1	ENSG00000044115
311	235	383	360	0,306169947	5,076182929	0,002061963	0,016720742	up	TBCEL	ENSG00000154114
208	159	267	233	0,306702273	4,509012894	0,008029415	0,049225734	up	ZBTB10	ENSG00000205189

695	576	896	837	0,307066957	6,291262486	6,38E-05	0,000940772	up	COPS5	ENSG00000121022
297	215	237	218	-0,307176443	4,669257452	0,007854728	0,048422416	down	IRAK4	ENSG00000198001
777	559	559	623	-0,307322908	6,044389326	0,002972653	0,022257632	down	CYBRD1	ENSG00000071967
422	298	334	305	-0,307453409	5,156584315	0,004043846	0,028522138	down	OGFOD3	ENSG00000181396
270	234	235	214	-0,307664736	4,650558402	0,00542392	0,035826479	down	SPSB1	ENSG00000171621
522	405	420	404	-0,307867308	5,522358381	0,000632863	0,006417814	down	CRAT	ENSG00000095321
282	237	238	224	-0,308008925	4,691800513	0,004117956	0,028896405	down	AGGF1	ENSG00000164252
313	275	417	386	0,308054061	5,186984835	0,001459099	0,012679546	up	CEP95	ENSG00000258890
1036	675	1246	1071	0,308105506	6,707443675	0,002442138	0,01905727	up	SAPCD2	ENSG00000186193
1146	747	1425	1140	0,308220299	6,852469164	0,005136387	0,034291895	up	PKMYT1	ENSG00000127564
690	583	900	838	0,308267007	6,29491059	6,37E-05	0,000940566	up	FAM192A	ENSG00000172775
342	265	392	434	0,308414251	5,229156017	0,004371446	0,030346084	up	HNRNPLL	ENSG00000143889
898	729	745	702	-0,308420867	6,333120223	5,48E-05	0,000823078	down	DEGS1	ENSG00000143753
556	410	676	639	0,308673755	5,893954516	0,000403019	0,004391918	up	ARL4C	ENSG00000188042
1217	926	932	967	-0,308760533	6,726765831	0,000188774	0,002311883	down	CD46	ENSG00000117335
258	202	218	191	-0,308796432	4,517100402	0,007775177	0,048027339	down	MIA2	ENSG00000150527
576	459	735	677	0,308886622	5,995837361	0,000159226	0,002006822	up	CDC45	ENSG00000093009
574	478	494	442	-0,309212615	5,705802113	0,000303436	0,003467297	down	SNRPA1	ENSG00000131876
483	462	669	626	0,309262504	5,871972674	0,000852923	0,008192271	up	NCOA3	ENSG00000124151
545	530	494	466	-0,309650047	5,742711244	0,001392599	0,012183477	down	MRPL32	ENSG00000106591
609	485	745	747	0,309667786	6,075860113	0,000191574	0,002342487	up	MIS12	ENSG00000167842
387	354	340	320	-0,30970445	5,204466799	0,001680426	0,014232336	down	IDI1	ENSG00000067064
468	369	368	375	-0,309744382	5,375276004	0,000982285	0,009123148	down	CAVIN2	ENSG00000168497
449	372	588	534	0,310093739	5,665287422	0,000323763	0,003659182	up	OCRL	ENSG00000122126
948	960	931	780	-0,310314821	6,572731508	0,002089023	0,016887262	down	UQCRH	ENSG00000173660
465	429	603	621	0,310605787	5,791672545	0,001007032	0,009319545	up	PHC3	ENSG00000173889
533	391	662	598	0,310735759	5,831143732	0,000504326	0,005320434	up	AIFM2	ENSG00000042286
1166	973	986	915	-0,31076769	6,727503469	2,48E-05	0,00041722	down	IDS	ENSG00000010404
1001	679	806	678	-0,310799244	6,369830826	0,002001633	0,01634246	down	EMP3	ENSG00000142227
3880	2907	4800	4443	0,311208127	8,699059517	2,19E-05	0,000372095	up	PTTG1IP	ENSG00000183255
924	729	787	680	-0,311430069	6,353281603	0,000104093	0,001408065	down	DENND5A	ENSG00000184014
1534	1180	1271	1134	-0,31163531	7,066300201	5,34E-05	0,000806016	down	POMGNT1	ENSG00000085998

3456	2937	2905	2776	-0,31167524	8,30741384	5,16E-06	0,000104739	down	ASPM	ENSG00000066279
274	222	217	223	-0,311718867	4,624464642	0,006074292	0,039352388	down	TRAF6	ENSG00000175104
3297	2588	4243	3795	0,311756452	8,496771881	9,30E-06	0,00017457	up	AP3D1	ENSG00000065000
939	770	1199	1138	0,311915516	6,719252804	2,46E-05	0,000414536	up	NUP43	ENSG00000120253
2879	2347	2382	2254	-0,311919291	8,014080401	5,62E-06	0,000112738	down	AURKA	ENSG00000087586
287	199	343	320	0,312109996	4,910638235	0,004576527	0,031446411	up	TFCP2L1	ENSG00000115112
1379	1037	1615	1675	0,312221981	7,212927356	0,000155465	0,001969184	up	PUM2	ENSG00000055917
282	210	234	202	-0,312440825	4,610467342	0,007232892	0,045473517	down	LANCL2	ENSG00000132434
805	679	665	652	-0,312446795	6,200333939	8,14E-05	0,001168639	down	TNFAIP1	ENSG00000109079
8884	6967	7326	6711	-0,312524565	9,611986912	3,43E-06	7,25E-05	down	PSMD2	ENSG00000175166
238	218	210	195	-0,313081363	4,505937239	0,007033138	0,044397397	down	PRDM5	ENSG00000138738
872	624	679	642	-0,313123556	6,204698082	0,000441231	0,004745066	down	EMP2	ENSG00000213853
421	402	369	363	-0,313343428	5,355396687	0,001898661	0,015656993	down	PTPN18	ENSG00000072135
425	357	579	493	0,313357662	5,597716055	0,000645302	0,006511307	up	RANBP10	ENSG00000141084
847	717	1104	1039	0,313432948	6,593929152	3,28E-05	0,000526398	up	DNAJC9	ENSG00000213551
2160	1571	2648	2437	0,31343908	7,837009741	6,95E-05	0,001016614	up	MCM2	ENSG00000073111
444	353	542	548	0,313499506	5,623462504	0,000501605	0,00529533	up	ST3GAL2	ENSG00000157350
604	513	793	738	0,313784528	6,110313753	8,93E-05	0,00125813	up	JRK	ENSG00000234616
908	794	750	760	-0,313986145	6,398730665	9,12E-05	0,001277704	down	FLRT2	ENSG00000185070
783	653	1079	892	0,314336083	6,471093707	0,000203044	0,002461411	up	MIDN	ENSG00000167470
1694	1258	2092	1938	0,31440206	7,501817976	4,23E-05	0,000656688	up	SFXN1	ENSG00000164466
570	489	480	459	-0,31441818	5,714054813	0,000237863	0,002819671	down	ARAP3	ENSG00000120318
2280	1858	2003	1667	-0,314708853	7,676073009	5,35E-05	0,000806227	down	TRABD	ENSG00000170638
412	333	357	303	-0,314967425	5,206143633	0,001276222	0,011292561	down	STXBP6	ENSG00000168952
631	452	736	742	0,315129479	6,060118994	0,000525412	0,005490666	up	SCD5	ENSG00000145284
355	309	455	456	0,315555653	5,365394842	0,000816742	0,007907228	up	POP4	ENSG00000105171
1009	764	1273	1153	0,315570849	6,770520483	6,79E-05	0,00099586	up	MSH2	ENSG00000095002
389	374	579	473	0,315663118	5,568821841	0,00298929	0,022306157	up	XRCC2	ENSG00000196584
3692	3111	4434	4882	0,315826621	8,712168028	0,000192498	0,002350078	up	ITGB4	ENSG00000132470
377	273	304	270	-0,316103213	5,00683733	0,00326926	0,023958632	down	PLPP2	ENSG00000141934
11612	9609	14354	14727	0,316512081	10,35171498	7,05E-06	0,000136656	up	YWHAZ	ENSG00000164924
395	272	320	268	-0,316695949	5,041711999	0,006863258	0,043580823	down	LMF2	ENSG00000100258

341	285	292	262	-0,316713369	4,956423669	0,001692962	0,014319174	down	PLEK2	ENSG00000100558
521	446	440	416	-0,316767245	5,582075765	0,000266098	0,003102278	down	FRMD4A	ENSG00000151474
1874	1726	2539	2422	0,316844468	7,800725164	3,95E-05	0,000619611	up	MYBL1	ENSG00000185697
908	718	750	687	-0,316870849	6,32727812	4,46E-05	0,000688906	down	SUCO	ENSG00000094975
1056	1012	991	850	-0,316879154	6,682896408	0,000350045	0,003905063	down	SFN	ENSG00000175793
454	306	531	507	0,316940074	5,551398348	0,001824298	0,015166417	up	HAUS5	ENSG00000249115
981	717	745	750	-0,317099961	6,386041106	0,000242852	0,002863517	down	C12orf75	ENSG00000235162
936	808	785	759	-0,317104728	6,431842582	4,55E-05	0,000701359	down	EPS8	ENSG00000151491
258	247	376	320	0,317685202	4,97700419	0,0044983	0,031018696	up	CCDC97	ENSG00000142039
867	749	1186	1038	0,317704581	6,644257288	4,85E-05	0,000739808	up	PNO1	ENSG00000115946
520	412	659	620	0,317741144	5,850205651	0,000152107	0,001934542	up	GATC	ENSG00000257218
789	641	1007	956	0,317751448	6,465902543	3,01E-05	0,000490364	up	B4GALT1	ENSG00000086062
844	832	762	728	-0,317961472	6,380326846	0,000526422	0,005493818	down	CLDND1	ENSG00000080822
295	227	243	218	-0,317995107	4,693490634	0,004179043	0,029219432	down	TMEM101	ENSG00000091947
5994	4838	4862	4698	-0,318143749	9,062100562	1,96E-06	4,35E-05	down	APP	ENSG00000142192
948	710	1172	1100	0,318568853	6,675424868	4,60E-05	0,000706959	up	PGAM5	ENSG00000247077
2933	2813	4133	3817	0,31879215	8,478451961	0,000100098	0,00136592	up	MT2A	ENSG00000125148
614	523	502	502	-0,319759535	5,813766295	0,000203965	0,00247065	down	NRSN2	ENSG00000125841
1424	1187	1151	1153	-0,319822862	7,010920591	2,59E-05	0,000432134	down	DOCK7	ENSG00000116641
699	580	891	868	0,320026989	6,307641016	3,64E-05	0,000577428	up	CAV2	ENSG00000105971
2512	2362	2304	2025	-0,320226867	7,918133987	9,27E-05	0,001295213	down	S100A11	ENSG00000163191
867	678	747	616	-0,320271325	6,2515504	0,000208436	0,002514995	down	CEP170B	ENSG00000099814
1470	984	1730	1620	0,320560478	7,233696452	0,000597512	0,006108473	up	ADM	ENSG00000148926
1048	829	1359	1222	0,320624507	6,857314273	2,75E-05	0,00045605	up	PDAP1	ENSG00000106244
2239	1935	2873	2878	0,321137932	8,012670399	1,19E-05	0,000216112	up	RABEP1	ENSG00000029725
217	179	271	274	0,321139029	4,627302233	0,004564968	0,031380868	up	MAGOHB	ENSG00000111196
1237	924	1022	879	-0,321453787	6,732134204	9,85E-05	0,00135176	down	FAM20B	ENSG00000116199
322	272	275	249	-0,321468565	4,879228979	0,00184909	0,01533076	down	AKIP1	ENSG00000166452
2491	2128	2003	2070	-0,321545714	7,834346836	2,40E-05	0,000404453	down	AHNAK2	ENSG00000185567
5092	4035	6368	6173	0,321712044	9,135260118	2,09E-06	4,60E-05	up	CNOT1	ENSG00000125107
473	357	584	557	0,321736259	5,684653266	0,000268725	0,003125863	up	KDM4B	ENSG00000127663
413	426	638	527	0,321785292	5,712049112	0,004521326	0,031163634	up	NSRP1	ENSG00000126653

291	211	350	340	0,321956305	4,963815673	0,002100852	0,01695641	up	FBXL5	ENSG00000118564
845	610	652	625	-0,322209273	6,161006224	0,000286602	0,003294328	down	CLDN12	ENSG00000157224
2324	1756	1899	1687	-0,322213649	7,648125577	2,50E-05	0,00041926	down	ADORA2B	ENSG00000170425
249	201	325	295	0,322307886	4,810172875	0,002170522	0,017349567	up	SBDSP1	ENSG00000225648
814	712	1091	1015	0,322328304	6,564918153	2,89E-05	0,000475754	up	GUF1	ENSG00000151806
4798	4218	4138	3829	-0,322649883	8,800672535	3,40E-06	7,20E-05	down	CAPZA1	ENSG00000116489
2254	2080	1973	1862	-0,322780993	7,746298426	3,04E-05	0,000494632	down	SIGMAR1	ENSG00000147955
4465	3181	3503	3192	-0,32295469	8,549738255	8,37E-05	0,001191048	down	PIEZO1	ENSG00000103335
899	681	1137	1037	0,323443853	6,609441205	3,67E-05	0,000581053	up	PDPR	ENSG00000090857
1489	1216	1237	1144	-0,323734996	7,059088493	9,75E-06	0,000181894	down	JPT1	ENSG00000189159
1637	1325	1313	1291	-0,324013209	7,189415913	1,18E-05	0,000213782	down	POGLUT3	ENSG00000178202
474	367	560	597	0,324025217	5,705360588	0,000669922	0,00670743	up	CHST15	ENSG00000182022
1204	1030	1609	1477	0,324049035	7,113424553	1,14E-05	0,00020821	up	NCBP1	ENSG00000136937
534	433	701	633	0,324157185	5,907441443	9,49E-05	0,001319435	up	WFS1	ENSG00000109501
250	201	219	178	-0,324217654	4,48224704	0,006696142	0,042774326	down	BMP1	ENSG00000168487
1104	845	1412	1272	0,324350708	6,911970509	2,99E-05	0,000489021	up	EIF2S3	ENSG00000130741
362	264	441	421	0,324663619	5,281237629	0,00087696	0,008370198	up	NCAPH2	ENSG00000025770
348	316	296	289	-0,324737128	5,039681323	0,001478392	0,012825669	down	DCAF17	ENSG00000115827
1435	1117	1190	1052	-0,324879654	6,972219395	2,72E-05	0,000452062	down	HMG20B	ENSG00000064961
728	597	903	924	0,32497795	6,360743774	5,56E-05	0,000834912	up	FANCD2	ENSG00000144554
669	541	825	843	0,325028547	6,229778907	7,66E-05	0,001109965	up	FAM126A	ENSG00000122591
759	805	747	643	-0,32509186	6,281340902	0,002957852	0,022168185	down	CCNH	ENSG00000134480
486	392	666	547	0,325177236	5,769270122	0,000514362	0,005393334	up	SLC7A6OS	ENSG00000103061
208	158	258	247	0,325651662	4,515822062	0,004348686	0,030215101	up	C19orf12	ENSG00000131943
782	550	1000	833	0,325755548	6,362035895	0,000535758	0,005565078	up	IER2	ENSG00000160888
211	184	283	263	0,325937046	4,627390524	0,003361752	0,024512737	up	BCORL1	ENSG00000085185
896	654	1041	1089	0,326114938	6,581387781	0,000212935	0,002559335	up	COL7A1	ENSG00000114270
277	204	218	204	-0,326209661	4,57167629	0,005229241	0,034821992	down	LYPD6	ENSG00000187123
669	608	648	481	-0,326233637	5,981261384	0,002534266	0,019667474	down	POLR2A	ENSG00000181222
6478	5487	8362	8180	0,326625402	9,532550946	1,10E-06	2,60E-05	up	LASP1	ENSG00000002834
1907	1356	2271	2211	0,327068429	7,650434702	8,11E-05	0,001165269	up	SLC38A2	ENSG00000134294
388	299	328	275	-0,327141177	5,082900857	0,001502991	0,012988287	down	INPP5A	ENSG00000068383

401	347	325	332	-0,327571027	5,208290802	0,000850579	0,008183788	down	RFFL	ENSG00000092871
1462	1330	1830	2037	0,327886781	7,440019399	0,000416725	0,004519084	up	IFIT3	ENSG00000119917
195	188	273	258	0,3281523	4,586809448	0,00634366	0,040892776	up	BEND7	ENSG00000165626
433	308	523	499	0,328367636	5,523839066	0,000551744	0,005704421	up	MKRN1	ENSG00000133606
2135	1671	2716	2540	0,328503836	7,878287475	3,40E-06	7,20E-05	up	MCMBP	ENSG00000197771
304	226	231	233	-0,328693786	4,709562429	0,003904169	0,02770925	down	FN3KRP	ENSG00000141560
3229	2645	4224	3906	0,329059198	8,506272653	9,25E-07	2,24E-05	up	TCOF1	ENSG00000070814
456	389	587	583	0,329144072	5,71837907	0,000166342	0,002076468	up	PARG	ENSG00000227345
479	387	626	573	0,329607869	5,752111354	0,000108701	0,001462747	up	ZKSCAN8	ENSG00000198315
8175	6881	6743	6464	-0,329793359	9,534638858	4,78E-07	1,25E-05	down	HSPD1	ENSG00000144381
482	424	400	395	-0,329833771	5,483312213	0,000283857	0,003272474	down	ISCA1	ENSG00000135070
1876	1628	1562	1514	-0,329999098	7,432760695	6,28E-06	0,000123682	down	CD63	ENSG00000135404
780	623	671	559	-0,330034988	6,109196028	0,000124601	0,001638379	down	PHACTR2	ENSG00000112419
755	680	1035	958	0,330228676	6,482169158	3,49E-05	0,000556633	up	CHD7	ENSG00000171316
592	543	791	785	0,330454577	6,145728496	0,000130123	0,001698055	up	SNRPB2	ENSG00000125870
291	231	229	228	-0,330554375	4,688568472	0,002742706	0,020908929	down	ZNF827	ENSG00000151612
578	407	462	398	-0,330641511	5,595414453	0,00112038	0,010174491	down	HPS5	ENSG00000110756
1427	980	1647	1662	0,330672742	7,212905624	0,000309949	0,003531327	up	GTF3C4	ENSG00000125484
463	369	363	365	-0,33101382	5,3572988	0,000388937	0,004265402	down	RBL2	ENSG00000103479
394	288	457	485	0,331333687	5,407217152	0,001196755	0,010692988	up	LYRM7	ENSG00000186687
1527	1253	1978	1875	0,331367167	7,430218939	3,04E-06	6,50E-05	up	PATL1	ENSG00000166889
5562	3847	6769	6179	0,331400306	9,175807194	0,00012723	0,001665904	up	ATP5F1B	ENSG00000110955
3918	3862	3497	3366	-0,331745517	8,59108395	0,0001512	0,001924578	down	RPL27A	ENSG00000166441
339	321	285	294	-0,332090129	5,029082156	0,002424949	0,018940696	down	CTSL	ENSG00000135047
404	341	538	496	0,332136497	5,538901422	0,000152568	0,001938807	up	DSCC1	ENSG00000136982
721	602	588	570	-0,332143353	6,025560782	4,19E-05	0,000652254	down	SGCB	ENSG00000163069
566	457	695	722	0,332150552	5,992620504	0,000140503	0,001807684	up	MRE11	ENSG00000020922
3846	3455	3307	3106	-0,332246712	8,493240727	4,32E-06	8,97E-05	down	RAC2	ENSG00000128340
537	441	448	408	-0,332262813	5,589938779	0,000129262	0,001689648	down	SGMS2	ENSG00000164023
307	254	258	233	-0,332411209	4,791778017	0,001532648	0,013172083	down	NBEAL1	ENSG00000144426
257	233	355	326	0,332704011	4,940465001	0,001524965	0,01313432	up	ZNF268	ENSG00000090612
1247	1178	1078	1053	-0,33275657	6,90483081	8,34E-05	0,001189116	down	EIF2AK2	ENSG00000055332

5916	5371	5247	4678	-0,33275802	9,122445763	7,53E-06	0,000145155	down	PEA15	ENSG00000162734
463	413	636	582	0,332861685	5,773598179	0,000148287	0,001893705	up	NPEPPS	ENSG00000141279
370	367	522	505	0,332883113	5,529351281	0,001128816	0,010218009	up	TCHP	ENSG00000139437
1346	958	1593	1586	0,333284721	7,153614208	9,42E-05	0,001312161	up	OAT	ENSG00000065154
1654	1466	2244	2099	0,333355694	7,601556884	5,24E-06	0,000105883	up	WARS	ENSG00000140105
354	318	465	469	0,333435512	5,393619915	0,000518315	0,005427452	up	RSBN1L	ENSG00000187257
1286	1230	1806	1706	0,333460532	7,295849149	6,47E-05	0,000951815	up	DNAJC7	ENSG00000168259
387	341	320	317	-0,33376	5,166979108	0,000645857	0,006512675	down	ARMCX1	ENSG00000126947
282	243	224	235	-0,333836171	4,697152774	0,003091995	0,022908205	down	LOX	ENSG00000113083
214	166	256	271	0,333888024	4,574269478	0,004779832	0,032469825	up	MANEA	ENSG00000172469
725	664	1017	919	0,334242851	6,438324417	4,99E-05	0,000759467	up	NEMF	ENSG00000165525
1173	1029	1513	1549	0,33426629	7,099694895	2,68E-05	0,000446457	up	NSD1	ENSG00000165671
1064	698	832	697	-0,334298665	6,426002372	0,001851078	0,015339052	down	GAS6	ENSG00000183087
229	168	291	260	0,334513701	4,6360444	0,003297567	0,024143287	up	ZNF488	ENSG00000265763
302	217	393	327	0,334539238	5,018031532	0,00259653	0,020040512	up	C19orf47	ENSG00000160392
248	220	195	214	-0,334780867	4,532732926	0,006377341	0,041075789	down	PIGC	ENSG00000135845
371	334	495	486	0,334790951	5,463188159	0,000345329	0,003857994	up	TMEM19	ENSG00000139291
1109	883	941	799	-0,334941317	6,61175041	2,78E-05	0,000460867	down	BLCAP	ENSG00000166619
4561	4020	3941	3580	-0,334961959	8,723981485	2,00E-06	4,41E-05	down	RTN4	ENSG00000115310
233	196	205	170	-0,334976534	4,406708206	0,004759457	0,032359724	down	TMEM41A	ENSG00000163900
995	777	785	760	-0,334992858	6,442419143	1,99E-05	0,00034239	down	BEX3	ENSG00000166681
4496	3650	5889	5430	0,335228069	8,980421732	3,97E-07	1,06E-05	up	RAD21	ENSG00000164754
311	272	440	372	0,335355828	5,190193069	0,000963999	0,009028325	up	ATG14	ENSG00000126775
655	520	832	800	0,335527037	6,192895699	2,28E-05	0,000386752	up	WASHC5	ENSG00000164961
316	254	241	256	-0,33597939	4,812417796	0,002305074	0,018225815	down	ZDHHC13	ENSG00000177054
361	269	286	263	-0,336063753	4,953866974	0,001398772	0,012216836	down	USP20	ENSG00000136878
1558	1374	1254	1308	-0,336221536	7,173722545	3,36E-05	0,00053669	down	DYNC1LI2	ENSG00000135720
303	263	406	382	0,336301743	5,148019816	0,000527617	0,005499894	up	TGFBR3	ENSG00000069702
365	292	265	307	-0,336338874	5,015557302	0,003747145	0,026753753	down	THSD4	ENSG00000187720
258	205	334	310	0,336450402	4,858620954	0,001139634	0,010271033	up	WRN	ENSG00000165392
248	216	323	323	0,336752485	4,863887614	0,001652182	0,014027739	up	RINT1	ENSG00000135249
961	934	1359	1293	0,33698265	6,890249295	0,000116119	0,001546499	up	VTI1B	ENSG00000100568

243	195	208	174	-0,337313773	4,434408524	0,004739327	0,032279389	down	ARL14EP	ENSG00000152219
318	263	409	400	0,337794444	5,185244723	0,000448583	0,004814127	up	RSBN1	ENSG00000081019
681	513	524	514	-0,338073443	5,871350269	0,000132307	0,001722212	down	EVA1A	ENSG00000115363
1211	952	1528	1479	0,338074574	7,070779892	6,25E-06	0,000123465	up	PPP1CB	ENSG00000213639
450	342	553	548	0,338125	5,626939178	0,00018222	0,002244013	up	CWF19L1	ENSG00000095485
470	403	374	387	-0,338185798	5,425411219	0,000327262	0,003682646	down	PRKAB2	ENSG00000131791
400	330	300	335	-0,339186431	5,166601163	0,001575829	0,013490104	down	LPCAT4	ENSG00000176454
388	326	525	471	0,339261052	5,481832673	0,000169561	0,002109868	up	ADD2	ENSG00000075340
259	222	211	208	-0,339320543	4,569026068	0,00249006	0,019387893	down	ZDHHC12	ENSG00000160446
1649	1303	2051	2055	0,339569587	7,519237325	5,16E-06	0,000104739	up	SAE1	ENSG00000142230
284	223	226	215	-0,340018025	4,642233435	0,002104454	0,01697666	down	FAM216A	ENSG00000204856
586	449	732	709	0,340184942	6,01211518	4,65E-05	0,000713471	up	CPT1A	ENSG00000110090
216	195	288	286	0,340543003	4,693276128	0,002526332	0,01963448	up	ELMOD2	ENSG00000179387
1297	946	1016	928	-0,340645717	6,77597712	4,71E-05	0,000721774	down	ARHGAP1	ENSG00000175220
895	928	1294	1270	0,340718905	6,840219164	0,000647782	0,006519379	up	SREK1	ENSG00000153914
1137	1010	909	961	-0,340901235	6,722368504	4,60E-05	0,000707003	down	CALD1	ENSG00000122786
462	412	632	590	0,341154961	5,775011599	9,54E-05	0,001320479	up	PANK2	ENSG00000125779
344	307	470	440	0,341307383	5,352189612	0,000273413	0,003175644	up	NSUN5	ENSG00000130305
622	437	814	660	0,341506097	6,041897073	0,000731491	0,007203125	up	TRMT61A	ENSG00000166166
218	192	197	160	-0,341782028	4,339822869	0,006068175	0,03932917	down	CD58	ENSG00000116815
444	329	546	531	0,341813467	5,59345066	0,000179007	0,0022097	up	IPMK	ENSG00000151151
720	580	571	558	-0,342094264	5,994490537	3,27E-05	0,000525257	down	TANC1	ENSG00000115183
221	210	289	314	0,342130862	4,763698175	0,005000947	0,033531823	up	ARID4A	ENSG00000032219
399	302	302	306	-0,342210938	5,104668988	0,000842431	0,00811861	down	ZBTB41	ENSG00000177888
257	213	234	175	-0,342577104	4,533793626	0,007273505	0,045581216	down	DDX60L	ENSG00000181381
1366	1185	1089	1129	-0,342597565	6,969166615	2,03E-05	0,000348006	down	CDC42BPA	ENSG00000143776
704	723	659	591	-0,342602225	6,139188479	0,000842759	0,00811861	down	TBCA	ENSG00000171530
739	699	614	639	-0,343252888	6,145963887	0,000230283	0,002740037	down	GAS2L3	ENSG00000139354
356	264	482	384	0,34328328	5,278178321	0,001657625	0,014066258	up	ZFP36L2	ENSG00000152518
195	143	240	232	0,343584348	4,411833388	0,003906943	0,027716273	up	DMPK	ENSG00000104936
272	223	348	344	0,343733696	4,959082224	0,000707986	0,007011676	up	CEP135	ENSG00000174799
4287	3474	5492	5347	0,343858824	8,915364262	3,37E-07	9,17E-06	up	TMPO	ENSG00000120802

357	324	299	293	-0,344051012	5,067224399	0,000735274	0,007231202	down	ETAA1	ENSG00000143971
730	605	604	555	-0,344260939	6,032742243	1,86E-05	0,000323834	down	SECISBP2L	ENSG00000138593
587	447	738	706	0,344612251	6,013015104	3,69E-05	0,00058374	up	WDFY2	ENSG00000139668
294	282	388	420	0,345162799	5,181424373	0,002373847	0,018636737	up	COG2	ENSG00000135775
1201	935	948	901	-0,345330783	6,706747	4,97E-06	0,000101329	down	RASAL2	ENSG00000075391
405	365	565	515	0,345352	5,595742416	0,000133344	0,001731352	up	OSTC	ENSG00000198856
992	870	799	817	-0,345822778	6,514077694	2,08E-05	0,00035584	down	ZNF462	ENSG00000148143
350	267	418	444	0,345967913	5,273605423	0,000701203	0,006963598	up	PARPBP	ENSG00000185480
648	572	946	769	0,346163044	6,257186332	0,000132634	0,001725019	up	ASNS	ENSG00000070669
234	174	302	269	0,346224934	4,682024188	0,002034953	0,016536254	up	PLXNA3	ENSG00000130827
548	421	414	424	-0,346230232	5,568231519	0,000192851	0,002352539	down	TOR1B	ENSG00000136816
2152	1601	2715	2523	0,346557898	7,865344495	4,59E-06	9,46E-05	up	AGO2	ENSG00000123908
211	157	262	253	0,346563943	4,53505967	0,002677984	0,020506121	up	AIF1L	ENSG00000126878
489	344	369	350	-0,346637859	5,347696281	0,000808362	0,007835861	down	PINK1	ENSG00000158828
1457	1182	1172	1113	-0,346722303	7,012852849	2,67E-06	5,74E-05	down	SEC23IP	ENSG00000107651
166	131	207	209	0,346895092	4,23075775	0,005265412	0,03501781	up	KIF16B	ENSG00000089177
277	216	367	324	0,347520493	4,954223571	0,000717724	0,007103595	up	ZNF740	ENSG00000139651
494	380	380	375	-0,348573683	5,418986067	0,000193281	0,002354081	down	BTRC	ENSG00000166167
529	433	705	645	0,348713225	5,914225997	2,35E-05	0,00039718	up	WDR48	ENSG00000114742
3023	2321	2337	2271	-0,348805685	8,02631155	1,91E-06	4,26E-05	down	UGCG	ENSG00000148154
1092	897	877	843	-0,349042152	6,604775454	3,37E-06	7,14E-05	down	TM4SF1	ENSG00000169908
1200	1040	982	958	-0,349078494	6,778616993	6,42E-06	0,000125789	down	STXBP1	ENSG00000136854
1440	1134	1895	1714	0,349195408	7,327352407	1,97E-06	4,35E-05	up	UBE2G1	ENSG00000132388
343	305	281	280	-0,349214873	4,992959176	0,000640016	0,006470583	down	NT5DC1	ENSG00000178425
1364	1224	1137	1107	-0,349321471	6,988606186	8,15E-06	0,000155123	down	TUBA4A	ENSG00000127824
595	505	495	457	-0,349412838	5,752460921	3,81E-05	0,000600248	down	ZCCHC24	ENSG00000165424
280	297	250	252	-0,349467127	4,832092641	0,006926243	0,043901071	down	CACHD1	ENSG00000158966
236	223	214	184	-0,349467275	4,499629286	0,003774142	0,026897038	down	FOXA2	ENSG00000125798
870	796	802	646	-0,349632799	6,353995097	0,000134542	0,001745455	down	PPP1R13L	ENSG00000104881
562	497	784	706	0,349741548	6,055477418	2,87E-05	0,000474224	up	AEBP2	ENSG00000139154
279	260	244	223	-0,349841917	4,729435361	0,001876633	0,015509436	down	FAM219A	ENSG00000164970
1075	819	797	834	-0,350158036	6,530269129	3,89E-05	0,000610647	down	HIF1AN	ENSG00000166135

2673	2242	3491	3413	0,350187134	8,262689516	4,36E-07	1,15E-05	up	MAVS	ENSG00000088888
303	235	381	374	0,350384755	5,080781489	0,000438342	0,004720541	up	QRSL1	ENSG00000130348
495	380	385	370	-0,35045507	5,419709111	0,000162032	0,002032463	down	GAN	ENSG00000261609
277	247	215	238	-0,35057361	4,687709743	0,003127995	0,023108773	down	AIG1	ENSG00000146416
429	349	345	327	-0,35078113	5,252459065	0,000159341	0,002006822	down	SEZ6L2	ENSG00000174938
1246	1020	979	977	-0,350845177	6,79177637	5,61E-06	0,000112738	down	PLOD2	ENSG00000152952
1395	1086	1077	1061	-0,350956631	6,919936661	6,01E-06	0,000119721	down	MVP	ENSG00000013364
364	269	301	245	-0,351185727	4,953334061	0,001811233	0,015089401	down	ATOX1	ENSG00000177556
833	650	659	620	-0,351304156	6,178681619	1,23E-05	0,000222146	down	ANKS6	ENSG00000165138
742	586	935	929	0,351532018	6,377536525	7,59E-06	0,000145872	up	IREB2	ENSG00000136381
242	194	318	295	0,351766412	4,781630967	0,00081958	0,007924817	up	TMEM65	ENSG00000164983
296	227	384	351	0,351990859	5,040934177	0,000458987	0,00490882	up	RHBDD1	ENSG00000144468
444	300	334	305	-0,352390345	5,181449124	0,001977455	0,01619615	down	HYAL2	ENSG00000068001
600	555	824	805	0,352414697	6,183613686	3,94E-05	0,000617872	up	PFN2	ENSG00000070087
2348	1816	1853	1732	-0,352427576	7,665367996	1,16E-06	2,75E-05	down	IQGAP3	ENSG00000183856
616	509	847	737	0,352513982	6,141447765	1,84E-05	0,0003211	up	CUL5	ENSG00000166266
2355	1792	3009	2808	0,352654117	8,013772982	1,27E-06	2,95E-05	up	PAFAH1B1	ENSG00000007168
860	677	1137	1024	0,352760804	6,588009176	4,47E-06	9,24E-05	up	NT5DC2	ENSG00000168268
1279	1080	981	1052	-0,35340208	6,850123391	2,50E-05	0,000419354	down	RAB3GAP2	ENSG00000118873
1481	1246	2111	1738	0,35355705	7,416385173	1,37E-05	0,000244291	up	MLLT1	ENSG00000130382
179	147	234	225	0,353583152	4,367991576	0,002724239	0,020798772	up	SPIN3	ENSG00000204271
223	220	191	192	-0,353950743	4,447972409	0,004928329	0,033254381	down	PON2	ENSG00000105854
357	317	507	444	0,354005353	5,409143228	0,000198145	0,002407658	up	CDKN1B	ENSG00000111276
231	204	175	200	-0,354053536	4,41917687	0,005591196	0,03672829	down	MICALL2	ENSG00000164877
1501	1316	1180	1250	-0,354218328	7,107644116	1,83E-05	0,000318844	down	TRIOBP	ENSG00000100106
1623	1309	2117	2010	0,354581914	7,519028377	5,57E-07	1,43E-05	up	ARPP19	ENSG00000128989
398	293	512	460	0,354694056	5,439933431	0,000165229	0,002065899	up	GMEB2	ENSG00000101216
257	221	349	325	0,354808706	4,916340542	0,000506292	0,005333923	up	L2HGDH	ENSG00000087299
1042	804	809	778	-0,354832087	6,491903807	6,39E-06	0,00012542	down	LRRC41	ENSG00000132128
1149	1004	987	872	-0,35529114	6,718996772	3,90E-06	8,18E-05	down	HECTD3	ENSG00000126107
349	284	485	408	0,355521197	5,317623492	0,000274087	0,003178711	up	MRPL18	ENSG00000112110
763	635	673	533	-0,355838417	6,094159389	0,000103564	0,001403356	down	MYL9	ENSG00000101335

383	336	520	495	0,356013358	5,502643733	7,66E-05	0,001109965	up	HAUS6	ENSG00000147874
1037	991	894	860	-0,356195613	6,63675826	3,33E-05	0,000533696	down	NLN	ENSG00000123213
263	208	336	328	0,356368082	4,894409021	0,000608338	0,00620281	up	TFDP2	ENSG00000114126
285	184	331	327	0,35648859	4,882231033	0,003439886	0,02496168	up	GPR176	ENSG00000166073
402	404	558	584	0,356529902	5,672291231	0,000953703	0,008986679	up	CLGN	ENSG00000153132
377	354	529	505	0,356753807	5,529021137	0,000166301	0,002076468	up	CIPC	ENSG00000198894
758	597	951	958	0,357358945	6,409585386	9,08E-06	0,00017089	up	USP33	ENSG00000077254
617	544	829	812	0,357480793	6,192001763	1,23E-05	0,000221227	up	TRMT6	ENSG00000089195
944	719	738	689	-0,357548515	6,339785015	7,90E-06	0,000150786	down	TIMP1	ENSG00000102265
335	235	397	405	0,358164066	5,164668087	0,000823776	0,007955481	up	FOXRED2	ENSG00000100350
165	133	219	202	0,35842213	4,24242003	0,003428581	0,024891273	up	KATNA1	ENSG00000186625
993	774	1280	1212	0,358571344	6,791258071	2,99E-06	6,41E-05	up	IDH3B	ENSG00000101365
934	823	765	747	-0,358807742	6,424692922	6,26E-06	0,000123465	down	TTC17	ENSG00000052841
520	436	666	685	0,359584088	5,912301288	3,69E-05	0,00058374	up	NDRG1	ENSG00000104419
927	751	698	740	-0,360065417	6,354231065	1,72E-05	0,000301373	down	ATP2B4	ENSG00000058668
447	344	566	551	0,360081829	5,638061343	5,10E-05	0,000773098	up	NAA40	ENSG00000110583
767	624	970	995	0,360323618	6,450253036	8,54E-06	0,000161748	up	GGCX	ENSG00000115486
212	200	174	180	-0,361018121	4,339675967	0,00448052	0,030923556	down	ZNF841	ENSG00000197608
1185	947	1525	1489	0,361634607	7,064127692	1,27E-06	2,95E-05	up	C2CD3	ENSG00000168014
2277	1894	1891	1688	-0,362140771	7,667497213	3,36E-07	9,14E-06	down	ELOVL1	ENSG00000066322
2100	1770	2868	2620	0,36228064	7,925413431	1,98E-07	5,74E-06	up	PDS5A	ENSG00000121892
391	304	489	494	0,362396608	5,454263073	0,000101191	0,001377207	up	POP1	ENSG00000104356
197	160	167	139	-0,36246914	4,131101574	0,004936431	0,033257196	down	THEM4	ENSG00000159445
1168	938	1545	1437	0,362832556	7,047393681	8,38E-07	2,04E-05	up	DHX38	ENSG00000140829
270	210	361	319	0,362955332	4,924774431	0,000467885	0,004986814	up	ZBTB5	ENSG00000168795
457	376	622	559	0,363131751	5,715958424	2,55E-05	0,00042754	up	PDE10A	ENSG00000112541
328	298	272	265	-0,363166414	4,937599343	0,000504698	0,005320745	down	TTLL7	ENSG00000137941
191	156	261	231	0,363265866	4,462418417	0,001779117	0,014861706	up	RARS2	ENSG00000146282
1080	970	923	838	-0,363524298	6,645915193	3,60E-06	7,58E-05	down	TP53BP2	ENSG00000143514
1378	1009	1060	976	-0,363687433	6,855508386	1,07E-05	0,00019691	down	EDEM1	ENSG00000134109
460	358	376	324	-0,36373865	5,317219252	0,000148221	0,001893705	down	PACSIN3	ENSG00000165912
336	253	425	409	0,363937059	5,217344006	0,000171673	0,002132724	up	ZNF587	ENSG00000198466

214	167	170	156	-0,363942872	4,222734685	0,003466084	0,025112013	down	NINJ1	ENSG00000131669
1957	1555	2534	2438	0,364044139	7,783396096	2,65E-07	7,44E-06	up	VPS35	ENSG00000069329
210	140	260	235	0,364215365	4,470906964	0,003814115	0,027156992	up	GCDH	ENSG00000105607
599	474	458	459	-0,364532625	5,707629491	3,39E-05	0,000541358	down	NLRP1	ENSG00000091592
1052	874	813	834	-0,364700424	6,55193534	4,45E-06	9,21E-05	down	HSDL2	ENSG00000119471
507	414	409	379	-0,364727329	5,488786635	3,52E-05	0,000560094	down	RBMXL1	ENSG00000213516
1298	1116	1781	1650	0,365013794	7,24834372	5,95E-07	1,51E-05	up	DNAJC10	ENSG00000077232
510	334	665	527	0,365427684	5,727245623	0,001902459	0,015656993	up	ATAD3B	ENSG00000160072
1992	1707	2650	2606	0,365735813	7,863206218	3,49E-07	9,44E-06	up	CDC42EP3	ENSG00000163171
160	148	223	215	0,366283824	4,296220557	0,003171962	0,023366884	up	KIF7	ENSG00000166813
1528	1198	1986	1880	0,366758411	7,419622229	4,12E-07	1,09E-05	up	PDZD8	ENSG00000165650
3868	3721	3438	3095	-0,366776198	8,538136201	1,12E-05	0,000204723	down	ANP32B	ENSG00000136938
332	293	457	432	0,366826774	5,308002391	9,47E-05	0,001317478	up	ID1	ENSG00000125968
2467	2205	2070	1934	-0,367218105	7,833047916	5,69E-07	1,46E-05	down	GOLM1	ENSG00000135052
2446	1921	1933	1791	-0,367221959	7,728315349	2,53E-07	7,12E-06	down	ITPR3	ENSG00000096433
2081	1758	2877	2588	0,367384276	7,916848477	1,85E-07	5,43E-06	up	DBN1	ENSG00000113758
715	556	977	829	0,36753134	6,32289492	1,09E-05	0,000200028	up	GATD1	ENSG00000177225
1942	1582	1574	1435	-0,367700642	7,420763483	2,56E-07	7,19E-06	down	TXNIP	ENSG00000265972
858	695	704	622	-0,367933492	6,239075827	3,22E-06	6,86E-05	down	STIM1	ENSG00000167323
442	358	607	531	0,368041627	5,660290239	3,22E-05	0,000518974	up	ARL2BP	ENSG00000102931
419	349	546	546	0,368164902	5,602753347	3,71E-05	0,000585576	up	IDH2	ENSG00000182054
488	384	665	575	0,368270129	5,783092996	3,23E-05	0,000520268	up	KATNB1	ENSG00000140854
331	322	268	291	-0,368318885	4,998412123	0,001717565	0,014456404	down	CYP4V2	ENSG00000145476
4478	3669	5739	5833	0,368543096	8,999890024	2,33E-07	6,64E-06	up	TMBIM6	ENSG00000139644
250	242	215	206	-0,368658285	4,591178484	0,002007184	0,01637057	down	ZNF532	ENSG00000074657
133	110	187	159	0,368946493	3,958377221	0,006521991	0,041833954	up	LSM6	ENSG00000164167
195	154	275	222	0,36934907	4,473506774	0,003001821	0,022378916	up	MIS18A	ENSG00000159055
3094	2587	4212	3881	0,369406341	8,482042742	4,03E-08	1,33E-06	up	ELAC2	ENSG00000006744
125	106	173	156	0,369415021	3,886994278	0,007246865	0,045487701	up	CLCN5	ENSG00000171365
204	133	227	250	0,369702647	4,418310503	0,007958929	0,048909458	up	PSEN2	ENSG00000143801
1227	939	1554	1521	0,369881898	7,089426702	1,50E-06	3,41E-05	up	ICE1	ENSG00000164151
1348	1099	1811	1674	0,370440973	7,26833336	2,71E-07	7,59E-06	up	TPM1	ENSG00000140416

1706	1418	1468	1199	-0,371053731	7,24657167	5,60E-06	0,000112691	down	TNFRSF12A	ENSG00000006327
452	386	356	358	-0,371242744	5,351504545	6,80E-05	0,000996415	down	KAZN	ENSG00000189337
1011	877	1250	1439	0,371584896	6,898754321	0,000141328	0,001815284	up	DPYSL2	ENSG00000092964
214	138	268	232	0,37163315	4,482140771	0,004748566	0,032323993	up	ANKRD10	ENSG00000088448
742	623	1001	946	0,371797992	6,430930143	1,24E-06	2,91E-05	up	ZNF770	ENSG00000198146
133	118	172	186	0,37182852	4,007221633	0,007667811	0,047528595	up	CXXC5	ENSG00000171604
483	408	680	592	0,371895906	5,818411897	1,98E-05	0,00034164	up	SYAP1	ENSG00000169895
638	565	532	494	-0,372077209	5,872775458	1,23E-05	0,000221344	down	PCBP4	ENSG00000090097
4187	3210	3293	2988	-0,37209302	8,484786954	2,20E-07	6,32E-06	down	DAP	ENSG00000112977
578	533	821	768	0,372223285	6,138865076	1,20E-05	0,000217823	up	KCTD9	ENSG00000104756
262	230	344	358	0,372281196	4,968302246	0,00047341	0,005035329	up	ANKRD26	ENSG00000107890
342	220	392	404	0,372282183	5,148936683	0,001884518	0,015566318	up	LMAN2L	ENSG00000114988
675	620	932	920	0,372708545	6,359476261	7,83E-06	0,000149755	up	BNIP3L	ENSG00000104765
1016	740	842	649	-0,372828658	6,408980164	0,000230439	0,002740037	down	TSPAN4	ENSG00000214063
328	283	260	260	-0,373055591	4,897018146	0,000334192	0,003752469	down	TCP11L1	ENSG00000176148
253	178	327	287	0,373320675	4,774385204	0,001052036	0,00962703	up	DDX19A	ENSG00000168872
497	308	581	558	0,373364281	5,661588594	0,00120544	0,010764396	up	SHISA2	ENSG00000180730
407	319	295	321	-0,373884836	5,141595704	0,00037082	0,004107318	down	ACSS2	ENSG00000131069
1093	921	874	839	-0,374048342	6,612775366	6,36E-07	1,60E-05	down	SCARB1	ENSG00000073060
255	256	214	222	-0,374127022	4,64441454	0,003109952	0,023008313	down	NRGN	ENSG00000154146
1103	934	1531	1382	0,374211253	7,008430314	5,86E-07	1,49E-05	up	LRRC8A	ENSG00000136802
964	750	1249	1195	0,374674597	6,756566362	1,25E-06	2,93E-05	up	WWP2	ENSG00000198373
2025	1798	2804	2672	0,374704643	7,917791581	2,53E-07	7,12E-06	up	LRRFIP1	ENSG00000124831
203	162	157	153	-0,374729488	4,157093622	0,002896866	0,021816411	down	PARP12	ENSG00000059378
216	152	174	138	-0,375175637	4,165753247	0,007200842	0,045327066	down	FKRP	ENSG00000181027
177	166	241	250	0,375669574	4,45574939	0,002611865	0,020138854	up	ITPKB	ENSG00000143772
1424	1053	1834	1695	0,375904149	7,283972462	1,47E-06	3,35E-05	up	UBE3C	ENSG00000009335
500	421	431	352	-0,376179484	5,484556859	7,88E-05	0,001136833	down	SH3D19	ENSG00000109686
387	279	299	265	-0,376289032	5,014310792	0,000487085	0,005166631	down	TMEM205	ENSG00000105518
1100	884	923	762	-0,376319628	6,587892716	5,42E-06	0,000109438	down	DGKZ	ENSG00000149091
877	587	666	569	-0,376479265	6,141832637	0,000310987	0,003537963	down	ACTR1B	ENSG00000115073
257	219	200	204	-0,376522208	4,537186385	0,000966057	0,009031851	down	CORO2B	ENSG00000103647

1167	1188	1148	874	-0,376548408	6,847711584	0,000893556	0,008492067	down	TRAPPC1	ENSG00000170043
708	539	574	483	-0,376619575	5,916765993	2,92E-05	0,000479095	down	MAFF	ENSG00000185022
318	310	284	251	-0,376890534	4,938298704	0,000819066	0,007924778	down	CHMP2A	ENSG00000130724
450	425	392	353	-0,376921151	5,414398827	0,000126036	0,001653054	down	USP25	ENSG00000155313
232	221	184	201	-0,377134694	4,468711888	0,003325799	0,0243156	down	NFATC2	ENSG00000101096
563	540	497	443	-0,377264083	5,748662525	8,36E-05	0,001190565	down	MESD	ENSG00000117899
311	250	388	414	0,377558034	5,156965294	0,000279618	0,003228678	up	THAP5	ENSG00000177683
241	180	309	293	0,377597889	4,744954881	0,00054666	0,00565707	up	IFT52	ENSG00000101052
421	345	355	295	-0,377647578	5,218106569	0,000131629	0,001714815	down	TNK2	ENSG00000061938
605	564	512	482	-0,378453466	5,830683632	2,91E-05	0,000478033	down	UFC1	ENSG00000143222
172	128	137	117	-0,378684499	3,874552004	0,007009048	0,044299353	down	NINL	ENSG00000101004
3471	3061	4642	4734	0,379227674	8,692039705	4,69E-07	1,23E-05	up	NT5E	ENSG00000135318
876	746	1206	1121	0,379294107	6,683813679	3,55E-07	9,55E-06	up	HELLS	ENSG00000119969
212	181	307	257	0,379338692	4,650322917	0,001058109	0,009676903	up	NKAP	ENSG00000101882
925	767	1259	1167	0,379428616	6,743521863	5,65E-07	1,45E-05	up	MCM10	ENSG00000065328
458	375	388	318	-0,379580068	5,337720098	9,65E-05	0,001331494	down	C1GALT1	ENSG00000106392
185	162	152	142	-0,379680853	4,084136825	0,003156952	0,023289459	down	GLIS3	ENSG00000107249
291	298	244	257	-0,379924345	4,846670777	0,0026864	0,020560427	down	TANK	ENSG00000136560
483	474	697	681	0,379948037	5,931424307	7,15E-05	0,001042158	up	MAX	ENSG00000125952
631	430	739	772	0,380169546	6,064977787	0,000160925	0,002021817	up	TGFBR1	ENSG00000106799
354	322	283	290	-0,38028328	5,040481657	0,000277495	0,003213439	down	PSD3	ENSG00000156011
525	422	690	668	0,381109962	5,909629242	4,45E-06	9,21E-05	up	CCDC88C	ENSG00000015133
397	311	293	305	-0,381181138	5,102389174	0,000193207	0,002354081	down	PDE4D	ENSG00000113448
365	292	502	441	0,381298771	5,385263168	4,02E-05	0,000628694	up	FBXO38	ENSG00000145868
932	731	1238	1146	0,381376891	6,717391346	3,28E-07	8,97E-06	up	ATXN1L	ENSG00000224470
263	193	178	206	-0,382125894	4,469360801	0,004234807	0,029542818	down	SEMA3B	ENSG00000012171
379	300	276	297	-0,3823395	5,042083982	0,000291921	0,003348039	down	INSIG1	ENSG00000186480
3077	2585	4223	3919	0,382823618	8,485104525	1,27E-08	4,61E-07	up	YWHAG	ENSG00000170027
356	254	270	244	-0,383113514	4,885099773	0,000611361	0,006225455	down	LRSAM1	ENSG00000148356
387	337	307	305	-0,383215535	5,136452325	9,60E-05	0,001327083	down	SKAP2	ENSG00000005020
220	185	284	298	0,383502019	4,695409884	0,000694321	0,006907186	up	UBE2Q2	ENSG00000140367
592	499	447	474	-0,383637595	5,725242986	2,86E-05	0,000472293	down	ELF1	ENSG00000120690

627	507	484	473	-0,383669605	5,779381806	6,85E-06	0,000133731	down	NCS1	ENSG00000107130
298	271	235	246	-0,383867534	4,791389409	0,000673378	0,006737689	down	TRIQK	ENSG00000205133
324	282	260	252	-0,383948318	4,880511459	0,000206448	0,002498779	down	CTNNBIP1	ENSG00000178585
269	226	224	194	-0,384540669	4,589179642	0,000644723	0,006509693	down	OSGIN1	ENSG00000140961
377	328	520	495	0,384598766	5,490575686	1,86E-05	0,000323788	up	ZNF252P	ENSG00000196922
1848	1510	2530	2301	0,384633151	7,732042624	4,13E-08	1,35E-06	up	STT3B	ENSG00000163527
279	163	345	286	0,3848587	4,809951835	0,007367109	0,046000737	up	THAP11	ENSG00000168286
7667	6137	5569	6034	-0,385288107	9,38168529	2,04E-06	4,49E-05	down	GPRC5A	ENSG00000013588
334	230	414	395	0,385461885	5,164829409	0,000257812	0,003021552	up	ZNF654	ENSG00000175105
207	183	156	173	-0,385503397	4,248824457	0,003015172	0,022435362	down	PFKFB4	ENSG00000114268
245	215	183	205	-0,385515187	4,484901477	0,001959013	0,016070528	down	NLK	ENSG00000087095
312	198	377	353	0,385781406	5,018087282	0,001260108	0,011181838	up	CCDC85B	ENSG00000175602
429	406	353	353	-0,385915797	5,34315153	0,000130624	0,001703163	down	HOXB9	ENSG00000170689
700	553	563	493	-0,385925064	5,921016285	5,19E-06	0,000105168	down	TMEM250	ENSG00000238227
527	391	682	637	0,386062401	5,865074895	9,46E-06	0,000176993	up	SNTB2	ENSG00000168807
1169	1067	1715	1520	0,386767169	7,153570364	1,26E-06	2,95E-05	up	CHAF1A	ENSG00000167670
1353	1033	969	1033	-0,386838533	6,846673119	7,01E-06	0,000136067	down	NNT	ENSG00000112992
382	296	505	471	0,386842691	5,432757284	1,96E-05	0,000338074	up	POMT1	ENSG00000130714
278	228	369	360	0,387047211	5,015358349	0,000107392	0,001446388	up	MINDY2	ENSG00000128923
2635	1935	1971	1861	-0,387128984	7,781224539	7,76E-07	1,90E-05	down	CSRP1	ENSG00000159176
3554	2900	4732	4561	0,387219848	8,67452435	8,89E-09	3,30E-07	up	SF3B1	ENSG00000115524
335	260	437	420	0,387772598	5,246367996	4,16E-05	0,00064923	up	TANGO6	ENSG00000103047
132	127	190	184	0,38781895	4,06230703	0,004212942	0,029416717	up	ETFDH	ENSG00000171503
140	109	184	175	0,388150202	4,003405124	0,003004617	0,022389009	up	PLA2G12A	ENSG00000123739
729	599	949	966	0,389430838	6,400707139	1,01E-06	2,42E-05	up	ARHGAP26	ENSG00000145819
442	342	359	300	-0,3896104	5,244457771	9,28E-05	0,001295788	down	GAS8	ENSG00000141013
1513	1472	1351	1178	-0,389870704	7,181180981	1,33E-05	0,000237146	down	PRKACA	ENSG00000072062
274	194	338	336	0,390237265	4,90185187	0,000391911	0,004294975	up	VPS16	ENSG00000215305
425	353	336	318	-0,390436594	5,235249355	2,90E-05	0,000478033	down	CHFR	ENSG00000072609
1262	905	997	818	-0,390637408	6,703182658	2,18E-05	0,000371269	down	DHCR7	ENSG00000172893
256	200	326	332	0,390681866	4,867517826	0,000261481	0,003058938	up	DNAL1	ENSG00000119661
483	416	384	372	-0,390707972	5,444091203	1,44E-05	0,00025538	down	ADAMTS6	ENSG00000049192

169	133	214	222	0,390888803	4,279693664	0,001700006	0,014363109	up	ZNF107	ENSG00000196247
406	331	526	538	0,390956628	5,556121811	1,79E-05	0,00031336	up	TGIF2	ENSG00000118707
200	171	172	140	-0,390996338	4,174143001	0,002163916	0,017321365	down	ZBTB45	ENSG00000119574
428	403	353	347	-0,391192696	5,333668301	9,12E-05	0,001277599	down	DNM1	ENSG00000106976
439	317	569	521	0,391322459	5,589012902	2,68E-05	0,000447058	up	ATXN7L3B	ENSG00000253719
182	156	248	241	0,392269459	4,442506551	0,000725019	0,007148462	up	ATXN3	ENSG00000066427
576	470	457	421	-0,392326211	5,659521754	4,48E-06	9,25E-05	down	PDE4A	ENSG00000065989
650	520	530	452	-0,392589797	5,819843687	7,35E-06	0,000141767	down	SORD	ENSG00000140263
846	597	1099	980	0,392791094	6,515247886	6,95E-06	0,00013508	up	PIMREG	ENSG00000129195
313	232	221	235	-0,393062474	4,720289697	0,00080178	0,007791511	down	SLC2A3	ENSG00000059804
638	512	525	440	-0,39321919	5,794850413	1,07E-05	0,000196764	down	ZNF367	ENSG00000165244
471	360	353	343	-0,39322916	5,326335733	3,51E-05	0,000558058	down	MAMLD1	ENSG00000013619
125	117	192	159	0,393361063	3,968604296	0,005430851	0,035841767	up	ZNF566	ENSG00000186017
390	306	290	293	-0,393611309	5,072390808	9,53E-05	0,001320479	down	RAB29	ENSG00000117280
1324	1214	1138	1000	-0,393940689	6,941803113	1,46E-06	3,33E-05	down	CREB3L1	ENSG00000157613
714	607	945	968	0,39437535	6,3973098	1,07E-06	2,54E-05	up	NFYA	ENSG00000001167
330	311	290	249	-0,394626412	4,958701313	0,000287345	0,003300431	down	FAM222B	ENSG00000173065
210	163	275	265	0,394664823	4,582789688	0,000455664	0,004879394	up	EEF1AKMT2	ENSG00000203791
257	208	181	208	-0,395019674	4,494209587	0,0017848	0,014893152	down	AGPAT4	ENSG00000026652
35020	39508	31582	31719	-0,395376024	11,83352635	0,000852114	0,008192271	down	MT-CO3	ENSG00000198938
637	566	534	476	-0,395401104	5,862526224	4,77E-06	9,75E-05	down	ARL2	ENSG00000213465
438	330	589	523	0,395748051	5,615485448	1,27E-05	0,00022697	up	EXOSC6	ENSG00000223496
1039	924	869	779	-0,396279959	6,568370819	5,00E-07	1,29E-05	down	ARHGAP17	ENSG00000140750
1173	893	1608	1384	0,396553037	7,036408756	8,34E-07	2,03E-05	up	MYBL2	ENSG00000101057
297	230	400	364	0,396574875	5,077667525	6,10E-05	0,000904171	up	SLC11A2	ENSG00000110911
1424	1382	1168	1193	-0,396602457	7,088732229	1,20E-05	0,000217756	down	HSPE1	ENSG00000115541
460	460	703	639	0,396692656	5,885258548	5,75E-05	0,000856692	up	WDR76	ENSG00000092470
286	289	231	252	-0,397098265	4,803999669	0,001990088	0,016265302	down	CAPRIN2	ENSG00000110888
207	212	181	171	-0,397229454	4,349835235	0,002988236	0,022306157	down	ZNF438	ENSG00000183621
361	253	268	244	-0,397330768	4,887552316	0,000514923	0,005395574	down	CLSTN3	ENSG00000139182
328	234	431	383	0,397417517	5,167807839	0,000118091	0,001570058	up	DCUN1D1	ENSG00000043093
277	210	354	352	0,397798844	4,964997198	0,000119682	0,001588495	up	RCAN1	ENSG00000159200

2511	2363	2255	1852	-0,397827107	7,8839498	7,88E-06	0,000150586	down	MT-ND4L	ENSG00000212907
234	179	176	169	-0,397893148	4,322389294	0,001130299	0,010218009	down	RELCH	ENSG00000134444
504	465	711	699	0,397925264	5,957080702	7,07E-06	0,000136978	up	ANKRD40	ENSG00000154945
318	310	273	254	-0,397987238	4,928823696	0,000388682	0,004265402	down	DECR1	ENSG00000104325
130	120	203	161	0,3982865	4,017813867	0,005427101	0,03583225	up	GOLGA8B	ENSG00000215252
553	468	742	741	0,398492152	6,029374944	1,73E-06	3,89E-05	up	CRNKL1	ENSG00000101343
4205	3682	3340	3265	-0,398669953	8,573872673	1,82E-08	6,36E-07	down	LIMA1	ENSG00000050405
314	226	383	399	0,399015667	5,111602808	0,000208224	0,0025144	up	UNC93B1	ENSG00000110057
882	729	692	654	-0,399174883	6,278972514	1,95E-07	5,69E-06	down	TBC1D5	ENSG00000131374
355	223	243	236	-0,399808217	4,795807804	0,003338325	0,024361302	down	WDR37	ENSG00000047056
342	263	240	264	-0,399818398	4,867866453	0,000460813	0,004921577	down	COX15	ENSG00000014919
613	509	855	777	0,399885767	6,164904555	4,97E-07	1,29E-05	up	TINAGL1	ENSG00000142910
312	237	396	401	0,400279747	5,137784753	7,52E-05	0,001091214	up	ROR1	ENSG00000185483
402	350	308	320	-0,400436645	5,183412363	6,10E-05	0,000904171	down	BTBD3	ENSG00000132640
1186	914	855	892	-0,40049756	6,657263901	1,22E-06	2,86E-05	down	CEMIP2	ENSG00000135048
408	341	309	316	-0,400607477	5,17641823	4,09E-05	0,000639748	down	COMMD2	ENSG00000114744
586	488	789	773	0,400617609	6,10270114	7,48E-07	1,84E-05	up	NUP54	ENSG00000138750
351	261	274	236	-0,40081989	4,883059942	0,0002587	0,003029675	down	CNTNAP1	ENSG00000108797
200	161	151	150	-0,401339552	4,129643102	0,001488843	0,012887542	down	ENOX2	ENSG00000165675
317	261	423	418	0,401414984	5,214208959	2,96E-05	0,000485972	up	EVI2A	ENSG00000126860
252	199	194	182	-0,401434775	4,447254846	0,000529328	0,005509337	down	NIPSNAP3A	ENSG00000136783
2149	2144	1956	1659	-0,401733175	7,702449632	2,35E-05	0,00039718	down	S100A6	ENSG00000197956
220	205	182	173	-0,401748539	4,365464825	0,000873671	0,008343937	down	CARMIL1	ENSG00000079691
592	467	788	752	0,401781835	6,081272476	6,84E-07	1,70E-05	up	C12orf4	ENSG00000047621
479	351	642	564	0,401822377	5,729214145	1,25E-05	0,000225139	up	NUDT19	ENSG00000213965
621	497	460	471	-0,401980954	5,750474335	6,00E-06	0,000119613	down	TOPORS	ENSG00000197579
281	201	391	310	0,402379244	4,950990614	0,000694929	0,006908806	up	ALDH16A1	ENSG00000161618
4755	4150	4083	3373	-0,402507896	8,747244685	3,88E-07	1,03E-05	down	NCOR2	ENSG00000196498
170	145	235	224	0,402548893	4,347809043	0,000752257	0,00736557	up	TIMM8A	ENSG00000126953
163	158	136	132	-0,402833435	3,96463373	0,003552789	0,025613395	down	SATB1	ENSG00000182568
303	226	235	205	-0,403729622	4,673117807	0,000417709	0,004526277	down	IL6R	ENSG00000160712
633	489	835	798	0,40381778	6,164603006	5,84E-07	1,49E-05	up	RNF169	ENSG00000166439

243	230	355	336	0,403819872	4,931856632	0,000184141	0,002261751	up	ZFP90	ENSG00000184939
308	203	209	214	-0,403893873	4,619560221	0,002553636	0,01979801	down	MEGF8	ENSG00000105429
205	204	292	306	0,404101837	4,725572939	0,001130819	0,010218009	up	ARL4A	ENSG00000122644
737	601	963	986	0,404428951	6,419776713	6,40E-07	1,60E-05	up	FAM168A	ENSG00000054965
825	682	698	558	-0,405106216	6,180150117	5,61E-06	0,000112738	down	NPTN	ENSG00000156642
988	918	1420	1371	0,405114029	6,934998004	6,96E-07	1,72E-05	up	MCM8	ENSG00000125885
991	896	1396	1365	0,405602716	6,91948542	3,42E-07	9,30E-06	up	RCOR1	ENSG00000089902
407	357	552	564	0,405881886	5,618552722	1,10E-05	0,000200841	up	INPP5F	ENSG00000198825
333	304	286	245	-0,405890931	4,943662695	0,000140094	0,001803914	down	CEP120	ENSG00000168944
314	255	438	393	0,406190739	5,194071649	2,24E-05	0,00038141	up	VPS50	ENSG00000004766
424	376	571	598	0,406281621	5,685268886	1,76E-05	0,000307995	up	LAP3	ENSG00000002549
1341	1113	1878	1708	0,40638944	7,293861477	1,91E-08	6,66E-07	up	NOP14	ENSG00000087269
1278	1093	1039	935	-0,406648365	6,834450378	9,53E-08	2,96E-06	down	IL7R	ENSG00000168685
242	170	293	307	0,407297613	4,72922401	0,00060584	0,006181403	up	ATG101	ENSG00000123395
158	144	120	131	-0,407456749	3,874529806	0,004331711	0,03012411	down	ADGRV1	ENSG00000164199
518	416	719	646	0,407576553	5,904935777	1,07E-06	2,53E-05	up	GPT2	ENSG00000166123
2412	1682	3136	2811	0,40765525	8,021292953	2,26E-06	4,94E-05	up	TK1	ENSG00000167900
253	195	324	330	0,407687044	4,851751029	0,000149549	0,001906689	up	GFOD2	ENSG00000141098
246	202	341	314	0,407742819	4,853141273	7,89E-05	0,001136886	up	SNX18	ENSG00000178996
217	161	286	266	0,40775445	4,608442894	0,000310393	0,003533788	up	RNF121	ENSG00000137522
675	592	972	884	0,408151366	6,346368286	2,10E-07	6,08E-06	up	EEA1	ENSG00000102189
311	261	246	229	-0,40827502	4,785918422	9,73E-05	0,001337449	down	COL1A1	ENSG00000108821
239	196	180	181	-0,408320621	4,393323693	0,000523035	0,005469501	down	GDAP1	ENSG00000104381
180	154	264	225	0,408480548	4,434848129	0,00066693	0,00668178	up	CCDC91	ENSG00000123106
912	787	707	704	-0,408931901	6,353210829	2,16E-07	6,22E-06	down	UBE2R2	ENSG00000107341
242	176	299	311	0,408972617	4,751917223	0,000382001	0,004207163	up	ZGRF1	ENSG00000138658
236	200	319	319	0,409072536	4,815810239	0,000122516	0,001617819	up	MED28	ENSG00000118579
2524	2104	3486	3290	0,409616519	8,209404219	1,60E-09	6,81E-08	up	NUDT21	ENSG00000167005
376	353	554	516	0,40981967	5,555581462	1,41E-05	0,000250066	up	INSYN2B	ENSG00000204767
360	348	298	291	-0,410049055	5,095685887	0,000148858	0,001899441	down	ODR4	ENSG00000157181
341	281	281	235	-0,410185181	4,90477401	9,35E-05	0,001304041	down	ZNF672	ENSG00000171161
827	652	593	630	-0,410500011	6,148905235	2,68E-06	5,75E-05	down	PRDX5	ENSG00000126432

4478	3735	6157	5875	0,410639885	9,036576037	5,22E-10	2,49E-08	up	NQO1	ENSG00000181019
4292	3228	3338	2878	-0,41070531	8,490555	1,26E-07	3,84E-06	down	QSOX1	ENSG00000116260
280	233	210	215	-0,410914417	4,628609975	0,000244701	0,002883131	down	PLEKHA3	ENSG00000116095
1592	1415	2415	2011	0,411159341	7,592959507	5,12E-07	1,32E-05	up	GCLM	ENSG00000023909
453	354	643	540	0,411955449	5,69691544	9,01E-06	0,000169759	up	TOP1MT	ENSG00000184428
331	202	237	201	-0,412044814	4,673536781	0,004758175	0,032359724	down	TSKU	ENSG00000182704
174	182	149	147	-0,412168636	4,110522266	0,004327398	0,030107598	down	PLEKHH1	ENSG00000054690
290	263	211	247	-0,412575831	4,737741626	0,001084815	0,009892014	down	KIAA1217	ENSG00000120549
214	160	280	268	0,412661762	4,596288011	0,000277978	0,003216632	up	POLR3K	ENSG00000161980
708	582	939	952	0,413021698	6,372596653	2,11E-07	6,09E-06	up	SEMA3C	ENSG00000075223
123	103	102	85	-0,413321469	3,458499844	0,008110218	0,049623159	down	AGA	ENSG00000038002
389	359	316	304	-0,413612185	5,171554653	4,11E-05	0,000640681	down	HCLS1	ENSG00000180353
1910	1550	2604	2471	0,413740797	7,791412372	2,80E-09	1,14E-07	up	RALBP1	ENSG00000017797
160	142	136	114	-0,413809409	3,871053403	0,002921008	0,021945007	down	DDX59	ENSG00000118197
433	377	322	348	-0,413921141	5,284341283	4,24E-05	0,000656849	down	FAM3C	ENSG00000196937
237	214	343	320	0,413993251	4,868347253	0,000101645	0,001380962	up	ZNF518B	ENSG00000178163
192	154	265	243	0,41403572	4,487347933	0,000284588	0,003276037	up	EIF2AK3	ENSG00000172071
260	213	367	328	0,414727604	4,934776138	5,51E-05	0,000827168	up	CSF1	ENSG00000184371
240	205	159	208	-0,415118818	4,423238089	0,005373267	0,035552388	down	SLC14A1	ENSG00000141469
3952	3221	3012	2909	-0,415352745	8,425338165	9,96E-10	4,44E-08	down	NEDD4L	ENSG00000049759
442	366	621	567	0,415619059	5,702661342	1,22E-06	2,85E-05	up	STK17A	ENSG00000164543
256	216	211	179	-0,416091067	4,5070978	0,000311691	0,003543376	down	DHRS7	ENSG00000100612
320	229	229	223	-0,416214288	4,719654907	0,000371202	0,004108627	down	STRADB	ENSG00000082146
2076	1655	2735	2739	0,416263743	7,900412374	1,16E-08	4,25E-07	up	MET	ENSG00000105976
136	129	104	115	-0,416344423	3,685228842	0,006915499	0,043850872	down	SLC41A2	ENSG00000136052
405	365	564	570	0,416730365	5,636927839	7,72E-06	0,000147796	up	CDYL	ENSG00000153046
218	171	288	284	0,417203158	4,656012157	0,000167882	0,002092331	up	KCTD7	ENSG00000243335
453	332	295	349	-0,41736725	5,231591502	0,00057595	0,005909238	down	DTX3L	ENSG00000163840
3807	3463	3391	2650	-0,417387691	8,450705802	6,15E-06	0,000121864	down	NFIC	ENSG00000141905
1152	1320	1063	1004	-0,417987077	6,905809408	0,000599914	0,006128985	down	MTATP6P1	ENSG00000248527
113	90	160	139	0,418211309	3,73102683	0,003569837	0,025712431	up	EEF1A2	ENSG00000101210
253	212	320	364	0,418527268	4,912912181	0,000408807	0,004442526	up	ALDH6A1	ENSG00000119711

274	304	236	244	-0,419173703	4,805237982	0,002789072	0,021168865	down	FBLIM1	ENSG00000162458
355	280	281	242	-0,41932158	4,929348006	5,60E-05	0,000838735	down	GALNS	ENSG00000141012
207	166	148	159	-0,419413041	4,168255067	0,001159384	0,010423664	down	TTC33	ENSG00000113638
154	144	235	205	0,419439574	4,280131309	0,000949352	0,008953342	up	ZNF777	ENSG00000196453
241	151	172	149	-0,419823855	4,232749797	0,005198345	0,03467573	down	EGFL7	ENSG00000172889
3301	2885	4673	4466	0,420027547	8,63629959	1,51E-09	6,45E-08	up	ATAD2	ENSG00000156802
6873	6259	5645	5216	-0,420624774	9,302745275	8,78E-09	3,27E-07	down	PRRC2C	ENSG00000117523
960	775	1308	1249	0,42069822	6,80214317	2,77E-08	9,33E-07	up	AKR1B1	ENSG00000085662
335	256	261	225	-0,420800368	4,824930283	0,000101329	0,001377879	down	SEMA6B	ENSG00000167680
122	98	96	85	-0,420913747	3,416620216	0,006563869	0,042033124	down	RUFY3	ENSG00000018189
612	489	488	418	-0,420995425	5,719815687	2,01E-06	4,43E-05	down	NECTIN1	ENSG00000110400
250	181	183	171	-0,421006562	4,371869884	0,000681055	0,00679262	down	MELTF	ENSG00000163975
129	104	190	154	0,421080159	3,928492678	0,002980987	0,022288918	up	RP9	ENSG00000164610
249	213	350	332	0,421164665	4,905824226	4,20E-05	0,000653002	up	MTIF3	ENSG00000122033
159	123	133	99	-0,42126682	3,768159549	0,005332938	0,035360884	down	GTF2H5	ENSG00000272047
495	430	689	677	0,421402941	5,901697647	7,15E-07	1,76E-05	up	MAT2B	ENSG00000038274
366	287	493	470	0,421591024	5,399397326	3,62E-06	7,61E-05	up	MAD2L2	ENSG00000116670
1373	1177	1083	1017	-0,421865364	6,932969111	1,77E-08	6,23E-07	down	ANXA3	ENSG00000138772
942	710	1243	1190	0,422613251	6,729764412	5,21E-08	1,67E-06	up	APMAP	ENSG00000101474
935	767	726	673	-0,422675837	6,347536766	2,66E-08	9,01E-07	down	RAB5A	ENSG00000144566
424	327	545	562	0,422793781	5,599746648	5,47E-06	0,000110336	up	PPARA	ENSG00000186951
1245	934	1720	1492	0,423012497	7,127429157	1,51E-07	4,52E-06	up	PPP3R1	ENSG00000221823
2638	2033	3617	3269	0,423074498	8,225913955	3,79E-09	1,51E-07	up	COLGALT1	ENSG00000130309
199	159	147	147	-0,423085999	4,107996957	0,000957584	0,009000401	down	MOB3C	ENSG00000142961
688	554	490	528	-0,424119267	5,892308537	3,28E-06	6,99E-05	down	LRRC8C	ENSG00000171488
142	115	194	186	0,424180643	4,069787653	0,001021232	0,009406119	up	ITGB2	ENSG00000160255
585	423	425	400	-0,424223418	5,588047646	8,81E-06	0,00016646	down	DMXL2	ENSG00000104093
675	526	515	470	-0,424283857	5,842683329	6,90E-07	1,71E-05	down	CDKN2C	ENSG00000123080
250	204	170	202	-0,424693379	4,446909387	0,001321838	0,011643124	down	CAMK4	ENSG00000152495
121	92	164	151	0,42476109	3,80254091	0,002344571	0,018478651	up	HLA-B	ENSG00000223532
3866	3283	2993	2880	-0,424807687	8,418905844	5,04E-10	2,42E-08	down	VDAC1	ENSG00000213585
507	415	386	370	-0,425868112	5,463371689	1,71E-06	3,84E-05	down	MOV10	ENSG00000155363

1310	1033	1779	1684	0,425958576	7,236022782	6,16E-09	2,40E-07	up	HIPK1	ENSG00000163349
349	303	496	470	0,426006622	5,402400217	3,34E-06	7,10E-05	up	TERF1	ENSG00000147601
298	263	219	241	-0,426665142	4,751368921	0,000242546	0,002862076	down	MSMO1	ENSG00000052802
3191	2546	4500	3993	0,426787138	8,526140846	1,05E-09	4,64E-08	up	HMGN2	ENSG00000198830
146	131	224	187	0,426837274	4,179607295	0,001213109	0,010814219	up	AC006001.3	ENSG00000229180
189	154	255	253	0,426903818	4,48274674	0,000219306	0,002619689	up	METTL4	ENSG00000101574
172	177	146	141	-0,427342793	4,074908089	0,002754886	0,020960623	down	TNFSF12	ENSG00000239697
1077	889	1493	1420	0,427443428	6,986770215	7,82E-09	2,96E-07	up	NOL11	ENSG00000130935
446	366	618	586	0,428436973	5,716969434	5,11E-07	1,32E-05	up	RPAIN	ENSG00000129197
216	184	280	313	0,428946976	4,70417501	0,000402898	0,004391918	up	BACE2	ENSG00000182240
440	356	623	558	0,429049113	5,68830351	7,74E-07	1,90E-05	up	FZD7	ENSG00000155760
135	93	193	145	0,429435603	3,89984289	0,006132065	0,03964398	up	IRX3	ENSG00000177508
468	399	377	333	-0,429439533	5,374419817	2,58E-06	5,57E-05	down	PCNX2	ENSG00000135749
158	127	114	119	-0,429471012	3,78038328	0,002744246	0,020910404	down	CTPS2	ENSG00000047230
947	797	751	676	-0,430624958	6,380239658	1,67E-08	5,87E-07	down	SEC24A	ENSG00000113615
374	357	285	314	-0,430715898	5,132401969	0,000170985	0,002125886	down	HIBADH	ENSG00000106049
958	810	782	665	-0,431330203	6,399827536	6,49E-08	2,07E-06	down	LRP8	ENSG00000157193
721	660	572	559	-0,431653439	6,046869477	6,07E-07	1,54E-05	down	INPP4B	ENSG00000109452
382	282	504	481	0,431776426	5,427485394	3,93E-06	8,23E-05	up	TBC1D8	ENSG00000204634
4490	3160	3424	2794	-0,431802338	8,502059785	4,11E-06	8,58E-05	down	AGRN	ENSG00000188157
439	405	613	644	0,432299946	5,778697874	7,63E-06	0,000146422	up	FHL1	ENSG00000022267
264	221	234	163	-0,432392592	4,539239384	0,001658675	0,014067484	down	TICAM1	ENSG00000127666
4043	3174	5698	5023	0,432668803	8,859176685	1,03E-09	4,56E-08	up	OGFRL1	ENSG00000119900
179	159	139	137	-0,432868345	4,023349659	0,000980944	0,009116142	down	MITF	ENSG00000187098
351	304	495	480	0,433019557	5,413044995	2,42E-06	5,26E-05	up	RAB12	ENSG00000206418
224	215	179	180	-0,433111952	4,398882773	0,000499472	0,00527999	down	STPG1	ENSG00000001460
520	458	413	386	-0,433463402	5,547152608	1,05E-06	2,51E-05	down	LGALS3	ENSG00000131981
249	180	318	319	0,433528122	4,803340159	1,00E-04	0,001365652	up	IPPK	ENSG00000127080
3275	2430	4303	4148	0,433882859	8,517769231	8,33E-09	3,13E-07	up	FSTL1	ENSG00000163430
257	183	348	306	0,434165974	4,839500143	9,53E-05	0,001320479	up	BAHCC1	ENSG00000266074
1544	1178	2098	1945	0,434480307	7,454872535	4,79E-09	1,90E-07	up	EDC4	ENSG00000038358
535	428	422	363	-0,434589428	5,521253698	1,55E-06	3,51E-05	down	ARSJ	ENSG00000180801

786	555	1072	918	0,434699819	6,43423353	1,66E-06	3,74E-05	up	G6PD	ENSG00000160211
400	347	302	307	-0,435179152	5,158380315	1,02E-05	0,000188422	down	CPT2	ENSG00000157184
474	381	647	626	0,435217302	5,794304665	3,48E-07	9,41E-06	up	MALT1	ENSG00000172175
330	296	293	219	-0,435323901	4,905733263	0,000306799	0,003500569	down	HDAC10	ENSG00000100429
89	72	121	119	0,43570249	3,413392919	0,005525516	0,036404642	up	C9orf85	ENSG00000155621
242	215	316	365	0,435713231	4,899700117	0,000438172	0,004720541	up	ITPR1	ENSG00000150995
511	400	685	671	0,435836497	5,884782747	2,74E-07	7,65E-06	up	WDR54	ENSG00000005448
511	389	359	372	-0,43632261	5,421752094	7,17E-06	0,000138522	down	MEAK7	ENSG00000140950
160	112	122	99	-0,436772761	3,708388854	0,004722005	0,032200774	down	GRAMD1B	ENSG00000023171
657	526	878	884	0,436777295	6,26099517	6,52E-08	2,07E-06	up	PHF6	ENSG00000156531
422	328	326	284	-0,436946691	5,160130762	1,01E-05	0,000186916	down	PLXNA2	ENSG00000076356
289	217	205	206	-0,437201027	4,595014961	0,000174378	0,002157701	down	SLC9A8	ENSG00000197818
162	93	193	185	0,437537153	4,058431839	0,004732907	0,032249811	up	SLC25A10	ENSG00000183048
1182	892	914	770	-0,437816589	6,621977105	1,95E-07	5,69E-06	down	MAPKAPK3	ENSG00000114738
115	86	156	144	0,438140803	3,727934778	0,002333008	0,018409187	up	ZNF324	ENSG00000083812
10052	7352	7541	6551	-0,438272087	9,687339166	3,54E-08	1,17E-06	down	FLNC	ENSG00000128591
709	566	509	526	-0,438406735	5,923564611	4,37E-07	1,15E-05	down	FBN1	ENSG00000166147
930	796	707	697	-0,438643376	6,362635667	1,66E-08	5,86E-07	down	KATNAL1	ENSG00000102781
299	239	195	241	-0,438764122	4,683030856	0,000969989	0,009041396	down	DCAKD	ENSG00000172992
905	827	762	651	-0,43957114	6,370112453	2,32E-07	6,62E-06	down	APBB1IP	ENSG00000077420
109	89	154	142	0,439642648	3,708481958	0,002213576	0,017646108	up	POP5	ENSG00000167272
200	195	292	300	0,440536244	4,696266472	0,000266705	0,00310469	up	ZNF473	ENSG00000142528
1527	1168	1050	1128	-0,440702253	6,998810266	3,14E-07	8,67E-06	down	CD59	ENSG00000085063
132	89	170	160	0,440746387	3,862321152	0,002075383	0,01681201	up	MCAT	ENSG00000100294
604	518	492	420	-0,441186452	5,740684101	6,66E-07	1,66E-05	down	PRKCD	ENSG00000163932
170	114	223	201	0,441261208	4,218466408	0,00075625	0,007400003	up	ZNF3	ENSG00000166526
402	346	576	544	0,441322422	5,608099436	5,00E-07	1,29E-05	up	FAM204A	ENSG00000165669
469	415	360	358	-0,441447998	5,39844899	2,38E-06	5,19E-05	down	TRIM6	ENSG00000121236
109	98	89	79	-0,441586776	3,322589583	0,006025497	0,039117869	down	ZNF253	ENSG00000256771
378	307	516	509	0,442198868	5,48090582	9,90E-07	2,39E-05	up	ZWILCH	ENSG00000174442
3960	3533	5825	5432	0,442300387	8,926720469	3,74E-10	1,86E-08	up	HNRNPF	ENSG00000169813
404	307	318	258	-0,44244258	5,080293424	3,64E-05	0,00057764	down	KLHL29	ENSG00000119771

1181	909	904	788	-0,442579288	6,631973834	2,70E-08	9,12E-07	down	CARS2	ENSG00000134905
132	103	186	166	0,44282656	3,952923893	0,001013044	0,009346157	up	SNX29	ENSG00000048471
1111	925	856	795	-0,442840402	6,597763571	3,38E-09	1,36E-07	down	EPN2	ENSG00000072134
177	135	234	233	0,443085763	4,35600574	0,000241521	0,002854318	up	FAM76B	ENSG00000077458
168	139	233	227	0,443279645	4,334429209	0,000240065	0,002843607	up	SIAH1	ENSG00000196470
4001	3338	5570	5426	0,443506765	8,893324131	4,87E-11	2,74E-09	up	EGFR	ENSG00000146648
921	817	788	626	-0,44361389	6,372159611	9,04E-07	2,19E-05	down	CUX1	ENSG00000257923
225	180	180	148	-0,444080067	4,274745206	0,000328266	0,003691266	down	ZNF385A	ENSG00000161642
384	316	303	264	-0,444295067	5,05938166	7,67E-06	0,000146975	down	FAM214B	ENSG00000005238
331	215	439	376	0,444511188	5,15041724	0,000153679	0,001949742	up	RHPN2	ENSG00000131941
269	241	191	222	-0,444627	4,607185145	0,000510408	0,005373649	down	LTBP1	ENSG00000049323
330	312	271	250	-0,445079711	4,938720118	3,86E-05	0,000607356	down	INPP1	ENSG00000151689
208	168	146	158	-0,445191822	4,168505943	0,000576121	0,005909238	down	ZNF605	ENSG00000196458
785	701	1234	1006	0,445931972	6,598763696	4,83E-07	1,26E-05	up	SKI	ENSG00000157933
1798	1467	1336	1302	-0,446346796	7,276526273	8,46E-10	3,85E-08	down	ARHGEF28	ENSG00000214944
1480	1253	1189	1025	-0,446462529	7,021694869	7,24E-09	2,77E-07	down	RHOBTB3	ENSG00000164292
760	638	591	540	-0,446567949	6,054466719	2,83E-08	9,52E-07	down	CCBE1	ENSG00000183287
255	220	184	200	-0,446572602	4,50349991	0,000183322	0,00225544	down	DNMT3A	ENSG00000119772
817	789	1239	1185	0,447034627	6,714570654	1,65E-07	4,89E-06	up	SEC22B	ENSG00000265808
402	346	559	565	0,447156725	5,611607238	8,29E-07	2,02E-05	up	SH3PXD2A	ENSG00000107957
244	198	182	175	-0,447473356	4,398702997	0,000124182	0,001636661	down	NIPSNAP2	ENSG00000146729
203	167	162	137	-0,447485983	4,144669889	0,000450491	0,004831266	down	BCL2L11	ENSG00000153094
251	226	336	381	0,447603289	4,96839856	0,000184243	0,002261751	up	KCNN4	ENSG00000104783
153	133	212	218	0,44787491	4,23693853	0,000399579	0,004363616	up	RHOT1	ENSG00000126858
466	334	327	317	-0,44790204	5,245515725	1,59E-05	0,000281581	down	DNPEP	ENSG00000123992
1265	1005	965	867	-0,448079627	6,750189866	6,35E-09	2,46E-07	down	NAV1	ENSG00000134369
650	521	483	462	-0,448108412	5,796981857	1,39E-07	4,19E-06	down	SYNPO	ENSG00000171992
563	517	814	813	0,448168865	6,141994904	2,41E-07	6,86E-06	up	MTIF2	ENSG00000085760
144	126	105	113	-0,448443123	3,696391355	0,002537155	0,019680058	down	GPR161	ENSG00000143147
491	466	741	703	0,448686538	5,969751176	6,44E-07	1,61E-05	up	AZI2	ENSG00000163512
172	121	127	109	-0,449249083	3,80877365	0,002225336	0,017703507	down	IL1RAP	ENSG00000196083
233	174	177	151	-0,449641223	4,278056493	0,000337003	0,003778566	down	SLC35F3	ENSG00000183780

203	151	138	147	-0,449697928	4,078910353	0,000853043	0,008192271	down	SLC22A5	ENSG00000197375
379	280	511	479	0,44972385	5,427186556	1,43E-06	3,27E-05	up	TCAIM	ENSG00000179152
391	313	312	256	-0,449793288	5,064389569	1,28E-05	0,000229563	down	GPSM1	ENSG00000160360
1740	1470	1391	1203	-0,449982723	7,252212559	2,58E-09	1,06E-07	down	PVR	ENSG00000073008
187	155	156	120	-0,450215333	4,031352761	0,001042207	0,009565263	down	SPATA33	ENSG00000167523
606	496	440	448	-0,45028003	5,709401867	3,27E-07	8,97E-06	down	TRIM5	ENSG00000132256
380	341	515	571	0,45032407	5,562131579	1,66E-05	0,000292774	up	RBM27	ENSG00000091009
182	137	140	117	-0,450484449	3,930407538	0,001123027	0,010180671	down	KITLG	ENSG00000049130
254	174	189	155	-0,450757237	4,347164953	0,000915943	0,008683562	down	NAGLU	ENSG00000108784
504	415	394	347	-0,450922638	5,44779759	5,84E-07	1,49E-05	down	LITAF	ENSG00000189067
550	451	475	334	-0,451159283	5,570932529	0,000133211	0,001731074	down	ST6GALNAC4	ENSG00000136840
2010	1467	1443	1346	-0,451324756	7,359082619	2,08E-08	7,21E-07	down	RHOC	ENSG00000155366
333	301	233	278	-0,451562232	4,916827433	0,000279643	0,003228678	down	RAP1GAP2	ENSG00000132359
2845	2519	2181	2150	-0,451735309	7,995250698	9,33E-10	4,23E-08	down	CEP250	ENSG00000126001
106	72	141	127	0,451736894	3,562580416	0,00355235	0,025613395	up	ANKS3	ENSG00000168096
241	210	164	199	-0,452114059	4,426982311	0,000856218	0,008207534	down	ILDR2	ENSG00000143195
314	315	476	476	0,452334281	5,371147884	2,50E-05	0,00041926	up	DNAJC2	ENSG00000105821
240	251	382	362	0,452357729	5,01724495	0,000138707	0,001789022	up	GTF2E2	ENSG00000197265
573	466	395	440	-0,452580898	5,623351337	4,73E-06	9,71E-05	down	CD47	ENSG00000196776
413	372	326	307	-0,452697935	5,223089559	3,33E-06	7,09E-05	down	CSNK1G1	ENSG00000169118
484	387	678	635	0,452854864	5,831017126	6,81E-08	2,16E-06	up	PCGF3	ENSG00000185619
1117	841	1468	1474	0,452979109	6,991273759	1,82E-08	6,36E-07	up	MCM6	ENSG00000076003
190	134	136	124	-0,45440859	3,949726347	0,001182578	0,010578486	down	LSS	ENSG00000160285
287	276	480	375	0,454574463	5,212968319	0,000129828	0,001695627	up	CRLS1	ENSG00000088766
4439	3413	3320	2978	-0,454694467	8,535400724	2,69E-10	1,36E-08	down	LGALS3BP	ENSG00000108679
168	165	250	254	0,454800395	4,460583587	0,000352791	0,003927229	up	HMGN3	ENSG00000118418
186	140	252	240	0,454865042	4,425268277	0,000110823	0,001481048	up	ILKAP	ENSG00000132323
359	365	564	535	0,454918491	5,575255694	1,24E-05	0,000223462	up	GGCT	ENSG00000006625
200	157	275	264	0,454964519	4,555604786	5,72E-05	0,000851857	up	GALNT11	ENSG00000178234
903	695	1255	1155	0,455195743	6,701918577	2,04E-09	8,45E-08	up	POLR2C	ENSG00000102978
349	286	261	249	-0,455819535	4,914472496	6,93E-06	0,000134765	down	SCML1	ENSG00000047634
400	306	557	509	0,455877004	5,530453747	3,56E-07	9,56E-06	up	UBL4A	ENSG00000102178

335	281	271	224	-0,456655731	4,870950964	1,91E-05	0,000331709	down	RTL8C	ENSG00000134590
187	168	253	284	0,456706677	4,551179268	0,000405246	0,004413085	up	MBP	ENSG00000197971
162	123	223	208	0,457355855	4,235434028	0,000206971	0,002501208	up	MTHFSD	ENSG00000103248
1283	1066	1795	1758	0,457443219	7,26054313	4,42E-10	2,19E-08	up	ADD1	ENSG00000087274
263	209	361	353	0,457942076	4,956471427	6,90E-06	0,000134413	up	STXBP4	ENSG00000166263
445	382	653	600	0,458028549	5,761850848	9,25E-08	2,89E-06	up	FKBP5	ENSG00000096060
150	141	214	227	0,458081599	4,269113948	0,000500998	0,00529252	up	RPGRIP1L	ENSG00000103494
118	93	80	89	-0,458485433	3,340983602	0,005450054	0,035953227	down	SQOR	ENSG00000137767
3055	2526	2196	2272	-0,459002358	8,04538971	9,58E-10	4,31E-08	down	NRP1	ENSG00000099250
151	117	221	185	0,459013083	4,149069356	0,00045994	0,00491563	up	ZNF205	ENSG00000122386
1142	916	821	826	-0,459058718	6,604674298	3,60E-09	1,44E-07	down	FADS3	ENSG00000221968
293	211	390	372	0,459328806	5,048613617	8,59E-06	0,000162489	up	MED6	ENSG00000133997
1709	1377	1282	1189	-0,459559877	7,188852378	2,67E-10	1,36E-08	down	GALNT2	ENSG00000143641
133	91	178	161	0,459591692	3,892761841	0,00104392	0,009575326	up	HAUS4	ENSG00000092036
457	370	341	321	-0,460415193	5,291621089	6,07E-07	1,54E-05	down	LPIN1	ENSG00000134324
4140	3489	5910	5668	0,461049671	8,959961586	4,79E-12	3,25E-10	up	CCND1	ENSG00000110092
1326	1011	988	878	-0,461636194	6,78431082	7,85E-09	2,96E-07	down	LRP5	ENSG00000162337
90	80	118	140	0,461873623	3,506441307	0,006770562	0,043143232	up	EFCAB11	ENSG00000140025
2151	1842	1644	1554	-0,462124327	7,562801022	7,96E-11	4,37E-09	down	EFCAB14	ENSG00000159658
93	62	119	116	0,462183312	3,373075933	0,004945209	0,033259326	up	GJA3	ENSG00000121743
45139	61083	40530	46163	-0,462245525	12,32852311	0,0075568	0,0469774	down	MT-ND4	ENSG00000198886
508	336	696	579	0,462306914	5,783923772	2,25E-05	0,000382809	up	APRT	ENSG00000198931
2994	2140	4058	3695	0,462686281	8,380325691	1,11E-08	4,07E-07	up	UHRF1	ENSG00000276043
222	171	171	143	-0,46280403	4,222934641	0,000248525	0,002925968	down	TACSTD2	ENSG00000184292
1403	1091	1912	1867	0,463201307	7,346733075	4,98E-10	2,40E-08	up	STK10	ENSG00000072786
403	278	556	476	0,464205524	5,480055302	7,55E-06	0,000145259	up	DUSP5	ENSG00000138166
4007	2885	2872	2603	-0,464264182	8,339211611	9,54E-09	3,53E-07	down	UHMK1	ENSG00000152332
415	359	613	565	0,464438243	5,670798864	9,94E-08	3,08E-06	up	GRPEL2	ENSG00000164284
13866	12232	10767	10134	-0,464696661	10,27307796	4,94E-12	3,33E-10	down	CCN1	ENSG00000142871
1300	1050	1895	1680	0,465244928	7,264300716	3,36E-10	1,68E-08	up	CSNK2A2	ENSG00000070770
130	91	90	86	-0,465584232	3,401852387	0,004837271	0,032773991	down	DNPH1	ENSG00000112667
535	422	381	381	-0,466698389	5,498098817	3,00E-07	8,33E-06	down	UNC119B	ENSG00000175970

126	116	93	100	-0,466879606	3,533544248	0,002887626	0,021757371	down	TRAF5	ENSG00000082512
396	310	576	499	0,466914188	5,53750897	3,51E-07	9,46E-06	up	NCKAP5L	ENSG00000167566
19743	23392	16877	18031	-0,466954743	11,01718909	0,000513776	0,005393334	down	MT-CYB	ENSG00000198727
267	206	369	351	0,467256308	4,964330531	4,23E-06	8,81E-05	up	SAT2	ENSG00000141504
471	433	366	356	-0,467281994	5,420710435	1,10E-06	2,60E-05	down	PTGR1	ENSG00000106853
688	574	487	518	-0,46731641	5,897971309	1,58E-07	4,71E-06	down	MAP3K9	ENSG00000006432
94	63	127	112	0,467489686	3,394360334	0,004022254	0,028404538	up	GALM	ENSG00000143891
1560	1256	2173	2112	0,46754944	7,525742779	7,15E-11	3,95E-09	up	PEBP1	ENSG00000089220
1947	1782	1560	1424	-0,467786921	7,465347408	1,71E-09	7,21E-08	down	GNL3	ENSG00000163938
505	416	354	379	-0,467878619	5,443808798	1,13E-06	2,67E-05	down	CRYZ	ENSG00000116791
134	82	168	160	0,468017954	3,843526011	0,002288874	0,018116167	up	TTC39C	ENSG00000168234
197	167	152	138	-0,468048725	4,112940425	0,000206757	0,002500572	down	DNAJB5	ENSG00000137094
213	192	177	146	-0,468654629	4,266427574	0,000192187	0,002348133	down	SARDH	ENSG00000123453
1297	1005	920	908	-0,468747006	6,760377403	3,04E-09	1,24E-07	down	PAM	ENSG00000145730
5004	4055	3627	3574	-0,468946565	8,738933553	9,13E-12	5,91E-10	down	PTPN14	ENSG00000152104
385	368	546	605	0,46895652	5,63780987	1,67E-05	0,000293659	up	CYP1B1	ENSG00000138061
314	249	239	209	-0,469009653	4,735241329	1,56E-05	0,000275236	down	SLC46A3	ENSG00000139508
1597	1525	1316	1184	-0,469436661	7,210460404	3,29E-08	1,10E-06	down	PLBD2	ENSG00000151176
474	402	342	355	-0,469526884	5,371940951	6,60E-07	1,65E-05	down	CERCAM	ENSG00000167123
193	113	242	222	0,469914293	4,336886993	0,001191411	0,010651361	up	ARTN	ENSG00000117407
153	125	107	114	-0,469957513	3,727615665	0,001131094	0,010218009	down	GAD1	ENSG00000128683
328	245	406	465	0,470366988	5,238383718	5,16E-05	0,000781881	up	HERC5	ENSG00000138646
101	77	153	119	0,471034501	3,575293301	0,003362234	0,024512737	up	ZSCAN22	ENSG00000182318
182	189	146	150	-0,47138084	4,143485857	0,00117509	0,010535782	down	C1orf21	ENSG00000116667
234	169	327	288	0,471625614	4,736518589	3,13E-05	0,000508566	up	R3HCC1	ENSG00000104679
1160	941	851	817	-0,471823446	6,629516317	3,02E-10	1,52E-08	down	GAA	ENSG00000171298
237	199	163	183	-0,472433471	4,369019416	0,000159917	0,00201244	down	PAQR5	ENSG00000137819
310	226	396	421	0,472918695	5,144402512	1,19E-05	0,000216414	up	MPP2	ENSG00000108852
833	696	1175	1163	0,473010591	6,652428864	5,60E-10	2,65E-08	up	PRPF38A	ENSG00000134748
72	62	97	108	0,473074605	3,176891677	0,007334873	0,045854767	up	TSPAN12	ENSG00000106025
953	843	1306	1442	0,473193251	6,886624993	2,28E-07	6,51E-06	up	IFIT2	ENSG00000119922
178	162	134	136	-0,473430548	4,014580063	0,00039483	0,004323914	down	PPP1R13B	ENSG00000088808

115	96	171	152	0,473475759	3,818781724	0,000692953	0,006897995	up	C8orf82	ENSG00000213563
1266	1084	961	906	-0,473523426	6,792834371	4,56E-10	2,24E-08	down	SLC41A1	ENSG00000133065
1407	1165	1074	968	-0,473621685	6,921377164	2,52E-10	1,28E-08	down	MARCH4	ENSG00000144583
90	83	131	134	0,474008704	3,538837807	0,002124877	0,017088146	up	RAB3IL1	ENSG00000167994
567	510	450	406	-0,474511326	5,669024662	1,18E-07	3,60E-06	down	TENM3	ENSG00000218336
234	185	176	156	-0,475030129	4,309733615	8,01E-05	0,001152083	down	SNTB1	ENSG00000172164
2384	2293	2020	1717	-0,475038684	7,792454995	8,16E-08	2,57E-06	down	NR2F2	ENSG00000185551
331	307	522	458	0,475199597	5,402158095	1,33E-06	3,06E-05	up	DCLRE1A	ENSG00000198924
76	50	100	93	0,476245625	3,089825746	0,00802661	0,049225734	up	ZNF891	ENSG00000214029
178	122	127	110	-0,476321819	3,830056247	0,001372692	0,012029646	down	RAC3	ENSG00000169750
1543	1216	1179	1005	-0,476335555	7,018964838	1,69E-09	7,13E-08	down	RUSC2	ENSG00000198853
1002	755	1382	1304	0,476406581	6,849523758	1,30E-09	5,66E-08	up	RMND5A	ENSG00000153561
147	162	128	118	-0,477305093	3,881280236	0,002642398	0,020333896	down	LACC1	ENSG00000179630
608	508	893	820	0,477436241	6,202602544	1,24E-09	5,42E-08	up	STAG1	ENSG00000118007
1368	1102	1921	1867	0,478728424	7,343706532	4,69E-11	2,66E-09	up	SORT1	ENSG00000134243
358	255	482	457	0,47898434	5,33971579	1,02E-06	2,44E-05	up	MAPKAPK5	ENSG00000089022
384	297	529	516	0,479576177	5,493132601	1,20E-07	3,65E-06	up	PHLPP2	ENSG00000040199
930	679	1315	1150	0,479577268	6,723526582	7,34E-09	2,80E-07	up	HASPIN	ENSG00000177602
354	312	287	240	-0,480194238	4,974168874	5,09E-06	0,000103566	down	ST5	ENSG00000166444
453	446	360	353	-0,480564955	5,409677979	4,75E-06	9,73E-05	down	LMLN	ENSG00000185621
168	147	260	225	0,480919022	4,394155564	7,00E-05	0,001022317	up	SPATS2	ENSG00000123352
7180	5569	5273	4770	-0,480968438	9,224101216	4,77E-12	3,25E-10	down	ARHGAP29	ENSG00000137962
1898	1594	1412	1346	-0,481017216	7,360240741	1,90E-11	1,17E-09	down	ARPC5	ENSG00000162704
698	588	538	478	-0,481164378	5,919339398	6,66E-09	2,57E-07	down	VEGFA	ENSG00000112715
104	113	158	177	0,481285933	3,86825804	0,003336046	0,024361302	up	EBAG9	ENSG00000147654
484	383	693	640	0,48142889	5,84087274	1,16E-08	4,25E-07	up	MED29	ENSG00000063322
278	255	202	219	-0,481813404	4,654900735	5,65E-05	0,000844499	down	SIM2	ENSG00000159263
828	669	629	552	-0,481950751	6,136326302	2,73E-09	1,12E-07	down	TNS1	ENSG00000079308
4306	4956	3962	3463	-0,482773384	8,787592987	9,54E-05	0,001320479	down	MT-ND3	ENSG00000198840
1145	1030	865	853	-0,483526624	6,679289262	1,09E-09	4,83E-08	down	IL13RA1	ENSG00000131724
458	396	675	642	0,48372918	5,823348632	1,37E-08	4,93E-07	up	E2F8	ENSG00000129173
95	95	83	67	-0,484071261	3,184784803	0,006952735	0,043997156	down	GPR3	ENSG00000181773

578	454	415	397	-0,484110006	5,598966879	4,28E-08	1,39E-06	down	ATP8B1	ENSG00000081923
254	239	192	197	-0,484228916	4,542507521	6,24E-05	0,000922538	down	PARP8	ENSG00000151883
6285	5310	4636	4501	-0,484420338	9,091209491	5,52E-13	4,42E-11	down	MAP1B	ENSG00000131711
148	135	122	101	-0,485401018	3,747800245	0,000866495	0,008290375	down	PRSS12	ENSG00000164099
75	63	107	106	0,485522137	3,225465116	0,003977549	0,028127215	up	C5	ENSG00000106804
141	116	96	106	-0,486217798	3,609092818	0,001513593	0,013065362	down	TMEM40	ENSG00000088726
104	68	132	133	0,486419387	3,533754943	0,002281452	0,018075849	up	ARL6	ENSG00000113966
1478	993	1934	1850	0,486566746	7,338965568	1,46E-07	4,38E-06	up	TMED9	ENSG00000184840
308	218	201	211	-0,486655291	4,627225022	8,74E-05	0,00123567	down	SGTB	ENSG00000197860
336	237	476	407	0,486992942	5,247452762	3,52E-06	7,43E-05	up	SAR1B	ENSG00000152700
541	383	763	660	0,487231326	5,931377233	2,12E-07	6,11E-06	up	CEBPG	ENSG00000153879
466	387	326	344	-0,487363967	5,325373647	4,75E-07	1,24E-05	down	PLCE1	ENSG00000138193
1199	1000	1730	1668	0,487822979	7,183580117	2,64E-11	1,59E-09	up	EIF4EBP2	ENSG00000148730
890	789	679	642	-0,488680332	6,30276831	7,52E-10	3,46E-08	down	C11orf24	ENSG00000171067
2040	1580	1476	1359	-0,489501507	7,404252889	3,40E-11	1,99E-09	down	CLSTN1	ENSG00000171603
222	160	147	152	-0,489605128	4,169570372	0,000240614	0,002847943	down	PRDM8	ENSG00000152784
132	85	168	167	0,490550386	3,864587162	0,000858182	0,008221293	up	CGRRF1	ENSG00000100532
248	210	385	325	0,490730208	4,934112308	4,88E-06	9,97E-05	up	TIPIN	ENSG00000075131
175	103	223	205	0,490879983	4,213334588	0,000969845	0,009041396	up	ARHGEF19	ENSG00000142632
1008	814	1395	1422	0,4910611	6,913491481	4,67E-10	2,28E-08	up	ITM2C	ENSG00000135916
471	413	727	645	0,491539317	5,877994039	1,23E-08	4,47E-07	up	TRIM41	ENSG00000146063
89	87	71	67	-0,492056283	3,072747147	0,005914043	0,038506989	down	IL1RAPL1	ENSG00000169306
86	74	118	130	0,492058245	3,438244734	0,002403472	0,018819614	up	MTURN	ENSG00000180354
697	677	533	548	-0,492093523	6,016245612	4,60E-07	1,21E-05	down	CASP4	ENSG00000196954
233	182	180	145	-0,492127729	4,288322553	8,83E-05	0,00124633	down	ADAMTSL1	ENSG00000178031
115	112	83	95	-0,492293654	3,43282282	0,003578479	0,025752658	down	PTGFRN	ENSG00000134247
1230	1147	1897	1804	0,492798664	7,30425598	6,77E-10	3,16E-08	up	CBX1	ENSG00000108468
227	198	350	310	0,493344061	4,829277214	3,86E-06	8,12E-05	up	PPCS	ENSG00000127125
210	159	293	279	0,493577643	4,624910448	1,09E-05	0,000200642	up	NSUN5P1	ENSG00000223705
89	72	138	112	0,493716957	3,447430155	0,002157734	0,017298631	up	SLC25A33	ENSG00000171612
163	154	117	131	-0,495547602	3,906004681	0,000681573	0,006793423	down	RRAS	ENSG00000126458
391	342	288	285	-0,496165091	5,104988746	5,28E-07	1,36E-05	down	BMP4	ENSG00000125378

513	360	705	647	0,496167137	5,855109748	9,35E-08	2,92E-06	up	ATG2B	ENSG00000066739
189	163	140	135	-0,496260717	4,053279394	0,000109359	0,001467796	down	RNF144B	ENSG00000137393
693	594	515	491	-0,496397406	5,914695105	1,95E-09	8,12E-08	down	RABGAP1	ENSG00000011454
83	60	104	118	0,496674827	3,280258736	0,004585988	0,031469647	up	ABTB1	ENSG00000114626
477	414	350	346	-0,497155378	5,385154738	7,72E-08	2,44E-06	down	RASEF	ENSG00000165105
133	143	218	213	0,497213288	4,21995223	0,000414878	0,0045022	up	ZNF254	ENSG00000213096
933	820	712	659	-0,497241891	6,360963811	1,90E-10	9,79E-09	down	PTPRJ	ENSG00000149177
2076	1723	2965	2941	0,497391852	7,975838405	3,06E-12	2,17E-10	up	IGFBP4	ENSG00000141753
112	88	86	70	-0,497669436	3,248317503	0,003028873	0,022526521	down	PTGFR	ENSG00000122420
326	239	456	422	0,498314205	5,235483214	3,59E-07	9,62E-06	up	LSM5	ENSG00000106355
128	96	74	100	-0,498497576	3,406694171	0,007709519	0,047716671	down	CAMK1D	ENSG00000183049
817	592	1112	1073	0,498770906	6,544045218	2,06E-09	8,49E-08	up	MYEOV	ENSG00000172927
378	349	568	566	0,498795893	5,603399802	1,18E-07	3,61E-06	up	KAT2B	ENSG00000114166
3548	2730	2532	2350	-0,498821003	8,194145637	3,13E-12	2,21E-10	down	ABL2	ENSG00000143322
1493	1197	2165	2020	0,498916665	7,478071264	2,40E-12	1,76E-10	up	CCAR2	ENSG00000158941
6394	4993	9033	8650	0,498970774	9,554569086	1,90E-13	1,64E-11	up	CAV1	ENSG00000105974
401	357	594	588	0,49942101	5,662484424	3,02E-08	1,01E-06	up	MTF2	ENSG00000143033
431	378	352	280	-0,499766959	5,245464107	1,52E-06	3,44E-05	down	C15orf39	ENSG00000167173
428	319	304	277	-0,499805557	5,126079209	8,11E-07	1,98E-05	down	ATP11C	ENSG00000101974
242	217	185	173	-0,499808173	4,432036631	2,14E-05	0,000365377	down	SMYD3	ENSG00000185420
87	71	59	64	-0,499978577	2,915830378	0,007932893	0,048793747	down	SNN	ENSG00000184602
592	424	434	355	-0,500651652	5,565816528	1,51E-06	3,42E-05	down	SPRY2	ENSG00000136158
427	351	298	307	-0,501816919	5,186566881	3,17E-07	8,72E-06	down	ABCA3	ENSG00000167972
608	475	420	421	-0,502283972	5,660449662	1,97E-08	6,86E-07	down	BHLHE40	ENSG00000134107
551	432	727	803	0,502869426	6,03284838	1,65E-07	4,89E-06	up	POLR1B	ENSG00000125630
520	433	388	353	-0,503344697	5,477932041	1,33E-08	4,79E-07	down	ABCA2	ENSG00000107331
587	523	435	429	-0,503420208	5,699935697	1,57E-08	5,56E-07	down	EDEM3	ENSG00000116406
141	108	211	178	0,503505861	4,070679522	0,000161053	0,002021817	up	SETD6	ENSG00000103037
148	105	218	177	0,503809509	4,09212058	0,000312206	0,003544089	up	MTMR9	ENSG00000104643
100	67	139	122	0,505539683	3,50397149	0,001486973	0,012878527	up	IFIH1	ENSG00000115267
129	91	174	170	0,506510617	3,895520687	0,000250397	0,002943541	up	ANKRD30A	ENSG00000148513
203	157	148	131	-0,506612398	4,079075847	9,68E-05	0,001332282	down	CEP85L	ENSG00000111860

922	715	1422	1141	0,506810939	6,767094505	1,61E-08	5,70E-07	up	E2F1	ENSG00000101412
176	188	154	130	-0,506814938	4,102114089	0,000798455	0,007778676	down	F2R	ENSG00000181104
270	203	389	351	0,506898204	4,98717512	8,42E-07	2,05E-05	up	TDRKH	ENSG00000182134
61	45	83	83	0,506919276	2,866734435	0,007568864	0,047033572	up	AC010422.8	ENSG00000285589
135	102	191	180	0,507005226	4,002529587	0,000121627	0,00160881	up	PDCD2L	ENSG00000126249
339	258	243	219	-0,508019832	4,801616679	2,39E-06	5,20E-05	down	GDPD5	ENSG00000158555
600	458	881	776	0,508877397	6,141307899	1,22E-09	5,37E-08	up	PAN3	ENSG00000152520
107	88	166	140	0,509013751	3,727847907	0,000531092	0,005523993	up	DENND1B	ENSG00000213047
120	97	97	71	-0,509347482	3,359030313	0,00267537	0,020496215	down	ABHD14A	ENSG00000248487
544	438	370	389	-0,509585383	5,517515919	4,29E-08	1,39E-06	down	ARHGAP30	ENSG00000186517
686	595	1049	964	0,509938823	6,42133694	6,63E-11	3,70E-09	up	COPS7A	ENSG00000111652
110	95	173	149	0,510038344	3,799961014	0,000348929	0,003895409	up	SLC6A9	ENSG00000196517
1032	877	761	717	-0,510249928	6,477039803	2,14E-11	1,30E-09	down	GALNT7	ENSG00000109586
394	389	357	254	-0,510290293	5,199315195	0,00012499	0,001642111	down	IKBKG	ENSG00000269335
1157	1105	1803	1764	0,51033823	7,244532842	1,37E-09	5,94E-08	up	ANP32A	ENSG00000140350
112	91	141	177	0,510610434	3,7846502	0,002253569	0,017875882	up	SYTL4	ENSG00000102362
70	63	100	109	0,511193083	3,189302309	0,003937072	0,02786636	up	RELL1	ENSG00000181826
292	251	418	435	0,511561951	5,190572458	4,63E-07	1,22E-05	up	FCHO2	ENSG00000157107
453	332	327	278	-0,512709222	5,191135806	9,97E-07	2,40E-05	down	MROH1	ENSG00000179832
105	88	80	69	-0,512881787	3,192233757	0,002588149	0,019985758	down	ZC3H11A	ENSG00000058673
735	582	536	480	-0,513283053	5,937743705	7,56E-10	3,47E-08	down	GSDME	ENSG00000105928
333	339	544	518	0,513321652	5,502768017	1,02E-06	2,44E-05	up	DAB2	ENSG00000153071
1779	1585	1348	1255	-0,514168762	7,295541894	8,56E-12	5,59E-10	down	FNBP1	ENSG00000187239
416	362	321	280	-0,514202725	5,182695737	1,36E-07	4,11E-06	down	TNRC6C	ENSG00000078687
71	56	105	95	0,514491783	3,125171742	0,002652175	0,020368753	up	ZNF16	ENSG00000170631
1702	1330	1237	1098	-0,514883732	7,138399469	1,20E-11	7,53E-10	down	TGFBR2	ENSG00000163513
87	89	76	60	-0,515464146	3,063790631	0,004957867	0,033314895	down	GPR17	ENSG00000144230
145	104	215	177	0,515565316	4,076743235	0,000195501	0,002377402	up	LSR	ENSG00000105699
307	188	185	193	-0,515577519	4,522874823	0,000578588	0,005930619	down	TMEM206	ENSG00000065600
128	121	92	100	-0,516024697	3,553435789	0,001027922	0,00945092	down	C1orf226	ENSG00000239887
205	187	330	289	0,516374341	4,728485207	4,62E-06	9,51E-05	up	CYFIP2	ENSG00000055163
1756	1375	2666	2267	0,516677611	7,705671799	2,46E-11	1,49E-09	up	BIRC2	ENSG00000110330

1040	767	723	664	-0,516855736	6,388859558	4,99E-10	2,40E-08	down	B4GALT2	ENSG00000117411
161	124	107	112	-0,518148513	3,741497334	0,000324929	0,003667017	down	DDB2	ENSG00000134574
105	111	98	69	-0,519644978	3,353398916	0,005856168	0,038233487	down	ARHGAP40	ENSG00000124143
183	161	285	259	0,519749082	4,543052381	5,77E-06	0,00011549	up	N4BP2	ENSG00000078177
148	109	224	182	0,52006156	4,124825508	0,000139446	0,001797064	up	SCARF2	ENSG00000244486
259	229	183	192	-0,520506549	4,510881376	9,20E-06	0,0001729	down	AP1S3	ENSG00000152056
477	412	731	676	0,520809986	5,902956153	4,55E-10	2,24E-08	up	TLE3	ENSG00000140332
78	76	118	126	0,522096774	3,403548818	0,001929147	0,015867439	up	ABCC2	ENSG00000023839
181	136	231	269	0,522117527	4,424035912	0,000144865	0,001854578	up	ARRB1	ENSG00000137486
176	121	109	118	-0,522256273	3,796004115	0,00074669	0,00731568	down	DNAJB2	ENSG00000135924
118	96	82	82	-0,522949183	3,333765066	0,001165465	0,010461563	down	TMEM53	ENSG00000126106
487	376	348	313	-0,523045015	5,324622393	3,17E-08	1,06E-06	down	FAH	ENSG00000103876
2073	1758	1521	1419	-0,523402971	7,476697631	1,80E-13	1,57E-11	down	MGST1	ENSG00000008394
257	209	190	167	-0,524161776	4,441313384	6,52E-06	0,000127728	down	LAMA3	ENSG00000053747
2614	2317	3889	3939	0,524206309	8,371153832	3,94E-12	2,72E-10	up	MAP4K4	ENSG00000071054
147	121	215	210	0,525074148	4,189321795	2,73E-05	0,000453182	up	IGF2BP3	ENSG00000136231
292	217	203	186	-0,525454124	4,565208617	6,04E-06	0,000119884	down	PARP10	ENSG00000178685
172	134	116	118	-0,525474418	3,839721295	0,000172531	0,002138254	down	ABCD1	ENSG00000101986
2130	1589	1497	1340	-0,525720705	7,425931772	2,05E-11	1,25E-09	down	SREBF1	ENSG00000072310
171	153	127	121	-0,526255551	3,922816705	9,52E-05	0,001320479	down	HS1BP3	ENSG00000118960
91	74	137	125	0,526448984	3,501643582	0,000635099	0,006433437	up	CTSV	ENSG00000136943
125	118	92	94	-0,5266218	3,514181121	0,000680637	0,00679262	down	ANKHD1	ENSG00000131503
1024	842	1496	1465	0,527009205	6,969948763	2,61E-12	1,88E-10	up	GALNT10	ENSG00000164574
291	215	194	192	-0,527352424	4,555827302	7,94E-06	0,000151226	down	FAM171B	ENSG00000144369
795	703	1228	1156	0,52765551	6,657664172	5,89E-12	3,91E-10	up	MAPK6	ENSG00000069956
96	87	80	60	-0,527668912	3,111895793	0,003268219	0,023958632	down	DTNA	ENSG00000134769
109	90	75	77	-0,527736375	3,229176664	0,001565344	0,013422545	down	BOC	ENSG00000144857
7095	5505	4894	4706	-0,527788396	9,187726773	2,59E-14	2,57E-12	down	CAPN2	ENSG00000162909
1488	1219	2115	2180	0,528190452	7,505511579	6,13E-12	4,05E-10	up	AFAP1	ENSG00000196526
702	720	561	533	-0,528772047	6,052908059	3,31E-07	9,04E-06	down	PSIP1	ENSG00000164985
500	424	339	366	-0,529609246	5,423128682	5,48E-08	1,75E-06	down	NUAK1	ENSG00000074590
130	94	174	182	0,530413765	3,935536853	0,000149866	0,001909158	up	AMDHD2	ENSG00000162066

641	529	943	919	0,530799037	6,301709965	8,89E-12	5,77E-10	up	AGPAT3	ENSG00000160216
287	181	375	366	0,531705611	4,980948613	1,23E-05	0,000221227	up	RMND5B	ENSG00000145916
4382	3871	3130	3171	-0,531937351	8,583542224	1,04E-12	8,05E-11	down	ELOVL5	ENSG00000012660
131	131	89	111	-0,532086126	3,620337638	0,002120859	0,017064673	down	ARHGEF4	ENSG00000136002
454	429	351	324	-0,532087259	5,360176026	8,36E-08	2,62E-06	down	FGD1	ENSG00000102302
337	290	254	224	-0,532565524	4,86450423	3,53E-07	9,50E-06	down	MAPK13	ENSG00000156711
362	268	258	221	-0,532836976	4,867383386	1,40E-06	3,23E-05	down	SLC16A2	ENSG00000147100
146	108	225	180	0,533255686	4,116111612	0,00011857	0,001575084	up	EPB41L5	ENSG00000115109
941	850	704	662	-0,534550019	6,377476223	2,75E-11	1,65E-09	down	BMP2K	ENSG00000138756
682	615	1098	979	0,535064741	6,455802555	4,49E-11	2,55E-09	up	SPRY4	ENSG00000187678
315	221	230	176	-0,536924612	4,632402602	5,08E-05	0,000771747	down	CARD19	ENSG00000165233
128	91	88	78	-0,537318523	3,358657583	0,001135124	0,010248456	down	FZD8	ENSG00000177283
2415	1990	1649	1691	-0,537486991	7,670843059	6,16E-13	4,90E-11	down	PXDN	ENSG00000130508
617	524	424	441	-0,539308377	5,722871756	1,93E-09	8,05E-08	down	IDH1	ENSG00000138413
356	335	262	263	-0,539530067	5,003980343	6,23E-07	1,57E-05	down	GLIPR1	ENSG00000139278
658	576	1038	942	0,539571516	6,385612911	4,57E-12	3,14E-10	up	ZFHX3	ENSG00000140836
213	152	145	131	-0,540153003	4,082992257	6,86E-05	0,001003927	down	SLCO1B3	ENSG00000111700
63	61	97	102	0,540698069	3,108934048	0,002809571	0,021293228	up	MOCS1	ENSG00000124615
128	98	90	81	-0,541013711	3,402623887	0,000733815	0,007221431	down	ATP6V0A4	ENSG00000105929
721	588	513	478	-0,541058631	5,918456482	3,88E-11	2,22E-09	down	YOD1	ENSG00000180667
315	224	476	387	0,541491436	5,192654101	1,25E-06	2,92E-05	up	TRIM16	ENSG00000221926
162	170	119	133	-0,54307519	3,954428408	0,000542588	0,005632249	down	FRY	ENSG00000073910
273	241	416	410	0,543282659	5,131587554	4,79E-08	1,55E-06	up	DPY19L4	ENSG00000156162
96	77	147	131	0,543684738	3,578793928	0,000301296	0,003447924	up	HDAC5	ENSG00000108840
408	342	305	262	-0,544381628	5,116191264	3,35E-08	1,11E-06	down	NEGR1	ENSG00000172260
292	185	187	171	-0,544705437	4,459356304	0,000120167	0,001592225	down	RHBDF1	ENSG00000007384
65	44	83	92	0,544728927	2,927031282	0,004675833	0,031973143	up	ZNF681	ENSG00000196172
141	107	223	176	0,545617532	4,090028986	0,00010577	0,001427194	up	C8orf58	ENSG00000241852
179	159	119	136	-0,546439315	3,974765833	0,000104085	0,001408065	down	STEAP2	ENSG00000157214
313	206	177	211	-0,547137071	4,579088559	0,000225759	0,002690571	down	SYNE1	ENSG00000131018
500	466	369	361	-0,547736272	5,482625605	1,42E-08	5,09E-07	down	EPB41L1	ENSG00000088367
680	602	492	475	-0,548907256	5,888233801	1,31E-10	6,95E-09	down	PRKAG2	ENSG00000106617

175	140	114	123	-0,548924286	3,871520993	9,73E-05	0,001337449	down	DBNDD1	ENSG00000003249
179	164	292	262	0,549352195	4,557505946	2,44E-06	5,30E-05	up	MARCKSL1	ENSG00000175130
1304	930	1843	1741	0,54945057	7,234880155	2,97E-11	1,74E-09	up	UCP2	ENSG00000175567
101	85	76	64	-0,549456053	3,12476848	0,001394774	0,012188767	down	NTHL1	ENSG00000065057
126	113	96	84	-0,549512961	3,48013275	0,000352646	0,003927229	down	SMIM29	ENSG00000186577
303	254	469	430	0,549942247	5,249314916	5,66E-09	2,21E-07	up	CDK2AP1	ENSG00000111328
903	794	1396	1346	0,54995742	6,850193109	1,24E-12	9,48E-11	up	FLG	ENSG00000143631
320	244	195	228	-0,550004314	4,7020337	1,14E-05	0,000207515	down	FRAS1	ENSG00000138759
336	191	211	181	-0,551048632	4,594947497	0,000688056	0,006853637	down	SLC45A3	ENSG00000158715
106	87	68	77	-0,551104445	3,176111113	0,001485543	0,012873321	down	CRYL1	ENSG00000165475
570	424	373	372	-0,551737895	5,51455074	6,26E-09	2,43E-07	down	ANK2	ENSG00000145362
343	247	506	446	0,552814336	5,329380144	2,62E-08	8,90E-07	up	SNRK	ENSG00000163788
125	121	220	179	0,553128061	4,087344821	9,67E-05	0,001332282	up	MT1X	ENSG00000187193
255	186	345	366	0,553246805	4,913706797	9,82E-07	2,37E-05	up	UBTD2	ENSG00000168246
67	70	105	117	0,553454658	3,258216218	0,002417405	0,018911779	up	NATD1	ENSG00000274180
77	59	54	48	-0,553526657	2,682792551	0,004933872	0,033254381	down	TXNRD3	ENSG00000197763
452	302	307	256	-0,554156538	5,112510316	5,19E-06	0,00010513	down	ITGB5	ENSG00000082781
126	83	180	158	0,555807758	3,851067944	0,000135626	0,001755109	up	RDH10	ENSG00000121039
417	377	300	296	-0,555838465	5,195767753	2,24E-08	7,72E-07	down	RAPGEF2	ENSG00000109756
59	52	84	96	0,55677378	2,962124479	0,003651152	0,026176707	up	RMI2	ENSG00000175643
1299	904	1868	1684	0,557214808	7,218098423	1,53E-10	8,07E-09	up	OSBPL8	ENSG00000091039
195	156	156	107	-0,55767115	4,022086789	0,000217051	0,002599113	down	AGFG2	ENSG00000106351
202	219	319	368	0,55903215	4,862340552	0,000110218	0,001475998	up	HIRIP3	ENSG00000149929
830	649	560	544	-0,559331238	6,085218829	9,71E-12	6,26E-10	down	ADCY7	ENSG00000121281
124	129	203	209	0,559342721	4,132242955	8,96E-05	0,001261662	up	ZNF92	ENSG00000146757
339	274	237	221	-0,559896668	4,819200062	7,71E-08	2,44E-06	down	DNAJC16	ENSG00000116138
110	82	64	79	-0,561268359	3,163138335	0,002352299	0,018514368	down	TRIM66	ENSG00000166436
161	143	222	272	0,561415596	4,391780123	0,00013471	0,001746175	up	GFPT2	ENSG00000131459
50	33	68	67	0,561664734	2,557498691	0,007724813	0,047773247	up	FBXO25	ENSG00000147364
92	65	64	53	-0,561973314	2,879493491	0,00333123	0,024343847	down	MRAS	ENSG00000158186
1517	1355	2409	2276	0,562040238	7,615453944	2,84E-14	2,75E-12	up	U2SURP	ENSG00000163714
292	271	476	442	0,562412867	5,274836822	1,03E-08	3,79E-07	up	B4GALT6	ENSG00000118276

982	686	622	615	-0,562414995	6,252029058	2,02E-09	8,38E-08	down	SNX17	ENSG00000115234
583	458	422	354	-0,56314303	5,577945567	9,85E-10	4,41E-08	down	MGAT5B	ENSG00000167889
87	72	134	125	0,563171833	3,471522004	0,000261751	0,003058938	up	IFT22	ENSG00000128581
1392	1149	940	953	-0,563444691	6,866262765	3,98E-13	3,22E-11	down	ZBTB38	ENSG00000177311
210	195	351	310	0,563654074	4,804004643	3,85E-07	1,03E-05	up	EYA3	ENSG00000158161
185	200	339	293	0,565648133	4,737997729	1,69E-05	0,000296668	up	UBN2	ENSG00000157741
109	86	72	73	-0,566008425	3,184060275	0,000882036	0,008408133	down	ST3GAL3	ENSG00000126091
301	283	497	458	0,56602486	5,329929163	7,81E-09	2,96E-07	up	GDF11	ENSG00000135414
2482	1813	1630	1550	-0,566260133	7,615730184	1,26E-12	9,53E-11	down	SREBF2	ENSG00000198911
60	77	111	114	0,566373814	3,270547532	0,004437444	0,030667142	up	RNF207	ENSG00000158286
1355	1675	1107	1184	-0,566374622	7,141662028	9,88E-05	0,001352517	down	MTND2P28	ENSG00000225630
535	407	796	739	0,566648312	6,009375996	1,14E-11	7,22E-10	up	STK35	ENSG00000125834
78	62	56	48	-0,567602596	2,7177392	0,003729408	0,026651626	down	LMCD1	ENSG00000071282
1334	1078	932	860	-0,567834151	6,788199718	6,19E-14	5,72E-12	down	PALLD	ENSG00000129116
317	254	216	208	-0,568412253	4,713685142	1,38E-07	4,18E-06	down	CCSAP	ENSG00000154429
150	126	102	103	-0,568435774	3,675952445	9,08E-05	0,00127315	down	HNMT	ENSG00000150540
90	81	62	65	-0,568715633	2,999077673	0,001524293	0,01313432	down	HECW2	ENSG00000138411
866	719	589	588	-0,568768218	6,183506621	1,26E-12	9,53E-11	down	KCTD12	ENSG00000178695
106	76	72	63	-0,568790129	3,08453087	0,001709234	0,014417539	down	TK2	ENSG00000166548
1112	921	1718	1607	0,569497504	7,118941283	6,19E-15	6,49E-13	up	RBBP4	ENSG00000162521
246	164	156	147	-0,569961658	4,234501445	4,57E-05	0,0007041	down	TPST2	ENSG00000128294
133	98	201	177	0,57090405	4,004104671	1,99E-05	0,00034239	up	CCNE1	ENSG00000105173
183	203	357	281	0,571279001	4,747444936	8,34E-05	0,001189116	up	ZCRB1	ENSG00000139168
178	134	115	116	-0,571498514	3,847515047	4,89E-05	0,00074441	down	CAMKK1	ENSG00000004660
2205	1871	1475	1546	-0,571584151	7,546454694	2,42E-13	2,05E-11	down	SERINC3	ENSG00000132824
1627	1417	2583	2413	0,571940475	7,703767934	1,27E-15	1,45E-13	up	SMARCC1	ENSG00000173473
3952	3102	2714	2504	-0,572209303	8,333275143	2,07E-16	2,59E-14	down	PLS3	ENSG00000102024
190	124	276	237	0,572209721	4,438488698	1,22E-05	0,000220097	up	TSPAN6	ENSG00000000003
271	208	397	387	0,572282299	5,045061339	1,28E-08	4,62E-07	up	TBC1D12	ENSG00000108239
244	174	161	148	-0,572437673	4,262928553	9,74E-06	0,000181894	down	APBB1	ENSG00000166313
60	36	75	82	0,572549351	2,764855237	0,005109751	0,034143425	up	PCMTD2	ENSG00000203880
457	369	278	332	-0,573313767	5,241738183	6,15E-07	1,55E-05	down	ADGRL2	ENSG00000117114

303	318	497	527	0,574086686	5,427631087	1,30E-06	3,02E-05	up	EVI2B	ENSG00000185862
132	93	201	168	0,574644836	3,968182497	4,23E-05	0,000656688	up	PBXIP1	ENSG00000163346
131	119	96	89	-0,574891307	3,533626508	0,000164436	0,002057635	down	SP6	ENSG00000189120
88	77	66	56	-0,575231681	2,945740607	0,001628942	0,013860737	down	ITGBL1	ENSG00000198542
613	439	399	376	-0,575397131	5,584797309	3,23E-09	1,31E-07	down	MPP4	ENSG00000082126
126	100	87	80	-0,575436121	3,388713053	0,000262449	0,003064343	down	ASIC1	ENSG00000110881
98	90	69	70	-0,575639091	3,130004338	0,000962676	0,009027409	down	OSBP2	ENSG00000184792
760	702	1257	1154	0,576638562	6,654059044	7,67E-13	6,04E-11	up	RBM22	ENSG00000086589
486	402	373	284	-0,576895223	5,345285903	4,87E-08	1,57E-06	down	FIGN	ENSG00000182263
539	494	405	360	-0,577305496	5,566295449	6,11E-10	2,88E-08	down	STK39	ENSG00000198648
157	124	239	223	0,57736616	4,287712326	1,94E-06	4,30E-05	up	GABBR2	ENSG00000136928
183	124	278	226	0,577798266	4,410652433	1,39E-05	0,000247405	up	NR2C2AP	ENSG00000184162
339	259	238	203	-0,577861134	4,774688226	1,65E-07	4,89E-06	down	CLN5	ENSG00000102805
216	185	339	321	0,578082121	4,796791348	5,05E-08	1,62E-06	up	INTS14	ENSG00000138614
218	176	325	323	0,578319671	4,770688366	8,30E-08	2,61E-06	up	HSDL1	ENSG00000103160
61	40	79	87	0,578577055	2,840403684	0,003506112	0,025323915	up	MYO1D	ENSG00000176658
76	65	61	43	-0,579116185	2,723577151	0,004277482	0,029800364	down	DMAC1	ENSG00000137038
80	52	106	111	0,579516795	3,216190043	0,001021973	0,009407363	up	RAB36	ENSG00000100228
4291	3187	2764	2721	-0,579560144	8,41114617	5,37E-14	4,99E-12	down	LDLR	ENSG00000130164
330	244	209	213	-0,580059554	4,714329554	4,67E-07	1,23E-05	down	GCNT1	ENSG00000187210
239	202	179	146	-0,581209853	4,33922956	1,92E-06	4,27E-05	down	PANX2	ENSG00000073150
159	133	111	104	-0,581222011	3,750673603	3,20E-05	0,000517757	down	KANK1	ENSG00000107104
289	249	429	458	0,581546134	5,219535359	2,50E-08	8,56E-07	up	ASS1	ENSG00000130707
2116	1816	3521	2984	0,581911048	8,077645469	2,79E-14	2,73E-12	up	SERTAD2	ENSG00000179833
313	266	203	223	-0,582008859	4,729454897	3,63E-07	9,69E-06	down	EML2	ENSG00000125746
889	790	620	617	-0,582993425	6,263593132	1,07E-12	8,25E-11	down	NPAS2	ENSG00000170485
91	71	66	53	-0,58386844	2,915411767	0,001556259	0,013359399	down	ECHDC3	ENSG00000134463
516	376	303	349	-0,584132529	5,34417406	3,47E-07	9,41E-06	down	GALC	ENSG00000054983
420	357	279	292	-0,584160088	5,151146224	4,80E-09	1,90E-07	down	REEP6	ENSG00000115255
193	178	143	130	-0,584217687	4,092523815	8,23E-06	0,000156447	down	LETM2	ENSG00000165046
597	417	393	349	-0,584488457	5,527107148	1,43E-08	5,10E-07	down	GAMT	ENSG00000130005
1025	767	720	594	-0,584780259	6,349208545	3,71E-11	2,14E-09	down	FHL2	ENSG00000115641

555	482	375	387	-0,58514952	5,566913785	1,85E-10	9,59E-09	down	SGMS1	ENSG00000198964
130	95	85	80	-0,585276315	3,377387227	0,000316403	0,003586438	down	RASSF4	ENSG00000107551
159	130	252	226	0,585368809	4,3330277	1,28E-06	2,96E-05	up	DCLRE1C	ENSG00000152457
192	182	146	129	-0,586330832	4,103786661	1,05E-05	0,000194253	down	SLC9A7	ENSG00000065923
264	224	173	185	-0,586393467	4,482411333	6,91E-07	1,71E-05	down	SMAGP	ENSG00000170545
142	109	205	210	0,586461175	4,132113834	6,03E-06	0,000119884	up	TENM2	ENSG00000145934
427	376	313	277	-0,586702909	5,198241864	1,73E-09	7,27E-08	down	DNASE2	ENSG00000105612
64	64	53	41	-0,586742181	2,58674682	0,006140527	0,039682162	down	TMEM108	ENSG00000144868
277	272	212	192	-0,587701702	4,654516023	1,31E-06	3,03E-05	down	SLITRK5	ENSG00000165300
110	104	84	73	-0,588011438	3,308315305	0,000351009	0,003913011	down	ERO1B	ENSG00000086619
17517	24905	15539	16306	-0,588049963	10,95549123	0,001046526	0,009587906	down	MT-ATP6	ENSG00000198899
59	65	108	98	0,58810854	3,13908356	0,001725588	0,014506637	up	NFKBIZ	ENSG00000144802
245	201	182	145	-0,588215203	4,351871574	2,09E-06	4,60E-05	down	MAFK	ENSG00000198517
346	347	570	583	0,588810976	5,591993601	2,11E-08	7,32E-07	up	COL27A1	ENSG00000196739
85	57	114	121	0,589003427	3,325074519	0,000614203	0,006250302	up	STAG3L4	ENSG00000106610
1396	1310	1135	863	-0,5890518	6,9535014	6,98E-09	2,68E-07	down	NFIX	ENSG00000008441
307	249	482	440	0,58954737	5,269978171	3,26E-10	1,63E-08	up	PRIM1	ENSG00000198056
635	590	969	1067	0,590383063	6,408576024	1,20E-09	5,28E-08	up	IFIT1	ENSG00000185745
570	467	405	354	-0,59047063	5,562563927	2,43E-11	1,47E-09	down	ENDOD1	ENSG00000149218
1138	942	783	738	-0,591540135	6,566001284	3,39E-15	3,65E-13	down	NEK6	ENSG00000119408
234	181	152	151	-0,592027643	4,246372267	1,96E-06	4,35E-05	down	KSR1	ENSG00000141068
138	98	82	90	-0,592396265	3,441392932	0,000382787	0,004212829	down	SLC35E4	ENSG00000100036
252	215	162	179	-0,593006208	4,416802793	1,30E-06	3,01E-05	down	THRB	ENSG00000151090
2188	1663	1301	1494	-0,593281516	7,449372598	6,50E-10	3,05E-08	down	TANC2	ENSG00000170921
184	152	287	272	0,594088472	4,553385135	1,60E-07	4,76E-06	up	ZNF286A	ENSG00000187607
83	89	138	149	0,594664358	3,605550932	0,000357821	0,003969006	up	EIF4E3	ENSG00000163412
297	291	193	237	-0,5949886	4,750434288	2,15E-05	0,000367168	down	PDZD2	ENSG00000133401
324	308	231	231	-0,595447879	4,852673915	1,48E-07	4,45E-06	down	SCARA3	ENSG00000168077
199	162	129	134	-0,595839488	4,046649544	5,83E-06	0,000116284	down	P2RX5	ENSG00000083454
808	619	530	508	-0,595980822	6,017766315	1,45E-12	1,08E-10	down	CYBA	ENSG00000051523
132	111	90	87	-0,596607934	3,483450558	8,22E-05	0,001176507	down	KCTD18	ENSG00000155729
220	180	347	320	0,597387319	4,804106889	1,51E-08	5,37E-07	up	ZEB2	ENSG00000169554

80	71	64	46	-0,597533952	2,812388691	0,002418624	0,018911788	down	FHOD3	ENSG00000134775
167	144	269	250	0,597841877	4,446052881	2,96E-07	8,23E-06	up	DOLK	ENSG00000175283
1511	1396	1067	1056	-0,598501935	7,051980346	2,91E-13	2,43E-11	down	LMO7	ENSG00000136153
1282	956	887	737	-0,599381146	6,66340188	3,56E-12	2,48E-10	down	IL11	ENSG00000095752
572	418	841	807	0,59954044	6,098982191	2,72E-12	1,95E-10	up	PFKFB3	ENSG00000170525
376	234	545	467	0,599549532	5,399425035	6,04E-07	1,53E-05	up	DCTPP1	ENSG00000179958
22763	29328	19100	19524	-0,599922972	11,24063814	7,95E-05	0,001143877	down	MT-ND1	ENSG00000198888
1190	931	1895	1645	0,600052779	7,19556219	9,70E-15	1,00E-12	up	RNF40	ENSG00000103549
237	208	379	365	0,600403606	4,959773333	5,29E-09	2,08E-07	up	B4GALNT1	ENSG00000135454
98	88	153	158	0,600625905	3,717207277	4,62E-05	0,00070862	up	PAIP2B	ENSG00000124374
62	51	48	34	-0,601635611	2,405573231	0,006488319	0,041669606	down	NFATC1	ENSG00000131196
348	266	492	532	0,601935338	5,417536994	5,04E-09	1,98E-07	up	GINS4	ENSG00000147536
91	96	164	150	0,60348438	3,729026209	9,76E-05	0,001339607	up	ARRDC3	ENSG00000113369
486	377	326	299	-0,60380217	5,291250051	1,64E-10	8,58E-09	down	UCKL1	ENSG00000198276
491	369	311	311	-0,603805163	5,285289029	9,57E-10	4,31E-08	down	RAB30	ENSG00000137502
176	163	127	119	-0,603975057	3,955596441	8,29E-06	0,000157462	down	FAM86C1	ENSG00000158483
241	208	185	141	-0,604065025	4,35613331	2,63E-06	5,66E-05	down	FAM102A	ENSG00000167106
640	554	433	433	-0,604222511	5,762125263	5,65E-12	3,77E-10	down	GLUL	ENSG00000135821
327	312	228	236	-0,605134604	4,864727645	1,65E-07	4,89E-06	down	TBC1D9	ENSG00000109436
179	151	124	115	-0,605235607	3,915144457	6,27E-06	0,000123519	down	ARID3A	ENSG00000116017
81	71	65	45	-0,607183923	2,817666674	0,002245374	0,017844637	down	ZBED6	ENSG00000257315
278	259	192	197	-0,607375195	4,613183998	3,11E-07	8,60E-06	down	DYRK3	ENSG00000143479
663	542	965	1054	0,607537439	6,389761687	1,16E-11	7,31E-10	up	MYO18A	ENSG00000196535
370	336	285	227	-0,607951505	5,005465765	4,12E-08	1,35E-06	down	RPIA	ENSG00000153574
5491	4972	3980	3612	-0,609339608	8,896271945	1,13E-16	1,46E-14	down	BTF3	ENSG00000145741
190	153	309	268	0,609775742	4,591904133	1,05E-07	3,22E-06	up	PDLIM2	ENSG00000120913
106	88	74	66	-0,609877581	3,159186443	0,000338706	0,003792183	down	ICA1	ENSG00000003147
117	91	76	74	-0,61013514	3,256897811	0,000233049	0,002766832	down	ANOS1	ENSG00000011201
350	256	552	466	0,610178114	5,402833457	1,50E-09	6,44E-08	up	CKB	ENSG00000166165
329	242	204	207	-0,610461337	4,694177173	1,28E-07	3,89E-06	down	PIK3CG	ENSG00000105851
161	108	87	106	-0,610575666	3,617523469	0,000423895	0,004580837	down	IL15RA	ENSG00000134470
214	177	139	143	-0,610668275	4,154822806	1,42E-06	3,25E-05	down	MBLAC2	ENSG00000176055

783	656	532	506	-0,611526528	6,026965779	3,37E-14	3,23E-12	down	SDC3	ENSG00000162512
100	94	76	64	-0,611601442	3,15985595	0,00040866	0,004442526	down	STK33	ENSG00000130413
2228	1598	3437	2977	0,61183539	8,046016568	3,35E-13	2,75E-11	up	MTHFD2	ENSG00000065911
248	196	368	379	0,61204359	4,961437062	6,56E-09	2,53E-07	up	RNF19A	ENSG00000034677
69	71	54	47	-0,612586659	2,701770968	0,002392898	0,018757855	down	MMP19	ENSG00000123342
73	57	101	118	0,613619555	3,217092295	0,000765406	0,007480172	up	MGAT4A	ENSG00000071073
133	114	220	197	0,614375381	4,127854468	1,78E-06	3,99E-05	up	DOK3	ENSG00000146094
278	210	161	189	-0,614510254	4,46787332	3,01E-06	6,45E-05	down	IGFBP6	ENSG00000167779
276	215	197	156	-0,615429149	4,477163209	4,23E-07	1,12E-05	down	APCDD1L	ENSG00000198768
193	140	121	118	-0,615608189	3,92141082	1,04E-05	0,000193187	down	SCN5A	ENSG00000183873
478	374	316	296	-0,615724146	5,268237674	6,94E-11	3,86E-09	down	TIAM1	ENSG00000156299
41	30	62	58	0,615837057	2,374216666	0,007300523	0,045713638	up	TBC1D32	ENSG00000146350
290	235	446	441	0,617246082	5,204867512	1,85E-10	9,59E-09	up	APOBEC3B	ENSG00000179750
371	326	639	542	0,617562896	5,613133393	5,91E-11	3,31E-09	up	RBM38	ENSG00000132819
74	52	109	104	0,617612819	3,175219333	0,000336799	0,003778566	up	ZNF354C	ENSG00000177932
1268	1079	2051	1923	0,618295391	7,356360652	9,95E-18	1,44E-15	up	MED13L	ENSG00000123066
42	40	77	62	0,618625841	2,577017315	0,004802773	0,032583445	up	STON2	ENSG00000140022
1030	867	1561	1646	0,618734523	7,04994404	4,79E-14	4,48E-12	up	PHLDB2	ENSG00000144824
64	56	44	42	-0,619393425	2,482367723	0,003648886	0,026176707	down	HCN4	ENSG00000138622
28657	36717	23003	24767	-0,619413608	11,56033521	5,85E-05	0,000870507	down	MT-ND2	ENSG00000198763
3503	2823	2279	2250	-0,619635458	8,158628109	1,27E-18	1,97E-16	down	PYGL	ENSG00000100504
145	119	91	98	-0,620948834	3,590930053	4,08E-05	0,000638069	down	RTL8B	ENSG00000212747
815	642	520	522	-0,620967895	6,038739493	1,17E-13	1,05E-11	down	NACC2	ENSG00000148411
719	641	1169	1140	0,621255418	6,575605043	3,93E-15	4,18E-13	up	SRSF4	ENSG00000116350
465	336	302	270	-0,621728637	5,174483867	2,83E-09	1,16E-07	down	PPP2R2C	ENSG00000074211
244	188	134	174	-0,621904673	4,290483744	3,19E-05	0,000516616	down	CA13	ENSG00000185015
108	76	163	149	0,622593869	3,712531787	2,40E-05	0,000405498	up	RHPN1	ENSG00000158106
573	423	358	352	-0,62388928	5,487528466	9,24E-11	5,00E-09	down	SLC8B1	ENSG00000089060
142	107	229	194	0,624529373	4,143731102	1,86E-06	4,15E-05	up	RANGRF	ENSG00000108961
84	51	46	50	-0,62483065	2,640538571	0,004972526	0,033370078	down	SLC1A1	ENSG00000106688
86	72	152	117	0,625377145	3,500858989	0,000147078	0,001881356	up	CPT1C	ENSG00000169169
758	586	464	493	-0,625633907	5,91973675	5,17E-12	3,46E-10	down	WNT7B	ENSG00000188064

122	117	96	75	-0,625849709	3,449950711	0,000135432	0,001754063	down	TNFAIP3	ENSG00000118503
536	434	349	343	-0,62612919	5,451703076	3,52E-12	2,46E-10	down	PPTC7	ENSG00000196850
80	75	132	132	0,626706765	3,475432856	8,25E-05	0,001179953	up	NDUFAF5	ENSG00000101247
155	95	224	200	0,627466863	4,146817905	1,14E-05	0,000207851	up	RPP25	ENSG00000178718
289	265	452	492	0,627543661	5,291474802	7,13E-09	2,73E-07	up	KDM3A	ENSG00000115548
296	228	188	185	-0,628345041	4,565062949	3,99E-08	1,32E-06	down	SRC	ENSG00000197122
144	124	100	91	-0,628526553	3,609678658	1,69E-05	0,000296668	down	CD274	ENSG00000120217
407	321	256	262	-0,628940229	5,03712484	7,26E-10	3,36E-08	down	P4HA2	ENSG00000072682
83	47	45	47	-0,629442327	2,584831628	0,007525601	0,046839703	down	TEC	ENSG00000135605
845	689	1303	1309	0,629472069	6,749857759	1,44E-15	1,62E-13	up	PURB	ENSG00000146676
211	157	327	300	0,629895098	4,703206631	1,23E-08	4,47E-07	up	GINS3	ENSG00000181938
1549	1323	2607	2305	0,631306395	7,654995242	4,89E-18	7,31E-16	up	FMNL1	ENSG00000184922
56	44	86	85	0,633118371	2,861422554	0,000842217	0,00811861	up	RTEL1	ENSG00000258366
388	327	260	248	-0,633232209	5,011162757	1,79E-10	9,33E-09	down	MCTP1	ENSG00000175471
276	221	193	160	-0,63327684	4,487929989	6,46E-08	2,06E-06	down	NIPAL1	ENSG00000163293
540	459	913	797	0,6333532	6,138050048	2,25E-14	2,24E-12	up	DCAF16	ENSG00000163257
45	31	69	61	0,633426663	2,478668131	0,0037722	0,026895542	up	DENND2C	ENSG00000175984
83	72	58	52	-0,633975202	2,8340012	0,000789234	0,007698498	down	TMEM44	ENSG00000145014
341	290	566	514	0,634155642	5,479642375	1,88E-12	1,39E-10	up	SEPHS2	ENSG00000179918
216	162	142	126	-0,634265121	4,095056963	1,33E-06	3,06E-05	down	MCC	ENSG00000171444
64	70	124	106	0,634424379	3,276893332	0,000406118	0,004419488	up	NSMCE2	ENSG00000156831
488	398	343	286	-0,634670289	5,317883718	3,42E-11	1,99E-09	down	RMDN3	ENSG00000137824
143	111	92	88	-0,63540396	3,52950118	3,05E-05	0,000495473	down	ARID3B	ENSG00000179361
282	274	217	179	-0,635685715	4,652938346	2,88E-07	8,03E-06	down	SMPD1	ENSG00000166311
65	69	51	44	-0,638312891	2,630597642	0,002508746	0,019518197	down	PDE11A	ENSG00000128655
1969	1528	1219	1248	-0,638377681	7,293599454	3,87E-16	4,69E-14	down	TSPAN14	ENSG00000108219
73	78	57	50	-0,639771395	2,797490715	0,00139434	0,012188767	down	FRMPD3	ENSG00000147234
1744	1432	2730	2718	0,639970251	7,802965197	8,85E-19	1,42E-16	up	ICK	ENSG00000112144
1369	1233	935	909	-0,640564908	6,873610367	8,55E-16	9,83E-14	down	TFPI	ENSG00000003436
35	30	60	52	0,642798645	2,269267842	0,006730697	0,042924434	up	CORO1A	ENSG00000102879
64	51	50	31	-0,644837942	2,412513612	0,004620599	0,031651202	down	SP5	ENSG00000204335
163	160	112	116	-0,645143511	3,871218846	1,10E-05	0,000200841	down	OSBPL1A	ENSG00000141447

131	98	82	79	-0,646296306	3,377820522	6,19E-05	0,000916361	down	RPL13AP20	ENSG00000234498
1353	1008	2121	1939	0,646608186	7,375534084	5,05E-17	6,95E-15	up	ATP13A3	ENSG00000133657
363	282	224	229	-0,647421767	4,855438978	1,67E-09	7,09E-08	down	CREB5	ENSG00000146592
402	333	671	599	0,64817125	5,706187926	1,19E-13	1,06E-11	up	ZIC2	ENSG00000043355
374	324	259	232	-0,648675061	4,970968457	1,63E-10	8,58E-09	down	BAZ2B	ENSG00000123636
53	66	113	94	0,649557355	3,121917981	0,001362578	0,011954508	up	CENPH	ENSG00000153044
510	458	351	330	-0,649742299	5,442418978	3,17E-12	2,23E-10	down	AP1G2	ENSG00000213983
627	550	373	453	-0,649844299	5,723559486	8,11E-09	3,05E-07	down	ITGA2	ENSG00000164171
79	65	56	45	-0,650619531	2,723761767	0,000922888	0,008738741	down	CCDC169	ENSG00000242715
162	121	100	98	-0,653094353	3,675313826	1,05E-05	0,000194675	down	PROS1	ENSG00000184500
154	97	92	83	-0,653341075	3,501543968	0,00015401	0,00195234	down	ADAMTS15	ENSG00000166106
143	131	218	257	0,65338661	4,300851512	6,29E-06	0,000123682	up	PER3	ENSG00000049246
92	71	54	60	-0,653661592	2,895888534	0,000544796	0,005643854	down	CPED1	ENSG00000106034
1103	736	687	592	-0,654010437	6,353173833	1,91E-10	9,83E-09	down	DUSP1	ENSG00000120129
152	134	262	235	0,655676464	4,362547183	5,95E-08	1,90E-06	up	GNAO1	ENSG00000087258
145	107	94	82	-0,655878775	3,509279443	2,52E-05	0,000421333	down	RGS20	ENSG00000147509
38	27	53	60	0,657245222	2,276988674	0,005202076	0,034685718	up	CEP19	ENSG00000174007
99	54	54	52	-0,657677126	2,799847942	0,00365056	0,026176707	down	PLEKHN1	ENSG00000187583
284	247	202	169	-0,658799591	4,574303343	9,71E-09	3,59E-07	down	FAM131A	ENSG00000175182
50	39	35	27	-0,659283347	2,052913667	0,007239149	0,045476027	down	PSPN	ENSG00000125650
494	398	332	290	-0,65979323	5,317189903	1,87E-12	1,39E-10	down	KIAA1522	ENSG00000162522
297	212	486	399	0,660136316	5,183497759	2,18E-09	8,99E-08	up	CARHSP1	ENSG00000153048
62	49	119	75	0,661131036	3,026021445	0,00271384	0,020739777	up	EPCAM	ENSG00000119888
69	56	45	42	-0,661190281	2,522068816	0,001697757	0,014351916	down	GPR155	ENSG00000163328
67	44	33	44	-0,661273342	2,354841747	0,006699732	0,042779665	down	OPLAH	ENSG00000178814
351	292	576	545	0,661620648	5,523082823	8,70E-14	7,99E-12	up	DPY19L3	ENSG00000178904
844	939	1622	1524	0,662272902	7,002795662	3,54E-09	1,42E-07	up	SRP14	ENSG00000140319
207	202	163	123	-0,66268741	4,202047124	3,92E-06	8,22E-05	down	ADAM12	ENSG00000148848
229	163	134	138	-0,662796054	4,134314877	1,13E-06	2,66E-05	down	IQSEC2	ENSG00000124313
58	32	68	88	0,66322123	2,725602996	0,004419898	0,030614007	up	CCDC28A	ENSG00000024862
82	55	135	104	0,663331158	3,32020922	0,000188241	0,002307181	up	TAF4B	ENSG00000141384
467	399	281	321	-0,663563002	5,275124065	5,05E-10	2,42E-08	down	PITPNM3	ENSG00000091622

92	59	40	64	-0,663960236	2,779476772	0,007467431	0,046537084	down	GLI2	ENSG00000074047
893	816	1504	1488	0,664263923	6,931729561	2,50E-16	3,08E-14	up	B4GALT5	ENSG00000158470
328	267	532	507	0,664448834	5,413217178	3,14E-13	2,61E-11	up	PLEKHA8	ENSG00000106086
123	103	78	79	-0,664643884	3,353127638	2,88E-05	0,000474734	down	KALRN	ENSG00000160145
1267	1174	877	824	-0,666874707	6,772329449	7,72E-16	9,08E-14	down	ECM1	ENSG00000143369
588	438	346	363	-0,668067994	5,51274037	1,02E-11	6,54E-10	down	PEX5	ENSG00000139197
45	43	36	25	-0,668198441	2,035185888	0,00799075	0,049066172	down	IL1R1	ENSG00000115594
121	91	184	187	0,668201775	3,942316834	1,17E-06	2,75E-05	up	ARRDC2	ENSG00000105643
237	198	177	125	-0,668735474	4,284064845	1,43E-06	3,27E-05	down	LIMS2	ENSG00000072163
335	283	542	541	0,669306403	5,47150316	3,32E-13	2,74E-11	up	OPHN1	ENSG00000079482
89	65	147	123	0,669740831	3,490330391	2,91E-05	0,000478845	up	RAB24	ENSG00000169228
415	370	277	267	-0,670724636	5,132025744	1,54E-11	9,58E-10	down	MPV17	ENSG00000115204
660	492	1064	953	0,670861621	6,360775784	1,91E-16	2,41E-14	up	RICTOR	ENSG00000164327
32	31	65	46	0,672668175	2,245618825	0,007343555	0,045872108	up	LBHD1	ENSG00000162194
36	41	80	56	0,672690561	2,525841885	0,00572256	0,037432873	up	MNS1	ENSG00000138587
91	59	129	134	0,672841246	3,453309235	4,86E-05	0,000741292	up	LRATD2	ENSG00000168672
97	77	53	67	-0,67327909	2,979974353	0,000468664	0,004991694	down	FUCA1	ENSG00000179163
261	232	187	154	-0,67390597	4,462311071	2,18E-08	7,53E-07	down	FAM86DP	ENSG00000244026
814	697	531	513	-0,674308716	6,073127518	9,43E-17	1,23E-14	down	SUCLG2	ENSG00000172340
935	690	1484	1367	0,675514315	6,856490062	4,22E-17	5,91E-15	up	PLXNA1	ENSG00000114554
112	73	66	61	-0,677906947	3,062032469	0,000319069	0,003614019	down	SMARCD3	ENSG00000082014
125	91	62	86	-0,678367939	3,280813152	0,000428353	0,004622582	down	FBXL2	ENSG00000153558
624	431	361	361	-0,678796951	5,54556628	1,35E-10	7,15E-09	down	GSN	ENSG00000148180
433	357	297	247	-0,678881189	5,135536222	1,29E-11	8,09E-10	down	SLC25A43	ENSG00000077713
87	63	49	54	-0,679357636	2,768582468	0,000583011	0,005972015	down	PCBD2	ENSG00000132570
693	590	1167	1099	0,679507514	6,525834071	6,40E-19	1,05E-16	up	CHRAC1	ENSG00000104472
548	453	375	314	-0,679713875	5,47595959	2,33E-13	1,99E-11	down	TFPI2	ENSG00000105825
1286	1055	2289	1853	0,680500832	7,389180397	7,62E-16	9,03E-14	up	EPN1	ENSG00000063245
629	537	1052	1009	0,680899498	6,389480178	8,54E-19	1,38E-16	up	CDCA7	ENSG00000144354
1207	1119	805	799	-0,681035835	6,697070462	2,22E-16	2,76E-14	down	AKAP12	ENSG00000131016
56	63	113	98	0,681193124	3,138692047	0,000261789	0,003058938	up	PEX11B	ENSG00000131779
46	36	71	74	0,681250663	2,614067333	0,000854783	0,008203914	up	HSD3B7	ENSG00000099377

112	99	174	199	0,681901428	3,946101725	5,81E-06	0,000116163	up	FOXO4	ENSG00000184481
435	329	287	237	-0,682140535	5,083653666	1,19E-10	6,37E-09	down	NOG	ENSG00000183691
78	62	139	109	0,683316921	3,365189361	5,61E-05	0,00083991	up	PID1	ENSG00000153823
361	315	230	234	-0,683447621	4,911423002	7,18E-11	3,96E-09	down	COBLL1	ENSG00000082438
42	32	60	71	0,683732085	2,472543547	0,002615198	0,020154544	up	ADORA1	ENSG00000163485
40	35	72	61	0,684254504	2,492487468	0,001571845	0,013463418	up	TMC7	ENSG00000170537
58	33	87	74	0,684653298	2,75844129	0,00100985	0,009323398	up	GSPT2	ENSG00000189369
114	93	201	166	0,684836879	3,919817442	1,06E-06	2,52E-05	up	CLDN11	ENSG00000013297
133	84	76	72	-0,685465688	3,283134312	0,000168852	0,002102729	down	C11orf45	ENSG00000174370
97	84	164	157	0,685564183	3,730541466	2,16E-06	4,74E-05	up	EME2	ENSG00000197774
1104	959	752	664	-0,685918512	6,518571882	2,43E-18	3,66E-16	down	NAV3	ENSG00000067798
710	629	1232	1147	0,68635774	6,593078523	9,68E-19	1,53E-16	up	AQR	ENSG00000021776
81	66	142	119	0,686924019	3,436421342	1,90E-05	0,00032983	up	ZNF844	ENSG00000223547
265	250	177	176	-0,687555699	4,52107931	1,31E-08	4,72E-07	down	HPSE	ENSG00000173083
840	746	603	486	-0,687656347	6,139328431	3,63E-14	3,44E-12	down	CD55	ENSG00000196352
101	72	159	148	0,687961059	3,665943672	3,05E-06	6,52E-05	up	HR	ENSG00000168453
293	212	170	174	-0,689498277	4,486048098	2,61E-08	8,89E-07	down	PDK1	ENSG00000152256
57	50	38	35	-0,69001734	2,295415555	0,002375611	0,018641165	down	CEP170	ENSG00000143702
137	95	210	202	0,690334827	4,082870734	2,23E-07	6,38E-06	up	CABLES1	ENSG00000134508
59	57	41	38	-0,693515415	2,406676429	0,002034308	0,016536254	down	ATL1	ENSG00000198513
97	91	61	67	-0,694595528	3,082417442	0,000105783	0,001427194	down	COLGALT2	ENSG00000198756
84	79	62	49	-0,694923904	2,881297698	0,000315602	0,003579969	down	PCDHB16	ENSG00000272674
121	101	79	72	-0,695237944	3,315596277	2,02E-05	0,000346778	down	ZNF699	ENSG00000196110
541	384	927	721	0,696261247	6,059715762	7,03E-11	3,90E-09	up	TFAP2C	ENSG00000087510
71	45	109	98	0,696365605	3,106484276	0,00010997	0,001474715	up	TSPAN33	ENSG00000158457
462	370	298	267	-0,697629026	5,201891063	3,49E-13	2,85E-11	down	SEMA3E	ENSG00000170381
117	90	57	83	-0,698052272	3,213721493	0,0003978	0,004353378	down	PPIC	ENSG00000168938
91	97	66	62	-0,698186994	3,082950076	0,000217136	0,002599113	down	ANK3	ENSG00000151150
113	92	67	72	-0,69904168	3,20132253	3,34E-05	0,000533728	down	PLXDC2	ENSG00000120594
37	20	59	43	0,69971617	2,120525576	0,006945166	0,043985107	up	HLA-B	ENSG00000234745
184	145	303	286	0,700422942	4,588416091	4,82E-10	2,34E-08	up	CPOX	ENSG00000080819
199	197	171	100	-0,701687072	4,142950531	0,000173104	0,002143646	down	ARL8A	ENSG00000143862

2382	2049	1505	1499	-0,701941821	7,616091689	1,34E-21	2,74E-19	down	DSP	ENSG00000096696
232	208	156	142	-0,703378475	4,287813055	8,58E-09	3,21E-07	down	ZNF804A	ENSG00000170396
354	309	241	208	-0,703917858	4,875240409	2,98E-11	1,75E-09	down	NRP2	ENSG00000118257
170	158	116	106	-0,704544865	3,868152072	5,02E-07	1,30E-05	down	FIBCD1	ENSG00000130720
45	34	68	74	0,705156447	2,57672643	0,000805389	0,007816801	up	STAG3L2	ENSG00000277072
77	55	54	35	-0,706287193	2,579130176	0,001483785	0,012865272	down	BMPER	ENSG00000164619
684	547	1167	1046	0,706467685	6,480807812	1,01E-19	1,68E-17	up	PLCD3	ENSG00000161714
299	272	201	185	-0,706947283	4,660542214	4,24E-10	2,10E-08	down	TDRD7	ENSG00000196116
49	42	89	75	0,707796047	2,776065852	0,000375981	0,004149683	up	MARCH8	ENSG00000165406
42	44	32	26	-0,708049009	1,988913158	0,007236125	0,04547543	down	XIRP2	ENSG00000163092
245	248	178	156	-0,708429606	4,452331334	9,50E-08	2,96E-06	down	LTBP3	ENSG00000168056
60	44	37	33	-0,70851308	2,247953481	0,002566161	0,019865365	down	TMEM144	ENSG00000164124
459	340	740	696	0,70874625	5,860059345	7,26E-16	8,68E-14	up	RAD51AP1	ENSG00000111247
70	44	100	105	0,708788391	3,089295737	0,000111772	0,001492437	up	CA8	ENSG00000178538
193	173	301	358	0,708888837	4,748138975	1,48E-07	4,45E-06	up	PNMA2	ENSG00000240694
74	46	118	98	0,709274724	3,161639508	8,98E-05	0,00126261	up	LIMD2	ENSG00000136490
943	772	586	569	-0,709490973	6,240267397	9,64E-20	1,63E-17	down	ADPRHL1	ENSG00000153531
53	27	61	82	0,710563078	2,588656151	0,00528255	0,035086709	up	CADPS2	ENSG00000081803
82	58	53	41	-0,712059788	2,658998888	0,000744773	0,007306115	down	NAP1L5	ENSG00000177432
304	291	194	207	-0,712576963	4,7191932	4,83E-09	1,90E-07	down	OBSCN	ENSG00000154358
114	93	194	180	0,712788929	3,937333648	1,09E-07	3,36E-06	up	SEMA4D	ENSG00000187764
81	83	157	140	0,713249695	3,61029462	6,22E-06	0,000123085	up	FAM161B	ENSG00000156050
27	32	55	52	0,714623509	2,181541223	0,004940544	0,033259326	up	C11orf74	ENSG00000166352
73	86	135	154	0,715118894	3,571226245	0,000112115	0,001495728	up	TRIM52	ENSG00000183718
442	357	278	258	-0,715245134	5,137076574	1,28E-13	1,13E-11	down	DSE	ENSG00000111817
259	210	452	397	0,715586539	5,104626794	8,15E-13	6,36E-11	up	THOC6	ENSG00000131652
31	22	45	51	0,715695792	2,032572071	0,005857265	0,038233487	up	SPICE1	ENSG00000163611
509	359	308	272	-0,716319199	5,251317362	2,77E-11	1,65E-09	down	STARD4	ENSG00000164211
188	144	310	291	0,716810638	4,611159295	2,15E-10	1,10E-08	up	MRPS6	ENSG00000243927
1720	1487	1138	1016	-0,717351809	7,143376778	1,39E-21	2,80E-19	down	ELK3	ENSG00000111145
1172	996	766	688	-0,71809908	6,576738822	4,52E-21	8,78E-19	down	TADA3	ENSG00000171148
38	28	72	48	0,719643964	2,336854893	0,003408213	0,02476656	up	ZNF286B	ENSG00000249459

169	139	256	302	0,719916042	4,506622416	1,78E-07	5,24E-06	up	TRIB2	ENSG00000071575
461	370	311	244	-0,722809171	5,190237175	5,04E-12	3,39E-10	down	MFGE8	ENSG00000140545
2053	1611	1313	1129	-0,724014728	7,328056496	5,34E-21	1,02E-18	down	CTSC	ENSG00000109861
1328	1036	806	767	-0,72482056	6,695209674	1,24E-21	2,57E-19	down	ESAM	ENSG00000149564
149	141	260	269	0,725278588	4,427382541	8,68E-09	3,24E-07	up	SH3D21	ENSG00000214193
80	82	45	63	-0,726356172	2,861953067	0,000888711	0,00846155	down	PARP9	ENSG00000138496
225	193	127	151	-0,727265838	4,204378832	9,97E-08	3,08E-06	down	ZSCAN18	ENSG00000121413
112	74	180	159	0,727640864	3,792145099	1,04E-06	2,49E-05	up	INAFM2	ENSG00000259330
652	545	1167	1021	0,728538827	6,4561471	4,63E-20	8,16E-18	up	C8orf33	ENSG00000182307
961	764	1649	1503	0,730675559	6,980067374	2,86E-22	6,52E-20	up	IGFBP3	ENSG00000146674
302	231	501	473	0,731058993	5,29600434	1,19E-14	1,21E-12	up	RAP2B	ENSG00000181467
50	51	36	31	-0,731816366	2,200700748	0,002085814	0,016870106	down	SYTL3	ENSG00000164674
31	23	51	48	0,732221909	2,068654954	0,003404264	0,024749444	up	MRPL53	ENSG00000204822
106	68	67	48	-0,732341907	2,954080169	0,000326473	0,003676434	down	CDKL1	ENSG00000100490
527	466	895	925	0,73304162	6,192939348	2,77E-17	3,95E-15	up	COL6A1	ENSG00000142156
80	46	41	42	-0,733529941	2,50111396	0,00184432	0,015299382	down	SLC4A3	ENSG00000114923
78	45	40	41	-0,734199062	2,467489562	0,002404412	0,018819614	down	ACOT11	ENSG00000162390
226	194	128	150	-0,734299596	4,208494175	4,35E-08	1,41E-06	down	TUBG2	ENSG00000037042
372	266	222	199	-0,735509178	4,80251913	1,74E-10	9,11E-09	down	OSBPL5	ENSG00000021762
1966	1588	1150	1196	-0,736201456	7,281047628	3,48E-21	6,94E-19	down	NAV2	ENSG00000166833
231	183	366	393	0,736574619	4,938935708	4,72E-11	2,66E-09	up	TGFA	ENSG00000163235
23	28	48	46	0,737589126	1,996076121	0,005389855	0,035646958	up	MAP3K15	ENSG00000180815
246	217	155	151	-0,738054842	4,346965529	7,98E-10	3,64E-08	down	VEGFC	ENSG00000150630
147	103	238	221	0,73805648	4,219246927	7,83E-09	2,96E-07	up	LZTFL1	ENSG00000163818
175	191	126	117	-0,739625187	4,015430856	2,26E-06	4,94E-05	down	GREB1L	ENSG00000141449
79	52	37	49	-0,740175682	2,55419877	0,001377397	0,012064072	down	TTC39B	ENSG00000155158
594	457	969	962	0,740637755	6,274100523	8,96E-20	1,53E-17	up	CD82	ENSG00000085117
309	220	496	476	0,741080185	5,289559518	9,48E-14	8,66E-12	up	HERPUD1	ENSG00000051108
243	212	160	140	-0,741739087	4,320233119	8,91E-10	4,05E-08	down	ALDH2	ENSG00000111275
127	139	83	93	-0,742100031	3,558912993	3,30E-05	0,000529936	down	RBPMS	ENSG00000157110
336	288	201	210	-0,74228645	4,772920081	1,15E-11	7,30E-10	down	SH3BP5	ENSG00000131370
101	74	67	48	-0,743667971	2,959681753	9,25E-05	0,001293907	down	ZSWIM5	ENSG00000162415

92	71	58	49	-0,745531655	2,859860816	7,84E-05	0,001133179	down	CSF2RA	ENSG00000198223
182	153	113	107	-0,746182585	3,880408461	4,08E-08	1,34E-06	down	G0S2	ENSG00000123689
1275	987	753	727	-0,748550279	6,622136574	5,95E-22	1,28E-19	down	TTL	ENSG00000114999
55	49	27	41	-0,750212517	2,233220221	0,002798748	0,021231928	down	TCN2	ENSG00000185339
7096	5330	4093	3991	-0,753265034	9,077007635	3,53E-24	9,13E-22	down	DHCR24	ENSG00000116133
173	142	301	285	0,755227735	4,561712915	2,89E-11	1,71E-09	up	LZIC	ENSG00000162441
634	576	405	386	-0,756426525	5,722891951	7,89E-17	1,05E-14	down	EPHB2	ENSG00000133216
46	37	29	25	-0,7572912	1,920187075	0,004544745	0,031283401	down	HS3ST1	ENSG00000002587
96	70	64	44	-0,758015671	2,87996449	0,000160336	0,002016079	down	RHOBTB1	ENSG00000072422
323	230	179	180	-0,758156874	4,588772746	7,33E-10	3,39E-08	down	MAOA	ENSG00000189221
89	77	53	55	-0,758919351	2,881412221	5,27E-05	0,000797937	down	BTBD19	ENSG00000222009
79	43	126	101	0,760467291	3,214319396	9,64E-05	0,001330731	up	NANOS1	ENSG00000188613
371	346	670	673	0,762549693	5,746136264	5,14E-16	6,19E-14	up	USP28	ENSG00000048028
299	257	513	527	0,763291963	5,379709768	3,93E-15	4,18E-13	up	PGPEP1	ENSG00000130517
321	277	205	183	-0,764936334	4,703006914	2,45E-12	1,78E-10	down	GPC6	ENSG00000183098
882	771	495	575	-0,766976878	6,167869873	1,13E-14	1,16E-12	down	COL8A1	ENSG00000144810
229	163	125	128	-0,767219833	4,09341888	2,65E-08	8,98E-07	down	TLR4	ENSG00000136869
235	261	181	143	-0,768673277	4,441446645	7,13E-07	1,76E-05	down	NME4	ENSG00000103202
51	42	29	31	-0,769534288	2,071574528	0,002174885	0,017373347	down	OSR1	ENSG00000143867
6263	4713	3633	3430	-0,769930451	8,891845609	6,28E-26	2,12E-23	down	TGM2	ENSG00000198959
79	125	61	73	-0,772983681	3,181056804	0,004539638	0,03126462	down	MTRNR2L10	ENSG00000256045
388	254	650	555	0,774840015	5,584088268	1,30E-12	9,72E-11	up	ZNF687	ENSG00000143373
1008	698	589	503	-0,776176343	6,19959388	3,04E-15	3,34E-13	down	SEMA7A	ENSG00000138623
90	102	60	64	-0,776730972	3,083797632	9,88E-05	0,001352517	down	ECHDC2	ENSG00000121310
35	32	24	19	-0,776827634	1,622996131	0,006864492	0,043580823	down	COL4A4	ENSG00000081052
122	107	63	84	-0,777868882	3,328244263	1,36E-05	0,000242244	down	EPHX4	ENSG00000172031
179	162	112	107	-0,77947239	3,894110169	1,56E-08	5,56E-07	down	TRAF3IP2	ENSG00000056972
111	75	58	61	-0,779644426	3,030782708	3,22E-05	0,000518974	down	BBS5	ENSG00000163093
271	208	157	150	-0,779837433	4,376777635	8,89E-11	4,84E-09	down	ALDH1A3	ENSG00000184254
52	43	84	96	0,781418939	2,882008765	7,31E-05	0,001062524	up	MAGEE1	ENSG00000198934
133	115	226	244	0,782213999	4,239026613	4,07E-09	1,62E-07	up	MPP7	ENSG00000150054
173	129	100	93	-0,783730603	3,716557274	7,88E-08	2,48E-06	down	PLAT	ENSG00000104368

88	75	52	52	-0,786903658	2,845042212	2,97E-05	0,000486871	down	TMEM80	ENSG00000177042
228	158	123	122	-0,790615957	4,061861763	2,32E-08	8,00E-07	down	PMEPA1	ENSG00000124225
470	380	813	807	0,791656989	6,004028713	7,44E-21	1,41E-18	up	TNFRSF10D	ENSG00000173530
2686	2231	1602	1527	-0,792086938	7,730307531	9,60E-30	4,66E-27	down	LIMCH1	ENSG00000064042
100	53	42	54	-0,793834033	2,745287395	0,001680862	0,014232336	down	CHST4	ENSG00000140835
145	112	255	236	0,793998269	4,295748113	8,27E-11	4,52E-09	up	C21orf91	ENSG00000154642
35	36	20	25	-0,795508123	1,695199001	0,007827391	0,048292234	down	MILR1	ENSG00000271605
558	520	1028	1039	0,79574843	6,352762266	2,92E-20	5,20E-18	up	DDX58	ENSG00000107201
214	180	123	126	-0,801384064	4,090961485	5,57E-10	2,65E-08	down	COL4A5	ENSG00000188153
185	174	118	109	-0,803084892	3,959309147	4,74E-09	1,88E-07	down	DGKA	ENSG00000065357
32	23	51	55	0,804742174	2,13855841	0,001302264	0,011490264	up	GNG7	ENSG00000176533
140	108	71	85	-0,805613756	3,428984578	1,36E-06	3,12E-05	down	HTRA1	ENSG00000166033
50	47	37	24	-0,808598444	2,115855951	0,001214215	0,010817548	down	EVA1C	ENSG00000166979
873	666	498	468	-0,808600523	6,04344201	8,22E-22	1,75E-19	down	TSEN15	ENSG00000198860
856	786	559	478	-0,808863864	6,144401697	7,60E-20	1,31E-17	down	EVC	ENSG00000072840
50	43	102	78	0,809754945	2,87095489	5,28E-05	0,000797937	up	AP4S1	ENSG00000100478
465	406	274	273	-0,811999873	5,226505694	6,78E-17	9,15E-15	down	SNAI2	ENSG00000019549
3635	2711	2006	1956	-0,812637628	8,084809665	4,32E-26	1,52E-23	down	TNC	ENSG00000041982
226	170	125	123	-0,812641197	4,091931467	6,62E-10	3,10E-08	down	MAP3K21	ENSG00000143674
1578	1229	940	818	-0,813182297	6,909548358	6,07E-25	1,81E-22	down	RAB14	ENSG00000119396
226	172	120	129	-0,813703495	4,098927259	1,09E-09	4,81E-08	down	TMTC4	ENSG00000125247
159	138	83	103	-0,81388072	3,683709459	2,49E-07	7,05E-06	down	SRPX2	ENSG00000102359
112	94	73	56	-0,814760256	3,163967347	2,20E-06	4,81E-05	down	C16orf74	ENSG00000154102
157	112	87	81	-0,81597086	3,539306515	2,02E-07	5,84E-06	down	CSPG4	ENSG00000173546
30	21	44	55	0,816253349	2,041684091	0,002152148	0,017280613	up	ACBD7	ENSG00000176244
334	258	193	176	-0,820237371	4,665050712	1,83E-13	1,59E-11	down	IMPA1	ENSG00000133731
149	121	280	247	0,824049005	4,386009629	8,23E-12	5,39E-10	up	IER5L	ENSG00000188483
55	32	86	84	0,827506669	2,785901317	4,59E-05	0,000705668	up	PPM1H	ENSG00000111110
71	47	34	39	-0,827655591	2,376947243	0,000427035	0,004611564	down	SLC29A4	ENSG00000164638
123	90	71	61	-0,827889635	3,20481248	1,52E-06	3,45E-05	down	PAPPA	ENSG00000182752
51	30	23	27	-0,828301494	1,858636858	0,00333727	0,024361302	down	SWSAP1	ENSG00000173928
128	92	74	62	-0,83115397	3,248991188	1,48E-06	3,38E-05	down	RASSF5	ENSG00000266094

724	621	425	408	-0,832167691	5,844856547	1,91E-22	4,49E-20	down	TSG101	ENSG00000074319
67	56	37	39	-0,832355972	2,434721502	0,000157399	0,001989265	down	KCNK1	ENSG00000135750
72	56	39	40	-0,833683806	2,489171283	9,06E-05	0,00127315	down	ARMH4	ENSG00000139971
43	30	31	14	-0,834251408	1,71680309	0,006052832	0,039262503	down	KBTBD3	ENSG00000182359
38	35	21	24	-0,834641679	1,7180328	0,00303343	0,022549609	down	ITGB3	ENSG00000259207
54	37	29	27	-0,83627936	2,016170086	0,001452775	0,012631659	down	CYTH4	ENSG00000100055
161	150	104	88	-0,837499179	3,741425696	1,39E-08	4,97E-07	down	SLC37A1	ENSG00000160190
1611	1498	985	940	-0,837710511	7,057294042	1,71E-24	4,91E-22	down	TPP1	ENSG00000166340
74	53	38	40	-0,839875533	2,475367556	0,000141249	0,001815284	down	ALPK1	ENSG00000073331
78	78	49	47	-0,84086165	2,764906998	3,14E-05	0,000508759	down	SMPDL3A	ENSG00000172594
24	27	44	57	0,841740322	2,060649295	0,001894388	0,015639527	up	REEP1	ENSG00000068615
1648	1480	994	934	-0,842475678	7,062484161	2,34E-27	9,10E-25	down	LAPTM5	ENSG00000162511
30	38	72	63	0,843526009	2,459272784	0,000341591	0,003818985	up	AUH	ENSG00000148090
122	103	73	65	-0,844387213	3,277760826	3,04E-07	8,40E-06	down	NINJ2	ENSG00000171840
318	251	575	552	0,846844032	5,464392305	1,64E-20	3,04E-18	up	CASP3	ENSG00000164305
132	165	327	269	0,847099453	4,550660179	1,89E-07	5,52E-06	up	HMGN5	ENSG00000198157
69	65	40	42	-0,847334276	2,549309254	5,35E-05	0,000806227	down	SPACA9	ENSG00000165698
121	114	78	66	-0,847656492	3,339519682	3,22E-07	8,84E-06	down	HOXB3	ENSG00000120093
205	157	365	353	0,848936854	4,819136868	3,05E-15	3,34E-13	up	SFXN2	ENSG00000156398
3819	2963	2132	2006	-0,848946007	8,169890052	8,14E-33	4,31E-30	down	COL12A1	ENSG00000111799
54	54	30	36	-0,849333042	2,249593756	0,000355851	0,003952792	down	FBXL13	ENSG00000161040
208	153	113	107	-0,851732507	3,94490975	7,85E-10	3,60E-08	down	RPS6KA2	ENSG00000071242
75	57	144	119	0,853382376	3,389748894	2,49E-07	7,05E-06	up	LHX4	ENSG00000121454
55	34	26	28	-0,854550049	1,978257368	0,001605767	0,013723696	down	MICA	ENSG00000235233
224	158	126	106	-0,855679844	4,023012947	1,92E-09	8,02E-08	down	PKIA	ENSG00000171033
42	34	22	24	-0,860471546	1,762837328	0,001901177	0,015656993	down	TMEM187	ENSG00000177854
43	46	32	22	-0,861445649	1,979609144	0,001388791	0,012157012	down	RORB	ENSG00000198963
832	673	477	435	-0,862012412	5,993790571	1,54E-25	4,88E-23	down	CLMP	ENSG00000166250
50	28	24	23	-0,862661013	1,794745839	0,003401196	0,02473872	down	GTF2H2C_2	ENSG00000274675
54	47	32	29	-0,864993459	2,150354668	0,000303022	0,003465114	down	NLGN1	ENSG00000169760
98	84	55	55	-0,865087669	2,971052298	1,91E-06	4,26E-05	down	IL31RA	ENSG00000164509
19	26	51	40	0,868280871	1,90835546	0,002266157	0,017963835	up	CCDC113	ENSG00000103021

30	29	64	55	0,868794241	2,276827575	0,000266637	0,00310469	up	IQCG	ENSG00000114473
1676	1341	943	875	-0,869258243	6,994663268	2,59E-31	1,30E-28	down	RGMB	ENSG00000174136
70	53	37	37	-0,870189788	2,420321598	8,32E-05	0,001188492	down	ACOX2	ENSG00000168306
280	209	157	137	-0,871640645	4,371282395	1,60E-12	1,19E-10	down	ALS2CL	ENSG00000178038
596	532	345	334	-0,874393391	5,576887548	9,77E-22	2,05E-19	down	FTO	ENSG00000140718
80	75	37	56	-0,875456388	2,74278096	7,30E-05	0,001062524	down	MYOM3	ENSG00000142661
909	734	512	471	-0,880261182	6,113696478	3,69E-28	1,47E-25	down	AC034102.1	ENSG00000111540
27	30	14	20	-0,882130606	1,370299206	0,007211079	0,045373113	down	BDH1	ENSG00000275544
126	107	71	68	-0,884244267	3,312719707	4,96E-08	1,60E-06	down	OPN3	ENSG00000054277
654	542	376	338	-0,884463011	5,654991723	2,83E-24	7,57E-22	down	FSTL3	ENSG00000070404
755	536	1339	1282	0,887993959	6,659980264	2,87E-26	1,06E-23	up	JADE2	ENSG00000043143
53	42	102	92	0,888436285	2,950612774	1,68E-06	3,78E-05	up	PARD6A	ENSG00000102981
188	154	390	309	0,889001769	4,765695194	2,82E-13	2,38E-11	up	GABPB2	ENSG00000143458
70	68	38	44	-0,890144693	2,575236057	3,00E-05	0,000489403	down	FKBP7	ENSG00000079150
46	40	25	26	-0,890599098	1,920748164	0,000641405	0,006480406	down	SOD3	ENSG00000109610
819	799	1710	1620	0,893514369	7,002116138	4,74E-25	1,44E-22	up	SKA2	ENSG00000182628
254	225	143	141	-0,894688063	4,336887812	2,20E-13	1,89E-11	down	PLPP3	ENSG00000162407
36	30	24	15	-0,89510874	1,560301784	0,003482539	0,025200537	down	AADACP1	ENSG00000240602
33	33	14	25	-0,895176056	1,561257752	0,004992681	0,033490861	down	CRYZL2P	ENSG00000242193
1876	4361	2048	1824	-0,895724474	8,096641086	0,003837758	0,027312808	down	MT-ATP8	ENSG00000228253
4445	3396	2304	2309	-0,89914662	8,360538622	7,85E-33	4,31E-30	down	SCD	ENSG00000099194
205	176	121	104	-0,900006518	4,006660593	1,11E-11	7,09E-10	down	ARHGAP22	ENSG00000128805
36	37	20	23	-0,900726615	1,695295551	0,002423804	0,018940696	down	FAM86B1	ENSG00000186523
811	591	403	420	-0,901569739	5,873092997	1,15E-20	2,16E-18	down	LRP1	ENSG00000123384
85	68	37	53	-0,901593256	2,713473188	2,30E-05	0,000390141	down	CLDN7	ENSG00000181885
66	58	35	38	-0,902183701	2,421078053	4,72E-05	0,000722886	down	DENND6B	ENSG00000205593
21	24	44	49	0,902729087	1,928332413	0,001105853	0,010054324	up	TUBB4A	ENSG00000104833
84	62	161	140	0,903167109	3,564247595	6,24E-09	2,43E-07	up	AKTIP	ENSG00000166971
45	46	87	101	0,904165355	2,90256528	1,03E-05	0,000191545	up	LHFPL4	ENSG00000156959
38	50	22	30	-0,904446705	1,95177661	0,002162846	0,017321365	down	LIPH	ENSG00000163898
360	295	672	680	0,906445319	5,705648699	1,66E-23	4,08E-21	up	SCFD1	ENSG00000092108
57	41	111	92	0,909323128	3,007091024	9,44E-07	2,28E-05	up	SRRM3	ENSG00000177679

1062	923	2111	2004	0,909593032	7,300716093	6,36E-35	4,30E-32	up	NRG1	ENSG00000157168
237	175	432	420	0,910130735	5,042993093	1,48E-18	2,28E-16	up	CCDC90B	ENSG00000137500
156	141	95	79	-0,912181435	3,647933044	1,31E-09	5,68E-08	down	C6orf132	ENSG00000188112
78	79	55	37	-0,914243157	2,748001169	1,44E-05	0,000256082	down	TFAP2A	ENSG00000137203
78	35	36	29	-0,917048195	2,277927006	0,001902553	0,015656993	down	TMEM184A	ENSG00000164855
579	454	287	314	-0,917837094	5,430104094	2,67E-20	4,82E-18	down	LZTS2	ENSG00000107816
433	335	229	218	-0,918692213	5,002826142	3,53E-19	5,83E-17	down	ALDH3B1	ENSG00000006534
1091	804	522	576	-0,919063581	6,301619304	3,65E-22	8,10E-20	down	F2RL1	ENSG00000164251
753	783	478	424	-0,920741002	6,013083614	6,61E-17	9,00E-15	down	ATP5MG	ENSG00000167283
63	54	38	30	-0,920745049	2,333585154	4,85E-05	0,000739808	down	KIAA1755	ENSG00000149633
1220	1048	689	632	-0,921737387	6,56706878	1,97E-33	1,18E-30	down	ANKRD13A	ENSG00000076513
41	40	26	21	-0,922750305	1,828414798	0,000637078	0,006445072	down	PCDHB2	ENSG00000112852
85	72	50	41	-0,925255369	2,741703788	2,51E-06	5,42E-05	down	GPR153	ENSG00000158292
195	187	119	103	-0,926026019	4,003287669	3,82E-11	2,19E-09	down	FABP5	ENSG00000164687
962	753	1746	1835	0,926764804	7,096871405	5,88E-29	2,68E-26	up	SLC20A1	ENSG00000144136
303	208	156	139	-0,926847613	4,412064603	2,53E-12	1,84E-10	down	FSCN1	ENSG00000075618
66	55	34	36	-0,926960985	2,377968946	3,27E-05	0,000525257	down	CYP2J2	ENSG00000134716
41	30	21	20	-0,927308296	1,647021111	0,001707199	0,014416035	down	AL513477.1	ENSG00000269896
37	39	90	70	0,929041815	2,667829145	1,80E-05	0,000314723	up	TIMP4	ENSG00000157150
842	640	1593	1509	0,929078193	6,88780643	8,33E-33	4,31E-30	up	MAP3K14	ENSG00000006062
111	72	174	208	0,929494263	3,896494868	3,07E-08	1,03E-06	up	RNF182	ENSG00000180537
36	30	19	19	-0,931750505	1,547749863	0,002118684	0,017064673	down	PALM2-AKAP2	ENSG00000157654
2271	1741	1147	1160	-0,932760469	7,381495007	8,31E-33	4,31E-30	down	HSPG2	ENSG00000142798
16	10	29	26	0,935092926	1,213769021	0,005888751	0,038358392	up	AKR1E2	ENSG00000165568
45	28	90	64	0,935917146	2,612246002	3,33E-05	0,000533623	up	NEK11	ENSG00000114670
466	401	916	917	0,939634261	6,130761096	3,66E-29	1,72E-26	up	EIF4E2	ENSG00000135930
185	148	353	351	0,940502433	4,76101325	1,51E-17	2,18E-15	up	BIN1	ENSG00000136717
169	135	83	91	-0,942966303	3,668491456	2,85E-10	1,44E-08	down	PRDM11	ENSG00000019485
303	266	169	157	-0,944383162	4,565777627	1,32E-16	1,69E-14	down	PDLIM4	ENSG00000131435
134	97	70	62	-0,944434159	3,277029607	2,39E-08	8,20E-07	down	SESN3	ENSG00000149212
99	86	199	194	0,945889672	3,929349404	7,01E-12	4,61E-10	up	FAM234B	ENSG00000084444
32	38	19	21	-0,946318456	1,624040866	0,001820143	0,015147375	down	SNAP91	ENSG00000065609

35	50	29	20	-0,947146972	1,891810778	0,002223772	0,017703507	down	HMCN1	ENSG00000143341
32	26	19	14	-0,948237674	1,369439874	0,003900632	0,027696805	down	NOTCH3	ENSG00000074181
87	81	57	39	-0,94824398	2,829679058	1,77E-06	3,97E-05	down	NREP	ENSG00000134986
117	99	66	57	-0,951308235	3,181436116	2,61E-08	8,89E-07	down	LPXN	ENSG00000110031
303	276	190	141	-0,951468872	4,58953464	1,38E-13	1,21E-11	down	PEAR1	ENSG00000187800
41	34	74	86	0,951862978	2,662056435	8,32E-06	0,000157745	up	IPO5P1	ENSG00000269837
168	150	79	101	-0,960186812	3,728234196	1,84E-09	7,73E-08	down	STC1	ENSG00000159167
30	44	20	22	-0,964824756	1,696257562	0,003451943	0,025037467	down	PTPRB	ENSG00000127329
30	34	17	19	-0,96721089	1,496024126	0,002224582	0,017703507	down	GPR35	ENSG00000178623
997	795	524	485	-0,96723204	6,207658323	2,19E-34	1,36E-31	down	PROCR	ENSG00000101000
46	36	83	94	0,969252975	2,797513436	2,48E-06	5,38E-05	up	VASH1	ENSG00000071246
16	8	28	24	0,969459343	1,130190282	0,00655647	0,042003061	up	GPR19	ENSG00000183150
338	234	578	656	0,978721263	5,553042041	8,26E-17	1,09E-14	up	SDC2	ENSG00000169439
30	31	19	15	-0,979307912	1,427203033	0,002043116	0,016593907	down	ZNF385B	ENSG00000144331
29	25	11	19	-0,981302046	1,264064264	0,004302436	0,029947348	down	SSC5D	ENSG00000179954
4095	3375	2083	2076	-0,983553937	8,265959508	6,16E-44	5,98E-41	down	FN1	ENSG00000115414
221	176	465	402	0,986252884	5,042236228	3,70E-21	7,27E-19	up	HMOX1	ENSG00000100292
33	23	17	14	-0,986506502	1,309687367	0,003214452	0,023612693	down	FDFT1	ENSG00000284967
41	33	20	21	-0,9871696	1,683035837	0,000720296	0,007119972	down	BANK1	ENSG00000153064
32	33	18	18	-0,9885011	1,509097457	0,001462396	0,012701087	down	EBI3	ENSG00000105246
231	189	121	112	-0,989183554	4,113530171	9,87E-15	1,01E-12	down	NES	ENSG00000132688
44	40	88	96	0,989245799	2,845542974	7,00E-07	1,73E-05	up	ACSS1	ENSG00000154930
39	28	21	16	-0,991079602	1,547338718	0,001181822	0,010578486	down	TMSB15B	ENSG00000269226
52	44	27	26	-0,993609498	2,035618662	8,53E-05	0,001211326	down	SELPLG	ENSG00000110876
41	21	18	16	-0,995775507	1,439749337	0,003118177	0,023047199	down	PDE4B	ENSG00000184588
292	238	603	569	1,00487108	5,468099941	2,75E-28	1,13E-25	up	SRPK2	ENSG00000135250
560	430	284	258	-1,006830253	5,336912902	7,95E-26	2,63E-23	down	H6PD	ENSG00000049239
141	93	257	260	1,007226187	4,299707737	3,46E-14	3,30E-12	up	CMTM3	ENSG00000140931
265	318	667	642	1,010881823	5,623695113	1,83E-14	1,84E-12	up	CFDP1	ENSG00000153774
12	9	23	24	1,014283145	0,986321405	0,007741218	0,04785564	up	CATSPERZ	ENSG00000219435
433	333	210	207	-1,014665658	4,96526247	3,35E-22	7,55E-20	down	LTBP2	ENSG00000119681
377	280	796	666	1,015152344	5,779400639	1,28E-25	4,15E-23	up	RSL24D1	ENSG00000137876

79	68	38	42	-1,015446747	2,618628408	7,79E-07	1,91E-05	down	ADGRB2	ENSG00000121753
46	26	20	19	-1,015691067	1,634422119	0,001251652	0,011119507	down	HS6ST3	ENSG00000185352
12	13	29	27	1,016028929	1,214219436	0,004392414	0,030478014	up	SHISAL1	ENSG00000138944
150	133	76	78	-1,017338184	3,542095774	3,78E-11	2,17E-09	down	RASGRF1	ENSG00000058335
153	143	70	91	-1,018701519	3,606606856	1,42E-09	6,10E-08	down	MAST4	ENSG00000069020
143	169	89	82	-1,020337559	3,686883641	4,02E-08	1,33E-06	down	ZNF608	ENSG00000168916
164	139	75	89	-1,024048403	3,636393465	3,64E-11	2,11E-09	down	SLC37A2	ENSG00000134955
1862	1489	907	903	-1,025919472	7,091839608	1,00E-41	8,66E-39	down	AP002956.1	ENSG00000076706
132	135	82	63	-1,02602486	3,459145354	3,51E-09	1,41E-07	down	KLF7	ENSG00000118263
180	169	97	92	-1,026203889	3,838595705	1,08E-12	8,26E-11	down	DNAH11	ENSG00000105877
60	44	26	30	-1,028566396	2,133126028	3,76E-05	0,000594045	down	PRCD	ENSG00000214140
42	38	27	16	-1,032669569	1,774095542	0,000269885	0,003137013	down	GOLGA7B	ENSG00000155265
33	25	17	14	-1,037325219	1,339972996	0,00171482	0,014450124	down	ANKAR	ENSG00000151687
231	206	492	499	1,040206643	5,218226753	2,57E-24	7,00E-22	up	CCNYL1	ENSG00000163249
31	29	14	18	-1,041191928	1,38458345	0,001155582	0,010396892	down	FN3K	ENSG00000167363
126	108	59	66	-1,043071513	3,263254189	7,21E-10	3,35E-08	down	TMEM156	ENSG00000121895
33	29	13	20	-1,043738426	1,427218178	0,001048528	0,009600581	down	VSIG1	ENSG00000101842
22	25	10	15	-1,044606499	1,062716045	0,006595971	0,042221285	down	LRP2BP	ENSG00000109771
463	423	238	236	-1,044870333	5,169437423	3,99E-24	1,02E-21	down	ZBED3	ENSG00000132846
29	20	10	16	-1,04522848	1,115047167	0,005167311	0,034483529	down	FAM13C	ENSG00000148541
276	272	183	112	-1,046939129	4,481048723	5,28E-10	2,51E-08	down	VEPH1	ENSG00000197415
80	65	39	38	-1,050477766	2,587225658	3,30E-07	9,02E-06	down	SLAMF7	ENSG00000026751
41	42	21	23	-1,052640054	1,818272605	0,000124474	0,001638105	down	CDK18	ENSG00000117266
4143	3356	2088	1890	-1,054131889	8,246897912	2,76E-52	3,06E-49	down	ANKRD52	ENSG00000139645
185	157	116	66	-1,054846081	3,79913015	7,61E-09	2,90E-07	down	PDE2A	ENSG00000186642
14	15	37	30	1,061001755	1,438616516	0,001299177	0,011476066	up	CFAP58	ENSG00000120051
27	30	15	15	-1,061321748	1,310769737	0,00131039	0,011548839	down	SEMA6A	ENSG00000092421
274	223	132	130	-1,062514036	4,329332124	2,10E-18	3,19E-16	down	FGF5	ENSG00000138675
29	30	16	15	-1,063351201	1,35550674	0,001026216	0,009440818	down	GFRA1	ENSG00000151892
708	602	357	334	-1,063562045	5,724952641	2,15E-34	1,36E-31	down	KRT81	ENSG00000205426
113	87	45	60	-1,065356558	3,032670972	2,36E-08	8,12E-07	down	TTYH2	ENSG00000141540
308	266	153	149	-1,066646139	4,535756531	1,84E-20	3,36E-18	down	SRPX	ENSG00000101955

209	208	112	108	-1,066674389	4,080854451	9,92E-14	9,01E-12	down	SPEG	ENSG00000072195
47	37	19	25	-1,067684694	1,828297894	0,000110256	0,001475998	down	THEMIS2	ENSG00000130775
61	44	31	24	-1,068621591	2,132846655	1,92E-05	0,000331709	down	AC118754.1	ENSG00000183018
34	31	18	16	-1,069564121	1,482109397	0,000553116	0,005710994	down	EGR1	ENSG00000120738
99	61	32	51	-1,071431958	2,712648528	1,87E-05	0,000325053	down	ABCG2	ENSG00000118777
221	202	121	101	-1,07193377	4,097429867	1,86E-15	2,08E-13	down	PLEKHA6	ENSG00000143850
45	28	18	20	-1,074168721	1,63478763	0,000521391	0,005455985	down	CDK5R2	ENSG00000171450
3933	3486	1997	1896	-1,074412467	8,230209577	8,81E-50	9,12E-47	down	ANXA5	ENSG00000164111
41	32	18	20	-1,076308672	1,635350813	0,000286199	0,003292133	down	FGF13	ENSG00000129682
132	93	59	58	-1,079243218	3,19322569	1,38E-09	5,95E-08	down	B4GALT4	ENSG00000121578
42	22	15	18	-1,083313029	1,453807208	0,0012812	0,011330159	down	NOXA1	ENSG00000188747
242	184	122	99	-1,084850393	4,099715802	8,05E-16	9,37E-14	down	SOCS2	ENSG00000120833
13	15	34	32	1,089782826	1,410794677	0,001111038	0,010095556	up	CCDC146	ENSG00000135205
260	232	545	609	1,089895617	5,421719057	2,44E-22	5,66E-20	up	SYNJ2BP	ENSG00000213463
44	17	17	14	-1,092559149	1,382695747	0,004858561	0,032875207	down	UBA7	ENSG00000182179
51	29	29	12	-1,094431711	1,750545726	0,000972504	0,009053966	down	DEPDC7	ENSG00000121690
135	122	57	75	-1,100152043	3,377790436	4,61E-10	2,26E-08	down	SECTM1	ENSG00000141574
716	515	1461	1435	1,100427471	6,734416641	1,58E-39	1,29E-36	up	CACUL1	ENSG00000151893
27	20	13	11	-1,10097512	1,043694274	0,002198888	0,017556056	down	TCAF2	ENSG00000170379
29	20	14	11	-1,102227516	1,097348914	0,001935752	0,015904916	down	ZNF563	ENSG00000188868
133	132	75	61	-1,105404232	3,420926708	1,02E-10	5,47E-09	down	STEAP1	ENSG00000164647
58	56	27	31	-1,112449128	2,233965221	4,39E-06	9,11E-05	down	MDK	ENSG00000110492
39	32	17	19	-1,114044019	1,586144917	0,000214223	0,002570846	down	FILIP1L	ENSG00000168386
67	48	23	35	-1,120833719	2,241040164	9,01E-06	0,000169759	down	ICAM1	ENSG00000090339
139	114	64	64	-1,121233073	3,347239972	4,76E-12	3,25E-10	down	NMNAT2	ENSG00000157064
20	17	44	45	1,121347565	1,803855618	7,14E-05	0,001041563	up	RGS9	ENSG00000108370
53	46	26	24	-1,12170041	2,035882486	9,92E-06	0,000184679	down	RBM43	ENSG00000184898
593	411	1253	1145	1,121918651	6,455942524	1,58E-37	1,11E-34	up	SDC4	ENSG00000124145
647	659	357	309	-1,122689335	5,708468438	1,31E-23	3,27E-21	down	IFI16	ENSG00000163565
191	184	92	98	-1,122906135	3,909508774	2,99E-14	2,88E-12	down	FGF1	ENSG00000113578
173	132	81	73	-1,123617281	3,610800839	1,31E-13	1,16E-11	down	SH3PXD2B	ENSG00000174705
399	326	195	171	-1,125692293	4,85038112	2,63E-26	9,95E-24	down	ELFN2	ENSG00000166897

29	15	10	12	-1,126811186	0,948907779	0,00635208	0,040930064	down	AKR1C3	ENSG00000196139
21	19	10	10	-1,129998893	0,82761877	0,005035739	0,033706889	down	SCN9A	ENSG00000169432
12	11	26	30	1,135794668	1,181360745	0,001211473	0,01080584	up	SYT7	ENSG00000011347
161	121	329	355	1,14102716	4,658579897	5,26E-20	9,18E-18	up	BIRC3	ENSG00000023445
59	69	35	29	-1,144268825	2,386894957	2,58E-06	5,57E-05	down	HOXC6	ENSG00000197757
40	35	17	20	-1,153861141	1,647848795	0,000100438	0,001368329	down	PCDHGB1	ENSG00000254221
36	25	18	12	-1,156293255	1,369284523	0,000486165	0,005160396	down	MPZL3	ENSG00000160588
108	73	43	46	-1,158375249	2,860486852	1,19E-08	4,34E-07	down	ORAI3	ENSG00000175938
316	284	160	136	-1,161381637	4,569063209	1,45E-22	3,46E-20	down	B3GALT5	ENSG00000183778
26	19	15	7	-1,16294889	0,968548068	0,001826712	0,01517764	down	ADAMTS3	ENSG00000156140
222	180	87	110	-1,16588265	3,991670232	1,92E-15	2,13E-13	down	GALNT6	ENSG00000139629
79	64	36	34	-1,167508076	2,529755295	4,10E-08	1,35E-06	down	PLA2G4A	ENSG00000116711
47	35	18	22	-1,169499553	1,763056782	5,04E-05	0,000765436	down	NLRP3	ENSG00000162711
40	32	20	15	-1,174696775	1,585963332	9,98E-05	0,00136365	down	PSTPIP1	ENSG00000140368
66	60	35	26	-1,184179963	2,349223472	2,51E-07	7,08E-06	down	NCF2	ENSG00000116701
12	13	28	35	1,185599226	1,32346659	0,000881718	0,008408133	up	SALL2	ENSG00000165821
27	27	12	14	-1,187087381	1,200190357	0,00063578	0,006436129	down	IFFO1	ENSG00000010295
884	766	2326	1833	1,187623133	7,223549207	5,34E-38	3,95E-35	up	RABL6	ENSG00000196642
90	76	211	207	1,1912242	3,942713846	8,59E-18	1,27E-15	up	DUSP22	ENSG00000112679
50	52	22	27	-1,195397657	2,055104184	4,08E-06	8,52E-05	down	B3GNT3	ENSG00000179913
37	52	14	29	-1,197675749	1,872339201	0,000866995	0,008290375	down	SLC40A1	ENSG00000138449
1323	1147	3174	3082	1,19897127	7,810728547	4,20E-61	5,43E-58	up	CDK6	ENSG00000105810
61	46	19	32	-1,202152015	2,116433099	4,67E-06	9,60E-05	down	MARC1	ENSG00000186205
199	179	94	87	-1,20270062	3,893654428	8,92E-18	1,31E-15	down	CA12	ENSG00000074410
64	39	25	24	-1,203667777	2,062169361	6,87E-06	0,000134103	down	FAM86C2P	ENSG00000160172
18	7	35	29	1,210502277	1,337183835	0,000420057	0,004542527	up	SORBS1	ENSG00000095637
97	93	46	44	-1,21739807	2,913687252	2,09E-10	1,07E-08	down	DENND1C	ENSG00000205744
36	30	19	12	-1,22365953	1,45468279	0,000105499	0,001425843	down	ABCC9	ENSG00000069431
614	535	1576	1392	1,225980407	6,730603802	3,99E-55	4,77E-52	up	ARPC3	ENSG00000111229
34	26	14	14	-1,231480252	1,325287479	0,000216894	0,002599113	down	PTPN22	ENSG00000134242
72	50	35	22	-1,232780771	2,287561875	4,25E-07	1,12E-05	down	FAM160A1	ENSG00000164142
115	87	47	47	-1,240392975	2,990506239	1,69E-11	1,05E-09	down	LOXL4	ENSG00000138131

360	299	143	163	-1,24508374	4,675841508	6,04E-26	2,08E-23	down	CLU	ENSG00000120885
39	24	20	9	-1,249715292	1,38348449	0,0002844	0,003276037	down	ACADS	ENSG00000122971
55	54	24	26	-1,261216052	2,125892202	4,92E-07	1,28E-05	down	BCHE	ENSG00000114200
133	96	63	42	-1,261458011	3,160035104	2,05E-11	1,25E-09	down	SPOCD1	ENSG00000134668
27	28	15	10	-1,270360353	1,20014885	0,000289412	0,003321715	down	PLA2R1	ENSG00000153246
12	19	9	5	-1,277932185	0,468426779	0,007136669	0,044996077	down	AC012435.1	ENSG00000260103
12	4	17	26	1,279975938	0,804248429	0,004252411	0,029638996	up	LRRIQ1	ENSG00000133640
127	89	294	284	1,281122112	4,377576346	2,89E-24	7,60E-22	up	PIM1	ENSG00000137193
166	143	76	64	-1,281419353	3,581125632	4,71E-17	6,53E-15	down	HIST1H2AC	ENSG00000180573
112	83	49	39	-1,284506363	2,927067456	1,80E-11	1,11E-09	down	MSLN	ENSG00000102854
8	6	22	16	1,285980884	0,645145345	0,005343706	0,03540205	up	SLC5A4	ENSG00000100191
8	6	22	16	1,28598107	0,645145345	0,00534282	0,03540205	up	FOXD3	ENSG00000187140
58	60	29	24	-1,293283056	2,226294745	1,88E-07	5,50E-06	down	CACNB4	ENSG00000182389
274	232	114	113	-1,295759593	4,281311764	1,72E-25	5,36E-23	down	TMEM154	ENSG00000170006
3361	2952	1474	1344	-1,307789977	7,924231583	2,74E-71	4,25E-68	down	SMS	ENSG00000102172
610	545	270	244	-1,31063542	5,466656727	1,37E-42	1,25E-39	down	CD163L1	ENSG00000177675
9	4	22	14	1,314272146	0,570892618	0,006986278	0,044173413	up	TVP23C-CDRT4	ENSG00000259024
32	25	15	10	-1,320013265	1,232084698	0,000125463	0,001646933	down	CPNE5	ENSG00000124772
297	285	882	731	1,323637448	5,829076655	3,90E-38	3,03E-35	up	COX7C	ENSG00000127184
179	177	76	81	-1,323780772	3,772902049	7,48E-17	1,00E-14	down	SERINC2	ENSG00000168528
37	27	19	9	-1,324450409	1,383942003	7,82E-05	0,00113113	down	TM4SF19	ENSG00000145107
34	30	16	12	-1,325026023	1,384529189	3,88E-05	0,000610175	down	PLAAT1	ENSG00000127252
90	63	40	27	-1,32644078	2,574154797	3,23E-09	1,31E-07	down	PRKCH	ENSG00000027075
110	93	40	49	-1,326836684	2,972519786	2,86E-12	2,04E-10	down	B3GNT5	ENSG00000176597
91	81	41	34	-1,335200344	2,737389876	2,84E-11	1,69E-09	down	WDR66	ENSG00000158023
31	20	7	15	-1,339283107	1,079987125	0,000513452	0,005393334	down	TSPAN1	ENSG00000117472
215	186	82	91	-1,351835387	3,932111604	5,15E-22	1,13E-19	down	AMPD3	ENSG00000133805
35	28	14	13	-1,353803807	1,355240371	4,25E-05	0,000658261	down	PKIB	ENSG00000135549
244	206	93	100	-1,360120636	4,094147839	2,40E-24	6,65E-22	down	IL18	ENSG00000150782
240	156	98	71	-1,360545655	3,905644424	1,36E-15	1,54E-13	down	MYO7B	ENSG00000169994
27	9	5	10	-1,366184517	0,621769068	0,006885241	0,04369469	down	ATP6V1C2	ENSG00000143882
12	21	5	9	-1,370062151	0,52231904	0,004852156	0,032846177	down	ADGRG2	ENSG00000173698

27	16	10	8	-1,382001138	0,848173752	0,000996166	0,009235498	down	GAREM2	ENSG00000157833
8	4	18	17	1,387166325	0,519614773	0,003467072	0,025112013	up	SLC22A20P	ENSG00000197847
235	199	85	96	-1,400301081	4,030769025	2,29E-24	6,47E-22	down	EFEMP1	ENSG00000115380
143	104	62	40	-1,412306451	3,222571626	4,16E-14	3,92E-12	down	LOXL1	ENSG00000129038
104	108	52	35	-1,429523177	3,006929935	3,57E-12	2,48E-10	down	PRR16	ENSG00000184838
10	15	29	46	1,434272864	1,493559707	0,000117391	0,001562091	up	RIMKLA	ENSG00000177181
49	59	20	24	-1,436973592	2,065084623	2,85E-07	7,94E-06	down	CDA	ENSG00000158825
1152	943	427	423	-1,44027235	6,287093485	3,58E-71	5,05E-68	down	ADAMTS1	ENSG00000154734
71	58	24	28	-1,445946159	2,304400511	9,59E-10	4,31E-08	down	MR1	ENSG00000153029
20	17	66	46	1,450391238	2,031138829	1,68E-07	4,96E-06	up	MAGEH1	ENSG00000187601
6	9	30	16	1,456594596	0,846707801	0,000969241	0,009041396	up	CDK20	ENSG00000156345
29	14	7	10	-1,458831767	0,826993137	0,000806643	0,007824084	down	CDC42BPG	ENSG00000171219
60	38	23	16	-1,460556958	1,920313754	1,92E-07	5,59E-06	down	PTGES	ENSG00000148344
131	123	366	407	1,464388442	4,745424025	1,26E-28	5,27E-26	up	RAB15	ENSG00000139998
13	10	5	4	-1,467009133	0,061603621	0,007488281	0,046626106	down	PRDM6	ENSG00000061455
19	19	51	66	1,47728589	2,08624979	1,82E-07	5,36E-06	up	ZNF391	ENSG00000124613
18	13	5	7	-1,488683482	0,412291406	0,002157525	0,017298631	down	SYNC	ENSG00000162520
30	29	11	12	-1,489207467	1,232826942	1,82E-05	0,000317448	down	SERPINB7	ENSG00000166396
16	15	5	7	-1,489286483	0,412582465	0,002165097	0,017321895	down	CLIC3	ENSG00000169583
18	18	59	53	1,490784792	2,022446701	4,22E-08	1,38E-06	up	CDH11	ENSG00000140937
17	9	5	5	-1,492881158	0,198869344	0,004585827	0,031469647	down	AL390726.5	ENSG00000283162
14	12	2	8	-1,493795426	0,199473619	0,004723637	0,032200774	down	ENTPD3	ENSG00000168032
1104	891	414	365	-1,496007679	6,200299313	7,40E-73	1,28E-69	down	AOX1	ENSG00000138356
8	5	20	21	1,500503331	0,69242882	0,000948699	0,008953342	up	C19orf57	ENSG00000132016
33	32	13	12	-1,509605645	1,355818585	4,59E-06	9,46E-05	down	BEST3	ENSG00000127325
25	22	12	6	-1,511765819	0,930115412	0,00014486	0,001854578	down	FAS	ENSG00000026103
17	7	5	4	-1,524095509	0,09676019	0,005960355	0,03875172	down	CTSS	ENSG00000163131
19	18	7	7	-1,524932204	0,622940771	0,000484189	0,005142939	down	MPZ	ENSG00000158887
221	171	81	68	-1,533104534	3,846626252	3,68E-26	1,33E-23	down	KCNQ3	ENSG00000184156
51	39	20	14	-1,536924645	1,785350184	1,13E-07	3,47E-06	down	SORCS2	ENSG00000184985
45	43	15	18	-1,54832445	1,753070779	1,00E-07	3,09E-06	down	NRK	ENSG00000123572
25	26	14	5	-1,554644005	1,026038795	8,74E-05	0,00123567	down	SPANXB1	ENSG00000227234

54	30	15	16	-1,563938416	1,682658883	9,95E-07	2,40E-05	down	CA9	ENSG00000107159
13	9	7	1	-1,568530281	-0,012620117	0,006289978	0,040580416	down	SPTLC3	ENSG00000172296
192	173	81	54	-1,576798352	3,735704748	1,29E-22	3,12E-20	down	S100A2	ENSG00000196754
21	7	3	7	-1,585591338	0,262761588	0,005681722	0,037212761	down	CYSRT1	ENSG00000197191
13	12	4	5	-1,586342641	0,132283298	0,003202742	0,023571273	down	TUB	ENSG00000166402
14	14	6	4	-1,600857092	0,263616544	0,001863137	0,015414304	down	HTR2C	ENSG00000147246
109	79	38	30	-1,602174262	2,786923083	5,04E-15	5,33E-13	down	PRLR	ENSG00000113494
20	35	10	10	-1,60592608	1,117062326	0,000191378	0,002341935	down	INHBA	ENSG00000122641
19	12	7	4	-1,611180706	0,383472063	0,001136301	0,010252505	down	CRABP2	ENSG00000143320
80	64	27	24	-1,632363731	2,407743306	7,71E-13	6,05E-11	down	IL1A	ENSG00000115008
6	9	27	25	1,637268607	0,967135638	4,79E-05	0,000731842	up	IL17RE	ENSG00000163701
57	51	21	17	-1,640850255	2,00882588	4,78E-10	2,33E-08	down	LAMA4	ENSG00000112769
69	45	20	20	-1,641853429	2,080929225	5,75E-10	2,71E-08	down	ZNF415	ENSG00000170954
20	23	6	9	-1,643998732	0,785290671	0,000107381	0,001446388	down	PDCD1LG2	ENSG00000197646
15	14	4	6	-1,65068489	0,294725236	0,001206376	0,010766556	down	ROBO2	ENSG00000185008
20	12	2	9	-1,653798031	0,412288544	0,000949588	0,008953342	down	COL4A6	ENSG00000197565
6	3	14	18	1,665133846	0,353138774	0,001759494	0,014733151	up	KDR	ENSG00000128052
9	10	27	40	1,666709665	1,292863843	1,83E-05	0,000319429	up	LONRF2	ENSG00000170500
62	50	22	16	-1,692646075	2,045516271	8,99E-11	4,88E-09	down	NGF	ENSG00000134259
13	14	4	5	-1,696826497	0,19963186	0,001370559	0,012017735	down	P2RX5-TAX1BP3	ENSG00000257950
15	15	2	8	-1,699242916	0,325326354	0,000910214	0,008639798	down	ZC3H6	ENSG00000188177
7135	6086	2275	2154	-1,719630563	8,883095213	1,57E-137	1,22E-133	down	F3	ENSG00000117525
22	18	3	10	-1,740483667	0,671174177	8,18E-05	0,001171377	down	ARHGAP24	ENSG00000138639
7	5	25	20	1,746870558	0,760334786	9,61E-05	0,00132798	up	TRIM73	ENSG00000178809
11	14	4	4	-1,752536186	0,097816276	0,00158644	0,013573456	down	HSPB8	ENSG00000152137
10	12	5	2	-1,756210834	-0,050537818	0,002733265	0,020847184	down	AIFM3	ENSG00000183773
62	49	17	19	-1,75676668	2,018022983	2,91E-11	1,72E-09	down	TRPM2	ENSG00000142185
139	79	33	37	-1,761508378	2,951401106	1,15E-13	1,04E-11	down	CPA4	ENSG00000128510
66	65	20	22	-1,776421664	2,243179657	1,06E-12	8,22E-11	down	ALOXE3	ENSG00000179148
106	104	36	30	-1,808873214	2,895057962	9,77E-19	1,53E-16	down	ADGRG4	ENSG00000156920
79	71	25	22	-1,809190064	2,42279628	6,74E-15	7,02E-13	down	SLC4A4	ENSG00000080493
23	19	6	7	-1,811191773	0,717639315	2,63E-05	0,000438546	down	GPC5	ENSG00000179399

66	53	16	21	-1,817638904	2,099821292	2,67E-12	1,92E-10	down	GPC4	ENSG00000076716
8	15	2	5	-1,825694292	-0,011486211	0,005607856	0,036810398	down	CYP24A1	ENSG00000019186
8	10	34	37	1,82576889	1,337819581	6,19E-07	1,56E-05	up	COCH	ENSG00000100473
53	42	184	188	1,827443228	3,623773042	1,11E-28	4,78E-26	up	OLFML3	ENSG00000116774
150	85	35	37	-1,828878674	3,041369439	3,35E-15	3,64E-13	down	DMBT1	ENSG00000187908
66	64	22	18	-1,835394408	2,218870927	4,01E-13	3,22E-11	down	GBP1	ENSG00000117228
6	7	26	26	1,840810363	0,927963515	1,00E-05	0,000186605	up	HAND1	ENSG00000113196
25	12	80	67	1,844898987	2,319948589	2,91E-13	2,43E-11	up	SERPINB9	ENSG00000170542
44	23	10	10	-1,863916955	1,309818055	6,84E-07	1,70E-05	down	HNF4G	ENSG00000164749
21	13	4	6	-1,877090403	0,440286455	0,000138098	0,001782647	down	LPL	ENSG00000175445
13	11	4	3	-1,8789188	0,025262356	0,000974496	0,009067073	down	ABI3BP	ENSG00000154175
5	1	11	14	1,879394562	0,023825186	0,001726336	0,014506637	up	EN2	ENSG00000164778
80	78	25	22	-1,885414612	2,47874393	3,46E-16	4,23E-14	down	CARD6	ENSG00000132357
20	21	8	4	-1,891151146	0,671311578	2,11E-05	0,000361133	down	FAM86FP	ENSG00000164845
20	11	5	4	-1,89235835	0,324554569	0,000241253	0,002853329	down	AQP11	ENSG00000178301
18	13	5	4	-1,893642684	0,32484601	0,000212539	0,002558538	down	AC005865.2	ENSG00000250770
49	36	15	10	-1,894039799	1,623640381	1,26E-09	5,49E-08	down	LRRC15	ENSG00000172061
7	4	18	28	1,90446462	0,76059577	5,45E-05	0,000819565	up	GPR68	ENSG00000119714
16	12	5	3	-1,91297094	0,199261364	0,000354543	0,003941087	down	ST14	ENSG00000149418
3313	2767	944	832	-1,916916925	7,713854708	1,51E-149	2,35E-145	down	TGFBI	ENSG00000120708
22	20	6	6	-1,924598836	0,694706538	1,12E-05	0,000205357	down	LDB2	ENSG00000169744
6	6	26	25	1,926389384	0,887774606	6,55E-06	0,000128186	up	TMEM74	ENSG00000164841
714	663	200	199	-1,930900962	5,563579545	4,27E-77	8,29E-74	down	IL2RB	ENSG00000100385
139	108	40	31	-1,934259236	3,093291045	1,23E-24	3,62E-22	down	RGS4	ENSG00000117152
1	3	8	10	1,966902158	-0,354132998	0,007935715	0,048793747	up	AC275455.1	ENSG00000283707
69	57	22	13	-1,980362007	2,143204846	2,09E-14	2,09E-12	down	CD22	ENSG00000012124
36	16	9	5	-2,003292052	0,948925273	7,11E-06	0,00013755	down	TRPV2	ENSG00000187688
19	11	6	2	-2,010699921	0,26313299	0,000157563	0,001989265	down	KRT19	ENSG00000171345
25	16	8	3	-2,011657586	0,646746479	1,46E-05	0,000259437	down	ANKFN1	ENSG00000153930
15	8	4	2	-2,03042216	-0,051091342	0,000675225	0,006751811	down	CLEC11A	ENSG00000105472
24	22	6	6	-2,055210708	0,785077991	1,76E-06	3,95E-05	down	LCTL	ENSG00000188501
4	3	16	17	2,060364803	0,323331887	0,00021083	0,002539941	up	PBX1	ENSG00000185630

88	48	18	17	-2,078896865	2,224831219	6,68E-13	5,29E-11	down	GGT1	ENSG00000100031
103	128	31	29	-2,096093203	2,972129852	3,19E-17	4,51E-15	down	F2RL2	ENSG00000164220
36	35	12	6	-2,105450071	1,341374952	1,63E-09	6,94E-08	down	LAT	ENSG00000213658
19	17	6	3	-2,108461183	0,468186935	1,92E-05	0,000331709	down	HAS2	ENSG00000170961
282	337	72	85	-2,134268208	4,373373678	1,02E-28	4,52E-26	down	MMP1	ENSG00000196611
18	15	5	3	-2,148372737	0,354833023	3,01E-05	0,000490689	down	AFF2	ENSG00000155966
9	12	1	4	-2,154644016	-0,172264521	0,001902044	0,015656993	down	SLC26A7	ENSG00000147606
18	20	5	4	-2,186866146	0,522059405	4,91E-06	0,000100203	down	KIRREL2	ENSG00000126259
476	413	113	102	-2,188067306	4,875993446	2,93E-80	6,50E-77	down	LCP1	ENSG00000136167
3	0	8	8	2,189281307	-0,506538308	0,008141014	0,049752775	up	SSR4P1	ENSG00000235374
10	8	1	3	-2,237095727	-0,353331356	0,00283323	0,0214307	down	PTGS1	ENSG00000095303
1	3	10	12	2,252731758	-0,173693608	0,00282733	0,021396493	up	ENHO	ENSG00000168913
769	621	188	128	-2,277387574	5,503408038	1,05E-92	4,10E-89	down	SPARC	ENSG00000113140
25	37	5	9	-2,277690334	1,13473364	1,26E-07	3,83E-06	down	IL1B	ENSG00000125538
8	15	4	1	-2,293300216	-0,089587778	0,00108915	0,009925711	down	ESM1	ENSG00000164283
13	6	3	1	-2,312226771	-0,306460161	0,00313807	0,023172177	down	PRSS3	ENSG00000010438
150	142	851	799	2,355691163	5,646322644	4,35E-121	2,25E-117	up	TULP3	ENSG00000078246
7	8	1	2	-2,364375591	-0,559689418	0,004802853	0,032583445	down	APOBEC3D	ENSG00000243811
10	15	68	76	2,374060427	2,203921788	1,62E-16	2,06E-14	up	ARHGAP44	ENSG00000006740
9	7	2	1	-2,45547724	-0,505473922	0,002752742	0,020954576	down	MTMR7	ENSG00000003987
39	29	8	5	-2,502912977	1,216673779	1,02E-10	5,48E-09	down	CIITA	ENSG00000179583
9	19	2	3	-2,580494956	0,098589821	0,000120168	0,001592225	down	CCDC190	ENSG00000185860
10	8	1	2	-2,622941555	-0,402235182	0,000939743	0,008882082	down	CD177	ENSG00000204936
16	14	3	2	-2,662815184	0,166457401	4,09E-06	8,54E-05	down	ODF3L2	ENSG00000181781
51	43	7	9	-2,674824849	1,62485346	8,08E-16	9,37E-14	down	CDCP1	ENSG00000163814
7	6	1	1	-2,688985564	-0,73679828	0,007639939	0,047380438	down	RGMA	ENSG00000182175
16	15	3	2	-2,709936172	0,19974135	2,38E-06	5,19E-05	down	MYH16	ENSG00000002079
24	14	4	2	-2,748776121	0,440340344	2,48E-07	7,04E-06	down	EYA1	ENSG00000104313
325	289	50	47	-2,800697864	4,245004226	2,47E-81	7,48E-78	down	CCND2	ENSG00000118971
11	10	0	3	-2,842758643	-0,259953336	0,000183439	0,00225544	down	TNFSF15	ENSG00000181634
15	13	2	2	-2,86810133	0,062093997	1,27E-05	0,000228178	down	MUC4	ENSG00000277585
15	11	0	3	-3,147193216	-0,050498438	1,07E-05	0,000196752	down	LRRN4	ENSG00000125872

0	1	6	6	3,167976754	-0,868771013	0,003611813	0,025954577	up	SPTBN4	ENSG00000160460
1	1	6	16	3,179783375	-0,26117006	0,000202458	0,002456226	up	KIF5C	ENSG00000168280
40	31	1	7	-3,247219612	1,184493761	2,71E-14	2,66E-12	down	DMKN	ENSG00000161249
7	4	0	1	-3,293639802	-0,938607711	0,006742669	0,04298313	down	LAMB4	ENSG00000091128
3	8	0	1	-3,304116222	-0,938016591	0,00493095	0,033254381	down	SERBP1P1	ENSG00000213740
42	24	5	2	-3,325164432	1,080331497	3,83E-13	3,12E-11	down	GALNT5	ENSG00000136542
1	1	23	4	3,446118882	-0,052688342	0,000380726	0,004196093	up	NPIPB12	ENSG00000169203
9	4	0	1	-3,528652043	-0,801161983	0,002079671	0,016837964	down	MAGEA3	ENSG00000221867
1	0	9	7	3,575652167	-0,617568877	0,000563624	0,00581176	up	B3GNT7	ENSG00000156966
25	28	350	413	3,702685249	4,410659297	2,89E-81	7,48E-78	up	SUSD2	ENSG00000099994
6	11	0	1	-3,917494897	-0,559237763	0,000124259	0,001636661	down	COL21A1	ENSG00000124749
19	23	1	1	-4,365603003	0,441723898	9,67E-11	5,21E-09	down	PODXL2	ENSG00000114631
0	0	4	4	4,978220398	-1,261073808	0,008015491	0,049198639	up	ITM2A	ENSG00000078596
0	0	5	4	5,142961548	-1,173672592	0,004049062	0,028528984	up	KCTD19	ENSG00000168676
7	2	0	0	-5,273857932	-1,173305344	0,004673119	0,03196866	down	EDN2	ENSG00000127129
6	3	0	0	-5,278290651	-1,173157386	0,004151504	0,029057817	down	MAGEC2	ENSG00000046774
6	10	0	0	-6,097247029	-0,674827141	2,26E-05	0,000384356	down	CD74	ENSG00000019582

Supplementary Table 2 Figure 7A

external_gene_name	disper	PValue	FDR	TEMDA231_SCR	TEMDA231_shFTO	log2FC_TE(MDA231_shFTO vs MDA231_SCR)	cntRnaMean	cntRiboMean
ENSG00000225630.1	0,002	0	0	0,05	0,142	1,504	978,427	83,678
IFI6	0,003	0	0	1,267	0,463	-1,452	75,837	58,445
C1orf122	0,002	0	0	0,605	0,29	-1,059	192,244	79,615
EBNA1BP2	0,001	0	0	0,358	0,461	0,363	2866,443	1170,19
NASP	0,001	0	0	0,307	0,408	0,41	2112,368	751,318
JUN	0,001	0	0	0,174	0,273	0,656	2053,409	444,716
CYR61	0,001	0	0	0,536	0,659	0,296	11184,336	6552,689
RPL5	0,001	0	0	0,276	0,357	0,37	2585,923	812,469
NOTCH2	0,001	0	0	0,079	0,121	0,62	3728,782	369,114
ECM1	0,001	0	0	0,393	0,628	0,674	1017,697	483,929
NUF2	0,001	0	0	0,246	0,372	0,6	793,417	245,211
LAMC1	0,001	0	0	0,131	0,223	0,761	1938,346	332,644
TPR	0,001	0	0	0,047	0,086	0,886	3102,831	200,475
CENPF	0,001	0	0	0,084	0,112	0,409	5249,81	511,535
MIA3	0,001	0	0	0,142	0,227	0,679	909,95	164,766
HNRNPU	0,001	0	0	0,081	0,107	0,408	9425,801	883,047
CRIM1	0,001	0	0	0,153	0,211	0,463	4826,809	873,063
RTN4	0,001	0	0	0,311	0,394	0,344	3840,999	1330,486
HSPE1	0,002	0	0	2,297	0,716	-1,683	53,189	67,941
FN1	0,001	0	0	0,064	0,124	0,962	2668,153	222,649
NCL	0,001	0	0	0,049	0,061	0,319	16611,196	911,104
RPSA	0,001	0	0	0,528	0,33	-0,678	651,543	268,061
MAP4	0,001	0	0	0,232	0,31	0,42	6565,502	1742,635
DAG1	0,001	0	0	0,198	0,274	0,468	2232,153	526,923
FLNB	0,001	0	0	0,048	0,068	0,495	15920,311	913,86
ALCAM	0,001	0	0	0,175	0,255	0,547	3005,537	656,306
GOLGB1	0,002	0	0	0,063	0,112	0,824	1039,332	91,501
TFRC	0,001	0	0	0,303	0,411	0,437	4470,104	1605,237
ENSG00000226950.2	0,001	0	0	0,73	0,467	-0,644	417,642	246,312
HNRNPD	0,001	0	0	0,244	0,336	0,46	3449,204	997,009

HNRNPDL	0,001	0	0	0,275	0,367	0,419	1633,484	526,66
CENPE	0,001	0	0	0,054	0,086	0,677	2463,657	172,431
FAT1	0,001	0	0	0,121	0,181	0,587	4864,598	739,701
CCT5	0,001	0	0	0,347	0,426	0,293	4740,411	1820,249
RPL37	0,001	0	0	0,49	0,738	0,593	2304,004	1369,883
TGFBI	0,001	0	0	0,332	0,463	0,482	1908,715	682,094
TMED9	0,001	0	0	0,666	0,511	-0,382	1391,479	797,492
DST	0,001	0	0	0,042	0,062	0,556	8430,247	440,862
PTPRK	0,002	0	0	0,124	0,245	0,981	466,847	84,328
PDCD2	0,001	0	0	0,312	0,201	-0,636	720,927	181,073
HNRNPA2B1	0,001	0	0	0,186	0,226	0,282	11055,421	2280,797
TSC22D4	0,002	0	0	0,128	0,258	1,011	341,377	61,091
LAMB1	0,001	0	0	0,161	0,258	0,676	1389,076	294,046
FLNC	0,001	0	0	0,062	0,095	0,618	6899,257	517,752
C7orf73	0,005	0	0	0,509	0,151	-1,756	110,032	32,54
DENND2A	0,004	0	0	0,292	0,114	-1,354	213,627	45,159
LOXL2	0,001	0	0	0,219	0,282	0,368	5369,763	1349,908
RPL30	0,001	0	0	2,027	3,299	0,703	242,336	628,847
EEF1D	0,001	0	0	0,611	0,471	-0,375	1935,855	1040,645
ENSG00000269900.2	0,001	0	0	6,148	3,25	-0,92	602,734	2790,255
CKS2	0,001	0	0	2,683	1,749	-0,618	311,871	673,601
HSPA5	0,001	0	0	0,305	0,454	0,576	5405,522	2027,492
ITGB1	0,001	0	0	0,333	0,423	0,342	4080,267	1547,021
NRP1	0,001	0	0	0,148	0,208	0,494	2066,077	359,017
ANAPC16	0,002	0	0	0,493	0,267	-0,887	278,037	100,529
VCL	0,001	0	0	0,05	0,077	0,626	5894,622	368,566
KIF20B	0,001	0	0	0,063	0,104	0,721	2562,471	213,265
ECHS1	0,001	0	0	0,647	0,486	-0,413	1082,966	608,881
RNH1	0,001	0	0	0,385	0,29	-0,409	1728,919	587,133
EIF3M	0,001	0	0	0,457	0,609	0,413	1475,997	782,389
CD44	0,001	0	0	0,317	0,406	0,36	11145,426	4016,704
MTCH2	0,001	0	0	0,655	0,462	-0,504	852,927	473,088

FAM111B	0,002	0	0	0,057	0,113	0,99	761,678	64,612
AHNAK	0,001	0	0	0,056	0,092	0,728	21029,951	1544,935
FOSL1	0,001	0	0	0,235	0,294	0,325	4065	1056,715
NUMA1	0,001	0	0	0,094	0,14	0,576	2404,445	276,699
RSF1	0,001	0	0	0,128	0,213	0,728	953,231	160,921
H2AFX	0,001	0	0	0,382	0,664	0,798	1696,11	841,688
STT3A	0,001	0	0	0,574	0,741	0,369	2971,02	1956,765
TPI1	0,001	0	0	1,191	0,68	-0,809	194,213	182,239
CD163L1	0,002	0	0	0,132	0,368	1,475	406,88	78,21
ATF7IP	0,001	0	0	0,286	0,407	0,506	1344,218	463,202
HSP90B1	0,001	0	0	0,294	0,441	0,584	4328,015	1596,659
ATP2A2	0,001	0	0	0,177	0,231	0,389	4599,397	935,59
SLC7A1	0,001	0	0	0,15	0,202	0,431	8267,271	1438,239
ITM2B	0,001	0	0	0,414	0,567	0,456	1382,826	672,858
STK24	0,001	0	0	0,193	0,133	-0,539	1644,763	264,169
COL4A1	0,001	0	0	0,196	0,296	0,59	2568,2	619,112
COL4A2	0,001	0	0	0,144	0,19	0,398	4835,739	799,258
HNRNPC	0,001	0	0	0,171	0,24	0,488	1749,751	358,941
MMP14	0,001	0	0	0,142	0,201	0,506	1666,813	281,772
ENSG00000265150.1	0,003	0	0	2,857	0,905	-1,658	34,49	61,173
CNIH1	0,001	0	0	0,969	0,745	-0,379	1009,955	855,159
KTN1	0,001	0	0	0,054	0,076	0,483	5704,447	372,12
DICER1	0,001	0	0	2,347	0,17	-3,79	1271,273	1490,491
THBS1	0,001	0	0	0,257	0,336	0,387	15719,388	4697,004
PDIA3	0,001	0	0	0,289	0,402	0,474	1394,796	478,103
LEO1	0,001	0	0	0,247	0,415	0,75	467,877	151,492
ANXA2	0,001	0	0	0,152	0,216	0,502	8404,23	1505,51
DENND4A	0,003	0	0	0,063	0,145	1,216	453,189	46,953
SMAD6	0,002	0	0	6,7	2,145	-1,643	30,477	107,313
SRRM2	0,001	0	0	0,053	0,084	0,661	8342,687	566,948
MT1E	0,001	0	0	1,009	0,69	-0,548	630,228	524,652
GLG1	0,001	0	0	0,219	0,313	0,515	1742,634	461,868

ENSG00000186594.8	0,002	0	0	0,12	0,327		1,443	349,818	75,539
CDK12	0,002	0	0	0,037	0,071		0,949	1584,856	83,108
EIF1	0,001	0	0	0,334	0,436		0,387	2805,526	1080,624
ITGA3	0,001	0	0	0,159	0,206		0,374	6984,24	1261,597
CCDC47	0,001	0	0	0,186	0,262		0,492	1355,08	306,588
LGALS3BP	0,001	0	0	0,487	0,601		0,304	3353,945	1789,05
ACTG1	0,001	0	0	0,219	0,293		0,418	5361,011	1365,684
ARHGDIA	0,001	0	0	0,412	0,519		0,331	3697,297	1692,208
DSG2	0,001	0	0	0,191	0,26		0,446	2317,557	519,482
SF3A2	0,001	0	0	0,171	0,238		0,475	1721,583	339,777
ZBTB7A	0,002	0	0	0,066	0,124		0,913	907,304	79,391
PRKCSH	0,001	0	0	0,406	0,531		0,39	5015,391	2327,302
CALR	0,001	0	0	0,492	0,632		0,361	7822,056	4376,944
FXYD5	0,001	0	0	0,552	0,711		0,364	2124,75	1323,491
TIMM50	0,001	0	0	0,426	0,293		-0,542	761,645	269,717
SLC1A5	0,001	0	0	0,407	0,508		0,321	3753,199	1720,61
SNRNP70	0,001	0	0	0,151	0,206		0,45	2439,01	428,973
RPL13A	0,001	0	0	0,298	0,193		-0,63	1719,015	411,961
RPL28	0,001	0	0	0,396	0,533		0,428	4742,576	2168,748
FKBP1A	0,001	0	0	0,23	0,129		-0,834	975,238	170,412
RPN2	0,001	0	0	0,463	0,573		0,308	4425,272	2277,582
UBE2C	0,001	0	0	0,604	0,773		0,356	1713,302	1166,256
ATP5E	0,001	0	0	0,66	1,245		0,915	373,066	338,784
APP	0,001	0	0	0,209	0,278		0,411	4624,211	1108,448
SCAF4	0,002	0	0	0,101	0,17		0,756	836,391	111,789
MYH9	0,001	0	0	0,075	0,091		0,271	47321,778	3902,605
RBX1	0,002	0	0	1,395	0,673		-1,052	119,897	115,78
C1GALT1C1	0,001	0	0	0,683	1,04		0,607	358,519	312,175
ENSG00000209082.1	0,002	0	0	0,985	2,781		1,498	47,305	85,746
UTP11L	0,001	0	0,001	0,259	0,376		0,539	764,279	241,257
MACF1	0,001	0	0,001	0,044	0,058		0,388	8179,088	416,408
UHMK1	0,001	0	0,001	0,059	0,037		-0,687	2587,486	126,395

EPRS	0,001	0	0,001	0,123	0,163	0,407	2969,547	426,426
GPX1	0,001	0	0,001	0,438	0,306	-0,52	709,175	265,098
EOGT	0,003	0	0,001	0,125	0,248	0,99	319,679	60,096
KIAA1524	0,001	0	0,001	0,167	0,247	0,565	957,825	198,745
SMC4	0,001	0	0,001	0,068	0,096	0,508	3320,806	272,15
LYAR	0,001	0	0,001	0,134	0,208	0,637	829,198	142,54
POLR2B	0,001	0	0,001	0,273	0,359	0,393	1584,63	504,929
MRPS18C	0,002	0	0,001	8,585	3,133	-1,454	28,05	127,762
AIMP1	0,001	0	0,001	0,723	1,003	0,472	536,559	461,571
SLC7A11	0,001	0	0,001	0,226	0,296	0,386	1993,03	515,023
CLGN	0,001	0	0,001	0,212	0,345	0,698	461,226	131
HMGB2	0,001	0	0,001	0,266	0,358	0,426	1487,697	462,554
ZFR	0,001	0	0,001	0,109	0,146	0,416	3223,561	410,216
COL12A1	0,001	0	0,001	0,064	0,096	0,582	2465,941	185,951
COX7A2	0,001	0	0,001	2,741	2	-0,455	344,811	803,83
SYNCRIP	0,001	0	0,001	0,09	0,128	0,503	2301,89	253,241
RAB32	0,001	0	0,001	0,662	0,389	-0,767	248,313	128,286
SERPINE1	0,001	0	0,001	0,362	0,44	0,281	4955,639	1980,608
SMC5	0,003	0	0,001	0,032	0,072	1,146	802,612	41,94
EIF3A	0,001	0	0,001	0,072	0,091	0,346	7420,251	602,355
NDUFS3	0,001	0	0,001	1,084	0,736	-0,559	356,245	325,25
HYOU1	0,001	0	0,001	0,213	0,323	0,596	672,048	178,56
APLP2	0,001	0	0,001	0,226	0,276	0,293	6851,086	1721,296
MRPL51	0,001	0	0,001	0,467	0,639	0,452	726,335	400,333
MED21	0,002	0	0,001	0,532	0,299	-0,829	236,978	95,303
RPS29	0,001	0	0,001	0,811	1,029	0,343	1210,201	1095,543
ISCA2	0,003	0	0,001	0,447	0,214	-1,065	166,641	51,485
CEP152	0,003	0	0,001	0,081	0,174	1,106	385,765	47,924
RPL27	0,001	0	0,001	0,577	0,703	0,284	3692,273	2337,866
EPS15L1	0,001	0	0,001	0,207	0,31	0,58	813,175	203,794
LSM4	0,001	0	0,001	0,475	0,341	-0,48	895,829	363,384
CST3	0,001	0	0,001	0,83	1,119	0,431	590,937	558,985

SNRPD3	0,001	0	0,001	0,608	0,802	0,398	845,42	590,739
PRR14L	0,001	0	0,001	0,147	0,215	0,543	1262,003	226,244
CDC42EP1	0,001	0	0,001	0,118	0,159	0,431	2796,076	378,041
MRPL20	0,001	0	0,002	0,414	0,636	0,618	346,962	174,346
WLS	0,001	0	0,002	0,55	0,709	0,365	1067,865	673,575
PYGO2	0,002	0	0,002	0,187	0,108	-0,788	559,599	79,219
RHOA	0,001	0	0,002	0,322	0,265	-0,28	4486,447	1307,715
IL7R	0,001	0	0,002	0,114	0,174	0,616	982,068	137,78
MAN2A1	0,001	0	0,002	0,157	0,226	0,526	1145,92	216,976
HMGA1	0,001	0	0,002	0,133	0,169	0,344	4554,771	674,945
GLO1	0,001	0	0,002	0,323	0,254	-0,346	2061,96	593,817
SEC16A	0,001	0	0,002	0,137	0,182	0,409	2385,41	376,705
RPL26	0,001	0	0,002	0,475	0,599	0,335	1405,442	759,282
MAPK7	0,002	0	0,002	0,121	0,212	0,812	489,189	76,691
SEC14L1	0,001	0	0,002	0,153	0,107	-0,509	1639,818	209,248
MRPL34	0,001	0	0,002	0,655	0,416	-0,656	293,842	155,86
AIFM1	0,002	0	0,002	0,225	0,123	-0,878	398,599	68,588
AURKAIP1	0,001	0	0,003	0,656	0,484	-0,437	644,128	364,581
PRDX1	0,001	0	0,003	0,999	0,842	-0,247	2625,822	2391,575
ACTR2	0,001	0	0,003	0,266	0,214	-0,308	3493,881	830,369
RPL31	0,001	0	0,003	1,817	1,431	-0,345	695,406	1125,015
RPL32	0,001	0	0,003	0,418	0,523	0,321	1728,89	809,763
EIF4G1	0,001	0	0,003	0,046	0,059	0,339	10676,301	555,199
TCERG1	0,001	0	0,003	0,128	0,177	0,467	1620,407	247,998
RRP36	0,001	0	0,003	0,284	0,421	0,568	517,145	179,756
MET	0,001	0	0,003	0,138	0,186	0,425	2000,947	333,487
PDIA4	0,001	0	0,003	0,428	0,539	0,331	1527,362	738,077
TCEB1	0,001	0	0,003	1,463	0,984	-0,572	232,583	277,695
ERP44	0,001	0	0,003	0,428	0,622	0,538	431,618	226,975
PTGES2	0,001	0	0,003	0,393	0,28	-0,492	750,403	252,805
CCAR1	0,001	0	0,003	0,096	0,134	0,482	1931,238	221,399
SLK	0,001	0	0,003	0,099	0,141	0,514	1588,042	191,213

RPLP2	0,001	0	0,003	1,148	1,344	0,227	3583,806	4423,814
SUCLA2	0,002	0	0,003	0,419	0,248	-0,757	288,833	91,269
ADAM10	0,001	0	0,003	0,207	0,282	0,441	1248,689	305,337
MFGE8	0,001	0	0,003	0,403	0,628	0,638	326,804	155,917
RSL1D1	0,001	0	0,003	0,142	0,19	0,419	1911,34	316,649
NLE1	0,001	0	0,003	0,397	0,261	-0,602	474,69	154,792
KPNB1	0,001	0	0,003	0,225	0,264	0,23	9165,183	2249,994
PSMG2	0,001	0	0,003	0,852	0,626	-0,444	516,767	377,58
CDC37	0,001	0	0,003	0,202	0,251	0,312	4201,38	931,957
CD97	0,001	0	0,003	0,582	0,716	0,299	1828,883	1181,498
PVR	0,001	0	0,003	0,204	0,278	0,448	1306,167	303,873
MYADM	0,001	0	0,003	0,603	0,747	0,308	1589,423	1069,492
IL17RA	0,002	0	0,003	0,12	0,208	0,788	486,233	76,934
NUDC	0,001	0	0,004	0,215	0,279	0,376	1810,988	437,231
PFDN2	0,001	0	0,004	0,706	0,92	0,381	730,934	577,197
SPARC	0,001	0	0,004	0,471	0,764	0,697	372,507	192,513
HNRNPAB	0,001	0	0,004	0,388	0,473	0,286	2572,653	1113,129
EPB41L2	0,001	0	0,004	0,094	0,128	0,444	2336,164	254,833
MGEA5	0,001	0	0,004	0,096	0,137	0,519	1515,4	177,77
LGMN	0,001	0	0,004	0,389	0,556	0,513	491,614	231,797
ACADVL	0,001	0	0,004	0,178	0,144	-0,305	4164,163	670,394
NFE2L1	0,001	0	0,004	0,121	0,158	0,382	2603,895	360,075
P4HB	0,001	0	0,004	0,504	0,592	0,233	5139,834	2812,62
SEH1L	0,001	0	0,004	0,439	0,343	-0,357	1440,904	554,294
POLR2I	0,002	0	0,004	1,12	0,602	-0,895	99,781	82,695
TPX2	0,001	0	0,004	0,047	0,061	0,382	7241,254	390,277
UBE2V1	0,002	0	0,004	7,775	2,749	-1,5	18,691	79,859
SF3A1	0,001	0	0,004	0,274	0,337	0,3	3113,925	943,381
SFPQ	0,001	0	0,005	0,099	0,125	0,331	5098,97	566,198
PTPRF	0,001	0	0,005	0,094	0,116	0,309	6547,81	676,139
ITGA6	0,001	0	0,005	0,153	0,196	0,363	2297,364	400,281
TFPI	0,001	0	0,005	0,254	0,347	0,448	947,125	276,097

TBC1D13	0,002	0	0,005	0,146	0,083	-0,824	560,493	64,442
PRDX3	0,001	0	0,005	0,603	0,484	-0,317	1567,943	845,679
TRIP11	0,002	0	0,005	0,068	0,109	0,674	1046,774	93,5
COA3	0,001	0	0,005	1,078	0,721	-0,58	233,228	203,456
NPC1	0,001	0	0,005	0,575	0,729	0,344	957,472	627,489
LAMP2	0,001	0	0,005	0,611	0,744	0,282	1707,099	1164,905
MAGOH	0,001	0	0,006	0,96	1,321	0,461	334,778	377,692
NUP210	0,001	0	0,006	0,139	0,194	0,482	1184,326	199,261
CANX	0,001	0	0,006	0,232	0,27	0,218	17004,077	4293,004
NDUFA4	0,001	0	0,006	2,713	1,888	-0,523	180,952	413,743
EGFR	0,001	0	0,006	0,123	0,154	0,323	4103,425	577,626
CFL1	0,001	0	0,006	0,313	0,37	0,24	7729,57	2625,773
SF3B2	0,001	0	0,006	0,272	0,334	0,299	2701,366	813,649
M6PR	0,001	0	0,006	0,299	0,381	0,352	1469,782	497,361
PIBF1	0,002	0	0,006	0,165	0,272	0,725	395,506	87,376
ENSG00000175061.13	0,001	0	0,006	0,774	0,66	-0,229	3326,266	2391,132
RAB12	0,002	0	0,006	0,322	0,185	-0,801	296,468	69,524
DDRGK1	0,001	0	0,006	0,274	0,403	0,554	491,11	162,612
HELZ2	0,004	0	0,006	0,083	0,04	-1,047	614,337	37,158
SON	0,001	0	0,006	0,135	0,17	0,325	3555,691	544,556
AKIRIN2	0,001	0	0,007	0,289	0,433	0,582	396,387	142,154
ATP6V1H	0,001	0	0,007	0,414	0,585	0,5	451,755	222,14
ENSG00000214110.3	0,002	0	0,007	1,866	0,96	-0,96	62,195	80,874
CYB561A3	0,002	0	0,007	0,368	0,21	-0,807	244,83	70,185
ACTN1	0,001	0	0,007	0,133	0,155	0,223	12624,075	1819,191
DCTPP1	0,001	0	0,007	0,592	0,393	-0,591	321,386	150,218
PLEKHG4	0,002	0	0,007	0,214	0,127	-0,756	446,616	76,932
RAB22A	0,002	0	0,007	0,172	0,104	-0,723	586,182	78,946
PKN2	0,002	0	0,008	0,064	0,097	0,6	1366,22	110,602
NTPCR	0,002	0	0,008	0,714	0,394	-0,86	130,614	68,805
PRPF40A	0,001	0	0,008	0,081	0,108	0,41	2855,833	272,185
NOP58	0,001	0	0,008	0,123	0,169	0,464	1387,455	199,651

ROBO1	0,002	0	0,008	0,094	0,147	0,644	815,212	95,295
LINC00998	0,002	0	0,008	1,371	0,778	-0,817	94,496	96,778
CALU	0,001	0	0,008	0,193	0,237	0,293	3051,713	658,692
TXN	0,001	0	0,008	2,236	1,844	-0,278	881,838	1791,035
NSMF	0,003	0	0,008	0,027	0,046	0,781	1710,579	60,137
PHLDA1	0,001	0	0,008	0,241	0,187	-0,365	1760,676	376,172
ATP5O	0,001	0	0,008	1,774	1,124	-0,659	129,488	179,757
POGZ	0,001	0	0,009	0,124	0,176	0,507	1073,399	160,578
IWS1	0,001	0	0,009	0,286	0,402	0,493	576,589	199,11
MTCH1	0,001	0	0,009	0,24	0,191	-0,333	2426,879	526,703
IGF2R	0,001	0	0,009	0,201	0,252	0,329	2174,174	492,237
PLAT	0,003	0	0,009	0,289	0,589	1,026	115,438	44,706
KIF11	0,001	0	0,009	0,105	0,14	0,412	2188,846	272,199
KIF18A	0,001	0	0,009	0,127	0,183	0,526	960,387	148,538
TTC17	0,001	0	0,009	0,153	0,223	0,544	771,388	141,728
LIMA1	0,001	0	0,009	0,1	0,128	0,354	3369,086	376,226
HEATR6	0,002	0	0,009	0,233	0,144	-0,694	452,906	83,987
ILVBL	0,001	0	0,009	0,466	0,337	-0,468	573,431	227,381
ARFGAP3	0,002	0	0,009	0,276	0,164	-0,75	343,649	75,147
DSP	0,002	0	0,01	0,045	0,07	0,621	1774,684	96,776
COX6C	0,001	0	0,01	2,099	2,955	0,493	180,211	450,867
RPS6	0,001	0	0,01	0,323	0,392	0,281	2335,907	832,232
TNC	0,001	0	0,01	0,121	0,158	0,383	2329,382	313,918
PRTFDC1	0,003	0	0,01	0,405	0,227	-0,839	195,963	59,858
KIF5B	0,001	0	0,01	0,102	0,122	0,268	6777,46	761,476
PTMS	0,001	0	0,01	0,166	0,221	0,411	1407,407	264,847
ZNF787	0,002	0	0,01	0,15	0,261	0,796	319,24	62,625
HUWE1	0,001	0	0,01	0,088	0,122	0,474	1672,002	175,32
CD58	0,001	0,001	0,011	0,624	0,971	0,637	175,391	136,383
FIP1L1	0,002	0	0,011	0,148	0,229	0,627	536,223	99,394
MKI67	0,001	0,001	0,011	0,013	0,017	0,397	16940,42	253,803
RSL24D1	0,002	0,001	0,011	0,474	0,277	-0,772	223,725	74,273

RUNX1	0,001	0,001	0,011	0,12	0,173	0,523	991,245	143,645
ENSG00000260464.1	0,003	0,001	0,012	10,475	44,895	2,1	3,303	56,541
ATP1A1	0,001	0,001	0,012	0,213	0,256	0,263	6119,078	1431,604
CYCS	0,001	0,001	0,012	0,912	0,678	-0,428	413,677	326,363
YME1L1	0,001	0,001	0,012	0,171	0,219	0,364	1773,968	347,663
UBAP2L	0,001	0,001	0,013	0,051	0,067	0,402	4187,845	242,642
SPTBN1	0,001	0,001	0,013	0,033	0,042	0,366	8020,867	297,488
HRSP12	0,003	0,001	0,013	0,863	0,449	-0,941	88,444	53,998
COMMD5	0,003	0,001	0,013	0,505	0,283	-0,837	158,789	59,384
SH3GLB2	0,001	0,001	0,013	0,173	0,237	0,458	950,561	192,302
TMEM258	0,001	0,001	0,013	3,041	2,09	-0,541	138,792	348,231
PGAM5	0,002	0,001	0,013	0,146	0,086	-0,768	529,447	59,869
EXOSC8	0,001	0,001	0,013	0,385	0,505	0,39	739,626	324,859
FAM96A	0,002	0,001	0,013	0,571	0,324	-0,818	149,329	65,279
RPL19	0,001	0,001	0,013	0,489	0,589	0,268	1851,869	999,935
PFKL	0,001	0,001	0,013	0,229	0,163	-0,489	884,97	176,737
CDC42	0,001	0,001	0,014	0,16	0,109	-0,543	929,549	123,598
ATPIF1	0,001	0,001	0,014	0,528	0,723	0,455	415,326	253,011
CCNI	0,001	0,001	0,014	0,137	0,104	-0,392	2138,435	256,677
GANAB	0,001	0,001	0,014	0,354	0,414	0,224	6638,572	2552,484
TPT1	0,001	0,001	0,014	0,176	0,213	0,275	3644,433	710,324
RNF114	0,001	0,001	0,014	0,376	0,28	-0,427	765,446	249,477
HNRNPR	0,001	0,001	0,015	0,151	0,188	0,311	2788,151	474,413
COL5A1	0,001	0,001	0,015	0,107	0,141	0,405	1894,052	231,761
BAZ2A	0,001	0,001	0,015	0,108	0,143	0,405	1821,2	228,905
CPSF6	0,001	0,001	0,015	0,193	0,258	0,417	1036,427	232,802
IQGAP1	0,001	0,001	0,015	0,061	0,077	0,35	4872,577	333,21
NUP93	0,001	0,001	0,015	0,345	0,273	-0,337	1335,19	407,496
ZFHX3	0,001	0,001	0,015	0,339	0,25	-0,44	751,521	217,47
KHSRP	0,001	0,001	0,015	0,196	0,233	0,249	4363,453	936,504
IRGQ	0,002	0,001	0,015	0,195	0,115	-0,755	409,001	61,38
PYGB	0,001	0,001	0,015	0,179	0,139	-0,361	2002,025	316,133

LEPRE1	0,001	0,001	0,016	0,34	0,416	0,292	1671,962	631,924
FUBP1	0,001	0,001	0,016	0,173	0,217	0,331	2097,997	405,759
ENSG00000234741.3	0,001	0,001	0,016	0,682	0,533	-0,356	699,826	423,355
TMX2	0,001	0,001	0,016	0,266	0,188	-0,502	676,895	150,264
PXDN	0,001	0,001	0,017	0,117	0,155	0,398	1906,119	252,313
TBCA	0,002	0,001	0,017	0,73	1,156	0,663	138,212	123,711
ENPP4	0,003	0,001	0,017	0,31	0,576	0,894	116,191	51,198
TMEM65	0,003	0,001	0,017	0,397	0,214	-0,892	161,125	46,502
HNRNPF	0,001	0,001	0,017	0,192	0,23	0,259	4252,936	908,266
TMEM223	0,002	0,001	0,017	0,7	0,437	-0,68	173,788	96,188
CLN5	0,002	0,001	0,017	0,329	0,528	0,682	209,882	87,849
PPP1R15A	0,001	0,001	0,017	0,093	0,13	0,48	1394,229	153,673
PLXNB2	0,001	0,001	0,017	0,173	0,201	0,22	9663,729	1789,732
ENSG00000210082.2	0,001	0,001	0,017	0,015	0,019	0,268	56580,483	959,032
NCSTN	0,001	0,001	0,018	0,345	0,433	0,327	1166,802	452,06
BHLHE40	0,002	0,001	0,018	0,121	0,198	0,708	441,209	67,383
TMF1	0,001	0,001	0,018	0,166	0,24	0,531	631,69	129,151
COX17	0,004	0,001	0,018	2,916	1,358	-1,103	34,956	65,473
HSPB1	0,001	0,001	0,018	0,798	0,951	0,254	1395,827	1209,688
FAM120A	0,001	0,001	0,018	0,146	0,117	-0,319	2946,053	385,871
COL13A1	0,001	0,001	0,018	0,138	0,183	0,407	1410,67	226,474
JAG1	0,003	0,001	0,018	0,183	0,311	0,764	250,583	61,336
UBE2J2	0,002	0,001	0,019	0,29	0,197	-0,558	487,019	117,979
DVL1	0,002	0,001	0,019	0,074	0,046	-0,688	1146,81	70,917
PPM1G	0,001	0,001	0,019	0,173	0,211	0,29	2682,96	513,747
MTA3	0,003	0,001	0,019	0,175	0,307	0,808	226,398	53,845
SHFM1	0,001	0,001	0,019	1,826	2,936	0,685	80,489	181,795
ZDHHC13	0,002	0,001	0,019	0,258	0,41	0,666	257,242	84,491
OS9	0,001	0,001	0,019	0,312	0,407	0,383	824,017	294,065
TBL3	0,001	0,001	0,019	0,391	0,288	-0,44	627,771	213,712
ENSG00000206630.1	0,001	0,001	0,019	31,747	212,716	2,744	3,392	251,867
SDHC	0,001	0,001	0,02	2,473	1,554	-0,671	87,473	166,946

ENSG00000208317.1	0,002	0,001	0,02	191,106	15,489	-3,625	3,164	90,509
KDM5B	0,003	0,001	0,02	0,117	0,069	-0,756	601,084	54,751
POLR2E	0,001	0,001	0,02	0,218	0,268	0,299	2313,582	558,721
ZMYM4	0,003	0,001	0,021	0,062	0,103	0,735	681,562	56,298
SARS	0,001	0,001	0,021	0,264	0,321	0,279	2347,949	676,604
MEF2D	0,002	0,001	0,021	0,099	0,162	0,705	502,109	63,212
NCAPH	0,001	0,001	0,021	0,264	0,329	0,315	1480,508	439,979
SELT	0,002	0,001	0,021	0,522	0,324	-0,689	198,138	83,378
SNRPC	0,001	0,001	0,021	0,358	0,463	0,368	788,408	319,05
BUD31	0,002	0,001	0,021	0,312	0,493	0,661	221,569	86,773
RRAGA	0,002	0,001	0,021	0,262	0,418	0,672	244,634	81,589
PITRM1	0,001	0,001	0,021	0,168	0,219	0,388	1352,706	261,758
ESRRA	0,003	0,001	0,021	0,293	0,169	-0,795	238,594	53,883
CKAP2	0,001	0,001	0,021	0,066	0,085	0,353	3639,785	275,138
CARS2	0,001	0,001	0,021	0,252	0,184	-0,455	851,43	190,48
EDC4	0,001	0,001	0,021	0,168	0,221	0,4	1222,259	239,549
RAB34	0,001	0,001	0,021	0,344	0,258	-0,413	758,257	226,454
NDUFA11	0,001	0,001	0,021	1,232	0,867	-0,508	206,201	216,1
RAB11B	0,001	0,001	0,021	0,261	0,342	0,39	948,168	278,382
TMEM147	0,001	0,001	0,021	0,787	0,591	-0,414	419,023	287,406
HMGXB4	0,002	0,001	0,021	0,077	0,117	0,603	884,688	85,697
CLSTN1	0,001	0,001	0,022	0,183	0,235	0,361	1459,572	297,867
ID3	0,001	0,001	0,022	0,461	0,656	0,509	297,671	167,935
SF3A3	0,001	0,001	0,022	0,141	0,177	0,327	2314,185	367,999
TOR3A	0,001	0,001	0,022	0,873	0,622	-0,49	271,717	203,916
RPL14	0,001	0,001	0,022	0,383	0,46	0,265	2333,336	973,99
PLOD2	0,001	0,001	0,022	0,202	0,269	0,409	956,297	222,171
CTBP1	0,001	0,001	0,022	0,208	0,155	-0,423	1106,928	199,331
MRPL22	0,001	0,001	0,022	1,558	1,127	-0,467	215,6	284,003
CDKN1A	0,003	0,001	0,022	0,302	0,167	-0,859	200,378	46,923
ACTB	0,001	0,001	0,022	0,639	0,564	-0,18	22346,643	13458,51
SEC61B	0,001	0,001	0,022	2,068	1,576	-0,392	281,624	508,942

FBXW5	0,002	0,001	0,022	0,128	0,088	-0,529	1101,894	119,62
ANKRD1	0,001	0,001	0,022	0,212	0,272	0,358	1262,679	307,66
DHCR7	0,001	0,001	0,022	0,429	0,54	0,332	901,65	427,914
FDX1	0,003	0,001	0,022	0,616	0,343	-0,847	117,056	54,399
GXYLT1	0,003	0,001	0,022	0,107	0,178	0,741	401,721	58,158
NDUFB10	0,001	0,001	0,022	0,483	0,646	0,421	454,34	252,465
RNASEH2A	0,001	0,001	0,022	0,852	0,684	-0,317	725,521	554,505
WDR54	0,001	0,001	0,023	0,774	0,597	-0,375	513,554	344,83
SP3	0,001	0,001	0,023	0,187	0,244	0,387	1149,592	247,766
LY6E	0,001	0,001	0,023	0,282	0,358	0,347	1091,472	348,586
NCOR2	0,001	0,001	0,023	0,121	0,149	0,299	4027,218	530,779
RAN	0,001	0,001	0,023	0,523	0,432	-0,276	1398,482	666,31
QSOX1	0,001	0,002	0,024	0,325	0,381	0,23	3045,818	1058,96
CNOT11	0,001	0,002	0,024	0,305	0,223	-0,453	668,651	175,261
ITGAV	0,001	0,002	0,024	0,2	0,264	0,399	989,406	228,377
NMD3	0,002	0,002	0,024	0,144	0,219	0,599	495,465	89,841
ANKRD17	0,001	0,002	0,024	0,105	0,135	0,358	2259,956	270,215
SPTAN1	0,001	0,002	0,024	0,091	0,111	0,281	5668,176	568,449
HNRNPH3	0,001	0,002	0,024	0,14	0,183	0,386	1452,503	234,852
ZNHIT2	0,004	0,002	0,024	1,24	0,601	-1,044	50,116	41,602
ARHGDIB	0,001	0,002	0,024	0,277	0,331	0,255	2664,413	800,223
RBM25	0,001	0,002	0,024	0,057	0,078	0,463	2083,756	140,823
COX5A	0,001	0,002	0,024	1,074	1,398	0,38	348,64	425,962
ITPRIPL2	0,002	0,002	0,024	0,117	0,073	-0,676	695,976	64,341
ATP5G1	0,001	0,002	0,024	1,397	1,089	-0,36	396,114	491,953
GRB2	0,001	0,002	0,024	0,307	0,234	-0,389	928,091	246,37
RCN3	0,001	0,002	0,024	0,403	0,55	0,449	427,883	201,202
EP300	0,001	0,002	0,024	0,11	0,147	0,416	1483,607	190,208
OSMR	0,001	0,002	0,025	0,24	0,315	0,391	855,845	237,876
MRPS18B	0,003	0,002	0,025	0,486	0,888	0,869	84,736	54,634
BCLAF1	0,002	0,002	0,025	0,05	0,071	0,519	1800,182	109,506
GRN	0,001	0,002	0,025	0,419	0,492	0,23	2715,998	1225,499

SH3GL1	0,001	0,002	0,025	0,144	0,187	0,384	1475,199	240,374
RALY	0,001	0,002	0,025	0,176	0,217	0,307	2121,232	414,555
DHX35	0,002	0,002	0,025	0,129	0,211	0,703	367,037	61,989
PAF1	0,001	0,002	0,026	0,135	0,185	0,459	957,038	151,532
ICMT	0,001	0,002	0,027	0,098	0,071	-0,448	1793,222	150,891
HYI	0,002	0,002	0,027	0,378	0,628	0,732	151,518	70,87
GPATCH4	0,001	0,002	0,027	0,09	0,124	0,459	1367,662	147,05
TMEM131	0,001	0,002	0,027	0,106	0,139	0,392	1794,953	221,006
COPS8	0,001	0,002	0,027	0,321	0,232	-0,471	571,109	154,846
GARS	0,001	0,002	0,027	0,187	0,222	0,249	3669,65	750,677
CHP1	0,001	0,002	0,027	0,217	0,153	-0,501	692,197	125,742
INO80E	0,001	0,002	0,027	0,282	0,403	0,514	402,945	134,726
DDAH1	0,001	0,002	0,028	0,14	0,112	-0,323	2735,336	346,58
WDR47	0,004	0,002	0,028	0,179	0,099	-0,863	289,703	39,162
LAMB2	0,001	0,002	0,028	0,134	0,167	0,317	2307,984	346,15
SEC61A1	0,001	0,002	0,028	0,242	0,285	0,234	3877,064	1015,021
MRPL32	0,001	0,002	0,028	0,461	0,619	0,425	427,419	225,836
MCAM	0,001	0,002	0,028	0,199	0,26	0,386	1209,703	263,624
PPIB	0,001	0,002	0,028	1,252	1,585	0,339	421,424	585,137
MIEF2	0,003	0,002	0,028	0,375	0,221	-0,76	195,212	58,378
DCAF7	0,001	0,002	0,028	0,116	0,092	-0,332	2915,59	303,1
SOD1	0,001	0,002	0,028	0,75	0,921	0,296	764,274	632,214
RPL22	0,001	0,002	0,029	0,911	0,682	-0,418	339,569	266,335
EMC1	0,001	0,002	0,029	0,277	0,349	0,333	1109,878	346,571
EHBP1L1	0,001	0,002	0,029	0,096	0,131	0,45	1375,937	152,498
LRP1	0,002	0,002	0,029	0,14	0,212	0,595	543,593	89,442
SRSF9	0,001	0,002	0,029	0,511	0,407	-0,329	863,808	395,26
ABCC1	0,001	0,002	0,029	0,247	0,313	0,345	1134,261	314,511
FTL	0,001	0,002	0,029	0,076	0,059	-0,356	3845,198	258,041
ATP6V0B	0,001	0,002	0,03	0,857	0,708	-0,275	988,943	772,56
EGLN1	0,003	0,002	0,03	0,153	0,091	-0,752	422,14	51,817
PTPN23	0,001	0,002	0,03	0,158	0,204	0,369	1448,898	255,394

RPL9	0,002	0,002	0,03	1,192	0,719	-0,729	93,967	84,08
UTP3	0,003	0,002	0,03	0,074	0,126	0,764	475,212	47,862
ZMAT2	0,001	0,002	0,03	0,239	0,328	0,453	583,405	163,497
CAPZA2	0,001	0,002	0,03	0,434	0,314	-0,468	441,367	161,844
TXNRD1	0,001	0,002	0,03	0,108	0,13	0,273	4165,446	497,407
NDUFB7	0,001	0,002	0,03	1,206	0,924	-0,383	341,343	364,819
RAE1	0,001	0,002	0,03	0,308	0,397	0,369	782,618	277,525
ECE1	0,001	0,002	0,031	0,226	0,276	0,286	1896,209	476,649
ACOX2	0,004	0,002	0,031	1,256	0,569	-1,142	42,014	41,253
SLC41A3	0,001	0,002	0,031	0,545	0,382	-0,513	301,056	137,866
AURKB	0,001	0,002	0,031	0,331	0,396	0,256	2086,832	753,04
SPCS1	0,001	0,002	0,032	0,709	0,566	-0,325	683,586	432,286
TENM3	0,003	0,002	0,032	0,096	0,157	0,706	462,437	55,537
YIPF3	0,001	0,002	0,032	0,409	0,535	0,389	552,585	256,135
PTBP3	0,001	0,002	0,032	0,169	0,131	-0,364	1697,579	250,33
NUP214	0,001	0,002	0,032	0,174	0,217	0,316	1786,708	346,214
PAWR	0,001	0,002	0,032	0,181	0,136	-0,415	1149,367	180,543
CPD	0,001	0,002	0,032	0,218	0,288	0,402	819,543	207,513
EIF3G	0,001	0,002	0,032	0,42	0,501	0,254	1704,029	777,251
MCAT	0,003	0,002	0,032	0,554	0,317	-0,807	124,269	50,69
FAM102B	0,003	0,003	0,033	0,11	0,065	-0,757	539,892	45,784
CAPG	0,001	0,003	0,033	0,537	0,454	-0,242	1778,618	886,197
DTYMK	0,001	0,002	0,033	0,457	0,355	-0,366	703,864	281,697
SEC61G	0,001	0,002	0,033	1,451	1,896	0,386	262,235	435,624
SEZ6L2	0,001	0,003	0,033	0,344	0,488	0,507	334,917	135,403
C19orf70	0,001	0,003	0,033	1,715	1,227	-0,483	169,756	251,046
NDUFB2	0,001	0,003	0,034	1,367	0,943	-0,536	149,979	171,735
ROBO4	0,001	0,003	0,034	0,202	0,276	0,453	660,666	154,739
SCNM1	0,002	0,003	0,035	0,338	0,486	0,522	302,982	123,144
DEGS1	0,001	0,003	0,035	0,665	0,829	0,32	618,232	457,566
NDUFS1	0,001	0,003	0,035	0,213	0,173	-0,3	1946,308	375,306
FYTTD1	0,002	0,003	0,035	0,108	0,165	0,614	541,687	75,367

CCNB1	0,001	0,003	0,035	0,427	0,363	-0,233	2878,777	1129,57
SEC63	0,001	0,003	0,035	0,144	0,203	0,491	690,306	121,074
KIAA1244	0,003	0,003	0,035	0,168	0,1	-0,757	372,029	50,592
RAC1	0,001	0,003	0,035	0,104	0,078	-0,411	1907,494	173,792
ERMP1	0,003	0,003	0,035	0,354	0,592	0,741	132,988	61,229
ENG	0,001	0,003	0,035	0,301	0,364	0,271	1752,993	577,652
PMP22	0,001	0,003	0,035	0,4	0,33	-0,278	1454,769	531,82
CALCOCO2	0,001	0,003	0,035	0,124	0,168	0,437	1055,864	153,954
PLAUR	0,001	0,003	0,035	0,523	0,662	0,34	618,402	365,883
DBI	0,001	0,003	0,036	0,431	0,568	0,397	483,527	236,866
TMEM14C	0,001	0,003	0,036	0,888	0,651	-0,449	273,705	211,002
CDK1	0,001	0,003	0,036	0,507	0,594	0,228	1889,626	1045,379
SLC6A6	0,001	0,003	0,037	0,17	0,215	0,335	1557,803	299,138
MGST2	0,002	0,003	0,037	0,933	0,587	-0,67	117,641	89,129
PAIP2	0,001	0,003	0,037	0,331	0,438	0,404	554,955	210,976
HSPH1	0,001	0,003	0,037	0,096	0,115	0,257	4840,702	510,449
MZT1	0,001	0,003	0,037	0,597	0,432	-0,465	330,654	164,934
IMP3	0,001	0,003	0,037	0,919	0,691	-0,411	320,071	254,059
FMNL1	0,001	0,003	0,037	0,101	0,134	0,412	1417,778	169,357
UQCR10	0,001	0,003	0,037	2,004	1,505	-0,413	212,4	371,435
KTI12	0,003	0,003	0,038	0,384	0,226	-0,76	175,687	53,774
PRPF38B	0,002	0,003	0,038	0,059	0,087	0,552	1165,006	84,617
KPNA4	0,002	0,003	0,038	0,069	0,101	0,546	1027,156	86,658
SNX2	0,001	0,003	0,038	0,406	0,516	0,347	669,724	309,35
IGFBP1	0,001	0,003	0,038	0,712	0,92	0,369	394,122	322,679
LIN7C	0,001	0,003	0,038	0,356	0,276	-0,367	788,796	247,083
MYL6B	0,003	0,003	0,038	0,339	0,195	-0,797	178,204	48,026
NDFIP2	0,001	0,003	0,038	0,639	0,499	-0,355	550,569	311,735
APEX1	0,001	0,003	0,038	0,246	0,301	0,291	1650,623	449,212
CTNS	0,001	0,003	0,038	0,213	0,298	0,484	497,969	128,141
UBB	0,001	0,003	0,038	1,695	2,072	0,29	477,278	888,845
PRKAR1A	0,001	0,003	0,038	0,125	0,099	-0,336	2337,35	260,179

ATP8B1	0,002	0,003	0,038	0,13	0,205	0,658	383,767	62,552
LSM10	0,003	0,003	0,039	0,304	0,186	-0,708	237,263	57,461
GALNT7	0,001	0,003	0,039	0,228	0,305	0,418	689,402	179,087
DEK	0,001	0,003	0,039	0,147	0,176	0,265	3088,001	502,113
HTATIP2	0,001	0,003	0,039	0,374	0,267	-0,489	415,676	134,213
TMEM179B	0,001	0,003	0,04	0,829	1,205	0,54	155,984	152,063
TIMM8B	0,002	0,003	0,04	0,969	0,619	-0,646	119,354	93,154
TMEM230	0,002	0,003	0,04	0,194	0,129	-0,593	483,855	75,077
SEP15	0,001	0,004	0,041	0,413	0,341	-0,278	1370,547	514,498
ZFP36L2	0,002	0,004	0,041	0,362	0,244	-0,568	307,611	91,057
MRS2	0,002	0,004	0,041	0,285	0,18	-0,662	277,732	62,515
CYC1	0,001	0,003	0,041	0,641	0,553	-0,212	2221,766	1330,26
SLC38A2	0,001	0,004	0,041	0,247	0,301	0,286	1676,403	467,131
DLGAP4	0,001	0,004	0,041	0,164	0,205	0,317	1796,596	332,418
DIDO1	0,001	0,004	0,041	0,057	0,079	0,477	1650,52	110,412
SLC4A7	0,001	0,004	0,042	0,122	0,166	0,45	931,493	136,978
SRPRB	0,001	0,004	0,042	0,536	0,418	-0,359	590,022	278,271
ADH5	0,001	0,004	0,042	0,541	0,391	-0,468	329,986	153,238
ACOX1	0,002	0,004	0,042	0,322	0,227	-0,507	418,786	112,475
SLC39A3	0,002	0,004	0,042	0,364	0,239	-0,608	270,338	82,088
ACN9	0,003	0,004	0,043	0,612	0,345	-0,825	100,197	45,914
SLC38A1	0,001	0,004	0,043	0,121	0,144	0,245	4386,289	582,238
ZC3H18	0,001	0,004	0,043	0,107	0,145	0,432	1159,013	144,099
ARRB2	0,002	0,004	0,043	0,153	0,221	0,53	517,236	96,261
SFN	0,001	0,004	0,044	0,418	0,516	0,306	906,347	414,181
ENSG00000200087.1	0,001	0,004	0,044	35,601	16,585	-1,102	15,688	381,406
PKM	0,001	0,004	0,044	0,195	0,222	0,188	17597,884	3655,731
MECR	0,004	0,004	0,045	0,339	0,186	-0,867	142,57	36,469
F3	0,001	0,004	0,045	0,324	0,375	0,214	3802,195	1277,649
PRRC2C	0,001	0,004	0,045	0,044	0,055	0,333	5452,006	265,31
ILKAP	0,002	0,004	0,045	0,765	0,524	-0,545	188,671	116,836
HEG1	0,001	0,004	0,045	0,091	0,124	0,434	1315,446	140,385

ZNF195	0,003	0,004	0,045	0,136	0,224	0,724	278,412	49,145
RCN1	0,002	0,004	0,045	0,111	0,155	0,486	832,586	109,764
TRAF7	0,002	0,004	0,045	0,123	0,087	-0,502	999,364	105,927
PRPF8	0,001	0,004	0,045	0,111	0,125	0,179	20780,701	2452,192
SNRPB	0,001	0,004	0,045	0,535	0,457	-0,228	2147,012	1055,194
DDX46	0,001	0,004	0,046	0,053	0,071	0,416	2281,122	143,198
PBK	0,001	0,004	0,046	0,462	0,364	-0,343	708,596	287,876
B3GNT1	0,002	0,004	0,046	0,833	0,566	-0,559	168,449	113,061
CDK4	0,001	0,004	0,046	0,523	0,408	-0,36	577,634	267,122
HAUS8	0,002	0,004	0,046	0,231	0,347	0,59	283,163	80,645
NDUFA3	0,003	0,004	0,046	3,173	9,225	1,54	10,922	50,755
LZTR1	0,003	0,004	0,046	0,113	0,069	-0,711	540,322	49,109
SNAPC1	0,001	0,004	0,047	0,166	0,221	0,41	866,77	165,339
BCL2L12	0,001	0,004	0,047	0,613	0,434	-0,499	254,392	132,064
CRBN	0,002	0,004	0,048	0,402	0,264	-0,606	239,402	76,695
ENSG00000270141.2	0,001	0,004	0,048	198,903	61,247	-1,699	6,408	652,752
GAK	0,002	0,004	0,048	0,162	0,113	-0,528	668,414	92,103
RPS18	0,001	0,004	0,048	2,827	3,671	0,377	190,722	613,442
TAGLN	0,003	0,004	0,048	0,636	1,222	0,941	52,669	43,476
ABCC4	0,002	0,004	0,048	0,141	0,194	0,462	737,686	124,372
GMIP	0,004	0,004	0,048	0,206	0,118	-0,808	257,807	42,915
MOCS3	0,004	0,004	0,048	0,85	0,451	-0,913	64,199	39,51
ALMS1	0,001	0,005	0,049	0,112	0,142	0,34	1931,797	246,793
SPATS2L	0,001	0,005	0,049	0,1	0,127	0,349	2093,908	234,167
ATP1B3	0,001	0,005	0,049	0,685	0,85	0,312	561,056	431,298
EIF2AK1	0,001	0,005	0,049	0,098	0,131	0,414	1292,249	148,327
MRPL52	0,001	0,005	0,049	2,094	1,575	-0,411	189,899	343,733
NUP85	0,001	0,005	0,049	0,399	0,321	-0,314	931,605	334,432
ELOVL5	0,001	0,005	0,05	0,407	0,469	0,203	3328,761	1442,278
AMD1	0,001	0,005	0,05	0,278	0,213	-0,383	800,868	191,505
PHPT1	0,001	0,005	0,05	1,235	0,911	-0,439	210,539	225,329
TCF7L2	0,002	0,005	0,05	0,166	0,244	0,558	408,874	83,248

ADRBK1	0,002	0,005	0,05	0,114	0,077	-0,551	839,877	79,762
PICALM	0,001	0,005	0,05	0,121	0,148	0,286	2619,295	352,339
MMP1	0,002	0,005	0,05	0,467	0,763	0,71	189,132	96,989
MRPL27	0,001	0,005	0,05	1,187	0,877	-0,437	217,511	221,399
C20orf24	0,001	0,005	0,05	0,781	1,111	0,509	167,586	153,679
NDUFA9	0,003	0,005	0,051	0,679	0,414	-0,712	115,55	60,918
DERL2	0,001	0,005	0,051	0,666	0,528	-0,336	542,103	318,949
PDHA1	0,001	0,005	0,051	0,281	0,356	0,344	827,572	260,428
SLC35C1	0,002	0,005	0,052	0,283	0,181	-0,641	277,944	64,856
NDUFV1	0,001	0,005	0,052	0,293	0,232	-0,337	987,311	258,559
TAF1D	0,001	0,005	0,052	0,132	0,177	0,424	932,292	143,887
ATM	0,002	0,005	0,052	0,093	0,064	-0,541	1032,537	79,205
NEO1	0,001	0,005	0,052	0,157	0,216	0,457	656,821	123,763
MRPS34	0,001	0,005	0,052	0,531	0,426	-0,317	730,679	347,797
SAFB	0,001	0,005	0,052	0,224	0,27	0,269	1771,956	435,65
TM9SF4	0,001	0,005	0,052	0,314	0,381	0,277	1325,643	458,587
ZNF644	0,002	0,005	0,053	0,083	0,115	0,478	1048,325	104,501
ATAD1	0,001	0,005	0,053	0,242	0,317	0,393	663,829	186,074
SLC25A28	0,002	0,005	0,053	0,173	0,268	0,628	298,419	64,021
CTSD	0,001	0,005	0,053	0,301	0,361	0,263	1492,668	490,205
IPO7	0,001	0,005	0,053	0,146	0,173	0,243	3490,355	556,398
ERO1L	0,001	0,005	0,053	0,169	0,212	0,327	1398,613	264,339
CUL4B	0,001	0,005	0,054	0,157	0,189	0,266	2588,752	450,538
DRAP1	0,001	0,005	0,055	0,296	0,351	0,247	1947,14	624,22
ATRN	0,001	0,005	0,055	0,182	0,246	0,435	648,756	139,69
AHCY	0,001	0,006	0,056	0,308	0,268	-0,204	3526,927	1012,69
SH3BGRL3	0,001	0,006	0,057	0,394	0,469	0,251	1527,71	647,601
BTF3	0,001	0,006	0,057	0,291	0,345	0,245	1996,616	619,092
MYO1C	0,001	0,006	0,057	0,166	0,146	-0,189	8119,541	1268,679
ZNF146	0,001	0,006	0,057	0,084	0,112	0,408	1450,955	143,257
AKIRIN1	0,001	0,006	0,058	0,197	0,146	-0,432	788,726	135,457
SNX17	0,001	0,006	0,058	0,366	0,281	-0,381	646,386	213,761

PTMA	0,001	0,006	0,058	0,363	0,304	-0,257	1589,632	526,86
PTTG1	0,001	0,006	0,058	0,46	0,551	0,261	1069,839	538,75
ASF1A	0,002	0,006	0,058	0,375	0,247	-0,605	234,61	71,268
TONSL	0,002	0,006	0,058	0,097	0,069	-0,509	1074,468	87,178
SGMS1	0,002	0,006	0,058	0,175	0,253	0,532	424,699	88,022
TSPAN14	0,001	0,006	0,058	0,163	0,126	-0,368	1365,794	202,366
USMG5	0,001	0,006	0,058	2,107	1,626	-0,374	217,426	401,43
SF1	0,001	0,006	0,058	0,171	0,197	0,207	4811,888	880,012
RPS3	0,001	0,006	0,058	0,617	0,699	0,18	4765,142	3122
ARL1	0,001	0,006	0,058	0,257	0,194	-0,408	707,464	156,304
SLC39A6	0,002	0,006	0,058	0,152	0,222	0,543	442,966	82,397
MRPL54	0,001	0,006	0,058	1,072	1,468	0,453	177,565	217,881
FAM32A	0,002	0,006	0,058	0,182	0,132	-0,466	716,457	111,886
SIRT2	0,003	0,006	0,058	0,239	0,15	-0,668	283,283	53,638
AKT1S1	0,001	0,006	0,058	0,129	0,172	0,414	999,726	147,178
TCEAL4	0,001	0,006	0,058	0,164	0,214	0,384	945,464	175,869
TMEM64	0,004	0,006	0,059	0,221	0,129	-0,771	229,799	39,148
GTF3C4	0,001	0,006	0,059	0,154	0,118	-0,388	1220,019	161,623
UBR4	0,001	0,006	0,06	0,212	0,242	0,193	5206,595	1176,641
BUB1	0,001	0,006	0,06	0,265	0,311	0,229	2412,854	695,805
CUL3	0,001	0,006	0,06	0,164	0,207	0,331	1283,852	240,44
TMEM106C	0,001	0,006	0,06	0,152	0,203	0,415	810,407	144,611
SLC2A1	0,001	0,006	0,061	0,506	0,564	0,159	6509,066	3473,375
PRDX6	0,001	0,006	0,061	0,633	0,537	-0,238	1201,004	699,268
EMC3	0,001	0,006	0,061	0,492	0,632	0,362	447,536	251,107
LYPLA1	0,003	0,006	0,061	0,214	0,139	-0,623	348,428	60,58
RECQL	0,002	0,006	0,061	0,098	0,143	0,547	635,67	77,167
SPG20	0,001	0,006	0,061	0,184	0,235	0,353	1059,275	217,801
PSMA7	0,001	0,006	0,061	0,566	0,655	0,211	1814,439	1107,654
MLPH	0,002	0,007	0,062	0,154	0,218	0,504	505,746	92,958
UGT8	0,002	0,006	0,062	0,303	0,431	0,507	285,739	103,933
SRRT	0,001	0,006	0,062	0,081	0,102	0,333	2376,75	215,12

RPS20	0,001	0,007	0,062	0,609	0,746	0,292	656,024	442,444
TCF3	0,002	0,007	0,062	0,102	0,074	-0,472	1179,575	104,068
UCKL1	0,001	0,006	0,062	0,355	0,251	-0,501	384,419	121,969
CPVL	0,003	0,007	0,063	0,477	0,859	0,847	64,756	43,991
YEATS4	0,002	0,007	0,063	0,291	0,41	0,494	312,583	109,416
HN1	0,001	0,007	0,063	0,768	0,65	-0,24	1084,584	772,772
GPX4	0,001	0,007	0,063	0,153	0,188	0,296	1877,889	318,396
C1orf85	0,001	0,007	0,064	0,412	0,558	0,438	315,56	151,341
PDE12	0,001	0,007	0,064	0,265	0,21	-0,333	1035,163	243,114
PAPD4	0,003	0,007	0,064	0,118	0,185	0,647	353,372	53,55
SLC12A9	0,002	0,007	0,064	0,417	0,284	-0,553	249,158	87,293
CDC123	0,001	0,007	0,064	0,237	0,293	0,309	1131,029	297,716
ABHD14B	0,002	0,007	0,065	0,238	0,159	-0,582	356,314	71,206
C4orf3	0,002	0,007	0,065	0,538	0,369	-0,545	207,711	92,996
STOML2	0,001	0,007	0,065	0,399	0,332	-0,263	1302,029	475,679
PPHLN1	0,002	0,007	0,065	0,132	0,189	0,523	523,29	83,403
ENSG00000259001.2	0,001	0,007	0,065	12,843	10,535	-0,286	259,95	3012,646
LSMD1	0,001	0,007	0,065	2,257	1,818	-0,312	307,082	630,476
LGALS8	0,002	0,007	0,066	0,126	0,175	0,479	660,96	98,618
SERPINE2	0,001	0,007	0,066	0,364	0,474	0,382	464,689	194,63
MAN2B2	0,001	0,007	0,066	0,121	0,163	0,428	913,042	127,38
HIBADH	0,002	0,007	0,066	0,36	0,25	-0,527	309,715	96,338
GUSB	0,001	0,007	0,066	0,336	0,423	0,334	677,183	257,662
CUL2	0,001	0,007	0,066	0,234	0,292	0,319	998,858	265,107
EIF4G2	0,001	0,007	0,066	0,097	0,109	0,174	16762,78	1724,133
CCDC85C	0,002	0,007	0,066	0,108	0,072	-0,588	719,975	65,605
PMM2	0,003	0,007	0,066	0,271	0,425	0,651	176,86	59,659
SEMA6B	0,004	0,007	0,066	0,196	0,113	-0,799	241,443	38,879
POLDIP3	0,002	0,007	0,066	0,1	0,072	-0,465	1181,768	101,312
THOC7	0,001	0,007	0,067	0,515	0,646	0,327	526,278	303,548
PPP1R2	0,003	0,007	0,067	0,264	0,161	-0,715	217,504	46,563
HECTD4	0,002	0,007	0,067	0,121	0,083	-0,543	727,827	74,677

BRI3BP	0,002	0,007	0,067	0,162	0,115	-0,492	672,775	91,335
NLGN2	0,002	0,007	0,067	0,058	0,08	0,468	1422,943	97,095
UQCC2	0,002	0,008	0,068	1,033	0,692	-0,579	117,227	99,87
WASL	0,002	0,008	0,068	0,195	0,129	-0,587	403,186	64,216
TULP3	0,003	0,008	0,068	0,093	0,172	0,887	331,521	52,368
KRT81	0,001	0,008	0,068	0,614	0,457	-0,427	390,083	221,881
SRSF5	0,001	0,007	0,068	0,156	0,193	0,31	1523,573	266,752
RPL3	0,001	0,008	0,068	0,217	0,247	0,19	4481,907	1033,219
PTPRM	0,002	0,008	0,069	0,076	0,104	0,449	1186,923	107,364
DSTN	0,001	0,008	0,069	0,303	0,238	-0,352	800,304	211,908
PODXL	0,001	0,008	0,07	0,18	0,214	0,25	2324,194	458,126
MTRNR2L3	0,002	0,008	0,07	1,826	1,064	-0,779	48,282	68,106
BCOR	0,002	0,008	0,07	0,09	0,132	0,551	633,522	69,315
BRCA2	0,002	0,008	0,071	0,059	0,083	0,493	1214,311	86,434
EXOC7	0,001	0,008	0,071	0,13	0,168	0,375	1122,039	166,511
ELOVL7	0,003	0,008	0,072	0,508	0,32	-0,668	140,915	57,005
CLDN12	0,001	0,008	0,072	0,269	0,2	-0,423	580,631	137,165
MPP5	0,001	0,008	0,072	0,095	0,125	0,392	1300,587	142,545
GPR56	0,001	0,008	0,072	0,215	0,276	0,364	767,606	186,796
RPS16	0,001	0,008	0,072	1,531	1,358	-0,173	1814,637	2616,204
COL6A1	0,001	0,008	0,072	0,153	0,207	0,434	683,017	126,107
PABPC4	0,001	0,008	0,073	0,098	0,117	0,258	3738,726	402,393
KIAA1143	0,003	0,008	0,073	0,323	0,193	-0,741	165,369	40,945
EPB41L4B	0,005	0,008	0,073	0,118	0,066	-0,827	353,497	33,34
ZDHHC16	0,002	0,008	0,073	0,116	0,08	-0,524	780,472	76,272
ARL6IP1	0,002	0,008	0,073	0,028	0,04	0,516	2223,508	75,644
WDR44	0,001	0,008	0,073	0,225	0,298	0,406	569,378	148,037
CNIH4	0,001	0,009	0,074	0,814	0,625	-0,382	309,184	222,01
INPP4B	0,002	0,008	0,074	0,102	0,146	0,525	632,866	76,343
LTV1	0,001	0,008	0,074	0,22	0,298	0,441	471,141	123,17
UBE2N	0,001	0,008	0,074	0,397	0,306	-0,374	539,92	188,795
FBN1	0,002	0,009	0,074	0,107	0,157	0,551	534,162	69,119

SUSD2	0,003	0,009	0,074	0,164	0,35	1,095	189,842	62,871
GPD1L	0,003	0,009	0,075	0,183	0,117	-0,642	349,67	52,442
GLRX3	0,001	0,009	0,075	0,612	0,482	-0,344	465,861	250,644
RPLP1	0,001	0,009	0,075	1,334	1,082	-0,302	389,238	467,413
PSMB6	0,001	0,009	0,075	1,25	1,099	-0,185	1620,155	1898,782
PNPO	0,002	0,009	0,075	0,515	0,345	-0,577	181,676	76,205
CLTC	0,001	0,009	0,075	0,125	0,142	0,178	7996,965	1071,829
GADD45B	0,001	0,009	0,075	0,32	0,436	0,446	347,378	128,978
RBM39	0,001	0,009	0,075	0,106	0,129	0,288	2474,248	290,134
HPCAL1	0,001	0,009	0,076	0,216	0,173	-0,321	1238,806	243,182
CAPN7	0,002	0,009	0,076	0,097	0,137	0,501	722,369	85,015
CBFB	0,001	0,009	0,076	0,317	0,252	-0,333	803,398	224,698
HS1BP3	0,004	0,009	0,077	0,297	0,163	-0,867	139,302	33,709
RIC8A	0,001	0,009	0,077	0,181	0,153	-0,244	2821,019	469,622
HEXIM1	0,001	0,009	0,077	0,17	0,214	0,334	1115,595	213,903
ZNF460	0,002	0,009	0,077	1,025	1,619	0,66	67,735	86,785
USP9X	0,001	0,009	0,077	0,114	0,145	0,34	1537,6	199,604
RPS23	0,001	0,009	0,078	1,89	1,61	-0,231	704,544	1233,751
SDC2	0,002	0,009	0,078	0,331	0,239	-0,471	414,569	110,191
RNF20	0,001	0,009	0,078	0,117	0,154	0,394	1056,534	142,073
RAB8A	0,001	0,009	0,078	0,287	0,36	0,331	731,729	236,025
CTSA	0,001	0,009	0,078	0,336	0,396	0,238	1503,617	544,528
SLC30A5	0,002	0,009	0,079	0,309	0,221	-0,479	382,442	100,048
RCE1	0,002	0,009	0,079	0,53	0,371	-0,516	217,005	97,56
ZMYM2	0,002	0,009	0,079	0,091	0,132	0,536	639,817	72,646
NR2F2	0,001	0,009	0,079	0,197	0,235	0,253	2024,368	428,485
NQO1	0,001	0,009	0,079	0,592	0,526	-0,17	3603,94	1998,667
MAN1A2	0,003	0,01	0,08	0,062	0,093	0,59	726,494	56,893
PTPRG	0,001	0,01	0,08	0,144	0,178	0,311	1468,443	238,619
PUS7	0,001	0,01	0,08	0,223	0,299	0,422	498,849	129,327
DUSP6	0,003	0,01	0,08	0,172	0,261	0,603	274,404	59,514
POMP	0,001	0,01	0,08	1,617	1,378	-0,231	666,11	988,014

OAT	0,001	0,01	0,081	0,516	0,431	-0,26	973,265	452,935
GOLT1B	0,001	0,01	0,082	0,39	0,3	-0,375	518,967	174,881
AHSA1	0,001	0,01	0,082	0,305	0,362	0,244	1489,144	493,113
PKN1	0,001	0,01	0,082	0,108	0,088	-0,297	2730,256	267,845
MRP63	0,001	0,01	0,083	0,559	0,438	-0,351	463,05	230,981
NFIC	0,001	0,01	0,083	0,063	0,078	0,323	3075,949	211,503
TAF7	0,001	0,01	0,084	0,136	0,176	0,372	1005,438	155,549
GLT8D1	0,002	0,01	0,085	0,186	0,264	0,505	378,208	85,482
SMARCA5	0,001	0,01	0,085	0,117	0,144	0,305	1827,066	239,569
MEPCE	0,002	0,01	0,085	0,1	0,136	0,438	895,975	106,156
PWP1	0,001	0,01	0,085	0,202	0,258	0,352	808,127	184,677
ENSG00000248527.1	0,001	0,011	0,087	0,575	0,704	0,291	586,161	368,831
EFEMP1	0,002	0,011	0,087	0,396	0,627	0,663	139,523	63,995
NAMPT	0,002	0,011	0,087	0,17	0,242	0,513	390,047	80,19
EEA1	0,002	0,011	0,087	0,101	0,142	0,491	688,004	86,043
RPS11	0,001	0,011	0,087	0,658	0,738	0,165	4257,317	2961,007
ITGB3BP	0,002	0,011	0,088	0,363	0,524	0,53	196,552	88,052
UQCRQ	0,001	0,011	0,088	1,609	1,284	-0,325	279,316	402,259
HM13	0,001	0,011	0,088	0,65	0,729	0,165	3306,873	2270,353
TXLNG	0,003	0,011	0,088	0,09	0,139	0,621	435,988	49,786
MRPL37	0,001	0,011	0,089	0,299	0,255	-0,228	1925,693	535,295
NUCKS1	0,001	0,011	0,089	0,118	0,136	0,212	4759,159	600,497
METTL5	0,001	0,011	0,089	0,434	0,58	0,417	295,043	148,61
MAEA	0,001	0,011	0,089	0,379	0,307	-0,302	825,425	281,2
SIPA1	0,001	0,011	0,089	0,19	0,144	-0,402	798,106	133,663
TYK2	0,001	0,011	0,089	0,109	0,082	-0,408	1276,438	122,728
URI1	0,002	0,011	0,089	0,177	0,128	-0,474	601,367	89,804
ZCCHC11	0,002	0,011	0,09	0,074	0,103	0,467	994,394	87,706
MTPN	0,001	0,011	0,09	0,237	0,194	-0,283	1399,797	300,223
ZNF503	0,002	0,011	0,09	0,272	0,395	0,539	242,528	77,851
SOCS4	0,003	0,011	0,09	0,079	0,052	-0,602	779,525	50,891
NPC2	0,001	0,011	0,09	1,006	1,221	0,28	451,445	498,269

RPF1	0,001	0,011	0,091	0,208	0,266	0,353	763,943	179,194
CLCC1	0,002	0,012	0,091	0,336	0,466	0,471	266,614	106,328
LAMB3	0,001	0,011	0,091	0,226	0,267	0,242	1921,655	471,199
RAI14	0,001	0,012	0,091	0,075	0,095	0,333	2134,489	180,877
TTC1	0,001	0,012	0,091	0,197	0,25	0,342	862,867	193,222
GNG11	0,002	0,012	0,091	0,391	0,568	0,54	174,338	84,363
STIM1	0,002	0,012	0,091	0,144	0,196	0,441	653,834	108,137
PTPRJ	0,002	0,011	0,091	0,184	0,249	0,437	550,998	114,701
DDX6	0,002	0,011	0,091	0,208	0,304	0,55	274,959	69,87
SCARB1	0,001	0,012	0,091	0,286	0,353	0,302	883,94	277,629
FLOT2	0,002	0,012	0,091	0,168	0,122	-0,46	664,298	96,924
NEDD4L	0,001	0,011	0,091	0,08	0,098	0,291	2828,155	248,834
YIPF6	0,001	0,012	0,091	0,274	0,212	-0,369	690,339	166,389
LNPEP	0,001	0,012	0,092	0,247	0,311	0,333	756,344	211,835
HIATL1	0,001	0,012	0,092	0,181	0,142	-0,351	1078,04	173,319
SMC3	0,001	0,012	0,092	0,059	0,075	0,338	2620,803	176,414
ZBED4	0,001	0,012	0,092	0,071	0,055	-0,383	2118,449	134,43
RPL6	0,001	0,012	0,093	0,356	0,452	0,346	511,283	207,249
LSM7	0,001	0,012	0,093	1,735	1,377	-0,334	247,461	381,809
COL6A2	0,001	0,012	0,093	0,258	0,326	0,339	705,049	204,013
TAF13	0,001	0,012	0,094	0,499	0,371	-0,428	310,167	130,788
PPP2CB	0,001	0,012	0,094	0,481	0,368	-0,384	394,463	164,402
KIAA0020	0,002	0,012	0,094	0,067	0,093	0,482	994,096	78,9
TMEM203	0,003	0,012	0,094	0,259	0,169	-0,616	252,207	53,368
LPCAT4	0,002	0,012	0,094	0,201	0,133	-0,591	354,378	60,819
PRR11	0,001	0,012	0,094	0,155	0,185	0,257	2158,615	368,402
LMNB1	0,001	0,012	0,095	0,217	0,257	0,246	1754,494	418,569
XPO7	0,001	0,012	0,095	0,193	0,161	-0,254	2078,785	364,005
ZZEF1	0,001	0,012	0,095	0,113	0,089	-0,35	1681,975	169,324
AKAP12	0,001	0,013	0,096	0,166	0,212	0,352	984,752	180,198
TMEM2	0,001	0,013	0,096	0,142	0,185	0,384	865,459	139,383
TSC22D1	0,001	0,012	0,096	0,276	0,227	-0,282	1206,506	304,578

VAT1	0,001	0,013	0,096	0,257	0,219	-0,228	2265,248	544,724
DDA1	0,001	0,013	0,096	0,178	0,223	0,33	1011,508	199,311
ADAMTS1	0,001	0,013	0,096	0,267	0,343	0,36	718,984	206,889
ALG6	0,001	0,013	0,097	0,573	0,763	0,412	239,919	159,591
DR1	0,001	0,013	0,097	0,243	0,199	-0,289	1246,781	275,965
UBE2E2	0,002	0,013	0,097	0,395	0,278	-0,504	259,123	86,223
SERP1	0,001	0,013	0,097	0,314	0,254	-0,306	870,247	245,088
SERPINH1	0,001	0,013	0,097	0,572	0,647	0,18	2179,208	1326,461
EIF3K	0,001	0,013	0,097	0,646	0,756	0,229	1024,269	718,726
ERCC2	0,001	0,013	0,097	0,232	0,185	-0,324	997,645	208,678
EMP3	0,001	0,013	0,097	1,4	1,2	-0,223	726,124	953,54
TGM2	0,001	0,013	0,097	0,117	0,099	-0,241	4166,115	460,674
MGA	0,002	0,013	0,098	0,068	0,096	0,489	925,922	75,552
PFN2	0,001	0,013	0,099	0,293	0,226	-0,373	611,481	155,452
PTDSS1	0,001	0,013	0,099	0,353	0,299	-0,242	1386,44	450,598
SMCR8	0,002	0,013	0,099	0,087	0,12	0,467	828,993	84,881
RPL38	0,001	0,013	0,099	0,905	1,024	0,178	1892,819	1813,806
USP33	0,002	0,014	0,101	0,205	0,155	-0,401	701,845	123,438
UBE2Q1	0,001	0,014	0,101	0,175	0,136	-0,364	989,803	153,605
STK3	0,002	0,014	0,101	0,188	0,277	0,557	277,498	65,868
MYCBP2	0,002	0,014	0,101	0,071	0,052	-0,444	1485,128	90,565
DALRD3	0,004	0,014	0,102	0,427	0,258	-0,726	119,059	40,034
FBRS	0,002	0,014	0,102	0,054	0,078	0,517	1005,957	65,47
SMARCA4	0,001	0,014	0,102	0,148	0,172	0,208	3746,529	597,629
PPT1	0,001	0,014	0,103	0,287	0,328	0,192	2649,914	815,948
MRPS16	0,001	0,014	0,103	0,504	0,41	-0,298	638,279	288,808
OTUB1	0,001	0,014	0,103	0,414	0,499	0,27	811,812	369,631
STAMBP	0,001	0,014	0,104	0,22	0,183	-0,266	1548,957	312,5
GPR126	0,001	0,014	0,104	0,143	0,18	0,334	1116,447	182,325
LANCL2	0,002	0,014	0,104	0,295	0,431	0,544	203,116	72,314
YWHAZ	0,001	0,014	0,104	0,084	0,069	-0,292	2943,864	222,815
AKAP13	0,002	0,014	0,104	0,13	0,18	0,467	554,897	87,263

OSGIN1	0,003	0,014	0,104	0,176	0,267	0,602	250,211	53,427
ENSG00000210100.1	0,003	0,014	0,104	1,992	4,1	1,041	16,972	51,184
GPBP1	0,002	0,014	0,105	0,097	0,133	0,458	758,656	87,933
SOD2	0,001	0,014	0,105	0,388	0,312	-0,316	668,36	230,353
TMC6	0,003	0,014	0,105	0,198	0,134	-0,564	358,214	58,376
RSRC1	0,002	0,015	0,106	0,323	0,221	-0,549	246,811	65,485
TRA2B	0,001	0,015	0,106	0,277	0,322	0,22	1952,268	584,899
C4orf48	0,002	0,015	0,106	3,062	1,982	-0,627	50,053	123,699
AGA	0,003	0,015	0,106	0,467	0,748	0,681	88,638	51,673
OXCT1	0,004	0,014	0,106	0,225	0,139	-0,694	216,535	39,171
SAR1B	0,001	0,015	0,106	0,583	0,436	-0,419	277,901	136,602
PIP4K2C	0,002	0,015	0,106	0,217	0,153	-0,5	417,731	77,221
ERP29	0,001	0,015	0,106	0,354	0,428	0,274	857,242	331,693
MYO5A	0,003	0,014	0,106	0,047	0,07	0,569	920,501	53,779
MYBBP1A	0,001	0,015	0,106	0,122	0,104	-0,223	4372,847	496,637
SYF2	0,002	0,015	0,107	0,3	0,405	0,433	326,893	115,442
TMEM87B	0,002	0,015	0,107	0,234	0,326	0,48	313,853	87,899
PDHB	0,001	0,015	0,107	0,697	0,583	-0,257	693,553	442,576
SUMF2	0,001	0,015	0,107	0,305	0,25	-0,288	1003,847	279,563
SSBP1	0,002	0,015	0,107	0,278	0,386	0,473	281,818	93,095
DCAF12	0,002	0,015	0,107	0,127	0,089	-0,509	643,427	69,241
VPS51	0,001	0,015	0,107	0,405	0,322	-0,332	574,535	207,793
EI24	0,002	0,015	0,107	0,096	0,07	-0,446	1085,986	89,649
MARS	0,001	0,015	0,107	0,467	0,533	0,19	2134,006	1063,099
SNRPF	0,001	0,015	0,107	1,601	2,037	0,348	197,277	355,822
PPP1CC	0,001	0,015	0,107	0,236	0,204	-0,211	2537,623	551,977
GART	0,001	0,015	0,107	0,198	0,241	0,279	1267,539	279,011
C3orf38	0,002	0,015	0,108	0,199	0,275	0,464	387,511	91,076
TRMT10C	0,002	0,015	0,108	0,276	0,192	-0,525	304,647	69,954
LRBA	0,002	0,015	0,108	0,129	0,173	0,419	712,928	107,155
RPL8	0,001	0,015	0,108	0,434	0,478	0,138	8226,108	3747,48
ANAPC2	0,002	0,015	0,109	0,256	0,19	-0,43	474,717	104,995

YRDC	0,002	0,016	0,11	0,36	0,268	-0,425	368,54	115,456
TTC13	0,002	0,016	0,11	0,243	0,344	0,5	272,305	79,274
CCDC80	0,002	0,016	0,11	0,059	0,084	0,513	903,654	65,035
SEC24B	0,001	0,016	0,11	0,196	0,251	0,353	722,457	160,643
C4orf46	0,003	0,016	0,11	0,192	0,128	-0,581	339,18	53,663
EEF1A1	0,001	0,016	0,11	0,165	0,2	0,28	1472,951	269,44
MDH2	0,001	0,016	0,11	0,173	0,219	0,341	905,488	173,972
AZIN1	0,001	0,016	0,11	0,139	0,167	0,262	2096,416	322,024
TBK1	0,002	0,016	0,11	0,159	0,229	0,529	342,671	66,028
CDK2AP1	0,002	0,016	0,11	0,878	0,579	-0,599	99,493	69,466
U2AF1	0,001	0,016	0,11	0,397	0,47	0,243	1022,967	440,944
MAST2	0,002	0,016	0,111	0,061	0,044	-0,48	1450,856	77,375
QRICH1	0,001	0,016	0,111	0,258	0,302	0,227	1721,729	480,789
SSR3	0,001	0,016	0,111	0,382	0,34	-0,168	3469,506	1253,297
GLRX5	0,002	0,016	0,111	0,43	0,32	-0,427	317,469	118,871
R3HDM4	0,002	0,016	0,111	0,192	0,141	-0,447	556,063	91,742
RBM3	0,001	0,016	0,111	1,381	1,077	-0,359	215,276	260,201
PTK7	0,001	0,016	0,112	0,299	0,369	0,303	737,962	244,64
ARGLU1	0,002	0,016	0,112	0,065	0,092	0,502	866,342	67,162
EIF2AK2	0,003	0,016	0,113	0,044	0,065	0,556	979,989	52,458
CCNJL	0,004	0,016	0,113	0,135	0,084	-0,687	367,053	41,286
NFE2L3	0,001	0,016	0,113	0,207	0,266	0,363	634,707	151,683
ZMIZ1	0,002	0,016	0,113	0,067	0,089	0,4	1440,825	110,031
NUTF2	0,001	0,016	0,113	0,406	0,481	0,242	979,873	434,677
RAI1	0,002	0,016	0,113	0,079	0,06	-0,41	1495,66	104,943
CNN2	0,001	0,016	0,113	0,289	0,25	-0,206	2512,723	680,715
MRPL9	0,001	0,017	0,115	0,239	0,313	0,392	472,413	130,468
NIT2	0,002	0,017	0,115	0,761	0,55	-0,468	173,153	113,766
FKBP14	0,003	0,017	0,115	0,132	0,196	0,57	330,598	54,566
KDELR1	0,001	0,017	0,115	0,614	0,705	0,197	1290,025	853,389
ERLEC1	0,002	0,017	0,116	0,137	0,193	0,493	442,899	72,934
BRPF1	0,003	0,017	0,116	0,057	0,084	0,561	750,085	52,079

ITGA2	0,002	0,017	0,116	0,185	0,251	0,441	459,331	97,731
GM2A	0,002	0,017	0,116	0,338	0,252	-0,425	373,899	109,299
PVRL2	0,001	0,017	0,116	0,232	0,289	0,315	807,237	207,592
SCAMP1	0,002	0,017	0,117	0,356	0,266	-0,419	372,116	114,766
GNL2	0,001	0,017	0,118	0,096	0,122	0,338	1474,945	161,245
DPY19L1	0,002	0,017	0,118	0,152	0,2	0,392	680,049	120,711
COG4	0,001	0,017	0,118	0,262	0,329	0,327	649,521	193,102
GOLPH3L	0,003	0,018	0,119	0,165	0,243	0,557	282,685	57,851
SLC12A2	0,001	0,018	0,119	0,261	0,34	0,385	445,713	134,701
NBN	0,002	0,018	0,119	0,11	0,147	0,421	779,408	101,702
EXOSC3	0,001	0,018	0,119	0,574	0,435	-0,4	281,738	141,518
PSPC1	0,002	0,018	0,119	0,182	0,247	0,441	442,137	94,742
MYH10	0,001	0,018	0,119	0,07	0,085	0,291	2974,713	231,182
TNPO2	0,001	0,018	0,119	0,131	0,158	0,264	2052,816	295,867
TPRG1L	0,003	0,018	0,12	0,248	0,161	-0,627	226,657	46,691
TRIM44	0,002	0,018	0,12	0,078	0,108	0,474	811,081	75,056
STT3B	0,001	0,018	0,121	0,379	0,434	0,197	1788,809	735,249
CTCF	0,001	0,018	0,121	0,161	0,199	0,31	1117,478	202,62
PLIN3	0,001	0,018	0,121	0,695	0,615	-0,175	1780,55	1172,967
EIF5	0,001	0,018	0,122	0,131	0,155	0,24	2852,619	410,567
N4BP1	0,002	0,018	0,122	0,134	0,186	0,468	506,517	80,178
HDGF	0,001	0,019	0,123	0,107	0,093	-0,194	5833,821	584,455
RRP15	0,001	0,018	0,123	0,275	0,36	0,386	426,921	133,648
RPL37A	0	0,018	0,123	1,726	1,562	-0,144	2056,292	3392,35
RNF123	0,003	0,019	0,124	0,142	0,098	-0,536	486,122	56,972
ROCK2	0,001	0,019	0,125	0,093	0,12	0,373	1142,856	122,002
CMTM7	0,002	0,019	0,125	0,305	0,226	-0,428	393,581	105,227
GTPBP8	0,002	0,019	0,125	0,254	0,371	0,548	201,584	62,461
ANXA5	0,001	0,019	0,125	0,311	0,357	0,198	2415,081	788,588
VCP	0,001	0,019	0,125	0,128	0,146	0,199	5181,885	710,612
SAR1A	0,001	0,019	0,125	0,326	0,279	-0,225	1603,47	481,656
NSMCE4A	0,002	0,019	0,125	0,291	0,409	0,49	232,386	80,939

NRGN	0,003	0,019	0,125	0,186	0,281	0,596	224,285	50,304
COX14	0,003	0,019	0,125	1,222	0,747	-0,709	54,953	53,386
ARPC3	0,001	0,019	0,125	0,628	0,479	-0,392	311,854	161,934
ELF1	0,002	0,019	0,125	0,175	0,237	0,433	470,619	95,689
TM9SF2	0,001	0,019	0,125	0,582	0,657	0,175	1802,08	1125,633
EIF3D	0,001	0,019	0,125	0,141	0,158	0,17	6502,139	969,752
FRRS1	0,003	0,019	0,126	0,688	1,095	0,671	63,867	56,4
SUCLG1	0,001	0,019	0,126	0,605	0,529	-0,195	1499,42	850,331
FAM177A1	0,003	0,019	0,126	0,334	0,218	-0,617	177,815	49,091
BUB1B	0,001	0,019	0,126	0,1	0,129	0,374	1075,491	122,381
FBXL12	0,002	0,019	0,126	0,27	0,387	0,52	219,976	70,304
SNRPD2	0,001	0,019	0,126	1,763	1,513	-0,221	575,007	939,504
LEPREL1	0,001	0,02	0,128	0,164	0,207	0,337	861,864	159,839
CLPTM1L	0,001	0,02	0,128	0,233	0,195	-0,262	1412,068	298,538
AKAP9	0,003	0,02	0,128	0,036	0,053	0,563	1103,818	48,975
C16orf58	0,002	0,02	0,128	0,694	0,513	-0,438	198,401	119,624
MTMR4	0,002	0,02	0,128	0,108	0,082	-0,407	1054,892	99,695
SEP6	0,002	0,02	0,128	0,128	0,179	0,487	461,393	70,877
POLK	0,003	0,02	0,129	0,115	0,17	0,565	361,581	51,942
MEA1	0,001	0,02	0,129	0,435	0,339	-0,361	408,595	157,076
SEMA3E	0,003	0,02	0,129	0,146	0,216	0,57	306,741	52,87
DAPK3	0,001	0,02	0,129	0,214	0,27	0,334	709,678	169,275
PABPC1	0,001	0,02	0,13	0,033	0,039	0,236	8843,829	318,985
RPP25L	0,002	0,02	0,13	1,296	0,834	-0,636	64,164	67,817
CARS	0,001	0,02	0,131	0,184	0,223	0,275	1297,698	263,106
LDHA	0,001	0,02	0,131	0,292	0,262	-0,157	6780,158	1880,203
TRAPPC1	0,001	0,02	0,131	0,877	1,04	0,246	568,776	534,441
NOL11	0,001	0,02	0,131	0,255	0,204	-0,323	795,734	178,921
MRPS28	0,001	0,021	0,132	1,373	1,033	-0,411	147,94	175,47
GPAA1	0,003	0,021	0,132	0,454	0,288	-0,654	120,954	43,649
KLF6	0,001	0,021	0,132	0,181	0,145	-0,32	1064,646	172,77
COG8	0,003	0,021	0,132	0,34	0,221	-0,624	167,437	45,078

TPM4	0,001	0,021	0,132	0,161	0,139	-0,212	3200,173	481,714
FAM129A	0,001	0,021	0,133	0,123	0,154	0,316	1295,252	177,213
INO80B	0,002	0,021	0,133	0,329	0,472	0,517	180,658	72,741
GATA2	0,002	0,021	0,133	0,164	0,233	0,506	336,822	66,182
HINT1	0,001	0,021	0,133	1,445	1,67	0,208	658,558	1020,077
CTDNEP1	0,002	0,021	0,133	0,082	0,11	0,417	978,298	92,243
ENSG00000210164.1	0,001	0,021	0,133	2,586	3,963	0,616	42,195	137,305
POLR2A	0,001	0,021	0,134	0,086	0,099	0,199	7253,249	662,865
TBCD	0,001	0,021	0,134	0,247	0,202	-0,288	1052,667	237,11
KDM4B	0,003	0,021	0,134	0,135	0,091	-0,571	430,157	47,659
ELL2	0,003	0,022	0,135	0,088	0,06	-0,544	707,149	51,977
CYHR1	0,002	0,022	0,135	0,18	0,127	-0,504	424,048	64,277
SLC35F2	0,001	0,022	0,135	0,374	0,31	-0,269	879,346	298,885
PDS5A	0,001	0,022	0,136	0,119	0,143	0,259	2052,32	272,278
F2RL1	0,001	0,022	0,136	0,441	0,355	-0,312	581,991	237,296
DCTN4	0,001	0,022	0,136	0,174	0,141	-0,297	1280,146	200,841
FNBP4	0,002	0,022	0,136	0,086	0,118	0,452	762,65	76,709
GNB1	0,001	0,022	0,138	0,206	0,182	-0,181	3741,304	724,289
C1orf86	0,003	0,022	0,138	0,183	0,119	-0,615	287,662	43,927
DTL	0,001	0,022	0,138	0,195	0,156	-0,323	950,878	166,322
TGOLN2	0,001	0,022	0,138	0,147	0,167	0,188	5343,838	840,273
HSPA4	0,001	0,022	0,138	0,199	0,227	0,192	3013,907	642,137
HIPK2	0,002	0,022	0,138	0,045	0,032	-0,463	1849,435	71,619
DECR1	0,001	0,022	0,138	0,469	0,609	0,376	286,733	152,032
DDX58	0,001	0,022	0,138	0,197	0,153	-0,368	749,244	126,514
DNAJC1	0,003	0,022	0,138	0,21	0,309	0,558	215,701	54,99
YIPF2	0,002	0,022	0,138	0,293	0,385	0,395	360,912	120,467
EXOSC10	0,001	0,023	0,139	0,105	0,131	0,32	1394,137	163,074
MRPL55	0,001	0,023	0,139	1,296	0,962	-0,43	135,943	154,463
MRPL36	0,002	0,023	0,139	1,506	1,012	-0,573	69,982	86,204
KIAA1191	0,002	0,022	0,139	0,072	0,054	-0,428	1336,625	84,199
UFL1	0,002	0,023	0,139	0,17	0,239	0,489	341,072	71,171

GOPC	0,002	0,023	0,139	0,167	0,122	-0,45	556,781	78,787
SEMA3C	0,001	0,022	0,139	0,161	0,206	0,356	742,168	139,217
TSR3	0,003	0,023	0,139	0,452	0,304	-0,57	152,962	57,307
MUM1	0,003	0,023	0,139	0,093	0,061	-0,597	563,275	43,632
C19orf52	0,002	0,023	0,139	0,868	0,592	-0,551	105,248	73,443
C1orf43	0,001	0,023	0,14	0,169	0,139	-0,282	1464,761	224,213
USB1	0,002	0,023	0,14	0,085	0,065	-0,377	1480,851	110,535
ICK	0,002	0,023	0,141	0,07	0,054	-0,372	1858,826	111,068
BRAT1	0,001	0,023	0,141	0,462	0,369	-0,325	468,581	194,918
CACUL1	0,002	0,023	0,141	0,116	0,085	-0,45	853,823	79,814
TMSB10	0,001	0,023	0,142	0,412	0,449	0,124	9801,49	4217,727
GGCT	0,002	0,023	0,142	0,272	0,202	-0,428	398,386	92,467
COA4	0,001	0,023	0,142	0,333	0,409	0,296	633,838	231,959
EXOSC7	0,002	0,024	0,143	0,916	0,674	-0,444	150,839	117,839
TMEM115	0,002	0,023	0,143	0,23	0,3	0,381	473,609	123,188
PHF10	0,003	0,024	0,143	0,11	0,075	-0,558	527,136	47,495
SLC25A37	0,002	0,024	0,143	0,067	0,089	0,412	1153,031	90,758
KIAA1737	0,003	0,024	0,143	0,145	0,096	-0,597	359,938	42,714
TELO2	0,002	0,024	0,143	0,307	0,229	-0,426	361,966	96,116
RPL23	0,001	0,024	0,143	1,088	1,219	0,164	1520,895	1753,461
GCC2	0,003	0,024	0,144	0,116	0,17	0,542	369,058	53,755
RPN1	0,001	0,024	0,144	0,412	0,464	0,171	2339,455	1023,035
PTPN12	0,001	0,024	0,144	0,098	0,117	0,266	2303,691	248,88
NOP56	0,001	0,024	0,144	0,158	0,183	0,214	2754,33	470,918
RPL22L1	0,001	0,024	0,145	1,198	1,588	0,406	132,228	183,116
MRFAP1	0,001	0,024	0,145	0,247	0,21	-0,232	1721,847	391,065
SLC22A5	0,005	0,024	0,145	0,267	0,163	-0,715	156,259	34,276
PPP6R3	0,001	0,024	0,145	0,116	0,138	0,247	2304,081	292,744
SLC25A3	0,001	0,024	0,145	0,559	0,505	-0,146	4437,169	2363,147
COMMD6	0,002	0,024	0,145	1,065	0,763	-0,48	117,143	105,569
RAB5A	0,002	0,025	0,146	0,18	0,233	0,377	579,793	117,576
SRP14	0,001	0,025	0,146	0,373	0,443	0,249	873,552	360,397

ALKBH5	0,001	0,024	0,146	0,105	0,084	-0,324	1617,226	151,037
FAM134C	0,001	0,025	0,146	0,315	0,249	-0,343	541,71	151,265
MED28	0,003	0,025	0,147	0,279	0,187	-0,578	211,876	48,232
ABCD4	0,002	0,025	0,147	0,22	0,168	-0,387	572,218	109,929
TTC19	0,002	0,025	0,147	0,153	0,118	-0,368	886,068	119,416
DDX50	0,002	0,025	0,148	0,106	0,145	0,452	593,306	74,112
CPSF7	0,001	0,025	0,148	0,083	0,102	0,302	1880,884	173,076
VPS4A	0,002	0,025	0,148	0,116	0,084	-0,465	693,723	68,702
AGTRAP	0,002	0,025	0,149	0,59	0,448	-0,397	250,22	130,368
TGFBRAP1	0,003	0,025	0,149	0,133	0,091	-0,545	456,264	50,967
QARS	0,001	0,025	0,149	0,237	0,272	0,202	2181,907	554,431
ARMC8	0,002	0,025	0,149	0,196	0,269	0,457	338,771	78,965
ETF1	0,001	0,025	0,149	0,143	0,172	0,272	1503,075	235,713
PPIL1	0,001	0,025	0,149	0,307	0,246	-0,316	662,858	183,195
PNISR	0,002	0,025	0,149	0,068	0,095	0,486	753,734	61,149
TMEM161A	0,001	0,025	0,149	0,735	0,598	-0,298	390,008	260,794
TNFRSF10D	0,001	0,025	0,15	0,296	0,231	-0,357	536,654	136,596
RSRC2	0,002	0,026	0,15	0,134	0,172	0,366	783,293	119,446
PHACTR4	0,002	0,026	0,152	0,078	0,111	0,497	620,693	59,299
FANCG	0,002	0,026	0,152	0,247	0,191	-0,376	534,124	115,745
POGK	0,001	0,026	0,153	0,184	0,144	-0,352	789,768	129,964
SFT2D1	0,001	0,026	0,153	0,585	0,719	0,298	389,185	253,33
TMEM60	0,003	0,026	0,153	0,924	0,581	-0,669	66,533	47,164
PREB	0,002	0,027	0,154	0,177	0,135	-0,393	655,16	101,439
MFSD10	0,001	0,027	0,154	0,468	0,374	-0,326	438,347	185,068
FRMD8	0,003	0,027	0,154	0,195	0,136	-0,521	343,691	55,731
SLC46A3	0,002	0,026	0,154	0,284	0,394	0,474	233,265	77,093
MYEF2	0,002	0,026	0,154	0,179	0,235	0,397	492,899	102,643
SNRPA	0,001	0,026	0,154	0,287	0,342	0,256	931,981	292,615
LCP1	0,004	0,027	0,155	0,111	0,191	0,787	251,509	31,639
CCDC57	0,003	0,027	0,155	0,108	0,072	-0,584	473,327	42,808
PIGT	0,001	0,027	0,155	0,237	0,278	0,228	1556,548	397,016

GNAI2	0,001	0,027	0,156	0,272	0,337	0,31	638,672	191,108
FBXO7	0,001	0,027	0,156	0,224	0,187	-0,26	1351,022	277,796
NOP14	0,002	0,027	0,157	0,093	0,119	0,361	1092,82	117,357
CNTRL	0,003	0,027	0,157	0,061	0,088	0,533	659,71	49,554
TRMT112	0,001	0,027	0,157	2,489	2,034	-0,291	215,419	482,067
PSMA3	0,001	0,027	0,157	1,382	1,212	-0,19	778,5	1001,888
SMG9	0,002	0,027	0,157	0,125	0,167	0,415	598,849	87,771
PEA15	0,001	0,028	0,158	0,064	0,075	0,226	5051,727	344,811
TACC3	0,001	0,028	0,158	0,361	0,41	0,184	1870,442	721,122
TNIP1	0,001	0,027	0,158	0,118	0,147	0,32	1140,179	150,931
POLR3E	0,002	0,028	0,158	0,137	0,097	-0,505	513,376	61,319
BECN1	0,002	0,027	0,158	0,162	0,211	0,385	581,378	106,644
PSME3	0,001	0,028	0,158	0,171	0,193	0,181	3461,644	629,942
DLG1	0,001	0,028	0,159	0,108	0,132	0,29	1552,556	185,971
RPS24	0,001	0,028	0,159	1,573	1,756	0,159	1182,723	1968,986
HIP1R	0,002	0,028	0,159	0,146	0,104	-0,489	496,925	62,352
C14orf2	0,001	0,028	0,159	2,081	1,704	-0,288	236,406	447,302
TUBB6	0,001	0,028	0,159	0,24	0,269	0,168	3955,431	997,706
TICAM1	0,003	0,028	0,159	0,219	0,326	0,572	197,817	50,713
ENTPD6	0,002	0,028	0,159	0,12	0,086	-0,487	597,442	61,388
REEP5	0,001	0,028	0,161	0,469	0,401	-0,226	951,498	413,31
C1QBP	0,001	0,028	0,161	0,805	0,735	-0,131	3327,178	2559,33
EPHB2	0,002	0,029	0,162	0,162	0,219	0,438	475,063	85,985
ODF2L	0,002	0,029	0,162	0,13	0,182	0,492	398,96	60,853
NRBP1	0,001	0,029	0,162	0,265	0,318	0,264	889,481	258,75
GOLIM4	0,002	0,029	0,162	0,107	0,144	0,436	612,583	76,787
CCT6A	0,001	0,029	0,162	0,253	0,286	0,177	2714,629	732,947
SBDS	0,001	0,029	0,162	0,164	0,204	0,312	901,974	165,738
LEPROTL1	0,001	0,029	0,162	0,44	0,346	-0,347	385,61	149,399
PSAT1	0,001	0,029	0,162	0,419	0,36	-0,219	1172,587	451,653
FTO	0,002	0,029	0,162	0,271	0,185	-0,548	265,186	64,271
SCPEP1	0,001	0,029	0,162	0,51	0,636	0,317	358,013	205,005

ISOC2	0,002	0,029	0,162	0,543	0,415	-0,387	266,404	127,944
SCAP	0,001	0,029	0,163	0,119	0,147	0,308	1255,563	165,528
STRIP2	0,003	0,029	0,163	0,171	0,115	-0,58	302,757	42,868
PFKP	0,001	0,029	0,163	0,243	0,216	-0,17	3734,113	861,469
NUDT19	0,001	0,029	0,163	0,593	0,46	-0,366	277,907	143,704
MAP7D1	0,001	0,029	0,164	0,103	0,122	0,246	2522,941	281,304
DNTTIP2	0,001	0,03	0,165	0,096	0,117	0,289	1804,599	191,175
EIF2A	0,001	0,03	0,165	0,243	0,289	0,25	1109,322	294,748
FAM171A1	0,003	0,03	0,165	0,081	0,057	-0,503	801,235	55,657
RHOG	0,001	0,03	0,165	0,257	0,321	0,318	589,536	167,848
RPL27A	0,001	0,03	0,165	0,502	0,574	0,192	1248,638	667,949
NUCB2	0,001	0,03	0,165	0,23	0,284	0,304	707,285	181,753
PPP2R1B	0,002	0,03	0,165	0,183	0,133	-0,465	432,47	67,165
CRY1	0,002	0,03	0,165	0,158	0,205	0,372	611,36	111,722
CHD8	0,002	0,03	0,165	0,076	0,097	0,345	1427,559	122,835
MAZ	0,002	0,03	0,165	0,245	0,33	0,428	307,42	88,495
RNF149	0,001	0,03	0,167	0,526	0,647	0,298	397,582	230,427
SLC39A10	0,001	0,03	0,167	0,215	0,272	0,335	590,904	143,99
ZFHX4	0,003	0,03	0,167	0,117	0,168	0,525	364,688	51,352
PDHX	0,001	0,03	0,167	0,306	0,247	-0,309	666,041	185,715
FURIN	0,003	0,03	0,167	0,08	0,055	-0,554	687,36	47,601
BRK1	0,001	0,031	0,168	0,343	0,274	-0,323	527,748	160,974
HACL1	0,002	0,031	0,168	0,319	0,449	0,491	181,628	70,066
LIMD1	0,002	0,031	0,168	0,14	0,108	-0,375	844,147	104,494
CRK	0,001	0,031	0,168	0,133	0,112	-0,241	2281,834	278,975
HEATR1	0,001	0,031	0,169	0,117	0,094	-0,323	1319,357	137,942
GCN1L1	0,001	0,031	0,169	0,076	0,064	-0,265	2980,188	207,608
RNF126	0,001	0,031	0,169	0,189	0,23	0,283	1005,751	208,758
SORT1	0,001	0,031	0,17	0,201	0,169	-0,254	1413,041	256,795
RIF1	0,001	0,031	0,17	0,118	0,14	0,253	1897,812	245,869
OBSL1	0,003	0,031	0,17	0,132	0,094	-0,489	518,269	58,543
CLDND1	0,002	0,031	0,17	0,193	0,15	-0,363	680,692	118,077

FBXO45	0,003	0,031	0,17	0,101	0,068	-0,577	484,528	40,915
NAF1	0,002	0,031	0,17	0,537	0,717	0,418	182,002	112,024
PLOD3	0,001	0,031	0,17	0,248	0,294	0,246	1095,481	293,955
POMK	0,003	0,031	0,17	0,84	1,284	0,611	59,614	61,762
MYBL1	0,001	0,031	0,17	0,069	0,085	0,306	1996,274	154,748
THAP11	0,003	0,031	0,17	0,457	0,312	-0,551	144,373	53,96
PLD2	0,002	0,031	0,17	0,234	0,171	-0,45	367,923	73,904
TMED1	0,003	0,031	0,17	0,927	1,422	0,618	56,807	63,711
PDXK	0,001	0,031	0,17	0,194	0,154	-0,334	806,72	140,222
SLC7A2	0,002	0,032	0,171	0,221	0,31	0,486	245,934	64,931
PLBD1	0,003	0,032	0,171	0,288	0,413	0,519	174,592	59,957
FUT8	0,002	0,032	0,171	0,185	0,238	0,365	546,025	115,428
PUSL1	0,003	0,032	0,173	0,386	0,265	-0,542	167,877	53,018
HSPB11	0,001	0,032	0,173	0,664	0,832	0,326	270,219	201,137
ABRACL	0,001	0,032	0,173	0,979	0,74	-0,404	158,065	136,428
AMBRA1	0,003	0,032	0,173	0,129	0,182	0,493	380,784	58,147
USP16	0,003	0,032	0,173	0,081	0,115	0,499	549,843	54,915
ITSN1	0,002	0,032	0,173	0,148	0,109	-0,448	549,269	69,646
CCDC134	0,002	0,032	0,174	0,587	0,827	0,494	115,423	80,33
PRKACA	0,001	0,033	0,175	0,172	0,208	0,272	1220,193	227,484
PLRG1	0,001	0,033	0,176	0,254	0,3	0,242	1065,908	294,644
IFNGR1	0,002	0,033	0,176	0,435	0,568	0,386	239,949	121,045
KLHL7	0,003	0,033	0,176	0,368	0,253	-0,542	172,278	51,851
STEAP1	0,004	0,033	0,176	0,504	0,861	0,772	59,102	35,313
TMEM66	0,001	0,033	0,176	0,302	0,243	-0,313	608,857	163,779
SCYL2	0,003	0,033	0,176	0,136	0,096	-0,5	473,608	53,377
EIF4G3	0,002	0,033	0,177	0,05	0,067	0,403	1372,512	80,014
MARCH4	0,003	0,033	0,177	0,042	0,059	0,502	1038,955	50,971
LPCAT1	0,001	0,033	0,177	0,143	0,117	-0,297	1317,342	172,772
TIAL1	0,001	0,034	0,177	0,254	0,297	0,224	1271,034	351,852
DAD1	0,001	0,033	0,177	0,965	0,817	-0,24	487,484	435,022
CALB2	0,001	0,033	0,177	0,288	0,371	0,365	385,522	124,049

MGAT5B	0,002	0,033	0,177	0,147	0,199	0,438	459,297	76,376
POFUT1	0,001	0,034	0,177	0,134	0,11	-0,276	1707,218	209,286
DESI1	0,001	0,033	0,177	0,113	0,141	0,318	1148,533	144,468
CRYZ	0,001	0,034	0,178	0,423	0,331	-0,353	371,684	142,05
GBP3	0,003	0,034	0,178	0,185	0,132	-0,494	364,449	57,295
RASSF1	0,002	0,034	0,178	0,473	0,654	0,468	149,166	82,316
PLSCR1	0,002	0,034	0,178	0,322	0,431	0,42	247,907	92,475
BRD8	0,001	0,034	0,178	0,118	0,147	0,316	1106,992	145,333
FOXF2	0,003	0,034	0,178	0,714	0,463	-0,625	82,44	49,795
CAV1	0,001	0,034	0,178	0,334	0,305	-0,128	6503,332	2059,36
PCM1	0,002	0,034	0,178	0,066	0,091	0,467	758,12	60,321
DOLK	0,002	0,034	0,178	0,466	0,623	0,418	191,531	106,273
NAP1L4	0,001	0,034	0,178	0,196	0,232	0,243	1350,99	289,351
PLEK2	0,003	0,034	0,178	0,15	0,219	0,546	260,722	46,788
NUDT21	0,001	0,034	0,178	0,215	0,188	-0,2	2395,097	476,373
AMMECR1	0,003	0,034	0,178	0,123	0,088	-0,487	546,807	57,451
CTSL	0,002	0,034	0,179	0,32	0,425	0,41	268,049	97,439
EHBP1	0,002	0,034	0,18	0,082	0,062	-0,413	1069,835	76,517
MCRS1	0,002	0,034	0,18	0,223	0,172	-0,374	537,666	106,505
GTF2F2	0,001	0,035	0,18	0,296	0,354	0,255	827,575	268,026
MT2A	0,001	0,035	0,18	0,575	0,638	0,149	2384,903	1443,639
GSK3A	0,002	0,034	0,18	0,15	0,196	0,39	545,955	93,225
PDIA6	0,001	0,035	0,182	0,488	0,557	0,191	1251,664	650,337
CCT7	0,001	0,035	0,182	0,414	0,452	0,126	8887,602	3836,414
WRNIP1	0,001	0,035	0,182	0,169	0,136	-0,316	957,486	145,111
NRG1	0,001	0,035	0,182	0,168	0,206	0,29	1029,722	197,65
CCNY	0,003	0,035	0,182	0,071	0,05	-0,492	867,53	52,33
MCMBP	0,001	0,035	0,182	0,271	0,237	-0,193	1961,505	494,375
HERPUD1	0,001	0,035	0,182	0,351	0,452	0,363	324,901	134,488
SCRN2	0,003	0,035	0,182	0,295	0,196	-0,59	171,183	41,327
ECSIT	0,001	0,035	0,182	0,344	0,276	-0,318	518,998	159,916
STK40	0,003	0,036	0,183	0,035	0,026	-0,461	1973,803	59,998

TPP1	0,001	0,036	0,183	0,212	0,254	0,259	1165,979	263,541
NAV2	0,002	0,035	0,183	0,068	0,05	-0,424	1331,292	81,515
XAB2	0,001	0,036	0,183	0,176	0,143	-0,292	1123,563	179,483
ARHGAP10	0,004	0,036	0,184	0,176	0,116	-0,597	259,152	37,62
COX7C	0,001	0,036	0,184	1,989	1,645	-0,274	286,456	497,008
PSMG4	0,003	0,036	0,184	0,506	0,336	-0,589	112,031	47,182
NTMT1	0,001	0,036	0,184	0,41	0,503	0,296	455,894	205,632
TMX1	0,001	0,036	0,184	0,508	0,44	-0,209	988,389	464,339
TMEM219	0,003	0,036	0,184	0,278	0,187	-0,574	188,131	42,769
LZIC	0,003	0,036	0,186	0,342	0,239	-0,519	198,27	54,625
ACVR1	0,002	0,036	0,186	0,204	0,285	0,481	257,659	61,927
TPRA1	0,002	0,037	0,186	0,461	0,336	-0,457	193,894	76,603
TRIM24	0,002	0,037	0,186	0,114	0,153	0,424	560,868	74,261
NEK7	0,002	0,037	0,187	0,164	0,126	-0,384	640,985	92,625
EIF2B5	0,001	0,037	0,188	0,249	0,2	-0,318	666,535	149,144
BUB3	0,001	0,037	0,188	0,359	0,321	-0,161	3017,827	1026,645
WDR1	0,001	0,037	0,19	0,196	0,177	-0,15	5634,201	1049,28
TRPC4AP	0,001	0,037	0,19	0,34	0,291	-0,222	1197,712	378,278
NOC2L	0,001	0,038	0,191	0,157	0,127	-0,302	1088,599	155,264
RND3	0,001	0,038	0,191	0,223	0,185	-0,268	1050,697	214,391
HNRNPK	0,001	0,038	0,191	0,162	0,185	0,196	2538,514	442,374
WNK1	0,001	0,038	0,191	0,129	0,146	0,179	4155,078	567,125
KIAA1033	0,001	0,038	0,191	0,183	0,15	-0,284	1093,038	181,326
COX4I1	0,001	0,038	0,191	0,712	0,808	0,182	1110,682	837,32
BABAM1	0,003	0,038	0,191	0,208	0,143	-0,536	267,691	46,35
LAMTOR5	0,001	0,038	0,192	0,7	0,569	-0,3	334,945	211,641
PRKAR2A	0,002	0,038	0,192	0,065	0,082	0,328	1668,361	123,312
CCNH	0,001	0,038	0,192	0,296	0,358	0,274	688,775	221,311
VPS13A	0,002	0,038	0,192	0,075	0,095	0,341	1341,152	112,981
TEAD1	0,001	0,038	0,192	0,054	0,045	-0,267	3939,439	195,691
KANK2	0,001	0,038	0,192	0,112	0,091	-0,288	1678,306	171,073
STX10	0,002	0,038	0,192	0,154	0,209	0,444	379,35	68,653

HCFC1	0,003	0,038	0,192	0,117	0,163	0,485	396,248	55,33
ANXA3	0,001	0,039	0,194	0,293	0,343	0,227	1061,423	334,321
RIPK1	0,002	0,039	0,194	0,135	0,179	0,406	513,095	81,674
SYVN1	0,001	0,039	0,194	0,4	0,47	0,234	767,133	332,086
THAP4	0,002	0,039	0,195	0,146	0,113	-0,368	763,784	99,219
PRKDC	0,001	0,039	0,195	0,161	0,177	0,135	11793,552	1996,966
PRCP	0,001	0,039	0,195	0,427	0,498	0,223	819,079	382,325
ZNF609	0,003	0,039	0,195	0,057	0,04	-0,507	987,634	48,353
GIGYF2	0,002	0,039	0,196	0,073	0,096	0,402	937,232	78,308
MED24	0,001	0,039	0,196	0,243	0,202	-0,262	1004,571	223,757
TMEM175	0,002	0,04	0,197	0,359	0,496	0,468	170,958	71,482
PROSER1	0,001	0,04	0,197	0,113	0,092	-0,306	1391,485	143,857
FTSJ2	0,003	0,04	0,198	0,187	0,134	-0,484	351,122	55,825
MVD	0,002	0,04	0,198	0,3	0,219	-0,45	274,219	71,614
YWHAE	0,001	0,04	0,198	0,268	0,244	-0,137	7237,675	1842,867
WDR82	0,001	0,04	0,199	0,18	0,151	-0,256	1368,254	225,401
WHSC1	0,001	0,04	0,199	0,036	0,029	-0,293	4508,576	145,122
SIX4	0,002	0,04	0,199	0,231	0,171	-0,43	368,234	73,75
EXO1	0,002	0,04	0,2	0,111	0,148	0,409	593,55	76,859
PDGFC	0,001	0,041	0,2	0,188	0,228	0,282	870,88	181,37
SIVA1	0,001	0,041	0,2	0,519	0,425	-0,29	430,535	202,358
STAP2	0,002	0,041	0,2	0,371	0,266	-0,48	199,962	63,723
STK4	0,001	0,041	0,2	0,075	0,093	0,313	1564,167	131,524
PSEN2	0,003	0,041	0,201	0,334	0,231	-0,532	179,346	48,69
TATDN1	0,002	0,041	0,201	0,413	0,583	0,5	129,727	66,287
NANS	0,001	0,041	0,201	0,355	0,43	0,277	554,219	215,069
TMED10	0,001	0,041	0,201	0,338	0,298	-0,181	1889,643	596,157
NDEL1	0,002	0,041	0,201	0,092	0,118	0,354	962,96	100,463
UBXN6	0,002	0,041	0,201	0,142	0,106	-0,42	586,462	72,83
IFNAR1	0,003	0,041	0,201	0,073	0,101	0,466	648,158	56,768
PLP2	0,001	0,041	0,201	1,405	1,105	-0,347	160,398	201,724
DUSP7	0,002	0,041	0,202	0,109	0,081	-0,428	729,154	70,249

NGDN	0,002	0,041	0,202	0,424	0,548	0,37	244,083	119,031
IP6K1	0,001	0,042	0,203	0,149	0,118	-0,328	931,827	125,021
SREK1	0,002	0,042	0,203	0,074	0,096	0,374	1048,544	90,026
CALM3	0,001	0,042	0,203	0,16	0,181	0,182	3204,123	543,185
LARP1	0,001	0,042	0,204	0,033	0,039	0,253	5766,734	208,11
ATE1	0,003	0,042	0,204	0,134	0,098	-0,45	536,105	61,193
TMEM194A	0,001	0,042	0,204	0,179	0,143	-0,327	774,794	123,501
KIAA0586	0,002	0,042	0,204	0,092	0,123	0,416	660,613	71,099
DHRS7B	0,001	0,042	0,204	0,708	0,572	-0,308	300,511	191,989
TTLL12	0,001	0,042	0,204	0,249	0,212	-0,234	1282,662	296,896
FAM127A	0,002	0,042	0,204	0,321	0,229	-0,487	237,274	68,014
MUTYH	0,005	0,043	0,205	0,205	0,133	-0,623	205,95	35,742
ZNHIT6	0,001	0,043	0,205	0,184	0,226	0,302	738,141	151,576
WDR48	0,002	0,043	0,205	0,13	0,174	0,416	491,879	76,265
AFAP1	0,002	0,042	0,205	0,046	0,034	-0,437	1548,733	59,981
LATS1	0,003	0,042	0,205	0,086	0,117	0,453	583,144	59,247
ATL3	0,001	0,042	0,205	0,277	0,24	-0,206	1524,648	392,263
SRP54	0,001	0,043	0,205	0,206	0,258	0,321	581,181	135,611
GSPT1	0,001	0,043	0,205	0,095	0,113	0,255	2042,077	211,088
SMCHD1	0,001	0,042	0,205	0,115	0,136	0,236	2076,226	260,894
PES1	0,001	0,043	0,205	0,161	0,192	0,259	1257,904	220,357
STIL	0,001	0,043	0,206	0,097	0,117	0,273	1686,718	181,007
LIN54	0,002	0,043	0,206	0,186	0,247	0,409	372,167	79,878
LMBRD1	0,001	0,043	0,206	0,675	0,863	0,353	195,485	152,183
PHF14	0,002	0,043	0,206	0,102	0,139	0,449	508,002	60,52
YKT6	0,002	0,043	0,206	0,088	0,111	0,334	1114,425	111,886
MRPL49	0,001	0,043	0,206	0,238	0,196	-0,275	892,079	193,714
KCTD14	0,002	0,043	0,206	0,543	0,778	0,519	101,452	64,312
ZC3H14	0,001	0,043	0,206	0,168	0,198	0,24	1454,867	266,278
RPL18A	0,003	0,043	0,206	0,983	0,677	-0,539	78,018	63,526
MLLT4	0,002	0,043	0,207	0,049	0,064	0,386	1420,332	79,038
ERH	0,001	0,043	0,207	0,666	0,586	-0,187	979,338	609,227

RPS27A	0,002	0,044	0,208	1,455	1,062	-0,455	87,261	108,262
EIF4A2	0,001	0,044	0,208	0,192	0,164	-0,227	1589,804	282,883
TMEM171	0,003	0,044	0,208	0,504	0,751	0,576	84,797	50,624
NDUFB9	0,001	0,044	0,208	0,33	0,395	0,259	648,968	236,302
SMPD1	0,002	0,044	0,208	0,347	0,466	0,424	225,801	87,773
BCKDK	0,002	0,044	0,208	0,284	0,222	-0,358	432,447	109,394
POLR2F	0,001	0,044	0,208	0,625	0,507	-0,301	339,279	192,112
DDIT4	0,002	0,044	0,209	0,717	0,551	-0,379	190,466	118,444
SMURF2	0,002	0,044	0,209	0,14	0,106	-0,398	653,661	81,938
P4HA1	0,001	0,045	0,21	0,283	0,343	0,278	608,918	191,317
PARPBP	0,002	0,045	0,21	0,338	0,251	-0,431	255,585	73,294
CAPN15	0,002	0,044	0,21	0,109	0,08	-0,449	648,924	62,235
NUDT16L1	0,002	0,044	0,21	0,817	0,589	-0,473	111,295	77,304
APBA3	0,002	0,044	0,21	0,355	0,479	0,432	196,614	80,242
TOMM40	0,001	0,044	0,21	0,325	0,386	0,249	713,169	253,582
PNKP	0,003	0,044	0,21	0,169	0,116	-0,542	297,202	42,368
PTP4A1	0,001	0,045	0,212	0,167	0,143	-0,23	1796,567	276,226
PSMD13	0,001	0,045	0,212	0,435	0,491	0,174	1435,401	664,373
CCND1	0,001	0,045	0,212	0,179	0,159	-0,165	3967,112	664,738
CCDC59	0,003	0,045	0,212	0,154	0,214	0,477	300,358	54,982
KIAA0368	0,001	0,045	0,213	0,127	0,147	0,209	2359,708	322,932
UFM1	0,001	0,045	0,213	0,239	0,292	0,289	645,361	171,385
TRADD	0,003	0,045	0,213	0,596	0,402	-0,569	96,997	48,885
PPAP2B	0,003	0,046	0,214	0,222	0,317	0,513	211,751	53,665
ZSWIM6	0,002	0,046	0,214	0,198	0,25	0,342	521,933	115,523
LRP6	0,002	0,046	0,214	0,083	0,108	0,377	887,589	84,109
C17orf70	0,003	0,046	0,214	0,105	0,072	-0,538	461,134	40,912
GRWD1	0,001	0,046	0,214	0,216	0,258	0,257	920,587	217,862
RPL18	0,001	0,046	0,214	0,789	0,861	0,127	2586,153	2134,116
RLF	0,002	0,046	0,215	0,089	0,114	0,361	907,931	93,161
TFG	0,001	0,046	0,215	0,299	0,348	0,217	1059,711	342,125
LOX	0,003	0,046	0,215	0,243	0,338	0,477	205,666	58,927

UBR1	0,002	0,046	0,215	0,171	0,13	-0,395	532,357	79,485
TUBGCP6	0,002	0,046	0,215	0,069	0,052	-0,415	1116,773	67,514
SERBP1	0,001	0,047	0,216	0,081	0,094	0,221	3159,239	275,219
ANP32B	0,001	0,047	0,216	0,172	0,196	0,185	2873,788	522,955
TIMM10	0,001	0,047	0,216	0,867	1,047	0,271	297,313	282,923
KIAA0101	0,001	0,047	0,216	0,899	1,048	0,221	483,156	469,473
GNA11	0,002	0,047	0,216	0,11	0,088	-0,314	1293,747	127,908
NSFL1C	0,001	0,046	0,216	0,236	0,289	0,292	621,951	164,654
SMTN	0,001	0,047	0,216	0,107	0,126	0,233	2341,352	267,895
DAZAP1	0,001	0,047	0,217	0,207	0,237	0,194	2057,672	452,052
SMYD5	0,001	0,047	0,218	0,2	0,168	-0,252	1247,059	230,346
COX5B	0,001	0,047	0,218	1,763	2,035	0,207	414,479	781,874
CTTN	0,001	0,047	0,218	0,157	0,136	-0,202	2433,369	356,852
AJUBA	0,001	0,047	0,218	0,148	0,126	-0,231	1862,707	255,184
BCL2L1	0,001	0,047	0,218	0,082	0,07	-0,236	3131,183	239,253
LSS	0,002	0,047	0,218	0,155	0,121	-0,364	684,7	95,304
SSB	0,001	0,048	0,219	0,17	0,206	0,282	886,58	166,527
CAB39	0,001	0,048	0,219	0,15	0,181	0,272	1053,554	174,863
SCFD2	0,003	0,048	0,219	0,275	0,191	-0,529	196,837	45,871
NDUFA2	0,001	0,048	0,219	2,251	1,72	-0,388	95,353	186,821
DPP8	0,002	0,048	0,219	0,134	0,099	-0,439	528,106	60,072
PLK1	0,001	0,048	0,219	0,214	0,241	0,17	2792,857	632,573
STARD3	0,003	0,048	0,219	0,137	0,187	0,456	376,859	59,276
WBP2	0,001	0,048	0,219	0,252	0,293	0,219	1233,498	331,701
CEP89	0,003	0,048	0,219	0,164	0,23	0,49	260,524	51,465
RALGAPB	0,002	0,048	0,219	0,088	0,071	-0,32	1492,247	117,987
MTG2	0,002	0,048	0,219	0,204	0,156	-0,391	458,684	82,434
EIF2S3	0,001	0,048	0,219	0,273	0,223	-0,29	654,039	159,736
PYCR2	0,002	0,049	0,22	0,184	0,229	0,322	639,477	129,247
MRPL2	0,001	0,049	0,22	0,546	0,657	0,267	410,512	244,265
C8orf33	0,001	0,048	0,22	0,204	0,254	0,316	592,311	138,487
DUS3L	0,002	0,048	0,22	0,247	0,311	0,333	444,114	122,654

TMEM243	0,003	0,049	0,221	0,339	0,231	-0,55	152,493	42,388
KCTD10	0,001	0,049	0,221	0,182	0,146	-0,318	756,762	123,303
STK17B	0,002	0,049	0,222	0,218	0,168	-0,378	452,34	86,23
BCKDHB	0,004	0,049	0,222	0,433	0,288	-0,587	111,592	40,177
ADAM8	0,004	0,049	0,222	0,295	0,198	-0,574	158,656	39,473
ACBD6	0,002	0,049	0,223	0,318	0,411	0,37	284,051	102,399
FKBP9	0,003	0,05	0,223	0,281	0,201	-0,48	230,465	55,795
ECD	0,001	0,049	0,223	0,16	0,199	0,313	714,372	127,839
RNF138	0,003	0,049	0,223	0,071	0,099	0,481	566,608	48,259
TNRC6B	0,003	0,05	0,223	0,074	0,1	0,439	672,315	58,473
ACBD3	0,002	0,05	0,224	0,294	0,233	-0,337	442,792	116,145
MTHFD2	0,001	0,05	0,225	0,306	0,266	-0,203	1359,286	382,062
GPRIN1	0,002	0,05	0,225	0,216	0,274	0,346	445,519	108,556
XPOT	0,001	0,05	0,225	0,114	0,135	0,245	1696,528	211,758
CLASP2	0,002	0,051	0,226	0,1	0,077	-0,376	914,004	79,778
HMGCR	0,001	0,051	0,226	0,303	0,358	0,241	764,202	252,396
INTS1	0,002	0,05	0,226	0,082	0,064	-0,359	1224,814	90,805
TRA2A	0,001	0,051	0,226	0,351	0,411	0,228	781,23	297,982
TNFRSF10A	0,002	0,051	0,226	0,284	0,361	0,343	360,866	115,425
ZHX1	0,002	0,051	0,226	0,084	0,113	0,43	621,076	60,461
DUSP5	0,002	0,051	0,226	0,205	0,152	-0,429	368,274	64,281
TMEM9B	0,002	0,051	0,226	0,334	0,253	-0,402	278,756	81,784
TMEM41B	0,001	0,05	0,226	0,329	0,412	0,327	360,093	132,592
ACTR6	0,003	0,05	0,226	0,502	0,342	-0,553	109,47	44,938
TPCN1	0,002	0,051	0,226	0,159	0,122	-0,382	592,105	83,648
PPP1R13L	0,001	0,051	0,226	0,189	0,231	0,293	772,742	158,099
EFHD2	0,001	0,051	0,227	0,23	0,197	-0,225	1368,278	293,974
ANXA1	0,001	0,051	0,227	0,358	0,39	0,124	4549,285	1705,276
NAV3	0,003	0,051	0,227	0,055	0,076	0,472	805,704	50,501
CARD10	0,002	0,051	0,227	0,098	0,077	-0,349	1082,006	95,435
CERK	0,002	0,051	0,227	0,064	0,049	-0,372	1449,949	81,923
THOC2	0,001	0,051	0,227	0,069	0,084	0,271	2160,66	165,385

CDKN2C	0,002	0,052	0,228	0,158	0,205	0,38	477,664	84,786
SMC6	0,003	0,052	0,228	0,055	0,076	0,455	804,419	52,633
POLA2	0,002	0,052	0,228	0,162	0,213	0,399	400,176	75,32
TFAP4	0,002	0,051	0,228	0,348	0,467	0,424	197,026	78,153
LDLR	0,001	0,051	0,228	0,239	0,268	0,164	2749,157	690,028
PPDPF	0,001	0,051	0,228	0,481	0,59	0,295	343,265	182,225
TLK1	0,003	0,052	0,229	0,072	0,098	0,435	682,532	58,097
MRRF	0,002	0,052	0,229	0,234	0,312	0,417	268,23	72,735
TAGLN2	0,001	0,052	0,23	0,326	0,36	0,142	2969,461	1015,904
TUBGCP2	0,001	0,053	0,23	0,194	0,228	0,235	1204,366	253,668
C12orf75	0,003	0,052	0,23	0,094	0,069	-0,451	676,924	55,569
NXN	0,002	0,052	0,23	0,239	0,19	-0,332	536,78	114,815
PHB	0,001	0,052	0,23	0,652	0,766	0,232	483,501	340,38
GLTSCR2	0,002	0,052	0,23	0,17	0,135	-0,339	689,462	104,461
IL11	0,001	0,052	0,23	0,168	0,208	0,307	739,971	135,659
SNRPB2	0,001	0,052	0,23	0,486	0,402	-0,274	461,304	202,204
BTD	0,001	0,053	0,231	0,746	0,593	-0,331	230,943	150,398
GTPBP4	0,001	0,053	0,232	0,123	0,144	0,234	1735,146	231,558
PEBP1	0,001	0,053	0,232	0,432	0,38	-0,183	1288,422	517,538
SSR4	0,001	0,053	0,232	1,742	1,308	-0,413	89,294	135,227
HIPK1	0,001	0,054	0,233	0,149	0,124	-0,267	1301,097	175,182
CMTM8	0,004	0,054	0,233	1,306	0,818	-0,675	39,479	42,093
HMCES	0,001	0,054	0,233	0,347	0,28	-0,306	458,37	143,012
MFSD1	0,001	0,054	0,233	0,442	0,352	-0,325	337,611	133,879
PPA2	0,001	0,054	0,233	1,148	0,927	-0,309	200,268	204,694
NDUFC1	0,001	0,054	0,233	2,288	1,741	-0,395	87,195	176,084
MTMR12	0,002	0,053	0,233	0,061	0,081	0,413	902,603	63,769
MRPS18A	0,002	0,053	0,233	0,63	0,856	0,441	114,507	84,026
PON2	0,003	0,054	0,233	0,191	0,271	0,502	209,202	47,367
TLN1	0,001	0,054	0,233	0,049	0,056	0,182	8756,9	455,912
DENND5A	0,004	0,053	0,233	0,065	0,045	-0,539	699,015	39,15
ZDHHC7	0,002	0,053	0,233	0,08	0,107	0,415	690,93	64,019

APRT	0,001	0,054	0,233	0,968	0,828	-0,224	447,951	397,602
TAF15	0,003	0,054	0,233	0,123	0,168	0,453	379,826	55,612
DUSP3	0,001	0,053	0,233	0,175	0,143	-0,295	879,442	140,177
TAF4	0,002	0,054	0,234	0,223	0,285	0,355	394,177	99,536
AKR7A2	0,001	0,054	0,235	0,55	0,452	-0,283	380,555	191,205
TGFBR3	0,003	0,055	0,235	0,126	0,176	0,484	317,09	48,799
TANC1	0,002	0,055	0,235	0,093	0,125	0,421	571,827	61,281
PSMD6	0,001	0,055	0,235	0,397	0,453	0,189	1080,136	459,741
ELOVL6	0,001	0,055	0,235	0,191	0,234	0,293	683,694	145,707
P4HA2	0,001	0,055	0,235	0,442	0,56	0,34	267,441	130,384
TWISTNB	0,002	0,054	0,235	0,17	0,134	-0,34	663,571	100,487
H2AFV	0,001	0,055	0,235	0,246	0,286	0,217	1156,346	308,35
WAC	0,001	0,055	0,235	0,097	0,114	0,228	2343,585	246,207
RELA	0,001	0,055	0,235	0,216	0,251	0,217	1335,232	308,932
CTSC	0,001	0,054	0,235	0,656	0,736	0,166	1302,78	895,251
AEBP2	0,001	0,055	0,235	0,254	0,31	0,29	552,824	157,419
TLE3	0,002	0,055	0,235	0,132	0,171	0,377	532,661	82,149
CDC25B	0,001	0,055	0,235	0,19	0,163	-0,221	1517,708	267,165
EDEM2	0,003	0,055	0,235	0,301	0,213	-0,501	190,961	49,38
THUMPD3	0,002	0,055	0,236	0,183	0,238	0,382	386,698	81,794
OASL	0,004	0,055	0,236	0,375	0,254	-0,56	128,713	40,622
ATXN2L	0,001	0,055	0,236	0,118	0,135	0,202	2643,635	332,035
GNA13	0,002	0,055	0,236	0,126	0,102	-0,306	1082,478	122,366
POLR2D	0,002	0,056	0,237	0,215	0,159	-0,439	330,008	59,441
ERRFI1	0,001	0,056	0,238	0,185	0,158	-0,228	1464,871	249,996
ACAD9	0,002	0,056	0,238	0,224	0,176	-0,354	486,046	97,301
MRPL17	0,001	0,056	0,238	0,666	0,579	-0,201	722,999	451,635
EIF3J	0,001	0,056	0,238	0,166	0,202	0,287	798,98	146,47
SLTM	0,001	0,056	0,238	0,072	0,089	0,299	1522,049	122,942
RBBP6	0,002	0,056	0,238	0,053	0,07	0,413	1001,775	61,258
ZNF217	0,001	0,056	0,238	0,198	0,235	0,244	1021,18	222,001
AHCYL1	0,001	0,056	0,239	0,139	0,161	0,215	1914,449	288,266

TGFB2	0,001	0,057	0,239	0,184	0,215	0,224	1305,416	260,225
ANKRD50	0,002	0,057	0,239	0,097	0,125	0,361	769,277	85,375
PREP	0,002	0,057	0,239	0,214	0,168	-0,347	524,886	100,441
DLD	0,001	0,057	0,239	0,329	0,283	-0,217	986,216	299,098
CENPB	0,002	0,056	0,239	0,105	0,133	0,339	830,02	99,278
GPC1	0,002	0,057	0,24	0,263	0,33	0,329	405,838	118,199
COL1A1	0,003	0,057	0,24	0,257	0,186	-0,465	260,868	59,363
PCBP4	0,003	0,057	0,241	0,108	0,146	0,445	455,961	56,023
HAUS5	0,002	0,057	0,241	0,239	0,184	-0,381	383,652	79,724
NDUFB4	0,001	0,058	0,242	1,246	1,494	0,262	241,559	329,241
PRKAA1	0,001	0,058	0,242	0,164	0,191	0,226	1416,831	252,35
LMAN2	0,001	0,058	0,242	0,452	0,4	-0,176	1284,061	547,833
CDKN1B	0,002	0,058	0,242	0,182	0,239	0,393	358,732	76,59
ARPP19	0,001	0,058	0,242	0,12	0,097	-0,3	1140,383	121,721
LUC7L3	0,001	0,058	0,242	0,084	0,103	0,295	1370,319	128,472
CDCA4	0,001	0,059	0,245	0,583	0,508	-0,198	791,358	429,924
EVI2A	0,003	0,059	0,245	0,237	0,326	0,459	202,448	57,91
PGRMC1	0,001	0,059	0,245	0,394	0,349	-0,172	1558,743	580,453
CAPZB	0,001	0,059	0,246	0,297	0,337	0,183	1488,444	468,837
DIS3	0,001	0,059	0,246	0,091	0,111	0,277	1429,002	144,746
HTATSF1	0,001	0,059	0,246	0,134	0,165	0,302	830,694	124,327
MRPL28	0,001	0,059	0,247	0,371	0,435	0,228	715,688	285,355
MRPS7	0,001	0,059	0,247	0,639	0,556	-0,201	706,422	421,596
NIF3L1	0,002	0,06	0,248	0,253	0,322	0,349	348,136	100,087
RNF145	0,001	0,06	0,248	0,281	0,238	-0,238	892,94	231,412
TM2D2	0,001	0,06	0,248	0,506	0,405	-0,32	296,568	133,683
ALDH1B1	0,001	0,06	0,248	0,347	0,292	-0,249	692,963	222,424
ANAPC5	0,001	0,06	0,248	0,289	0,235	-0,296	540,312	140,633
TP53I13	0,003	0,059	0,248	0,191	0,262	0,455	253,516	55,85
SVIL	0,004	0,06	0,249	0,042	0,028	-0,545	971,299	34,662
PRPF39	0,003	0,06	0,249	0,131	0,181	0,471	312,434	48,995
EIF3E	0,001	0,06	0,25	0,449	0,497	0,149	1936,299	919,933

WDR75	0,001	0,061	0,252	0,138	0,167	0,276	993,135	151,196
RFWD3	0,001	0,061	0,252	0,221	0,25	0,179	1821,504	430,016
FXR2	0,001	0,061	0,252	0,081	0,096	0,245	2145,264	189,649
TFB2M	0,002	0,061	0,253	0,448	0,331	-0,436	172,033	66,528
TIMELESS	0,002	0,061	0,253	0,13	0,103	-0,334	830,845	95,941
GOSR1	0,002	0,061	0,253	0,225	0,168	-0,422	325,282	63,937
PDLIM7	0,001	0,062	0,254	0,284	0,325	0,193	1305,925	395,724
UPF1	0,001	0,061	0,254	0,097	0,114	0,237	1958,745	206,942
UBP1	0,002	0,062	0,255	0,077	0,061	-0,34	1319,251	90,421
SNX19	0,002	0,062	0,255	0,101	0,081	-0,319	1154,518	104,944
CEBPZ	0,003	0,063	0,257	0,07	0,095	0,448	598,658	49,582
SRI	0,001	0,062	0,257	0,622	0,532	-0,225	545,552	314,286
IGFBP6	0,001	0,063	0,258	1,011	0,814	-0,313	203,376	188,93
MTMR3	0,004	0,063	0,258	0,1	0,07	-0,518	446,504	37,989
WDR3	0,001	0,063	0,259	0,087	0,106	0,271	1520,437	146,053
SUPT6H	0,002	0,063	0,259	0,026	0,034	0,381	2199,479	66,409
SRP68	0,001	0,063	0,259	0,188	0,163	-0,205	1758,451	308,011
FEM1C	0,003	0,064	0,26	0,112	0,153	0,449	387,349	51,132
AP1M2	0,001	0,064	0,26	0,36	0,299	-0,267	549,187	181,4
RPRD2	0,002	0,064	0,262	0,074	0,095	0,345	1013,664	85,887
ERGIC1	0,001	0,064	0,263	0,122	0,103	-0,247	1650,139	186,497
BTN2A1	0,003	0,064	0,263	0,124	0,17	0,45	352,268	51,43
FADD	0,002	0,064	0,263	0,919	0,715	-0,362	149,761	122,852
STX12	0,004	0,065	0,264	0,282	0,195	-0,531	165,834	39,526
CHST3	0,003	0,065	0,264	0,06	0,081	0,425	764,304	53,967
STRAP	0,001	0,065	0,264	0,238	0,268	0,169	2057,715	520,214
DYNC1H1	0,001	0,065	0,264	0,046	0,05	0,143	16446,349	788,862
ETFB	0,002	0,065	0,264	0,83	0,647	-0,359	162,014	120,541
NDUFS5	0,001	0,066	0,266	1,11	1,246	0,167	741,297	867,571
PTDSS2	0,001	0,065	0,266	0,34	0,276	-0,297	448,499	138,096
MIER2	0,002	0,065	0,266	0,194	0,253	0,377	363,823	79,195
ENSG00000225091.3	0,004	0,066	0,266	6,652	3,195	-1,058	8,296	39,558

CTBS	0,002	0,066	0,267	0,213	0,271	0,347	387,901	93,411
KATNA1	0,003	0,066	0,267	0,23	0,322	0,488	170,855	47,938
FZD6	0,001	0,066	0,267	0,329	0,396	0,266	495,458	179,623
ADM	0,001	0,066	0,267	0,45	0,399	-0,174	1276,96	536,4
CHEK1	0,001	0,066	0,267	0,219	0,262	0,261	724,365	175,076
UBL7	0,001	0,066	0,267	0,445	0,362	-0,297	366,741	148,56
TCF25	0,002	0,066	0,267	0,137	0,105	-0,385	586,341	71,684
ITGB1BP1	0,002	0,067	0,269	0,164	0,125	-0,386	483,578	69,583
FAM208A	0,001	0,067	0,269	0,124	0,149	0,265	1133,865	155,395
NAA15	0,001	0,067	0,269	0,129	0,151	0,225	1638,535	231,126
VDAC1	0,001	0,067	0,269	0,17	0,2	0,236	1163,819	213,256
KRT8	0,001	0,067	0,269	0,244	0,285	0,222	990,44	260,442
ST7L	0,002	0,067	0,27	0,546	0,406	-0,427	148,619	70,639
CAMSAP2	0,002	0,067	0,27	0,075	0,094	0,332	1098,245	92,495
VPS35	0,001	0,067	0,27	0,123	0,104	-0,235	1739,87	195,085
KDM1B	0,003	0,068	0,271	0,147	0,105	-0,487	338,991	42,721
SEC31A	0,001	0,068	0,273	0,153	0,178	0,219	1483,596	244,519
SAAL1	0,001	0,068	0,273	0,465	0,378	-0,301	333,71	140,005
PDXDC1	0,002	0,068	0,273	0,16	0,126	-0,341	623,193	88,414
VAV2	0,002	0,068	0,274	0,094	0,119	0,349	780,153	82,186
NUDT5	0,001	0,068	0,274	0,408	0,478	0,229	584,76	259,418
ACOT9	0,001	0,068	0,274	0,271	0,33	0,281	499,349	149,735
S100A13	0,002	0,069	0,275	0,422	0,565	0,422	146,334	71,046
DYNC1I2	0,004	0,069	0,275	0,191	0,134	-0,505	246,781	39,794
MGAT1	0,001	0,069	0,275	0,181	0,152	-0,248	1130,488	188,741
SLC35E1	0,002	0,069	0,275	0,13	0,105	-0,314	904,911	104,909
TXNRD2	0,002	0,069	0,275	0,405	0,312	-0,374	239,476	86,167
DNMBP	0,001	0,069	0,276	0,065	0,077	0,257	2293,397	162,127
PPP6R1	0,001	0,069	0,276	0,108	0,126	0,226	1877,74	219,436
PBRM1	0,002	0,07	0,277	0,04	0,05	0,332	1904,25	85,778
CKB	0,001	0,07	0,277	0,566	0,47	-0,27	364,588	186,302
PSMA4	0,001	0,07	0,278	0,517	0,581	0,169	1072,911	591,271

ALG2	0,002	0,07	0,279	0,246	0,2		-0,301	560,688	125,66
TMEM127	0,002	0,071	0,28	0,143	0,115		-0,309	842,125	109,841
KRIT1	0,003	0,071	0,28	0,102	0,14		0,458	383,149	47,011
POLR2K	0,002	0,071	0,28	0,378	0,302		-0,323	333,747	112,131
FAM91A1	0,002	0,071	0,28	0,124	0,102		-0,29	1083,64	120,045
TECPR2	0,003	0,071	0,28	0,141	0,1		-0,495	332,103	39,883
SHISA5	0,001	0,071	0,281	0,135	0,113		-0,256	1313,625	164,92
RPS27L	0,001	0,071	0,281	2,41	1,932		-0,319	115,434	246,041
JUNB	0,002	0,071	0,281	0,254	0,337		0,406	228,579	67,151
GSTA4	0,003	0,071	0,282	0,392	0,286		-0,458	161,106	54,603
SCAF8	0,002	0,071	0,282	0,087	0,114		0,389	628,816	63,114
MCM6	0,001	0,072	0,283	0,27	0,234		-0,207	1135,374	281,912
LANCL1	0,002	0,072	0,283	0,251	0,196		-0,36	367,658	81,378
SEC22C	0,002	0,072	0,283	0,148	0,118		-0,324	722,501	94,94
MRPS22	0,001	0,072	0,283	0,623	0,527		-0,244	413,854	236,627
GPX8	0,001	0,072	0,283	0,263	0,311		0,241	724,858	207,571
SPDL1	0,001	0,072	0,283	0,173	0,198		0,193	1773,857	329,925
LRCH4	0,002	0,072	0,283	0,168	0,131		-0,353	548,534	82,383
MLF2	0,001	0,072	0,283	0,275	0,247		-0,156	2537,244	663,412
HIF1A	0,001	0,072	0,283	0,097	0,111		0,188	3264,004	337,701
GLYR1	0,002	0,072	0,283	0,095	0,124		0,376	650,128	69,559
AKAP1	0,001	0,072	0,283	0,169	0,198		0,227	1190,011	217,954
PRPS2	0,001	0,072	0,283	0,32	0,268		-0,256	613,549	180,732
NAA50	0,001	0,073	0,284	0,171	0,195		0,193	1746,173	320,197
QPCTL	0,003	0,073	0,284	0,37	0,261		-0,505	138,047	43,066
SCAND1	0,001	0,073	0,284	0,997	1,178		0,242	287,304	309,656
HMOX1	0,001	0,073	0,284	0,364	0,455		0,32	298,028	125,356
PBXIP1	0,003	0,073	0,285	0,394	0,547		0,474	115,109	55,544
AAK1	0,002	0,073	0,285	0,165	0,203		0,298	645,121	118,65
SEP8	0,002	0,073	0,285	0,147	0,118		-0,313	803,714	107,424
KLHDC3	0,002	0,073	0,285	0,161	0,131		-0,297	814,165	118,843
ZER1	0,003	0,073	0,285	0,161	0,119		-0,434	374,548	52,638

RPS13	0,001	0,074	0,286	2,978	2,683	-0,15	616,895	1743,756
TMEM14A	0,001	0,074	0,287	1,084	0,886	-0,292	196,948	193,831
PUF60	0,001	0,074	0,287	0,486	0,602	0,311	247,859	134,597
SMARCAD1	0,002	0,075	0,288	0,119	0,15	0,339	641,523	87,208
TMEM167A	0,003	0,075	0,288	0,105	0,078	-0,433	541,316	48,647
CHD1	0,002	0,075	0,288	0,045	0,057	0,345	1504,398	76,013
FKBP15	0,002	0,074	0,288	0,088	0,114	0,369	698,893	69,985
POLE3	0,001	0,075	0,288	0,089	0,107	0,264	1462,19	143,002
RAD51	0,002	0,075	0,288	0,257	0,339	0,399	228,159	68,207
SOGA2	0,002	0,075	0,288	0,046	0,037	-0,342	1897,659	78,15
CDC34	0,001	0,074	0,288	0,268	0,222	-0,271	619,686	151,764
IGF2BP2	0,002	0,075	0,289	0,096	0,075	-0,352	885,472	75,347
RAP1GDS1	0,001	0,075	0,289	0,273	0,327	0,264	546,764	163,71
PARP4	0,001	0,075	0,289	0,082	0,095	0,213	2691,361	237,905
FAM98B	0,002	0,075	0,289	0,24	0,299	0,316	402,751	109,27
EPS15	0,001	0,075	0,29	0,243	0,2	-0,277	628,333	138,07
SCP2	0,001	0,076	0,29	0,492	0,417	-0,239	499,307	226,711
METAP1	0,001	0,076	0,29	0,254	0,207	-0,292	543,342	124,704
KDM3B	0,001	0,076	0,29	0,116	0,096	-0,276	1197,805	126,339
NT5E	0,001	0,076	0,29	0,213	0,235	0,138	3611,194	815,074
ASNS	0,001	0,075	0,29	0,232	0,283	0,285	520,069	134,994
QSOX2	0,001	0,076	0,29	0,211	0,249	0,241	860,144	195,629
AGPAT2	0,002	0,075	0,29	0,249	0,2	-0,316	477,243	107,297
UBE4A	0,001	0,076	0,29	0,276	0,336	0,284	459,322	141,171
DDX55	0,002	0,075	0,29	0,136	0,179	0,4	388,709	60,678
SAP18	0,001	0,075	0,29	0,651	0,578	-0,171	937,193	576,294
TAOK2	0,003	0,076	0,29	0,057	0,076	0,416	786,343	51,595
PTTG1IP	0,001	0,076	0,29	0,163	0,147	-0,156	3528,312	542,721
GAS2L1	0,002	0,076	0,29	0,09	0,069	-0,371	846,276	67,353
APEX2	0,001	0,076	0,29	0,206	0,242	0,233	937,226	209,192
PSME4	0,001	0,077	0,291	0,141	0,166	0,235	1215,794	187,523
CYBRD1	0,001	0,077	0,291	0,291	0,24	-0,277	547,695	145,35

ATP5G3	0,001	0,076	0,291	1,416	1,284	-0,141	1010,139	1365,448
APBB2	0,002	0,076	0,291	0,13	0,099	-0,384	561,221	63,906
IL6ST	0,001	0,077	0,291	0,09	0,108	0,273	1300,148	129,17
SLC25A46	0,001	0,077	0,291	0,205	0,168	-0,286	671,514	124,369
MATR3	0,001	0,077	0,291	0,096	0,11	0,194	2947,935	305,609
CD2AP	0,002	0,077	0,291	0,085	0,107	0,331	890,839	86,434
TAX1BP1	0,001	0,077	0,291	0,171	0,199	0,217	1237,834	229,307
DUSP4	0,001	0,077	0,291	0,137	0,16	0,223	1493,233	222,532
LAPTM4B	0,001	0,076	0,291	0,758	0,67	-0,177	773,124	547,816
TNFRSF1A	0,001	0,076	0,291	0,245	0,28	0,197	1232,646	321,576
PNP	0,001	0,076	0,291	0,384	0,441	0,2	815,778	334,437
MPG	0,002	0,077	0,291	0,392	0,526	0,425	148,722	66,145
AMFR	0,001	0,077	0,291	0,289	0,247	-0,226	840,293	224,959
BBS2	0,003	0,077	0,291	0,216	0,161	-0,429	287,459	53,617
CMC2	0,002	0,077	0,291	0,382	0,493	0,366	200,375	86,747
RAB5C	0,002	0,076	0,291	1,019	1,368	0,425	78,277	92,624
RPL36	0,001	0,077	0,291	1,377	1,27	-0,117	1907,376	2527,89
VPS16	0,003	0,076	0,291	0,276	0,203	-0,446	217,346	50,75
PRMT6	0,003	0,078	0,292	0,172	0,125	-0,467	301,004	45,296
GORASP2	0,001	0,077	0,292	0,187	0,216	0,207	1316,347	263,4
ATP2B1	0,001	0,078	0,292	0,126	0,151	0,265	1027,862	143,554
ITGB4	0,001	0,078	0,292	0,141	0,157	0,155	4020,194	600,064
UBE2A	0,001	0,078	0,292	0,387	0,439	0,183	1004,185	416,633
ADO	0,002	0,078	0,293	0,18	0,146	-0,3	679,883	111,085
TIMM9	0,002	0,078	0,293	0,26	0,339	0,384	238,592	71,825
SDF2L1	0,002	0,078	0,293	0,867	1,106	0,35	129,124	125,998
ACSL3	0,001	0,078	0,294	0,126	0,147	0,222	1564	213,437
ACAT1	0,001	0,078	0,294	0,353	0,405	0,198	867,79	328,201
SS18L2	0,003	0,079	0,295	0,886	0,624	-0,506	70,309	52,472
INSIG1	0,003	0,079	0,295	0,188	0,137	-0,457	285,679	46,741
ZNF384	0,002	0,079	0,295	0,094	0,12	0,357	687,929	72,502
ATP5B	0,001	0,079	0,295	0,561	0,518	-0,116	4727,209	2534,762

ATP5A1	0,001	0,079	0,295	0,457	0,407	-0,169	1151,956	497,363
RBM28	0,003	0,079	0,296	0,085	0,063	-0,433	637,994	47,075
UBR5	0,002	0,079	0,296	0,06	0,073	0,286	1696,657	112,425
TNFRSF12A	0,001	0,079	0,296	0,493	0,44	-0,162	1283,895	604,421
S100A6	0,001	0,08	0,297	3,831	3,524	-0,121	1405,079	5188,467
KIAA0922	0,003	0,08	0,297	0,169	0,225	0,413	290,754	56,826
MKL2	0,002	0,08	0,297	0,153	0,199	0,378	383,934	67,361
NCOA5	0,002	0,08	0,297	0,112	0,088	-0,345	775,709	77,156
MORF4L2	0,001	0,08	0,297	0,197	0,218	0,148	3405,788	705,58
TMEM222	0,003	0,081	0,298	0,261	0,197	-0,407	265,096	60,955
MKRN2	0,001	0,081	0,298	0,251	0,208	-0,27	629,126	142,784
TSC22D2	0,001	0,08	0,298	0,32	0,388	0,278	422,761	147,637
LETM1	0,001	0,081	0,298	0,133	0,157	0,245	1148,615	166,529
PCYOX1L	0,002	0,08	0,298	0,429	0,331	-0,376	208,333	78,764
GNB2L1	0,001	0,081	0,298	0,551	0,514	-0,099	12177,724	6473,552
MED20	0,002	0,081	0,298	0,242	0,189	-0,355	368,749	79,197
MB21D1	0,002	0,081	0,298	0,152	0,118	-0,371	503,634	67,832
ZMIZ2	0,001	0,08	0,298	0,067	0,081	0,281	1574,109	115,51
EIF4H	0,003	0,081	0,298	0,304	0,224	-0,441	198,137	52,201
PSMC3	0,001	0,08	0,298	0,355	0,392	0,144	2147,007	797,64
SLC3A2	0,001	0,08	0,298	1,014	1,099	0,116	2883	3054,766
EHD1	0,001	0,081	0,298	0,19	0,171	-0,152	2980,281	539,091
CCDC90B	0,003	0,081	0,298	0,239	0,171	-0,483	235,245	44,499
AP1G1	0,001	0,081	0,298	0,088	0,105	0,261	1451,554	141,942
BAIAP2	0,001	0,08	0,298	0,276	0,325	0,236	693,007	206,935
ACSS2	0,003	0,081	0,298	0,189	0,14	-0,428	322,847	54,071
MAPK12	0,002	0,08	0,298	0,105	0,085	-0,293	1200,846	115,705
RCC2	0,001	0,081	0,299	0,205	0,173	-0,247	891,189	166,46
SPECC1	0,002	0,081	0,299	0,11	0,142	0,364	557,584	69,713
ABHD12	0,002	0,081	0,299	0,276	0,216	-0,351	338,565	83,54
MAPKAP1	0,002	0,082	0,3	0,074	0,06	-0,302	1463,843	98,976
IDI1	0,002	0,082	0,3	0,223	0,289	0,369	291,872	73,728

SCAMP3	0,002	0,082	0,301	0,343	0,469	0,454	169,16	63,394
TMEM237	0,002	0,082	0,301	0,206	0,159	-0,371	383,566	70,122
POT1	0,003	0,082	0,301	0,166	0,22	0,407	297,326	58,051
RSPRY1	0,002	0,082	0,301	0,138	0,107	-0,366	559,274	67,793
FAM83G	0,001	0,082	0,301	0,112	0,133	0,25	1328,847	160,427
MED15	0,001	0,082	0,301	0,151	0,178	0,235	1141,795	188,078
FNDC3B	0,002	0,083	0,302	0,087	0,068	-0,357	895,279	69,128
UQCRC2	0,001	0,083	0,302	0,355	0,313	-0,178	1184,718	394,308
RAP1A	0,002	0,084	0,303	0,27	0,331	0,294	406,383	123,474
KISS1	0,001	0,083	0,303	1,387	1,237	-0,165	600,073	784,061
CD55	0,001	0,084	0,303	0,422	0,492	0,223	603,554	269,157
TKT	0,001	0,084	0,303	0,344	0,299	-0,206	851,961	272,211
ZFYVE16	0,001	0,083	0,303	0,129	0,156	0,28	852,23	122,283
MTFR2	0,003	0,084	0,303	0,335	0,464	0,471	120,772	48,481
RELN	0,002	0,084	0,303	58,546	19,016	-1,622	2,539	85,609
FAM160B2	0,003	0,083	0,303	0,175	0,128	-0,459	292,407	44,706
DNAJA1	0,001	0,083	0,303	0,186	0,208	0,164	2473,535	487,053
STRBP	0,002	0,083	0,303	0,127	0,162	0,351	517,002	75,108
MRPL41	0,001	0,084	0,303	0,912	1,082	0,246	264,131	262,242
SGPL1	0,002	0,084	0,303	0,124	0,156	0,33	603,125	84,769
ZDHHC6	0,002	0,083	0,303	0,181	0,145	-0,314	593,343	96,461
IKBIP	0,001	0,084	0,303	0,352	0,409	0,215	678,473	259,149
RNF10	0,002	0,084	0,303	0,058	0,071	0,294	1618,565	103,961
OAZ2	0,002	0,083	0,303	0,24	0,196	-0,293	547,858	119,522
FAM173A	0,004	0,084	0,303	0,97	0,656	-0,565	52,029	41,613
SPG7	0,003	0,084	0,303	0,129	0,097	-0,414	463,068	52,197
RAD51C	0,002	0,084	0,303	0,544	0,435	-0,323	231,127	112,163
SUPT5H	0,001	0,083	0,303	0,062	0,074	0,271	1802,489	122,653
PRMT1	0,001	0,084	0,303	0,357	0,392	0,137	2369,178	885,536
DNAJC5	0,001	0,084	0,303	0,096	0,08	-0,274	1380,266	120,688
TRAPPC10	0,003	0,084	0,303	0,077	0,058	-0,4	794,912	53,256
GTSE1	0,001	0,083	0,303	0,138	0,121	-0,193	2186,208	283,033

MAT2A	0,001	0,084	0,304	0,194	0,218	0,168	2025,554	418,083
BMP2K	0,003	0,085	0,304	0,164	0,22	0,427	280,799	52,23
TARS	0,001	0,085	0,304	0,171	0,188	0,133	5100,851	914,96
ENSG00000251562.3	0,001	0,085	0,304	0,123	0,146	0,249	1185,588	162,63
RIOK3	0,002	0,085	0,304	0,099	0,123	0,311	837,64	92,746
OSBPL2	0,003	0,085	0,304	0,144	0,194	0,43	292,189	49,579
PKNOX1	0,003	0,084	0,304	0,197	0,263	0,418	238,33	55,166
FASTKD5	0,003	0,085	0,305	0,151	0,199	0,398	332,464	58,737
DIP2A	0,003	0,085	0,305	0,1	0,075	-0,43	536,604	46,871
NDRG3	0,001	0,085	0,306	0,249	0,292	0,234	731,39	196,787
YAP1	0,001	0,086	0,307	0,219	0,248	0,182	1432,267	335,73
TMEM55B	0,003	0,087	0,309	0,281	0,376	0,418	177,733	57,906
RPL11	0,001	0,087	0,31	0,777	0,838	0,109	3365,18	2717,159
UXS1	0,002	0,087	0,31	0,25	0,314	0,328	327,568	93,138
CNPPD1	0,002	0,087	0,31	0,242	0,301	0,31	393,326	105,024
OSBPL5	0,005	0,087	0,31	0,152	0,103	-0,568	250,156	33,662
SHISA2	0,003	0,087	0,31	0,165	0,126	-0,385	423,9	60,097
SPTLC2	0,002	0,087	0,31	0,127	0,1	-0,337	695,74	78,922
PCYT2	0,002	0,087	0,31	0,183	0,145	-0,334	511,225	84,194
MYL12B	0,001	0,087	0,31	0,443	0,488	0,139	1898,063	880,961
TMEM205	0,001	0,087	0,31	0,969	0,782	-0,31	171,018	150,956
ETS2	0,001	0,087	0,31	0,202	0,241	0,254	710,82	156,545
MAGEB2	0,001	0,087	0,31	0,438	0,384	-0,191	830,259	342,962
KLHL21	0,002	0,088	0,311	0,165	0,134	-0,294	720,394	108,095
TMEM177	0,003	0,088	0,311	0,399	0,282	-0,501	122,124	42,245
DCAF10	0,003	0,088	0,311	0,149	0,113	-0,394	436,702	56,906
ARAP1	0,001	0,088	0,311	0,148	0,176	0,244	1032,887	166,038
RFC5	0,001	0,088	0,311	0,468	0,404	-0,209	630,886	272,528
BNIP2	0,002	0,088	0,311	0,176	0,143	-0,295	672,231	105,999
MED16	0,001	0,088	0,311	0,204	0,244	0,252	706,101	157,036
CSE1L	0,001	0,088	0,311	0,332	0,36	0,119	5049,787	1755,707
FOXRED2	0,003	0,088	0,311	0,212	0,16	-0,405	300,586	54,974

KDELR2	0,001	0,089	0,312	0,43	0,389	-0,147	1753,815	713,778
CAV2	0,001	0,089	0,312	0,244	0,204	-0,261	657,525	145,736
PITPNM1	0,002	0,089	0,312	0,075	0,06	-0,321	1227,869	83,373
TMEM126B	0,002	0,089	0,312	0,366	0,3	-0,287	384,39	127,492
USP5	0,003	0,089	0,312	0,151	0,113	-0,413	394,411	52,428
ENSG00000234912.5	0,001	0,088	0,312	10,79	8,198	-0,396	46,83	448,798
GAMT	0,001	0,089	0,312	0,473	0,39	-0,276	375,42	165,976
MFSD12	0,001	0,089	0,312	0,197	0,17	-0,213	1241,957	229,686
CCDC19	0,003	0,089	0,313	0,205	0,271	0,399	253,493	59,336
ZNF106	0,002	0,089	0,313	0,042	0,033	-0,357	1741,938	65,412
NFAT5	0,002	0,089	0,313	0,079	0,099	0,318	971,679	87,354
MAU2	0,002	0,089	0,313	0,157	0,189	0,273	739,473	127,156
DNAJB4	0,003	0,09	0,314	0,182	0,244	0,423	239,917	50,773
ADAM9	0,001	0,09	0,314	0,195	0,216	0,143	3008,338	617,7
C9orf64	0,002	0,09	0,314	0,273	0,213	-0,358	305,247	73,488
FAM188A	0,003	0,09	0,314	0,302	0,228	-0,407	220,892	56,909
ZNF598	0,002	0,09	0,314	0,122	0,096	-0,356	634,165	69,558
SERTAD1	0,003	0,09	0,314	0,411	0,554	0,43	121,925	59,574
IGBP1	0,002	0,09	0,314	0,335	0,266	-0,333	299,821	88,821
ABL1	0,002	0,09	0,315	0,046	0,035	-0,362	1559,648	63,819
COTL1	0,001	0,09	0,315	0,188	0,171	-0,133	4489,902	806,826
MTOR	0,001	0,091	0,316	0,186	0,163	-0,19	1707,534	296,779
PRELID1	0,001	0,091	0,316	0,424	0,347	-0,286	338,468	129,314
ATP6V0E2	0,004	0,091	0,316	0,144	0,104	-0,467	316,921	39,218
FXN	0,002	0,091	0,316	0,435	0,329	-0,403	166,515	63,175
FUBP3	0,001	0,091	0,316	0,203	0,23	0,184	1442,782	311,427
UBAC2	0,001	0,091	0,316	0,293	0,24	-0,284	462,636	122,901
PRC1	0,001	0,091	0,316	0,111	0,128	0,197	2064,88	246,175
PPP5C	0,001	0,091	0,316	0,247	0,283	0,196	1058,734	280,218
ACO2	0,001	0,09	0,316	0,176	0,209	0,244	865,182	165,483
TBC1D22A	0,002	0,091	0,316	0,186	0,147	-0,344	455,422	75,321
C10orf137	0,002	0,091	0,317	0,244	0,196	-0,316	432,25	94,46

TLCD1	0,004	0,092	0,317	0,636	0,439	-0,534	73,231	39,456
DDX49	0,001	0,091	0,317	0,334	0,283	-0,239	599,907	183,799
ENSG00000211459.2	0,001	0,091	0,317	0,059	0,064	0,137	10871,027	663,451
DCTN1	0,001	0,092	0,318	0,08	0,09	0,165	4673,897	398,089
TMSB4X	0,002	0,092	0,318	0,332	0,255	-0,382	226,577	65,616
LMAN2L	0,002	0,092	0,319	0,443	0,359	-0,304	287,895	113,055
VPS37C	0,002	0,092	0,319	0,179	0,23	0,359	351,51	70,702
RFX5	0,003	0,093	0,32	0,073	0,096	0,404	600,457	51,58
MCTP1	0,005	0,093	0,32	0,162	0,115	-0,497	282,633	40,369
FAM196B	0,003	0,093	0,32	0,106	0,139	0,402	428,306	53,33
PERP	0,001	0,093	0,32	0,517	0,438	-0,237	432,816	207,452
CDK5	0,003	0,093	0,32	0,499	0,364	-0,456	117,757	51,128
ZFAND1	0,002	0,093	0,32	0,199	0,252	0,336	361,676	82,01
JMJD1C	0,001	0,093	0,32	0,057	0,068	0,251	2167,279	134,936
FKBP2	0,001	0,093	0,32	0,477	0,565	0,244	395,29	202,617
PDRG1	0,002	0,093	0,32	0,4	0,318	-0,33	260,36	93,189
UFD1L	0,001	0,093	0,32	0,368	0,442	0,262	389,064	156,825
SYPL1	0,001	0,094	0,321	0,764	0,678	-0,171	721,129	514,679
WWP2	0,002	0,094	0,321	0,086	0,107	0,309	919,085	89,812
CSTF2	0,001	0,094	0,321	0,36	0,414	0,201	732,919	284,316
CEPT1	0,001	0,094	0,322	0,621	0,757	0,287	217,809	150,28
CIAO1	0,001	0,094	0,322	0,268	0,233	-0,2	1028,999	257,7
RPA3	0,001	0,094	0,322	1,494	1,834	0,296	126,471	211,654
FADS2	0,001	0,094	0,322	0,159	0,178	0,157	2775,168	464,92
DPP3	0,001	0,094	0,322	0,341	0,398	0,221	611,214	225,334
ATP6V0A1	0,002	0,094	0,322	0,116	0,144	0,315	675,458	87,468
NDC80	0,001	0,094	0,322	0,163	0,192	0,238	930,273	164,851
COG6	0,002	0,095	0,323	0,266	0,213	-0,324	371,906	89,125
BRI3	0,002	0,095	0,324	0,577	0,439	-0,393	148,223	77,465
ARHGEF17	0,003	0,095	0,324	0,074	0,055	-0,421	704,026	45,864
RPAIN	0,001	0,095	0,324	0,725	0,611	-0,246	311,278	205,678
ALG14	0,003	0,095	0,325	0,39	0,284	-0,458	136,866	45,677

WDR55	0,002	0,095	0,325	0,258	0,206	-0,325	376,691	87,061
PPIA	0,001	0,095	0,325	1,161	1,035	-0,166	586,68	642,056
KMT2C	0,002	0,096	0,325	0,069	0,053	-0,378	908,62	55,611
HIPK3	0,003	0,096	0,325	0,092	0,069	-0,407	614,543	49,739
VRK1	0,002	0,096	0,325	0,24	0,29	0,275	480,507	127,561
PCGF1	0,002	0,096	0,326	0,402	0,501	0,318	235,33	104,702
PITX1	0,002	0,096	0,326	0,238	0,182	-0,389	284,346	60,17
NGFRAP1	0,001	0,096	0,326	0,629	0,555	-0,18	706,252	420,087
ZMYND11	0,002	0,097	0,327	0,079	0,062	-0,353	917,665	63,711
MED29	0,002	0,097	0,327	0,161	0,126	-0,357	468,988	66,09
ZBTB40	0,002	0,097	0,328	0,107	0,083	-0,366	635,625	59,875
UBA3	0,001	0,097	0,328	0,275	0,235	-0,225	761,264	193,38
ENSG00000245910.4	0,001	0,097	0,328	1,033	1,268	0,296	148,466	170,107
TAF2	0,002	0,097	0,328	0,098	0,119	0,283	972,85	106,433
CTNNAL1	0,001	0,097	0,328	0,283	0,308	0,12	4133,874	1227,307
TRAFD1	0,003	0,097	0,328	0,244	0,179	-0,449	207,317	43,819
RNF6	0,002	0,097	0,328	0,103	0,125	0,281	950,049	108,144
B2M	0,001	0,097	0,328	0,572	0,621	0,119	2603,467	1552,052
PDCD7	0,003	0,097	0,328	0,197	0,146	-0,426	277,324	47,687
DKC1	0,002	0,097	0,328	0,133	0,172	0,379	383,574	58,467
RNF181	0,001	0,098	0,329	1,092	0,932	-0,228	284,977	288,928
BNIP1	0,002	0,098	0,329	0,573	0,436	-0,393	136,51	68,036
MPV17	0,002	0,098	0,33	0,395	0,319	-0,309	317,373	116,164
DUSP11	0,001	0,098	0,33	0,267	0,319	0,254	513,429	150,456
RGS3	0,002	0,098	0,33	0,195	0,159	-0,298	560,527	98,85
TCIRG1	0,001	0,098	0,33	0,187	0,158	-0,236	984,338	170,794
GPC6	0,003	0,099	0,331	0,178	0,241	0,441	228,728	45,988
SGTA	0,001	0,099	0,331	0,225	0,251	0,157	1874,324	444,629
DDX18	0,002	0,099	0,332	0,089	0,072	-0,295	1155,862	91,468
C7orf50	0,002	0,099	0,332	0,24	0,191	-0,333	371,911	79,865
CCDC132	0,003	0,099	0,332	0,149	0,198	0,414	287,619	51,127
SNRNP40	0,001	0,1	0,333	0,494	0,424	-0,22	501,377	230,465

CKAP2L	0,002	0,1	0,333	0,081	0,064	-0,324	1042,729	74,794
LMBRD2	0,002	0,1	0,333	0,157	0,203	0,367	348,748	63,863
CMTR1	0,002	0,1	0,333	0,108	0,085	-0,352	671,818	63,861
MACROD1	0,003	0,1	0,333	0,537	0,722	0,426	99,862	61,382
UBN1	0,003	0,1	0,333	0,05	0,038	-0,402	1066,496	46,416
UPF3B	0,003	0,1	0,333	0,093	0,12	0,377	523,31	55,848
GNPAT	0,002	0,1	0,334	0,23	0,188	-0,292	499,571	104,336
TOPBP1	0,002	0,1	0,334	0,087	0,104	0,265	1232,243	118,217
H2AFY	0,001	0,1	0,334	0,107	0,123	0,2	2011,751	229,783
TMEM30A	0,001	0,1	0,334	0,335	0,373	0,155	1409,252	502,238
ACADM	0,001	0,101	0,335	0,278	0,236	-0,232	701,351	180,862
WWC1	0,002	0,101	0,335	0,062	0,049	-0,338	1219,424	67,838
PFKFB3	0,003	0,101	0,335	0,104	0,079	-0,388	580,968	51,429
MFSD11	0,003	0,101	0,335	0,812	0,593	-0,452	82,512	55,716
RCHY1	0,003	0,101	0,336	0,377	0,28	-0,429	154,469	50,589
MAD2L1	0,001	0,102	0,336	0,474	0,402	-0,237	437,46	190,326
MBNL2	0,002	0,101	0,336	0,107	0,088	-0,28	1069,886	103,846
TANK	0,002	0,102	0,338	0,219	0,281	0,361	277,075	68,089
HSPA4L	0,003	0,102	0,338	0,081	0,105	0,374	597,159	55,419
HBEGF	0,001	0,102	0,338	0,201	0,172	-0,226	973,055	180,121
ZNF259	0,001	0,102	0,338	0,196	0,226	0,209	1030,034	216,148
SCAF1	0,001	0,102	0,338	0,116	0,097	-0,259	1179,594	126,132
SRP72	0,001	0,103	0,339	0,147	0,169	0,203	1479,068	232,753
SCD5	0,001	0,103	0,339	0,39	0,331	-0,238	496,003	176,665
SIL1	0,002	0,103	0,339	0,427	0,527	0,304	236,457	111,818
CNOT8	0,002	0,103	0,339	0,234	0,19	-0,303	452,128	94,394
ARHGAP12	0,003	0,103	0,339	0,109	0,082	-0,411	480,569	45,232
HMGA2	0,002	0,103	0,339	0,101	0,128	0,343	595,921	67,623
DLGAP5	0,001	0,102	0,339	0,111	0,126	0,188	2192,063	260,687
CCDC137	0,003	0,103	0,339	0,089	0,114	0,36	607,888	61,269
NCOA3	0,002	0,103	0,339	0,116	0,148	0,349	501,966	66,804
FAM114A1	0,002	0,103	0,34	0,316	0,392	0,313	277,291	97,324

CCND3	0,001	0,103	0,34	0,281	0,247	-0,187	1115,018	295,551
RNF121	0,002	0,104	0,34	0,469	0,37	-0,34	203,521	84,23
MRPS11	0,001	0,104	0,34	0,322	0,385	0,259	416,73	146,215
PEX2	0,002	0,104	0,341	0,342	0,277	-0,302	327,825	100,885
CC2D1B	0,002	0,104	0,342	0,13	0,104	-0,326	651,755	77,072
TMCO1	0,001	0,105	0,342	0,394	0,443	0,169	1007,549	422,956
POC5	0,002	0,104	0,342	0,346	0,429	0,311	257,02	99,986
ZDHHC24	0,003	0,105	0,342	0,65	0,464	-0,486	79,375	44,102
KRT7	0,001	0,105	0,342	0,301	0,265	-0,185	1151,712	329,318
CNOT2	0,002	0,105	0,342	0,138	0,169	0,287	655,032	101,541
PDK2	0,002	0,104	0,342	0,339	0,442	0,383	172,643	65,708
STRN4	0,002	0,105	0,342	0,11	0,092	-0,254	1300,263	132,526
TST	0,001	0,104	0,342	0,996	0,873	-0,191	433,77	404,093
SDF4	0,001	0,105	0,343	0,259	0,295	0,187	1052,539	289,168
B4GALT3	0,002	0,105	0,343	0,131	0,106	-0,306	744,676	89,51
NFATC3	0,002	0,105	0,343	0,114	0,146	0,354	483,724	63,536
ENO1	0,001	0,106	0,344	0,287	0,269	-0,093	20377,392	5676,502
NCKIPSD	0,002	0,106	0,344	0,158	0,191	0,273	657,089	114,207
SASS6	0,002	0,106	0,345	0,14	0,179	0,358	396,019	63,053
PDAP1	0,002	0,106	0,345	0,199	0,249	0,322	369,414	83,731
ELOVL1	0,001	0,107	0,346	0,581	0,634	0,126	1834,623	1106,428
ANTXR2	0,001	0,107	0,346	0,146	0,175	0,258	782,86	125,18
GOLPH3	0,001	0,107	0,346	0,22	0,19	-0,212	972,181	198,312
NUDCD2	0,002	0,106	0,346	0,393	0,321	-0,291	314,039	111,047
NHP2	0,001	0,107	0,346	0,952	0,792	-0,266	205,052	177,125
CLIC1	0,001	0,107	0,346	0,66	0,558	-0,241	321,85	196,631
RMI1	0,002	0,106	0,346	0,164	0,204	0,319	441,371	81,252
ATXN2	0,002	0,107	0,346	0,083	0,104	0,335	733,701	68,043
RPL36AL	0,001	0,107	0,346	0,691	0,569	-0,279	228,37	144,388
RCN2	0,001	0,107	0,346	0,325	0,378	0,22	585,969	206,709
PELP1	0,001	0,107	0,346	0,217	0,251	0,207	951,35	220,525
LLGL1	0,001	0,107	0,346	0,236	0,204	-0,209	978,646	215,777

TIMP2	0,002	0,107	0,346	0,118	0,099	-0,256	1159,027	125,208
SSBP4	0,003	0,107	0,346	0,173	0,128	-0,435	286,623	43,934
SRM	0,001	0,108	0,347	0,321	0,288	-0,158	1514,522	461,758
FAM13B	0,002	0,108	0,347	0,075	0,096	0,358	685,629	58,958
ERMARD	0,002	0,108	0,347	0,378	0,291	-0,377	190,661	63,62
GSTO1	0,001	0,108	0,347	1,11	0,98	-0,18	451,433	469,922
TSEN54	0,003	0,108	0,347	0,222	0,169	-0,396	268,915	52,399
CAPN2	0,001	0,108	0,348	0,156	0,142	-0,133	5010,017	752,35
CEP57	0,002	0,108	0,348	0,115	0,14	0,283	773,504	99,869
SERF2	0,001	0,108	0,348	0,59	0,516	-0,193	561,571	310,708
MIEF1	0,003	0,108	0,348	0,081	0,063	-0,36	795,753	56,867
ARHGAP29	0,001	0,109	0,349	0,134	0,147	0,129	5052,885	705,24
HINT2	0,002	0,109	0,349	1,051	0,827	-0,346	111,206	103,665
CAMTA1	0,003	0,109	0,35	0,296	0,219	-0,437	171,917	43,934
POU2F1	0,002	0,11	0,35	0,153	0,122	-0,325	538,585	73,332
KIF14	0,002	0,109	0,35	0,064	0,078	0,287	1278,456	90,487
DYSF	0,001	0,11	0,35	0,066	0,056	-0,225	2511,619	153,737
RFTN1	0,001	0,11	0,35	0,165	0,137	-0,268	756,353	114,94
ABHD10	0,001	0,11	0,35	0,435	0,37	-0,232	457,371	184,085
TMEM181	0,003	0,11	0,35	0,139	0,107	-0,378	439,198	53,861
NUDCD3	0,001	0,11	0,35	0,125	0,15	0,261	856,29	118,403
IMPA1	0,002	0,11	0,35	0,498	0,394	-0,339	203,109	93,023
GSDMD	0,001	0,109	0,35	0,357	0,295	-0,275	380,19	124,91
TMEM132A	0,002	0,11	0,35	0,163	0,129	-0,344	459,094	67,136
HNRNPA1	0,001	0,11	0,35	0,173	0,206	0,247	737,734	140,939
CCDC53	0,002	0,11	0,35	0,484	0,371	-0,384	151,535	64,797
PDS5B	0,002	0,11	0,35	0,074	0,091	0,292	1085,648	89,493
EIF2S1	0,001	0,11	0,35	0,241	0,267	0,145	2078,453	530,014
NUBP1	0,002	0,109	0,35	0,846	1,129	0,416	74,114	72,193
CFDP1	0,002	0,11	0,35	0,192	0,243	0,342	335,718	75,247
DCXR	0,001	0,11	0,35	0,743	0,634	-0,229	324,426	222,873
MED14	0,001	0,11	0,35	0,161	0,188	0,221	1037,994	180,236

CMC1	0,002	0,11	0,351	0,888	0,665	-0,416	84,717	64,973
MLXIP	0,003	0,111	0,351	0,063	0,049	-0,374	940,416	52,636
UBE3A	0,002	0,11	0,351	0,108	0,13	0,271	905,912	107,972
SGSH	0,006	0,111	0,351	0,257	0,181	-0,503	161,899	35,876
TET3	0,003	0,111	0,352	0,042	0,032	-0,382	1300,618	48,383
TACC2	0,002	0,111	0,352	0,102	0,129	0,33	609,375	70,273
KPNA3	0,001	0,111	0,352	0,161	0,14	-0,203	1409,533	211,553
PSMB5	0,001	0,112	0,353	0,916	0,823	-0,153	732,232	636,99
GSTZ1	0,002	0,112	0,353	0,43	0,343	-0,325	226,401	87,132
CDKN2D	0,002	0,111	0,353	0,485	0,372	-0,382	150,425	63,415
GPI	0,001	0,112	0,353	0,339	0,313	-0,115	3477,4	1130,791
RBBP7	0,001	0,112	0,353	0,256	0,288	0,168	1314,507	357,465
COL4A3BP	0,003	0,112	0,354	0,153	0,119	-0,362	435,186	59,411
NOV	0,002	0,112	0,354	0,39	0,309	-0,335	229,871	80,545
SMNDC1	0,001	0,112	0,354	0,317	0,379	0,255	403,901	141,294
TSPAN3	0,001	0,112	0,354	0,393	0,449	0,191	678,731	284,709
UGGT1	0,001	0,113	0,355	0,12	0,139	0,213	1382,121	180,808
CCDC12	0,002	0,113	0,355	0,377	0,467	0,307	239,173	100,024
TUBB	0,002	0,113	0,355	1,299	0,991	-0,39	76,305	86,006
CLCF1	0,002	0,113	0,355	0,203	0,159	-0,35	378,474	69,832
METTL23	0,003	0,113	0,355	0,449	0,33	-0,445	117,471	44,856
AKT2	0,002	0,113	0,355	0,091	0,074	-0,295	1057,411	87,392
SLC35F3	0,003	0,113	0,356	0,374	0,281	-0,412	162,485	54,224
GLUD1	0,002	0,113	0,356	0,192	0,16	-0,266	653,808	113,991
TNFAIP2	0,001	0,113	0,356	0,142	0,121	-0,24	1084,514	142,024
SLC4A1AP	0,002	0,114	0,357	0,166	0,21	0,341	361,787	68,213
ANXA6	0,001	0,114	0,357	0,159	0,175	0,135	4241,664	706,031
ASCC3	0,002	0,114	0,357	0,075	0,059	-0,358	836,986	55,901
FNBP1	0,001	0,114	0,357	0,083	0,099	0,253	1356,716	121,917
TBCE	0,002	0,115	0,358	0,149	0,187	0,327	434,242	72,467
HIGD2A	0,002	0,114	0,358	0,399	0,323	-0,308	262,894	94,883
DKK1	0,001	0,115	0,358	0,519	0,575	0,146	1077,432	590,699

UACA	0,002	0,115	0,358	0,042	0,053	0,33	1348,977	64,324
TRAP1	0,001	0,115	0,358	0,405	0,357	-0,184	831,961	317,145
PYCR1	0,001	0,114	0,358	0,366	0,312	-0,231	521,314	177,389
CTU1	0,003	0,114	0,358	1,12	0,782	-0,517	47,158	44,461
TSR2	0,002	0,115	0,358	0,195	0,162	-0,272	621,594	110,983
COPE	0,001	0,115	0,359	0,635	0,565	-0,169	693,912	415,738
C21orf33	0,002	0,115	0,359	0,411	0,332	-0,31	253,265	94,32
EIF5B	0,002	0,116	0,36	0,019	0,024	0,289	3867,402	83,795
SLC35A4	0,002	0,116	0,36	0,083	0,1	0,267	1146,369	104,18
LRRC41	0,001	0,116	0,362	0,333	0,291	-0,196	821,275	258,573
SMARCB1	0,001	0,117	0,362	0,316	0,36	0,185	846,351	285,606
LYPLA2	0,002	0,117	0,363	0,275	0,214	-0,358	261,465	64,295
GLUL	0,002	0,118	0,363	0,155	0,12	-0,365	443,332	62,954
MRPL44	0,001	0,118	0,363	0,486	0,587	0,271	249,701	134,658
SEP2	0,001	0,117	0,363	0,192	0,176	-0,131	4044,85	742,109
ATP2C1	0,001	0,117	0,363	0,162	0,191	0,239	795,321	140,756
FCHO2	0,002	0,117	0,363	0,245	0,194	-0,336	326,752	69,914
NFYA	0,001	0,118	0,363	0,2	0,236	0,234	693,913	153,235
MAD1L1	0,002	0,118	0,363	0,211	0,254	0,265	506,651	117,317
POLD2	0,001	0,117	0,363	0,164	0,184	0,161	2166,535	374,699
PSMC2	0,001	0,117	0,363	0,642	0,577	-0,152	884,634	536,667
NMT2	0,002	0,117	0,363	0,148	0,185	0,321	448,63	75,786
CSTF2T	0,001	0,117	0,363	0,233	0,273	0,224	696,756	176,557
USP47	0,002	0,118	0,363	0,128	0,153	0,261	804,598	112,946
RPS25	0,001	0,118	0,363	23,704	34,17	0,528	19,847	559,526
METAP2	0,001	0,118	0,363	0,159	0,18	0,179	1575,306	267,173
SART3	0,001	0,117	0,363	0,131	0,155	0,242	924,685	133,008
MIPEP	0,004	0,118	0,363	0,304	0,22	-0,468	142,237	37,487
EDC3	0,003	0,118	0,363	0,195	0,152	-0,363	334,879	57,221
COMMD4	0,001	0,117	0,363	0,741	0,623	-0,249	259,89	176,238
UNC45A	0,002	0,118	0,363	0,166	0,137	-0,274	677,646	102,361
METTL16	0,002	0,118	0,363	0,095	0,115	0,276	921,885	97,277

ASXL1	0,001	0,117	0,363	0,069	0,061	-0,174	4182,684	272,021
CASK	0,003	0,118	0,363	0,174	0,131	-0,406	301,047	46,007
BRD3	0,002	0,119	0,365	0,148	0,119	-0,316	569,091	76,109
SFSWAP	0,002	0,119	0,365	0,129	0,157	0,288	632,694	90,073
VIMP	0,002	0,119	0,365	0,351	0,424	0,274	309,956	119,639
TSC2	0,002	0,119	0,366	0,143	0,119	-0,26	866,096	113,224
MCTS1	0,001	0,119	0,366	0,693	0,796	0,199	401,182	301,114
GNG12	0,001	0,12	0,367	0,075	0,067	-0,168	4362,759	310,582
RNPEP	0,001	0,12	0,367	0,302	0,258	-0,228	615,836	173,842
ADAM17	0,002	0,12	0,367	0,258	0,315	0,29	342,988	98,745
PSME2	0,002	0,12	0,367	0,746	0,612	-0,284	193,937	131,273
NAV1	0,003	0,12	0,368	0,058	0,044	-0,41	839,797	44,247
E4F1	0,003	0,121	0,369	0,272	0,208	-0,388	218,67	52,096
TRIM28	0,001	0,121	0,369	0,27	0,292	0,114	5271,034	1482,968
IARS2	0,001	0,121	0,37	0,138	0,159	0,209	1245,903	183,766
R3HDM1	0,002	0,121	0,37	0,073	0,091	0,309	892,858	73,58
AGO2	0,001	0,121	0,37	0,205	0,174	-0,238	750,198	141,004
NCDN	0,002	0,122	0,371	0,213	0,175	-0,28	522,368	101,521
SRSF11	0,001	0,122	0,371	0,189	0,21	0,155	1957,119	389,451
CERS2	0,001	0,122	0,371	0,394	0,43	0,124	2024,591	830,767
MMS22L	0,002	0,122	0,371	0,153	0,126	-0,285	654,091	90,114
HEBP2	0,003	0,122	0,371	0,263	0,195	-0,432	181,776	41,625
CBL	0,001	0,122	0,371	0,073	0,062	-0,251	1672,914	112,219
PNMA1	0,001	0,122	0,371	0,192	0,229	0,253	588	124,025
SPG21	0,002	0,122	0,371	0,269	0,224	-0,264	475,852	116,581
COX11	0,002	0,122	0,371	0,27	0,225	-0,267	460,382	113,247
PRKAA2	0,003	0,124	0,372	0,104	0,079	-0,395	490,2	45,055
JTB	0,001	0,123	0,372	0,274	0,232	-0,239	576,966	145,206
UNC50	0,002	0,124	0,372	0,526	0,652	0,311	170,56	100,719
SAP130	0,001	0,124	0,372	0,287	0,326	0,184	884,708	270,915
ALAS1	0,001	0,123	0,372	0,31	0,347	0,16	1154,633	379,207
UMPS	0,001	0,123	0,372	0,551	0,471	-0,226	373,662	190,527

MCCC1	0,003	0,123	0,372	0,258	0,196	-0,392	220,877	49,6
PAXIP1	0,002	0,123	0,372	0,214	0,172	-0,322	382,015	73,353
SH2B3	0,002	0,123	0,372	0,039	0,031	-0,333	1762,887	60,117
MED31	0,001	0,123	0,372	5,527	3,999	-0,467	30,062	139,345
FECH	0,003	0,123	0,372	0,308	0,234	-0,398	185,328	49,133
ATP6V1E1	0,001	0,123	0,372	0,496	0,576	0,216	404,735	215,876
NUP50	0,002	0,123	0,372	0,057	0,07	0,314	1091,015	69,315
PGK1	0,001	0,123	0,372	0,407	0,471	0,211	490,195	215,018
MAP7D3	0,001	0,123	0,372	0,073	0,084	0,214	2114,908	164,565
FLNA	0,001	0,123	0,372	0,075	0,084	0,158	4384,826	347,434
NPAS2	0,003	0,124	0,373	0,095	0,073	-0,378	613,313	52,711
FBXO3	0,002	0,124	0,373	0,345	0,28	-0,3	300,108	94,586
MGRN1	0,002	0,124	0,373	0,171	0,139	-0,303	517,85	79,743
ARHGAP17	0,001	0,124	0,373	0,169	0,202	0,253	690,088	125,444
MRPS12	0,001	0,124	0,373	0,815	0,7	-0,219	310,647	234,244
COL18A1	0,002	0,124	0,373	0,32	0,392	0,292	280,098	99,803
SLC39A1	0,001	0,125	0,374	0,289	0,262	-0,141	1902,884	526,871
PCYT1A	0,003	0,125	0,374	0,078	0,058	-0,414	580,221	39,445
WFS1	0,001	0,125	0,374	0,378	0,436	0,207	532,258	217,235
EDF1	0,001	0,125	0,374	0,435	0,481	0,146	1215,228	552,453
LRRC20	0,003	0,125	0,374	0,086	0,065	-0,397	584,908	44,642
MED17	0,001	0,125	0,374	0,235	0,278	0,237	561,04	144,089
EP400	0,001	0,124	0,374	0,146	0,169	0,214	1141,871	178,939
ADAT1	0,003	0,125	0,374	0,131	0,169	0,366	356,955	54,077
LGALS1	0	0,125	0,374	1,227	1,298	0,081	5081,56	6417,39
SUCLG2	0,002	0,125	0,375	0,363	0,299	-0,28	349,671	118,332
MOB1B	0,002	0,126	0,375	0,107	0,133	0,314	597,471	72,044
NUP153	0,001	0,126	0,375	0,092	0,106	0,207	1741,964	172,303
GPRC5A	0,001	0,126	0,375	0,309	0,332	0,102	5543,895	1772,123
DPH3	0,003	0,127	0,377	0,181	0,142	-0,352	361,861	58,052
CLDN11	0,003	0,127	0,378	0,508	0,392	-0,376	140,631	61,239
GRSF1	0,001	0,127	0,378	0,325	0,288	-0,177	958,331	292,658

ASS1	0,003	0,127	0,378	0,305	0,234	-0,382	201,641	52,73
TUBA1C	0,001	0,127	0,378	0,325	0,362	0,157	1160,553	396,671
STRN3	0,002	0,127	0,378	0,15	0,19	0,335	380,881	64,522
ATIC	0,001	0,128	0,379	0,335	0,367	0,129	1984,875	693,608
SDHA	0,001	0,127	0,379	0,252	0,221	-0,191	986,035	233,672
TANC2	0,002	0,128	0,379	0,058	0,048	-0,285	1591,927	85,866
TMEM104	0,001	0,128	0,379	0,177	0,209	0,237	734,285	139,618
CRLS1	0,002	0,127	0,379	0,343	0,28	-0,294	303,947	93,332
NEK6	0,001	0,128	0,38	0,177	0,15	-0,237	871,106	144,896
HP1BP3	0,001	0,129	0,381	0,121	0,137	0,181	1877,322	241,353
BOLA3	0,002	0,129	0,381	0,339	0,271	-0,319	260,09	79,204
UAP1L1	0,004	0,129	0,381	0,131	0,098	-0,43	330,475	38,304
QSER1	0,001	0,129	0,381	0,072	0,083	0,219	1928,125	148,508
WDR76	0,002	0,129	0,381	0,15	0,184	0,294	506,389	85,481
STAMBPL1	0,001	0,129	0,382	0,279	0,238	-0,231	582,814	149,537
UHRF1BP1L	0,002	0,129	0,382	0,131	0,161	0,298	553,113	80,144
MPRIP	0,001	0,129	0,382	0,048	0,042	-0,193	4209,694	189,707
USF1	0,002	0,13	0,383	0,25	0,207	-0,275	450,281	103,532
QRSL1	0,003	0,13	0,383	0,255	0,194	-0,39	218,79	47,965
FUCA2	0,001	0,13	0,383	0,322	0,365	0,182	807,905	277,325
CDS2	0,001	0,13	0,383	0,231	0,27	0,228	612,954	153,25
UBE2E1	0,001	0,13	0,384	0,417	0,369	-0,176	790,532	309,415
MBTPS1	0,002	0,131	0,384	0,081	0,066	-0,286	1114,788	82,065
RPS15	0,001	0,13	0,384	2,159	1,958	-0,141	548,796	1132,71
PELO	0,001	0,132	0,386	0,327	0,276	-0,241	465,51	140,717
SEC24A	0,001	0,131	0,386	0,211	0,247	0,228	675,117	152,615
KIAA1429	0,001	0,131	0,386	0,169	0,144	-0,234	861,364	134,01
ZFYVE21	0,003	0,131	0,386	0,236	0,181	-0,385	238,168	50,02
LSR	0,003	0,131	0,386	0,448	0,341	-0,391	136,785	52,858
SLC23A2	0,002	0,131	0,386	0,115	0,145	0,332	476,16	62,587
TPD52L2	0,001	0,131	0,386	0,228	0,207	-0,139	2380,673	517,608
ADIPOR1	0,002	0,132	0,387	0,119	0,098	-0,272	873,152	94,965

NDUFA5	0,003	0,132	0,387	0,324	0,244	-0,408	160,908	45,158
NOC3L	0,003	0,132	0,387	0,046	0,058	0,344	1033,36	54,295
ENSG00000202198.1	0,001	0,133	0,388	9,227	7,044	-0,389	37,443	301,386
HEATR2	0,001	0,133	0,388	0,301	0,266	-0,175	1035,538	294,907
VKORC1L1	0,001	0,133	0,388	0,114	0,096	-0,242	1122,889	117,705
DIS3L	0,003	0,133	0,388	0,107	0,084	-0,349	569,826	54,054
PRKCA	0,002	0,132	0,388	0,046	0,054	0,235	2478,226	123,314
SLC43A2	0,002	0,133	0,389	0,451	0,354	-0,352	167,241	67,583
CLUH	0,001	0,133	0,389	0,106	0,116	0,13	4768,057	529,105
TMEM14B	0,002	0,133	0,39	0,437	0,529	0,275	248,785	118,173
SEP7	0,001	0,134	0,39	0,186	0,158	-0,229	817,821	139,696
NOG	0,002	0,134	0,39	0,347	0,422	0,282	302,238	112,568
MCM8	0,002	0,134	0,39	0,105	0,126	0,265	850,326	99,507
LEMD2	0,003	0,135	0,392	0,127	0,162	0,349	383,778	55,501
NACA	0,001	0,134	0,392	0,708	0,763	0,107	1899,95	1395,511
SUB1	0,001	0,136	0,393	0,518	0,577	0,155	812,841	444,633
HMGXB3	0,002	0,135	0,393	0,071	0,058	-0,299	1117,094	71,967
RIOK1	0,002	0,135	0,393	0,144	0,176	0,287	532,336	85,441
YWHAG	0,001	0,135	0,393	0,277	0,255	-0,118	3098,938	819,966
ELP3	0,002	0,135	0,393	0,134	0,167	0,317	450,075	68,194
XPNPEP1	0,002	0,136	0,393	0,181	0,149	-0,28	542,703	89,167
CDK2AP2	0,001	0,135	0,393	0,315	0,369	0,23	456,12	155,338
PIGL	0,003	0,136	0,393	0,598	0,457	-0,387	109,993	57,481
CLPP	0,001	0,136	0,393	0,459	0,512	0,157	879,894	423,01
ZMYM3	0,001	0,135	0,393	0,161	0,186	0,209	1013,722	175,348
RNASEH2C	0,001	0,136	0,394	0,503	0,433	-0,216	409,389	190,565
IMPA2	0,003	0,136	0,394	0,284	0,222	-0,357	228,855	57,555
LTBP4	0,002	0,136	0,394	0,106	0,126	0,247	1007,702	115,578
TCEAL1	0,002	0,136	0,394	0,296	0,375	0,342	200,227	66,258
NOL7	0,002	0,137	0,395	0,986	0,765	-0,365	88,821	77,229
COMTD1	0,002	0,137	0,395	0,765	0,587	-0,384	94,902	64,37
HECTD1	0,002	0,137	0,395	0,048	0,059	0,287	1441,887	76,959

HAGH	0,003	0,137	0,395	0,333	0,255	-0,383	174,778	51,615
LONP1	0,001	0,137	0,395	0,118	0,106	-0,165	2656,31	298,362
PLOD1	0,001	0,137	0,396	0,266	0,297	0,162	1204,191	338,061
GMDS	0,003	0,138	0,397	0,285	0,222	-0,358	226,566	57,816
POLE	0,002	0,138	0,397	0,077	0,065	-0,241	1631,263	116,118
ALDOA	0,001	0,138	0,397	0,468	0,497	0,088	9722,856	4685,598
SLC9A1	0,002	0,138	0,398	0,182	0,219	0,263	530,115	105,222
ALDH9A1	0,001	0,139	0,398	0,367	0,329	-0,157	1047,058	364,042
URB2	0,001	0,138	0,398	0,343	0,292	-0,231	472,735	150,246
NISCH	0,002	0,139	0,398	0,103	0,085	-0,281	904,574	85,07
FAT4	0,002	0,138	0,398	0,167	0,21	0,333	331,09	62,813
RARS	0,001	0,139	0,398	0,335	0,304	-0,14	1570,138	498,709
FARS2	0,003	0,139	0,398	0,415	0,31	-0,421	119,857	43,139
AHR	0,003	0,139	0,398	0,108	0,086	-0,338	586,783	56,844
POLR3A	0,003	0,139	0,398	0,064	0,081	0,337	760,948	54,875
TAOK3	0,002	0,139	0,398	0,104	0,128	0,289	701,902	80,202
HMGB1	0,002	0,138	0,398	0,152	0,18	0,248	684,264	113,82
DYNC1LI2	0,001	0,138	0,398	0,189	0,164	-0,197	1093,407	193,971
NUFIP2	0,001	0,139	0,398	0,075	0,087	0,215	1795,658	145,645
IFI35	0,003	0,139	0,398	0,779	0,598	-0,382	93,134	63,98
BCAM	0,002	0,139	0,398	0,226	0,283	0,322	275,194	69,637
CRKL	0,002	0,138	0,398	0,109	0,091	-0,249	1088,867	108,755
ARID1A	0,001	0,14	0,399	0,148	0,165	0,158	2101,304	327,752
TRIM33	0,001	0,14	0,399	0,139	0,159	0,197	1279,484	189,982
SMIM15	0,002	0,139	0,399	0,191	0,16	-0,252	634,711	110,929
BOD1	0,002	0,14	0,399	0,163	0,133	-0,29	546,809	80,42
TSFM	0,002	0,14	0,399	0,559	0,436	-0,358	131,609	65,443
APPBP2	0,002	0,14	0,399	0,266	0,223	-0,255	462,845	112,204
TMEM259	0,001	0,14	0,399	0,207	0,184	-0,166	1526,853	299,569
ENSG00000239830.1	0,002	0,14	0,399	0,059	0,048	-0,297	1333,476	71,203
RECQL4	0,002	0,141	0,4	0,063	0,051	-0,308	1166,126	66,432
ENSG00000204272.6	0,002	0,141	0,4	0,325	0,392	0,269	307,314	108,951

VHL	0,002	0,141	0,401	0,136	0,108	-0,33	489,593	59,451
BCL9L	0,001	0,141	0,401	0,085	0,096	0,166	3128,435	279,714
HSPA8	0,001	0,141	0,401	0,247	0,262	0,087	12614,96	3210,659
GRB10	0,003	0,142	0,402	0,101	0,078	-0,366	526,42	47,075
MLEC	0,002	0,142	0,402	0,059	0,05	-0,221	2514,197	136,192
SETD1A	0,002	0,141	0,402	0,097	0,118	0,281	777,82	83,079
NCAPH2	0,002	0,142	0,402	0,351	0,292	-0,268	332,379	105,772
AAMP	0,001	0,142	0,403	0,405	0,364	-0,155	990,871	382,115
PNPLA2	0,001	0,142	0,403	0,309	0,349	0,174	847,992	278,517
TEAD4	0,001	0,142	0,403	0,182	0,209	0,204	900,63	176,803
NAP1L1	0,001	0,143	0,403	0,143	0,156	0,123	4320,699	649,518
DHX37	0,002	0,143	0,403	0,069	0,085	0,3	908,267	69,418
CTU2	0,002	0,142	0,403	0,249	0,2	-0,317	314,122	70,542
PXMP4	0,003	0,143	0,403	0,48	0,374	-0,358	145,841	62,47
GALNT2	0,001	0,143	0,404	0,208	0,238	0,195	925,152	204,376
MED7	0,003	0,143	0,404	0,514	0,393	-0,39	117,527	52,036
CLU	0,001	0,143	0,404	0,515	0,623	0,276	241,854	132,286
EIF3H	0,001	0,144	0,404	0,315	0,34	0,113	2597,758	852,641
NOTCH1	0,001	0,143	0,404	0,199	0,231	0,216	728,832	155,799
BTBD10	0,002	0,144	0,404	0,199	0,164	-0,277	493,949	89,697
SIAE	0,002	0,144	0,404	0,415	0,328	-0,338	180,122	66,352
KNTC1	0,002	0,143	0,404	0,093	0,113	0,278	819,614	84,975
MED6	0,003	0,143	0,404	0,229	0,293	0,36	209,685	55,443
MIS12	0,002	0,143	0,404	0,155	0,186	0,263	576,96	99,691
TBL1X	0,003	0,143	0,404	0,077	0,059	-0,381	616,242	41,969
LMTK2	0,003	0,145	0,405	0,07	0,089	0,359	601,023	47,364
SGK223	0,003	0,144	0,405	0,274	0,208	-0,4	186,26	45,679
GSKIP	0,003	0,144	0,405	0,357	0,276	-0,372	167,341	52,376
NARS	0,001	0,145	0,405	0,203	0,178	-0,194	1023,773	194,449
NUMBL	0,003	0,144	0,405	0,154	0,118	-0,379	338,829	46,603
PSMG1	0,001	0,145	0,405	0,339	0,394	0,216	467,468	171,933
VMA21	0,002	0,144	0,405	0,152	0,127	-0,261	708,387	97,533

CHST11	0,001	0,145	0,406	0,136	0,16	0,23	873,203	128,538
NUBP2	0,002	0,145	0,406	0,248	0,198	-0,324	296,9	66,247
ZRANB2	0,002	0,145	0,407	0,1	0,119	0,248	980,721	107,637
CSRP1	0,001	0,145	0,407	0,232	0,209	-0,148	1863,207	413,469
PPP4R2	0,002	0,146	0,407	0,144	0,175	0,279	536,02	85,6
PSMD2	0,001	0,146	0,407	0,222	0,239	0,107	5360,205	1230,443
RWDD1	0,002	0,146	0,407	0,214	0,173	-0,308	368,389	71,203
TMEM168	0,002	0,146	0,407	0,331	0,41	0,307	217,015	81,516
CREM	0,003	0,145	0,407	0,253	0,327	0,37	179,797	51,931
PIP5K1C	0,002	0,146	0,407	0,078	0,064	-0,283	1121,414	80,825
JUND	0,002	0,145	0,407	0,364	0,294	-0,309	245,168	81,556
PCNT	0,002	0,146	0,407	0,076	0,091	0,247	1250,88	104,483
HEATR5B	0,003	0,147	0,408	0,2	0,157	-0,347	307,218	54,88
ARL6IP6	0,001	0,147	0,408	0,545	0,46	-0,244	283,372	140,255
SSFA2	0,001	0,146	0,408	0,074	0,063	-0,221	1957,809	134,559
PNN	0,001	0,146	0,408	0,085	0,096	0,185	2149,91	193,768
GPATCH8	0,002	0,147	0,408	0,05	0,06	0,262	1609,305	88,345
EFCAB14	0,003	0,147	0,409	0,04	0,032	-0,331	1557,041	56,839
SETD2	0,002	0,147	0,409	0,065	0,077	0,227	1747,918	124,319
BTBD2	0,002	0,147	0,409	0,117	0,094	-0,312	638,212	68,665
RBL1	0,001	0,147	0,409	0,123	0,143	0,222	1006,525	134,878
SLC30A7	0,001	0,148	0,41	0,416	0,474	0,191	533,076	237,87
TOP2B	0,002	0,148	0,41	0,059	0,069	0,237	1710,123	110,101
DCAF16	0,002	0,148	0,41	0,11	0,137	0,312	538,029	68,306
TFDP1	0,001	0,148	0,41	0,096	0,084	-0,183	2262,661	204,302
KCTD3	0,002	0,148	0,411	0,143	0,174	0,289	493,133	78,992
GPN3	0,001	0,148	0,411	0,645	0,545	-0,243	252,589	148,865
SLC25A38	0,002	0,149	0,412	0,26	0,208	-0,324	276,298	64,492
PSMB3	0,001	0,149	0,412	1,478	1,346	-0,135	630,562	888,815
ZC3H15	0,001	0,15	0,413	0,138	0,16	0,214	996,277	148,445
MSMO1	0,002	0,149	0,413	0,681	0,557	-0,29	167,839	103,834
TDP2	0,002	0,149	0,413	0,231	0,28	0,277	352,663	90,139

CASC3	0,002	0,149	0,413	0,067	0,055	-0,273	1331,994	81,181
PRR12	0,002	0,15	0,413	0,172	0,205	0,249	593,415	110,582
COPS3	0,001	0,15	0,414	0,512	0,473	-0,113	2025,982	996,183
CELSR1	0,002	0,15	0,414	0,063	0,077	0,286	1061,524	73,601
TLE1	0,002	0,15	0,415	0,097	0,119	0,306	609,374	65,634
KDM1A	0,001	0,152	0,416	0,233	0,208	-0,162	1293,284	284,869
RAD54L	0,002	0,151	0,416	0,13	0,161	0,316	435,703	63,086
KIAA0907	0,002	0,152	0,416	0,172	0,146	-0,236	748,949	118,973
GTF3C2	0,002	0,151	0,416	0,144	0,176	0,296	451,671	72,565
SKP2	0,001	0,152	0,416	0,248	0,211	-0,232	569,25	129,739
OGFRL1	0,002	0,151	0,416	0,038	0,032	-0,228	3367,339	116,617
FBXO5	0,001	0,152	0,416	0,242	0,285	0,235	488,817	128,923
ENTPD7	0,003	0,151	0,416	0,217	0,167	-0,381	231,19	43,924
PPP1R14B	0,002	0,152	0,416	0,13	0,104	-0,315	547,785	64,69
KCTD12	0,002	0,152	0,416	0,181	0,152	-0,253	641,564	107,886
RPA1	0,001	0,152	0,416	0,172	0,159	-0,117	3876,668	641,69
SNF8	0,002	0,151	0,416	0,155	0,186	0,259	569,4	97,203
CIRBP	0,001	0,151	0,416	0,392	0,436	0,155	880,803	366,626
MIS18A	0,002	0,151	0,416	0,503	0,403	-0,321	166,578	74,626
MICALL1	0,002	0,151	0,416	0,087	0,104	0,262	991,491	93,32
CENPA	0,002	0,152	0,417	0,476	0,392	-0,281	226,051	97,1
SLC30A9	0,002	0,153	0,417	0,137	0,166	0,277	547,523	83,74
ARPC5L	0,001	0,153	0,417	0,411	0,357	-0,203	504,006	193,431
INTS6	0,002	0,152	0,417	0,127	0,157	0,312	450,908	64,613
NPM1	0,001	0,153	0,418	0,155	0,172	0,153	2028,006	330,737
MED30	0,001	0,153	0,418	0,687	0,577	-0,253	216,01	135,65
USP34	0,001	0,153	0,419	0,124	0,138	0,149	2531,279	333,375
CLINT1	0,001	0,154	0,419	0,137	0,155	0,178	1492,936	219,002
CLTB	0,001	0,154	0,419	0,384	0,343	-0,163	873,286	317,213
WASF1	0,002	0,154	0,419	0,224	0,18	-0,316	321,362	64,445
RAD23B	0,001	0,154	0,419	0,347	0,375	0,109	2751,319	991,995
COX15	0,002	0,154	0,419	0,219	0,18	-0,281	428,077	85,878

PCNXL3	0,001	0,154	0,419	0,108	0,093	-0,219	1323,778	133,478
ETS1	0,001	0,154	0,419	0,135	0,147	0,126	3745,936	531,28
C2CD5	0,002	0,153	0,419	0,098	0,12	0,294	641,739	70,041
LHFP	0,002	0,154	0,419	0,222	0,186	-0,258	500,986	102,572
WDR61	0,002	0,154	0,419	0,632	0,525	-0,266	204,422	117,946
RPL13	0,001	0,155	0,419	0,547	0,591	0,114	1576,379	895,948
CSNK1D	0,001	0,154	0,419	0,122	0,14	0,196	1370,711	177,949
MAST3	0,003	0,154	0,419	0,144	0,11	-0,396	309,041	39,515
BTAF1	0,001	0,155	0,42	0,099	0,116	0,229	1099,609	118,568
TSSC4	0,001	0,155	0,42	0,417	0,354	-0,234	357,758	136,778
TJP1	0,002	0,155	0,42	0,05	0,06	0,256	1670,203	91,399
ACOT8	0,003	0,155	0,42	0,547	0,419	-0,385	107,224	51,047
FANCD2	0,002	0,155	0,421	0,152	0,125	-0,287	547,376	74,656
PAPSS1	0,002	0,155	0,421	0,186	0,151	-0,301	418,491	70,782
POMGNT1	0,002	0,156	0,422	0,101	0,086	-0,24	1179,729	111,229
MRPL24	0,001	0,156	0,422	0,453	0,504	0,152	813,298	387,462
MYO1B	0,001	0,156	0,422	0,128	0,146	0,19	1374,054	187,745
SLC39A13	0,001	0,156	0,422	0,206	0,176	-0,223	708,91	135,697
DCPS	0,002	0,156	0,422	0,385	0,46	0,259	266,703	112,877
NAPA	0,001	0,156	0,422	0,379	0,428	0,176	653,188	262,857
SF3B14	0,001	0,157	0,423	0,866	0,965	0,156	504,575	463,862
CTGF	0,001	0,157	0,423	0,657	0,712	0,115	1483,665	1011,858
PPP1R8	0,002	0,158	0,424	0,214	0,255	0,253	447,543	104,849
IPO13	0,002	0,157	0,424	0,124	0,146	0,236	851,189	113,267
MRPL35	0,001	0,158	0,424	0,232	0,2	-0,22	643,984	138,087
CNBP	0,001	0,158	0,424	0,49	0,454	-0,113	1781,125	837,346
LRPAP1	0,001	0,158	0,424	0,396	0,444	0,164	733,526	307,712
DHX15	0,001	0,158	0,424	0,132	0,118	-0,163	2088,714	258,807
UBE2J1	0,001	0,158	0,424	0,138	0,117	-0,231	908,778	115,647
CLP1	0,002	0,157	0,424	0,462	0,559	0,273	206,728	105,434
PRDX5	0,001	0,158	0,424	0,651	0,583	-0,159	632,95	393,534
ALG5	0,001	0,158	0,424	0,731	0,848	0,214	262,88	206,921

DUS1L	0,003	0,158	0,424	0,057	0,073	0,34	760,706	49,053
DNMT1	0,001	0,158	0,424	0,062	0,057	-0,13	7535,118	449,818
MRPS14	0,002	0,159	0,425	0,876	0,719	-0,285	141,289	112,978
CHPF	0,002	0,159	0,425	0,2	0,24	0,263	448,353	97
SOAT1	0,001	0,159	0,426	0,489	0,538	0,136	1039,078	533,086
ECE2	0,003	0,159	0,426	0,788	0,587	-0,424	67,767	46,188
RAB1B	0,001	0,159	0,426	0,158	0,182	0,207	912,284	153,979
PSMA6	0,002	0,159	0,426	15,346	8,622	-0,832	6,852	76,552
MED11	0,002	0,159	0,426	0,536	0,433	-0,308	166,51	80,76
BTG3	0,002	0,159	0,426	0,278	0,336	0,273	303,666	92,737
ISG20L2	0,002	0,16	0,427	0,103	0,123	0,256	814,453	91,562
MRPL33	0,002	0,16	0,427	0,943	0,769	-0,294	127,645	110,176
HARS	0,001	0,16	0,427	0,246	0,276	0,166	1043,916	271,103
DNAJC15	0,001	0,16	0,427	0,464	0,529	0,189	471,45	233,003
ENSG00000200783.1	0,003	0,16	0,427	16,668	8,08	-1,045	4,285	50,854
ZNF507	0,002	0,16	0,427	0,1	0,122	0,289	634,8	70,674
ACTN4	0,001	0,16	0,427	0,13	0,139	0,106	12149,927	1630,875
ZNF281	0,002	0,161	0,428	0,117	0,144	0,296	520,401	67,676
SLC44A1	0,001	0,161	0,428	0,253	0,219	-0,209	661,229	155,048
CDC42BPB	0,002	0,161	0,428	0,073	0,062	-0,237	1575,237	105,237
DVL2	0,001	0,161	0,428	0,198	0,176	-0,171	1288,17	240,622
DDX3X	0,001	0,161	0,428	0,112	0,123	0,134	3681,585	433,123
ATG9A	0,001	0,162	0,43	0,155	0,178	0,2	982,935	162,354
RPS15A	0,002	0,162	0,43	0,319	0,257	-0,31	241,772	69,579
IP6K2	0,002	0,163	0,431	0,196	0,234	0,258	447,709	95,635
WTAP	0,002	0,163	0,431	0,284	0,336	0,243	374,671	116,726
DSC2	0,003	0,162	0,431	0,243	0,311	0,354	189,652	51,688
AES	0,001	0,163	0,431	0,266	0,238	-0,161	1143,988	288,597
AIMP2	0,001	0,163	0,432	0,644	0,727	0,176	443,195	302,4
ZNF282	0,003	0,163	0,432	0,175	0,138	-0,35	313,234	48,975
RACGAP1	0,001	0,163	0,432	0,147	0,166	0,169	1488,934	233,82
UQCRH	0,001	0,163	0,433	0,935	1,056	0,175	354,437	350,123

GSTM3	0,002	0,164	0,433	0,661	0,795	0,266	170,071	122,404
INTS8	0,002	0,164	0,433	0,238	0,197	-0,271	396,436	85,634
CSTB	0,001	0,163	0,433	0,516	0,581	0,17	555,2	302,171
OGG1	0,002	0,164	0,434	0,204	0,248	0,281	359,705	80,647
HNRNPH1	0,001	0,164	0,434	0,142	0,153	0,105	6357,5	939,249
HOMER3	0,002	0,164	0,434	0,243	0,198	-0,294	342,052	76,14
EPHA2	0,001	0,165	0,435	0,278	0,297	0,097	4523,998	1304,391
BACE1	0,002	0,165	0,435	0,079	0,064	-0,308	821,071	58,876
LAMP1	0,001	0,165	0,435	0,713	0,76	0,093	2424,581	1785,249
SHOC2	0,002	0,166	0,436	0,186	0,155	-0,263	520,531	87,393
CPT2	0,002	0,166	0,437	0,269	0,334	0,313	229,18	67,827
ZBTB38	0,002	0,167	0,437	0,065	0,079	0,281	1015,096	72,18
BAG6	0,002	0,166	0,437	0,178	0,208	0,224	678,994	129,508
ZW10	0,001	0,166	0,437	0,253	0,291	0,2	624,794	171,418
SPPL2A	0,001	0,166	0,437	0,863	0,73	-0,242	192,508	151,499
ZNF414	0,003	0,166	0,437	0,3	0,39	0,381	135,617	45,873
VASP	0,001	0,166	0,437	0,27	0,301	0,16	997,103	283,816
URB1	0,002	0,166	0,437	0,083	0,07	-0,247	1245,787	95,081
SAMM50	0,001	0,166	0,437	0,421	0,363	-0,214	404,594	157,55
TSPAN6	0,002	0,166	0,437	0,699	0,568	-0,298	152,857	93,502
SLC25A36	0,003	0,167	0,438	0,091	0,113	0,311	567,482	58,104
HADH	0,001	0,167	0,438	0,44	0,377	-0,224	356,789	145,548
CYFIP1	0,001	0,167	0,438	0,206	0,182	-0,177	1123,61	218,471
SEMA7A	0,001	0,167	0,438	0,312	0,357	0,191	645,036	211,693
DHX8	0,002	0,167	0,438	0,061	0,073	0,264	1174,683	79,011
WDR18	0,001	0,167	0,438	0,485	0,433	-0,164	665,559	305,165
HMGN4	0,002	0,168	0,439	0,273	0,219	-0,316	256,106	62,722
ATP6V1D	0,001	0,168	0,439	0,354	0,402	0,184	570,91	215,873
DPH2	0,001	0,168	0,44	0,183	0,158	-0,215	799,405	136,774
RPL34	0,001	0,168	0,44	1,96	2,244	0,195	188,834	395,147
FOXP4	0,002	0,168	0,44	0,12	0,14	0,226	891,02	115,785
TCTN3	0,001	0,169	0,44	0,312	0,267	-0,227	447,036	128,984

PYGL	0,001	0,168	0,44	0,253	0,275	0,119	2313,739	604,979
TMCO3	0,002	0,169	0,441	0,169	0,198	0,229	647,812	117,874
ZFYVE26	0,002	0,169	0,441	0,111	0,091	-0,297	637,011	64,813
UBE2Z	0,001	0,169	0,441	0,16	0,18	0,166	1431,243	243,459
RANBP1	0,001	0,169	0,441	0,399	0,447	0,166	673,361	286,255
TP53BP2	0,002	0,17	0,442	0,097	0,08	-0,274	883,534	79,158
SCCPDH	0,002	0,17	0,442	0,459	0,388	-0,241	292,22	123,389
GFM2	0,002	0,17	0,442	0,206	0,173	-0,252	506,354	95,552
EXOC5	0,002	0,17	0,442	0,11	0,094	-0,223	1151,185	117,047
GCFC2	0,002	0,17	0,443	0,131	0,157	0,259	605,683	87,148
PTX3	0,001	0,171	0,444	0,793	0,893	0,172	383,782	323,911
TSPAN5	0,003	0,171	0,444	0,175	0,139	-0,336	325,796	50,849
SIGMAR1	0,001	0,171	0,444	0,164	0,181	0,145	1965,31	336,508
C11orf58	0,001	0,171	0,444	0,43	0,483	0,166	625,547	285,927
PVRL1	0,003	0,171	0,444	0,104	0,081	-0,371	449,819	42,399
ATF1	0,002	0,171	0,444	0,296	0,356	0,264	288,706	94,673
EXOSC6	0,002	0,171	0,444	1,073	0,868	-0,307	101,143	97,124
COX7B	0,002	0,171	0,444	1,009	1,27	0,332	78,368	88,914
DFNA5	0,001	0,172	0,445	0,283	0,243	-0,22	545,746	145,912
RAB18	0,001	0,172	0,445	0,214	0,246	0,203	673,491	154,976
ABLIM1	0,002	0,172	0,445	0,068	0,058	-0,234	1581,435	99,146
NADSYN1	0,002	0,172	0,445	0,238	0,198	-0,269	406,375	89,735
FAM57A	0,002	0,172	0,445	0,186	0,155	-0,26	524,106	89,971
GCAT	0,002	0,172	0,445	0,491	0,405	-0,277	204,959	91,172
TTK	0,002	0,173	0,446	0,123	0,144	0,226	853,401	114,317
TUSC3	0,002	0,173	0,446	0,379	0,314	-0,268	265,698	91,777
EGFL7	0,003	0,173	0,446	0,269	0,344	0,356	167,182	50,182
NEK9	0,002	0,172	0,446	0,121	0,147	0,285	527,458	70,438
CCNK	0,001	0,173	0,446	0,191	0,215	0,17	1123,475	227,893
GNL3	0,001	0,173	0,447	0,149	0,168	0,172	1416,245	221,822
FAM162A	0,002	0,174	0,447	0,561	0,671	0,258	189,186	115,942
PPP3CA	0,001	0,174	0,447	0,147	0,126	-0,221	898,354	123,202

MTIF3	0,003	0,174	0,447	0,308	0,245	-0,332	206,427	55,725
DNAJA3	0,001	0,174	0,447	0,194	0,172	-0,18	1091,645	200,192
TM4SF1	0,001	0,174	0,448	0,573	0,519	-0,143	783,961	429,142
SLC43A3	0,001	0,174	0,448	0,433	0,388	-0,159	741,058	302,343
KEAP1	0,001	0,174	0,448	0,264	0,292	0,148	1235,34	343,266
FAM120B	0,002	0,175	0,449	0,137	0,163	0,251	622,284	92,277
LARS2	0,002	0,175	0,45	0,095	0,116	0,288	626,667	66,815
RAB20	0,002	0,176	0,45	0,451	0,369	-0,292	194,126	79,404
NAA30	0,003	0,176	0,45	0,155	0,124	-0,328	371,281	51,099
H3F3B	0,001	0,176	0,45	0,303	0,279	-0,117	2035,673	592,903
WDR83OS	0,002	0,176	0,45	0,244	0,204	-0,26	403,098	90,466
SS18L1	0,003	0,176	0,45	0,164	0,129	-0,347	316,555	46,033
RPS4X	0,001	0,175	0,45	0,301	0,322	0,098	4216,329	1311,815
ARPC2	0,001	0,177	0,452	0,277	0,298	0,108	2631,807	753,554
STK11IP	0,002	0,177	0,452	0,287	0,35	0,286	244,724	77,377
MCUR1	0,002	0,177	0,452	0,125	0,105	-0,257	726,476	82,614
CDCA7L	0,001	0,177	0,452	0,169	0,193	0,187	996,292	179,432
TOMM7	0,001	0,177	0,452	1,48	1,248	-0,246	132,61	181,728
NIPSNAP3A	0,002	0,177	0,452	0,669	0,545	-0,295	146,839	90,161
CPNE2	0,003	0,177	0,452	0,162	0,131	-0,307	412,106	60,504
PPP1R12A	0,002	0,178	0,453	0,066	0,078	0,241	1295,438	93,84
BCAS2	0,001	0,178	0,454	0,387	0,443	0,197	438,325	181,996
NDUFS2	0,001	0,178	0,454	0,566	0,514	-0,137	905,52	490,61
ATP5I	0,002	0,178	0,454	0,81	1,025	0,34	82,114	74,547
ENSG00000269893.2	0,001	0,178	0,454	3,068	2,596	-0,241	102,65	287,356
TFPI2	0,001	0,179	0,454	0,593	0,678	0,194	366,018	228,498
DHRS1	0,004	0,178	0,454	0,407	0,303	-0,427	99,803	35,895
TRIM25	0,001	0,178	0,454	0,113	0,101	-0,168	2098,174	225,465
SYAP1	0,001	0,179	0,454	0,291	0,338	0,215	444,101	140,754
NIT1	0,002	0,179	0,455	0,633	0,518	-0,289	157,028	91,38
ARL6IP5	0,001	0,179	0,455	0,565	0,506	-0,161	579,059	311,023
SLC35G2	0,002	0,179	0,455	0,537	0,654	0,286	155,693	91,332

TXNDC15	0,002	0,179	0,455	0,421	0,355	-0,244	288,801	111,173
C11orf82	0,002	0,179	0,455	0,138	0,164	0,256	563,769	85,585
PPFIBP1	0,002	0,179	0,455	0,121	0,146	0,273	559,474	74,319
RNMTL1	0,001	0,179	0,455	0,32	0,279	-0,197	561,786	167,559
DDX10	0,003	0,18	0,456	0,074	0,091	0,306	669,004	55,348
CCDC85B	0,002	0,18	0,457	0,198	0,167	-0,243	536,143	97,875
IST1	0,002	0,18	0,457	0,106	0,127	0,266	653,8	77,054
KLHDC2	0,002	0,181	0,458	0,332	0,268	-0,308	215,126	63,923
TSR1	0,001	0,181	0,458	0,141	0,155	0,137	2438,997	361,883
SHMT1	0,001	0,181	0,459	0,32	0,282	-0,181	664,819	199,259
AK2	0,001	0,182	0,46	0,149	0,129	-0,207	969,688	134,325
RBM24	0,002	0,182	0,46	0,227	0,189	-0,262	406,778	84,742
MRPL43	0,001	0,182	0,46	0,515	0,447	-0,202	368,21	177,493
E2F8	0,002	0,182	0,46	0,141	0,17	0,274	476,578	74,845
USP15	0,002	0,182	0,46	0,168	0,196	0,222	643,345	117,891
GMFB	0,001	0,182	0,46	0,145	0,128	-0,179	1347,234	183,242
AHNAK2	0,002	0,182	0,46	0,09	0,105	0,217	1240,643	119,916
ACSF2	0,004	0,182	0,46	0,283	0,212	-0,415	142,404	35,938
RPRD1B	0,002	0,182	0,46	0,143	0,167	0,223	728,01	113,316
CHUK	0,002	0,183	0,461	0,168	0,141	-0,257	544,522	83,995
MGST1	0,001	0,183	0,461	0,722	0,67	-0,107	1552,011	1087,318
TOM1	0,002	0,183	0,461	0,289	0,242	-0,255	354,803	94,684
TXNDC9	0,001	0,185	0,462	0,396	0,455	0,201	397,084	169,902
RAB3GAP1	0,002	0,185	0,462	0,156	0,134	-0,222	778,159	113,154
AGFG1	0,001	0,185	0,462	0,292	0,264	-0,149	1117,706	309,945
RAD54L2	0,003	0,184	0,462	0,074	0,059	-0,328	713,179	47,378
SLC25A4	0,002	0,184	0,462	0,429	0,508	0,244	252,273	116,575
SERPINB1	0,001	0,184	0,462	0,562	0,49	-0,197	364,256	192,111
ARPC1B	0,001	0,184	0,462	0,426	0,371	-0,202	419,211	166,89
TMUB1	0,003	0,184	0,462	0,136	0,168	0,303	409,055	60,909
VCPIP1	0,002	0,183	0,462	0,132	0,158	0,259	559,52	82,19
TROAP	0,001	0,184	0,462	0,191	0,168	-0,184	977,834	175,776

NHLRC3	0,003	0,184	0,462	0,368	0,285	-0,366	140,284	45,997
FAM210A	0,002	0,184	0,462	0,213	0,253	0,249	403,607	94,414
MVB12A	0,001	0,184	0,462	0,668	0,577	-0,209	281,802	174,94
CECR5	0,002	0,184	0,462	0,301	0,259	-0,218	469,789	131,473
OST4	0,002	0,186	0,463	0,723	0,6	-0,269	156,386	103,132
PNO1	0,001	0,186	0,463	0,24	0,273	0,188	680,745	175,705
C5orf15	0,001	0,185	0,463	0,312	0,274	-0,187	631,488	184,33
PMPCA	0,001	0,185	0,463	0,286	0,249	-0,2	586,637	157,469
RTKN2	0,003	0,185	0,463	0,309	0,242	-0,353	170,029	46,315
CD151	0,001	0,186	0,463	0,736	0,684	-0,107	1398,738	997,795
CSTF3	0,002	0,185	0,463	0,297	0,253	-0,231	416,388	114,51
C11orf68	0,001	0,185	0,463	0,25	0,219	-0,196	701,653	166,152
TMBIM6	0,001	0,186	0,463	0,404	0,38	-0,088	4544,374	1774,863
TRIM13	0,002	0,186	0,463	0,239	0,293	0,295	251,905	66,856
TMX3	0,002	0,185	0,463	0,278	0,332	0,26	295,767	90,944
KDELR3	0,002	0,185	0,463	0,467	0,397	-0,237	275,741	117,996
LPP	0,002	0,187	0,464	0,1	0,084	-0,249	920,546	84,951
FGFRL1	0,003	0,187	0,464	0,051	0,065	0,331	789,568	45,082
TMA16	0,003	0,186	0,464	0,424	0,337	-0,331	150,548	56,416
TMEM248	0,001	0,187	0,464	0,209	0,186	-0,171	1045,848	206,097
ESYT1	0,001	0,187	0,464	0,182	0,199	0,13	2140,801	407,013
NCLN	0,001	0,187	0,464	0,216	0,198	-0,126	2116,074	438,637
IRF3	0,002	0,186	0,464	0,255	0,217	-0,232	470,139	111,766
RAB9A	0,003	0,186	0,464	0,482	0,368	-0,39	99,271	42,206
SRPX	0,002	0,187	0,464	0,478	0,576	0,269	209,941	106,776
ALG13	0,002	0,187	0,464	0,226	0,269	0,25	380,287	93,677
KCNAB2	0,002	0,188	0,465	0,079	0,092	0,231	1155,345	97,732
ADAM15	0,001	0,188	0,465	0,172	0,157	-0,138	2061,826	340,954
NDUFAF2	0,003	0,187	0,465	0,357	0,452	0,342	132,433	53,47
DPYSL2	0,001	0,188	0,465	0,185	0,164	-0,175	1113,898	192,561
SLC25A22	0,001	0,188	0,465	0,206	0,178	-0,212	683,4	131,961
SLC36A4	0,003	0,188	0,465	0,174	0,141	-0,308	354,989	55,731

MBD6	0,003	0,187	0,465	0,069	0,085	0,305	726,595	55,08
DIABLO	0,004	0,188	0,465	2,421	3,596	0,571	16,825	48,844
EID2	0,002	0,188	0,465	1,039	0,865	-0,264	129,073	122,517
NELFCD	0,001	0,188	0,465	0,286	0,258	-0,147	1209,445	328,535
SLC50A1	0,002	0,189	0,466	0,402	0,476	0,245	249,804	108,909
GTF2H1	0,002	0,189	0,466	0,132	0,112	-0,235	806,102	98,275
TRIAP1	0,002	0,189	0,466	0,489	0,396	-0,305	157,215	68,652
SHKBP1	0,002	0,189	0,466	0,224	0,193	-0,212	627,128	130,839
ZNF574	0,002	0,189	0,466	0,283	0,347	0,293	224,813	69,916
ZMPSTE24	0,001	0,189	0,467	0,463	0,499	0,109	1629,804	789,338
SMG5	0,001	0,189	0,467	0,06	0,052	-0,196	2457,603	137,33
HPS6	0,002	0,19	0,467	0,303	0,253	-0,263	310,777	87,048
RIN3	0,002	0,19	0,467	0,093	0,112	0,271	692,982	70,138
SUPT4H1	0,002	0,189	0,467	0,203	0,174	-0,223	601,038	112,691
HSD17B10	0,002	0,189	0,467	0,868	0,709	-0,292	116,076	91,515
UBLCP1	0,001	0,19	0,468	0,306	0,348	0,187	556,293	183,448
SEL1L	0,001	0,19	0,468	0,259	0,285	0,14	1246,054	340,8
TOP2A	0,001	0,191	0,468	0,066	0,073	0,133	5314,539	369,797
PNPT1	0,001	0,192	0,469	0,189	0,219	0,215	597,923	121,866
DGUOK	0,001	0,191	0,469	0,662	0,6	-0,141	669,268	421,197
DBR1	0,003	0,191	0,469	0,352	0,284	-0,308	197,378	61,556
EXOC4	0,003	0,191	0,469	0,153	0,125	-0,297	429,421	59,916
ENSG00000257790.1	0,002	0,191	0,469	1,653	2,203	0,414	35,405	68,899
C19orf60	0,001	0,191	0,469	0,928	0,784	-0,244	162,046	139,637
MCM3AP	0,003	0,191	0,469	0,075	0,06	-0,326	681,876	45,532
DNAJC11	0,001	0,192	0,47	0,202	0,176	-0,204	726,04	136,759
PSMD4	0,001	0,193	0,47	0,516	0,464	-0,154	647,383	316,799
PSMD14	0,001	0,192	0,47	0,36	0,395	0,135	1086,375	409,448
CUTA	0,002	0,193	0,47	0,426	0,505	0,246	235,68	108,727
CBX3	0,001	0,192	0,47	0,211	0,241	0,194	685,964	154,53
AKR1B1	0,001	0,193	0,47	0,388	0,345	-0,166	657,758	239,58
BNIP3L	0,001	0,192	0,47	0,352	0,314	-0,166	713,451	235,404

ZNF703	0,002	0,193	0,47	0,294	0,248	-0,246	358,678	97,695
SDCBP	0,001	0,192	0,47	0,333	0,303	-0,136	1168,558	369,32
SKA3	0,001	0,192	0,47	0,18	0,161	-0,155	1475,41	250,75
FARP1	0,002	0,193	0,47	0,063	0,053	-0,267	1191,235	69,003
PML	0,002	0,193	0,47	0,264	0,226	-0,221	502,668	124,437
DDOST	0,001	0,194	0,472	0,566	0,61	0,108	1574,335	927,341
AAED1	0,002	0,194	0,472	0,408	0,33	-0,309	170,796	62,715
MAP4K4	0,001	0,195	0,473	0,05	0,056	0,19	2710,09	145,177
OARD1	0,003	0,195	0,473	0,252	0,317	0,333	177,315	50,342
ATP6V1C1	0,001	0,195	0,473	0,221	0,25	0,18	770,128	182,944
EXOC6	0,003	0,195	0,473	0,277	0,35	0,335	162,207	51,051
WEE1	0,002	0,195	0,473	0,177	0,145	-0,292	377,052	60,542
VPS26B	0,003	0,195	0,473	0,086	0,068	-0,345	528,017	40,597
POLR1D	0,001	0,195	0,473	0,31	0,348	0,166	712,729	233,877
RABGGTA	0,003	0,195	0,473	0,235	0,294	0,324	207,565	53,692
MORF4L1	0,002	0,194	0,473	0,181	0,21	0,213	632,764	124,258
GPS1	0,001	0,194	0,473	0,394	0,429	0,122	1347,864	554,898
FBL	0,001	0,195	0,473	0,31	0,338	0,128	1369,604	443,926
MAPRE1	0,001	0,195	0,473	0,207	0,189	-0,126	2222,448	437,903
CABIN1	0,002	0,194	0,473	0,088	0,107	0,282	643,181	62,866
NKRF	0,002	0,195	0,473	0,102	0,123	0,279	569,897	64,223
WAPAL	0,001	0,196	0,474	0,096	0,108	0,178	1617,401	165,975
PANX1	0,002	0,195	0,474	0,168	0,203	0,281	361,369	67,479
NABP2	0,002	0,196	0,474	0,238	0,285	0,258	322,295	84,178
ZFAND6	0,002	0,196	0,474	0,399	0,484	0,279	181,07	79,753
PSMD7	0,001	0,196	0,474	0,285	0,252	-0,179	698,886	186,69
NDUFB5	0,001	0,197	0,475	1,317	1,167	-0,174	263,987	326,704
SDC4	0,001	0,196	0,475	0,463	0,415	-0,159	699,267	300,045
PMS1	0,003	0,197	0,476	0,148	0,184	0,316	309,076	51,213
NCBP2	0,002	0,197	0,476	0,164	0,14	-0,228	663,15	100,68
MCFD2	0,002	0,198	0,477	0,071	0,083	0,233	1188,649	90,903
DNAJB11	0,001	0,198	0,477	0,279	0,312	0,165	768,608	226,947

ITPR3	0,002	0,198	0,477	0,063	0,073	0,211	1713,492	114,73
MRPL18	0,001	0,198	0,477	0,65	0,57	-0,188	338,352	204,775
CERCAM	0,002	0,198	0,477	0,205	0,17	-0,271	401,651	76,706
TYSND1	0,002	0,198	0,477	0,351	0,302	-0,218	388,843	127,314
NFKB2	0,002	0,198	0,477	0,098	0,118	0,267	651,506	70,207
FEN1	0,001	0,198	0,477	0,281	0,306	0,122	1628,982	478,533
RBM26	0,002	0,198	0,477	0,078	0,091	0,224	1229,562	102,949
TIPIN	0,002	0,198	0,477	0,355	0,431	0,28	195,577	78,042
QTRTD1	0,002	0,199	0,478	0,168	0,138	-0,277	436,356	66,676
RNF40	0,002	0,199	0,478	0,084	0,097	0,21	1273,469	116,788
SYNGR2	0,001	0,199	0,478	0,461	0,414	-0,156	651,397	285,947
WARS2	0,003	0,2	0,479	0,28	0,227	-0,301	236,611	59,585
CPSF3	0,002	0,2	0,479	0,136	0,162	0,246	571,69	85,512
RBM27	0,001	0,2	0,479	0,159	0,179	0,172	1115,389	189,326
SUN1	0,001	0,199	0,479	0,18	0,201	0,161	1168,404	222,716
POLM	0,001	0,201	0,479	0,2	0,23	0,199	661,658	141,018
TRIM14	0,002	0,2	0,479	0,068	0,056	-0,28	990,932	61,758
MRPL50	0,002	0,2	0,479	0,632	0,538	-0,232	216,085	126,168
NUP37	0,002	0,2	0,479	0,491	0,584	0,249	196,663	105,789
CTDSPL2	0,002	0,2	0,479	0,1	0,12	0,271	610,032	68,011
GLCE	0,002	0,2	0,479	0,121	0,147	0,28	473,323	63,503
HELZ	0,002	0,2	0,479	0,07	0,083	0,251	993,749	76,081
LDOC1L	0,002	0,2	0,479	0,076	0,09	0,24	1011,72	83,967
ZZZ3	0,001	0,201	0,48	0,09	0,104	0,201	1271,736	124,061
IVNS1ABP	0,003	0,201	0,48	0,09	0,111	0,309	500,723	50,004
ZXDC	0,003	0,201	0,48	0,242	0,192	-0,334	215,389	46,398
ECI1	0,001	0,201	0,48	0,9	0,777	-0,213	206,7	172,91
FAM64A	0,001	0,201	0,48	0,346	0,39	0,175	553,838	205,092
TMEM256	0,001	0,201	0,48	1,25	1,054	-0,245	127,532	146,982
PCNA	0,001	0,201	0,48	0,726	0,67	-0,116	964,846	670,53
CHRNB1	0,001	0,202	0,481	0,248	0,278	0,161	903,789	236,698
RERE	0,002	0,202	0,482	0,094	0,113	0,269	660,023	67,581

RPS12	0,001	0,202	0,482	0,675	0,605	-0,158	475,187	304,266
AGPS	0,001	0,203	0,483	0,18	0,162	-0,149	1521,158	259,381
MBNL1	0,001	0,203	0,483	0,106	0,117	0,142	2503,039	278,681
ARHGAP18	0,002	0,203	0,483	0,189	0,16	-0,242	502,809	86,741
UBE2R2	0,002	0,203	0,483	0,145	0,169	0,217	714,877	111,149
MIDN	0,001	0,204	0,483	0,199	0,226	0,183	771,032	164,402
COMMD7	0,002	0,203	0,483	0,171	0,204	0,254	433,843	80,457
MSN	0,001	0,203	0,483	0,04	0,044	0,109	12562,839	525,341
MSH6	0,002	0,204	0,484	0,077	0,067	-0,209	1536,25	109,799
USP39	0,001	0,205	0,484	0,169	0,188	0,15	1404,543	251,971
NME6	0,002	0,204	0,484	0,447	0,371	-0,271	197,905	79,903
PARL	0,002	0,205	0,484	0,284	0,336	0,243	303,346	94,46
DNAJC21	0,002	0,205	0,484	0,114	0,095	-0,264	659,861	68,294
AGPAT5	0,002	0,205	0,484	0,359	0,295	-0,281	218,284	70,614
CHMP5	0,001	0,204	0,484	0,455	0,515	0,178	427,632	207,266
FAM168A	0,002	0,204	0,484	0,095	0,079	-0,267	759,171	65,505
ALDH2	0,004	0,204	0,484	0,335	0,255	-0,395	132,004	40,504
BLMH	0,002	0,204	0,484	0,227	0,264	0,218	466,548	114,402
XYLT2	0,002	0,205	0,484	0,129	0,156	0,277	456,425	64,092
CUEDC1	0,003	0,205	0,484	0,076	0,061	-0,327	632,429	43,138
ENSG00000163597.10	0,001	0,204	0,484	1,115	0,973	-0,196	214,234	222,886
SLC38A10	0,001	0,205	0,484	0,168	0,192	0,193	793,45	142,204
ILF3	0,001	0,205	0,484	0,1	0,107	0,103	7584,941	785,46
PMVK	0,002	0,205	0,485	0,432	0,517	0,26	191,365	90,588
PHF19	0,001	0,205	0,485	0,207	0,189	-0,134	1765,331	348,917
COPS7A	0,001	0,206	0,485	0,218	0,247	0,178	765,48	179,11
PGP	0,001	0,205	0,485	0,493	0,424	-0,219	281,628	128,212
EIF4ENIF1	0,003	0,206	0,485	0,128	0,157	0,293	395,356	56,306
IRF2BP2	0,003	0,207	0,486	0,173	0,14	-0,306	340,329	53,67
CHAC2	0,003	0,206	0,486	0,427	0,543	0,346	104,583	50,296
HIF1AN	0,002	0,207	0,486	0,119	0,101	-0,237	789,831	87,268
ZNF511	0,002	0,207	0,486	0,185	0,225	0,285	307,711	62,864

DCAKD	0,002	0,207	0,486	0,348	0,286	-0,286	216,407	69,206
YES1	0,002	0,207	0,486	0,082	0,069	-0,239	1084,183	81,165
NMI	0,003	0,208	0,487	0,475	0,366	-0,375	98,945	41,926
DCTN2	0,001	0,207	0,487	0,363	0,403	0,148	771,738	295,827
PTPRA	0,002	0,207	0,487	0,117	0,138	0,241	651,875	83,132
GTF2B	0,001	0,208	0,488	0,584	0,681	0,221	220,319	140,395
FREM2	0,003	0,208	0,488	0,235	0,29	0,303	215,389	57,002
TUBGCP4	0,002	0,208	0,488	0,161	0,134	-0,262	487,471	71,271
IDH2	0,001	0,208	0,488	0,332	0,287	-0,209	418,117	128,384
CIAPIN1	0,001	0,208	0,488	0,308	0,357	0,21	384,79	128,177
YTHDF1	0,001	0,208	0,488	0,174	0,196	0,172	980,612	181,943
WDR12	0,002	0,209	0,489	0,145	0,17	0,221	641,687	101,067
IDE	0,001	0,209	0,489	0,128	0,112	-0,198	1048,061	125,398
HNRNPA0	0,001	0,209	0,49	0,429	0,397	-0,109	1624,087	669,665
SPRED1	0,003	0,209	0,49	0,115	0,093	-0,307	472,633	49,269
TMEM39B	0,002	0,21	0,491	0,409	0,499	0,286	161,096	72,443
TOMM20	0,002	0,21	0,491	0,14	0,123	-0,194	1033,37	135,799
ZC3HAV1	0,001	0,21	0,491	0,08	0,091	0,198	1424,656	121,927
PCNX	0,002	0,21	0,491	0,075	0,064	-0,233	1248	86,378
DYNLL2	0,001	0,21	0,491	0,142	0,124	-0,203	899,222	118,831
C1orf112	0,002	0,211	0,492	0,393	0,328	-0,262	224,991	80,36
AP1S1	0,001	0,211	0,492	0,396	0,443	0,162	568,86	238,355
MDFIC	0,003	0,211	0,492	0,129	0,103	-0,334	359,114	41,762
CTR9	0,002	0,211	0,492	0,064	0,076	0,256	992,703	69,097
YIF1A	0,001	0,211	0,492	0,47	0,525	0,16	536,387	265,091
MDM2	0,002	0,211	0,492	0,177	0,151	-0,223	611,64	99,324
LITAF	0,002	0,211	0,492	0,304	0,258	-0,239	344,905	97,891
GATAD2A	0,001	0,211	0,492	0,137	0,15	0,131	2441,923	348,897
NDUFAB1	0,001	0,212	0,493	1,137	1,272	0,162	295,449	354,77
FAM192A	0,002	0,212	0,493	0,145	0,122	-0,253	553,949	73,226
IGFBP4	0,001	0,212	0,493	0,184	0,168	-0,126	2220,451	388,148
POLRMT	0,003	0,212	0,493	0,097	0,077	-0,322	507,188	44,32

MUL1	0,002	0,213	0,494	0,314	0,263	-0,257	277,008	79,765
WNT7B	0,003	0,212	0,494	0,093	0,074	-0,332	514,28	43,943
MRTO4	0,001	0,213	0,495	0,238	0,267	0,165	806,299	203,266
THADA	0,002	0,213	0,495	0,194	0,165	-0,237	490,118	87,687
RPF2	0,002	0,213	0,495	0,142	0,168	0,248	502,441	78,127
GPALPP1	0,002	0,213	0,495	0,152	0,182	0,259	430,818	72,672
NARFL	0,003	0,214	0,495	0,313	0,251	-0,315	184,586	51,794
UBALD2	0,001	0,213	0,495	0,451	0,523	0,214	279,885	134,829
CEP250	0,001	0,214	0,495	0,049	0,056	0,201	2255,846	116,132
GEMIN6	0,002	0,214	0,496	0,56	0,679	0,278	133,698	82,175
MRPL13	0,001	0,214	0,496	0,689	0,761	0,143	516,056	375,518
GMEB1	0,003	0,215	0,498	0,179	0,222	0,315	244,162	48,737
MAP2K3	0,001	0,215	0,498	0,262	0,232	-0,174	731,697	181,412
AMOTL2	0,001	0,216	0,499	0,126	0,138	0,13	2723,647	358,401
PAQR3	0,003	0,216	0,499	0,143	0,117	-0,289	425,503	55,609
MYO10	0,001	0,216	0,499	0,08	0,09	0,168	2055,012	174,49
ZNF277	0,003	0,216	0,499	0,282	0,352	0,32	160,381	51,316
NCAPG2	0,001	0,217	0,499	0,171	0,152	-0,169	1116,451	179,471
PRSS23	0,001	0,216	0,499	0,383	0,358	-0,096	2623,351	974,639
SEC11A	0,001	0,216	0,499	0,437	0,493	0,172	445,118	207,852
DNASE2	0,002	0,216	0,499	0,441	0,515	0,224	264,544	124,162
NXT1	0,002	0,216	0,499	0,425	0,507	0,255	192,226	89,43
RASA1	0,002	0,217	0,5	0,139	0,161	0,214	699,847	105,628
IQGAP3	0,001	0,217	0,501	0,096	0,085	-0,172	1831,808	166,738
TBCC	0,002	0,217	0,501	0,465	0,389	-0,26	196,037	83,402
NHP2L1	0,001	0,217	0,501	0,729	0,679	-0,103	1213,094	854,637
SZRD1	0,001	0,219	0,502	0,077	0,088	0,194	1495,575	122,563
SMARCC2	0,002	0,218	0,502	0,076	0,089	0,214	1195,8	98,069
PRIM1	0,002	0,219	0,502	0,324	0,377	0,217	334,528	118,775
DOHH	0,001	0,218	0,502	0,485	0,557	0,198	301,845	157,053
PIGK	0,001	0,219	0,503	0,322	0,36	0,161	651,944	222,441
TTF2	0,002	0,219	0,503	0,092	0,108	0,222	932,505	92,971

LCOR	0,003	0,219	0,503	0,075	0,093	0,298	584,607	49,03
ACSL5	0,001	0,219	0,503	0,151	0,17	0,174	1027,515	164,735
OXSM	0,003	0,22	0,504	0,575	0,452	-0,346	94,171	47,694
STK17A	0,002	0,22	0,504	0,202	0,234	0,21	520,2	114,422
SLC27A4	0,001	0,22	0,504	0,215	0,189	-0,182	763,4	154,642
PFN1	0,001	0,22	0,504	0,251	0,265	0,074	14253,533	3671,556
XPNPEP3	0,003	0,22	0,504	0,192	0,155	-0,311	277,702	47,614
ERAP1	0,002	0,221	0,505	0,209	0,251	0,264	304,712	69,99
NRF1	0,003	0,221	0,505	0,227	0,281	0,309	202,297	51,151
ARHGAP1	0,001	0,221	0,505	0,27	0,242	-0,155	917,724	236,711
SSRP1	0,001	0,221	0,505	0,12	0,129	0,103	4929,146	614,465
CASC4	0,003	0,221	0,505	0,107	0,088	-0,289	539,801	52,202
TGFB1I1	0,001	0,221	0,505	0,465	0,406	-0,195	361,391	158,527
CHMP4B	0,001	0,221	0,505	0,206	0,231	0,166	907,717	196,921
RNF113A	0,003	0,221	0,505	0,293	0,239	-0,297	215,579	57,821
DNAJC8	0,002	0,222	0,506	0,142	0,169	0,25	476,273	73,927
NABP1	0,002	0,223	0,506	0,083	0,098	0,248	783,687	70,815
SMARCAL1	0,003	0,222	0,506	0,16	0,196	0,292	313,58	55,052
CDC40	0,002	0,222	0,506	0,316	0,373	0,24	265,763	91,785
RFC2	0,003	0,222	0,506	0,291	0,362	0,313	159,187	52,075
TNPO3	0,001	0,222	0,506	0,192	0,214	0,153	1098,689	223,325
SQLE	0,002	0,222	0,506	0,207	0,244	0,242	367,727	83,204
C11orf24	0,001	0,222	0,506	0,216	0,245	0,183	712,43	161,678
ERGIC2	0,002	0,222	0,506	0,196	0,23	0,229	437,059	93,863
RANBP3	0,001	0,223	0,506	0,254	0,28	0,139	1104,259	294,514
XRCC6	0,001	0,223	0,506	0,191	0,177	-0,108	3076,897	564,521
PSMD10	0,001	0,222	0,506	0,459	0,402	-0,19	371,241	159,079
CCNA2	0,001	0,223	0,507	0,29	0,315	0,12	1468,89	444,618
GCNT1	0,005	0,223	0,507	0,189	0,145	-0,379	218,467	37,243
CHMP1B	0,002	0,224	0,507	0,427	0,365	-0,225	276,444	109,654
MKL1	0,003	0,223	0,507	0,181	0,222	0,292	284,101	56,241
SDC3	0,003	0,224	0,508	0,078	0,096	0,297	602,411	50,961

RFWD2	0,003	0,224	0,508	0,21	0,169	-0,316	247,219	46,383
ATF4	0,001	0,224	0,508	0,458	0,419	-0,131	874,233	383,654
DDX20	0,001	0,225	0,509	0,225	0,256	0,187	604,728	145,364
NUP133	0,002	0,225	0,509	0,163	0,142	-0,2	777,167	118,253
AHCTF1	0,003	0,225	0,509	0,07	0,086	0,303	591,324	45,991
PIKFYVE	0,002	0,225	0,509	0,093	0,079	-0,23	960,314	82,467
COA1	0,001	0,225	0,509	0,209	0,235	0,17	805,157	177,727
UBE3C	0,001	0,225	0,509	0,154	0,138	-0,161	1296,908	187,768
DUT	0,001	0,226	0,509	0,886	0,969	0,129	549,027	510,124
YIF1B	0,001	0,225	0,509	0,614	0,551	-0,155	463,806	269,604
CPNE1	0,001	0,225	0,509	0,251	0,232	-0,112	2019,108	489,883
SLCO4A1	0,002	0,225	0,509	0,344	0,297	-0,213	361,261	114,777
GNL3L	0,001	0,225	0,509	0,221	0,246	0,15	1004,778	234,291
KIF2C	0,001	0,227	0,511	0,097	0,106	0,128	3115,004	316,584
ACTR3	0,001	0,226	0,511	0,224	0,243	0,116	2015,747	471,813
TRNT1	0,003	0,227	0,511	0,301	0,243	-0,311	184,44	49,981
PDLIM1	0,001	0,226	0,511	0,376	0,339	-0,146	770,208	275,441
NOL3	0,002	0,226	0,511	0,398	0,48	0,27	174,889	75,744
DHX9	0,001	0,227	0,512	0,056	0,05	-0,161	3304,564	174,322
PHF3	0,001	0,227	0,512	0,082	0,094	0,184	1489,054	131,758
NAGLU	0,002	0,227	0,512	0,417	0,339	-0,297	163,694	62,97
CDC45	0,001	0,227	0,512	0,214	0,246	0,199	534,417	123,736
DNPH1	0,002	0,228	0,513	0,888	0,717	-0,309	87,723	71,194
SPATA20	0,003	0,228	0,513	0,099	0,081	-0,286	592,581	53,823
MED25	0,001	0,228	0,513	0,325	0,37	0,189	436,35	150,364
PFDN4	0,001	0,229	0,513	0,938	1,074	0,196	194,382	195,259
HCCS	0,001	0,228	0,513	0,411	0,472	0,199	324,153	142,537
ABHD5	0,002	0,229	0,514	0,223	0,191	-0,225	447,718	92,37
NCEH1	0,001	0,229	0,514	0,267	0,29	0,118	1603,81	447,549
RRS1	0,001	0,229	0,514	0,361	0,397	0,137	826,661	312,825
DPH7	0,002	0,23	0,514	0,229	0,195	-0,238	395,06	83,871
POLR2G	0,001	0,23	0,514	0,879	0,964	0,133	489,889	453,171

PTGES3	0,001	0,229	0,514	0,154	0,138	-0,152	1471,596	213,312
WARS	0,001	0,229	0,514	0,213	0,232	0,127	1592,847	355,326
KIAA0195	0,003	0,23	0,514	0,077	0,093	0,276	663,924	55,861
RHBDF2	0,002	0,229	0,514	0,154	0,127	-0,271	441,404	62,734
AP3D1	0,001	0,229	0,514	0,114	0,104	-0,127	3171,835	345,742
NDUFA1	0,001	0,23	0,514	2,016	2,239	0,152	257,616	542,431
MIER1	0,001	0,23	0,515	0,164	0,186	0,184	795,47	139,089
KLHL18	0,003	0,231	0,515	0,099	0,081	-0,29	548,873	49,245
DUSP1	0,001	0,231	0,515	0,368	0,329	-0,16	657,221	231,413
TRMT11	0,003	0,23	0,515	0,581	0,472	-0,298	120,363	62,372
PCID2	0,001	0,231	0,515	0,449	0,402	-0,16	535,477	228,526
SYNE2	0,002	0,231	0,515	0,075	0,087	0,205	1253,551	101,581
MAPK1	0,001	0,231	0,515	0,175	0,193	0,14	1397,635	257,362
IFT27	0,002	0,231	0,515	0,633	0,529	-0,259	149,242	86,059
RAMP1	0,003	0,232	0,516	0,425	0,536	0,335	107,827	50,158
FIGNL1	0,002	0,231	0,516	0,173	0,206	0,248	393,399	74,186
CUL5	0,002	0,231	0,516	0,163	0,189	0,214	569,202	101,028
GMFG	0,003	0,231	0,516	0,926	1,192	0,365	52,262	54,182
CBR1	0,002	0,231	0,516	0,429	0,37	-0,212	297,915	118,449
MTBP	0,002	0,233	0,517	0,215	0,25	0,221	418,1	97,86
MFAP1	0,002	0,232	0,517	0,164	0,136	-0,263	428,887	64,67
NUP88	0,001	0,232	0,517	0,181	0,195	0,108	2816,45	529,484
PPP2R1A	0,001	0,232	0,517	0,353	0,329	-0,101	1933,702	660,151
DBNL	0,001	0,233	0,518	0,197	0,219	0,154	1012,982	210,472
SORD	0,002	0,234	0,518	0,333	0,394	0,245	250,079	88,884
AATF	0,001	0,233	0,518	0,161	0,179	0,157	1127,326	192,479
ALKBH7	0,001	0,233	0,518	1,431	1,219	-0,232	115,898	153,847
SEPHS1	0,003	0,234	0,519	0,107	0,086	-0,317	426,574	41,016
TALDO1	0,001	0,234	0,519	0,751	0,706	-0,09	1667,702	1216,724
ABHD2	0,001	0,234	0,519	0,12	0,136	0,176	1153,128	148,016
IER2	0,003	0,234	0,519	0,182	0,148	-0,298	296,218	48,342
ERCC6L	0,002	0,234	0,519	0,094	0,109	0,22	882,766	89,763

ANAPC13	0,002	0,235	0,52	0,293	0,248	-0,239	307,363	83,278
DROSHA	0,002	0,235	0,52	0,067	0,077	0,195	1573,029	113,383
FASTK	0,003	0,235	0,52	0,105	0,085	-0,308	462,258	44,189
PNPLA6	0,001	0,235	0,52	0,138	0,122	-0,181	1068,939	139,368
TXN2	0,001	0,235	0,52	0,348	0,315	-0,143	804,011	265,957
NEGR1	0,003	0,236	0,521	0,141	0,175	0,315	287,59	44,383
RPAP2	0,003	0,236	0,521	0,171	0,14	-0,286	339,528	52,361
ENDOD1	0,002	0,236	0,521	0,155	0,186	0,265	384,684	64,214
MPST	0,001	0,236	0,521	0,357	0,323	-0,143	811,893	275,342
NONO	0,001	0,236	0,521	0,098	0,105	0,111	4424,135	448,911
PLXND1	0,002	0,236	0,522	0,096	0,112	0,214	915,274	94,859
COG5	0,002	0,236	0,522	0,235	0,273	0,215	412,337	103,587
ADPRHL2	0,002	0,237	0,523	0,354	0,3	-0,24	260,242	84,612
ARID2	0,002	0,238	0,523	0,138	0,163	0,239	504,531	75,829
C15orf39	0,002	0,238	0,523	0,224	0,266	0,248	332,826	79,707
SYNRG	0,003	0,237	0,523	0,16	0,195	0,287	296,866	53,081
ARHGEF12	0,002	0,239	0,525	0,034	0,03	-0,213	2789,738	89,292
KPNA6	0,002	0,239	0,526	0,072	0,062	-0,218	1278,014	85,781
GTF2E2	0,002	0,239	0,526	0,231	0,275	0,255	280,186	71,79
PPIF	0,001	0,239	0,526	0,361	0,336	-0,105	1701,457	594,927
UBXN1	0,001	0,24	0,526	0,366	0,404	0,144	713,99	274,792
C14orf1	0,002	0,24	0,526	0,307	0,263	-0,227	331,034	94,85
NME1	0,001	0,239	0,526	0,438	0,393	-0,156	558,327	232,039
TECR	0,001	0,239	0,526	0,594	0,646	0,121	749,466	464,076
CLASRP	0,003	0,239	0,526	0,134	0,108	-0,301	384,177	46,968
TMEM201	0,001	0,24	0,527	0,198	0,223	0,172	764,192	160,093
DPH5	0,002	0,241	0,527	0,658	0,552	-0,253	151,496	89,476
TPM3	0,001	0,24	0,527	0,12	0,131	0,118	3077,111	386,401
CDK6	0,002	0,241	0,527	0,059	0,067	0,193	1955,61	126,818
TRIM8	0,002	0,241	0,527	0,076	0,066	-0,202	1397,497	99,901
OSGEP	0,002	0,24	0,527	0,402	0,344	-0,222	271,107	100,789
TP53BP1	0,001	0,24	0,527	0,359	0,395	0,139	772,36	291,424

CCP110	0,003	0,24	0,527	0,095	0,079	-0,264	670,443	57,584
XPO6	0,001	0,24	0,527	0,135	0,122	-0,142	1809,753	232,401
USP10	0,002	0,24	0,527	0,083	0,07	-0,248	850,226	65,356
STARD8	0,003	0,241	0,527	0,115	0,095	-0,28	511,427	54,196
HDAC1	0,001	0,242	0,528	0,332	0,305	-0,125	1113,707	353,882
PEX13	0,003	0,242	0,528	0,237	0,197	-0,272	276,545	59,801
CISD2	0,003	0,241	0,528	0,529	0,654	0,305	99,57	59,184
STARD3NL	0,002	0,242	0,528	0,205	0,177	-0,212	520,329	99,83
COX16	0,002	0,242	0,528	0,842	0,72	-0,227	154,643	119,915
CALM2	0,001	0,243	0,529	0,408	0,382	-0,095	2117,057	834,066
UBE2E3	0,002	0,243	0,529	0,26	0,221	-0,229	368,47	89,325
MAPK14	0,001	0,244	0,529	0,174	0,155	-0,17	957,301	156,779
MTO1	0,002	0,243	0,529	0,187	0,157	-0,249	401,633	68,738
CD164	0,001	0,244	0,529	0,38	0,347	-0,131	901,828	324,952
CHCHD2	0,001	0,243	0,529	0,979	0,886	-0,143	388,316	361,135
ST7	0,003	0,243	0,529	0,282	0,228	-0,307	185,88	47,131
TMEM245	0,001	0,243	0,529	0,204	0,22	0,108	2329,086	492,452
UGCG	0,001	0,243	0,529	0,303	0,324	0,099	2167,419	677,877
ATG2A	0,001	0,242	0,529	0,217	0,193	-0,167	817,21	167,93
UCHL3	0,002	0,242	0,529	0,522	0,634	0,279	125,336	71,312
PCK2	0,002	0,243	0,529	0,232	0,279	0,265	252,027	64,328
CHD3	0,002	0,242	0,529	0,031	0,035	0,18	4007,63	129,718
CMPK1	0,001	0,244	0,53	0,117	0,129	0,142	1915,128	235,513
PSMB1	0,001	0,244	0,53	0,96	0,902	-0,09	1460,326	1359
TMEM123	0,001	0,244	0,53	0,157	0,168	0,095	4524,838	736,091
CCDC71	0,002	0,245	0,531	0,282	0,233	-0,273	235,549	60,773
DAB2	0,003	0,245	0,531	0,119	0,144	0,274	406,497	53,816
TRIP6	0,001	0,245	0,531	0,395	0,364	-0,119	1085,304	411,365
DRG1	0,001	0,245	0,531	0,334	0,305	-0,128	1015,002	323,615
TRMT2B	0,002	0,245	0,531	0,149	0,124	-0,259	449,625	61,115
MRPS5	0,001	0,246	0,532	0,19	0,214	0,171	771,615	155,457
DOCK2	0,002	0,245	0,532	0,163	0,196	0,264	340,446	60,629

NCBP1	0,001	0,246	0,532	0,193	0,174	-0,148	1173,898	213,922
TMUB2	0,002	0,246	0,532	0,229	0,265	0,206	432,539	106,755
HSPA13	0,001	0,246	0,532	0,472	0,515	0,125	785,395	389,504
ZMYM1	0,003	0,246	0,533	0,188	0,227	0,273	275,48	57,717
EPHX1	0,002	0,247	0,533	0,433	0,504	0,218	238,931	110,788
ITSN2	0,002	0,247	0,533	0,113	0,133	0,245	547,6	67,098
RAF1	0,001	0,247	0,533	0,114	0,103	-0,157	1687,444	183,444
NGLY1	0,002	0,247	0,533	0,165	0,198	0,261	337,812	61,379
FIBP	0,001	0,246	0,533	0,28	0,255	-0,135	1057,578	283,956
CDK17	0,003	0,247	0,533	0,138	0,167	0,275	351,655	53,757
HN1L	0,001	0,246	0,533	0,129	0,117	-0,151	1551,638	190,573
DONSON	0,003	0,246	0,533	0,168	0,204	0,276	296,244	55,396
EXTL2	0,002	0,248	0,534	0,384	0,327	-0,232	248,97	87,454
PPP3R1	0,002	0,248	0,534	0,093	0,11	0,253	621,389	63,994
OSBPL10	0,002	0,248	0,534	0,095	0,112	0,233	729,938	75,012
POLR1C	0,002	0,248	0,534	0,396	0,333	-0,253	209,169	76,864
SLC35B4	0,003	0,248	0,534	0,103	0,123	0,264	497,761	56,496
NXF1	0,002	0,248	0,534	0,11	0,096	-0,198	1024,229	105,527
SLC11A2	0,002	0,249	0,534	0,322	0,273	-0,236	279,484	81,995
RPLP0	0,001	0,248	0,534	0,094	0,101	0,115	3739,465	364,484
RRAS	0,003	0,249	0,534	0,368	0,296	-0,313	147,601	49,918
WIPI2	0,002	0,249	0,535	0,18	0,156	-0,206	596,732	99,932
ATP6V1B2	0,002	0,249	0,535	0,261	0,227	-0,197	486,225	118,747
SIRT1	0,002	0,25	0,535	0,209	0,244	0,22	396,052	90,353
NDUFS8	0,001	0,25	0,535	0,785	0,844	0,105	888,734	720,562
PHF23	0,001	0,249	0,535	0,247	0,223	-0,145	997,514	234,285
SNRPD1	0,001	0,249	0,535	0,805	0,715	-0,17	282,623	213,886
SBNO2	0,001	0,25	0,535	0,108	0,098	-0,143	2101,355	217,904
SAFB2	0,001	0,249	0,535	0,204	0,225	0,145	1081,657	231,398
NFS1	0,002	0,249	0,535	0,218	0,184	-0,245	354,211	71,347
KIAA0930	0,003	0,249	0,535	0,059	0,049	-0,266	1014,395	55,279
PARK7	0,001	0,251	0,537	0,601	0,646	0,104	1045,033	651,883

CDC20	0,001	0,251	0,537	0,338	0,32	-0,081	3450,886	1134,721
ECHDC1	0,001	0,251	0,537	0,345	0,388	0,172	457,243	168,292
MCM2	0,001	0,252	0,539	0,078	0,07	-0,165	2030,747	149,641
ARL3	0,002	0,252	0,539	0,436	0,373	-0,226	237,053	96,201
AEN	0,002	0,252	0,539	0,126	0,145	0,199	750,151	102,042
NAGS	0,002	0,252	0,539	0,217	0,259	0,252	289,692	68,23
CAMK2D	0,003	0,253	0,541	0,163	0,201	0,302	249,26	44,859
ERI1	0,003	0,253	0,541	0,159	0,195	0,294	263,08	47,159
TINAGL1	0,002	0,255	0,542	0,178	0,203	0,19	616,138	117,743
PSRC1	0,001	0,254	0,542	0,243	0,271	0,157	755,457	193,158
TIMM17A	0,001	0,254	0,542	0,746	0,677	-0,14	447,528	318,644
APPL1	0,001	0,255	0,542	0,153	0,136	-0,171	1003,799	145,078
ASB6	0,002	0,254	0,542	0,203	0,171	-0,247	361,973	67,507
BICC1	0,003	0,255	0,542	0,108	0,089	-0,289	467,754	46,21
SAMD8	0,002	0,255	0,542	0,126	0,108	-0,224	671,879	78,447
PAPOLA	0,001	0,255	0,542	0,106	0,098	-0,113	3784,388	383,622
EVI2B	0,002	0,254	0,542	0,226	0,264	0,226	348,96	86,735
GNA15	0,002	0,254	0,542	0,108	0,129	0,256	495,465	58,853
RPE	0,003	0,256	0,543	0,241	0,2	-0,268	263,428	57,66
HLTF	0,002	0,256	0,543	0,081	0,069	-0,246	816,603	60,879
TOP1MT	0,002	0,255	0,543	0,171	0,145	-0,237	455,469	71,497
APTX	0,002	0,256	0,543	0,237	0,201	-0,242	326,969	71,45
SLC22A18	0,004	0,256	0,543	0,794	0,6	-0,404	51,846	37,24
FZR1	0,001	0,255	0,543	0,147	0,164	0,157	1119,122	173,359
C1orf52	0,002	0,257	0,544	0,313	0,266	-0,235	283,604	83,09
RPUSD3	0,001	0,257	0,544	0,333	0,371	0,153	607,972	212,912
MATN2	0,002	0,257	0,544	0,246	0,283	0,203	403,198	106,742
RAB14	0,001	0,256	0,544	0,251	0,226	-0,151	926,46	223,642
TBC1D15	0,003	0,257	0,544	0,168	0,202	0,264	312,066	57,75
RPAP1	0,002	0,256	0,544	0,217	0,189	-0,201	519,299	105,659
CLN6	0,001	0,257	0,544	0,235	0,208	-0,18	609,723	135,044
CREBBP	0,001	0,257	0,544	0,175	0,191	0,13	1478,897	270,378

SLC25A11	0,001	0,257	0,544	0,469	0,433	-0,116	960,235	432,626
THBD	0,003	0,257	0,544	0,408	0,33	-0,306	131,227	48,153
SAMHD1	0,001	0,256	0,544	0,155	0,139	-0,157	1183,27	173,316
MBTPS2	0,002	0,257	0,544	0,303	0,259	-0,23	295,925	82,486
SLC16A2	0,003	0,257	0,544	0,227	0,273	0,266	245,481	60,523
RAB7A	0,001	0,258	0,546	0,228	0,245	0,104	2092,923	493,905
PHF15	0,003	0,259	0,546	0,074	0,062	-0,252	869,82	58,15
ASAP1	0,001	0,258	0,546	0,089	0,098	0,15	1986,424	185,366
UBALD1	0,003	0,259	0,546	0,229	0,284	0,31	174,883	44,146
POLD1	0,002	0,259	0,546	0,157	0,139	-0,182	840,843	124,062
TCP1	0,001	0,259	0,547	0,3	0,322	0,098	1967,823	612,271
PA2G4	0,002	0,259	0,547	0,085	0,097	0,186	1234,269	111,964
TUBB4B	0,001	0,26	0,548	0,394	0,412	0,065	7384,721	2969,313
NAGA	0,002	0,26	0,548	0,2	0,172	-0,22	451,463	83,693
GNG5	0,001	0,261	0,549	0,603	0,543	-0,151	428,393	245,883
FBXO28	0,001	0,261	0,549	0,212	0,188	-0,173	710,083	142,019
RPIA	0,002	0,261	0,549	0,29	0,342	0,239	257,91	79,895
FAF2	0,001	0,261	0,549	0,144	0,158	0,134	1575,338	237,203
TM7SF3	0,002	0,26	0,549	0,176	0,2	0,185	648,806	122,695
FBXW11	0,002	0,261	0,55	0,089	0,104	0,217	827,412	80,144
GTF2H5	0,002	0,261	0,55	0,795	0,967	0,282	86,007	74,665
CEP78	0,002	0,262	0,55	0,147	0,128	-0,197	732,483	100,637
NSMCE1	0,002	0,262	0,55	0,758	0,64	-0,244	130,224	90,6
AP2S1	0,001	0,262	0,55	0,652	0,705	0,113	762,016	515,882
GJA1	0,003	0,263	0,551	0,304	0,368	0,277	170,961	56,872
HHEX	0,003	0,263	0,552	0,251	0,306	0,289	180,47	49,858
GNB5	0,002	0,263	0,552	0,424	0,36	-0,235	212,839	83,574
AGL	0,002	0,264	0,553	0,13	0,154	0,249	431,48	61,518
SACM1L	0,001	0,263	0,553	0,233	0,208	-0,164	723,066	158,987
MYEOV	0,002	0,264	0,553	0,149	0,132	-0,181	868,941	120,914
LRRC8C	0,002	0,265	0,554	0,235	0,205	-0,195	512,24	113,449
CENPJ	0,003	0,265	0,554	0,08	0,096	0,267	569,744	50,202

SREBF1	0,001	0,265	0,554	0,174	0,19	0,128	1597,527	288,507
RNF213	0,001	0,265	0,554	0,103	0,093	-0,146	1995,731	197,09
KDM4A	0,002	0,265	0,555	0,155	0,134	-0,206	624,945	90,409
CHID1	0,001	0,265	0,555	0,414	0,374	-0,148	567,763	223,265
YIPF4	0,001	0,267	0,556	0,485	0,551	0,183	285,952	148,354
SMEK2	0,002	0,267	0,556	0,134	0,117	-0,191	843,648	104,963
SEMA4C	0,002	0,267	0,556	0,237	0,272	0,2	413,793	105,035
ACAA1	0,002	0,266	0,556	0,386	0,45	0,219	230,523	95,685
MRPS2	0,001	0,266	0,556	0,274	0,297	0,12	1232,669	350,066
MPI	0,002	0,267	0,556	0,367	0,312	-0,233	236,669	80,039
C16orf70	0,002	0,266	0,556	0,211	0,252	0,256	264,327	61,861
NPEPPS	0,003	0,266	0,556	0,185	0,154	-0,263	327,137	54,973
CDR2L	0,003	0,267	0,556	0,081	0,066	-0,293	565,588	41,85
RAD23A	0,001	0,266	0,556	0,209	0,225	0,104	2189,695	474,312
TMEM184B	0,001	0,266	0,556	0,131	0,118	-0,148	1485,269	186,089
ZNF326	0,002	0,268	0,557	0,122	0,103	-0,248	526,443	58,921
GPN1	0,002	0,267	0,557	0,257	0,222	-0,212	381,97	91,159
IPO8	0,001	0,268	0,557	0,141	0,16	0,179	816,713	123,806
CLIP1	0,002	0,268	0,557	0,051	0,044	-0,232	1403,853	66,768
FAM213B	0,003	0,268	0,558	0,131	0,161	0,294	301,842	43,417
TBC1D9B	0,002	0,269	0,558	0,071	0,062	-0,196	1425,007	95,269
DENND1A	0,002	0,269	0,558	0,101	0,119	0,234	613,67	66,998
PTPN9	0,003	0,268	0,558	0,093	0,112	0,267	491,325	50,206
LCMT1	0,003	0,269	0,558	0,377	0,458	0,278	137,49	57,857
ARMC6	0,001	0,268	0,558	0,314	0,288	-0,125	1023,902	308,02
C19orf33	0,002	0,268	0,558	1,508	1,236	-0,288	62,244	85,476
PPM1F	0,001	0,269	0,558	0,124	0,11	-0,173	1101,552	128,778
SMAP2	0,002	0,27	0,559	0,147	0,167	0,186	727,91	113,654
SIKE1	0,001	0,269	0,559	0,157	0,14	-0,171	900,938	133,322
TMEM71	0,002	0,27	0,559	0,249	0,295	0,243	255,775	69,088
ITGA5	0,001	0,27	0,559	0,271	0,294	0,116	1294,423	364,772
ZIC2	0,002	0,27	0,559	0,206	0,237	0,201	450,458	100,798

MYBL2	0,002	0,27	0,559	0,072	0,083	0,201	1140,032	88,938
ARHGEF2	0,001	0,27	0,56	0,08	0,091	0,172	1503,606	127,482
WIPF1	0,002	0,27	0,56	0,153	0,131	-0,226	530,451	76,293
WDR41	0,002	0,27	0,56	0,21	0,177	-0,245	335,096	64,857
YWHAQ	0,001	0,272	0,561	0,443	0,418	-0,083	2562,832	1101,818
TPRKB	0,001	0,271	0,561	0,743	0,824	0,148	340,276	267,617
PEX6	0,002	0,272	0,561	0,235	0,199	-0,236	325,285	70,523
AVL9	0,002	0,271	0,561	0,086	0,101	0,23	714,665	66,949
ARMC1	0,001	0,271	0,561	0,287	0,256	-0,165	582,054	156,39
KAT7	0,002	0,272	0,561	0,151	0,129	-0,223	537,427	74,539
DGCR2	0,002	0,272	0,561	0,122	0,141	0,213	640,58	83,463
RANBP9	0,001	0,272	0,562	0,213	0,238	0,158	734,371	165,418
RPS21	0,001	0,273	0,563	3,269	3,475	0,088	687,027	2317,207
SLC35F5	0,002	0,273	0,564	0,304	0,266	-0,19	401,487	113,791
WDR33	0,001	0,274	0,564	0,171	0,151	-0,173	805,871	129,886
BCCIP	0,001	0,274	0,564	0,368	0,339	-0,116	1067,003	376,037
DDX24	0,001	0,274	0,564	0,06	0,067	0,141	2996,01	189,571
TMOD3	0,001	0,273	0,564	0,245	0,224	-0,131	1149,084	268,87
LONRF3	0,002	0,274	0,564	0,262	0,22	-0,248	267,56	64,568
PSMB7	0,001	0,274	0,565	0,768	0,814	0,082	1518,975	1200,784
HNRNPL	0,001	0,275	0,565	0,295	0,273	-0,114	1305,186	370,254
SLBP	0,001	0,276	0,566	0,338	0,37	0,131	775,077	275,469
HSPA14	0,001	0,275	0,566	0,35	0,314	-0,157	547,044	180,842
NIN	0,002	0,276	0,566	0,03	0,036	0,231	1923,941	63,184
RHOC	0,001	0,276	0,567	0,17	0,152	-0,157	1054,818	171,224
LAPTM4A	0,001	0,276	0,567	0,711	0,765	0,106	767,48	568,68
FSTL3	0,003	0,276	0,567	0,141	0,115	-0,29	374,374	49,529
PUS1	0,002	0,277	0,569	0,211	0,248	0,239	295,532	67,546
ATAD3A	0,002	0,278	0,57	0,177	0,156	-0,186	659,26	109,882
AP3B1	0,002	0,278	0,57	0,09	0,078	-0,207	998,427	83,697
ARFGEF1	0,002	0,278	0,57	0,104	0,121	0,222	637,759	71,383
DOCK1	0,002	0,278	0,57	0,109	0,096	-0,184	1062,314	108,9

SLC15A4	0,002	0,278	0,57	0,476	0,414	-0,201	253,178	112,976
ENSG00000196756.7	0,002	0,278	0,57	0,315	0,361	0,199	313,043	105,995
TRIM11	0,003	0,279	0,571	0,127	0,153	0,266	362,265	50,102
RAVER1	0,001	0,279	0,571	0,319	0,296	-0,11	1306,122	401,995
TMEM51	0,003	0,281	0,572	0,275	0,227	-0,279	196,888	49,485
ZNF687	0,002	0,28	0,572	0,242	0,277	0,198	402,089	105,98
PSMD1	0,001	0,28	0,572	0,244	0,228	-0,098	2437,279	573,51
SMAP1	0,002	0,28	0,572	0,336	0,396	0,239	199,484	72,578
IGFBP3	0,001	0,28	0,572	0,319	0,346	0,117	1082,639	364,06
LRRC8A	0,001	0,28	0,572	0,186	0,169	-0,141	1176,823	207,799
RSU1	0,001	0,28	0,572	0,294	0,264	-0,155	632,569	176,273
INPP5F	0,003	0,28	0,572	0,136	0,114	-0,25	446,163	55,179
MAP4K2	0,004	0,28	0,572	0,119	0,097	-0,301	356,356	38,95
IL18	0,003	0,28	0,572	0,332	0,41	0,304	143,81	51,033
LTA4H	0,001	0,281	0,572	0,214	0,192	-0,154	837,043	169,806
MON1B	0,002	0,281	0,572	0,114	0,134	0,232	536,983	65,995
ZNF335	0,002	0,28	0,572	0,169	0,147	-0,199	603,915	95,597
S100A11	0,001	0,282	0,573	0,73	0,773	0,082	1880,115	1406,63
NAGK	0,001	0,282	0,573	0,358	0,316	-0,183	373,227	126,243
PPARD	0,003	0,281	0,573	0,071	0,058	-0,285	629,117	40,81
TMTC3	0,002	0,282	0,573	0,26	0,294	0,178	467,185	130,101
WDHD1	0,002	0,281	0,573	0,111	0,097	-0,192	908,499	93,85
SREBF2	0,001	0,282	0,573	0,101	0,091	-0,155	1671,618	162,561
COQ3	0,003	0,283	0,574	0,826	0,662	-0,32	68,393	49,361
PRKAR1B	0,003	0,282	0,574	0,197	0,163	-0,27	281,022	51,038
CLTA	0,001	0,282	0,574	0,66	0,704	0,093	1143,872	779,576
LARP4B	0,001	0,283	0,574	0,13	0,144	0,149	1272,463	174,827
CASP7	0,003	0,283	0,574	0,194	0,163	-0,252	317,164	56,673
BRF1	0,002	0,283	0,574	0,128	0,15	0,233	473,92	65,393
LACTB	0,002	0,282	0,574	0,294	0,343	0,221	258,375	82,622
USP22	0,001	0,283	0,574	0,169	0,16	-0,077	6408,108	1056,661
COASY	0,001	0,283	0,574	0,113	0,126	0,165	1097,691	130,751

COX7A2L	0,001	0,284	0,575	0,419	0,376	-0,154	476,645	189,186
RETSAT	0,001	0,284	0,575	0,256	0,287	0,164	559,694	151,686
SBNO1	0,001	0,284	0,575	0,13	0,143	0,137	1516,707	208,051
FKBP8	0,001	0,284	0,575	0,231	0,214	-0,111	1660,153	371,488
ATXN10	0,001	0,284	0,575	0,394	0,369	-0,096	1560,719	594,22
RAB2A	0,001	0,285	0,576	0,34	0,373	0,13	754,737	270,025
AKAP11	0,001	0,285	0,576	0,123	0,135	0,139	1558,312	200,945
XBP1	0,002	0,285	0,576	0,146	0,167	0,191	626,922	98,253
TCEAL8	0,001	0,285	0,576	0,415	0,368	-0,173	369,824	144,627
ITM2C	0,001	0,286	0,577	0,253	0,231	-0,128	1079,1	259,286
SP2	0,002	0,286	0,577	0,203	0,231	0,183	527,305	113,604
NIPSNAP1	0,003	0,286	0,577	0,238	0,285	0,26	218,583	56,37
SKP1	0,001	0,286	0,578	0,246	0,275	0,162	586,471	152,498
MGST3	0,001	0,287	0,579	1,331	1,188	-0,164	195,681	246,596
NNT	0,002	0,287	0,579	0,115	0,13	0,173	980,581	119,861
ID1	0,001	0,287	0,579	0,52	0,578	0,154	367,28	202,403
GNB1L	0,003	0,287	0,579	0,524	0,42	-0,318	88,825	41,897
YBX1	0,001	0,288	0,58	0,106	0,097	-0,13	2193,261	222,433
SHC1	0,001	0,288	0,58	0,078	0,085	0,133	2510,93	204,264
KDSR	0,002	0,288	0,58	0,243	0,282	0,214	314,327	82,471
GTF2F1	0,001	0,288	0,58	0,082	0,091	0,145	1935,859	166,411
DPYD	0,002	0,288	0,581	0,219	0,192	-0,189	504,621	103,342
ADSS	0,001	0,289	0,582	0,326	0,368	0,174	392,851	135,882
KIDINS220	0,002	0,29	0,582	0,064	0,074	0,214	1051,354	72,465
IDH1	0,002	0,289	0,582	0,343	0,298	-0,204	313,402	101,714
CSNK1G3	0,002	0,29	0,582	0,118	0,1	-0,229	596,399	64,815
KLF4	0,002	0,29	0,582	0,216	0,185	-0,228	343,338	68,69
ME3	0,002	0,29	0,582	0,274	0,234	-0,232	270,722	68,37
GLOD4	0,001	0,289	0,582	0,314	0,344	0,131	778,828	257,109
CCDC97	0,003	0,29	0,582	0,176	0,146	-0,271	291,184	46,695
CCNC	0,002	0,291	0,583	0,176	0,202	0,198	486,448	93,071
MYOF	0,001	0,291	0,583	0,054	0,057	0,092	9773,684	543,439

PLBD2	0,001	0,29	0,583	0,42	0,464	0,145	508,945	223,15
SIRT6	0,002	0,29	0,583	0,384	0,331	-0,215	255,248	92,431
PRPF38A	0,002	0,292	0,585	0,135	0,118	-0,192	741,892	92,961
TMEM206	0,002	0,293	0,585	0,363	0,426	0,229	195,294	76,584
LRP12	0,002	0,292	0,585	0,214	0,252	0,236	277,958	65,15
RPP30	0,001	0,293	0,585	0,373	0,413	0,149	489,215	192,839
HMBS	0,002	0,292	0,585	0,178	0,204	0,202	459,55	87,608
VAC14	0,001	0,292	0,585	0,205	0,186	-0,14	1023,598	199,25
U2AF2	0,001	0,292	0,585	0,283	0,301	0,086	2535,524	741,493
ABL2	0,001	0,293	0,586	0,078	0,086	0,136	2412,488	197,258
PEX3	0,002	0,294	0,586	0,264	0,31	0,234	236,198	67,942
BIN3	0,004	0,293	0,586	0,584	0,467	-0,325	76,951	40,522
FOXA2	0,003	0,293	0,586	0,232	0,281	0,277	192,092	48,318
GLMN	0,002	0,295	0,587	0,381	0,45	0,241	166,664	69,801
TMEM167B	0,003	0,294	0,587	0,189	0,158	-0,251	313,335	54,274
CDCA7	0,002	0,294	0,587	0,133	0,151	0,184	741,339	106,752
RNF41	0,002	0,294	0,587	0,169	0,144	-0,235	394,174	61,715
LONP2	0,002	0,294	0,587	0,109	0,096	-0,19	914,748	93,412
C19orf53	0,001	0,294	0,587	0,385	0,422	0,129	676,943	271,632
RBBP5	0,002	0,296	0,588	0,157	0,184	0,228	382,568	65,326
ANKRD39	0,004	0,296	0,588	0,512	0,407	-0,33	81,733	37,847
HIBCH	0,003	0,296	0,588	0,439	0,359	-0,29	116,873	46,405
GLRX	0,002	0,296	0,588	0,447	0,526	0,235	154,495	75,552
PPP2CA	0,001	0,296	0,588	0,271	0,253	-0,1	1725,645	450,742
ARAP3	0,003	0,296	0,588	0,099	0,082	-0,274	479,599	44,133
INTS5	0,001	0,296	0,588	0,525	0,469	-0,164	331,886	165,346
TAF6L	0,003	0,295	0,588	0,297	0,357	0,268	159,63	52,357
MRPS31	0,002	0,295	0,588	0,268	0,311	0,212	287,546	83,232
METTL17	0,002	0,295	0,588	0,456	0,402	-0,181	295,99	126,491
GMPR2	0,001	0,296	0,588	0,332	0,297	-0,159	501,54	157,229
TDP1	0,002	0,295	0,588	0,154	0,135	-0,19	661,37	95,39
E2F4	0,001	0,295	0,588	0,119	0,107	-0,158	1258,03	141,846

CPOX	0,002	0,297	0,589	0,393	0,335	-0,232	205,785	73,248
C5orf24	0,003	0,297	0,589	0,113	0,095	-0,258	469,602	49,206
ILF2	0,001	0,298	0,59	0,236	0,25	0,081	3118,262	758,011
COQ4	0,001	0,298	0,59	0,379	0,34	-0,155	478,214	172,095
PPP4C	0,001	0,297	0,59	0,416	0,452	0,121	736,292	318,706
ODC1	0,001	0,298	0,591	0,15	0,136	-0,142	1225,642	174,167
C9orf114	0,002	0,299	0,591	0,154	0,177	0,196	537,719	88,679
CDC73	0,001	0,299	0,592	0,2	0,222	0,148	811,174	170,471
GEMIN5	0,001	0,299	0,592	0,215	0,197	-0,128	1158,523	238,052
TM9SF3	0,001	0,299	0,592	0,325	0,345	0,086	2129,987	716,637
CDIPT	0,002	0,3	0,592	0,25	0,22	-0,188	437,321	102,306
CSNK2A2	0,001	0,3	0,592	0,105	0,117	0,156	1274,003	142,927
CNOT1	0,001	0,3	0,592	0,123	0,131	0,09	4761,179	607,676
DTD1	0,001	0,299	0,592	0,446	0,403	-0,146	474,528	201,07
ZSWIM1	0,002	0,3	0,592	0,243	0,287	0,24	238,168	62,416
BCL2L13	0,002	0,3	0,592	0,147	0,169	0,205	504,184	79,82
SH3BP1	0,003	0,299	0,592	0,209	0,251	0,263	226,141	51,213
RAB1A	0,001	0,3	0,593	0,51	0,479	-0,092	1315,738	648,783
NR3C1	0,001	0,301	0,593	0,13	0,118	-0,14	1497,216	184,652
UTRN	0,001	0,301	0,593	0,1	0,111	0,144	1590,505	168,525
DCTN3	0,001	0,301	0,593	0,791	0,719	-0,139	351,927	265,699
IAH1	0,002	0,302	0,594	0,329	0,39	0,243	180,376	64,235
TNPO1	0,001	0,302	0,594	0,167	0,157	-0,093	3007,271	486,543
NDFIP1	0,001	0,302	0,594	0,577	0,524	-0,14	431,323	237,301
URM1	0,001	0,302	0,594	0,288	0,317	0,139	681,35	205,463
RRM1	0,001	0,302	0,594	0,129	0,138	0,104	2980,951	397,42
GPR137	0,002	0,302	0,594	0,127	0,148	0,228	468,3	63,533
YBX3	0,001	0,301	0,594	0,147	0,157	0,099	2811,901	425,43
ANKRD11	0,002	0,301	0,594	0,022	0,026	0,217	2639,215	62,616
RLIM	0,002	0,301	0,594	0,081	0,071	-0,176	1343,043	101,954
NT5DC2	0,002	0,303	0,595	0,14	0,124	-0,178	785,469	102,994
TRAF2	0,002	0,303	0,595	0,19	0,217	0,192	465,883	94,546

KANSL2	0,003	0,303	0,595	0,211	0,252	0,259	218,128	50,758
PAN3	0,002	0,303	0,595	0,14	0,16	0,194	594,618	90,172
KLF16	0,002	0,303	0,595	0,231	0,262	0,187	427,16	104,263
RFXANK	0,002	0,303	0,595	0,406	0,465	0,193	248,213	107,555
C12orf65	0,003	0,304	0,596	0,189	0,157	-0,268	261,581	44,842
TANGO6	0,003	0,304	0,596	0,196	0,166	-0,241	313,613	56,112
EPG5	0,003	0,304	0,596	0,134	0,112	-0,261	374,283	45,808
SSX2IP	0,003	0,305	0,597	0,077	0,091	0,228	705,882	59,36
RPL26L1	0,002	0,305	0,597	0,684	0,811	0,246	106,825	78,557
PLEKHA2	0,003	0,304	0,597	0,135	0,114	-0,241	436,29	54,25
FOXRED1	0,002	0,305	0,597	0,231	0,265	0,195	371,797	92,452
GALK1	0,002	0,305	0,597	0,59	0,515	-0,195	204,602	113,319
EIF3L	0,001	0,305	0,597	0,414	0,389	-0,089	1688,004	675,741
YARS	0,001	0,306	0,598	0,252	0,27	0,101	1653,691	431,134
B4GALT2	0,003	0,306	0,598	0,056	0,046	-0,287	736,205	38,026
MYSM1	0,002	0,306	0,598	0,069	0,06	-0,219	982,445	62,887
UGP2	0,001	0,306	0,598	0,232	0,252	0,12	1136,776	276,771
ZNF142	0,003	0,306	0,598	0,07	0,083	0,245	668,214	50,612
HIGD1A	0,002	0,307	0,598	0,255	0,296	0,215	272,584	75,415
RNF4	0,002	0,306	0,598	0,145	0,165	0,183	638,629	99,183
ACSL1	0,002	0,306	0,598	0,199	0,172	-0,208	417,747	77,835
DIMT1	0,001	0,307	0,598	0,281	0,314	0,161	485,337	143,941
SUFU	0,003	0,307	0,598	0,182	0,219	0,266	231,199	46,144
TPM1	0,001	0,306	0,598	0,221	0,242	0,133	913,701	212,251
POLG	0,002	0,307	0,598	0,122	0,107	-0,184	835,306	95,508
PRKD2	0,003	0,307	0,598	0,157	0,131	-0,261	331,273	48,203
SCMH1	0,003	0,307	0,599	0,063	0,052	-0,27	707,857	40,966
C1orf123	0,002	0,308	0,599	0,367	0,425	0,213	213,775	84,104
NT5DC3	0,003	0,308	0,599	0,142	0,119	-0,254	369,713	47,747
CPNE8	0,004	0,308	0,6	0,145	0,119	-0,286	292,736	38,712
C19orf55	0,003	0,309	0,6	0,177	0,215	0,283	214,243	41,241
PATZ1	0,002	0,308	0,6	0,137	0,119	-0,196	659,676	84,246

JAGN1	0,001	0,31	0,601	0,646	0,711	0,137	376,001	254,822
SENP6	0,003	0,309	0,601	0,056	0,066	0,234	878,257	53,969
EPHB4	0,003	0,31	0,601	0,108	0,091	-0,247	500,556	49,435
RRP12	0,001	0,31	0,601	0,095	0,086	-0,148	1696,66	154,521
SBF2	0,003	0,31	0,601	0,086	0,072	-0,255	586,198	46,36
SNX1	0,001	0,309	0,601	0,236	0,261	0,144	697,222	174,05
STRA13	0,001	0,31	0,601	0,393	0,351	-0,166	382,398	142,763
ZNF724P	0,003	0,309	0,601	0,265	0,316	0,251	189,66	55,012
TTC38	0,001	0,309	0,601	0,276	0,298	0,11	1153,676	332,095
PRDX4	0,001	0,309	0,601	0,892	0,818	-0,125	399,933	340,47
MAGEF1	0,002	0,311	0,602	0,308	0,261	-0,237	216,267	61,407
TMEM33	0,001	0,311	0,602	0,518	0,553	0,094	1065,598	573,066
MED13L	0,002	0,311	0,602	0,075	0,066	-0,183	1304,561	91,328
CEP192	0,002	0,311	0,602	0,151	0,134	-0,179	695,891	98,816
MANBAL	0,002	0,311	0,602	0,154	0,176	0,2	506,678	82,689
OSER1	0,001	0,31	0,602	0,463	0,415	-0,157	373,379	162,95
APOL6	0,002	0,311	0,602	0,099	0,116	0,23	532,505	57,584
CAD	0,001	0,312	0,603	0,098	0,106	0,115	2539,448	258,327
CYB5R1	0,002	0,313	0,604	0,47	0,409	-0,201	216,272	94,75
FUS	0,001	0,313	0,604	0,176	0,186	0,074	5284,934	957,047
PCGF5	0,003	0,314	0,605	0,084	0,071	-0,238	669,662	51,298
ITPK1	0,002	0,313	0,605	0,064	0,075	0,229	816,214	55,861
SLC7A5	0,001	0,313	0,605	0,136	0,129	-0,07	11326,387	1499,448
TMEM63A	0,002	0,314	0,606	0,186	0,215	0,21	370,362	73,763
MAML1	0,002	0,315	0,606	0,103	0,092	-0,172	1070,818	104,281
PM20D2	0,001	0,314	0,606	0,199	0,178	-0,157	730,675	136,998
ZNF143	0,002	0,315	0,606	0,335	0,292	-0,198	286,043	89,448
ELK3	0,002	0,314	0,606	0,073	0,083	0,189	1149,905	88,112
ANKLE2	0,002	0,314	0,606	0,139	0,123	-0,173	803,943	105,016
MRPL39	0,001	0,314	0,606	0,899	0,799	-0,17	197,732	166,528
HELLS	0,001	0,315	0,607	0,158	0,142	-0,159	852,501	126,703
TMED3	0,001	0,315	0,607	0,338	0,306	-0,14	611,489	197,67

RRP7A	0,001	0,315	0,607	0,217	0,194	-0,158	672,427	138,259
TRIT1	0,002	0,316	0,608	0,257	0,291	0,177	399,711	109,841
COA6	0,003	0,316	0,608	0,625	0,518	-0,271	95,175	53,866
KANSL3	0,001	0,316	0,608	0,13	0,117	-0,154	1077,799	132,447
ZMYND19	0,003	0,316	0,608	0,145	0,171	0,233	361,074	56,981
KIF18B	0,001	0,316	0,608	0,134	0,146	0,122	1628,332	228,134
SERINC3	0,001	0,316	0,608	0,348	0,326	-0,095	1561,825	529,067
ZYG11B	0,002	0,318	0,609	0,147	0,128	-0,201	555,68	76,505
ATL2	0,001	0,317	0,609	0,234	0,262	0,168	482,417	120,125
ENSG00000188971.4	0,002	0,318	0,609	0,061	0,071	0,204	1042,084	69,265
POLR1A	0,001	0,318	0,609	0,069	0,075	0,128	2617,505	187,796
COQ2	0,002	0,318	0,609	0,464	0,403	-0,201	214,611	93,125
MELK	0,001	0,317	0,609	0,17	0,186	0,13	1164,869	208,271
SSNA1	0,002	0,317	0,609	0,242	0,273	0,177	426,582	109,237
GINS3	0,003	0,317	0,609	0,273	0,231	-0,244	222,902	55,199
THOP1	0,001	0,318	0,609	0,251	0,234	-0,103	1493,286	362,051
ZFX	0,002	0,318	0,609	0,252	0,22	-0,199	351,371	82,488
RER1	0,001	0,321	0,611	0,298	0,273	-0,125	824,173	234,8
TSN	0,001	0,32	0,611	0,298	0,274	-0,122	887,426	253,249
FARP2	0,003	0,32	0,611	0,096	0,082	-0,218	686,829	60,881
PAICS	0,001	0,32	0,611	0,135	0,144	0,088	3746,615	522,286
PLAC8	0,001	0,319	0,611	1,209	1,293	0,097	568,844	713,219
TRAM2	0,001	0,32	0,611	0,073	0,078	0,091	5669,804	429,748
SMC2	0,001	0,32	0,611	0,094	0,103	0,132	1909,593	188,416
RDH11	0,001	0,32	0,611	0,149	0,164	0,138	1095,457	171,068
SNW1	0,001	0,32	0,611	0,138	0,152	0,138	1176,32	170,721
RAB11A	0,001	0,32	0,611	0,428	0,396	-0,114	777,352	320,488
NFATC2IP	0,003	0,319	0,611	0,1	0,084	-0,249	507,851	46,148
CDT1	0,001	0,32	0,611	0,301	0,275	-0,13	762,558	219,648
UBE2L3	0,002	0,32	0,611	0,064	0,075	0,211	919,104	63,618
RBMS1	0,002	0,321	0,612	0,152	0,133	-0,2	531,491	75,689
TPGS2	0,002	0,321	0,612	0,2	0,176	-0,187	479,23	89,539

ARMC9	0,003	0,321	0,613	0,225	0,265	0,236	239,107	57,896
ATG16L1	0,003	0,322	0,613	0,187	0,159	-0,232	325,212	55,842
ATP5J	0,001	0,322	0,613	0,466	0,514	0,143	404,498	198,008
MBD4	0,003	0,324	0,615	0,161	0,188	0,229	328,625	57,325
SFXN1	0,001	0,323	0,615	0,299	0,279	-0,099	1453,426	418,269
BZW2	0,001	0,324	0,615	0,28	0,308	0,139	626,267	183,795
CPNE3	0,002	0,324	0,615	0,102	0,09	-0,177	993,636	95,155
DSCC1	0,002	0,323	0,615	0,294	0,331	0,168	387,248	121,53
REEP3	0,002	0,323	0,615	0,139	0,124	-0,161	912,323	119,985
CHKA	0,001	0,324	0,615	0,419	0,467	0,157	348,837	154,529
NIPA2	0,001	0,323	0,615	0,296	0,268	-0,145	585,503	164,602
WIZ	0,002	0,324	0,615	0,087	0,098	0,166	1149,311	106,091
PAK4	0,002	0,324	0,615	0,106	0,093	-0,186	858,641	85,231
TP53RK	0,002	0,323	0,615	0,248	0,287	0,206	287,531	77,101
LSM5	0,001	0,325	0,616	0,624	0,557	-0,165	263,477	153,111
PTOV1	0,001	0,325	0,616	0,502	0,447	-0,17	279,394	132,66
CMSS1	0,001	0,325	0,617	0,357	0,32	-0,16	406,723	137,645
TSPAN4	0,001	0,326	0,617	0,812	0,759	-0,098	770,046	608,576
KHDRBS1	0,001	0,329	0,618	0,152	0,141	-0,105	2217,995	324,508
SMYD2	0,002	0,329	0,618	0,22	0,195	-0,175	489,301	101,124
WDR43	0,001	0,328	0,618	0,229	0,246	0,104	1485,299	352,685
CREB1	0,001	0,327	0,618	0,164	0,183	0,157	733,067	127,299
MFN1	0,002	0,326	0,618	0,216	0,188	-0,2	385,458	77,313
ARSJ	0,003	0,328	0,618	0,111	0,133	0,255	370,293	44,452
ATOX1	0,001	0,328	0,618	1,209	1,327	0,134	262,04	330,034
MAPK9	0,002	0,326	0,618	0,109	0,095	-0,208	648,712	65,891
TNS3	0,001	0,327	0,618	0,094	0,085	-0,149	1498,518	133,373
ZNF655	0,003	0,327	0,618	0,085	0,073	-0,222	712,689	55,689
SORBS3	0,002	0,327	0,618	0,107	0,124	0,212	559,649	64,437
NFX1	0,003	0,328	0,618	0,072	0,061	-0,236	736,232	49,177
RFK	0,002	0,327	0,618	0,171	0,152	-0,164	711,006	114,386
FAM129B	0,001	0,326	0,618	0,181	0,172	-0,072	5059,678	894,952

UBE2D1	0,002	0,328	0,618	0,361	0,311	-0,215	215,307	71,92
PRPF19	0,001	0,327	0,618	0,292	0,308	0,077	2679,58	803,176
RPAP3	0,002	0,328	0,618	0,175	0,2	0,189	456,344	86,03
EAPP	0,002	0,327	0,618	0,499	0,587	0,235	125,322	68,292
SGPP1	0,002	0,327	0,618	0,485	0,424	-0,194	212,576	96,59
POMT2	0,003	0,326	0,618	0,116	0,097	-0,256	414,886	44,406
BCAR1	0,001	0,329	0,618	0,214	0,228	0,088	2312,85	511,662
KARS	0,001	0,328	0,618	0,183	0,194	0,09	2592,672	489,808
SENP3	0,002	0,326	0,618	0,146	0,129	-0,181	664,511	90,711
TRIB3	0,003	0,329	0,618	0,188	0,222	0,245	248,214	50,524
PIM3	0,001	0,327	0,618	0,269	0,251	-0,1	1449,225	377,736
AGRN	0,001	0,331	0,619	0,166	0,177	0,088	3028,212	515,776
CDCA8	0,001	0,331	0,619	0,151	0,161	0,095	2645,97	413,851
GADD45A	0,001	0,329	0,619	0,291	0,323	0,15	502,196	153,526
PDCD10	0,001	0,331	0,619	0,431	0,482	0,161	312,327	143,282
CWC27	0,002	0,33	0,619	0,174	0,2	0,202	390,992	73,049
CCNG1	0,002	0,33	0,619	0,162	0,141	-0,198	494,396	74,303
GRK6	0,003	0,33	0,619	0,112	0,096	-0,226	528,221	54,69
TAB2	0,002	0,331	0,619	0,058	0,066	0,19	1218,562	75,529
URGCP	0,002	0,329	0,619	0,157	0,18	0,199	456,564	76,326
PFKM	0,001	0,33	0,619	0,214	0,194	-0,14	829,233	169,346
ENSG00000006062.9	0,001	0,33	0,619	0,155	0,173	0,158	788,834	131,54
IFT52	0,002	0,33	0,619	0,332	0,284	-0,222	215,154	65,564
CD40	0,002	0,331	0,619	0,515	0,586	0,187	199,698	109,224
SNRNP200	0,001	0,331	0,62	0,08	0,076	-0,082	6917,533	540,52
MFI2	0,005	0,331	0,62	0,236	0,191	-0,301	183,924	40,09
MTX1	0,003	0,332	0,621	0,763	0,632	-0,273	77,629	54,3
OCIAD2	0,001	0,332	0,621	0,966	0,87	-0,151	230,048	211,781
RUNX2	0,002	0,333	0,621	0,156	0,182	0,216	367,394	61,995
SNX3	0,001	0,333	0,621	0,24	0,22	-0,127	905,626	208,248
SND1	0,001	0,333	0,621	0,116	0,107	-0,115	2210,889	245,987
KHDRBS3	0,003	0,333	0,621	0,221	0,265	0,26	188,62	45,235

CAMSAP1	0,003	0,332	0,621	0,061	0,052	-0,225	925,316	52,457
EHMT1	0,001	0,333	0,621	0,13	0,116	-0,157	974,879	120,489
FADS1	0,001	0,332	0,621	0,176	0,165	-0,091	2625,323	447,954
GOLGA3	0,001	0,332	0,621	0,12	0,108	-0,152	1128,576	128,701
SCFD1	0,002	0,333	0,621	0,309	0,276	-0,163	454,82	130,499
C16orf72	0,002	0,333	0,621	0,233	0,264	0,179	395,959	98,587
CDH4	0,001	0,333	0,621	0,115	0,126	0,135	1384,214	165,872
PRPF6	0,002	0,333	0,621	0,071	0,064	-0,157	1752,12	118,382
PUM2	0,001	0,334	0,622	0,188	0,173	-0,119	1278,349	228,778
SATB2	0,002	0,334	0,622	0,115	0,103	-0,16	1024,865	111,978
NARF	0,002	0,334	0,622	0,239	0,209	-0,197	348,592	77,853
EVI5L	0,003	0,334	0,622	0,084	0,072	-0,229	667,129	51,946
ECT2	0,001	0,335	0,623	0,124	0,134	0,117	1734,629	224,438
DISP2	0,002	0,335	0,623	0,362	0,319	-0,18	301,202	102,233
SMYD4	0,002	0,335	0,623	0,183	0,16	-0,189	479,577	81,975
WRAP53	0,001	0,335	0,623	0,463	0,498	0,107	732,315	352,303
CLIC4	0,001	0,337	0,624	0,154	0,143	-0,112	1807,83	267,542
PLEC	0,002	0,336	0,624	0,063	0,072	0,195	1054,506	70,523
VIM	0,001	0,337	0,624	0,116	0,121	0,062	16637,831	1968,688
ELP4	0,002	0,337	0,624	0,375	0,327	-0,197	240,632	84,016
CLPB	0,002	0,337	0,624	0,199	0,224	0,174	475,302	100,11
RNF111	0,003	0,336	0,624	0,1	0,083	-0,256	452,184	41,632
BTBD1	0,001	0,335	0,624	0,154	0,139	-0,148	916,055	133,57
TERF2	0,002	0,336	0,624	0,181	0,157	-0,206	403,71	68,183
EPN2	0,002	0,336	0,624	0,147	0,166	0,178	623,509	96,44
CHMP2A	0,001	0,336	0,624	0,536	0,478	-0,164	287,826	147,529
RPA2	0,001	0,338	0,626	0,375	0,414	0,144	427,637	169,12
BSDC1	0,003	0,339	0,626	0,133	0,111	-0,259	333,349	40,661
MTF1	0,003	0,339	0,626	0,081	0,069	-0,229	667,95	49,899
TRRAP	0,001	0,338	0,626	0,165	0,181	0,131	1033,527	178,837
CHPF2	0,002	0,339	0,626	0,162	0,187	0,201	408,642	71,168
MED27	0,003	0,338	0,626	0,208	0,244	0,232	244,131	54,958

ARCN1	0,002	0,338	0,626	0,575	0,497	-0,212	152,288	80,505
PEMT	0,001	0,338	0,626	0,382	0,342	-0,159	382,448	139,108
NPLOC4	0,001	0,338	0,626	0,097	0,089	-0,121	2180,05	203,488
CASP8	0,002	0,34	0,627	0,148	0,128	-0,21	458,644	63,101
C9orf78	0,002	0,34	0,627	0,153	0,172	0,172	601,227	97,332
L3HYPDH	0,002	0,34	0,627	0,563	0,645	0,196	160,64	97,392
COPS2	0,001	0,339	0,627	0,187	0,205	0,137	836,988	164,747
RPS2	0,001	0,339	0,627	0,223	0,208	-0,101	1680,348	361,853
BAX	0,001	0,34	0,627	0,355	0,322	-0,138	531,68	179,894
RNASEH2B	0,002	0,341	0,629	0,302	0,342	0,178	316,799	101,689
S100A10	0,001	0,342	0,63	0,815	0,777	-0,069	2055,9	1634,368
LAMTOR3	0,003	0,342	0,63	0,333	0,279	-0,258	149,067	45,131
CDC16	0,001	0,341	0,63	0,178	0,196	0,143	779,584	145,571
DDX52	0,002	0,342	0,63	0,093	0,108	0,204	640,17	64,836
ELAVL1	0,001	0,342	0,63	0,336	0,316	-0,088	1667,871	543,647
IRS1	0,001	0,343	0,631	0,134	0,125	-0,107	2085,674	270,765
TADA3	0,002	0,344	0,631	0,085	0,098	0,195	848,645	76,105
TMPPE	0,003	0,344	0,631	0,926	0,759	-0,287	59,732	50,75
MCM7	0,001	0,343	0,631	0,175	0,186	0,083	3214,309	580,492
C11orf84	0,002	0,344	0,631	0,13	0,148	0,192	544,138	75,078
KAT5	0,002	0,343	0,631	0,246	0,278	0,177	371,879	97,648
MRPL42	0,001	0,343	0,631	0,399	0,437	0,132	487,631	203,841
MAP4K5	0,002	0,344	0,631	0,094	0,107	0,19	748,369	74,771
PCNXL4	0,001	0,343	0,631	0,137	0,148	0,109	1872,028	267,767
CSK	0,001	0,343	0,631	0,212	0,193	-0,134	860,472	174,908
CKLF	0,003	0,343	0,631	0,411	0,485	0,238	130,016	58,337
INPP5K	0,002	0,344	0,631	0,188	0,166	-0,184	471,729	83,228
NAPG	0,001	0,343	0,631	0,233	0,258	0,147	584,716	143,673
RUSC1	0,001	0,345	0,632	0,166	0,149	-0,153	776,77	122,404
RNF130	0,002	0,346	0,632	0,188	0,216	0,203	343,025	69,219
CHRAC1	0,003	0,345	0,632	0,095	0,082	-0,212	670,898	58,52
RNF214	0,002	0,345	0,632	0,34	0,385	0,181	270,119	98

VWA8	0,003	0,345	0,632	0,107	0,091	-0,233	484,305	47,788
GGA2	0,002	0,345	0,632	0,125	0,11	-0,19	634,275	74,686
PCGF2	0,002	0,345	0,632	0,224	0,197	-0,189	405,953	86,449
SNX12	0,001	0,345	0,632	0,232	0,209	-0,149	614,956	135,494
DCBLD2	0,002	0,347	0,634	0,129	0,144	0,163	761,615	103,895
HCLS1	0,002	0,347	0,635	0,211	0,182	-0,214	329,93	65,67
PDCD6	0,001	0,347	0,635	0,45	0,411	-0,13	487,385	209,557
ADORA2B	0,001	0,347	0,635	0,369	0,348	-0,084	1749,865	629,441
ANAPC4	0,003	0,348	0,636	0,251	0,295	0,235	192,15	52,656
CTSV	0,003	0,348	0,636	0,548	0,656	0,26	87,501	53,329
NUDT15	0,002	0,348	0,636	0,13	0,15	0,209	434,398	60,971
MRPL4	0,001	0,349	0,636	0,429	0,46	0,103	839,263	371,54
PI4KB	0,001	0,35	0,637	0,151	0,138	-0,13	1199,232	174,067
COX18	0,002	0,349	0,637	0,418	0,359	-0,219	167,901	64,793
UBE2I	0,001	0,349	0,637	0,302	0,33	0,126	662,564	209,206
FLII	0,001	0,349	0,637	0,157	0,164	0,067	9055,411	1448,431
SDF2	0,002	0,349	0,637	0,474	0,541	0,19	186,642	94,54
SIN3B	0,001	0,35	0,637	0,153	0,169	0,14	916,347	147,178
CSTF1	0,001	0,349	0,637	0,212	0,234	0,144	651,001	145,026
RBFOX2	0,001	0,349	0,637	0,092	0,101	0,132	1679,046	162,198
PHYHD1	0,002	0,351	0,639	0,43	0,372	-0,207	185,402	74,639
CTC1	0,002	0,351	0,639	0,139	0,122	-0,184	604,07	79,222
OPA1	0,001	0,352	0,64	0,106	0,116	0,13	1562,303	172,958
UGDH	0,002	0,352	0,64	0,322	0,286	-0,171	342,91	104,478
SEC24D	0,001	0,352	0,64	0,237	0,217	-0,126	874,866	198,989
CCND2	0,003	0,352	0,64	0,222	0,283	0,353	184,322	42,406
CYB5B	0,001	0,352	0,64	0,56	0,523	-0,099	763,933	412,269
PHF12	0,003	0,353	0,64	0,098	0,084	-0,23	526,275	48,027
CHERP	0,001	0,352	0,64	0,188	0,202	0,107	1461,303	283,729
CLPTM1	0,001	0,352	0,64	0,417	0,44	0,078	1843,417	787,762
NT5C	0,002	0,353	0,641	0,38	0,432	0,184	233,534	94,306
CHCHD10	0,001	0,353	0,641	3,454	3,931	0,187	75,854	277,37

ADAR	0,001	0,355	0,642	0,071	0,076	0,104	3587,157	264,118
PRKCE	0,002	0,354	0,642	0,158	0,138	-0,195	471,34	69,133
CCDC14	0,003	0,354	0,642	0,061	0,071	0,218	771,582	51,027
PDSS1	0,003	0,355	0,642	0,383	0,328	-0,225	167,729	59,425
STK32C	0,003	0,354	0,642	0,276	0,231	-0,258	167,952	42,86
SMAD3	0,001	0,355	0,642	0,091	0,083	-0,128	1912,913	167,171
DPH1	0,001	0,355	0,642	0,383	0,427	0,158	319,391	129,37
UTP6	0,002	0,354	0,642	0,167	0,145	-0,201	421,858	65,457
RUNDC1	0,002	0,355	0,642	0,234	0,204	-0,198	322,952	70,728
SMARCD2	0,001	0,355	0,642	0,21	0,19	-0,141	719,361	143,598
ATP5H	0,001	0,355	0,642	1,162	1,057	-0,137	228,669	254,287
MYO1E	0,003	0,356	0,643	0,143	0,124	-0,211	434,698	57,983
C17orf89	0,003	0,355	0,643	0,508	0,607	0,256	92,628	51,319
PPCS	0,002	0,358	0,644	0,503	0,442	-0,189	193,388	90,589
PCYOX1	0,001	0,356	0,644	0,225	0,243	0,111	1071,525	251,798
SMARCC1	0,001	0,358	0,644	0,076	0,084	0,133	1811,539	146,213
RNF7	0,003	0,358	0,644	0,311	0,262	-0,248	159,9	45,669
DCK	0,002	0,358	0,644	0,267	0,238	-0,169	391,355	98,506
DCTD	0,001	0,356	0,644	0,159	0,146	-0,126	1169,799	178,651
GMNN	0,001	0,358	0,644	0,449	0,407	-0,14	395,167	167,722
MCM3	0,001	0,357	0,644	0,203	0,192	-0,083	2676,695	526,925
PARD3	0,002	0,359	0,644	0,092	0,082	-0,175	940,265	81,629
SCYL1	0,001	0,358	0,644	0,122	0,134	0,135	1220,29	155,514
PC	0,002	0,358	0,644	0,186	0,166	-0,165	569,605	100,624
INPPL1	0,001	0,358	0,644	0,09	0,082	-0,132	1813,134	157,247
ZC3H13	0,003	0,357	0,644	0,022	0,026	0,222	1969,954	47,379
DLST	0,001	0,357	0,644	0,119	0,108	-0,135	1309,668	148,295
VPS13C	0,002	0,357	0,644	0,144	0,162	0,168	622,967	95,155
SCAMP2	0,001	0,356	0,644	0,383	0,414	0,114	679,08	269,553
SKA2	0,001	0,356	0,644	0,142	0,157	0,147	906,133	137,539
MAP1S	0,001	0,357	0,644	0,175	0,194	0,142	769,775	141
SAE1	0,001	0,356	0,644	0,216	0,201	-0,106	1317,689	273,429

AP1B1	0,001	0,357	0,644	0,165	0,177	0,103	1718,67	292,881
THRAP3	0,002	0,359	0,645	0,044	0,049	0,153	2253,998	104,923
PROSC	0,003	0,359	0,645	0,198	0,171	-0,206	341,677	62,445
MRPL15	0,001	0,359	0,645	0,421	0,459	0,126	484,87	213,821
UNKL	0,003	0,359	0,645	0,126	0,108	-0,23	410,191	48,254
PLD3	0,001	0,359	0,645	0,345	0,374	0,115	702,677	251,918
COQ9	0,002	0,361	0,647	0,214	0,241	0,167	440,025	99,617
FBXO38	0,002	0,361	0,648	0,192	0,22	0,191	355,927	73,929
RPL41	0,002	0,361	0,648	3,279	2,723	-0,268	35,465	105,495
ASF1B	0,001	0,361	0,648	0,28	0,261	-0,103	1142,626	309,573
SDHB	0,001	0,362	0,649	0,459	0,491	0,099	805,675	383
HAX1	0,001	0,363	0,649	0,384	0,354	-0,117	646,823	238,569
ACP1	0,001	0,362	0,649	0,337	0,305	-0,144	448,639	143,405
SFXN3	0,001	0,362	0,649	0,216	0,197	-0,132	793,205	164,153
ATPAF1	0,002	0,364	0,652	0,198	0,172	-0,205	332,113	61,483
CLIP4	0,003	0,365	0,652	0,132	0,152	0,211	396,05	56,249
CDC23	0,001	0,364	0,652	0,248	0,228	-0,123	810,542	192,552
RPUSD4	0,002	0,364	0,652	0,176	0,202	0,197	351,47	66,507
SNRPA1	0,003	0,365	0,652	0,296	0,346	0,223	179,495	57,096
PIEZO1	0,001	0,365	0,652	0,122	0,13	0,094	2661,437	334,41
AP2B1	0,001	0,365	0,652	0,217	0,204	-0,088	1964,173	413,118
ISG15	0,001	0,367	0,653	0,951	0,884	-0,106	443,257	407,417
NECAP2	0,002	0,365	0,653	0,229	0,255	0,158	460,136	111,222
NME7	0,002	0,367	0,653	0,421	0,373	-0,173	258,9	103,279
TOR1AIP1	0,001	0,366	0,653	0,138	0,152	0,14	927,551	133,897
MPP4	0,002	0,368	0,653	0,311	0,346	0,152	407,475	132,59
HMGN3	0,003	0,367	0,653	0,261	0,307	0,231	177,889	50,956
AP5Z1	0,002	0,366	0,653	0,224	0,256	0,191	307,597	73,581
NSMAF	0,002	0,366	0,653	0,081	0,092	0,19	756,817	65,346
BLOC1S2	0,003	0,367	0,653	0,286	0,242	-0,241	174,786	45,793
AP2A2	0,001	0,366	0,653	0,22	0,238	0,112	1052,877	240,38
INTS4	0,002	0,367	0,653	0,231	0,264	0,194	288,181	71,7

GIT2	0,002	0,366	0,653	0,134	0,154	0,198	444,333	64,015
LRP10	0,001	0,365	0,653	0,189	0,175	-0,112	1232,324	224,298
CPSF2	0,001	0,367	0,653	0,17	0,158	-0,105	1634,152	267,999
PTPLAD1	0,001	0,367	0,653	0,244	0,263	0,105	1086,197	275,315
GTPBP3	0,002	0,366	0,653	0,231	0,204	-0,178	388,408	84,25
EPN1	0,001	0,366	0,653	0,115	0,105	-0,13	1399,065	152,37
MCL1	0,001	0,368	0,654	0,124	0,131	0,08	3997,45	512,108
COPA	0,001	0,368	0,654	0,174	0,164	-0,082	2881,528	486,755
SLAIN2	0,002	0,368	0,654	0,083	0,093	0,16	1063,376	94,21
ORC5	0,002	0,369	0,654	0,272	0,312	0,196	242,306	70,92
SAC3D1	0,002	0,368	0,654	0,497	0,577	0,218	124,605	66,605
NMRAL1	0,002	0,368	0,654	0,247	0,282	0,191	282,073	74,204
LMF2	0,002	0,369	0,654	0,441	0,492	0,158	277,05	128,174
ANGEL2	0,003	0,37	0,655	0,154	0,179	0,221	302,762	50,351
MZT2A	0,003	0,37	0,655	0,575	0,492	-0,225	116,634	62,375
ELP6	0,002	0,37	0,655	0,34	0,379	0,156	333,968	120,258
ANXA7	0,001	0,369	0,655	0,441	0,414	-0,091	1053,472	449,565
JKAMP	0,001	0,369	0,655	0,524	0,482	-0,122	449,106	224,933
SLIRP	0,001	0,37	0,655	3,137	3,387	0,111	228,246	742,785
EMC8	0,002	0,369	0,655	0,313	0,348	0,157	358,512	117,919
CSNK1G2	0,002	0,37	0,655	0,075	0,066	-0,169	1187,441	83,94
MAN2B1	0,002	0,37	0,655	0,251	0,28	0,157	421,822	111,414
GMEB2	0,002	0,37	0,655	0,174	0,151	-0,203	367,736	59,301
SELK	0,003	0,371	0,656	0,318	0,275	-0,213	201,716	59,324
TBP	0,002	0,371	0,656	0,33	0,368	0,155	345,786	120,529
DHX32	0,002	0,371	0,656	0,172	0,193	0,169	489,849	89,313
PUM1	0,001	0,372	0,657	0,181	0,167	-0,117	1141,508	199,508
CCT3	0,001	0,372	0,657	0,311	0,325	0,061	5190,219	1648,089
INTS9	0,002	0,372	0,657	0,255	0,223	-0,196	283,368	67,508
PTPN11	0,002	0,372	0,657	0,042	0,038	-0,155	2466,398	98,826
C20orf27	0,001	0,372	0,657	0,282	0,259	-0,122	715,748	193,414
ENAH	0,002	0,374	0,659	0,091	0,102	0,163	929,95	89,599

TTL	0,004	0,374	0,659	0,046	0,038	-0,265	821,063	35,179
CASP4	0,001	0,373	0,659	0,262	0,289	0,14	527,55	144,161
RFC3	0,001	0,373	0,659	0,435	0,407	-0,094	943,681	396,148
DCTN5	0,002	0,374	0,659	0,149	0,134	-0,154	735,577	104,204
SMG8	0,002	0,374	0,659	0,174	0,197	0,178	423,301	78,693
TMEM214	0,002	0,375	0,66	0,133	0,12	-0,147	914,69	115,458
ALYREF	0,001	0,375	0,66	0,731	0,771	0,076	1105,743	829,07
SH3KBP1	0,002	0,375	0,66	0,109	0,122	0,16	836,187	96,087
ARPC5	0,001	0,376	0,661	0,324	0,304	-0,092	1297,563	409,012
UBXN4	0,001	0,376	0,661	0,159	0,173	0,116	1197,293	198,486
ME1	0,002	0,376	0,661	0,266	0,238	-0,163	396,563	99,802
C2CD3	0,002	0,376	0,661	0,055	0,063	0,192	1036,27	61,93
SLC38A7	0,002	0,376	0,661	0,17	0,193	0,188	385,986	69,749
ELAC2	0,001	0,376	0,661	0,129	0,137	0,083	3144,797	419,485
HNRNPUL1	0,001	0,376	0,661	0,111	0,117	0,078	4484,318	509,744
NENF	0,002	0,377	0,662	0,787	0,688	-0,193	128,881	95,816
STAM2	0,002	0,377	0,662	0,266	0,295	0,151	418,566	117,461
G3BP2	0,001	0,378	0,662	0,104	0,114	0,123	1534,291	167,924
DBN1	0,001	0,377	0,662	0,175	0,164	-0,089	2198,239	372,153
NT5DC1	0,002	0,377	0,662	0,324	0,373	0,206	188,496	65,242
ENSG00000218426.5	0,003	0,377	0,662	6,087	4,467	-0,446	10,682	57,274
NFRKB	0,002	0,377	0,662	0,115	0,13	0,178	605,377	74,364
MSRB3	0,003	0,377	0,662	0,078	0,067	-0,232	584,286	42,493
GCDH	0,002	0,377	0,662	0,41	0,356	-0,202	178,424	67,745
RPS9	0,001	0,378	0,662	1,087	0,998	-0,124	266,25	278,111
SUZ12	0,002	0,379	0,663	0,09	0,102	0,173	790,474	76,066
SLC35E3	0,003	0,379	0,664	0,467	0,39	-0,257	101,164	43,599
TAF1A	0,003	0,38	0,665	0,401	0,343	-0,226	146,043	54,755
XPO1	0,001	0,381	0,665	0,154	0,161	0,064	6293,109	994,78
BZW1	0,001	0,38	0,665	0,676	0,723	0,098	570,828	399,992
RPL15	0,001	0,38	0,665	0,157	0,149	-0,082	2821,216	430,851
UBXN7	0,003	0,38	0,665	0,094	0,108	0,197	576,495	58,811

EXOC3	0,002	0,381	0,665	0,207	0,229	0,149	529,034	115,656
NAA35	0,002	0,38	0,665	0,172	0,194	0,174	439,591	80,388
FKBP4	0,001	0,381	0,665	0,281	0,265	-0,087	1538,463	419,49
TOM1L2	0,002	0,381	0,665	0,097	0,085	-0,184	740,773	67,774
PIH1D1	0,001	0,381	0,665	0,382	0,351	-0,121	547,044	200,47
RNF2	0,003	0,382	0,666	0,302	0,262	-0,203	220,496	61,875
NOA1	0,002	0,382	0,666	0,288	0,257	-0,164	358,276	97,438
ACAT2	0,001	0,382	0,666	0,682	0,638	-0,095	637,803	419,912
OSTF1	0,002	0,381	0,666	0,555	0,498	-0,155	245,089	128,656
SOX9	0,001	0,382	0,666	0,246	0,229	-0,104	1094,263	260,768
ACADSB	0,002	0,383	0,667	0,203	0,233	0,199	284,746	61,863
MRC2	0,002	0,383	0,667	0,19	0,213	0,163	460,672	93,132
PEF1	0,001	0,384	0,668	0,345	0,314	-0,136	460,121	151,715
RNF11	0,002	0,383	0,668	0,073	0,065	-0,185	926,238	64,122
MOGS	0,001	0,384	0,668	0,316	0,297	-0,093	1132,981	346,779
LRRFIP2	0,001	0,384	0,668	0,141	0,155	0,138	855,323	126,146
QDPR	0,003	0,384	0,668	0,279	0,324	0,217	180,536	54,158
ATG5	0,002	0,385	0,668	0,3	0,266	-0,174	299,573	84,382
BANF1	0,001	0,384	0,668	0,845	0,783	-0,11	390,235	316,525
VEZT	0,003	0,385	0,668	0,097	0,084	-0,207	581,589	52,687
HERC1	0,003	0,383	0,668	0,121	0,139	0,202	429,805	55,827
YWHAB	0,001	0,384	0,668	0,239	0,249	0,06	5012,317	1223,632
NSUN4	0,002	0,386	0,669	0,141	0,158	0,169	544,334	81,229
MDH1	0,001	0,385	0,669	0,53	0,504	-0,072	1505,86	777,908
LSG1	0,003	0,385	0,669	0,112	0,129	0,199	473,208	57,242
SH3BP2	0,003	0,386	0,669	0,069	0,06	-0,213	739,094	47,581
EXOC2	0,002	0,386	0,669	0,141	0,127	-0,156	714,963	95,625
VDAC2	0,002	0,385	0,669	1,09	0,963	-0,178	117,884	120,141
PEX5	0,002	0,386	0,669	0,247	0,276	0,16	392,97	101,83
ETNK1	0,001	0,386	0,669	0,226	0,246	0,122	751,537	177,382
SMARCD1	0,002	0,385	0,669	0,121	0,133	0,135	1052,162	133,64
GDE1	0,002	0,385	0,669	0,099	0,112	0,179	669,249	70,367

STX4	0,002	0,385	0,669	0,261	0,299	0,196	236,636	65,968
DSCR3	0,002	0,386	0,669	0,203	0,178	-0,193	335,414	63,703
DNAJC6	0,002	0,387	0,67	0,127	0,114	-0,162	730,377	88,163
NFU1	0,001	0,387	0,67	0,504	0,462	-0,125	406,166	196,465
POMT1	0,002	0,388	0,67	0,26	0,29	0,156	381,989	105,473
TRUB1	0,002	0,387	0,67	0,241	0,215	-0,164	401,901	91,184
C12orf5	0,002	0,388	0,67	0,43	0,486	0,178	195,165	89,825
CD9	0,001	0,388	0,67	1,151	1,22	0,084	590,118	699,073
CHFR	0,003	0,387	0,67	0,118	0,139	0,231	338,879	42,853
PHKB	0,002	0,388	0,67	0,109	0,122	0,165	716,203	82,648
RPS19	0,001	0,387	0,67	2,235	2,122	-0,075	620,081	1350,491
MRPS26	0,001	0,388	0,67	0,655	0,596	-0,136	277,992	173,539
FARSB	0,001	0,389	0,671	0,232	0,217	-0,096	1400,423	313,49
SPG11	0,002	0,389	0,671	0,176	0,194	0,147	595,231	110,162
SLC12A4	0,001	0,389	0,671	0,297	0,322	0,116	669,205	205,89
EIF4A3	0,001	0,389	0,671	0,538	0,51	-0,076	1280,874	670,23
DNAJC16	0,002	0,39	0,672	0,257	0,223	-0,202	254,842	61,694
PKP4	0,002	0,391	0,673	0,055	0,05	-0,14	2185,926	113,331
SSSCA1	0,001	0,39	0,673	1,099	1,196	0,122	249,211	284,884
NUSAP1	0,001	0,391	0,673	0,184	0,196	0,09	1786,146	339,325
SF3B3	0,001	0,39	0,673	0,075	0,07	-0,088	4502,953	326,054
FN3KRP	0,002	0,391	0,673	0,299	0,261	-0,194	235,048	66,028
ORC1	0,003	0,392	0,674	0,074	0,086	0,209	604,672	48,118
GFPT1	0,001	0,393	0,674	0,142	0,132	-0,109	1557,159	213,923
RRP9	0,001	0,391	0,674	0,303	0,278	-0,127	565,596	164,137
DHTKD1	0,003	0,392	0,674	0,141	0,123	-0,193	453,324	59,448
ITPRIP	0,001	0,393	0,674	0,146	0,161	0,138	807,379	123,289
HTRA1	0,003	0,393	0,674	0,433	0,516	0,252	96,459	44,832
POC1B	0,003	0,392	0,674	0,202	0,174	-0,215	261,393	48,932
ENSG00000231607.4	0,003	0,392	0,674	0,322	0,377	0,226	142,985	50,637
POLE2	0,002	0,392	0,674	0,319	0,361	0,176	245,689	83,785
C15orf52	0,003	0,392	0,674	0,04	0,045	0,187	1387,734	59,222

RBM12	0,001	0,393	0,674	0,172	0,185	0,106	1342,651	239,704
CREBZF	0,003	0,394	0,675	0,09	0,078	-0,204	610,869	51,218
CDCA2	0,001	0,394	0,676	0,193	0,177	-0,129	778,439	143,705
SECTM1	0,004	0,394	0,676	0,358	0,291	-0,298	105,426	35,512
WASF2	0,002	0,395	0,677	0,055	0,049	-0,159	1656,312	85,825
NDC1	0,001	0,395	0,677	0,278	0,293	0,073	2406,501	686,666
MIOS	0,003	0,395	0,677	0,18	0,209	0,214	256,453	49,577
NUP160	0,001	0,395	0,677	0,193	0,18	-0,102	1327,488	247,083
SLC25A15	0,002	0,395	0,677	0,273	0,312	0,192	225,572	66,392
ZBTB4	0,002	0,396	0,677	0,05	0,044	-0,168	1610,342	75,988
PMAIP1	0,002	0,395	0,677	0,224	0,254	0,186	285,837	68,124
PCNP	0,002	0,397	0,678	0,147	0,132	-0,16	614,107	85,327
MRPS30	0,001	0,397	0,678	0,361	0,338	-0,094	930,692	325,373
WDR36	0,001	0,396	0,678	0,24	0,223	-0,105	1041,759	241,497
ZWINT	0,001	0,397	0,678	0,283	0,269	-0,072	2521,995	695,973
RDX	0,002	0,396	0,678	0,057	0,064	0,175	1123,071	68,258
OGFOD1	0,002	0,396	0,678	0,112	0,124	0,151	821,013	97,316
OXSR1	0,001	0,397	0,679	0,114	0,104	-0,132	1170,053	127,226
GTF3C5	0,001	0,398	0,68	0,169	0,182	0,109	1174,932	205,394
SSR2	0,001	0,4	0,681	0,404	0,423	0,066	2253,948	931,899
MSH2	0,001	0,399	0,681	0,161	0,148	-0,13	876,626	134,578
C3orf14	0,002	0,4	0,681	0,384	0,438	0,19	172,395	71,124
MINA	0,002	0,399	0,681	0,168	0,188	0,158	507,89	90,199
SERINC1	0,001	0,399	0,681	0,53	0,558	0,076	1171,746	638,284
GINM1	0,002	0,4	0,681	0,432	0,487	0,173	194,666	90,082
RBM14	0,001	0,4	0,681	0,14	0,129	-0,121	1152,56	154,839
UBL3	0,002	0,399	0,681	0,189	0,169	-0,157	517,331	91,825
RARA	0,002	0,399	0,681	0,211	0,242	0,195	272,351	61,129
NMT1	0,001	0,4	0,681	0,143	0,152	0,088	2270,299	333,679
TYMS	0,001	0,399	0,681	0,386	0,365	-0,079	1341,466	502,114
XRN2	0,001	0,4	0,681	0,118	0,125	0,09	2498,849	303,753
STX7	0,002	0,401	0,682	0,29	0,261	-0,156	364,972	100,442

TMEM209	0,001	0,401	0,682	0,389	0,36	-0,113	576,64	215,215
PINK1	0,003	0,403	0,684	0,425	0,362	-0,231	125,191	49,983
ABHD6	0,003	0,402	0,684	0,289	0,248	-0,22	178,176	47,713
PALLD	0,001	0,403	0,684	0,168	0,183	0,121	937,148	163,141
UBTD1	0,003	0,402	0,684	0,154	0,132	-0,218	317,212	45,559
STARD13	0,002	0,403	0,684	0,082	0,073	-0,176	868,751	67,881
GALC	0,002	0,403	0,684	0,269	0,3	0,158	343,815	97,181
PIAS1	0,003	0,402	0,684	0,14	0,16	0,196	373,819	55,69
APMAP	0,001	0,402	0,684	0,42	0,394	-0,093	878,834	355,389
GLTPD1	0,003	0,404	0,685	0,163	0,187	0,2	319,938	55,236
ZC3HAV1L	0,002	0,404	0,685	0,506	0,576	0,186	147,107	79,337
UAP1	0,001	0,404	0,686	0,224	0,238	0,086	1600,493	369,622
ERCC6L2	0,003	0,405	0,686	0,093	0,105	0,186	594,847	58,923
PTPRS	0,001	0,404	0,686	0,154	0,169	0,135	772,541	123,825
ARF1	0,001	0,407	0,687	0,233	0,223	-0,064	4304,532	985,874
USP19	0,002	0,406	0,687	0,117	0,105	-0,144	950,249	106,251
PPP2R3A	0,003	0,406	0,687	0,109	0,125	0,206	412,946	48,113
PWWP2A	0,003	0,406	0,687	0,24	0,209	-0,202	242,115	54,104
XPO5	0,001	0,405	0,687	0,158	0,147	-0,105	1445,34	220,32
MAK16	0,002	0,407	0,687	0,135	0,151	0,161	570,805	81,851
CISD1	0,001	0,406	0,687	0,451	0,416	-0,118	465,137	202,3
ARFGAP2	0,002	0,407	0,687	0,174	0,154	-0,177	418,898	68,705
SAMD4A	0,002	0,407	0,687	0,072	0,065	-0,165	1064,969	72,908
PRPSAP2	0,001	0,406	0,687	0,424	0,39	-0,123	432,265	175,707
ZNF24	0,003	0,407	0,687	0,129	0,111	-0,213	374,049	44,547
GID8	0,002	0,407	0,687	0,304	0,275	-0,148	376,999	108,833
CGGBP1	0,001	0,408	0,688	0,123	0,112	-0,13	1083,419	126,999
TMCO6	0,004	0,408	0,688	0,329	0,277	-0,248	129,327	39,688
G3BP1	0,001	0,407	0,688	0,15	0,143	-0,074	3363,5	491,697
RC3H2	0,002	0,408	0,688	0,042	0,037	-0,18	1522,785	60,713
HERC4	0,001	0,408	0,688	0,171	0,185	0,116	953,446	169,541
IFIT5	0,002	0,408	0,688	0,279	0,251	-0,155	364,263	96,574

TMEM101	0,001	0,407	0,688	0,738	0,671	-0,137	234,889	166,196
LRP5	0,002	0,41	0,69	0,125	0,114	-0,139	974,398	117,709
SLC35C2	0,002	0,41	0,69	0,206	0,233	0,177	321,148	70,297
MRPL3	0,001	0,411	0,692	0,294	0,275	-0,095	1021,806	290,346
TSC1	0,003	0,411	0,692	0,105	0,092	-0,188	579,335	57,443
DCAF5	0,003	0,411	0,692	0,074	0,084	0,19	689,064	54,454
BAG5	0,002	0,411	0,692	0,129	0,145	0,168	537,272	74,213
CMIP	0,001	0,411	0,692	0,2	0,212	0,085	1727,185	354,979
WRAP73	0,002	0,412	0,693	0,324	0,366	0,176	225,443	77,399
AP4B1	0,003	0,412	0,693	0,317	0,272	-0,218	162,49	48,102
SMG7	0,002	0,413	0,693	0,076	0,083	0,135	1410,826	111,516
RMDN1	0,002	0,412	0,693	0,364	0,326	-0,158	277,722	95,277
MOB3A	0,001	0,412	0,693	0,173	0,162	-0,099	1396,582	234,27
GALE	0,001	0,414	0,694	0,536	0,494	-0,118	385,956	198,841
DRAM2	0,003	0,414	0,694	0,349	0,304	-0,201	176,634	57,869
DCLRE1B	0,002	0,414	0,694	0,182	0,161	-0,172	408,322	69,917
CENPL	0,002	0,414	0,694	0,27	0,239	-0,176	280,056	70,966
MTIF2	0,002	0,413	0,694	0,142	0,158	0,153	598,087	90,503
SCOC	0,002	0,413	0,694	0,38	0,341	-0,157	269,521	96,574
CTSB	0,001	0,413	0,694	0,27	0,289	0,099	942,535	262,668
TSTA3	0,001	0,413	0,694	0,396	0,427	0,108	552,124	227,346
PTRF	0,001	0,414	0,694	0,114	0,119	0,056	9833,014	1144,761
B4GALT5	0,002	0,413	0,694	0,09	0,099	0,146	1040,017	99,244
ENSA	0,001	0,415	0,695	0,178	0,164	-0,122	866,736	148,041
G0S2	0,003	0,415	0,695	0,402	0,465	0,211	138,825	58,873
TSSC1	0,002	0,415	0,695	0,272	0,241	-0,177	275,911	70,709
BTG1	0,002	0,415	0,695	0,22	0,25	0,184	272,184	63,907
TP53	0,001	0,415	0,695	0,287	0,274	-0,066	2818,323	791,587
PROSER2	0,003	0,417	0,696	0,202	0,231	0,195	260,26	56,072
OSBP	0,001	0,417	0,696	0,124	0,135	0,125	1016,932	131,868
DPF2	0,002	0,417	0,696	0,145	0,133	-0,131	882,7	122,849
PDCD5	0,001	0,416	0,696	0,404	0,368	-0,136	348,494	134,476

MID1IP1	0,002	0,416	0,696	0,283	0,312	0,142	389,413	115,364
ARID1B	0,003	0,418	0,697	0,115	0,131	0,186	462,989	56,839
CD2BP2	0,002	0,417	0,697	0,116	0,103	-0,168	641,051	70
TRMT1	0,001	0,418	0,697	0,243	0,262	0,109	804,782	203,269
KIF3B	0,002	0,417	0,697	0,036	0,04	0,163	1876,962	71,59
PLCG1	0,002	0,418	0,697	0,065	0,071	0,135	1585,399	107,337
RAD18	0,002	0,419	0,698	0,127	0,139	0,13	908,961	121,112
RPS5	0,001	0,418	0,698	1,037	1	-0,053	2371,818	2414,519
PGD	0,001	0,42	0,699	0,198	0,212	0,1	1136,316	232,366
EXOC6B	0,002	0,42	0,699	0,11	0,121	0,141	867,233	100,779
SLC33A1	0,002	0,42	0,699	0,265	0,297	0,162	297,058	83,855
OSTM1	0,002	0,42	0,699	0,265	0,239	-0,148	400,196	100,218
OGDH	0,001	0,419	0,699	0,156	0,165	0,076	2778,323	445,226
BAD	0,002	0,419	0,699	0,437	0,387	-0,175	189,514	78,013
SACS	0,001	0,42	0,699	0,086	0,092	0,098	2566,36	227,886
BCAT2	0,001	0,42	0,699	0,552	0,511	-0,11	425,145	226,111
NRSN2	0,002	0,419	0,699	0,13	0,146	0,167	536,228	73,227
TIMP1	0,001	0,42	0,699	1,061	1,119	0,077	665,878	723,114
SELRC1	0,001	0,421	0,7	0,346	0,317	-0,125	459,4	152,395
S100A16	0,001	0,421	0,7	0,35	0,33	-0,083	1251,674	426,962
EFNA5	0,003	0,421	0,7	0,155	0,18	0,213	264,689	44,128
DNAJB6	0,002	0,421	0,7	0,091	0,083	-0,13	1365,135	118,527
AZI2	0,002	0,422	0,701	0,26	0,231	-0,174	286,831	69,765
PSMD5	0,001	0,422	0,701	0,265	0,283	0,093	1044,022	286,847
COMMD9	0,001	0,422	0,701	0,391	0,359	-0,124	422,806	159,177
GIT1	0,002	0,423	0,701	0,067	0,075	0,167	953,883	67,718
AACS	0,002	0,423	0,702	0,19	0,212	0,157	418,919	84,193
COMMD2	0,002	0,424	0,703	0,231	0,26	0,172	295,291	71,898
PDDC1	0,002	0,424	0,703	0,127	0,115	-0,153	694,692	83,705
NTHL1	0,003	0,424	0,703	0,526	0,622	0,242	82,845	46,396
FBXO42	0,003	0,425	0,704	0,203	0,176	-0,211	235,049	44,35
PRPF4B	0,002	0,425	0,704	0,052	0,059	0,169	1146,708	63,597

SLC9A3R2	0,001	0,425	0,705	0,212	0,196	-0,114	819,335	166,969
SLC25A17	0,003	0,426	0,705	0,261	0,229	-0,188	240,223	58,356
NIPAL3	0,003	0,427	0,706	0,1	0,088	-0,183	588,137	55,049
SLC26A2	0,002	0,426	0,706	0,179	0,2	0,16	409,668	77,829
KIAA0196	0,002	0,427	0,706	0,134	0,15	0,156	574,129	82,129
SUPT20H	0,001	0,426	0,706	0,119	0,11	-0,121	1223,459	140,188
RP11-676J12.7	0,002	0,427	0,706	0,583	0,518	-0,169	162,493	89,792
DOCK6	0,003	0,427	0,706	0,12	0,104	-0,216	358,935	40,451
TMX4	0,003	0,426	0,706	0,114	0,099	-0,205	421,545	44,977
CCBL2	0,002	0,428	0,707	0,292	0,329	0,173	232,18	72,116
GNAI3	0,001	0,428	0,707	0,288	0,273	-0,077	1554,093	433,984
RNF187	0,002	0,428	0,707	0,19	0,21	0,141	526,288	104,625
TMEM63B	0,003	0,428	0,707	0,184	0,161	-0,188	328,739	57,08
TES	0,002	0,428	0,707	0,196	0,177	-0,149	491,462	91,385
ASUN	0,002	0,429	0,707	0,169	0,15	-0,17	417,428	66,562
RAB35	0,001	0,428	0,707	0,163	0,176	0,116	917,894	154,991
C1orf109	0,001	0,429	0,708	0,258	0,237	-0,125	563,036	138,569
METTL13	0,002	0,43	0,708	0,152	0,137	-0,149	606,91	88,159
NLN	0,002	0,429	0,708	0,133	0,146	0,129	871,971	121,15
E2F7	0,002	0,429	0,708	0,11	0,1	-0,136	1007,507	106,125
DYNLL1	0,001	0,429	0,708	0,715	0,757	0,081	684,428	503,877
VANGL1	0,001	0,431	0,709	0,122	0,112	-0,123	1113,339	130,163
TTLL4	0,002	0,431	0,709	0,128	0,114	-0,168	560,209	67,932
FANCC	0,002	0,431	0,709	0,208	0,236	0,179	279,404	62,128
STXBP1	0,002	0,431	0,709	0,097	0,107	0,144	917,461	92,767
C9orf142	0,002	0,432	0,709	0,349	0,316	-0,14	341,895	113,973
HK1	0,001	0,43	0,709	0,096	0,101	0,082	3432,02	336,505
WDR74	0,001	0,431	0,709	0,407	0,44	0,114	444,933	188,195
KIF23	0,001	0,43	0,709	0,077	0,083	0,111	1960,027	157,577
MST4	0,002	0,432	0,709	0,205	0,23	0,169	320,506	69,695
ASPM	0,002	0,434	0,711	0,021	0,024	0,168	2725,963	60,58
PTPN14	0,001	0,434	0,711	0,035	0,038	0,118	3725,489	136,885

MARCH7	0,002	0,434	0,711	0,097	0,106	0,128	1127,134	114,33
EEF1B2	0,001	0,433	0,711	1,438	1,536	0,095	311,197	462,04
CRTAP	0,001	0,433	0,711	0,251	0,262	0,062	3075,921	787,272
RPL35A	0,001	0,434	0,711	0,764	0,795	0,057	1936,346	1507,737
MED23	0,003	0,434	0,711	0,156	0,18	0,2	277,443	46,501
MKLN1	0,002	0,433	0,711	0,117	0,13	0,156	614,255	76,089
LPAR1	0,001	0,433	0,711	0,249	0,266	0,094	1037,988	266,168
ATP5C1	0,001	0,433	0,711	0,364	0,386	0,085	975,696	365,429
ESD	0,001	0,433	0,711	0,642	0,601	-0,096	495,454	308,077
SMG6	0,001	0,434	0,711	0,068	0,074	0,115	2027,364	143,795
RAC2	0,001	0,432	0,711	0,696	0,728	0,066	1359,224	963,668
UXT	0,001	0,434	0,711	1,092	0,986	-0,147	139,996	145,543
OGT	0,002	0,433	0,711	0,042	0,046	0,126	2668,307	117,189
CLDN1	0,003	0,435	0,712	11,278	7,812	-0,53	5,259	50,084
SF3B1	0,001	0,436	0,713	0,084	0,089	0,084	3518,568	304,283
ARHGEF28	0,001	0,437	0,714	0,102	0,111	0,115	1370,479	144,875
MYO6	0,002	0,436	0,714	0,106	0,118	0,155	671,603	75,036
HMGN2	0,002	0,437	0,715	0,688	0,62	-0,15	174,314	113,12
UBR2	0,003	0,437	0,715	0,092	0,081	-0,187	578,524	49,797
MPLKIP	0,001	0,438	0,715	0,552	0,503	-0,135	253,652	134,051
TOLLIP	0,001	0,438	0,715	0,261	0,283	0,118	567,857	153,689
MUS81	0,002	0,438	0,715	0,143	0,16	0,167	435,26	66,195
CCNDBP1	0,002	0,437	0,715	0,524	0,583	0,154	183,428	101,639
FBXO22	0,002	0,438	0,715	0,404	0,363	-0,154	241,172	92,226
RRM2	0,001	0,439	0,716	0,216	0,226	0,067	2919,867	645,892
PPAT	0,002	0,439	0,716	0,224	0,198	-0,177	289,679	60,851
MED10	0,002	0,439	0,716	0,424	0,384	-0,143	270,132	108,354
MTA2	0,001	0,439	0,716	0,201	0,211	0,071	2652,896	545,797
MAP3K11	0,002	0,439	0,716	0,078	0,071	-0,135	1359,374	102,262
RNF26	0,001	0,439	0,716	0,221	0,203	-0,12	667,227	142,062
CEBPB	0,001	0,439	0,716	0,978	1,078	0,14	153,774	157,375
APOA1BP	0,001	0,44	0,717	0,323	0,352	0,123	428,55	144,62

POLR3D	0,003	0,44	0,717	0,093	0,106	0,185	508,492	50,782
SCAMP4	0,001	0,441	0,717	0,442	0,47	0,088	752,112	342,084
BID	0,002	0,441	0,717	0,315	0,287	-0,134	386,603	115,878
CTNNBIP1	0,003	0,442	0,718	0,193	0,222	0,206	220,091	45,106
ETV5	0,001	0,442	0,718	0,233	0,215	-0,116	685,828	153,377
C9orf69	0,004	0,442	0,718	0,079	0,067	-0,228	500,497	36,946
MEN1	0,001	0,441	0,718	0,142	0,154	0,115	971,072	143,591
GSTP1	0,001	0,442	0,718	0,897	0,814	-0,141	167,096	143,082
MINK1	0,001	0,442	0,718	0,074	0,069	-0,106	2447,073	175,568
AKAP8	0,003	0,442	0,718	0,098	0,11	0,173	561,733	58,103
BRD1	0,002	0,441	0,718	0,056	0,063	0,15	1333,151	78,762
PITHD1	0,002	0,444	0,719	0,246	0,277	0,174	244,562	64,205
DHDDS	0,002	0,444	0,719	0,157	0,176	0,163	412,979	68,77
MPC2	0,002	0,443	0,719	0,348	0,307	-0,178	196,104	64,084
SUCO	0,002	0,443	0,719	0,144	0,158	0,137	653,792	98,403
ATRAID	0,002	0,443	0,719	0,401	0,442	0,138	280,549	117,691
ABI2	0,002	0,444	0,719	0,072	0,065	-0,142	1269,715	87,586
LRCH1	0,002	0,444	0,719	0,136	0,152	0,161	488,841	69,989
BRD4	0,001	0,444	0,719	0,152	0,161	0,079	2242,605	350,707
MORC4	0,002	0,443	0,719	0,118	0,131	0,155	595,432	74,022
NUDT2	0,002	0,445	0,72	0,907	0,796	-0,188	89,06	75,573
KRR1	0,002	0,444	0,72	0,244	0,268	0,138	409,262	105,094
EIF5A	0,001	0,444	0,72	0,36	0,375	0,058	4318,948	1588,276
MKNK1	0,002	0,446	0,721	0,212	0,189	-0,162	352,479	70,734
AMPD2	0,002	0,447	0,721	0,135	0,148	0,133	733,665	102,872
RAP2B	0,003	0,446	0,721	0,147	0,168	0,195	310,426	49,733
PI4K2B	0,003	0,445	0,721	0,192	0,168	-0,191	274,894	49,112
TTC33	0,004	0,446	0,721	0,271	0,232	-0,224	152,539	38,645
TMEM261	0,004	0,446	0,721	0,884	1,067	0,271	42,073	39,965
PDCL	0,002	0,447	0,721	0,464	0,422	-0,136	271,392	119,992
TOR4A	0,001	0,445	0,721	0,208	0,224	0,106	859,578	185,118
SIK3	0,002	0,446	0,721	0,188	0,17	-0,145	490,072	88,013

YARS2	0,002	0,447	0,721	0,662	0,731	0,143	177,046	123,08
MPDU1	0,001	0,446	0,721	0,338	0,322	-0,068	1724,436	568,859
EZH2	0,002	0,448	0,722	0,085	0,095	0,152	817,48	73,56
MLLT1	0,001	0,447	0,722	0,229	0,249	0,123	546,464	130,658
S100A2	0,002	0,449	0,723	1,182	1,343	0,183	111,97	136,181
SETD5	0,001	0,449	0,723	0,057	0,053	-0,114	2378,715	131,358
PDLIM4	0,001	0,45	0,723	0,639	0,578	-0,145	218,739	135,438
TRERF1	0,003	0,448	0,723	0,086	0,075	-0,205	497,184	40,384
MARCKS	0,001	0,449	0,723	0,524	0,496	-0,078	953,16	487,183
HDDC2	0,002	0,449	0,723	0,384	0,348	-0,143	284,38	104,526
ASH2L	0,002	0,448	0,723	0,299	0,274	-0,13	420,898	120,887
RB1CC1	0,002	0,449	0,723	0,092	0,102	0,144	855,653	83,692
MTPAP	0,002	0,45	0,723	0,239	0,218	-0,13	487,617	110,911
C12orf10	0,001	0,449	0,723	0,44	0,481	0,129	297,871	136,448
DHX40	0,003	0,449	0,723	0,114	0,129	0,176	452,083	54,998
BCORL1	0,003	0,449	0,723	0,221	0,252	0,189	218,244	51,78
ATP2B4	0,002	0,45	0,724	0,106	0,118	0,145	736,59	82,075
RANBP2	0,002	0,45	0,724	0,046	0,052	0,153	1417,7	69,48
CLASP1	0,003	0,451	0,724	0,063	0,071	0,177	757,501	51,01
PMPCB	0,001	0,451	0,724	0,399	0,37	-0,108	482,118	184,979
KMT2E	0,002	0,45	0,724	0,081	0,072	-0,164	811,36	62,445
CREB3L2	0,001	0,451	0,724	0,083	0,089	0,108	1779,932	152,279
EFTUD2	0,001	0,45	0,724	0,112	0,106	-0,077	3332,621	362,016
SRSF7	0,001	0,452	0,725	0,461	0,439	-0,069	1287,98	578,944
BYSL	0,001	0,452	0,725	0,221	0,238	0,104	818,887	187,725
ANLN	0,001	0,452	0,725	0,077	0,081	0,059	9455,581	747,797
EXT1	0,001	0,453	0,725	0,099	0,105	0,078	3657,186	373,488
PSAP	0,001	0,452	0,725	0,558	0,578	0,052	3576,991	2028,427
C16orf59	0,003	0,453	0,725	0,255	0,292	0,196	178,707	48,696
MRPL10	0,001	0,453	0,725	0,353	0,381	0,111	475,323	174,244
RNMT	0,002	0,452	0,725	0,128	0,141	0,135	711,64	96,054
TAB1	0,003	0,452	0,725	0,179	0,205	0,193	247,321	47,316

IK	0,002	0,454	0,726	0,068	0,076	0,16	884,517	63,781
CASP2	0,003	0,453	0,726	0,179	0,156	-0,191	282,033	46,946
TXNDC12	0,001	0,455	0,727	0,263	0,281	0,095	844,282	229,818
ETV3	0,003	0,454	0,727	0,118	0,103	-0,189	420,05	46,229
BRIX1	0,001	0,456	0,727	0,226	0,21	-0,102	845,632	184,068
ZNF451	0,001	0,455	0,727	0,205	0,189	-0,121	632,122	124,186
BAG2	0,001	0,455	0,727	0,308	0,289	-0,09	878,103	262,349
ENPP1	0,002	0,456	0,727	0,228	0,254	0,159	299,874	72,576
NDUFA8	0,001	0,455	0,727	0,799	0,858	0,103	321,194	264,997
ATG13	0,003	0,454	0,727	0,066	0,058	-0,19	701,011	43,411
SMAGP	0,003	0,455	0,727	0,237	0,273	0,203	182,678	45,785
ALDH3A2	0,001	0,455	0,727	0,21	0,194	-0,116	704,211	141,832
ETV4	0,002	0,456	0,727	0,148	0,134	-0,147	562,778	78,995
MCM5	0,001	0,455	0,727	0,173	0,165	-0,066	3062,507	516,534
SRGN	0,001	0,457	0,728	0,764	0,737	-0,052	2182,119	1637,548
IVD	0,001	0,457	0,728	0,238	0,219	-0,119	581,904	132,491
USP14	0,001	0,456	0,728	0,219	0,231	0,074	1759,274	396,289
MANEAL	0,003	0,458	0,729	0,286	0,323	0,175	202,885	61,398
TMED5	0,002	0,459	0,729	0,104	0,095	-0,121	1149,406	113,715
ACTL6A	0,001	0,458	0,729	0,454	0,426	-0,089	659,98	289,892
APC	0,003	0,457	0,729	0,053	0,059	0,169	974,712	54,594
DOCK5	0,001	0,458	0,729	0,095	0,102	0,108	1481,166	146,363
MAPK8	0,001	0,458	0,729	0,151	0,163	0,114	867,177	136,285
ISCU	0,003	0,458	0,729	0,184	0,207	0,17	313,458	60,942
ZNF317	0,002	0,457	0,729	0,107	0,119	0,152	635,913	71,643
CHM	0,002	0,457	0,729	0,184	0,167	-0,137	540,267	94,608
NUP205	0,001	0,46	0,73	0,168	0,159	-0,082	1982,978	323,03
TBC1D31	0,002	0,459	0,73	0,198	0,219	0,149	383,148	80,21
LTBR	0,001	0,459	0,73	0,222	0,235	0,08	1447,647	329,59
TENC1	0,003	0,46	0,73	0,05	0,044	-0,187	942,013	44,244
UBR7	0,002	0,46	0,73	0,098	0,089	-0,14	889,408	83,111
NFIX	0,003	0,459	0,73	0,041	0,046	0,188	1107,035	47,139

DNA2	0,002	0,46	0,731	0,231	0,255	0,142	368,557	89,83
API5	0,001	0,461	0,731	0,135	0,146	0,114	950,424	133,426
C12orf4	0,002	0,461	0,731	0,137	0,123	-0,151	563,572	72,573
RAB5B	0,002	0,461	0,731	0,624	0,713	0,192	107,464	70,081
NFKBIA	0,002	0,461	0,731	0,327	0,364	0,156	233,084	79,912
SETD3	0,002	0,461	0,731	0,101	0,091	-0,141	854,761	81,679
TOM1L1	0,002	0,461	0,731	0,304	0,336	0,145	285,067	90,933
NDUFB3	0,001	0,464	0,732	1,163	1,273	0,13	145,455	176,781
NR1D2	0,002	0,462	0,732	0,14	0,128	-0,129	763,13	102,277
P4HTM	0,002	0,463	0,732	0,336	0,37	0,138	292,53	102,836
ABCE1	0,001	0,462	0,732	0,161	0,151	-0,093	1350,131	209,504
RPP40	0,003	0,462	0,732	0,634	0,55	-0,206	86,34	51,069
C6orf62	0,002	0,463	0,732	0,103	0,095	-0,119	1197,927	118,281
FBXL18	0,002	0,463	0,732	0,36	0,329	-0,127	355,01	122,743
ASL	0,002	0,462	0,732	0,405	0,456	0,172	156,288	67,242
SART1	0,002	0,462	0,732	0,086	0,079	-0,12	1415,108	116,316
PPP1CA	0,001	0,463	0,732	0,58	0,557	-0,059	1637,833	931,666
SIK2	0,002	0,463	0,732	0,094	0,104	0,151	711,766	70,343
FGFR1OP2	0,003	0,463	0,732	0,196	0,221	0,174	268,598	56,148
JMJD6	0,002	0,462	0,732	0,236	0,259	0,133	416,909	102,744
PXN	0,001	0,464	0,733	0,147	0,154	0,07	2987,009	447,465
MITD1	0,002	0,465	0,734	0,354	0,392	0,148	236,219	87,997
NUMB	0,002	0,465	0,734	0,094	0,103	0,131	964,14	95,148
MEAF6	0,002	0,465	0,735	0,29	0,326	0,166	221,267	67,545
MTF2	0,003	0,467	0,735	0,117	0,131	0,17	448,406	56,174
ENSG00000270066.2	0,003	0,467	0,735	5,175	4,139	-0,322	13,759	64,051
PTCD3	0,001	0,467	0,735	0,177	0,165	-0,098	1069,127	182,4
UTP15	0,002	0,466	0,735	0,197	0,216	0,134	468,939	97,246
SCD	0,001	0,466	0,735	0,266	0,283	0,088	1028,322	280,032
PCGF6	0,003	0,467	0,735	0,434	0,499	0,199	106,276	49,684
TPCN2	0,002	0,467	0,735	0,409	0,363	-0,173	164,182	63,279
SLC8B1	0,002	0,466	0,735	0,242	0,219	-0,142	398,365	92,498

GUCD1	0,002	0,467	0,735	0,074	0,066	-0,158	891,395	62,395
TRMU	0,002	0,467	0,735	0,079	0,071	-0,147	953,866	71,732
MAX	0,002	0,468	0,736	0,168	0,152	-0,144	496,841	79,044
YEATS2	0,002	0,468	0,737	0,098	0,108	0,153	657,59	67,559
ENTPD4	0,003	0,469	0,737	0,065	0,057	-0,174	808,51	49,046
EIF3F	0,001	0,468	0,737	0,643	0,613	-0,07	889,112	558,72
TMEM126A	0,001	0,47	0,738	0,919	1,006	0,13	159,304	153,336
TMEM19	0,003	0,47	0,738	0,146	0,166	0,18	315,5	49,575
DNAJC3	0,001	0,469	0,738	0,173	0,186	0,108	826,095	148,749
MRI1	0,002	0,469	0,738	0,465	0,419	-0,151	195,679	86,701
ZC3H7B	0,002	0,47	0,738	0,048	0,044	-0,121	2284,972	106,123
GCA	0,002	0,472	0,739	0,872	0,776	-0,168	102,083	84,127
ABCF3	0,002	0,472	0,739	0,192	0,211	0,138	448,026	89,601
TMED4	0,001	0,472	0,739	0,37	0,349	-0,085	805,974	290,04
ENSG00000233016.2	0,002	0,472	0,739	0,313	0,284	-0,139	313,165	92,827
ENO2	0,003	0,472	0,739	0,372	0,325	-0,194	142,262	49,956
UBE3B	0,003	0,472	0,739	0,102	0,09	-0,174	522,147	50,106
EIF2B1	0,001	0,473	0,739	0,32	0,299	-0,098	644,723	199,262
DCLK1	0,002	0,47	0,739	0,144	0,13	-0,153	493,734	67,657
ALG1	0,002	0,473	0,739	0,896	0,813	-0,141	143,096	122,231
PGS1	0,002	0,471	0,739	0,219	0,195	-0,167	296,565	61,906
SLC14A1	0,002	0,472	0,739	0,387	0,345	-0,165	188,242	69,241
SMAD2	0,003	0,471	0,739	0,15	0,133	-0,17	382,903	53,846
C19orf24	0,002	0,471	0,739	0,289	0,263	-0,134	360,595	99,665
YDJC	0,002	0,473	0,739	0,284	0,314	0,145	281,539	83,808
CBX6	0,002	0,471	0,739	0,063	0,058	-0,123	1689,053	101,534
NDUFA12	0,001	0,473	0,74	0,948	1,011	0,092	331,947	324,668
ZNF462	0,003	0,474	0,741	0,062	0,07	0,165	836,804	54,716
MESDC2	0,002	0,474	0,741	0,216	0,237	0,129	470,797	105,706
UBE2M	0,001	0,474	0,741	0,377	0,4	0,086	719,986	278,924
SEPN1	0,002	0,476	0,742	0,067	0,061	-0,142	1170,477	75,552
CREG1	0,001	0,475	0,742	0,432	0,4	-0,111	383,125	158,641

SLC35F6	0,001	0,476	0,742	0,271	0,255	-0,088	927,609	243,341
AUP1	0,001	0,476	0,742	0,248	0,238	-0,059	2760,086	670,096
MAP3K7	0,002	0,475	0,742	0,179	0,196	0,128	532,867	100,181
NHLRC2	0,001	0,476	0,742	0,13	0,14	0,11	1008,198	136,464
PAFAH1B2	0,001	0,475	0,742	0,227	0,24	0,081	1232,966	287,211
LATS2	0,002	0,476	0,742	0,089	0,098	0,14	817,579	76,893
GNPDA1	0,001	0,477	0,743	0,251	0,272	0,114	518,462	135,595
ZNF367	0,002	0,477	0,743	0,16	0,144	-0,15	477,893	73,214
CRAT	0,002	0,477	0,743	0,186	0,205	0,141	424,294	82,426
ARL5B	0,003	0,477	0,743	0,098	0,087	-0,176	508,667	46,816
COX10	0,002	0,477	0,743	0,176	0,162	-0,122	659,426	111,558
WSB1	0,002	0,476	0,743	0,171	0,187	0,124	602,173	107,477
MED13	0,002	0,477	0,743	0,072	0,078	0,112	1638,935	122,8
ATP8B3	0,003	0,477	0,743	0,235	0,266	0,176	217,765	54,133
ZC4H2	0,003	0,477	0,743	0,229	0,2	-0,191	204,839	43,834
C1orf174	0,002	0,479	0,744	0,346	0,311	-0,154	225,867	73,947
PCBP1	0,001	0,479	0,744	0,411	0,395	-0,056	2284,98	920,417
EDIL3	0,002	0,479	0,744	0,379	0,347	-0,128	307,908	111,384
CSNK1A1	0,001	0,478	0,744	0,434	0,415	-0,067	1283,978	544,268
PPP2R5D	0,001	0,478	0,744	0,119	0,127	0,09	1728,28	212,725
DNM1	0,003	0,479	0,744	0,167	0,188	0,172	302,534	53,115
DOLPP1	0,002	0,48	0,745	0,214	0,233	0,126	470,766	105,103
TOP3A	0,001	0,48	0,745	0,12	0,128	0,088	1697,673	210,846
ELMO2	0,002	0,48	0,745	0,12	0,132	0,141	605,346	76,211
SRSF4	0,002	0,483	0,746	0,11	0,12	0,127	827,659	95,767
GPSM2	0,001	0,483	0,746	0,239	0,257	0,107	606,258	149,989
NRAS	0,001	0,481	0,746	0,111	0,104	-0,093	1701,963	182,746
KCMF1	0,001	0,484	0,746	0,146	0,139	-0,079	1945,791	276,758
SLC26A6	0,002	0,484	0,746	0,252	0,23	-0,131	399,817	96,345
BAP1	0,002	0,481	0,746	0,078	0,072	-0,117	1462,478	109,566
SLC35A5	0,002	0,482	0,746	0,32	0,291	-0,14	286,684	86,855
RABL3	0,003	0,482	0,746	0,187	0,164	-0,188	247,676	43,733

MFAP3	0,002	0,484	0,746	0,205	0,223	0,119	532,996	114,899
ARHGEF10	0,002	0,481	0,746	0,136	0,122	-0,156	492,133	63,932
DERL1	0,001	0,483	0,746	0,373	0,351	-0,085	742,434	268,212
CREB3	0,002	0,481	0,746	0,262	0,285	0,12	435,265	118,91
EXOSC2	0,001	0,483	0,746	0,395	0,418	0,082	739,478	300,041
DPP7	0,001	0,483	0,746	0,221	0,206	-0,1	802,141	171,865
CAPN1	0,001	0,481	0,746	0,17	0,16	-0,087	1439,285	237,993
UNC93B1	0,002	0,482	0,746	0,443	0,403	-0,137	232,842	98,02
CD63	0,001	0,484	0,746	0,695	0,722	0,055	1498,854	1060,038
ANAPC7	0,002	0,481	0,746	0,169	0,155	-0,126	611,786	99,094
TOX4	0,001	0,483	0,746	0,232	0,25	0,109	587,629	141,9
MTHFD1	0,001	0,484	0,746	0,138	0,145	0,072	2440,15	345,097
EMP2	0,001	0,483	0,746	0,338	0,362	0,103	500,793	174,815
TAX1BP3	0,003	0,481	0,746	0,54	0,474	-0,187	106,664	53,716
STAU1	0,001	0,483	0,746	0,073	0,078	0,107	1702,147	128,139
SH3BGRL	0,001	0,482	0,746	0,327	0,303	-0,108	490,268	154,8
BCAP31	0,001	0,483	0,746	0,737	0,805	0,127	184,509	141,683
LUZP1	0,003	0,485	0,747	0,042	0,047	0,162	1154,251	51,495
RPS8	0,001	0,485	0,747	0,667	0,644	-0,051	1975,615	1294,781
LRRC8D	0,001	0,486	0,747	0,175	0,163	-0,107	817,635	137,757
EML4	0,002	0,484	0,747	0,063	0,07	0,15	933,941	62,117
RYBP	0,003	0,486	0,747	0,09	0,08	-0,167	588,973	50,182
SCRN1	0,001	0,485	0,747	0,064	0,068	0,083	3412,395	226,981
TACC1	0,001	0,485	0,747	0,096	0,102	0,085	2268,621	224,849
TPD52	0,002	0,485	0,747	0,171	0,158	-0,113	753,254	123,632
CIZ1	0,001	0,486	0,747	0,139	0,131	-0,081	2013,576	272,688
ENSG00000255717.2	0,001	0,486	0,747	2,38	2,305	-0,046	1384,525	3237,968
IFI27L1	0,003	0,485	0,747	0,822	0,718	-0,195	76,943	59,971
PRR14	0,003	0,485	0,747	0,12	0,135	0,172	386,068	49,273
NAE1	0,001	0,486	0,747	0,317	0,34	0,102	525,022	172,743
FKBP10	0,003	0,486	0,747	0,241	0,211	-0,19	192,55	43,814
CEP76	0,003	0,486	0,747	0,427	0,376	-0,181	131,968	52,615

EEF1A2	0,003	0,486	0,747	0,459	0,518	0,176	121,218	59,415
NOL12	0,002	0,486	0,747	0,318	0,288	-0,142	276,765	83,916
MED12	0,002	0,485	0,747	0,132	0,143	0,113	896,886	122,897
ZNF330	0,002	0,487	0,748	0,245	0,223	-0,136	367,018	85,858
FOXN2	0,002	0,488	0,749	0,135	0,15	0,157	411,485	58,921
NOP16	0,001	0,488	0,749	0,329	0,352	0,098	550,606	187,356
QKI	0,001	0,489	0,749	0,136	0,144	0,083	1667,57	233,216
ELOF1	0,002	0,489	0,749	0,288	0,263	-0,133	337,528	93,286
ZNF274	0,002	0,488	0,749	0,269	0,295	0,13	342,142	96,313
GGA1	0,001	0,488	0,749	0,162	0,151	-0,099	1016,741	159,378
KIF15	0,002	0,49	0,75	0,114	0,124	0,121	832,108	99,166
SLU7	0,002	0,49	0,75	0,118	0,131	0,142	572,186	71,242
FPGS	0,001	0,49	0,75	0,301	0,283	-0,087	826,097	241,607
PPRC1	0,001	0,49	0,75	0,108	0,102	-0,085	2026,749	212,753
FBXO21	0,002	0,49	0,75	0,182	0,165	-0,137	459,557	79,6
COX6A1	0,002	0,49	0,75	0,453	0,409	-0,147	189,821	81,37
TNRC6A	0,002	0,49	0,75	0,069	0,062	-0,152	900,773	58,766
GAA	0,001	0,491	0,75	0,295	0,278	-0,084	923,236	265,905
PIAS4	0,002	0,489	0,75	0,225	0,206	-0,13	439,317	94,983
TOP1	0,001	0,491	0,75	0,05	0,053	0,099	2837,378	146,031
SELM	0,002	0,49	0,75	0,376	0,42	0,162	168,479	66,524
TMEM164	0,002	0,49	0,75	0,075	0,084	0,154	734,816	58,109
MFSD6	0,003	0,493	0,751	0,115	0,103	-0,159	503,429	54,626
NDUFAF3	0,001	0,493	0,751	0,731	0,673	-0,118	218,354	153,785
PLAU	0,001	0,493	0,751	0,406	0,387	-0,069	1094,039	433,645
FAM178A	0,002	0,492	0,751	0,054	0,059	0,139	1239,197	70,192
MAPK3	0,002	0,492	0,751	0,255	0,28	0,134	341,883	90,813
STAT3	0,001	0,491	0,751	0,186	0,177	-0,066	2665,193	483,055
SPOP	0,002	0,492	0,751	0,197	0,181	-0,12	572,071	108,324
SLC44A2	0,001	0,492	0,751	0,187	0,196	0,071	1949,608	373,923
PITPNB	0,001	0,492	0,751	0,44	0,406	-0,114	329,286	139,074
EIF2B3	0,001	0,493	0,752	0,336	0,313	-0,103	501,345	162,578

YY1AP1	0,003	0,495	0,752	0,135	0,152	0,166	359,192	51,407
B3GALNT2	0,003	0,493	0,752	0,196	0,175	-0,171	271,888	50,488
FHL2	0,001	0,495	0,752	0,504	0,533	0,079	677,193	348,802
ASNSD1	0,002	0,495	0,752	0,191	0,177	-0,111	670,457	123,151
PYCRL	0,002	0,495	0,752	0,229	0,206	-0,152	296,998	64,444
TMEM38B	0,001	0,495	0,752	0,474	0,508	0,1	380,014	186,985
NDOR1	0,003	0,495	0,752	0,092	0,082	-0,156	650,111	56,788
FOXM1	0,001	0,494	0,752	0,052	0,055	0,085	3698,212	199,206
NUP107	0,001	0,494	0,752	0,168	0,18	0,099	913,734	159,488
TMED2	0,001	0,493	0,752	0,134	0,141	0,077	1970,356	272,198
VPS36	0,002	0,495	0,752	0,107	0,118	0,136	665,078	74,897
CINP	0,002	0,495	0,752	0,355	0,326	-0,121	338,177	114,807
GALNS	0,002	0,494	0,752	0,221	0,246	0,157	270,285	62,721
WDR62	0,001	0,493	0,752	0,149	0,159	0,093	1171,244	180,594
WRB	0,003	0,495	0,752	0,482	0,424	-0,182	114,515	51,526
CBX1	0,002	0,496	0,753	0,121	0,111	-0,118	882,145	101,703
WWTR1	0,002	0,497	0,754	0,156	0,144	-0,108	848,8	127,288
SKIV2L2	0,002	0,497	0,754	0,124	0,115	-0,114	928,542	110,66
BPTF	0,002	0,497	0,754	0,078	0,071	-0,15	796,329	59,194
HADHB	0,001	0,499	0,755	0,261	0,243	-0,104	581,56	146,349
MTMR14	0,002	0,499	0,755	0,234	0,214	-0,125	431,802	96,466
ATP6V1F	0,002	0,499	0,755	0,331	0,304	-0,126	319,376	101,568
UBE2H	0,003	0,499	0,755	0,105	0,117	0,153	520,812	57,782
CHMP7	0,002	0,498	0,755	0,131	0,142	0,122	690,735	94,386
ATP6V1G1	0,001	0,499	0,755	0,362	0,382	0,077	865,782	321,201
AKT1	0,001	0,498	0,755	0,102	0,096	-0,089	1861,093	184,716
HEXA	0,003	0,499	0,755	0,313	0,351	0,165	174,136	58,027
TUFM	0,001	0,498	0,755	0,376	0,362	-0,055	2180,573	805,346
PAPD5	0,003	0,498	0,755	0,184	0,164	-0,161	315,224	54,589
CLSPN	0,002	0,5	0,756	0,1	0,109	0,121	890,368	93,242
WDFY3	0,002	0,5	0,756	0,066	0,072	0,136	1043,856	72,125
SNX14	0,002	0,5	0,756	0,169	0,184	0,128	498,482	88,315

SLC39A14	0,001	0,5	0,756	0,308	0,296	-0,061	1841,381	554,623
DDX21	0,001	0,5	0,756	0,052	0,055	0,067	6937,227	373,006
TUBGCP3	0,001	0,5	0,756	0,225	0,211	-0,092	874,755	190,804
EDEM3	0,002	0,501	0,757	0,133	0,148	0,151	443,161	61,8
SDC1	0,001	0,501	0,757	0,246	0,259	0,074	1273,626	321,455
CLOCK	0,003	0,502	0,757	0,062	0,055	-0,159	866,264	50,762
TAF8	0,002	0,501	0,757	0,188	0,206	0,138	385,948	76,173
CHAF1A	0,001	0,502	0,757	0,141	0,133	-0,094	1213,284	165,969
PGLS	0,001	0,502	0,757	0,345	0,368	0,095	539,457	191,789
ENSG00000222041.6	0,002	0,503	0,758	0,318	0,351	0,146	222,314	74,001
SMPD4	0,001	0,503	0,758	0,162	0,151	-0,104	890,727	139,897
COL8A1	0,001	0,503	0,758	0,233	0,25	0,101	669,505	160,099
SPIN1	0,002	0,504	0,758	0,079	0,085	0,11	1380,058	113,161
FBXW2	0,002	0,503	0,758	0,052	0,058	0,147	1063,796	58,523
MSRB2	0,004	0,503	0,758	0,682	0,577	-0,24	54,295	34,813
TUBA1B	0,001	0,503	0,758	0,297	0,311	0,068	1393,593	421,857
TRMT5	0,002	0,503	0,758	0,178	0,195	0,137	409,385	76,647
DHX33	0,001	0,502	0,758	0,122	0,115	-0,086	1757,002	208,059
UBTF	0,002	0,503	0,758	0,036	0,04	0,126	2200,587	83,342
UBE2O	0,002	0,502	0,758	0,154	0,166	0,11	746,845	119,316
UBA2	0,001	0,503	0,758	0,208	0,222	0,094	815,083	175,68
MPHOSPH10	0,002	0,505	0,759	0,126	0,116	-0,126	716,855	86,397
ORC2	0,002	0,505	0,759	0,177	0,192	0,116	577,685	106,606
EIF4E2	0,002	0,506	0,759	0,14	0,127	-0,142	520,617	68,369
PDCD6IP	0,001	0,507	0,759	0,163	0,17	0,065	2438,136	407,188
CAST	0,001	0,507	0,759	0,152	0,16	0,073	1932,659	300,579
ENSG00000227706.3	0,002	0,506	0,759	0,27	0,294	0,126	341,053	96,866
AIG1	0,002	0,507	0,759	0,449	0,49	0,128	225,5	105,399
RBM18	0,002	0,507	0,759	0,213	0,195	-0,13	416,925	85,058
ZDHHC5	0,001	0,505	0,759	0,086	0,092	0,091	2006,916	178,893
NAA25	0,002	0,506	0,759	0,116	0,125	0,111	925,621	111,639
MICU2	0,001	0,506	0,759	0,287	0,27	-0,092	674,116	187,637

NOP10	0,001	0,507	0,759	1,105	1,172	0,084	327,916	372,093
SNAP23	0,001	0,505	0,759	0,422	0,454	0,107	345,49	151,796
ZNF207	0,001	0,505	0,759	0,15	0,158	0,077	1708,377	263,295
TACO1	0,002	0,506	0,759	0,302	0,328	0,118	358,657	112,863
CDC42EP4	0,002	0,506	0,759	0,147	0,136	-0,117	719,335	101,488
SIRT7	0,003	0,507	0,759	0,138	0,123	-0,171	350,584	46,005
B3GNTL1	0,002	0,507	0,759	0,17	0,187	0,135	429,682	76,446
MCOLN1	0,001	0,506	0,759	0,553	0,595	0,105	296,222	169,358
ZNF121	0,002	0,506	0,759	0,097	0,107	0,141	642,541	65,6
MBOAT7	0,002	0,506	0,759	0,497	0,454	-0,13	231,798	111,454
PHF6	0,002	0,507	0,759	0,114	0,125	0,133	640,015	77,365
TMEM59	0,001	0,508	0,76	0,676	0,645	-0,067	768,527	507,431
GDAP2	0,002	0,508	0,76	0,239	0,262	0,135	318,779	80,336
RAB11FIP5	0,002	0,509	0,76	0,05	0,046	-0,121	1815,76	88,348
TMEM185B	0,001	0,508	0,76	0,419	0,452	0,109	322,771	140,542
NDUFA10	0,001	0,508	0,76	0,335	0,353	0,076	891,587	306,52
RARS2	0,003	0,509	0,76	0,287	0,321	0,16	190,375	58,295
SHARPIN	0,002	0,509	0,76	0,261	0,285	0,125	345,718	94,402
ANXA11	0,001	0,508	0,76	0,243	0,256	0,074	1268,8	316,581
CHSY1	0,002	0,509	0,76	0,132	0,12	-0,141	533,382	66,643
PIN4	0,003	0,508	0,76	0,489	0,548	0,166	118,998	61,64
ETFA	0,001	0,51	0,761	0,791	0,752	-0,073	553,218	426,567
FAF1	0,001	0,512	0,762	0,246	0,233	-0,078	1079,77	258,646
IFI44	0,003	0,511	0,762	0,234	0,26	0,154	241,46	59,912
XPR1	0,002	0,512	0,762	0,117	0,128	0,126	672,836	82,176
POLR1B	0,002	0,511	0,762	0,188	0,173	-0,116	587,711	105,199
DNAJC10	0,001	0,511	0,762	0,142	0,151	0,087	1280,286	188,337
CAPRIN1	0,001	0,512	0,762	0,108	0,112	0,053	6008,889	663,331
STX5	0,002	0,511	0,762	0,303	0,327	0,112	385,72	121,255
NEU3	0,003	0,512	0,762	0,162	0,181	0,165	281,789	48,313
TMEM135	0,003	0,512	0,762	0,276	0,247	-0,16	212,78	55,481
CBX5	0,002	0,512	0,763	0,05	0,046	-0,108	2238,839	107,428

SOX12	0,003	0,513	0,763	0,181	0,16	-0,177	245,677	42,121
ERI3	0,001	0,515	0,764	0,428	0,402	-0,089	516,187	214,245
KLHL23	0,003	0,514	0,764	0,15	0,135	-0,154	378,061	53,88
SLC38A9	0,003	0,514	0,764	0,374	0,421	0,172	128,328	51,136
MRPS10	0,002	0,514	0,764	0,284	0,263	-0,113	413,091	112,334
FYN	0,003	0,514	0,764	0,146	0,131	-0,164	346,308	48,006
TAF10	0,001	0,514	0,764	0,429	0,4	-0,104	370,128	153,775
MICAL2	0,001	0,513	0,764	0,064	0,06	-0,098	2248,078	140,155
MYRF	0,003	0,515	0,764	0,108	0,096	-0,16	475,84	48,665
MTMR6	0,001	0,515	0,764	0,21	0,198	-0,084	1034,825	210,5
C18orf8	0,003	0,515	0,764	0,279	0,251	-0,156	216,92	57,213
SLMO2	0,001	0,515	0,764	0,185	0,195	0,075	1424,325	270,539
CDR1	0,004	0,515	0,764	0,764	0,646	-0,242	50,869	37,008
ACOT7	0,001	0,517	0,765	0,431	0,455	0,081	605,143	267,532
PRKACB	0,002	0,516	0,765	0,18	0,167	-0,115	581,386	100,882
PRCC	0,001	0,516	0,765	0,217	0,23	0,085	939,527	209,805
TIPRL	0,001	0,518	0,765	0,389	0,413	0,084	580,684	232,971
VGLL4	0,003	0,516	0,765	0,203	0,18	-0,17	240,41	46,204
NFKB1	0,002	0,516	0,765	0,11	0,12	0,132	633,377	72,871
STK38	0,002	0,515	0,765	0,087	0,094	0,118	984,77	89,461
LAMTOR4	0,001	0,516	0,765	1,261	1,15	-0,133	109,457	132,51
PPP3CB	0,001	0,517	0,765	0,242	0,226	-0,097	656,063	153,27
DPAGT1	0,001	0,517	0,765	0,46	0,435	-0,082	577,927	258,376
ARHGAP11A	0,002	0,517	0,765	0,059	0,054	-0,117	1589,239	90,06
HAUS2	0,002	0,518	0,765	0,238	0,259	0,121	385,774	96,161
IL4R	0,003	0,516	0,765	0,119	0,107	-0,151	480,504	54,304
MLKL	0,003	0,517	0,765	0,121	0,108	-0,167	393,482	45,252
PAFAH1B1	0,001	0,516	0,765	0,144	0,137	-0,077	1763,115	246,363
PIN1	0,001	0,516	0,765	0,3	0,282	-0,09	673,245	196,338
NACC1	0,001	0,517	0,765	0,1	0,107	0,09	1648,805	170,279
IDH3B	0,001	0,518	0,765	0,435	0,455	0,066	959,13	427,383
DDX56	0,001	0,519	0,766	0,317	0,331	0,064	1337,643	434,048

MOB1A	0,001	0,52	0,767	0,155	0,149	-0,054	4209,294	638,443
ENSG00000143429.5	0,003	0,52	0,767	1,007	1,163	0,209	45,528	48,752
MGLL	0,002	0,521	0,767	0,068	0,063	-0,116	1386,188	90,424
ADD1	0,001	0,519	0,767	0,133	0,141	0,088	1305,24	179,55
PTAR1	0,003	0,519	0,767	0,069	0,063	-0,152	777,858	50,916
HSDL2	0,001	0,521	0,767	0,215	0,201	-0,095	764,297	159,461
ARRDC1	0,003	0,52	0,767	0,192	0,215	0,161	247,791	50,236
RTN3	0,001	0,52	0,767	0,431	0,41	-0,071	861,186	362,158
RAD51AP1	0,002	0,52	0,767	0,24	0,221	-0,118	450,905	102,795
CS	0,001	0,52	0,767	0,196	0,207	0,079	1163,456	234,716
OSBPL8	0,002	0,519	0,767	0,102	0,095	-0,105	1198,725	117,125
MVP	0,001	0,521	0,767	0,213	0,201	-0,082	1092,293	227,092
GYS1	0,002	0,519	0,767	0,096	0,105	0,124	808,513	81,133
YIPF1	0,002	0,522	0,768	0,421	0,386	-0,124	249,862	101,236
SLC25A24	0,001	0,522	0,768	0,231	0,219	-0,077	1145,718	256,984
DPM3	0,002	0,523	0,768	2,2	1,955	-0,171	48,957	101,841
LMNA	0,001	0,522	0,768	0,182	0,187	0,043	6357,634	1170,245
CCT4	0,001	0,524	0,768	0,518	0,504	-0,039	6259,312	3200,123
SETD7	0,002	0,523	0,768	0,05	0,046	-0,117	1804,262	86,034
TOR1B	0,002	0,522	0,768	0,267	0,247	-0,114	425,836	109,908
CEP55	0,001	0,523	0,768	0,123	0,129	0,07	2262,309	285,83
TPP2	0,001	0,523	0,768	0,154	0,163	0,081	1359,98	215,648
CUL4A	0,001	0,523	0,768	0,245	0,255	0,06	1972,802	493,532
AQR	0,003	0,524	0,768	0,079	0,072	-0,142	794,498	59,408
RPUSD1	0,002	0,522	0,768	0,119	0,108	-0,143	528,619	59,798
POLR2C	0,001	0,521	0,768	0,397	0,377	-0,073	861,512	331,897
DDX42	0,001	0,523	0,768	0,088	0,093	0,089	1815,905	164,212
STK11	0,003	0,522	0,768	0,06	0,066	0,148	850,886	53,675
FLAD1	0,002	0,526	0,769	0,268	0,246	-0,121	359,773	92,417
HADHA	0,001	0,525	0,769	0,166	0,176	0,083	1164,671	199,119
MGAT5	0,002	0,526	0,769	0,095	0,103	0,124	780,305	77,487
NFE2L2	0,001	0,526	0,769	0,267	0,255	-0,066	1410,233	368,871

SGOL1	0,002	0,527	0,769	0,157	0,173	0,14	383,729	63,418
GMPS	0,001	0,526	0,769	0,192	0,183	-0,076	1329,95	249,337
SRA1	0,001	0,524	0,769	0,469	0,436	-0,102	332,73	150,355
TBRG4	0,001	0,525	0,769	0,303	0,29	-0,066	1247,912	369,702
TMEM70	0,001	0,525	0,769	0,664	0,619	-0,102	262,779	168,206
AP3M1	0,001	0,525	0,769	0,153	0,162	0,087	1133,311	178,883
NOLC1	0,001	0,527	0,769	0,067	0,07	0,057	6409,018	437,389
PDCD11	0,003	0,525	0,769	0,034	0,037	0,151	1378,042	48,899
RAB6A	0,002	0,526	0,769	0,143	0,155	0,115	629,703	94,031
NCAPD3	0,001	0,524	0,769	0,095	0,089	-0,09	1694,545	155,142
CKAP4	0,001	0,525	0,769	0,179	0,187	0,069	1866,005	340,747
SUGT1	0,001	0,524	0,769	0,322	0,303	-0,085	684,142	213,488
PPP2R5E	0,002	0,526	0,769	0,121	0,131	0,114	754,266	94,943
ANKFY1	0,001	0,526	0,769	0,102	0,107	0,078	2117,717	221,338
THOC1	0,002	0,525	0,769	0,25	0,269	0,105	484,907	126,243
ACTR5	0,002	0,524	0,769	0,334	0,367	0,136	219,606	77,105
CACYBP	0,001	0,527	0,77	0,167	0,178	0,099	774,629	133,64
MTDH	0,001	0,527	0,77	0,102	0,107	0,069	2877,741	299,505
ARFIP2	0,001	0,528	0,77	0,28	0,296	0,079	851,105	244,163
FAU	0,001	0,528	0,77	0,992	1,025	0,047	1344,787	1355,708
NPTN	0,001	0,529	0,77	0,364	0,385	0,084	608,804	226,808
DBF4B	0,002	0,529	0,77	0,086	0,094	0,13	763,125	68,857
ADNP2	0,003	0,528	0,77	0,127	0,114	-0,155	407,167	48,944
SPC24	0,001	0,528	0,77	0,498	0,462	-0,107	288,035	138,774
TBCB	0,001	0,528	0,77	0,58	0,548	-0,083	466,557	263,481
SMARCA1	0,003	0,529	0,77	0,124	0,138	0,151	397,167	52,422
HSPG2	0,001	0,531	0,773	0,145	0,154	0,087	1347,552	199,372
NDRG1	0,001	0,531	0,773	0,247	0,23	-0,1	550,016	130,624
COPB1	0,001	0,531	0,773	0,171	0,164	-0,065	2119,109	354,187
ADCY9	0,003	0,531	0,773	0,097	0,107	0,144	549,344	55,827
LSM14B	0,001	0,532	0,773	0,285	0,265	-0,102	473,785	130,229
KIF2A	0,002	0,533	0,774	0,139	0,15	0,104	803,163	116,205

FSCN1	0,003	0,532	0,774	0,277	0,315	0,186	147,149	42,488
TGIF1	0,002	0,532	0,774	0,341	0,374	0,132	221,017	79,099
TAF5	0,002	0,533	0,775	0,32	0,295	-0,117	327,785	100,446
KPNA1	0,002	0,535	0,776	0,122	0,134	0,128	551,896	70,767
AFF4	0,002	0,534	0,776	0,054	0,058	0,097	2288,406	127,738
CYSTM1	0,002	0,534	0,776	0,694	0,638	-0,123	167,391	111,81
GEM	0,004	0,534	0,776	0,229	0,202	-0,178	181,569	39,289
TFAP2C	0,001	0,534	0,776	0,272	0,254	-0,099	535,831	139,697
PLK4	0,002	0,535	0,777	0,105	0,116	0,137	545,803	60,316
ALDH18A1	0,001	0,535	0,777	0,161	0,17	0,076	1379,211	228,746
AKR1A1	0,001	0,538	0,778	0,385	0,411	0,096	393,025	156,642
CDC42BPA	0,002	0,537	0,778	0,045	0,05	0,141	1141,109	53,9
MRPL19	0,001	0,537	0,778	0,318	0,304	-0,065	1167,997	362,707
COQ10B	0,002	0,537	0,778	0,49	0,447	-0,133	174,901	81,592
AGAP1	0,003	0,538	0,778	0,054	0,048	-0,151	906,189	46,245
TMEM165	0,001	0,538	0,778	0,431	0,408	-0,076	630,702	264,315
ATP5J2	0,001	0,536	0,778	0,485	0,518	0,094	360,435	180,121
GSN	0,002	0,537	0,778	0,293	0,272	-0,111	400,832	114,193
CLPX	0,002	0,538	0,778	0,192	0,177	-0,116	492,317	90,318
SIN3A	0,001	0,536	0,778	0,184	0,195	0,083	1004,886	190,6
TMEM92	0,004	0,536	0,778	0,288	0,254	-0,177	149,673	40,667
LRRC59	0,001	0,537	0,778	0,227	0,234	0,047	4158,432	958,59
CCBE1	0,002	0,537	0,778	0,113	0,124	0,136	524,705	61,92
DNAJB1	0,001	0,538	0,778	0,252	0,241	-0,069	1310,052	322,623
TMEM50A	0,001	0,54	0,779	0,422	0,4	-0,076	643,534	264,15
TMEM69	0,001	0,543	0,779	0,292	0,275	-0,088	629,894	178,945
SH3GLB1	0,001	0,539	0,779	0,23	0,219	-0,069	1345,979	302,277
BCAR3	0,001	0,543	0,779	0,136	0,143	0,075	1621,961	225,989
SNX27	0,003	0,541	0,779	0,095	0,086	-0,143	595,36	53,685
FDPS	0,001	0,541	0,779	0,453	0,425	-0,092	405,698	178,492
SEP10	0,002	0,54	0,779	0,154	0,143	-0,104	739,63	109,698
GLS	0,001	0,54	0,779	0,203	0,191	-0,089	847,925	166,084

PPP1R7	0,001	0,54	0,779	0,595	0,558	-0,094	315,037	181,933
ARIH2	0,002	0,542	0,779	0,087	0,093	0,101	1235,557	111,478
RPL29	0,001	0,542	0,779	0,363	0,38	0,065	1040,904	385,895
HPS3	0,002	0,539	0,779	0,213	0,196	-0,119	431,541	88,431
SGMS2	0,003	0,542	0,779	0,112	0,125	0,155	385,527	45,565
PLK2	0,001	0,54	0,779	0,183	0,191	0,063	1924,972	359,567
TRAPPC13	0,003	0,539	0,779	0,311	0,28	-0,15	193,831	57,323
NUP43	0,001	0,542	0,779	0,333	0,316	-0,077	737,493	238,484
MALSU1	0,002	0,541	0,779	0,599	0,553	-0,117	194,034	111,531
UPP1	0,001	0,542	0,779	0,354	0,379	0,101	371,314	135,759
CUL1	0,001	0,541	0,779	0,206	0,193	-0,093	730,671	145,887
DCTN6	0,002	0,54	0,779	0,38	0,344	-0,143	179,42	64,692
RAD21	0,001	0,539	0,779	0,144	0,15	0,057	3525,411	520,723
ENSG00000233137.2	0,003	0,539	0,779	0,128	0,116	-0,143	449,896	54,571
PPP2R4	0,001	0,54	0,779	0,212	0,223	0,071	1319,836	286,917
LDLRAD3	0,002	0,542	0,779	0,109	0,1	-0,124	705,627	74,046
ST3GAL4	0,003	0,54	0,779	0,094	0,104	0,149	499,189	49,197
EMP1	0,001	0,539	0,779	0,31	0,323	0,06	1474,632	466,399
DDX23	0,003	0,54	0,779	0,054	0,059	0,135	1025,294	57,919
RAB21	0,001	0,543	0,779	0,269	0,252	-0,095	548,637	142,313
RBM23	0,002	0,539	0,779	0,235	0,252	0,104	483,942	117,771
PPAP2C	0,001	0,54	0,779	0,617	0,661	0,1	257,626	164,043
RUVBL2	0,001	0,541	0,779	0,405	0,392	-0,049	2044,704	815,4
EIF6	0,001	0,543	0,779	0,444	0,459	0,046	2207,84	995,25
ARAF	0,002	0,542	0,779	0,125	0,135	0,115	686,403	88,674
UBQLN2	0,001	0,543	0,779	0,363	0,384	0,08	605,226	225,525
CEP85	0,002	0,545	0,78	0,107	0,116	0,126	616,903	68,847
PTP4A2	0,002	0,544	0,78	0,067	0,072	0,098	1680,02	116,915
PSMB2	0,001	0,544	0,78	0,476	0,46	-0,052	1574,052	736,238
GTPBP2	0,003	0,546	0,78	0,079	0,088	0,155	538,147	44,729
TOMM5	0,002	0,546	0,78	4,371	5,075	0,215	21,021	99,366
UBAC1	0,003	0,545	0,78	0,141	0,155	0,142	376,775	55,63

PIK3C2A	0,002	0,544	0,78	0,103	0,111	0,106	944,676	100,921
PATL1	0,001	0,544	0,78	0,108	0,115	0,088	1335,553	149,391
KIAA1467	0,003	0,545	0,78	0,345	0,39	0,18	118,429	44,538
MFSD5	0,001	0,544	0,78	0,596	0,553	-0,108	228,508	131,618
UNG	0,001	0,545	0,78	0,316	0,295	-0,1	426,562	129,683
C14orf166	0,001	0,544	0,78	0,319	0,334	0,065	1110,102	362,831
NCOR1	0,001	0,545	0,78	0,07	0,067	-0,066	4457,141	305,06
MSI2	0,002	0,545	0,78	0,127	0,138	0,118	599,899	79,907
TRIM37	0,003	0,544	0,78	0,073	0,081	0,142	673,089	51,853
SEP9	0,001	0,545	0,78	0,128	0,124	-0,052	4320,797	544,307
C19orf25	0,003	0,545	0,78	0,238	0,215	-0,144	250,724	56,891
EHD2	0,001	0,544	0,78	0,161	0,152	-0,078	1336,954	209,103
NRD1	0,001	0,547	0,781	0,121	0,116	-0,06	2984,869	354,889
CHCHD1	0,001	0,547	0,781	1,012	1,08	0,094	208,143	217,657
MMACHC	0,002	0,549	0,783	0,202	0,218	0,111	452,774	95,102
DPY30	0,002	0,549	0,783	0,768	0,696	-0,143	104,445	76,038
COPZ1	0,001	0,548	0,783	0,257	0,245	-0,071	1088,673	272,962
SPATA5L1	0,003	0,549	0,783	0,285	0,319	0,166	145,953	43,912
SFT2D2	0,001	0,551	0,784	0,232	0,247	0,094	573,303	137,699
PIGC	0,003	0,551	0,784	0,264	0,292	0,146	203,561	56,309
RBPJ	0,002	0,55	0,784	0,086	0,079	-0,111	1037,872	85,304
UBE2B	0,002	0,551	0,784	0,583	0,539	-0,112	205,557	115,01
CAMLG	0,002	0,551	0,784	0,395	0,362	-0,126	216,149	81,785
BICD2	0,001	0,55	0,784	0,141	0,149	0,08	1312,89	190,325
IDH3A	0,001	0,55	0,784	0,581	0,619	0,091	313,64	188,588
ECI2	0,002	0,552	0,785	0,251	0,271	0,107	405,061	105,577
CNTROB	0,002	0,552	0,785	0,073	0,078	0,093	1702,28	128,677
CCDC94	0,002	0,552	0,785	0,316	0,289	-0,129	245,899	74,613
CDK5RAP1	0,002	0,551	0,785	0,183	0,196	0,099	622,442	118,082
RALGPS2	0,003	0,553	0,786	0,128	0,116	-0,152	371,041	45,305
DCAF6	0,003	0,554	0,787	0,111	0,123	0,144	425,06	49,91
RFC4	0,002	0,554	0,787	0,31	0,334	0,107	333,72	107,532

MB21D2	0,003	0,553	0,787	0,341	0,309	-0,14	188,489	60,995
DCP2	0,003	0,554	0,787	0,101	0,092	-0,134	587,135	56,789
DNAJC2	0,003	0,553	0,787	0,203	0,185	-0,138	303,321	58,562
RIMS2	0,003	0,555	0,787	0,117	0,106	-0,144	445,616	49,566
PCBD1	0,001	0,554	0,787	0,336	0,359	0,095	412,449	143,064
CUTC	0,002	0,555	0,787	0,509	0,461	-0,142	134,858	65,152
WSB2	0,002	0,554	0,787	0,036	0,033	-0,122	1934,764	67,71
GOLGA5	0,002	0,553	0,787	0,19	0,175	-0,117	441,654	80,254
MRGBP	0,003	0,555	0,787	0,108	0,098	-0,134	549,865	56,545
FXR1	0,002	0,556	0,788	0,111	0,118	0,091	1171,99	134,182
ZNF622	0,003	0,556	0,788	0,136	0,123	-0,145	382,532	49,533
ATP6V0E1	0,001	0,556	0,788	0,423	0,401	-0,076	571,355	234,801
ARPC1A	0,001	0,556	0,788	0,194	0,206	0,09	739,809	147,359
TRUB2	0,001	0,555	0,788	0,401	0,426	0,085	456,318	188,425
WDR20	0,002	0,556	0,788	0,178	0,195	0,133	331,662	61,902
GABARAP	0,002	0,556	0,788	7,744	6,51	-0,25	14,089	99,585
SUMO2	0,003	0,556	0,788	0,112	0,124	0,144	420,773	49,371
UQCRFS1	0,001	0,556	0,788	0,474	0,446	-0,087	402,837	185,114
KCNN4	0,002	0,556	0,788	0,301	0,325	0,112	306,388	96,284
CNNM3	0,002	0,558	0,789	0,247	0,226	-0,127	293,099	69,185
HEXB	0,001	0,558	0,789	0,56	0,584	0,059	884,185	505,089
ZNF592	0,003	0,558	0,789	0,073	0,066	-0,143	687,38	47,658
NR2C2AP	0,002	0,558	0,789	0,532	0,484	-0,135	145,2	73,148
MTR	0,002	0,559	0,79	0,093	0,086	-0,121	768,271	68,724
PNKD	0,001	0,559	0,79	0,498	0,466	-0,096	314,179	151,877
SFMBT1	0,003	0,56	0,79	0,144	0,13	-0,144	362,112	49,346
PHC3	0,002	0,558	0,79	0,187	0,203	0,113	439,547	85,967
SYNPO	0,003	0,56	0,79	0,096	0,106	0,144	495,563	49,637
CDK5RAP2	0,002	0,56	0,79	0,085	0,091	0,098	1244,828	109,203
PKN3	0,002	0,56	0,79	0,096	0,089	-0,121	760,864	70,781
C11orf30	0,002	0,559	0,79	0,092	0,1	0,123	695,82	66,667
DUSP14	0,001	0,56	0,79	0,472	0,444	-0,087	391,453	179,232

TK1	0,001	0,559	0,79	0,407	0,394	-0,045	2223,177	888,408
TPGS1	0,003	0,559	0,79	0,981	1,111	0,18	50,491	52,514
GLTSCR1	0,003	0,559	0,79	0,298	0,27	-0,142	205,1	58,459
DDX17	0,001	0,559	0,79	0,106	0,111	0,075	1811,225	196,289
MMADHC	0,001	0,562	0,791	0,637	0,611	-0,059	780,772	487,55
MTX2	0,001	0,561	0,791	0,496	0,464	-0,096	304,552	146,183
CYP20A1	0,002	0,561	0,791	0,393	0,365	-0,107	286,946	108,312
DNAJC13	0,002	0,561	0,791	0,086	0,08	-0,108	1033,237	85,827
DHX36	0,002	0,561	0,791	0,103	0,094	-0,128	618,291	60,644
TBL1XR1	0,001	0,561	0,791	0,159	0,169	0,088	848,911	139,72
POLR3H	0,002	0,562	0,791	0,131	0,122	-0,108	696,065	87,891
PTGER4	0,003	0,563	0,792	0,306	0,273	-0,165	145,717	42,444
OXR1	0,002	0,562	0,792	0,173	0,189	0,128	350,222	63,653
RPL12	0,001	0,563	0,792	0,12	0,126	0,074	1621,783	199,21
THOC6	0,002	0,563	0,792	0,302	0,327	0,113	299,301	94,902
HAT1	0,001	0,564	0,793	0,372	0,389	0,064	891,011	338,859
BIRC2	0,001	0,564	0,793	0,107	0,113	0,075	1759,974	193,952
MYL12A	0,001	0,564	0,793	0,534	0,512	-0,059	876,197	458,561
FBLN1	0,002	0,564	0,793	0,274	0,254	-0,108	365,907	96,583
MED8	0,001	0,566	0,794	0,355	0,336	-0,08	565,365	195,339
CNN3	0,001	0,565	0,794	0,41	0,422	0,041	3339,889	1389,341
UBE2T	0,001	0,567	0,794	0,968	0,925	-0,067	437,506	414,352
COPS7B	0,002	0,566	0,794	0,173	0,161	-0,102	604,213	100,693
MRFAP1L1	0,002	0,566	0,794	0,201	0,184	-0,13	321,498	62,074
WBSCR22	0,003	0,566	0,794	0,627	0,558	-0,168	78,84	46,852
IARS	0,001	0,565	0,794	0,19	0,196	0,046	4525,865	872,319
CLMP	0,003	0,567	0,794	0,16	0,177	0,15	310,545	51,325
C12orf29	0,002	0,566	0,794	0,274	0,297	0,12	272,047	77,84
RPS6KA3	0,002	0,565	0,794	0,161	0,148	-0,124	429,844	66,252
FAM127B	0,003	0,566	0,794	0,228	0,206	-0,15	217,593	47,281
HDLBP	0,001	0,568	0,795	0,077	0,08	0,049	8494,641	665,353
EML3	0,003	0,567	0,795	0,119	0,109	-0,133	492,116	56,33

USP28	0,002	0,568	0,795	0,146	0,159	0,121	472,129	72,962
CDCA3	0,003	0,567	0,795	0,355	0,32	-0,153	147,16	49,941
SPAG7	0,001	0,568	0,795	0,476	0,504	0,083	398,57	194,794
PTPN1	0,001	0,568	0,795	0,138	0,144	0,071	1572,598	221,505
PDZD11	0,001	0,568	0,795	0,328	0,309	-0,085	525,691	167,224
TXLNA	0,001	0,569	0,796	0,15	0,157	0,067	1659,03	254,065
EVI5	0,003	0,57	0,796	0,131	0,119	-0,13	461,045	57,693
RPL7L1	0,002	0,57	0,796	0,038	0,035	-0,12	1742,433	63,314
RNF216	0,002	0,569	0,796	0,104	0,096	-0,107	851,588	85,188
PAK1	0,002	0,569	0,796	0,142	0,132	-0,101	748,235	102,585
AARS	0,001	0,57	0,796	0,162	0,155	-0,064	1801,893	286,374
PITPNA	0,001	0,569	0,796	0,118	0,125	0,082	1344,881	163,109
ATPAF2	0,003	0,569	0,796	0,265	0,293	0,145	185,025	51,474
CDK5RAP3	0,002	0,57	0,796	0,131	0,143	0,126	448,293	61,241
PHIP	0,002	0,571	0,797	0,068	0,073	0,111	1055,473	74,908
DYNLT1	0,002	0,571	0,797	0,549	0,508	-0,112	197,682	104,983
CCNF	0,002	0,571	0,797	0,136	0,144	0,091	895,104	125,121
SRCAP	0,001	0,571	0,797	0,087	0,091	0,072	2491,012	221,804
PPIL2	0,003	0,571	0,797	0,099	0,109	0,135	503,131	52,06
PAPD7	0,003	0,572	0,798	0,083	0,076	-0,132	661,482	52,577
MOCS2	0,001	0,572	0,798	0,308	0,328	0,091	440,639	140,103
DCAF13	0,001	0,572	0,798	0,541	0,572	0,08	380,735	212,586
IFIT1	0,002	0,574	0,799	0,183	0,172	-0,092	719,731	126,598
C15orf57	0,003	0,573	0,799	0,526	0,467	-0,171	84,331	42,133
TMEM8A	0,003	0,573	0,799	0,077	0,071	-0,134	691,613	51,215
RFX1	0,002	0,574	0,799	0,42	0,389	-0,11	240,7	97,655
SPEN	0,003	0,575	0,8	0,029	0,027	-0,138	1622,175	45,14
ACTR1B	0,001	0,576	0,8	0,241	0,226	-0,088	624,488	146,53
BMPR2	0,002	0,575	0,8	0,108	0,101	-0,102	864,493	90,367
CAP2	0,002	0,576	0,8	0,21	0,197	-0,096	564,446	115,144
RXRA	0,003	0,575	0,8	0,076	0,083	0,136	620,782	49,158
PARVA	0,001	0,576	0,8	0,204	0,194	-0,077	912,64	181,578

DDB1	0,001	0,576	0,8	0,128	0,125	-0,044	5484,067	693,343
ANO6	0,002	0,575	0,8	0,101	0,108	0,102	869,362	91,284
LIG4	0,003	0,575	0,8	0,111	0,101	-0,131	502,926	53,111
ING1	0,003	0,575	0,8	0,221	0,243	0,134	244,228	56,64
GTF2A2	0,001	0,576	0,8	0,399	0,426	0,095	317,715	130,921
MAP2K1	0,002	0,576	0,8	0,248	0,233	-0,094	500,607	120,192
YTHDF2	0,001	0,578	0,801	0,253	0,238	-0,093	492,335	120,7
USP24	0,001	0,577	0,801	0,085	0,089	0,08	1771,605	154,274
ITGB5	0,003	0,577	0,801	0,188	0,17	-0,139	294,319	53,185
FAM49B	0,001	0,578	0,801	0,236	0,248	0,072	888,364	215,064
GOLM1	0,001	0,577	0,801	0,134	0,14	0,063	2097,59	286,827
TRAPPC2L	0,001	0,577	0,801	0,695	0,649	-0,097	217,076	146,37
AP2A1	0,001	0,577	0,801	0,227	0,218	-0,057	1839,53	410,343
MAN1B1	0,001	0,578	0,802	0,264	0,278	0,076	711,096	192,312
INTS7	0,001	0,58	0,803	0,251	0,238	-0,075	800,248	195,934
TMEM194B	0,002	0,579	0,803	0,222	0,203	-0,127	285,777	60,813
EXTL3	0,002	0,579	0,803	0,085	0,078	-0,122	731,769	59,567
PRKAG1	0,002	0,58	0,803	0,294	0,314	0,095	391,333	119,026
NGRN	0,002	0,58	0,803	0,351	0,378	0,108	261,052	95,032
FLCN	0,002	0,58	0,803	0,15	0,162	0,113	492,261	76,309
BRIP1	0,002	0,58	0,803	0,108	0,1	-0,116	644,469	66,755
CYP1B1	0,002	0,581	0,804	0,152	0,14	-0,121	441,171	63,845
SLC1A4	0,002	0,582	0,804	0,355	0,388	0,13	171,974	63,948
MPP6	0,003	0,582	0,804	0,17	0,155	-0,128	352,571	56,955
OBFC1	0,003	0,581	0,804	0,21	0,23	0,129	267,2	58,869
NUP98	0,001	0,581	0,804	0,115	0,119	0,054	3436,741	400,942
GLTP	0,002	0,581	0,804	0,175	0,163	-0,102	567,812	96,299
USP36	0,002	0,581	0,804	0,067	0,072	0,111	1055,93	73,267
GPR180	0,002	0,582	0,805	0,167	0,18	0,111	444,203	77,118
RABGGTB	0,001	0,586	0,806	0,327	0,34	0,054	1286,66	428,7
BCL10	0,001	0,583	0,806	0,427	0,402	-0,086	379,363	156,952
GALNT14	0,002	0,586	0,806	0,328	0,305	-0,105	297,598	93,812

BIRC6	0,001	0,585	0,806	0,174	0,183	0,07	1201,046	214,405
MRPL47	0,001	0,584	0,806	0,575	0,606	0,078	365,75	215,74
COPS4	0,001	0,586	0,806	0,446	0,473	0,084	363,511	167,553
TTC37	0,002	0,584	0,806	0,081	0,086	0,095	1223,534	101,996
KIAA0141	0,001	0,583	0,806	0,251	0,236	-0,092	499,707	121,871
VARS	0,002	0,585	0,806	0,241	0,256	0,09	523,904	129,572
REPS1	0,002	0,583	0,806	0,188	0,201	0,098	527,781	102,31
GNB2	0,001	0,586	0,806	0,474	0,491	0,049	1285,815	620,348
MARVELD1	0,003	0,585	0,806	0,106	0,096	-0,146	412,77	41,767
POLR2L	0,001	0,584	0,806	1,473	1,424	-0,049	709,941	1029,955
DNM1L	0,001	0,585	0,806	0,194	0,204	0,071	1023,625	203,797
SCAF11	0,002	0,586	0,806	0,076	0,081	0,096	1238,815	96,897
ANKRD13A	0,002	0,586	0,806	0,084	0,077	-0,125	772,39	62,879
GNPNAT1	0,002	0,586	0,806	0,166	0,178	0,097	580,364	100,272
CCNB2	0,001	0,585	0,806	0,429	0,447	0,06	814,251	356,992
BLVRB	0,001	0,584	0,806	1,232	1,168	-0,078	252,101	304,017
YWHAH	0,001	0,585	0,806	0,327	0,337	0,044	2265,694	752,955
ENSG00000210195.2	0,001	0,586	0,806	88,219	64,003	-0,463	3,044	223,462
UFC1	0,002	0,588	0,807	0,219	0,234	0,097	491,955	110,756
ZFAND5	0,001	0,587	0,807	0,135	0,142	0,081	1037,995	143,791
CKAP5	0,001	0,587	0,807	0,051	0,049	-0,064	4792,339	240,115
TMEM11	0,002	0,587	0,807	0,223	0,237	0,09	538,964	123,94
DPP9	0,001	0,587	0,807	0,121	0,116	-0,059	2473,983	294,237
ADAM19	0,002	0,588	0,808	0,079	0,085	0,105	941,448	77,661
CDC5L	0,002	0,589	0,808	0,068	0,063	-0,118	920,387	59,998
ACP2	0,002	0,588	0,808	0,306	0,33	0,109	277,752	87,962
LAMTOR1	0,001	0,59	0,808	0,199	0,189	-0,074	951,137	184,825
CLMN	0,003	0,59	0,808	0,139	0,152	0,126	383,421	55,558
RCOR1	0,001	0,589	0,808	0,114	0,107	-0,087	1056,196	115,862
C19orf43	0,001	0,589	0,808	0,24	0,25	0,057	1410,637	345,286
ATP13A1	0,001	0,589	0,808	0,18	0,188	0,066	1265,367	232,095
CAPZA1	0,001	0,591	0,809	0,255	0,263	0,048	2561,862	662,131

CERS6	0,001	0,591	0,809	0,295	0,278	-0,086	485,49	138,893
MTRR	0,002	0,59	0,809	0,136	0,145	0,093	736,48	103,694
YTHDF3	0,001	0,591	0,809	0,102	0,108	0,08	1338,17	140,586
ACLY	0,002	0,59	0,809	0,108	0,115	0,089	1047,917	116,361
MLX	0,001	0,591	0,809	0,278	0,266	-0,062	1083,639	294,499
SUGP2	0,002	0,591	0,809	0,082	0,076	-0,102	1050,222	83,263
EXOSC5	0,001	0,59	0,809	0,729	0,686	-0,088	238,259	168,7
RRBP1	0,002	0,591	0,809	0,031	0,032	0,086	3578,459	113,008
SMIM12	0,003	0,592	0,81	0,167	0,184	0,136	273,012	47,892
MKI67IP	0,002	0,592	0,81	0,313	0,335	0,096	344,323	111,52
EIF4E	0,002	0,592	0,81	0,468	0,431	-0,118	172,204	77,424
NDUFB6	0,001	0,593	0,81	2,546	2,702	0,086	132,464	347,424
STX3	0,003	0,592	0,81	0,102	0,093	-0,139	445,322	43,481
UROD	0,001	0,593	0,811	0,519	0,502	-0,048	1342,182	686,278
DDX41	0,001	0,593	0,811	0,157	0,148	-0,081	936,239	142,932
FANCE	0,002	0,593	0,811	0,289	0,269	-0,101	341,373	95,355
GCLC	0,002	0,596	0,811	0,179	0,194	0,112	383,064	71,34
STOM	0,002	0,596	0,811	0,323	0,298	-0,119	219,85	68,053
OPTN	0,003	0,595	0,811	0,188	0,204	0,123	301,749	59,231
PPA1	0,001	0,595	0,811	0,495	0,475	-0,061	681,415	330,553
DLG5	0,003	0,595	0,811	0,05	0,055	0,119	1121,59	58,437
C11orf83	0,003	0,594	0,811	0,654	0,585	-0,162	70,927	44,013
WIPF2	0,003	0,595	0,811	0,088	0,096	0,124	583,097	53,623
HDGFRP2	0,002	0,595	0,811	0,071	0,076	0,111	901,986	66,245
ETHE1	0,002	0,594	0,811	0,538	0,493	-0,126	135,86	70,079
TMEM160	0,003	0,595	0,811	1,036	0,925	-0,163	52,325	51,022
MAVS	0,001	0,595	0,811	0,144	0,137	-0,072	1263,231	176,565
TRMT6	0,002	0,595	0,811	0,191	0,203	0,087	624,278	123,456
HNRNPLL	0,002	0,596	0,812	0,287	0,268	-0,102	335,519	92,573
HJURP	0,001	0,597	0,812	0,146	0,152	0,055	2446,431	364,121
UBE2D2	0,001	0,596	0,812	0,33	0,313	-0,076	575,672	185,689
LIPA	0,001	0,597	0,812	0,445	0,464	0,062	691,599	314,967

SEPHS2	0,002	0,597	0,812	0,213	0,197	-0,112	353,956	71,997
UBE2S	0,001	0,597	0,812	0,318	0,333	0,066	766,292	248,893
ALS2	0,002	0,598	0,813	0,146	0,157	0,104	533,151	80,635
ZDHHC3	0,002	0,598	0,813	0,076	0,082	0,101	1021,881	80,693
RPS14	0,001	0,598	0,813	0,646	0,663	0,037	2815,373	1842,661
TFAM	0,001	0,598	0,813	0,306	0,295	-0,051	1558,676	467,474
FTH1	0,002	0,598	0,813	0,601	0,655	0,125	123,149	76,559
TBC1D4	0,001	0,598	0,813	0,157	0,166	0,078	965,502	156,606
ENSG00000235847.2	0,003	0,6	0,814	0,113	0,104	-0,122	513,47	56,053
PDE6D	0,003	0,599	0,814	0,327	0,3	-0,126	188,28	58,814
FADS3	0,001	0,599	0,814	0,305	0,318	0,064	883,431	274,027
UTP18	0,002	0,6	0,814	0,153	0,165	0,102	518,393	82,757
TRAPPC8	0,002	0,6	0,814	0,152	0,162	0,087	747,88	117,717
POC1A	0,001	0,602	0,815	0,309	0,327	0,083	439,394	139,91
SEP11	0,001	0,601	0,815	0,11	0,105	-0,059	2529,484	271,923
LYRM4	0,002	0,601	0,815	0,362	0,394	0,123	169,761	64,258
CBLL1	0,002	0,601	0,815	0,112	0,104	-0,11	629,465	68,175
COPS5	0,001	0,602	0,815	0,447	0,426	-0,07	514,554	224,023
ENSG00000201302.1	0,002	0,602	0,815	24,814	34,462	0,474	2,715	77,646
ASCC1	0,003	0,601	0,815	0,239	0,219	-0,124	248,941	56,847
CCS	0,003	0,601	0,815	0,293	0,323	0,141	152,718	46,767
XPO4	0,001	0,602	0,815	0,117	0,123	0,073	1419,176	170,287
DDX19A	0,003	0,601	0,815	0,16	0,173	0,12	351,529	58,761
VPS53	0,001	0,601	0,815	0,119	0,125	0,069	1558,864	189,985
ADNP	0,002	0,602	0,815	0,09	0,095	0,088	1176,543	108,905
RRP1	0,002	0,603	0,815	0,176	0,189	0,107	406,758	74,236
MID1	0,002	0,601	0,815	0,105	0,112	0,096	840,923	91,071
WDR35	0,002	0,603	0,816	0,115	0,124	0,108	583,644	69,932
ERCC3	0,001	0,604	0,816	0,192	0,181	-0,085	646,859	120,581
DARS2	0,002	0,605	0,817	0,167	0,157	-0,089	656,218	106,143
RCOR3	0,003	0,605	0,817	0,206	0,189	-0,127	266,163	52,596
GSTK1	0,002	0,605	0,817	0,287	0,307	0,098	337,568	99,832

MRPL21	0,001	0,605	0,817	0,84	0,885	0,075	269,708	232,678
RMDN3	0,002	0,605	0,817	0,214	0,198	-0,112	357,728	74,289
ANKRD27	0,002	0,605	0,817	0,074	0,068	-0,105	974,221	69,196
APOBEC3B	0,003	0,605	0,817	0,364	0,332	-0,13	163,399	56,179
MERTK	0,004	0,607	0,818	0,327	0,364	0,153	116,486	39,932
PGM2	0,001	0,607	0,818	0,265	0,254	-0,064	908,064	235,241
DTNBP1	0,003	0,607	0,818	0,435	0,397	-0,133	130,846	54,118
CENPW	0,001	0,607	0,818	1,145	1,228	0,101	118,023	140,216
DDX54	0,002	0,607	0,818	0,065	0,07	0,091	1411,539	95,251
SEC23A	0,001	0,606	0,818	0,234	0,224	-0,065	1006,542	229,99
MKNK2	0,002	0,607	0,818	0,124	0,134	0,105	576,505	73,968
NR1H2	0,002	0,606	0,818	0,231	0,216	-0,101	386,448	86,68
ENSG00000232956.4	0,002	0,608	0,819	0,336	0,362	0,107	236,245	82,105
FAM216A	0,002	0,608	0,819	0,43	0,463	0,105	203,15	90,448
PSMD11	0,001	0,608	0,819	0,249	0,258	0,051	1704,764	432,065
LMO7	0,003	0,609	0,82	0,041	0,045	0,129	1068,529	45,421
DAP3	0,001	0,61	0,821	0,239	0,248	0,057	1301,169	316,724
POLR3K	0,002	0,61	0,821	0,332	0,36	0,118	190,771	66,55
PPIH	0,001	0,611	0,822	0,642	0,672	0,064	440,52	289,642
GPBP1L1	0,002	0,611	0,822	0,085	0,09	0,081	1390,233	121,998
TNFRSF10B	0,001	0,611	0,822	0,185	0,193	0,06	1354,052	256,405
BMS1	0,002	0,611	0,822	0,047	0,05	0,104	1415,993	68,851
EIF1AD	0,002	0,611	0,822	0,16	0,171	0,094	563,333	93,323
ELP5	0,001	0,612	0,822	0,282	0,296	0,072	638,946	184,899
ARFIP1	0,002	0,613	0,823	0,349	0,371	0,089	323,237	116,534
ISOC1	0,002	0,612	0,823	0,353	0,33	-0,095	305,366	104,578
RTF1	0,002	0,613	0,823	0,087	0,082	-0,101	875,994	74,169
ENSG00000210184.1	0,001	0,612	0,823	59,174	46,417	-0,35	4,543	234,97
SLC30A1	0,001	0,615	0,824	0,262	0,276	0,077	548,528	147,902
PDLIM5	0,001	0,614	0,824	0,174	0,166	-0,068	1167,538	198,893
HSP90AB1	0,001	0,615	0,824	0,203	0,197	-0,039	4118,409	823,377
GLE1	0,001	0,614	0,824	0,196	0,187	-0,064	1163,827	223,052

GOT1	0,001	0,615	0,824	0,296	0,307	0,055	1117,02	336,874
NEDD1	0,002	0,615	0,824	0,174	0,187	0,099	456,218	82,252
CAMTA2	0,002	0,614	0,824	0,061	0,056	-0,116	948,535	55,716
RABEP1	0,001	0,614	0,824	0,074	0,071	-0,072	2094,959	151,15
KIF4A	0,001	0,615	0,824	0,092	0,087	-0,08	1315,605	116,974
FAM105B	0,003	0,615	0,825	0,118	0,128	0,121	416,555	51,382
EPDR1	0,001	0,616	0,825	0,269	0,283	0,073	614,318	169,49
SLC25A12	0,003	0,617	0,826	0,319	0,291	-0,134	154,11	46,899
TOMM70A	0,001	0,617	0,826	0,226	0,217	-0,059	1173,981	259,308
EPS8L2	0,001	0,617	0,826	0,12	0,115	-0,061	1908,777	223,53
GPN2	0,002	0,619	0,827	0,316	0,338	0,097	280,493	92,021
CTPS1	0,001	0,619	0,827	0,129	0,124	-0,059	1859,766	235,719
PPP1CB	0,001	0,619	0,827	0,162	0,154	-0,072	1016,408	159,612
MLF1	0,003	0,619	0,827	0,46	0,423	-0,121	141,885	62,451
HBS1L	0,002	0,619	0,827	0,195	0,184	-0,084	598,696	113,455
TNRC18	0,002	0,618	0,827	0,093	0,088	-0,083	1249,674	113,511
GTPBP10	0,002	0,619	0,827	0,175	0,162	-0,109	383,945	64,309
HSD17B12	0,001	0,619	0,827	0,326	0,338	0,052	1184,898	393,468
CAPNS1	0,001	0,618	0,827	0,502	0,514	0,036	2800,324	1420,611
AXL	0,001	0,618	0,827	0,181	0,185	0,031	14752,144	2694,317
ASCC2	0,002	0,619	0,827	0,186	0,176	-0,082	664,798	120,372
CEP97	0,002	0,62	0,828	0,279	0,263	-0,086	424,226	114,849
CD47	0,001	0,621	0,828	0,345	0,326	-0,08	416,359	139,798
TIMMDC1	0,002	0,62	0,828	0,376	0,354	-0,088	311,623	113,554
MYC	0,001	0,62	0,828	0,214	0,222	0,055	1454,075	316,663
HSP90AA1	0,001	0,621	0,828	0,171	0,168	-0,03	19068,077	3230,068
THOC5	0,001	0,622	0,829	0,236	0,224	-0,076	641,355	147,359
MRPL16	0,001	0,623	0,83	0,596	0,621	0,06	500,087	304,327
KCTD5	0,001	0,622	0,83	0,16	0,152	-0,079	802,34	125,365
AKAP10	0,002	0,623	0,83	0,114	0,121	0,091	755,701	89,115
PPM1B	0,002	0,624	0,832	0,2	0,214	0,103	352,836	73,257
DHCR24	0,001	0,628	0,833	0,163	0,167	0,041	4620,492	760,113

RTCA	0,001	0,628	0,833	0,328	0,313	-0,07	571,033	183,151
STRIP1	0,002	0,628	0,833	0,196	0,183	-0,095	441,743	83,581
ASB1	0,002	0,628	0,833	0,097	0,091	-0,094	850,315	80,389
IQSEC1	0,002	0,627	0,833	0,081	0,075	-0,106	779,316	60,569
RBM6	0,003	0,627	0,833	0,06	0,065	0,11	886,948	55,559
PDIA5	0,003	0,625	0,833	0,412	0,376	-0,135	119,94	47,368
STARD4	0,003	0,628	0,833	0,18	0,165	-0,127	274,122	47,63
STK10	0,002	0,627	0,833	0,081	0,077	-0,079	1456,144	114,941
GGH	0,001	0,628	0,833	0,741	0,773	0,06	425,094	322,158
KLF10	0,002	0,628	0,833	0,247	0,261	0,081	488,725	123,981
EFR3A	0,002	0,626	0,833	0,122	0,129	0,08	909,936	114,802
GAPVD1	0,001	0,627	0,833	0,15	0,157	0,066	1153,071	176,965
PAPSS2	0,001	0,628	0,833	0,123	0,118	-0,063	1647,761	198,793
EXOSC1	0,001	0,625	0,833	0,657	0,689	0,069	345,654	232,235
NECAP1	0,002	0,626	0,833	0,25	0,266	0,09	390,265	100,52
MAGOHB	0,001	0,625	0,833	1,188	1,266	0,091	131,225	161,279
NUPL1	0,001	0,628	0,833	0,213	0,219	0,043	2396,184	517,853
SPRYD7	0,002	0,628	0,833	0,402	0,378	-0,088	290,693	113,629
DLEU1	0,003	0,629	0,833	0,367	0,398	0,116	160,929	61,62
TMEM87A	0,001	0,628	0,833	0,464	0,491	0,083	279,355	133,878
GINS2	0,002	0,625	0,833	0,285	0,305	0,099	283,214	83,912
FZD2	0,001	0,626	0,833	0,579	0,597	0,042	1279,138	752,318
VMP1	0,001	0,627	0,833	0,358	0,346	-0,052	1009,06	354,798
KRI1	0,002	0,627	0,833	0,066	0,07	0,088	1327,628	90,169
AP5S1	0,004	0,626	0,833	0,355	0,321	-0,143	118,728	40,22
RAB10	0,001	0,632	0,834	0,15	0,143	-0,063	1360,358	198,989
EPT1	0,001	0,632	0,834	0,264	0,275	0,058	963,902	260,295
HK2	0,001	0,633	0,834	0,067	0,063	-0,072	2077,858	135,18
EAF1	0,002	0,633	0,834	0,137	0,128	-0,101	507,581	66,971
ATP6V1A	0,001	0,632	0,834	0,301	0,286	-0,075	496,059	145,264
GUF1	0,002	0,63	0,834	0,127	0,119	-0,09	714,202	87,58
FRYL	0,002	0,631	0,834	0,058	0,062	0,099	1095,818	66,272

BAG4	0,003	0,633	0,834	0,247	0,228	-0,114	241,858	57,545
SMARCA2	0,002	0,632	0,834	0,159	0,17	0,096	472,578	77,531
GOLGA2	0,003	0,63	0,834	0,048	0,044	-0,118	1016,139	46,858
ZDHHC12	0,003	0,632	0,834	0,23	0,211	-0,129	209,905	46,612
TOR1A	0,002	0,632	0,834	0,277	0,261	-0,084	410,634	110,382
ABI1	0,002	0,631	0,834	0,222	0,209	-0,088	453,985	97,677
CD82	0,001	0,631	0,834	0,535	0,514	-0,058	591,556	308,973
CDC42EP2	0,002	0,631	0,834	0,214	0,23	0,098	358,132	79,202
MRPL48	0,002	0,633	0,834	0,395	0,424	0,102	196,761	80,909
LARP4	0,002	0,633	0,834	0,086	0,091	0,076	1404,472	124,234
ATP6V0A2	0,002	0,633	0,834	0,227	0,243	0,097	338,942	80,055
TTC8	0,002	0,629	0,834	0,274	0,295	0,106	248,767	70,38
ZNF768	0,003	0,632	0,834	0,143	0,132	-0,114	389,416	53,529
TMEM107	0,003	0,633	0,834	0,436	0,395	-0,142	100,542	41,981
CC2D1A	0,002	0,632	0,834	0,107	0,115	0,104	569,588	63,179
LAMA5	0,001	0,631	0,834	0,112	0,117	0,052	2730,65	312,699
STMN3	0,002	0,633	0,834	0,305	0,325	0,09	320,079	100,52
L3MBTL2	0,003	0,63	0,834	0,132	0,121	-0,12	376,355	47,52
ANKRD28	0,001	0,634	0,835	0,08	0,077	-0,066	2027,759	158,635
RASSF7	0,002	0,634	0,835	0,308	0,287	-0,103	245,048	72,996
FBXL3	0,001	0,634	0,835	0,204	0,194	-0,075	681,69	135,667
PARP2	0,002	0,634	0,835	0,171	0,16	-0,096	462,129	76,228
UNC13D	0,001	0,634	0,835	0,12	0,116	-0,045	3440,823	407,464
C17orf53	0,003	0,635	0,836	0,235	0,256	0,12	208,166	51,032
TREX1	0,002	0,636	0,838	0,518	0,562	0,117	117,405	63,503
ZCCHC9	0,003	0,637	0,838	0,159	0,147	-0,116	326,677	50,051
SPPL3	0,002	0,637	0,838	0,263	0,247	-0,094	332,735	84,739
RNF220	0,002	0,639	0,839	0,104	0,111	0,091	779,222	83,583
FMNL2	0,003	0,638	0,839	0,067	0,072	0,107	801,166	55,565
CTNNB1	0,001	0,638	0,839	0,343	0,335	-0,033	3646,783	1236,479
BBX	0,002	0,639	0,839	0,049	0,052	0,1	1222,648	61,765
SEMA4B	0,002	0,638	0,839	0,242	0,258	0,097	315,417	78,595

KXD1	0,001	0,639	0,839	0,17	0,179	0,075	728,1	126,522
DESI2	0,003	0,64	0,84	0,08	0,074	-0,111	663,084	51,264
GPAT2	0,003	0,641	0,841	0,319	0,293	-0,124	156,334	47,659
LRRFIP1	0,001	0,641	0,841	0,115	0,12	0,063	1505,245	176,804
IMPAD1	0,001	0,642	0,842	0,116	0,12	0,055	2050,108	241,96
MIS18BP1	0,003	0,642	0,842	0,048	0,052	0,108	1044,467	52,101
ATG2B	0,003	0,642	0,842	0,1	0,109	0,113	484,33	51,129
TRIM21	0,003	0,643	0,843	0,193	0,177	-0,124	240,909	44,768
CNDP2	0,001	0,643	0,843	0,179	0,187	0,065	940,443	171,675
AKAP8L	0,003	0,642	0,843	0,104	0,097	-0,107	562,167	56,454
RAPGEF1	0,003	0,644	0,844	0,056	0,052	-0,109	928,121	50,367
FAM104A	0,002	0,644	0,844	0,353	0,333	-0,085	308,841	105,95
NEK2	0,002	0,646	0,845	0,206	0,218	0,081	502,234	106,522
AFTPH	0,001	0,646	0,845	0,184	0,192	0,065	881,335	165,805
STEAP3	0,002	0,646	0,845	0,137	0,145	0,088	600,365	84,805
PEG10	0,002	0,644	0,845	0,084	0,089	0,082	1102,61	95,442
HPS5	0,003	0,646	0,845	0,153	0,142	-0,107	376,045	55,582
GAS6	0,002	0,646	0,845	0,152	0,143	-0,086	625,481	92,726
ACD	0,001	0,645	0,845	0,43	0,45	0,066	432,32	190,155
SNAPC2	0,002	0,646	0,845	0,261	0,278	0,089	342,123	91,869
RGS19	0,004	0,646	0,845	0,33	0,3	-0,138	122,775	38,93
RANGAP1	0,001	0,646	0,845	0,233	0,239	0,035	3801,995	897,204
GCLM	0,001	0,647	0,846	0,295	0,286	-0,045	1496,719	433,779
FOSL2	0,001	0,647	0,846	0,069	0,066	-0,067	2073,106	140,766
XRCC5	0,001	0,647	0,846	0,134	0,138	0,034	6305,108	857,965
SCARB2	0,001	0,647	0,846	0,255	0,244	-0,06	840,887	209,449
PIGV	0,003	0,649	0,847	0,379	0,411	0,115	142,146	55,933
H1FX	0,001	0,649	0,847	0,605	0,636	0,071	290,115	180,01
HDAC2	0,001	0,649	0,847	0,328	0,318	-0,043	1486,349	480,044
POP7	0,001	0,648	0,847	0,594	0,565	-0,073	286,628	166,226
NBR1	0,001	0,648	0,847	0,148	0,153	0,051	1886,217	283,753
PXK	0,003	0,65	0,848	0,141	0,131	-0,102	436,791	59,104

TMEM184C	0,002	0,65	0,848	0,174	0,186	0,093	413,714	74,632
GCNT2	0,002	0,65	0,848	0,125	0,132	0,086	723,664	94,019
SHCBP1	0,001	0,65	0,848	0,284	0,274	-0,055	907,914	252,922
TOR1AIP2	0,001	0,651	0,849	0,219	0,228	0,057	1016,887	227,092
UCHL5	0,001	0,651	0,849	0,26	0,272	0,065	637,309	169,743
GGA3	0,003	0,651	0,849	0,079	0,073	-0,106	697,784	53,43
CCRN4L	0,001	0,653	0,85	0,572	0,602	0,074	268,942	157,769
KRT18	0,001	0,653	0,85	0,308	0,321	0,059	703,147	220,757
CDK8	0,002	0,653	0,85	0,165	0,154	-0,101	387,545	61,789
TERF2IP	0,002	0,653	0,85	0,248	0,262	0,079	441,454	112,177
UBA52	0,001	0,653	0,85	0,922	0,942	0,03	2460,895	2292,198
GTPBP1	0,002	0,653	0,85	0,205	0,218	0,091	385,888	81,111
POLA1	0,003	0,653	0,85	0,084	0,078	-0,107	628,559	50,555
CAP1	0,001	0,66	0,851	0,22	0,224	0,032	5104,421	1132,508
TADA1	0,003	0,658	0,851	0,334	0,364	0,122	131,961	45,818
STX6	0,003	0,654	0,851	0,167	0,181	0,116	266,072	46,368
ARL8A	0,005	0,658	0,851	0,233	0,259	0,157	139,439	33,49
BPNT1	0,001	0,655	0,851	0,376	0,395	0,074	354,955	136,645
ASAP2	0,002	0,658	0,851	0,161	0,17	0,082	576,699	95,026
NBAS	0,003	0,658	0,851	0,113	0,104	-0,115	406,212	44,079
CDC42EP3	0,001	0,655	0,851	0,121	0,125	0,053	1954,624	241,447
B3GNT2	0,002	0,658	0,851	0,321	0,304	-0,077	372,933	116,291
DOK1	0,003	0,659	0,851	0,128	0,119	-0,112	378,864	47,023
DARS	0,001	0,656	0,851	0,288	0,278	-0,054	897,07	253,939
SUMO1	0,002	0,659	0,851	0,333	0,356	0,098	208,431	71,594
ARL4C	0,003	0,66	0,851	0,098	0,106	0,103	530,875	54,449
MRPS25	0,002	0,658	0,851	0,221	0,21	-0,075	542,582	116,714
MYD88	0,002	0,655	0,851	0,178	0,167	-0,092	426,87	73,476
ARF4	0,001	0,66	0,851	0,332	0,34	0,037	2010,791	676,056
BDH1	0,002	0,659	0,851	0,207	0,193	-0,101	301,838	60,312
NMU	0,003	0,657	0,851	1,219	1,357	0,155	37,675	47,848
KIAA1430	0,002	0,658	0,851	0,159	0,15	-0,087	526,919	81,229

MIER3	0,003	0,656	0,851	0,142	0,152	0,101	406,568	59,891
FAM53C	0,003	0,658	0,851	0,065	0,06	-0,113	697,431	43,697
SMIM13	0,003	0,658	0,851	0,133	0,122	-0,12	321,065	41,085
FOXK1	0,001	0,66	0,851	0,112	0,107	-0,071	1095,167	120,282
FGFR1	0,001	0,657	0,851	0,12	0,125	0,06	1474,243	180,88
B4GALT1	0,001	0,658	0,851	0,166	0,174	0,07	764,431	130,269
NUP188	0,001	0,658	0,851	0,139	0,135	-0,038	4208,925	577,696
RABL6	0,003	0,656	0,851	0,056	0,06	0,105	956,869	55,882
NPDC1	0,005	0,654	0,851	0,255	0,231	-0,146	145,507	35,709
C10orf12	0,002	0,654	0,851	0,198	0,211	0,094	353,083	72,43
HRAS	0,002	0,656	0,851	0,203	0,193	-0,077	555,478	110,178
CEP164	0,003	0,658	0,851	0,056	0,052	-0,112	810,88	43,977
MRPS35	0,001	0,656	0,851	0,38	0,363	-0,067	446,002	165,884
SLC25A30	0,003	0,656	0,851	0,167	0,179	0,103	340,717	58,697
ATP11A	0,002	0,657	0,851	0,071	0,076	0,099	791,171	58,342
IREB2	0,002	0,658	0,851	0,165	0,156	-0,077	665,995	106,519
WDR73	0,002	0,66	0,851	0,306	0,286	-0,1	223,382	66,024
JMJD8	0,003	0,655	0,851	0,223	0,207	-0,11	244,526	52,675
DHODH	0,003	0,657	0,851	0,308	0,283	-0,123	148,116	43,687
TIMM44	0,001	0,658	0,851	0,18	0,172	-0,062	939,227	165,291
COLGALT1	0,001	0,657	0,851	0,215	0,222	0,041	2536,352	555,148
NFKBIB	0,002	0,659	0,851	0,235	0,221	-0,093	325,342	74,247
DGCR14	0,003	0,658	0,851	0,236	0,254	0,11	214,875	52,625
RPS19BP1	0,002	0,659	0,851	0,274	0,29	0,08	372,176	105,027
CLCN3	0,002	0,662	0,852	0,155	0,147	-0,081	626,132	94,113
MAP1B	0,001	0,662	0,852	0,152	0,156	0,036	4842,891	742,341
DNM2	0,001	0,661	0,852	0,168	0,163	-0,041	2784,262	461,368
H1F0	0,001	0,662	0,852	0,152	0,149	-0,03	8295,45	1251,399
CENPM	0,001	0,662	0,852	0,438	0,418	-0,066	410,015	175,206
ENSG00000237973.1	0,001	0,662	0,853	0,203	0,209	0,037	2914,36	599,933
CSF1	0,002	0,662	0,853	0,26	0,242	-0,099	257,509	64,3
MON1A	0,003	0,663	0,853	0,174	0,187	0,105	298,346	53,76

TRIP13	0,001	0,663	0,853	0,207	0,216	0,065	714,727	151,031
MARCH6	0,001	0,663	0,853	0,322	0,313	-0,042	1489,429	473,08
EZR	0,001	0,662	0,853	0,078	0,08	0,047	3719,978	293,894
C8orf59	0,003	0,664	0,853	1,852	2,043	0,142	33,397	65,25
DCUN1D5	0,001	0,663	0,853	0,436	0,418	-0,062	458,782	195,697
ESAM	0,001	0,664	0,853	0,296	0,308	0,053	908,665	273,042
MYO9B	0,001	0,663	0,853	0,107	0,111	0,06	1539,364	168,15
FAM83D	0,001	0,663	0,853	0,301	0,309	0,042	1538,681	468,673
TMEM109	0,001	0,665	0,854	0,389	0,38	-0,035	2310,519	889,94
CDIP1	0,003	0,665	0,854	0,223	0,241	0,113	206,169	47,763
DOPEY2	0,003	0,665	0,854	0,167	0,155	-0,105	325,007	52,088
PSMB4	0,001	0,666	0,855	0,936	0,916	-0,032	1806,277	1672,313
PIGG	0,002	0,667	0,855	0,303	0,322	0,088	267,489	83,368
ZNF706	0,002	0,666	0,855	0,429	0,403	-0,09	205,348	85,016
RGS10	0,001	0,666	0,855	0,558	0,543	-0,039	1131,463	622,816
FAR1	0,001	0,666	0,855	0,182	0,175	-0,055	1191,235	212,375
BRMS1	0,001	0,666	0,855	0,233	0,222	-0,067	622,893	141,806
SAMD1	0,002	0,665	0,855	0,166	0,157	-0,081	553,453	89,33
UQCC1	0,001	0,667	0,855	0,209	0,202	-0,05	1349,372	277,935
SNRPE	0,002	0,669	0,856	0,759	0,811	0,096	115,304	90,251
FAM98A	0,002	0,668	0,856	0,123	0,129	0,07	921,39	116,314
HDAC4	0,002	0,668	0,856	0,136	0,127	-0,094	487,36	64,09
REST	0,002	0,668	0,856	0,137	0,128	-0,095	458,045	60,437
ALKBH1	0,003	0,667	0,856	0,287	0,265	-0,111	180,252	49,636
HERC2	0,002	0,669	0,856	0,075	0,08	0,078	1180,236	91,512
CDC27	0,001	0,668	0,856	0,069	0,066	-0,067	1854,788	125,434
GPR108	0,001	0,668	0,856	0,315	0,302	-0,062	576,931	178,164
PWP2	0,001	0,668	0,856	0,245	0,235	-0,063	672,524	161,163
COG2	0,002	0,67	0,857	0,257	0,242	-0,087	322,85	80,08
EIF2B4	0,002	0,67	0,857	0,312	0,293	-0,09	256,789	77,688
IMPDH2	0,001	0,67	0,857	0,354	0,362	0,03	4370,968	1566,205
MCM4	0,001	0,671	0,857	0,138	0,135	-0,036	4476,339	609,172

MCM10	0,002	0,671	0,857	0,077	0,082	0,085	922,314	73,886
NCOA4	0,003	0,67	0,857	0,322	0,348	0,11	158,353	52,82
CMTR2	0,003	0,671	0,857	0,111	0,119	0,101	454,52	52,577
SAMD4B	0,002	0,67	0,857	0,105	0,1	-0,072	1046,942	107,899
RALB	0,002	0,672	0,858	0,168	0,158	-0,087	449,986	73,048
STAT1	0,001	0,672	0,858	0,136	0,142	0,059	1188,39	165,123
SP100	0,001	0,672	0,858	0,305	0,316	0,05	859,804	267,077
USP13	0,003	0,672	0,858	0,112	0,105	-0,096	529,982	57,283
TMEM161B	0,002	0,672	0,858	0,372	0,348	-0,093	201,036	72,103
CDK13	0,003	0,673	0,858	0,057	0,053	-0,104	844,152	46,596
PHTF2	0,002	0,673	0,858	0,116	0,11	-0,084	680,203	76,783
SURF4	0,001	0,672	0,858	0,703	0,667	-0,075	204,579	140,19
CDK2	0,001	0,674	0,858	0,247	0,238	-0,054	913,922	221,456
BRAP	0,002	0,673	0,858	0,174	0,185	0,095	339,799	61,172
PKMYT1	0,001	0,672	0,858	0,206	0,215	0,059	820,293	172,969
GTF3C1	0,002	0,674	0,858	0,053	0,051	-0,07	2026,598	105,484
USP32	0,002	0,673	0,858	0,086	0,091	0,081	892,281	79,398
TIMM13	0,001	0,673	0,858	0,569	0,591	0,054	468,091	271,899
APOL2	0,002	0,673	0,858	0,275	0,29	0,075	383,774	108,355
PTPLB	0,001	0,675	0,859	0,209	0,218	0,064	667,225	142,566
ALG8	0,001	0,675	0,859	0,887	0,912	0,041	697,678	627,415
ANKRD52	0,001	0,675	0,859	0,092	0,095	0,053	2672,362	248,599
GAS2L3	0,002	0,675	0,859	0,125	0,132	0,081	658,622	84,277
ZWILCH	0,002	0,676	0,859	0,237	0,223	-0,091	303,687	69,433
MAGED2	0,001	0,675	0,859	0,304	0,314	0,047	1007,383	312
DHX30	0,001	0,677	0,86	0,062	0,065	0,06	2375,668	150,058
TBC1D23	0,002	0,676	0,86	0,22	0,231	0,072	486,284	109,886
APIP	0,003	0,676	0,86	0,29	0,313	0,11	160,104	48,249
ALDH6A1	0,002	0,677	0,86	0,307	0,287	-0,097	213,708	63,043
NIP7	0,001	0,677	0,86	0,359	0,346	-0,053	667,445	235,017
VAPB	0,002	0,676	0,86	0,164	0,156	-0,068	750,491	119,85
RMND5A	0,002	0,679	0,861	0,095	0,1	0,078	896,011	87,986

PLS1	0,002	0,678	0,861	0,169	0,159	-0,087	428,792	70,247
SLC39A8	0,002	0,677	0,861	0,264	0,25	-0,076	401,578	103,313
HCFC1R1	0,002	0,679	0,861	0,322	0,344	0,095	200,028	66,666
KIF22	0,001	0,678	0,861	0,181	0,188	0,051	1222,131	225,313
EMC6	0,003	0,679	0,861	7,301	8,605	0,237	7,47	59,044
LMAN1	0,001	0,678	0,861	0,234	0,242	0,045	1341,104	319,707
GYG1	0,002	0,679	0,862	0,373	0,396	0,087	211,988	81,648
ATP13A3	0,001	0,68	0,862	0,112	0,117	0,06	1340,471	154,814
RALA	0,002	0,679	0,862	0,24	0,252	0,069	496,339	122,511
LAYN	0,001	0,681	0,862	0,248	0,259	0,063	611,967	154,33
GTF3A	0,002	0,68	0,862	0,061	0,065	0,086	1035,308	65,179
PLEKHJ1	0,003	0,679	0,862	0,176	0,189	0,105	266,232	48,557
PGRMC2	0,001	0,682	0,863	0,318	0,303	-0,066	436,14	135,297
SRPK1	0,001	0,682	0,863	0,12	0,115	-0,053	1653,88	193,885
PHF20L1	0,002	0,681	0,863	0,059	0,055	-0,087	1112,383	63,168
UROS	0,003	0,681	0,863	0,125	0,117	-0,096	456,239	55,156
TEX30	0,001	0,682	0,863	0,546	0,52	-0,07	263,252	139,963
ITFG1	0,002	0,682	0,863	0,307	0,323	0,073	353,2	111,481
HSBP1	0,001	0,681	0,863	0,351	0,338	-0,052	701,549	241,91
MRPL11	0,001	0,683	0,864	0,481	0,499	0,053	498,777	244,547
AIP	0,001	0,683	0,864	0,447	0,465	0,057	469,83	213,687
MAD2L2	0,002	0,684	0,865	0,293	0,278	-0,073	377,531	107,467
FASTKD3	0,003	0,684	0,865	0,26	0,278	0,097	214,883	58,029
CNPY3	0,002	0,684	0,865	0,241	0,23	-0,067	532,565	125,446
MCU	0,003	0,685	0,865	0,128	0,137	0,094	418,122	55,591
CHTF18	0,002	0,685	0,865	0,108	0,101	-0,088	597,517	62,395
SNX7	0,002	0,686	0,866	0,188	0,2	0,089	340,39	66,062
RQCD1	0,001	0,685	0,866	0,194	0,201	0,048	1291,064	254,626
WDFY1	0,002	0,686	0,866	0,152	0,146	-0,065	835,478	124,596
CPEB4	0,003	0,687	0,866	0,158	0,169	0,096	335,846	54,802
KCTD20	0,002	0,687	0,866	0,069	0,066	-0,067	1609,693	108,687
KLC2	0,001	0,687	0,866	0,18	0,173	-0,056	1007,212	177,708

PRPSAP1	0,002	0,685	0,866	0,278	0,264	-0,076	355,341	96,429
AP2M1	0,001	0,688	0,867	0,213	0,218	0,034	2826,958	610,016
LRCH3	0,002	0,688	0,867	0,157	0,148	-0,085	443,406	67,619
CCDC127	0,003	0,687	0,867	0,162	0,173	0,099	307,389	51,331
CMBL	0,002	0,689	0,867	0,396	0,376	-0,075	270,684	104,435
NUP155	0,001	0,687	0,867	0,174	0,17	-0,037	2741,43	471,921
ENC1	0,001	0,688	0,867	0,11	0,106	-0,048	2327,884	251,241
CHCHD3	0,002	0,69	0,867	0,245	0,231	-0,081	341,128	80,996
TRAM1	0,001	0,687	0,867	0,372	0,381	0,033	1964,684	739,671
RRP8	0,003	0,688	0,867	0,209	0,194	-0,11	209,376	42,169
KDM2A	0,002	0,689	0,867	0,051	0,053	0,069	1880,868	97,693
TMPO	0,001	0,687	0,867	0,14	0,144	0,036	3377,227	480,962
CEP170B	0,003	0,689	0,867	0,062	0,057	-0,106	708,119	42,371
ACACA	0,002	0,689	0,867	0,068	0,072	0,075	1196,684	83,514
ENDOV	0,003	0,688	0,867	0,335	0,31	-0,115	128,834	41,595
CCDC124	0,001	0,689	0,867	0,284	0,292	0,042	1320,529	380,031
UQCRC1	0,001	0,69	0,868	0,466	0,478	0,036	1240,103	584,663
PKP3	0,002	0,691	0,869	0,148	0,157	0,084	458,742	69,805
PRKCDBP	0,001	0,692	0,869	0,78	0,759	-0,038	808,556	623,52
DHRS7	0,001	0,691	0,869	0,81	0,771	-0,072	184,073	145,901
CORO1B	0,001	0,692	0,87	0,289	0,281	-0,041	1309,65	373,181
PANK4	0,003	0,694	0,871	0,243	0,26	0,1	209,544	52,409
MFN2	0,001	0,695	0,871	0,117	0,121	0,046	2063,436	245,95
WWC2	0,003	0,695	0,871	0,058	0,054	-0,088	1030,024	57,48
NDST1	0,002	0,693	0,871	0,039	0,037	-0,07	2437,67	91,898
SSR1	0,001	0,694	0,871	0,309	0,317	0,033	2502,724	783,699
LRRC14	0,003	0,695	0,871	0,143	0,135	-0,091	403,645	56,156
TUBA1A	0,003	0,694	0,871	0,201	0,214	0,092	285,427	58,962
YY1	0,001	0,695	0,871	0,208	0,202	-0,041	1757,285	360,355
CYBA	0,001	0,694	0,871	0,955	0,928	-0,041	606,119	572,507
ROCK1	0,002	0,694	0,871	0,095	0,1	0,071	942,405	91,714
ASNA1	0,001	0,694	0,871	0,392	0,38	-0,042	998,263	385,385

DYNLRB1	0,001	0,695	0,871	0,417	0,43	0,044	787,079	333,414
BLCAP	0,001	0,694	0,871	0,197	0,205	0,058	798,117	159,717
TRABD	0,001	0,694	0,871	0,166	0,161	-0,045	1771,459	290,955
TGFBR2	0,001	0,697	0,872	0,127	0,133	0,057	1179,756	152,752
COPB2	0,001	0,696	0,872	0,123	0,119	-0,051	1595,738	192,33
FBXL5	0,002	0,697	0,872	0,272	0,256	-0,084	273,601	71,922
ZDHHC20	0,002	0,697	0,872	0,305	0,289	-0,077	301,589	89,857
OXA1L	0,001	0,696	0,872	0,367	0,378	0,042	975,351	363,401
ANP32A	0,001	0,697	0,872	0,188	0,196	0,055	886,791	170,913
KIAA0100	0,002	0,696	0,872	0,034	0,033	-0,062	3443,114	116,068
PTBP1	0,001	0,697	0,872	0,138	0,135	-0,031	7178,712	979,69
BACH1	0,003	0,696	0,872	0,177	0,19	0,101	253,611	46,538
LRRC40	0,002	0,7	0,873	0,216	0,206	-0,071	457,672	96,283
ARNT	0,002	0,698	0,873	0,152	0,16	0,072	586,753	91,235
ATF6	0,001	0,699	0,873	0,124	0,129	0,061	971,347	123,147
LCLAT1	0,001	0,699	0,873	0,409	0,391	-0,065	335,519	133,74
BCS1L	0,002	0,699	0,873	0,285	0,301	0,081	263,154	77,043
USP38	0,002	0,702	0,873	0,231	0,243	0,069	430,213	102,082
NEIL3	0,003	0,699	0,873	0,237	0,222	-0,095	231,358	52,905
PJA2	0,001	0,7	0,873	0,122	0,127	0,062	975,605	121,668
PPIC	0,003	0,7	0,873	0,939	0,869	-0,112	63,66	57,909
FKBP5	0,003	0,701	0,873	0,116	0,123	0,09	455,13	54,666
FBXO30	0,002	0,7	0,873	0,254	0,268	0,078	313,749	81,974
PIGO	0,001	0,702	0,873	0,487	0,506	0,055	393,341	195,006
CHD4	0,001	0,701	0,873	0,042	0,044	0,05	4231,722	182,143
CLSTN3	0,003	0,701	0,873	0,168	0,18	0,102	255,291	44,225
DIP2B	0,003	0,699	0,873	0,076	0,071	-0,091	702,057	51,47
METTL3	0,002	0,7	0,873	0,235	0,222	-0,082	316,395	72,32
YLPM1	0,002	0,699	0,873	0,035	0,033	-0,078	1997,936	68,701
CHST14	0,002	0,7	0,873	0,441	0,417	-0,079	204,669	87,691
FANCI	0,001	0,698	0,873	0,204	0,212	0,057	743,377	155,211
CIB1	0,001	0,698	0,873	0,499	0,479	-0,061	329,37	160,862

DHPS	0,001	0,702	0,873	0,539	0,516	-0,063	283,092	149,316
THAP7	0,003	0,698	0,873	0,209	0,224	0,099	222,971	48,384
PMM1	0,002	0,698	0,873	0,437	0,463	0,082	185,079	83,293
DMAP1	0,002	0,704	0,874	0,181	0,172	-0,073	493,515	87,138
CTTNBP2NL	0,002	0,703	0,874	0,118	0,124	0,08	570,843	69,04
SH3BP4	0,001	0,703	0,874	0,13	0,134	0,04	2544,335	336,719
HSD17B11	0,001	0,704	0,874	0,54	0,519	-0,056	360,468	190,467
CDKN2AIP	0,002	0,703	0,874	0,322	0,303	-0,09	195,571	60,996
PPWD1	0,002	0,703	0,874	0,221	0,21	-0,071	441,947	95,129
CDYL	0,003	0,704	0,874	0,129	0,137	0,086	436,815	58,244
MPC1	0,002	0,704	0,874	0,576	0,607	0,076	174,054	103,31
ZC3HC1	0,002	0,702	0,874	0,368	0,392	0,091	166,478	63,199
CCDC34	0,003	0,702	0,874	0,227	0,211	-0,105	196,004	42,985
TMEM138	0,001	0,703	0,874	0,435	0,453	0,059	370,068	164,291
CLK3	0,003	0,703	0,874	0,128	0,137	0,093	379,687	50,186
UBFD1	0,002	0,703	0,874	0,131	0,125	-0,075	609,108	77,873
WBP5	0,001	0,702	0,874	0,311	0,323	0,052	629,718	199,266
SLC30A6	0,002	0,706	0,875	0,271	0,283	0,067	397,798	110,529
INPP4A	0,003	0,706	0,875	0,087	0,081	-0,097	518,425	43,481
TMEM43	0,001	0,706	0,875	0,241	0,235	-0,04	1503,172	358,376
TSPYL1	0,001	0,705	0,875	0,263	0,252	-0,06	547,793	141,006
SHMT2	0,001	0,706	0,875	0,282	0,276	-0,033	2605,832	726,59
POP5	0,002	0,706	0,875	0,624	0,586	-0,091	117,801	70,732
GINS1	0,002	0,706	0,875	0,271	0,257	-0,077	311,019	81,807
PARP1	0,001	0,707	0,876	0,059	0,061	0,049	3137,372	189,234
ZNF638	0,002	0,708	0,876	0,05	0,053	0,081	1185,679	60,831
CHIC2	0,003	0,708	0,876	0,487	0,456	-0,095	124,346	58,524
BASP1	0,001	0,708	0,876	0,276	0,267	-0,047	856,922	232,411
FAM114A2	0,002	0,707	0,876	0,454	0,427	-0,086	159,286	69,912
FIS1	0,002	0,708	0,876	0,343	0,361	0,076	252,576	88,601
INCENP	0,001	0,708	0,876	0,059	0,057	-0,059	1995,484	115,841
NPRL3	0,001	0,708	0,876	0,172	0,179	0,059	778,427	136,144

PCIF1	0,002	0,708	0,876	0,198	0,188	-0,07	488,014	94,432
GBE1	0,001	0,71	0,877	0,325	0,315	-0,044	876,919	280,867
ATR	0,001	0,709	0,877	0,189	0,197	0,057	726,25	140,13
PTGR1	0,001	0,709	0,877	0,459	0,439	-0,063	317,108	142,938
PPM1A	0,002	0,71	0,877	0,163	0,172	0,076	447,011	74,93
SLC25A39	0,001	0,71	0,877	0,14	0,135	-0,048	1521,356	209,292
ELP2	0,003	0,71	0,877	0,219	0,234	0,095	214,114	48,593
GPR107	0,001	0,711	0,878	0,427	0,444	0,057	373,718	162,907
CLCN7	0,002	0,711	0,878	0,237	0,226	-0,063	497,001	115,027
WDR45B	0,002	0,711	0,878	0,102	0,098	-0,059	1208,3	121,133
INPP5B	0,003	0,712	0,879	0,234	0,219	-0,096	205,909	46,575
PGM1	0,001	0,713	0,879	0,18	0,173	-0,056	824,305	145,511
SMIM4	0,003	0,712	0,879	1,457	1,583	0,12	38,825	58,545
SERPINB6	0,001	0,713	0,879	0,435	0,419	-0,052	474,57	202,558
EMC2	0,002	0,713	0,879	0,352	0,373	0,082	201,403	72,773
UBAP1	0,002	0,713	0,879	0,179	0,171	-0,07	507,759	88,996
PEX14	0,002	0,714	0,88	0,276	0,292	0,083	251,468	70,896
CDC25A	0,001	0,715	0,88	0,167	0,173	0,056	811,49	138,214
CCDC51	0,002	0,714	0,88	0,348	0,368	0,079	216,111	77,197
ACO1	0,001	0,715	0,88	0,193	0,187	-0,044	1288,312	244,676
PLCB3	0,001	0,714	0,88	0,145	0,14	-0,046	1596,195	228,113
NUCB1	0,001	0,714	0,88	0,347	0,356	0,041	939,75	330,222
DBT	0,002	0,716	0,881	0,142	0,135	-0,077	481,889	66,586
GOLGA4	0,002	0,715	0,881	0,043	0,045	0,064	2236,037	97,614
ACAP2	0,002	0,715	0,881	0,128	0,122	-0,07	671,21	83,947
TRIM5	0,002	0,716	0,881	0,152	0,161	0,079	429,234	66,914
ACIN1	0,001	0,715	0,881	0,128	0,124	-0,041	2285,453	288,153
NSF	0,003	0,716	0,881	0,094	0,089	-0,08	662,768	60,663
PLXNA1	0,002	0,717	0,882	0,073	0,076	0,07	1055,034	79,069
CENPK	0,002	0,718	0,882	0,388	0,371	-0,067	278,728	105,623
TMEM106B	0,002	0,717	0,882	0,271	0,283	0,065	390,218	108,214
ETV1	0,003	0,718	0,882	0,16	0,17	0,086	337,867	55,565

C2CD2L	0,003	0,717	0,882	0,123	0,115	-0,097	359,271	42,881
EDEM1	0,002	0,719	0,883	0,066	0,062	-0,079	943,757	60,482
MRPS33	0,003	0,719	0,883	0,546	0,512	-0,093	111,028	58,691
IKBKAP	0,002	0,719	0,883	0,103	0,098	-0,065	938,172	94,087
GHITM	0,001	0,719	0,883	0,525	0,535	0,027	2017,735	1069,394
CTNNBL1	0,001	0,719	0,883	0,184	0,191	0,056	734,01	137,579
SERTAD2	0,001	0,72	0,884	0,108	0,105	-0,042	2336,241	248,319
STARD7	0,001	0,721	0,884	0,075	0,078	0,052	1960,215	149,636
NCKAP1	0,001	0,72	0,884	0,133	0,136	0,039	2345,563	315,726
PHLDB2	0,002	0,72	0,884	0,084	0,087	0,063	1183,197	101,708
DAP	0,001	0,72	0,884	0,088	0,091	0,04	3038,317	271,491
RAD1	0,002	0,72	0,884	0,163	0,171	0,076	415,711	69,556
RB1	0,001	0,721	0,884	0,127	0,131	0,04	2215,912	286,637
RAB8B	0,002	0,721	0,884	0,141	0,135	-0,062	743,087	102,087
PPIE	0,001	0,723	0,885	0,332	0,342	0,046	699,434	235,864
VTA1	0,001	0,722	0,885	0,282	0,272	-0,05	670,686	185,637
MTMR2	0,001	0,723	0,885	0,092	0,088	-0,052	1593,557	143,275
SPRY2	0,002	0,723	0,885	0,183	0,192	0,073	409,62	76,405
TXNDC17	0,001	0,722	0,885	1,135	1,169	0,042	374,251	431,005
GHDC	0,002	0,722	0,885	0,328	0,342	0,062	354,111	118,823
GGCX	0,001	0,725	0,886	0,414	0,426	0,042	696,541	292,937
ESYT2	0,001	0,724	0,886	0,116	0,121	0,054	1180,673	139,985
TMEM5	0,003	0,724	0,886	0,181	0,193	0,093	239,654	44,842
NAA20	0,001	0,724	0,886	0,387	0,401	0,05	474,619	187,343
SOGA1	0,002	0,725	0,886	0,023	0,022	-0,065	3851,015	85,587
SMS	0,002	0,724	0,886	0,172	0,164	-0,069	603,917	102,505
C9orf89	0,002	0,726	0,887	0,456	0,434	-0,073	209,014	93,537
SPTSSA	0,002	0,725	0,887	0,351	0,372	0,08	190,577	68,813
FOXJ3	0,001	0,727	0,888	0,105	0,109	0,05	1497,624	160,452
FAM136A	0,001	0,728	0,888	0,122	0,117	-0,053	1170,437	139,73
MYEOV2	0,001	0,728	0,888	1,597	1,518	-0,074	89,233	139,036
RUVBL1	0,001	0,728	0,888	0,328	0,319	-0,041	875,615	283,381

UTP23	0,003	0,727	0,888	0,14	0,148	0,084	357,449	51,536
NINJ1	0,004	0,729	0,888	0,225	0,241	0,102	164,991	38,202
RPL35	0,001	0,728	0,888	1,098	1,117	0,025	1612,55	1783,547
LDB1	0,002	0,726	0,888	0,172	0,179	0,06	627,38	109,741
DEAF1	0,003	0,728	0,888	0,125	0,132	0,081	429,372	55,237
WBP11	0,001	0,727	0,888	0,08	0,083	0,055	1542,772	125,501
FRMD6	0,001	0,727	0,888	0,165	0,162	-0,029	3642,498	595,078
DPY19L3	0,003	0,728	0,888	0,145	0,137	-0,081	403,345	56,327
ZHX3	0,002	0,728	0,888	0,077	0,081	0,061	1232,641	97,029
C2orf47	0,002	0,73	0,889	0,622	0,652	0,069	169,459	107,684
REPIN1	0,003	0,729	0,889	0,074	0,07	-0,079	775,901	56,253
MMS19	0,001	0,73	0,889	0,108	0,104	-0,049	1471,174	155,834
C19orf48	0,001	0,729	0,889	0,175	0,182	0,054	754,893	134,773
ANKRD54	0,003	0,73	0,889	0,166	0,157	-0,08	357,629	57,753
CCNL2	0,002	0,731	0,89	0,133	0,14	0,075	451,739	61,844
MGAT4B	0,002	0,731	0,89	0,123	0,117	-0,066	742,596	88,363
MFSD2A	0,002	0,732	0,891	0,513	0,536	0,064	214,585	112,485
GNAI1	0,003	0,733	0,891	0,242	0,257	0,087	201,94	50,325
GBF1	0,001	0,733	0,891	0,104	0,107	0,042	2215,5	232,683
APBB1	0,004	0,732	0,891	0,214	0,198	-0,111	168,356	34,99
CACNA2D4	0,002	0,733	0,891	0,13	0,136	0,065	647	85,796
DSN1	0,001	0,733	0,891	0,357	0,346	-0,045	622,654	218,258
TOMM22	0,001	0,733	0,891	0,297	0,307	0,048	612,065	185,182
GLRX2	0,002	0,737	0,892	1,01	1,067	0,079	87,643	91,054
TAF1B	0,001	0,735	0,892	0,349	0,363	0,055	399,761	142,297
SLC20A1	0,001	0,738	0,892	0,206	0,201	-0,041	1280,345	259,131
NUP35	0,002	0,736	0,892	0,505	0,484	-0,06	254,038	125,577
ACTR8	0,002	0,739	0,892	0,181	0,19	0,065	441,905	82,009
GSTCD	0,003	0,736	0,892	0,171	0,162	-0,086	284,723	47,302
LSM6	0,003	0,737	0,892	0,485	0,515	0,086	114,904	57,711
ALDH7A1	0,002	0,736	0,892	0,263	0,25	-0,074	267,406	68,657
PAK1IP1	0,001	0,735	0,892	0,289	0,299	0,049	579,195	170,793

MDN1	0,002	0,737	0,892	0,07	0,067	-0,059	1428,141	97,912
LRWD1	0,002	0,737	0,892	0,217	0,207	-0,07	349,084	74,083
ST6GALNAC4	0,002	0,736	0,892	0,145	0,137	-0,079	431,594	61,228
FAM208B	0,001	0,734	0,892	0,08	0,083	0,046	2089,965	171,176
FBXO18	0,003	0,737	0,892	0,108	0,102	-0,082	472,041	49,389
ZFYVE27	0,003	0,736	0,892	0,155	0,164	0,083	320,131	51,17
WDR11	0,002	0,737	0,892	0,163	0,169	0,058	659,694	109,729
FAM175B	0,002	0,737	0,892	0,185	0,177	-0,069	413,624	74,819
MED19	0,002	0,737	0,892	0,298	0,312	0,068	274,265	83,686
MSANTD4	0,003	0,739	0,892	0,139	0,131	-0,086	335,72	45,132
NCAPD2	0,001	0,736	0,892	0,188	0,185	-0,024	5958,523	1109,498
ZDHHC17	0,003	0,735	0,892	0,216	0,204	-0,084	249,586	52,216
C12orf45	0,002	0,736	0,892	0,731	0,777	0,088	86,22	64,763
BMP4	0,002	0,739	0,892	0,212	0,201	-0,076	314,537	65,434
TMED8	0,003	0,735	0,892	0,069	0,065	-0,08	759,111	50,577
FAM65A	0,001	0,736	0,892	0,162	0,165	0,035	2163,491	353,535
LASP1	0,001	0,737	0,892	0,147	0,15	0,026	5249,462	780,945
PPP1R9B	0,003	0,734	0,892	0,046	0,044	-0,077	1234,341	55,518
FTSJ3	0,002	0,739	0,892	0,065	0,067	0,053	1817,914	120,294
TXNL4A	0,002	0,734	0,892	0,36	0,379	0,073	212,533	78,389
UBL5	0,001	0,735	0,892	1,171	1,135	-0,046	276,69	318,759
IRF2BP1	0,002	0,737	0,892	0,206	0,217	0,075	304,4	64,224
RIN2	0,001	0,736	0,892	0,247	0,255	0,05	618,03	155,25
RBBP4	0,001	0,741	0,893	0,389	0,375	-0,052	402,249	153,338
IER5	0,001	0,739	0,893	0,37	0,384	0,055	365,669	137,717
FH	0,001	0,74	0,893	0,542	0,528	-0,036	732,68	392,107
LIMS2	0,005	0,74	0,893	0,152	0,163	0,102	219,455	34,059
TMBIM1	0,001	0,741	0,893	0,347	0,337	-0,041	762,902	261,196
EXOSC9	0,002	0,741	0,893	0,227	0,236	0,057	490,932	113,756
ACBD5	0,003	0,74	0,893	0,174	0,183	0,077	321,21	57,353
CPPED1	0,002	0,74	0,893	0,278	0,267	-0,059	401,003	109,366
FARSA	0,001	0,74	0,893	0,312	0,318	0,03	1811,891	570,708

PSMF1	0,001	0,74	0,893	0,276	0,283	0,034	1402,344	392,555
MKKS	0,001	0,74	0,893	0,276	0,286	0,054	466,655	131,405
PLEKHB2	0,001	0,742	0,894	0,208	0,203	-0,036	1541,329	316,135
SRF	0,002	0,742	0,894	0,075	0,078	0,063	1058,438	80,816
LRSAM1	0,003	0,743	0,894	0,186	0,176	-0,084	271,02	49,23
MADD	0,002	0,742	0,894	0,102	0,106	0,059	938,204	97,189
GLIPR1	0,002	0,742	0,894	0,379	0,399	0,074	186,641	72,238
UBC	0,001	0,742	0,894	0,278	0,27	-0,041	872,626	238,687
RALBP1	0,002	0,743	0,894	0,093	0,089	-0,055	1196,317	108,245
ATG4C	0,003	0,746	0,895	0,271	0,287	0,083	177,285	49,554
SLC25A44	0,003	0,746	0,895	0,094	0,089	-0,073	625,859	57,397
BLZF1	0,003	0,745	0,895	0,209	0,197	-0,085	228,968	46,617
SLC41A1	0,002	0,747	0,895	0,084	0,08	-0,062	1015,072	83,786
SLC5A6	0,001	0,744	0,895	0,41	0,399	-0,04	668,737	270,115
TIPARP	0,001	0,746	0,895	0,193	0,187	-0,047	826,774	156,925
ENOPH1	0,001	0,744	0,895	0,249	0,24	-0,051	584,884	142,789
CCDC109B	0,003	0,747	0,895	0,205	0,194	-0,082	244,341	48,792
NIPBL	0,001	0,745	0,895	0,068	0,07	0,05	1826,56	126,316
CDC25C	0,003	0,746	0,895	0,256	0,243	-0,077	231,262	57,649
HSPA9	0,001	0,747	0,895	0,17	0,167	-0,026	4475,407	756,275
CHCHD7	0,003	0,745	0,895	0,391	0,413	0,078	154,763	62,341
ASPH	0,001	0,746	0,895	0,197	0,201	0,03	2373,307	472,667
PIP4K2A	0,003	0,743	0,895	0,081	0,076	-0,086	533,418	42,04
CCDC6	0,001	0,745	0,895	0,15	0,145	-0,041	1375,343	202,785
FOXJ2	0,003	0,746	0,895	0,103	0,098	-0,073	595,18	60,211
ATP5G2	0,001	0,747	0,895	1,167	1,194	0,033	533,481	629,141
CIT	0,001	0,745	0,895	0,079	0,082	0,047	1866,215	149,961
MPHOSPH6	0,002	0,745	0,895	0,376	0,359	-0,064	252,51	92,531
ABR	0,001	0,747	0,895	0,086	0,083	-0,043	2366,808	200,643
RNF167	0,001	0,747	0,895	0,208	0,202	-0,041	1075,812	220,654
EVPL	0,002	0,746	0,895	0,055	0,052	-0,072	1068,847	57,304
RBM42	0,001	0,747	0,895	0,605	0,619	0,031	855,783	524,022

TTPAL	0,003	0,746	0,895	0,1	0,095	-0,074	580,766	56,721
OGFR	0,002	0,745	0,895	0,138	0,143	0,054	815,829	114,546
NOL9	0,002	0,749	0,896	0,174	0,181	0,054	622,901	110,838
DFFA	0,001	0,749	0,896	0,134	0,138	0,044	1234,229	167,965
ASXL2	0,003	0,75	0,896	0,046	0,049	0,073	1118,266	53,133
GORASP1	0,002	0,75	0,896	0,156	0,164	0,07	398,211	63,661
HTT	0,001	0,75	0,896	0,15	0,154	0,043	1216,246	185,051
KIAA0947	0,002	0,749	0,896	0,069	0,066	-0,062	1167,419	78,759
CNOT6	0,002	0,748	0,896	0,103	0,099	-0,064	751,424	75,938
TEX10	0,002	0,748	0,896	0,167	0,161	-0,053	719,158	118,229
FJX1	0,001	0,749	0,896	0,175	0,169	-0,049	817,51	140,815
CORO1C	0,001	0,75	0,896	0,152	0,154	0,026	4167,252	637,734
MARK3	0,002	0,75	0,896	0,077	0,081	0,062	1028,756	81,065
TLDC1	0,002	0,748	0,896	0,192	0,182	-0,072	339,514	63,537
ERAL1	0,001	0,748	0,896	0,236	0,228	-0,049	659,184	153,222
PAK2	0,001	0,751	0,897	0,17	0,165	-0,039	1413,499	236,194
CHORDC1	0,002	0,751	0,897	0,15	0,144	-0,057	670,673	98,417
ORC6	0,001	0,752	0,897	0,387	0,4	0,047	453,37	178,385
GABARAPL2	0,001	0,751	0,897	0,702	0,729	0,055	209,6	150,203
SPRTN	0,003	0,754	0,898	0,177	0,186	0,074	311,681	56,608
TRAPPC12	0,001	0,753	0,898	0,479	0,497	0,052	298,312	145,359
SPR	0,001	0,752	0,898	0,299	0,307	0,038	869,261	262,813
STK25	0,002	0,752	0,898	0,115	0,111	-0,051	1071,922	121,132
PIDD	0,003	0,753	0,898	0,126	0,119	-0,08	367,55	45,063
PPME1	0,001	0,754	0,898	0,173	0,17	-0,031	2330,688	399,354
RPL4	0,001	0,754	0,898	0,216	0,212	-0,027	2984,579	639,22
NDE1	0,003	0,753	0,898	0,128	0,122	-0,078	394,979	49,472
CISD3	0,003	0,754	0,898	0,345	0,325	-0,083	145,959	48,842
PLEKHM2	0,003	0,757	0,899	0,093	0,088	-0,072	612,734	55,47
CDC42SE1	0,001	0,757	0,899	0,134	0,129	-0,05	943,837	124,294
TSEN15	0,002	0,755	0,899	0,216	0,226	0,06	429,41	94,465
SUPT7L	0,002	0,755	0,899	0,166	0,173	0,064	437,434	74,058

SF3B5	0,001	0,756	0,899	0,924	0,901	-0,035	465,373	424,658
INTS10	0,002	0,754	0,899	0,188	0,198	0,072	312,117	60,256
SLC25A32	0,001	0,755	0,899	0,289	0,3	0,051	445,194	131,341
SLC25A29	0,003	0,756	0,899	0,188	0,199	0,082	235,762	45,51
HEATR3	0,003	0,756	0,899	0,137	0,131	-0,071	433,527	57,973
HSDL1	0,003	0,754	0,899	0,207	0,219	0,083	217,11	46,507
CNTNAP1	0,003	0,756	0,899	0,192	0,203	0,08	243,21	47,792
ME2	0,003	0,755	0,899	0,18	0,171	-0,075	304,67	53,605
INSR	0,003	0,755	0,899	0,179	0,189	0,078	272,782	50,166
SUMO3	0,001	0,755	0,899	0,199	0,204	0,04	1059,108	213,677
CRELD2	0,001	0,755	0,899	0,254	0,245	-0,049	563,648	140,873
SLC25A5	0,001	0,756	0,899	0,542	0,55	0,02	3353,162	1831,206
OCRL	0,003	0,756	0,899	0,115	0,109	-0,077	430,181	47,875
GNB4	0,002	0,76	0,9	0,251	0,261	0,055	414,473	106,234
UBA6	0,001	0,759	0,9	0,233	0,239	0,037	1046,803	247,15
GAR1	0,001	0,759	0,9	0,465	0,481	0,05	313,092	148,392
CREB5	0,004	0,759	0,9	0,158	0,167	0,087	243,833	39,369
GBAS	0,003	0,758	0,9	0,272	0,257	-0,082	176,101	46,717
ABCF2	0,002	0,759	0,9	0,072	0,075	0,057	1172,793	86,362
FAM160A2	0,003	0,759	0,9	0,201	0,191	-0,08	236,488	46,397
AKIP1	0,002	0,758	0,9	0,609	0,585	-0,059	194,551	116,408
COX8A	0,001	0,76	0,9	0,491	0,502	0,032	846,646	419,827
GTF2H3	0,001	0,759	0,9	0,271	0,262	-0,049	499,39	133,053
PPP2R5C	0,001	0,76	0,9	0,173	0,168	-0,041	1119,047	190,338
TGFB1	0,001	0,76	0,9	0,186	0,191	0,041	1007,48	189,813
ARFRP1	0,003	0,759	0,9	0,174	0,166	-0,072	327,986	55,773
GUK1	0,002	0,761	0,901	0,103	0,098	-0,064	669,514	67,517
CENPO	0,002	0,764	0,901	0,179	0,171	-0,063	401,441	70,171
AFF1	0,002	0,762	0,901	0,106	0,101	-0,064	642,488	66,732
HMGCS1	0,002	0,761	0,901	0,239	0,228	-0,065	305,496	71,31
SNAI2	0,002	0,762	0,901	0,232	0,243	0,065	325,294	76,703
ATAD2	0,001	0,762	0,901	0,036	0,037	0,047	3386,922	123,389

SARDH	0,003	0,763	0,901	0,236	0,223	-0,082	206,618	47,735
PHLDA2	0,001	0,763	0,901	0,555	0,566	0,03	864,341	484,465
CERS5	0,002	0,763	0,901	0,348	0,332	-0,068	207,2	70,343
AAAS	0,002	0,763	0,901	0,369	0,384	0,056	283,686	106,778
CFL2	0,002	0,764	0,901	0,217	0,209	-0,052	511,485	108,972
PSMC6	0,001	0,763	0,901	0,577	0,56	-0,043	378,477	214,821
FCF1	0,002	0,762	0,901	0,163	0,156	-0,063	450,114	71,624
HMOX2	0,001	0,762	0,901	0,323	0,332	0,043	577,323	188,739
B9D1	0,002	0,761	0,901	0,59	0,56	-0,076	113,382	65,37
SRSF1	0,001	0,763	0,901	0,323	0,318	-0,023	2952,322	946,041
FASN	0,001	0,764	0,901	0,138	0,136	-0,022	7081,392	970,316
SPINT2	0,001	0,763	0,901	0,464	0,454	-0,031	938,404	430,613
DHX34	0,002	0,761	0,901	0,151	0,145	-0,054	672,93	99,749
HMGN1	0,002	0,764	0,901	0,217	0,225	0,05	552,264	122
TAF1	0,002	0,764	0,901	0,125	0,119	-0,064	539,798	65,702
FEZ2	0,002	0,766	0,903	0,213	0,205	-0,055	459,237	96,075
RAPH1	0,002	0,767	0,903	0,291	0,281	-0,053	381,468	108,932
FAM134A	0,002	0,766	0,903	0,124	0,129	0,057	663,052	83,66
USO1	0,002	0,766	0,903	0,128	0,133	0,048	926,983	121,024
GALNT18	0,003	0,767	0,903	0,234	0,246	0,071	233,959	56,236
FERMT3	0,001	0,767	0,903	0,227	0,223	-0,028	2427,703	547,189
PFDN5	0,001	0,766	0,903	1,053	1,074	0,029	658,624	700,398
STAT6	0,001	0,766	0,903	0,13	0,126	-0,041	1370,926	175,524
EMC7	0,001	0,766	0,903	0,627	0,645	0,041	372,918	237,348
QTRT1	0,001	0,766	0,903	0,508	0,493	-0,043	402,793	201,931
EIF2S2	0,001	0,765	0,903	0,194	0,199	0,035	1337,929	262,662
CYB5R3	0,001	0,767	0,903	0,263	0,258	-0,027	2036,826	531,28
ACAD8	0,002	0,768	0,904	0,246	0,257	0,064	277,584	69,943
GSS	0,001	0,768	0,904	0,37	0,363	-0,028	1370,751	502,189
CPSF3L	0,001	0,771	0,905	0,225	0,232	0,041	759,335	173,512
IGSF8	0,002	0,771	0,905	0,471	0,452	-0,061	186,354	86,001
INPP1	0,003	0,771	0,905	0,162	0,171	0,078	268,393	44,464

HSPD1	0,001	0,769	0,905	0,13	0,127	-0,027	3419,992	439,944
MLH1	0,001	0,771	0,905	0,165	0,17	0,046	767,124	128,498
SAP30	0,003	0,77	0,905	0,77	0,726	-0,084	71,671	53,283
PFDN1	0,002	0,771	0,905	0,234	0,226	-0,05	480,599	110,403
RBM22	0,002	0,771	0,905	0,125	0,13	0,05	844,066	107,793
RPL10A	0,001	0,769	0,905	1,002	0,982	-0,029	640,688	635,508
NUDT1	0,002	0,77	0,905	0,573	0,599	0,066	134,946	79,066
LRR1	0,002	0,77	0,905	0,354	0,341	-0,052	331,237	115,001
PSEN1	0,001	0,771	0,905	0,236	0,229	-0,042	695,586	161,474
NEDD4	0,002	0,77	0,905	0,094	0,097	0,054	914,395	87,187
FITM2	0,002	0,769	0,905	0,295	0,309	0,066	227,871	68,891
TUFT1	0,002	0,773	0,906	0,18	0,187	0,057	434,579	79,856
FLVCR1	0,003	0,773	0,906	0,3	0,314	0,069	196,44	60,225
LRPPRC	0,001	0,774	0,906	0,186	0,189	0,025	2901,54	544,4
H2AFZ	0,001	0,773	0,906	0,737	0,751	0,027	864,013	643,656
RGMB	0,001	0,773	0,906	0,123	0,119	-0,046	1103,438	134,375
HDAC3	0,002	0,772	0,906	0,191	0,197	0,048	628,104	121,602
TXNDC5	0,001	0,773	0,906	33,336	29,023	-0,2	4,726	148,363
ENSG00000221500.1	0,002	0,772	0,906	37,761	31,851	-0,246	3,013	104,362
LGR4	0,002	0,773	0,906	0,196	0,189	-0,049	581,492	111,737
FAM111A	0,002	0,773	0,906	0,078	0,075	-0,063	797,465	60,759
KATNAL1	0,003	0,774	0,906	0,074	0,07	-0,071	647,075	46,63
TXNL1	0,001	0,774	0,906	0,452	0,437	-0,049	296,403	131,444
DIAPH2	0,003	0,774	0,906	0,116	0,121	0,069	437,072	51,785
CENPI	0,002	0,774	0,906	0,182	0,175	-0,057	458,975	81,63
STAG2	0,001	0,774	0,906	0,094	0,097	0,045	1346,03	129,083
UBIAD1	0,002	0,776	0,907	0,226	0,235	0,055	392,836	90,517
MIIP	0,003	0,776	0,907	0,239	0,228	-0,068	241,273	56,373
EIF3I	0,001	0,779	0,907	0,609	0,619	0,022	1695,305	1040,705
TARS2	0,002	0,777	0,907	0,247	0,256	0,056	343,669	86,425
CHTOP	0,001	0,775	0,907	0,178	0,173	-0,041	925,83	162,718
ANXA4	0,002	0,778	0,907	0,321	0,31	-0,051	342,672	108,252

SNRPG	0,002	0,778	0,907	0,785	0,749	-0,067	103,708	79,513
EIF1B	0,002	0,778	0,907	0,495	0,513	0,051	232,718	117,45
ALG3	0,001	0,779	0,907	0,458	0,447	-0,038	506,373	229,571
MLF1IP	0,001	0,779	0,907	0,259	0,267	0,043	563,466	148,481
RHOBTB3	0,002	0,778	0,907	0,11	0,113	0,047	1017,535	113,199
SLC29A1	0,001	0,777	0,907	0,549	0,559	0,026	1110,508	615,008
LMBR1	0,002	0,777	0,907	0,145	0,151	0,06	451,148	66,724
CWF19L1	0,002	0,779	0,907	0,253	0,244	-0,056	335,069	83,083
RASA3	0,002	0,778	0,907	0,105	0,102	-0,046	1094,751	113,589
CALM1	0,001	0,775	0,907	0,327	0,321	-0,024	2243,865	725,93
BFAR	0,002	0,775	0,907	0,193	0,187	-0,048	599,345	113,638
ITGAE	0,002	0,776	0,907	0,39	0,407	0,063	187,807	74,884
G6PC3	0,001	0,775	0,907	0,598	0,613	0,034	538,876	325,937
CANT1	0,001	0,778	0,907	0,14	0,144	0,042	1070,381	151,779
FAM98C	0,003	0,775	0,907	0,514	0,487	-0,077	102,91	51,466
PRNP	0,001	0,776	0,907	0,302	0,297	-0,024	2271,319	680,579
ACSL4	0,001	0,777	0,907	0,13	0,133	0,028	3044,735	400,424
IPO11	0,001	0,78	0,908	0,274	0,282	0,038	720,355	200,432
PRRC1	0,001	0,78	0,908	0,307	0,313	0,032	932,043	288,9
BLVRA	0,002	0,78	0,908	0,596	0,622	0,063	131,963	80,411
IFITM3	0,001	0,78	0,908	1,051	1,069	0,024	872,157	923,91
C14orf119	0,001	0,78	0,908	0,558	0,542	-0,043	333,341	183,45
THUMPD1	0,003	0,78	0,908	0,099	0,104	0,068	486,453	49,561
RICTOR	0,003	0,783	0,91	0,078	0,082	0,065	667,27	53,616
COL17A1	0,003	0,782	0,91	0,319	0,335	0,068	172,213	56,379
SLC43A1	0,003	0,782	0,91	0,34	0,359	0,077	131,275	45,629
RBMX	0,003	0,782	0,91	0,044	0,042	-0,066	1125,256	48,777
PRUNE	0,003	0,784	0,911	0,203	0,193	-0,073	228,488	45,144
UBE2K	0,001	0,784	0,911	0,279	0,285	0,033	921,264	259,732
UBE2V2	0,001	0,784	0,911	0,318	0,311	-0,034	789,804	248,46
VEZF1	0,002	0,783	0,911	0,078	0,081	0,05	1122,413	89,239
ANAPC11	0,001	0,785	0,911	1,056	1,027	-0,04	239,465	249,586

RCC1	0,001	0,787	0,912	0,16	0,156	-0,035	1281,441	202,267
ENSG00000197989.9	0,001	0,786	0,912	1,5	1,453	-0,046	148,805	219,266
DEPDC1	0,002	0,787	0,912	0,091	0,088	-0,047	1173,193	105,203
TBC1D5	0,003	0,785	0,912	0,084	0,081	-0,062	680,036	56,271
FSTL1	0,001	0,786	0,912	0,225	0,222	-0,022	3086,475	689,226
MND1	0,003	0,786	0,912	0,51	0,487	-0,069	119,33	59,387
REEP4	0,001	0,786	0,912	0,414	0,402	-0,041	416,066	169,693
SIX1	0,002	0,786	0,912	0,242	0,232	-0,06	286,07	67,777
TNNT1	0,003	0,786	0,912	0,137	0,13	-0,069	353,897	47,289
DOCK7	0,001	0,789	0,913	0,142	0,146	0,039	1116,4	160,678
SMYD3	0,002	0,789	0,913	0,493	0,474	-0,058	174,147	84,421
SHQ1	0,003	0,788	0,913	0,22	0,23	0,066	239,324	53,75
SPRY4	0,002	0,789	0,913	0,121	0,117	-0,05	731,138	86,752
PCMT1	0,001	0,788	0,913	0,651	0,665	0,031	536,53	353,334
NAA38	0,002	0,789	0,913	0,471	0,454	-0,051	222,769	102,93
ARL2	0,002	0,789	0,913	0,247	0,255	0,048	459,384	114,84
SC5D	0,001	0,788	0,913	0,5	0,516	0,045	285,221	145,045
DOCK9	0,003	0,789	0,913	0,08	0,083	0,061	678,302	55,418
TBC1D10B	0,001	0,787	0,913	0,221	0,215	-0,041	670,813	146,197
SLC7A6	0,002	0,788	0,913	0,247	0,255	0,045	477,059	119,752
FAM73A	0,003	0,792	0,914	0,185	0,177	-0,062	313,019	56,344
SNAP47	0,003	0,793	0,914	0,217	0,226	0,063	251,998	55,834
ADI1	0,001	0,792	0,914	0,212	0,217	0,038	763,986	164,134
TNS1	0,002	0,791	0,914	0,108	0,113	0,059	597,309	65,736
MFF	0,002	0,792	0,914	0,316	0,306	-0,046	372,114	115,501
TMEM39A	0,002	0,79	0,914	0,302	0,292	-0,051	316,709	93,888
GSK3B	0,002	0,79	0,914	0,125	0,121	-0,05	690,412	85,003
PPID	0,002	0,792	0,914	0,273	0,264	-0,047	411,911	110,615
MAT2B	0,001	0,79	0,914	0,393	0,383	-0,038	510,087	197,33
TGFBR1	0,002	0,79	0,914	0,115	0,12	0,057	559,246	65,892
ACTR1A	0,001	0,79	0,914	0,254	0,259	0,027	1557,563	398,796
CREB3L1	0,001	0,791	0,914	0,162	0,166	0,037	1093,579	178,834

PACSIN3	0,002	0,792	0,914	0,19	0,198	0,058	357,538	68,982
VWA9	0,003	0,791	0,914	0,215	0,224	0,064	250,828	55,242
C17orf85	0,001	0,792	0,914	0,157	0,161	0,037	1035,608	164,45
PSMC5	0,001	0,792	0,914	0,511	0,519	0,023	1414,716	729,225
FDXR	0,002	0,79	0,914	0,338	0,323	-0,062	191,808	63,471
ASPSCR1	0,002	0,791	0,914	0,369	0,355	-0,058	212,865	77,241
COX6B1	0,001	0,793	0,914	1,197	1,22	0,028	498,243	601,781
UBE2D3	0,001	0,795	0,915	0,103	0,106	0,041	1206,837	126,562
SYNJ2	0,002	0,794	0,915	0,1	0,103	0,049	853,144	86,574
TAF6	0,002	0,795	0,915	0,173	0,179	0,048	533,919	93,907
GSR	0,001	0,794	0,915	0,305	0,313	0,037	616,017	190,272
CA13	0,003	0,794	0,915	0,283	0,297	0,073	149,79	43,295
TBC1D2	0,002	0,793	0,915	0,135	0,131	-0,046	776,864	103,417
CCDC86	0,001	0,794	0,915	0,286	0,28	-0,03	1064,321	301,481
CDR2	0,003	0,795	0,915	0,197	0,206	0,065	252,622	50,865
GPT2	0,003	0,795	0,915	0,102	0,106	0,061	515,041	53,599
SPIRE1	0,002	0,795	0,915	0,091	0,095	0,055	714,879	66,462
ATP5D	0,001	0,795	0,915	1,013	1,03	0,024	760,309	776,239
ARHGEF1	0,003	0,794	0,915	0,158	0,151	-0,062	347,824	53,643
ZDHHC9	0,002	0,794	0,915	0,162	0,156	-0,058	399,898	63,754
TOE1	0,001	0,797	0,916	0,297	0,304	0,036	617,386	185,31
TSPYL4	0,003	0,797	0,916	0,189	0,198	0,069	231,609	44,741
DPY19L4	0,002	0,797	0,916	0,276	0,288	0,059	237,372	67,33
MED4	0,002	0,797	0,916	0,251	0,26	0,054	290,572	74,359
DDX27	0,001	0,797	0,916	0,154	0,158	0,032	1502,146	234,196
TRMT1L	0,002	0,799	0,917	0,271	0,261	-0,056	262,443	69,736
BROX	0,001	0,801	0,917	0,21	0,205	-0,039	679,565	140,774
TTC27	0,002	0,801	0,917	0,201	0,194	-0,056	320,315	63,287
ENSG00000175701.6	0,003	0,8	0,917	1,108	1,047	-0,081	45,002	48,504
ATG4B	0,002	0,799	0,917	0,201	0,195	-0,043	568,683	112,577
LSM3	0,001	0,801	0,917	0,563	0,548	-0,039	320,155	177,608
NBEAL2	0,001	0,8	0,917	0,089	0,086	-0,042	1285,21	112,642

ATP11B	0,002	0,8	0,917	0,093	0,09	-0,052	761,704	69,612
TRIO	0,001	0,801	0,917	0,05	0,051	0,032	3674,47	187,086
MAD2L1BP	0,002	0,8	0,917	0,619	0,641	0,051	165,945	104,43
ENSG00000205903.2	0,003	0,799	0,917	0,361	0,346	-0,063	159,846	56,475
UBAP2	0,002	0,801	0,917	0,075	0,077	0,043	1360,807	103,548
RUSC2	0,001	0,799	0,917	0,108	0,111	0,041	1190,852	129,923
CAT	0,001	0,8	0,917	0,327	0,335	0,035	603,811	199,855
RIN1	0,002	0,799	0,917	0,174	0,168	-0,048	545,182	93,652
WNT5B	0,003	0,798	0,917	0,231	0,24	0,059	259,622	61,141
LDHB	0,001	0,798	0,917	0,522	0,528	0,016	5120,981	2687,789
KRT80	0,001	0,801	0,917	0,111	0,114	0,037	1336,704	150,742
WDFY2	0,002	0,799	0,917	0,162	0,156	-0,057	381,166	60,548
ALG11	0,003	0,801	0,917	0,748	0,787	0,074	64,582	49,677
NEMF	0,003	0,8	0,917	0,069	0,072	0,06	715,313	50,763
CENPV	0,003	0,8	0,917	0,275	0,287	0,063	189,311	53,19
VAPA	0,001	0,801	0,917	0,291	0,297	0,027	1360,179	399,93
SPATC1L	0,003	0,801	0,917	0,452	0,475	0,071	102,253	47,339
C11orf31	0,001	0,803	0,918	0,99	1,017	0,039	223,541	223,987
PIGQ	0,003	0,803	0,918	0,121	0,116	-0,062	410,251	48,82
FAM46B	0,003	0,804	0,919	0,235	0,246	0,066	192,201	46,168
ANTXR1	0,001	0,804	0,919	0,112	0,115	0,033	1650,229	187,22
CDV3	0,001	0,803	0,919	0,11	0,108	-0,033	1711,322	186,724
STX18	0,003	0,804	0,919	0,161	0,168	0,06	316,866	52,122
HPSE	0,003	0,805	0,919	0,251	0,262	0,064	200,245	51,119
ZYX	0,001	0,805	0,919	0,321	0,316	-0,019	2586,579	824,315
PLIN2	0,001	0,805	0,919	0,53	0,542	0,034	420,277	225,487
FEM1B	0,003	0,804	0,919	0,094	0,098	0,058	548,714	52,837
SLC2A4RG	0,003	0,805	0,919	0,111	0,115	0,06	438,214	49,436
PHF5A	0,001	0,804	0,919	0,467	0,456	-0,035	425,551	196,267
YIPF5	0,001	0,806	0,92	0,418	0,428	0,036	438,945	185,683
RREB1	0,003	0,806	0,92	0,078	0,075	-0,061	608,234	46,41
ZNF76	0,003	0,806	0,92	0,074	0,071	-0,058	713,754	51,793

NET1	0,002	0,806	0,92	0,162	0,157	-0,046	571,848	91,026
SLC39A9	0,001	0,807	0,92	0,125	0,122	-0,035	1277,352	157,032
HGS	0,001	0,806	0,92	0,264	0,268	0,025	1493,711	396,647
ECH1	0,002	0,807	0,92	0,441	0,428	-0,045	255,105	110,708
ITPA	0,001	0,807	0,92	0,605	0,622	0,041	247,084	151,431
AURKA	0,001	0,806	0,92	0,184	0,18	-0,028	1621,36	295,743
PSMA5	0,001	0,809	0,921	0,954	0,941	-0,02	1150,392	1089,225
ADCK3	0,002	0,809	0,921	0,309	0,299	-0,046	321,704	97,587
LRRC58	0,001	0,808	0,921	0,103	0,106	0,037	1364,197	142,874
PISD	0,001	0,809	0,921	0,213	0,208	-0,038	622,37	131,136
LAS1L	0,001	0,808	0,921	0,209	0,213	0,027	1391,984	292,936
ENSG00000242125.2	0,001	0,812	0,922	0,465	0,456	-0,028	667,116	307,356
CHCHD5	0,004	0,814	0,922	0,742	0,783	0,077	55,216	41,918
HNMT	0,003	0,812	0,922	0,423	0,443	0,067	108,451	46,8
USP4	0,002	0,81	0,922	0,196	0,19	-0,042	543,791	104,779
ATG3	0,001	0,814	0,922	0,294	0,287	-0,032	680,138	197,355
U2SURP	0,002	0,812	0,922	0,067	0,066	-0,039	1661,743	110,198
TEAD3	0,003	0,814	0,922	0,12	0,115	-0,06	390,845	45,915
AGAP3	0,003	0,81	0,922	0,079	0,083	0,062	543,954	44
NUDCD1	0,001	0,813	0,922	0,216	0,22	0,03	1030,129	224,907
PRPF4	0,001	0,81	0,922	0,25	0,245	-0,029	1091,935	270,043
ODF2	0,002	0,811	0,922	0,054	0,056	0,046	1385,848	76,498
NT5C2	0,003	0,813	0,922	0,221	0,213	-0,056	261,609	56,549
CLNS1A	0,001	0,814	0,922	0,327	0,334	0,032	635,092	209,99
REXO2	0,001	0,812	0,922	0,274	0,281	0,035	597,013	165,486
CHPT1	0,002	0,814	0,922	0,391	0,378	-0,047	237,002	91,17
FNDC3A	0,001	0,813	0,922	0,108	0,11	0,033	1573,433	171,458
CIRH1A	0,001	0,811	0,922	0,276	0,269	-0,036	575,835	156,61
PTRH2	0,001	0,813	0,922	0,619	0,634	0,036	307,601	192,623
WIPI1	0,003	0,811	0,922	0,198	0,208	0,065	210,348	42,559
REEP6	0,002	0,81	0,922	0,256	0,247	-0,049	350,515	88,577
ENSG00000005206.12	0,002	0,814	0,922	0,218	0,225	0,048	357,891	79,133

EEF2	0,001	0,81	0,922	0,185	0,187	0,014	26730,968	4972,53
PPP1R37	0,003	0,813	0,922	0,143	0,137	-0,063	301,762	42,264
EML2	0,003	0,812	0,922	0,213	0,204	-0,061	249,492	52,244
MYL9	0,001	0,811	0,922	1,437	1,473	0,036	189,42	275,188
PRMT2	0,002	0,812	0,922	0,146	0,141	-0,052	451,69	64,825
USP11	0,001	0,811	0,922	0,279	0,285	0,031	775,379	218,755
SRRM1	0,002	0,816	0,923	0,079	0,081	0,039	1353,831	107,827
MARCKSL1	0,002	0,815	0,923	0,367	0,381	0,055	171,333	64,202
DIRC2	0,002	0,815	0,923	0,561	0,584	0,057	116,813	66,838
NDUFS6	0,001	0,816	0,923	1,061	1,083	0,029	332,346	356,05
EED	0,002	0,815	0,923	0,334	0,324	-0,042	343,26	112,745
IPO5	0,001	0,816	0,923	0,222	0,22	-0,015	7190,555	1588,693
PSME1	0,001	0,816	0,923	1,037	1,056	0,027	399,133	417,564
HS3ST3B1	0,003	0,815	0,923	0,13	0,125	-0,056	407,851	51,993
DNAJC7	0,003	0,816	0,923	0,239	0,23	-0,059	216,963	50,699
SLC31A1	0,002	0,818	0,924	0,261	0,254	-0,042	398,453	102,569
CTSH	0,003	0,817	0,924	0,534	0,556	0,06	109,11	59,47
TROVE2	0,001	0,821	0,925	0,197	0,202	0,036	676,431	135,082
RABIF	0,002	0,82	0,925	0,362	0,376	0,052	191,13	70,379
OXNAD1	0,003	0,821	0,925	0,278	0,267	-0,058	184,357	50,033
CYB561D2	0,002	0,818	0,925	0,862	0,898	0,059	80,636	70,877
IFRD1	0,001	0,82	0,925	0,212	0,216	0,028	1157,162	247,869
DPM2	0,002	0,82	0,925	0,225	0,218	-0,048	331,495	73,612
IFIT2	0,001	0,82	0,925	0,129	0,126	-0,035	1063,628	135,577
MRPL23	0,001	0,82	0,925	0,884	0,907	0,037	208,786	186,632
EXT2	0,001	0,818	0,925	0,392	0,384	-0,028	683,367	265,214
ALDH3B1	0,003	0,819	0,925	0,159	0,166	0,064	272,548	44,083
HDAC7	0,002	0,821	0,925	0,135	0,138	0,038	837,502	114,378
MRPS23	0,001	0,82	0,925	0,789	0,772	-0,03	371,98	290,249
LIG1	0,002	0,821	0,925	0,106	0,103	-0,046	701,219	73,099
CTSZ	0,001	0,818	0,925	0,245	0,25	0,027	1165,299	288,475
MRPL40	0,002	0,819	0,925	0,675	0,653	-0,048	146,553	97,248

TYW3	0,003	0,822	0,926	0,067	0,07	0,055	688,502	47,099
STK39	0,002	0,821	0,926	0,258	0,265	0,042	392,518	102,315
KBTBD2	0,002	0,823	0,926	0,159	0,164	0,044	507,605	81,914
UBQLN1	0,001	0,823	0,926	0,291	0,287	-0,018	2770,409	800,456
ABHD13	0,003	0,823	0,926	0,238	0,229	-0,054	234,399	54,744
GTF2A1	0,001	0,822	0,926	0,202	0,199	-0,028	1164,222	233,176
XRCC3	0,002	0,822	0,926	0,129	0,125	-0,046	563,484	71,452
GABPB1	0,002	0,822	0,926	0,253	0,26	0,041	398,856	102,273
CCT8	0,001	0,822	0,926	0,445	0,451	0,018	1900,531	851,139
SNAP29	0,002	0,823	0,926	0,155	0,15	-0,045	500,632	76,404
DDX1	0,001	0,826	0,927	0,184	0,18	-0,027	1310,094	238,155
FAM168B	0,001	0,824	0,927	0,078	0,077	-0,034	1732,493	134,148
TRAK1	0,003	0,825	0,927	0,052	0,05	-0,055	893,372	45,245
AGPAT9	0,001	0,824	0,927	0,296	0,304	0,037	430,302	128,973
PPP1R35	0,003	0,826	0,927	1,283	1,223	-0,069	43,908	54,954
DOCK4	0,002	0,825	0,927	0,15	0,146	-0,037	749,777	111,252
IFIT3	0,001	0,826	0,927	0,17	0,167	-0,025	1520,554	256,536
PPFIA1	0,002	0,826	0,927	0,089	0,087	-0,038	1156,179	102,071
PTS	0,002	0,825	0,927	0,506	0,489	-0,05	146,696	72,959
MYL6	0,001	0,824	0,927	0,67	0,663	-0,017	1853,793	1235,953
INF2	0,001	0,825	0,927	0,206	0,21	0,024	1538,17	319,493
TRPM7	0,003	0,824	0,927	0,078	0,075	-0,054	640,113	48,76
MLST8	0,002	0,826	0,927	0,184	0,179	-0,04	548,066	99,732
GSG2	0,002	0,826	0,927	0,116	0,113	-0,041	770,869	88,138
RBCK1	0,002	0,826	0,927	0,125	0,128	0,04	747,53	94,447
PACSIN2	0,002	0,824	0,927	0,155	0,159	0,04	640,059	100,3
TRABD2A	0,002	0,827	0,928	0,224	0,23	0,037	515,263	116,945
GTF3C3	0,003	0,828	0,928	0,124	0,12	-0,051	440,801	53,877
WDR6	0,001	0,827	0,928	0,228	0,232	0,022	1582,953	363,713
SMU1	0,001	0,827	0,928	0,275	0,269	-0,035	505,017	137,302
TJP2	0,002	0,828	0,928	0,056	0,054	-0,038	1763,807	97,391
SFXN2	0,003	0,828	0,928	0,261	0,251	-0,053	222,05	56,592

RTCB	0,001	0,827	0,928	0,324	0,329	0,02	1780,653	581,493
MCCC2	0,002	0,83	0,929	0,129	0,125	-0,039	739,208	93,86
SLC37A3	0,002	0,829	0,929	0,198	0,192	-0,048	331,28	64,508
C11orf54	0,003	0,83	0,929	0,327	0,315	-0,054	168,145	53,739
PLEKHG3	0,003	0,83	0,929	0,086	0,089	0,055	496,779	43,255
C16orf13	0,002	0,83	0,929	0,419	0,431	0,041	241,657	102,505
CRNKL1	0,003	0,83	0,929	0,098	0,101	0,049	577,952	57,64
RRP1B	0,002	0,829	0,929	0,058	0,06	0,043	1214,031	71,38
RRAGC	0,002	0,832	0,93	0,233	0,24	0,04	387,204	91,77
PPP1R15B	0,001	0,831	0,93	0,153	0,156	0,031	1030,998	159,222
PHAX	0,001	0,831	0,93	0,197	0,192	-0,034	659,318	128,049
UIMC1	0,003	0,832	0,93	0,157	0,162	0,049	357,706	57,09
BCAP29	0,002	0,832	0,93	0,219	0,225	0,036	532,508	118,167
SLC7A6OS	0,002	0,831	0,93	0,516	0,534	0,049	144,552	76,075
PI4KA	0,001	0,832	0,93	0,15	0,153	0,032	946,776	143,177
DUSP12	0,002	0,835	0,931	0,385	0,395	0,038	277,011	108,032
TMEM9	0,001	0,836	0,931	0,493	0,505	0,034	298,759	148,837
MZT2B	0,002	0,834	0,931	0,257	0,25	-0,043	321,418	81,602
TMEM40	0,003	0,836	0,931	0,434	0,417	-0,059	107,124	45,716
PCCB	0,002	0,834	0,931	0,379	0,389	0,04	266,697	102,306
GRPEL1	0,001	0,836	0,931	0,497	0,489	-0,024	651,291	321,107
ADRB2	0,001	0,835	0,931	0,613	0,599	-0,034	269,734	163,488
IGFBPL1	0,002	0,835	0,931	1,704	1,643	-0,053	59,324	99,118
VPS26A	0,001	0,836	0,931	0,21	0,206	-0,029	890,984	185,004
UBASH3B	0,001	0,835	0,931	0,097	0,099	0,029	1614,955	158,084
SRPR	0,001	0,834	0,931	0,095	0,097	0,025	2647,061	254,34
H2AFJ	0,002	0,836	0,931	0,601	0,618	0,041	177,634	108,123
IRS2	0,003	0,836	0,931	0,104	0,101	-0,049	514,633	52,629
AP5M1	0,002	0,834	0,931	0,215	0,209	-0,04	418,453	88,785
FHOD1	0,001	0,835	0,931	0,192	0,197	0,032	703,059	136,783
ERBB2	0,002	0,835	0,931	0,136	0,14	0,041	579,927	79,979
SPAG9	0,001	0,834	0,931	0,108	0,106	-0,028	1740,232	186,205

SPHK1	0,003	0,836	0,931	0,212	0,221	0,056	199,904	43,263
ATP6AP2	0,001	0,835	0,931	0,577	0,588	0,027	463,933	270,378
SNAPIN	0,002	0,838	0,932	0,362	0,351	-0,042	235,489	83,971
MUT	0,003	0,838	0,932	0,222	0,23	0,053	210,297	47,741
PSMA1	0,002	0,838	0,932	0,396	0,409	0,044	194,776	78,487
NUDT22	0,002	0,837	0,932	0,483	0,499	0,048	145,337	71,315
VPS18	0,003	0,837	0,932	0,116	0,112	-0,05	441,846	50,57
HMHA1	0,002	0,838	0,932	0,106	0,103	-0,034	1026,351	107,331
CLCN4	0,003	0,837	0,932	0,153	0,158	0,052	311,66	48,31
PIP5K1A	0,001	0,84	0,933	0,125	0,128	0,028	1369,426	172,928
SNX4	0,002	0,84	0,933	0,319	0,31	-0,04	275,588	86,706
NCAPG	0,001	0,839	0,933	0,161	0,164	0,03	1006,611	163,444
NQO2	0,002	0,839	0,933	0,384	0,374	-0,04	242,872	92,039
CCAR2	0,002	0,839	0,933	0,104	0,107	0,034	1086,145	114,546
RCCD1	0,003	0,84	0,933	0,16	0,154	-0,049	341,199	53,608
PIGS	0,002	0,839	0,933	0,226	0,221	-0,035	509,356	113,84
PIP4K2B	0,003	0,839	0,933	0,071	0,068	-0,048	724,011	50,313
POLE4	0,001	0,841	0,934	0,586	0,573	-0,033	273,957	158,75
PRRC2A	0,003	0,841	0,934	0,061	0,059	-0,046	926,279	55,31
TSPAN9	0,003	0,842	0,934	0,231	0,239	0,053	194,912	45,732
RNPS1	0,002	0,841	0,934	0,136	0,14	0,04	569,185	78,744
ABHD3	0,002	0,841	0,934	0,763	0,74	-0,044	123,636	92,769
TTI1	0,001	0,841	0,934	0,21	0,206	-0,029	825,709	171,729
MRPS15	0,001	0,843	0,935	0,428	0,436	0,028	465,928	201,419
NELFA	0,001	0,843	0,935	0,355	0,363	0,033	376,785	135,44
IGFBP7	0,001	0,843	0,935	1,276	1,299	0,026	284,789	366,606
PPIL4	0,003	0,843	0,935	0,131	0,135	0,047	399,114	53,118
SMEK1	0,001	0,844	0,935	0,113	0,115	0,029	1296,262	148,124
FAM96B	0,001	0,844	0,935	1,222	1,245	0,027	256,773	316,573
SLC39A11	0,003	0,844	0,935	0,329	0,317	-0,057	127,529	41,243
RBBP9	0,002	0,843	0,935	0,163	0,159	-0,04	464,378	74,601
TRAPPC3	0,001	0,846	0,936	0,343	0,351	0,033	364,651	126,592

BARD1	0,003	0,846	0,936	0,115	0,119	0,048	422,861	49,649
EIF3B	0,001	0,846	0,936	0,319	0,322	0,014	3378,722	1081,77
TTYH3	0,002	0,846	0,936	0,077	0,075	-0,032	1590,933	121,022
VDAC3	0,001	0,846	0,936	0,459	0,469	0,031	346,533	160,874
CD81	0,001	0,846	0,936	0,945	0,935	-0,015	1241,181	1166,983
PSMD3	0,001	0,845	0,936	0,218	0,215	-0,019	1907,055	412,942
MAP2K7	0,002	0,846	0,936	0,173	0,169	-0,033	672,577	115,32
KIF1B	0,002	0,847	0,937	0,091	0,094	0,035	991,51	91,874
CSDE1	0,001	0,847	0,937	0,058	0,059	0,017	7953,589	465,025
ARV1	0,002	0,847	0,937	0,371	0,383	0,046	168,219	63,384
MRPS9	0,002	0,848	0,937	0,337	0,346	0,04	230,448	78,7
APEH	0,001	0,848	0,937	0,213	0,209	-0,026	907,068	191,043
SLC25A13	0,001	0,848	0,937	0,261	0,256	-0,031	513,306	132,672
RABEPK	0,002	0,849	0,937	0,254	0,249	-0,033	453,753	114,172
CD59	0,001	0,848	0,937	0,436	0,442	0,02	997,145	436,977
AASDHPPT	0,001	0,848	0,937	0,265	0,26	-0,027	684,622	179,464
COG3	0,002	0,847	0,937	0,228	0,222	-0,035	455,678	102,422
C16orf80	0,001	0,849	0,937	0,442	0,45	0,026	484,285	216,282
COG1	0,001	0,849	0,937	0,277	0,272	-0,028	580,6	159,47
RBBP8	0,002	0,849	0,937	0,075	0,077	0,039	904,921	69,231
SEC13	0,001	0,851	0,938	0,454	0,46	0,017	1257,395	574,778
PRIM2	0,001	0,85	0,938	0,373	0,366	-0,027	480,985	177,715
C9orf41	0,003	0,852	0,938	0,134	0,138	0,045	384,928	52,386
C10orf76	0,002	0,852	0,938	0,163	0,159	-0,042	389,099	62,597
AMOTL1	0,002	0,852	0,938	0,093	0,091	-0,031	1195,205	109,974
EPS8	0,002	0,852	0,938	0,088	0,091	0,039	754,22	67,466
SNX6	0,001	0,85	0,938	0,373	0,38	0,027	509,063	191,54
STUB1	0,002	0,851	0,938	0,554	0,57	0,041	148,852	83,535
DUS2	0,003	0,851	0,938	0,268	0,259	-0,05	179,655	47,319
STX8	0,002	0,851	0,938	0,409	0,398	-0,041	196,211	79,289
RPTOR	0,001	0,851	0,938	0,088	0,09	0,029	1538,947	137,316
AFG3L2	0,002	0,85	0,938	0,16	0,157	-0,033	646,202	102,408

REXO1	0,002	0,851	0,938	0,071	0,073	0,04	898,977	65,104
PAFAH1B3	0,001	0,851	0,938	0,672	0,685	0,027	313,126	212,545
ROMO1	0,001	0,85	0,938	2,646	2,695	0,027	183,506	489,396
SDPR	0,003	0,854	0,939	0,152	0,157	0,046	328,251	50,496
TUBA4A	0,001	0,855	0,939	0,418	0,412	-0,019	991,134	411,791
ZNF131	0,003	0,854	0,939	0,096	0,099	0,042	566,322	55,103
EEF1E1	0,002	0,854	0,939	0,546	0,562	0,041	143,578	79,59
FDFT1	0,001	0,855	0,939	0,429	0,437	0,028	404,445	175,002
PTK2	0,001	0,855	0,939	0,107	0,109	0,021	2263,48	245,108
IFITM2	0,001	0,853	0,939	0,459	0,453	-0,02	813,209	371,086
CELF1	0,001	0,855	0,939	0,145	0,143	-0,021	1930,771	277,929
ESPL1	0,002	0,854	0,939	0,093	0,091	-0,032	1050,961	96,707
VPS37B	0,002	0,854	0,939	0,208	0,203	-0,035	449,569	92,308
ADPGK	0,002	0,855	0,939	0,225	0,22	-0,031	516,274	114,962
CENPT	0,003	0,855	0,939	0,128	0,124	-0,043	439,256	55,234
NTN1	0,003	0,854	0,939	0,169	0,164	-0,045	314,734	52,403
ENSG00000126005.11	0,003	0,855	0,939	0,18	0,185	0,046	269,418	49,152
TEX264	0,001	0,857	0,94	0,471	0,463	-0,026	418,541	195,65
SLMAP	0,002	0,857	0,94	0,097	0,095	-0,032	1025,572	98,145
CPSF4	0,002	0,857	0,94	0,229	0,235	0,041	275,852	63,938
ENY2	0,001	0,856	0,94	0,826	0,842	0,028	252,949	211,075
ATG4D	0,003	0,857	0,94	0,191	0,186	-0,042	318,437	60,064
ADSL	0,001	0,857	0,94	0,519	0,525	0,016	1228,859	641,142
VAMP3	0,001	0,859	0,941	0,218	0,214	-0,026	734,247	158,86
FAM118B	0,003	0,859	0,941	0,272	0,281	0,043	204,166	56,518
C12orf23	0,002	0,858	0,941	0,123	0,12	-0,034	677,025	81,957
DYRK1A	0,003	0,858	0,941	0,12	0,116	-0,044	404,643	47,855
DYNC1LI1	0,001	0,86	0,942	0,415	0,41	-0,02	825,82	340,615
GOT2	0,001	0,86	0,942	0,258	0,255	-0,015	2324,742	595,838
TOB2	0,002	0,86	0,942	0,077	0,079	0,036	899,348	70,539
TMEM173	0,002	0,861	0,943	0,304	0,297	-0,034	301,049	90,518
GRHPR	0,001	0,861	0,943	0,404	0,411	0,023	596,445	242,732

EHD4	0,001	0,861	0,943	0,227	0,224	-0,02	1290,175	290,355
SOCS3	0,002	0,861	0,943	0,157	0,162	0,039	387,127	61,785
DOT1L	0,002	0,861	0,943	0,051	0,05	-0,034	1547,589	78,708
ANO10	0,001	0,864	0,945	0,283	0,278	-0,028	474,562	133,147
BBS7	0,003	0,865	0,945	0,315	0,324	0,045	152,748	48,737
C5orf51	0,002	0,866	0,945	0,079	0,08	0,035	834,262	66,393
SMAD5	0,002	0,866	0,945	0,129	0,131	0,027	912,507	118,593
ENSG00000203875.6	0,001	0,864	0,945	0,965	0,981	0,024	286,018	278,367
OSBPL3	0,002	0,866	0,945	0,071	0,069	-0,035	976,664	68,46
WHSC1L1	0,003	0,865	0,945	0,057	0,055	-0,041	879,459	49,185
XPA	0,003	0,865	0,945	0,352	0,363	0,045	140,937	50,434
NELFB	0,001	0,864	0,945	0,255	0,251	-0,021	908,503	229,68
FAM204A	0,002	0,865	0,945	0,214	0,209	-0,037	329,347	69,527
MARK2	0,002	0,864	0,945	0,105	0,103	-0,033	821,045	85,27
CPT1A	0,002	0,863	0,945	0,182	0,178	-0,031	570,495	102,744
BCAT1	0,001	0,866	0,945	0,129	0,128	-0,019	2099,235	269,564
MED1	0,001	0,864	0,945	0,099	0,101	0,024	1636,661	163,794
ZMAT5	0,003	0,865	0,945	1,312	1,265	-0,053	44,852	57,846
POLD3	0,002	0,867	0,946	0,153	0,156	0,028	751,306	115,913
SRSF6	0,001	0,868	0,946	0,119	0,12	0,02	1977,373	236,441
CSNK1E	0,001	0,868	0,946	0,114	0,116	0,024	1349,472	155,101
PRPS1	0,001	0,867	0,946	0,281	0,284	0,018	1282,735	362,239
SLC19A2	0,003	0,869	0,947	0,325	0,316	-0,041	169,473	54,281
HRH1	0,001	0,87	0,947	0,218	0,222	0,025	663,822	145,996
NR2C2	0,002	0,868	0,947	0,224	0,219	-0,033	376,377	83,32
MRPL1	0,002	0,87	0,947	0,415	0,404	-0,038	167,633	68,663
OSTC	0,001	0,869	0,947	1,089	1,112	0,031	135,454	149,286
MFSD8	0,003	0,87	0,947	0,427	0,414	-0,043	125,621	52,619
DDX31	0,003	0,869	0,947	0,131	0,134	0,039	394,258	52,205
SEC23IP	0,001	0,869	0,947	0,17	0,173	0,022	1102,612	189,252
ADIPOR2	0,001	0,868	0,947	0,141	0,138	-0,023	1280,126	178,587
AAGAB	0,002	0,87	0,947	0,262	0,257	-0,03	382,755	99,306

ARFGAP1	0,002	0,869	0,947	0,12	0,117	-0,027	935,558	110,835
RMND5B	0,003	0,871	0,948	0,182	0,177	-0,041	274,193	49,147
ORC3	0,002	0,872	0,948	0,219	0,224	0,032	372,707	82,763
POP4	0,002	0,871	0,948	0,316	0,322	0,028	357,066	113,95
PROCR	0,001	0,872	0,948	0,76	0,769	0,017	649,18	495,118
RTFDC1	0,001	0,871	0,948	0,293	0,289	-0,022	704,247	205,02
IMMT	0,001	0,872	0,949	0,175	0,173	-0,017	1745,401	304,412
IER5L	0,002	0,873	0,949	0,687	0,67	-0,036	121,337	82,165
STXBP2	0,002	0,873	0,949	0,266	0,259	-0,038	238,016	62,571
ABCD3	0,001	0,875	0,95	0,313	0,317	0,018	948,185	298,537
TNKS2	0,002	0,874	0,95	0,103	0,101	-0,027	1054,758	107,585
PAM	0,001	0,876	0,951	0,188	0,185	-0,023	918,142	171,174
LACTB2	0,002	0,876	0,951	0,469	0,459	-0,03	219,042	101,54
SUPT16H	0,001	0,875	0,951	0,057	0,056	-0,022	2591,738	147,524
TUBG1	0,001	0,876	0,951	0,282	0,278	-0,018	962,545	269,549
PANK2	0,002	0,876	0,951	0,182	0,186	0,031	448,537	82,413
FAM118A	0,003	0,876	0,951	0,156	0,161	0,039	315,572	50,075
SELO	0,001	0,876	0,951	0,208	0,212	0,024	709,443	148,756
CCNL1	0,003	0,878	0,952	0,12	0,123	0,036	451,3	54,731
ERLIN1	0,001	0,878	0,952	0,223	0,22	-0,02	979,632	216,57
LMNB2	0,001	0,878	0,952	0,082	0,083	0,015	5176,042	425,581
FHL1	0,002	0,878	0,952	0,236	0,241	0,028	426,345	101,812
HECTD3	0,002	0,879	0,953	0,124	0,126	0,025	962,314	120,02
VPS72	0,002	0,879	0,953	0,169	0,172	0,028	539,176	91,828
ABCC10	0,003	0,879	0,953	0,243	0,25	0,036	233,65	57,548
OAF	0,002	0,879	0,953	0,188	0,192	0,026	586,949	111,321
CARHSP1	0,002	0,878	0,953	0,422	0,413	-0,032	195,606	81,558
FOXK2	0,002	0,88	0,953	0,078	0,08	0,027	1226,422	96,772
HSPBP1	0,001	0,879	0,953	0,581	0,573	-0,02	416,396	240,257
CMTM6	0,001	0,881	0,954	0,219	0,216	-0,018	1151,453	250,045
FAH	0,001	0,881	0,954	0,412	0,405	-0,025	339,043	138,728
SUN2	0,002	0,882	0,954	0,254	0,259	0,026	448,044	114,999

UCP2	0,001	0,884	0,956	0,272	0,269	-0,016	1300,582	350,849
LMO4	0,002	0,886	0,957	0,211	0,215	0,03	358,587	76,301
LAMTOR2	0,002	0,885	0,957	0,926	0,908	-0,028	137,871	126,425
GTF2E1	0,003	0,885	0,957	0,199	0,194	-0,037	244,443	48,044
GFM1	0,002	0,886	0,957	0,158	0,155	-0,027	581,354	90,756
OTUD4	0,003	0,886	0,957	0,085	0,087	0,033	640,257	54,996
MTHFD1L	0,001	0,885	0,957	0,177	0,174	-0,023	764,545	134,236
DAGLB	0,001	0,885	0,957	0,307	0,312	0,024	426,775	132,122
COPS6	0,001	0,884	0,957	0,575	0,58	0,014	1075,757	621,006
POLR1E	0,001	0,886	0,957	0,234	0,231	-0,02	818,861	190,224
DENR	0,001	0,885	0,957	0,168	0,171	0,023	752,521	127,464
PRMT7	0,002	0,885	0,957	0,165	0,162	-0,027	567,455	92,993
ARHGAP35	0,003	0,886	0,957	0,028	0,029	0,032	1828,254	52,68
LRRC47	0,002	0,888	0,958	0,302	0,307	0,024	401,075	121,897
USP48	0,002	0,889	0,958	0,139	0,142	0,024	702,839	98,897
KIRREL	0,001	0,889	0,958	0,098	0,1	0,021	1348,544	133,709
UCK2	0,001	0,888	0,958	0,233	0,236	0,015	1310,432	306,996
FASTKD2	0,002	0,888	0,958	0,206	0,209	0,024	550,563	114,357
SLC36A1	0,003	0,889	0,958	0,091	0,089	-0,032	633,912	56,824
AIFM2	0,001	0,887	0,958	0,311	0,315	0,022	477,544	149,603
SP1	0,001	0,888	0,958	0,196	0,198	0,016	1400,701	275,977
CTDSP2	0,002	0,888	0,958	0,083	0,085	0,03	769,81	64,686
ENSG00000249550.2	0,004	0,887	0,958	0,174	0,169	-0,041	214,59	37,011
FBRSL1	0,003	0,887	0,958	0,111	0,114	0,037	394,297	44,261
CD276	0,003	0,887	0,958	0,105	0,107	0,036	440,655	46,653
PIGU	0,001	0,889	0,958	0,707	0,697	-0,02	315,064	221,165
SLC17A9	0,001	0,888	0,958	0,208	0,211	0,02	823,506	172,567
SPCS3	0,001	0,891	0,959	0,259	0,262	0,017	930,491	242,216
MAF1	0,001	0,891	0,959	0,131	0,13	-0,019	1305,319	170,128
SPTLC1	0,001	0,892	0,959	0,344	0,339	-0,019	590,347	201,556
PRMT5	0,001	0,892	0,959	0,222	0,22	-0,016	1257,197	278,029
NOB1	0,001	0,891	0,959	0,366	0,372	0,02	498,737	184,112

MAP2K4	0,002	0,891	0,959	0,098	0,096	-0,03	647,626	62,989
COPRS	0,002	0,891	0,959	0,452	0,443	-0,029	180,472	80,758
MGME1	0,002	0,892	0,959	0,189	0,193	0,027	434,434	83,07
TIMP3	0,002	0,892	0,959	0,294	0,299	0,024	386,271	114,676
STMN1	0,001	0,897	0,96	0,526	0,523	-0,009	2580,919	1353,56
CRTC2	0,002	0,894	0,96	0,154	0,151	-0,023	728,176	111,238
SDE2	0,002	0,896	0,96	0,247	0,243	-0,024	420,294	103,068
ATG7	0,002	0,893	0,96	0,248	0,253	0,027	326,206	81,614
MANF	0,003	0,897	0,96	0,612	0,597	-0,035	88,675	53,571
HSD17B4	0,002	0,893	0,96	0,16	0,158	-0,023	701,367	111,378
TCOF1	0,001	0,895	0,96	0,089	0,089	0,015	3194,401	284,533
ZFAND3	0,001	0,895	0,96	0,17	0,168	-0,019	959,556	161,863
NT5C3A	0,002	0,895	0,96	0,347	0,354	0,031	183,657	64,47
PSPH	0,002	0,894	0,96	0,465	0,475	0,031	147,644	69,388
MTFR1	0,002	0,896	0,96	0,189	0,192	0,024	489,382	93,441
RBM17	0,002	0,895	0,96	0,144	0,142	-0,023	720,085	102,939
KIAA1279	0,001	0,895	0,96	0,216	0,214	-0,018	916,77	197,183
TSG101	0,001	0,896	0,96	0,367	0,361	-0,02	489,557	178,537
PRMT3	0,002	0,895	0,96	0,231	0,228	-0,022	547,952	125,915
BORA	0,002	0,892	0,96	0,252	0,247	-0,024	429,289	107,081
ARHGEF7	0,002	0,892	0,96	0,078	0,079	0,028	903,879	70,767
LGALS3	0,001	0,894	0,96	0,581	0,589	0,018	444,105	259,471
ZFP36L1	0,001	0,893	0,96	0,191	0,189	-0,015	1664,455	316,054
TCF12	0,002	0,893	0,96	0,11	0,112	0,024	810,319	90,312
FOPNL	0,002	0,897	0,96	0,188	0,191	0,022	606,482	115,04
SCARF1	0,002	0,893	0,96	0,369	0,362	-0,028	274,297	100,715
CYB5D2	0,002	0,895	0,96	0,377	0,369	-0,031	170,109	63,419
SLC9A3R1	0,001	0,895	0,96	0,177	0,179	0,018	1064,438	189,188
TRIM65	0,002	0,896	0,96	0,172	0,175	0,024	538,26	93,534
C19orf10	0,001	0,895	0,96	0,494	0,499	0,013	912,643	453,034
ITCH	0,001	0,895	0,96	0,136	0,137	0,018	1318,711	179,987
CDK16	0,003	0,893	0,96	0,038	0,039	0,035	1134,11	43,205

KIAA0226	0,003	0,899	0,962	0,103	0,1	-0,032	445,146	45,231
CD320	0,001	0,899	0,962	0,609	0,604	-0,014	709,694	430,654
SSU72	0,001	0,902	0,963	0,164	0,162	-0,019	830,47	135,593
WNT5A	0,003	0,9	0,963	0,207	0,211	0,031	253,996	53,011
TRIM23	0,003	0,901	0,963	0,305	0,312	0,033	157,422	48,628
SQSTM1	0,001	0,901	0,963	0,344	0,342	-0,011	1700,092	583,013
PPP2R2A	0,001	0,901	0,963	0,24	0,237	-0,017	789,387	188,309
UQCRB	0,001	0,901	0,963	1,48	1,462	-0,017	225,126	331,004
ADK	0,001	0,902	0,963	0,571	0,566	-0,014	628,662	357,39
MRE11A	0,003	0,9	0,963	0,168	0,165	-0,029	343,646	57,074
CMAS	0,001	0,9	0,963	0,294	0,29	-0,021	434,383	126,801
PSMD8	0,001	0,901	0,963	0,529	0,533	0,01	1557,183	826,583
SNX5	0,001	0,902	0,963	0,219	0,217	-0,017	835,646	182,185
TSPO	0,001	0,9	0,963	1,084	1,074	-0,014	491,405	530,609
LRP8	0,002	0,904	0,964	0,114	0,116	0,026	639,886	73,391
ROR1	0,002	0,905	0,964	0,229	0,225	-0,026	301,62	68,449
JAK1	0,001	0,903	0,964	0,074	0,074	0,018	1859,722	137,556
HIAT1	0,001	0,905	0,964	0,255	0,252	-0,017	683,498	173,239
SGCE	0,001	0,904	0,964	0,36	0,355	-0,02	363,632	130,055
ZNHIT1	0,001	0,905	0,964	0,627	0,618	-0,021	223,65	139,14
CENPN	0,001	0,904	0,964	0,273	0,269	-0,019	494,461	133,907
CDC6	0,001	0,904	0,964	0,106	0,107	0,018	1235,697	131,478
OAZ1	0,001	0,903	0,964	0,062	0,063	0,015	3302,213	205,679
TOMM34	0,001	0,904	0,964	0,191	0,189	-0,015	1285,182	244,154
EWSR1	0,001	0,903	0,964	0,113	0,114	0,013	2647,291	300,082
ALG12	0,001	0,904	0,964	0,421	0,426	0,018	445,67	188,709
WDR77	0,001	0,907	0,965	0,617	0,611	-0,014	590,298	362,728
SNX8	0,002	0,907	0,965	0,177	0,18	0,022	496,487	88,559
LYN	0,002	0,906	0,965	0,142	0,14	-0,022	642,124	90,555
CAAP1	0,003	0,907	0,965	0,216	0,221	0,031	204,511	44,634
GNE	0,002	0,906	0,965	0,121	0,119	-0,025	566,028	68,183
PPP6C	0,001	0,906	0,965	0,362	0,365	0,013	914,395	332,568

PRRC2B	0,001	0,907	0,965	0,044	0,044		0,016	3828,956	169,291
SETX	0,002	0,908	0,965	0,058	0,057		-0,021	1607,468	92,651
PACS1	0,002	0,908	0,965	0,089	0,088		-0,02	1171,797	103,712
PHB2	0,001	0,906	0,965	0,401	0,398		-0,014	670,125	267,815
PLEKHA5	0,002	0,906	0,965	0,091	0,093		0,023	864,599	79,557
SLC16A7	0,002	0,906	0,965	0,34	0,346		0,021	315,833	108,366
SLAIN1	0,003	0,909	0,965	0,209	0,205		-0,03	231,883	48,081
GCH1	0,003	0,909	0,965	0,26	0,266		0,031	165,647	43,493
ZC3H7A	0,002	0,907	0,965	0,106	0,108		0,024	676,197	72,273
GADD45GIP1	0,001	0,908	0,965	0,933	0,941		0,012	660,707	618,832
SUGP1	0,003	0,907	0,965	0,127	0,124		-0,028	431,514	54,144
USF2	0,002	0,905	0,965	0,095	0,093		-0,026	653,047	61,558
NUP62	0,001	0,907	0,965	0,217	0,215		-0,013	1403,99	302,679
ABCB7	0,001	0,908	0,965	0,57	0,577		0,019	281,575	161,299
LAPTM5	0,001	0,912	0,966	0,376	0,373		-0,011	1246,45	467,455
OCIAD1	0,001	0,912	0,966	0,437	0,441		0,013	661,099	290,088
TPBG	0,002	0,911	0,966	0,298	0,302		0,021	342,94	102,817
MAP3K4	0,003	0,912	0,966	0,111	0,113		0,027	420,245	47,172
SLC4A2	0,001	0,912	0,966	0,141	0,14		-0,014	1600,079	224,583
MTERFD1	0,003	0,911	0,966	0,416	0,424		0,028	141,686	59,633
BNIP3	0,001	0,912	0,966	0,309	0,313		0,017	490,409	152,556
ELMSAN1	0,002	0,911	0,966	0,078	0,077		-0,023	892,182	69,146
GPATCH2L	0,002	0,913	0,966	0,064	0,063		-0,024	931,785	59,109
SMG1	0,002	0,912	0,966	0,062	0,063		0,018	1790,964	111,78
UBE2G1	0,001	0,912	0,966	0,18	0,178		-0,015	1019,541	182,522
ENSG00000266402.2	0,001	0,91	0,966	51,999	49,792		-0,063	6,998	356,247
SLC16A3	0,001	0,912	0,966	0,829	0,833		0,007	2337,358	1941,881
PPP4R1	0,001	0,91	0,966	0,195	0,193		-0,013	1309,072	254,218
MPV17L2	0,002	0,91	0,966	0,338	0,342		0,02	338,07	114,906
ATP1B1	0,001	0,914	0,967	0,52	0,525		0,015	434,412	226,853
SET	0,001	0,913	0,967	0,157	0,158		0,011	2446,672	385,294
CTNND1	0,001	0,913	0,967	0,07	0,069		-0,016	2113,955	147,175

ERC1	0,003	0,913	0,967	0,036	0,036	0,027	1300,726	46,602
EIF2B2	0,001	0,914	0,967	0,294	0,297	0,015	690,676	204,23
PPP1R12C	0,002	0,914	0,967	0,082	0,08	-0,024	709,556	57,542
DNPEP	0,002	0,916	0,968	0,356	0,351	-0,019	327,868	115,935
DVL3	0,002	0,917	0,968	0,075	0,076	0,022	858,434	64,618
BOD1L1	0,001	0,916	0,968	0,105	0,104	-0,016	1328,771	138,329
KIF20A	0,001	0,917	0,968	0,217	0,218	0,01	2184,958	474,978
FAM126A	0,002	0,915	0,968	0,116	0,114	-0,022	640,2	73,444
DNAJB12	0,001	0,915	0,968	0,195	0,193	-0,018	656,59	127,43
NPAT	0,002	0,916	0,968	0,11	0,112	0,021	712,863	79,08
DLAT	0,003	0,917	0,968	0,292	0,287	-0,024	211,097	61,073
DHX38	0,002	0,916	0,968	0,064	0,065	0,021	1079,578	70,189
PFAS	0,001	0,916	0,968	0,128	0,129	0,012	2252,97	288,642
IL13RA1	0,001	0,915	0,968	0,15	0,152	0,017	844,15	127,64
TRNAU1AP	0,002	0,918	0,969	0,419	0,426	0,024	155,226	65,478
C9orf3	0,003	0,919	0,969	0,134	0,132	-0,026	351,393	46,676
FRMD4A	0,004	0,918	0,969	0,108	0,106	-0,03	348,975	37,417
KDELC2	0,001	0,919	0,969	0,162	0,16	-0,014	1163,692	187,45
TRMT61A	0,002	0,918	0,969	0,215	0,218	0,017	550,288	119,292
TCEB2	0,001	0,919	0,969	1,954	1,971	0,012	275,664	540,811
ERGIC3	0,001	0,919	0,969	0,588	0,592	0,008	1696,338	1000,688
NDUFV3	0,001	0,919	0,969	0,457	0,451	-0,017	301,979	137,153
PHC2	0,001	0,923	0,97	0,16	0,158	-0,013	1131,627	179,959
USP1	0,001	0,922	0,97	0,171	0,17	-0,01	2151,943	366,79
C1orf198	0,002	0,925	0,97	0,123	0,125	0,019	627,117	77,983
SH3BP5L	0,002	0,927	0,97	0,103	0,101	-0,02	627,83	64,153
HTRA2	0,002	0,929	0,97	0,502	0,508	0,017	238,527	120,393
IMP4	0,001	0,928	0,97	0,221	0,219	-0,012	868,083	191,181
OLA1	0,001	0,924	0,97	0,212	0,21	-0,013	1001,66	211,676
KIAA1715	0,001	0,92	0,97	0,174	0,172	-0,015	907,379	157,219
GMPPA	0,002	0,925	0,97	0,27	0,273	0,016	441,404	119,61
NPRL2	0,003	0,923	0,97	0,364	0,371	0,026	128,54	47,235

RPL24	0,001	0,922	0,97	1,288	1,278	-0,011	349,511	448,645
PVRL3	0,002	0,923	0,97	0,291	0,295	0,018	329,557	96,724
LAP3	0,002	0,92	0,97	0,233	0,236	0,018	411,016	96,325
SGCB	0,001	0,924	0,97	0,254	0,257	0,015	526,528	134,423
SPATA5	0,003	0,924	0,97	0,138	0,141	0,023	375,23	52,429
WDR70	0,003	0,923	0,97	0,288	0,283	-0,024	197,991	56,531
MRPS27	0,001	0,929	0,97	0,286	0,288	0,01	1132,853	325,433
RIOK2	0,003	0,927	0,97	0,147	0,145	-0,022	391,294	57,122
KCNQ5	0,003	0,929	0,97	0,099	0,097	-0,021	558,225	54,694
PGM3	0,002	0,925	0,97	0,239	0,236	-0,016	512,682	121,643
SGK1	0,002	0,921	0,97	0,305	0,301	-0,021	247,989	75,128
CITED2	0,001	0,923	0,97	0,347	0,35	0,01	1296,334	451,647
ENSG00000253504.1	0,003	0,927	0,97	0,447	0,439	-0,024	124,22	55,083
VPS13B	0,002	0,921	0,97	0,09	0,091	0,023	648,844	58,756
ALAD	0,003	0,922	0,97	0,123	0,125	0,027	344,156	42,665
TRIM32	0,003	0,924	0,97	0,134	0,132	-0,023	403,89	53,659
SDCCAG3	0,002	0,928	0,97	0,094	0,093	-0,018	740,21	68,93
SUPV3L1	0,002	0,929	0,97	0,21	0,208	-0,015	574,558	119,861
MARCH5	0,001	0,923	0,97	0,299	0,295	-0,016	452,828	134,519
TNKS1BP1	0,003	0,922	0,97	0,062	0,061	-0,023	861,497	53,092
RPS6KA4	0,001	0,924	0,97	0,153	0,155	0,014	990,082	152,433
THYN1	0,002	0,927	0,97	0,484	0,49	0,017	238,638	116,203
EFCAB4B	0,003	0,922	0,97	0,121	0,119	-0,023	466,121	56,16
NOP2	0,002	0,929	0,97	0,061	0,062	0,014	1871,586	115,513
KMT2D	0,001	0,927	0,97	0,147	0,148	0,011	1791,664	264,162
DAZAP2	0,001	0,921	0,97	0,371	0,374	0,011	1075,576	400,621
UTP20	0,001	0,928	0,97	0,076	0,077	0,013	1976,585	151,536
VPS29	0,001	0,929	0,97	0,629	0,624	-0,012	382,724	239,623
PSMD9	0,003	0,921	0,97	0,413	0,406	-0,025	140,021	57,399
EBPL	0,002	0,928	0,97	0,839	0,825	-0,023	80,613	67,051
CDKN3	0,001	0,926	0,97	0,336	0,338	0,011	845,221	285,029
IFI27L2	0,002	0,923	0,97	1,513	1,539	0,024	65,426	99,65

RRN3	0,003	0,922	0,97	0,173	0,176	0,024	293,229	51,066
ATP6V0D1	0,001	0,927	0,97	0,543	0,547	0,01	760,271	414,596
SCO1	0,001	0,922	0,97	0,364	0,367	0,013	616,266	225,407
ICT1	0,001	0,929	0,97	0,71	0,716	0,013	283,591	202,244
TWSG1	0,001	0,92	0,97	0,227	0,225	-0,013	1012,156	229,079
HMG20B	0,001	0,927	0,97	0,158	0,159	0,013	1083,689	171,529
ENSG00000034063.9	0,001	0,926	0,97	0,152	0,151	-0,012	1346,596	204,041
AP1M1	0,001	0,923	0,97	0,219	0,218	-0,011	1489,778	325,32
CEBPG	0,003	0,926	0,97	0,11	0,108	-0,022	486,227	52,983
CIC	0,002	0,924	0,97	0,076	0,075	-0,019	1058,588	79,698
SEC23B	0,001	0,929	0,97	0,225	0,223	-0,011	1206,178	269,947
NECAB3	0,003	0,929	0,97	0,296	0,301	0,022	193,352	57,724
ADRM1	0,001	0,926	0,97	0,402	0,404	0,008	1493,153	601,31
IL2RB	0,003	0,929	0,97	0,089	0,087	-0,033	460,653	40,887
CXorf56	0,003	0,926	0,97	0,214	0,21	-0,023	250,257	52,987
ORMDL1	0,001	0,933	0,971	0,313	0,316	0,013	438,117	137,731
TRIP12	0,001	0,931	0,971	0,046	0,046	-0,011	4435,664	204,158
GLB1	0,001	0,931	0,971	0,395	0,392	-0,011	604,17	237,726
CHMP2B	0,002	0,932	0,971	0,239	0,242	0,017	374,272	89,942
ZNF148	0,003	0,93	0,971	0,103	0,101	-0,021	513,99	52,383
CDC42SE2	0,003	0,931	0,971	0,208	0,205	-0,021	264,88	54,658
FNIP1	0,003	0,931	0,971	0,108	0,107	-0,02	501,984	53,863
MASTL	0,002	0,93	0,971	0,207	0,205	-0,015	542,864	111,628
CAMK2G	0,002	0,93	0,971	0,168	0,166	-0,017	509,598	85,392
BAG3	0,001	0,931	0,971	0,172	0,173	0,009	2364,4	406,914
EMC4	0,001	0,93	0,971	0,505	0,509	0,011	537,964	272,626
GEMIN4	0,001	0,931	0,971	0,266	0,268	0,007	2247,965	600,149
ERCC1	0,001	0,931	0,971	0,303	0,301	-0,012	639,888	193,439
ZMYND8	0,001	0,933	0,971	0,075	0,075	-0,013	1711,01	128,218
UBA1	0,001	0,932	0,971	0,22	0,218	-0,014	555,461	121,613
TARDBP	0,001	0,934	0,972	0,152	0,153	0,011	1121,138	170,921
STXBP3	0,003	0,934	0,972	0,185	0,182	-0,021	255,489	46,951

RPS27	0,001	0,935	0,972	26,464	26,992	0,029	17,784	474,302
SP4	0,003	0,933	0,972	0,327	0,332	0,021	164,944	54,382
PLAA	0,001	0,933	0,972	0,231	0,233	0,013	566,607	131,572
ARF6	0,001	0,935	0,972	0,305	0,303	-0,009	1161,33	352,798
C17orf62	0,002	0,934	0,972	0,234	0,231	-0,016	355,376	82,649
ENOSF1	0,003	0,933	0,972	0,13	0,132	0,021	381,893	49,954
RNF25	0,002	0,935	0,973	0,346	0,349	0,014	352,174	122,388
CETN3	0,002	0,936	0,973	0,461	0,466	0,017	197,719	91,657
RPS6KA1	0,001	0,94	0,974	0,215	0,216	0,012	609,913	131,493
FHL3	0,002	0,94	0,974	0,239	0,241	0,016	295,948	70,976
ANKRD13C	0,002	0,941	0,974	0,127	0,126	-0,013	763,238	96,49
LBR	0,001	0,941	0,974	0,281	0,279	-0,007	1765,998	494,745
ADCY3	0,002	0,941	0,974	0,127	0,128	0,017	466,398	59,323
UBR3	0,002	0,939	0,974	0,147	0,149	0,014	662,038	97,946
ARL8B	0,002	0,94	0,974	0,166	0,167	0,012	693,871	115,432
MAPKAPK3	0,002	0,938	0,974	0,114	0,116	0,014	843,858	96,914
FOXP1	0,001	0,941	0,974	0,167	0,166	-0,011	965,423	161,005
POLR2H	0,001	0,939	0,974	0,546	0,541	-0,012	322,277	175,13
LIMCH1	0,001	0,941	0,974	0,059	0,059	-0,013	1914,07	113,259
NUP54	0,001	0,939	0,974	0,324	0,326	0,011	599,284	194,89
LARS	0,001	0,94	0,974	0,115	0,115	-0,009	2283,497	262,9
CALD1	0,002	0,938	0,974	0,098	0,099	0,014	939,383	92,182
ASAH1	0,002	0,941	0,974	0,575	0,582	0,017	132,7	76,731
WDR5	0,001	0,937	0,974	0,143	0,144	0,012	1072,139	154,211
ABCA2	0,003	0,941	0,974	0,09	0,089	-0,021	434,633	39,112
CUEDC2	0,002	0,941	0,974	0,295	0,292	-0,015	309,196	90,783
FAM160B1	0,002	0,939	0,974	0,187	0,184	-0,017	349,234	64,781
CDCA5	0,001	0,939	0,974	0,11	0,11	0,01	1620,485	178,166
DIAPH3	0,001	0,94	0,974	0,075	0,075	0,01	2152,821	161,381
ACTR10	0,001	0,938	0,974	0,427	0,43	0,011	469,343	201,078
LUC7L	0,001	0,937	0,974	0,413	0,416	0,011	483,506	200,524
DNAJA2	0,001	0,939	0,974	0,289	0,287	-0,009	976,281	281,315

RANBP10	0,003	0,94	0,974	0,128	0,126	-0,018	409,085	51,983
SAT2	0,002	0,938	0,974	0,313	0,316	0,017	238,914	75,217
TRIP10	0,001	0,941	0,974	0,191	0,19	-0,008	1377,998	262,839
ERF	0,002	0,941	0,974	0,11	0,109	-0,016	559,287	61,382
CDC7	0,002	0,942	0,975	0,156	0,155	-0,014	554,694	86,12
NOL10	0,002	0,942	0,975	0,234	0,232	-0,013	461,222	107,432
NDUFAF4	0,002	0,943	0,975	0,712	0,719	0,014	154,173	110,424
FBXO9	0,002	0,944	0,976	0,122	0,123	0,014	646,939	79,355
CTBP2	0,002	0,944	0,976	0,236	0,234	-0,014	342,084	80,425
NOC4L	0,001	0,943	0,976	0,392	0,395	0,012	328,183	129,051
SPAG5	0,001	0,944	0,976	0,232	0,231	-0,008	1416,208	327,711
UBE4B	0,002	0,946	0,977	0,132	0,133	0,011	825,152	109,373
GPD2	0,001	0,947	0,977	0,102	0,101	-0,01	1296,073	131,414
SLC35B1	0,001	0,946	0,977	0,402	0,405	0,009	543,674	219,358
MAP2K2	0,001	0,946	0,977	0,263	0,262	-0,008	1241,205	325,87
HNRNPM	0,001	0,947	0,977	0,199	0,2	0,005	4119,026	822,703
ELL	0,003	0,946	0,977	0,142	0,14	-0,016	358,691	50,657
DNTTIP1	0,001	0,946	0,977	0,47	0,467	-0,01	444,095	208,123
COMMD1	0,002	0,949	0,978	0,873	0,865	-0,013	127,976	111,246
AOX1	0,002	0,949	0,978	0,152	0,15	-0,014	610,485	92,39
POMGNT2	0,003	0,948	0,978	0,158	0,16	0,016	299,732	47,579
COPG1	0,001	0,948	0,978	0,215	0,216	0,007	1835,969	395,846
RINT1	0,002	0,947	0,978	0,327	0,331	0,015	213,939	70,473
CCNE2	0,002	0,949	0,978	0,371	0,375	0,015	181,342	67,669
ABHD17B	0,003	0,948	0,978	0,279	0,283	0,017	187,903	52,837
ZEB1	0,002	0,949	0,978	0,09	0,091	0,011	1061,158	95,813
GNAS	0,001	0,947	0,978	0,323	0,324	0,005	6047,478	1953,537
MAFF	0,002	0,948	0,978	0,164	0,166	0,013	488,328	80,503
FGD1	0,003	0,948	0,978	0,108	0,107	-0,017	430,183	46,167
KIAA0319L	0,003	0,951	0,979	0,093	0,094	0,016	495,297	46,111
TACSTD2	0,002	0,95	0,979	0,557	0,552	-0,014	153,535	85,241
SPRED2	0,002	0,95	0,979	0,055	0,056	0,014	1109,806	61,431

CASP3	0,002	0,951	0,979	0,283	0,28	-0,012	367,071	103,21
SLC35B2	0,001	0,952	0,979	0,361	0,358	-0,009	478,332	172,066
NAT10	0,002	0,952	0,979	0,084	0,083	-0,01	1458,822	121,935
STIP1	0,001	0,951	0,979	0,233	0,232	-0,005	3967,359	923,4
MAPK1IP1L	0,001	0,952	0,979	0,316	0,318	0,006	1694,638	537,062
KPNA2	0,002	0,952	0,979	0,174	0,175	0,012	476,035	83,061
CARM1	0,001	0,951	0,979	0,247	0,248	0,007	1225,511	303,766
SIPA1L3	0,001	0,952	0,979	0,126	0,125	-0,008	1560,794	196,115
NOSIP	0,001	0,951	0,979	0,496	0,499	0,008	480,42	238,823
ENSG00000232388.2	0,001	0,953	0,979	1,311	1,322	0,012	102,195	134,504
EIF1AX	0,003	0,95	0,979	0,188	0,19	0,015	275,958	52,125
BIN1	0,003	0,954	0,98	0,192	0,19	-0,015	250,879	47,948
C5orf22	0,002	0,954	0,98	0,144	0,145	0,011	611,462	88,479
NDUFB1	0,002	0,954	0,98	0,738	0,732	-0,012	141,563	103,986
NFYC	0,001	0,955	0,981	0,29	0,289	-0,006	991,762	287,32
SH3D19	0,003	0,957	0,981	0,105	0,106	0,015	390,569	41,339
HDAC9	0,003	0,955	0,981	0,18	0,181	0,014	268,904	48,513
C9orf40	0,002	0,957	0,981	0,407	0,404	-0,01	269,741	109,292
TTC9C	0,002	0,957	0,981	0,393	0,39	-0,011	226,894	88,802
HYLS1	0,001	0,956	0,981	0,334	0,331	-0,01	390,316	129,761
ORMDL2	0,003	0,956	0,981	0,789	0,798	0,015	80,076	63,493
CHMP1A	0,001	0,957	0,981	0,225	0,226	0,008	866,778	195,423
KIF1C	0,001	0,957	0,981	0,029	0,029	0,008	4928,896	141,405
PSENEN	0,002	0,957	0,981	0,729	0,735	0,012	117,338	85,832
ENSG00000177410.8	0,001	0,956	0,981	0,761	0,765	0,007	425,399	324,719
DPM1	0,001	0,955	0,981	0,513	0,516	0,008	379,545	195,191
SS18	0,001	0,958	0,982	0,285	0,283	-0,008	559,178	158,704
WDR26	0,001	0,96	0,983	0,177	0,178	0,007	1030,121	183,104
C6orf211	0,002	0,959	0,983	0,414	0,417	0,01	220,706	91,775
PRKAG2	0,003	0,959	0,983	0,091	0,092	0,013	536,956	48,925
VPS28	0,002	0,959	0,983	0,774	0,766	-0,013	87,669	67,525
PEX16	0,002	0,961	0,983	0,547	0,551	0,011	161,036	88,412

RHOT2	0,001	0,96	0,983	0,209	0,208	-0,008	667,081	139,332
FANCA	0,001	0,959	0,983	0,19	0,191	0,008	688,033	131,282
SAP30BP	0,001	0,959	0,983	0,229	0,228	-0,007	723,395	165,194
ZNFX1	0,002	0,96	0,983	0,07	0,071	0,009	1503,604	105,993
MED18	0,003	0,962	0,984	0,45	0,446	-0,013	103,881	46,538
OSBPL9	0,002	0,965	0,984	0,104	0,105	0,007	1013,531	106,132
ATP5F1	0,001	0,964	0,984	0,702	0,704	0,004	923,145	648,72
IPO9	0,001	0,962	0,984	0,169	0,168	-0,005	2149,003	362,037
NSL1	0,002	0,966	0,984	0,283	0,285	0,01	213,81	60,757
SNRNP27	0,002	0,966	0,984	0,287	0,288	0,008	316,965	91,12
COMMD8	0,002	0,965	0,984	0,61	0,614	0,009	139,787	85,484
SRFBP1	0,003	0,963	0,984	0,186	0,184	-0,012	258,02	47,704
CRCP	0,003	0,964	0,984	0,106	0,105	-0,011	476,605	50,287
ERLIN2	0,001	0,964	0,984	0,239	0,238	-0,007	634,023	151,501
NOL6	0,001	0,964	0,984	0,128	0,128	-0,006	1812,893	232,158
CDK9	0,001	0,962	0,984	0,292	0,29	-0,007	652,434	189,848
GDI2	0,001	0,962	0,984	0,233	0,232	-0,005	2150,262	500,303
PTER	0,002	0,964	0,984	0,167	0,166	-0,008	516,26	85,849
APBB1IP	0,001	0,961	0,984	0,193	0,194	0,007	702,731	135,692
MINPP1	0,001	0,964	0,984	0,563	0,566	0,008	239,209	135,053
HPS1	0,003	0,963	0,984	0,132	0,131	-0,011	426,436	56,156
SWAP70	0,002	0,961	0,984	0,087	0,087	-0,01	861,268	74,924
ENSG00000239002.2	0,003	0,964	0,984	5,288	5,194	-0,026	8,948	46,74
SETD1B	0,002	0,963	0,984	0,131	0,131	0,009	561,889	73,545
MNAT1	0,001	0,965	0,984	0,316	0,318	0,007	384,772	122,089
PARN	0,003	0,964	0,984	0,146	0,145	-0,011	347,458	50,549
TAOK1	0,002	0,962	0,984	0,039	0,039	0,011	1509,468	59,002
NDUFS7	0,001	0,964	0,984	1,153	1,149	-0,006	266,098	306,322
CHAF1B	0,002	0,962	0,984	0,179	0,18	0,009	487,798	87,453
TRMT2A	0,002	0,965	0,984	0,167	0,169	0,009	415,024	69,722
MPZL1	0,001	0,968	0,985	0,136	0,136	-0,006	1030,912	140,208
CD46	0,001	0,968	0,985	0,308	0,306	-0,005	814,11	249,964

RAB3GAP2	0,002	0,969	0,985	0,099	0,099	-0,007	1042,639	103,277
VAMP8	0,001	0,967	0,985	0,429	0,427	-0,007	329,101	140,925
MARS2	0,002	0,97	0,985	0,308	0,306	-0,007	306,034	93,944
ERBB2IP	0,001	0,968	0,985	0,103	0,103	0,004	3323,974	343,471
MRPL14	0,001	0,967	0,985	0,79	0,793	0,006	272,649	215,762
MICU1	0,002	0,966	0,985	0,186	0,185	-0,007	580,477	107,588
SEC24C	0,001	0,968	0,985	0,201	0,201	0,004	2582,032	519,213
PDZD8	0,002	0,97	0,985	0,05	0,05	0,008	1172,421	58,544
UEVLD	0,003	0,97	0,985	0,179	0,18	0,009	318,729	57,132
STXBP6	0,002	0,97	0,985	0,25	0,251	0,008	298,555	74,742
FKBP3	0,002	0,97	0,985	0,436	0,433	-0,007	237,292	103,104
EIF2AK4	0,002	0,969	0,985	0,124	0,123	-0,006	921,768	114,098
BSG	0,001	0,969	0,985	0,517	0,518	0,002	7863,116	4068,159
MEX3D	0,003	0,967	0,985	0,206	0,204	-0,01	266,016	54,572
PRDX2	0,001	0,969	0,985	0,842	0,845	0,005	445,755	376,16
PLS3	0,002	0,969	0,985	0,201	0,203	0,009	316,762	63,933
NSUN2	0,001	0,971	0,986	0,167	0,166	-0,003	2297,854	382,535
WDR34	0,001	0,971	0,986	0,271	0,271	0,005	927,707	251,349
ENSG00000215417.6	0,002	0,97	0,986	3,834	3,802	-0,012	33,956	129,442
TIMM22	0,001	0,971	0,986	0,215	0,214	-0,006	644,78	138,302
DRG2	0,001	0,971	0,986	0,264	0,265	0,005	812,846	214,927
KRT10	0,002	0,971	0,986	0,647	0,644	-0,007	163,529	105,611
ARFGEF2	0,001	0,971	0,986	0,13	0,129	-0,005	1527,017	197,411
APOBEC3C	0,002	0,972	0,986	0,151	0,152	0,007	474,947	71,832
PQLC2	0,003	0,974	0,987	0,432	0,43	-0,009	133,321	57,479
HEBP1	0,001	0,972	0,987	0,565	0,567	0,004	523,655	296,245
CCT2	0,001	0,973	0,987	0,507	0,506	-0,003	2771,92	1403,188
COMT	0,001	0,974	0,987	0,514	0,513	-0,003	847,435	434,879
SZT2	0,002	0,975	0,988	0,133	0,133	0,007	552,86	73,427
SGOL2	0,002	0,975	0,988	0,06	0,06	-0,007	1065,191	63,549
SH3RF1	0,003	0,975	0,988	0,133	0,132	-0,008	361,276	47,822
PTPN3	0,003	0,975	0,988	0,105	0,104	-0,008	421,445	43,932

DERA	0,001	0,975	0,988	0,426	0,425	-0,005	349,398	148,612
SEC62	0,002	0,977	0,989	0,118	0,118	0,005	989,449	116,844
GTF3C6	0,003	0,977	0,989	0,272	0,273	0,008	166,715	45,435
RBM33	0,002	0,976	0,989	0,101	0,101	0,006	676,703	68,204
EIF4EBP1	0,001	0,976	0,989	0,387	0,386	-0,003	750,723	289,962
NCOA2	0,003	0,977	0,989	0,125	0,124	-0,006	479,096	59,732
PDP1	0,002	0,977	0,989	0,243	0,242	-0,005	398,899	96,735
MTSS1L	0,003	0,976	0,989	0,111	0,111	0,007	501,933	55,602
ARHGEF11	0,002	0,979	0,99	0,088	0,089	0,005	896,084	79,233
ATF2	0,002	0,978	0,99	0,185	0,184	-0,005	539,04	99,484
NDUFS4	0,001	0,979	0,99	1,301	1,297	-0,004	205,104	266,405
CTNNA1	0,001	0,978	0,99	0,098	0,097	-0,003	4502,771	439,188
RUFY1	0,001	0,98	0,99	0,24	0,24	-0,004	558,674	133,983
TNFRSF21	0,001	0,98	0,99	0,168	0,168	0,003	1396,499	235,019
SFXN4	0,001	0,98	0,99	0,497	0,499	0,004	303,817	151,34
CAND1	0,001	0,979	0,99	0,153	0,153	-0,003	1823,516	278,566
DCAF15	0,001	0,98	0,99	0,202	0,202	-0,004	732,099	147,792
PEPD	0,001	0,979	0,99	0,352	0,353	0,003	636,077	224,342
ST6GALNAC5	0,003	0,982	0,991	0,293	0,291	-0,007	152,978	44,66
TMEM18	0,001	0,982	0,991	0,377	0,378	0,004	365,258	137,748
MLTK	0,001	0,981	0,991	0,098	0,098	-0,003	1993,808	196,062
RBM5	0,003	0,981	0,991	0,062	0,061	-0,005	767,561	47,186
ARF5	0,001	0,981	0,991	0,54	0,539	-0,004	286,063	154,312
DLC1	0,001	0,981	0,991	0,124	0,123	-0,004	1095,759	135,494
RABGAP1	0,002	0,982	0,991	0,161	0,16	-0,004	553,065	88,78
GIPC1	0,001	0,981	0,991	0,199	0,199	0,003	1137,32	226,167
CNOT10	0,001	0,983	0,992	0,241	0,24	-0,003	550,704	132,5
CCDC50	0,002	0,984	0,992	0,185	0,185	-0,004	491,908	91,075
TJAP1	0,003	0,984	0,992	0,132	0,133	0,005	403,022	53,373
NUB1	0,003	0,985	0,992	0,111	0,11	-0,005	426,185	47,095
PHRF1	0,002	0,983	0,992	0,062	0,062	0,004	1154,337	71,22
FERMT2	0,001	0,985	0,992	0,119	0,118	-0,002	1612,976	191,052

FAHD1	0,003	0,985	0,992	0,489	0,487	-0,005	106,241	51,822
USP7	0,001	0,983	0,992	0,113	0,113	0,003	1637,026	184,375
DDX5	0,002	0,984	0,992	0,148	0,148	0,004	636,91	94,295
AAR2	0,001	0,984	0,992	0,214	0,213	-0,003	802,396	171,307
LZTS2	0,003	0,985	0,993	0,106	0,106	-0,005	392,794	41,755
NPM3	0,001	0,987	0,994	0,498	0,498	-0,002	520,546	259,198
TMEM216	0,003	0,987	0,994	0,263	0,264	0,004	162,046	42,632
LEPROT	0,002	0,988	0,995	0,113	0,113	0,003	989,154	112,084
FZD7	0,001	0,989	0,995	0,29	0,29	0,002	458,619	133,104
GALNT10	0,002	0,989	0,995	0,064	0,064	-0,003	1116,958	71,526
CDK10	0,003	0,988	0,995	0,087	0,087	-0,004	505,515	44,093
DDX39A	0,001	0,989	0,995	0,454	0,454	0,001	1881,27	854,528
PURA	0,001	0,991	0,996	0,386	0,387	0,002	436,209	168,685
VPS41	0,003	0,991	0,996	0,106	0,106	-0,002	510,13	53,995
GAPDH	0,001	0,991	0,996	0,452	0,452	0,001	9614,227	4345,45
KNSTRN	0,001	0,991	0,996	0,342	0,341	-0,001	670,052	228,894
OIP5	0,002	0,99	0,996	0,683	0,684	0,003	101,579	69,427
ARMC7	0,003	0,991	0,996	0,298	0,298	-0,002	169,548	50,541
PSMC4	0,001	0,991	0,996	0,63	0,631	0,001	1128,25	711,573
ATP13A2	0,002	0,993	0,997	0,127	0,126	-0,001	1016,528	128,593
LRRC42	0,001	0,996	0,997	0,346	0,346	0,001	527,985	182,779
LRIG2	0,002	0,997	0,997	0,252	0,252	-0,001	300,255	75,789
CCDC88A	0,002	0,996	0,997	0,071	0,071	-0,001	1150,658	81,327
EVA1A	0,001	0,994	0,997	0,301	0,301	0,001	541,374	163,095
RFT1	0,002	0,994	0,997	0,407	0,408	0,002	191,257	77,928
DIAPH1	0,001	0,993	0,997	0,098	0,098	-0,001	3472,422	340,974
RNF14	0,003	0,995	0,997	0,174	0,174	0,001	298,304	51,897
C6orf89	0,002	0,996	0,997	0,135	0,135	0,001	620,698	83,935
ABCB8	0,003	0,994	0,997	0,226	0,226	0,002	214,928	48,515
RHNO1	0,002	0,995	0,997	0,102	0,102	-0,001	592,674	60,69
PCBP2	0,001	0,997	0,997	0,164	0,164	0	1231,316	201,967
GNS	0,001	0,992	0,997	0,301	0,301	0,001	1344,99	405,342

IRF2BPL	0,002	0,996	0,997	0,175	0,175	0,001	445,433	77,918
CASC5	0,002	0,997	0,997	0,069	0,069	-0,001	1437,083	98,555
ADCY7	0,002	0,992	0,997	0,2	0,2	0,002	328,141	65,627
TMEM208	0,001	0,995	0,997	1,017	1,016	-0,001	157,363	160,018
PLA2G15	0,003	0,993	0,997	0,234	0,234	0,002	209,247	48,963
VPS25	0,001	0,996	0,997	0,523	0,523	0,001	605,044	316,604
PSMD12	0,001	0,994	0,997	0,304	0,304	-0,001	1395,938	423,982
AFMID	0,002	0,996	0,997	0,412	0,412	-0,001	186,03	76,682
SYDE1	0,001	0,992	0,997	0,125	0,125	0,002	1455,168	182,246
SYMPK	0,001	0,994	0,997	0,163	0,163	0,001	1873,944	305,338
NCOA6	0,001	0,992	0,997	0,128	0,128	0,002	1336,884	170,825
HPRT1	0,001	0,993	0,997	0,669	0,669	0,001	352,049	235,552
MECOM	0,003	0,998	0,998	0,101	0,101	-0,001	473,974	47,724
SRSF3	0,001	0,997	0,998	0,37	0,37	0	1884,734	696,53
NME3	0,003	0,997	0,998	0,856	0,857	0,001	64,108	54,886
RASAL2	0,002	0,999	0,999	0,076	0,076	0	919,264	69,95
ORC4	0,002	1	1	0,243	0,243	0	283,48	68,836
